# Comparative transcriptome analysis during early fruit development between three seedy citrus genotypes and their seedless mutants

**DOI:** 10.1038/hortres.2017.41

**Published:** 2017-09-13

**Authors:** Shujian Zhang, Qingchun Shi, Ute Albrecht, Robert G Shatters, Ric Stange, Greg McCollum, Shuo Zhang, Chengming Fan, Ed Stover

**Affiliations:** 1 U.S. Horticultural Research Laboratory, USDA-ARS, Ft. Pierce, FL 34945, USA; 2 Institute of Genetics and Developmental Biology, Chinese Academy of Sciences, Beijing 100101, China

## Abstract

Identification of genes with differential transcript abundance (GDTA) in seedless mutants may enhance understanding of seedless citrus development. Transcriptome analysis was conducted at three time points during early fruit development (Phase 1) of three seedy citrus genotypes: Fallglo (Bower citrus hybrid (*Citrus reticulata*×*C. reticulata*×*C. paradisi*)×Temple (*C. reticulata*×*C. sinensis*)), grapefruit (*C. paradisi*), Pineapple sweet orange (*C. sinensis*), and their seedless mutants. Seed abortion in seedless mutants was observed at 26 days post anthesis (Time point 2). Affymetrix transcriptomic analysis revealed 359 to 1077 probe sets with differential transcript abundance in the comparison of seedless versus seedy fruits for each citrus genotypes and time points. The GDTA identified by 18 microarray probe sets were validated by qPCR. Hierarchical clustering analysis revealed a range of GDTA associated with development, hormone and protein metabolism, all of which may reflect genes associated with seedless fruit development. There were 14, 9 and 12 genes found exhibiting similar abundance ratios in all three seedless versus seedy genotype comparisons at time point 1, 2 and 3, respectively. Among those genes were genes coding for an aspartic protease and a cysteine protease, which may play important roles in seedless fruit development. New insights into seedless citrus fruit development may contribute to biotech approaches to create seedless cultivars.

## Introduction

Citrus is the most widely cultivated fruit in the world.^1^ Seedlessness is an important trait in relation to fruit quality, and consumers’ interest in seedless citrus such as oranges, mandarins and lemons has increased.^2^ Therefore, breeding seedless citrus varieties is a major objective. Seedless citrus fruits can be obtained through parthenocarpy, stenospermocarpy, male or female sterility, self-incompatibility, abnormal embryo sacs and unfertilized ovules, and various factors that result in meiotic irregularities.^3–6^ Parthenocarpy indicates production of fruit without fertilization or embryo abortion. In the absence of pollination, parthenocarpic plants will set seedless fruit.^7^ Parthenocarpy has a genetic basis and hence can be targeted for genetic engineering of seedlessness.^8^ For example, RNA interference (RNAi)-mediated suppression of chalcone synthase, the first enzymatic step in the flavonoid pathway, resulted in parthenocarpic tomato (*Solanum lycopersicum*) fruits.^9^ Parthenocarpic fruit development was also seen in tobacco (*Nicotiana tabacum*) and eggplant (*Solanum melongena*) expressing the coding region of the *iaaM* gene (encoding tryptophan monooxygenase involved in biosynthesis of indol-3-acetic acid, an auxin class plant hormone) from *Pseudomonas syringae pv. savastanoi,* under control of the promoter of *DefH9* (*Deficiens* homologue 9, *Antirrhinum majus*).^10^ The *DefH9::iaaM* expression promotes the synthesis of auxin (IAA) specifically in the placenta, ovules and tissues derived therefrom.^11^ The agronomical advantages of *DefH9::iaaM* genetically modified plants have been assessed in greenhouse and field trials using transgenic eggplant,^12^ strawberry and raspberry ^13^ and cucumber (*Cucumis sativus*).^14^ Goetz *et al.*^15^ demonstrated that AUXIN RESPONSE FACTOR 8 (ARF8) is a negative regulator of fruit initiation in the absence of fertilization in *Arabidopsis*. Parthenocarpy was also induced in *Arabidopsis* and tomato through constitutive expression of the mutant genomic (g) *Atarf8-4* sequence ^16^ or ovary specific expression of a tyrosine phosphatase *RolB* gene (proposed as an activator of an array of secondary metabolic processes).^17^ Accordingly, parthenocarpy can be induced in a variety of agricultural species by the exogenous application of auxins, cytokinins, or gibberellin,^18,19^ indicating that a number of independent and possibly redundant hormone pathways can direct parthenocarpy. In citrus, parthenocarpy occurs naturally in Navel orange (*C. sinensis*) and Satsuma mandarin (*C. unshiu* Marc.). Only in ‘Wilking’ mandarin hybrid has a gene been reported to cause parthenocarpy-related seed abortion, and it was a recessive gene responsible for asynapsis in meiosis.^20^

Comparing GDTA between seedless and seedy variants during development of fruitlets can provide insight into molecular mechanism of seed formation. These genes would then be potential targets for inducing seedlessness using gene knockout or overexpression. The chloroplast chaperonin 21 (*ch-Cpn21*) gene has higher expression during flowering in seedy compared to seedless Thompson grapes and silencing of *ch-Cpn21* in tobacco and tomato resulted in seed abortion.^21^ The ubiquitin extension protein S27a is differentially expressed in developing flower organs of Thompson seedless versus seedy grape isogenic clones. Overexpression of ubiquitin extension protein S27a gene in carpels and integuments led to embryo abortion and seedlessness.^22^ A male sterility-like gene was identified in ‘Ponkan’ mandarin and its transcript is more abundant in seedless mutants than in the seedy progenitor.^23^ This gene codes for a fatty acyl-CoA reductase involved in lipid metabolism. A comparative analysis of miRNAs between a fertile line and male sterile cybrid line of pummelo (*Citrus grandis*) revealed that miR167a was involved in floral bud development and cytoplasmic male sterility in citrus.^24^ The genes associated with male sterility or parthenocarpy have been exploited in efforts for seedlessness development in citrus. For example, the chimeric barnase gene, a ribonuclease derived from *Bacillus amyloliquefaciens*, was introduced into embryogenic callus of ‘Ponkan’ mandarin although there were no further reports about the outcomes.^25^ The *Arabidopsis thaliana MAC12.2* gene, associated with male sterility and parthenocarpy, was introduced into precocious trifoliate orange (*Poncirus trifoliata*) and resulted in fruits with reduced number of seeds.^26^

‘Fallglo’ mandarin hybrid (*Citrus reticulata*) is an early-maturing, seedy mandarin-type derived from ‘Bower’ **×** ‘Temple’.^27^ ‘Duncan’ grapefruit (*C. paradisi*) and ‘Pineapple’ sweet orange (*C. sinensis*) are seedy citrus varieties grown in Florida. Numerous seedless grapefruit varieties grown are ultimately derived from seedy grapefruit similar to ‘Duncan’ through selection of natural or induced mutants, and all true grapefruit are near isogenic.^28^ ‘US Early Pride’ and ‘US Seedless Pineapple’ are released cultivars from irradiated ‘Fallglo’ and ‘Pineapple’ sweet orange, respectively.^29,30^ In this study, we compared the transcriptome profiles of three seedy citrus genotypes: ‘Fallglo’ mandarin hybrid, ‘Pineapple’ sweet orange and ‘Duncan’-like seedy grapefruit and associated seedless mutants during early fruit development (Phase 1).^31^ The objective of this work was to identify GDTA in seedless versus seedy fruits (gene expression in seedless relative to seedy will be used in all comparisons unless otherwise stated) at three time points across all three citrus genotypes. Ultimately, it is hoped the genes identified will be useful in development of seedless variants of desirable but seedy citrus varieties.

## Materials and methods

### Citrus genotypes and sampling

Three genotypes were compared in this experiment for which seedy progenitors and their seedless mutants were available. The genotypes used were: Fallglo and Seedless Fallglo (US Early Pride); Pineapple and US Seedless Pineapple; and individual trees of three seedy true grapefruit (Duncan, Hudson, Inman Late) and individual trees of three seedless mutants (Henderson, Marsh, and Redblush). Three trees of any single named grapefruit cultivar were not available at the same location as the other genotypes. However, grapefruit are near-isogenic and therefore three seedy and seedless cultivars represent reasonable biological replicates. Controlled crosses were made on 10 flowers per tree and monitored for fruit growth, to provide reference fruit sizes for approximate days from pollination in material collected for RNA extraction. Three trees of each seedy and seedless genotype were sampled on 17 April 2009 (time point 1), 4 May 2009 (time point 2) and 28 May 2009 (time point 3). On each date from each tree 12–25 fruits were collected. Several fruits were assessed for seed abortion, while the remainder were subjected to RNA extraction from entire fruits. At harvest, fruits were wrapped in aluminum foil, immediately frozen in liquid nitrogen and stored at −80 °C for RNA extraction. Fruits at each time point from each tree were assessed for ovule development by hand-sectioning and Toluidine Blue staining.

### RNA extraction and microarray hybridization

RNA extractions were performed using the Guanidinium thiocyanate procedure^32^ and pellets were resuspended in 500 μl RNase-free H_2_O. RNA was purified using MinElute purification and was eluted in 30 μl RNase-free H_2_O, followed by DNase treatment (final volume 45 μl). RNA samples were pooled for the three trees of each seedy or seedless variant of each citrus genotype for each sampling date. The concentration of RNA was measured in a NanoDrop ND-1000 spectrophotometer (NanoDrop Technologies, Wilmington, DE, USA). RNA quality was evaluated using an Agilent Bioanalyzer Model 2100 (Agilent Technologies, Palo Alto, CA, USA).

Hybridization were performed using the Affymetrix Citrus GeneChip (Affymetrix, Santa Clara, CA, USA) at the Interdisciplinary Center for Biotechnology Research Microarray Core, University of Florida, Gainesville, according to the manufacturer’s instructions. The GeneChip Citrus Genome Array contains 30,171 probe sets representing up to 33,879 citrus transcripts, and 5023 SNP probe sets.

### Data analysis

The signal intensities were determined with a GeneChip scanner controlled by GCOS (GeneChip Operating Software, Santa Clara, CA, USA) Affymetrix software. Then we used the GCOS software to analyze fluorescence readings with Robust multiarray analysis (RMA). Criteria for inclusion in further analysis were that the probes display fluorescence significantly above background and that they had an absolute reading of 20 or greater. The signal ratio between seedless and seedy was calculated to represent the abundance ratio for each probe set. The differentially expressed probes between seedless versus seedy citrus were chosen with the criterion of *P*⩽0.05 and false discovery rate (FDR)⩽0.05, in combination with a cutoff value of abundance ratio >2 or<−2.

Further analysis was conducted using the corresponding *Arabidopsis* orthologs of differentially expressed probe set IDs on the Citrus GeneChip by searching the *Arabidopsis thaliana* genome via the HarvEST database.^33^ In some cases, several probe set IDs were annotated to the same *Arabidopsis* gene. The average fold change of probe IDs representing the same gene was calculated and used for Venn diagram and MapMan pathway analysis. We used MapMan to locate the genes with differential transcript abundance (GDTA) in the metabolic network.^34^ Hierarchical Cluster Analysis of GDTA transcription profiles was achieved with Permutmatrix software (http://www.atgc-montpellier.fr/permutmatrix; V1.9.3) with Euclidian distance and McQuitty's linkage WPGMA method.^35^

### Microarray validation by real-time qPCR (RT-qPCR)

The same RNA samples used for microarray experiments were also used for qPCR. The cDNA synthesis was performed following the protocol as described.^36^ RT-qPCR was performed using an ABI7500 thermal cycler (Thermo Fisher Scientific, Waltham, MA, USA) with Bright Green PCR Master Mix (Sigma-Aldrich, St Louis, MO, USA). Melting cures and electrophoresis were performed to verify the specificity and identify of the PCR products. Citrus 5.8s rRNA gene was used for internal control as described^71^ and 23 genes were selected for validation. The relative quantification of gene expression level was determined by the comparative C_t_ method 2^−ΔΔCt;^,^37^ where ΔΔCt=(Ct (target gene)—Ct (internal control))_seedless_—[Ct (target gene)—Ct (internal control)]_seedy_.

## Results

### Phenotype observance during early fruit development

**Figure 1:**
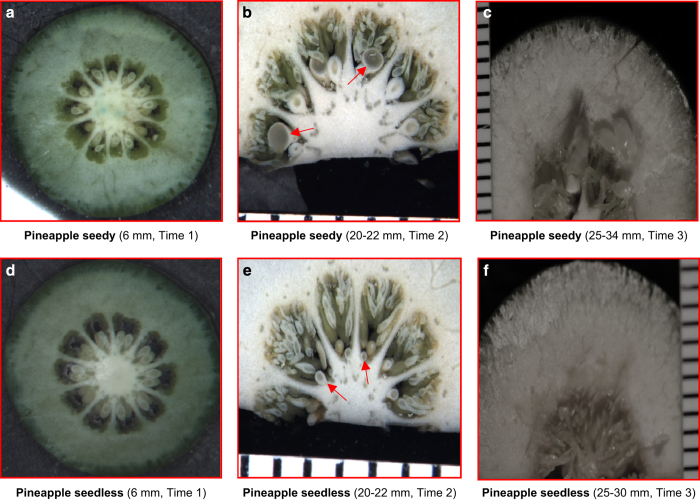
Seed development in seedy and seedless Pineapple sweet orange at 9 days (**a** and **d**), 26 days (**b** and **e**) and 50 days (**c** and **f**) post anthesis. The distance between black lines in rulers of (**b**, **c**, **e** and **f**) was 1 mm and the sizes of fruits at each time point are shown under each panel. The red arrows in (**b**) indicate the developing seeds and the red arrows in (**e**) indicate the aborted seeds.

The fruit samples were collected at 9 days, 26 days and 50 days post anthesis, with the time points selected to bracket expected time of embryo abortion based on an earlier report in seedless grape.^38^ Seed abortion was apparent in the seedless mutants of all three genotypes by the second time point, which was about 4 weeks after anthesis (Figure 1e; only Pineapple sweet orange shown, but the other two genotypes were similar) and is consistent with another report for seedless citrus.^39^

### Overview of microarray data

**Figure 2:**
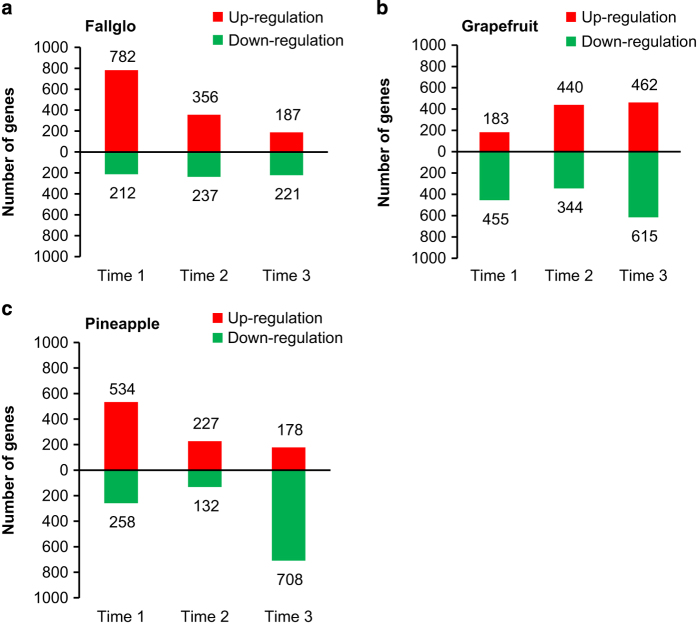
Numbers of probes with differential transcript abundance (PDTA) in seedless versus seedy fruits at each of three time points in Fallglo tangerine (**a**), grapefruit (**b**) and Pineapple sweet orange (**c**). The red bars represent the number of genes with higher transcript abundance in the seedless fruits in comparison with the seedy fruits (seedless/seedy abundance ratio >2). The green bars represent the number of genes with lower transcript abundance from the same comparison (seedless/seedy abundance ratio <−2).

Transcript abundance was quantified in the fruits of three seedy citrus genotypes and their seedless mutants at three times after anthesis using the Affymetrix GeneChip Citrus Genome Array, and those meeting our criteria where defined as probes with differential transcript abundance (PDTA). PDTA at time point 1, 2 and 3, respectively were: 994, 593 and 408 probe sets in Fallglo; 638, 784, and 1077 probes sets in grapefruit; and 792, 359 and 886 probe sets in Pineapple sweet orange (Figure 2). The detailed transcript abundance data is attached as Supplementary Tables S1–S9. In comparison between seedless and seedy fruits, Fallglo had 78.7%, 60% and 45.8% of PDTA probe set IDs exhibiting higher transcript abundance at time points 1, 2 and 3, respectively. These values were 28.7, 56.1 and 42.9% of PDTA in grapefruit and 67.4, 63.2 and 20% in Pineapple.

### Validation of microarray data with RT-qPCR

**Figure 3:**
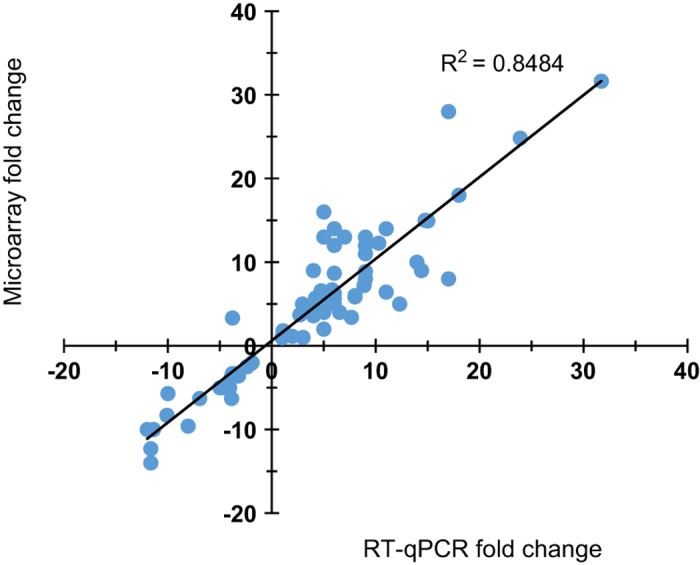
The correlation between microarray data and RT-qPCR data. Eighty-four data points from different time points of three citrus genotypes data sets were validated by RT-qPCR. The abundance ratio values of gene transcript abundance in seedless versus seedy from array were plotted against that of the qPCR results. A correlation coefficient of 0.84 was obtained.

A total of 18 probe set IDs were selected for reverse-transcription qPCR (RT-qPCR) validation and are listed in Supplementary Table S10. The probe sets selected were based on putative gene functions that may be associated with seedless fruit development. These included genes for an aspartic protease, a cysteine protease, a GDSL lipase, a gibberellin-responsive (GASA1) protein, a group 4 late embryogenesis abundant (LEA) domain-containing protein, an indole-3-acetic acid synthase and two transcription factors. The expression data of RT-qPCR and microarray measurements for the selected genes are presented in Supplementary Table S10. The sequences of primers used for RT-qPCR analysis is listed in Supplementary Table S11. In total 84 data points from three citrus genotypes at different time points were obtained from RT-qPCR. Abundance ratios of gene transcripts between seedless and seedy fruits from microarray were plotted against the ratios from the RT-qPCR results, and a high correlation (*R*^2^=0.84) was obtained (Figure 3). In addition, all the genes except one, Cit.27555.1.S1_at at time pint 1 in grapefruit, exhibited the same expression trend at each time point across citrus genotypes between these two methods, confirming that the differentially expressed genes identified in the microarray accurately reflected the transcriptomes.

### Transcriptional profiles of gene transcripts in seedless versus seedy fruits

The probe sets in the Affymetrix Citrus Genechip were annotated by searching the *Arabidopsis thaliana* genomic data. In the MapMan analysis, we used the corresponding *Arabidopsis thaliana* Genome Initiative IDs (AtGID) of *Arabidopsis* orthologs instead of the citrus probe set IDs. It is noteworthy that not all the probes set IDs have AtGID annotations and one AtGID could cover multiple probe set IDs. In our analysis, we chose probes with matching AtGIDs and when multiple probes matched the same AtGID, we calculated the average abundance ratio. A summary of the number of matched GDTA in seedless versus seedy fruits at each time point of the three citrus genotypes are presented in Table 1. For consistency, discussion of GDTA pertains to seedless relative to seedy in this paper. These GDTA between seedless and seedy were used for subsequent MapMan analysis, Venn diagrams and hierarchical clustering.

**Figure 4:**
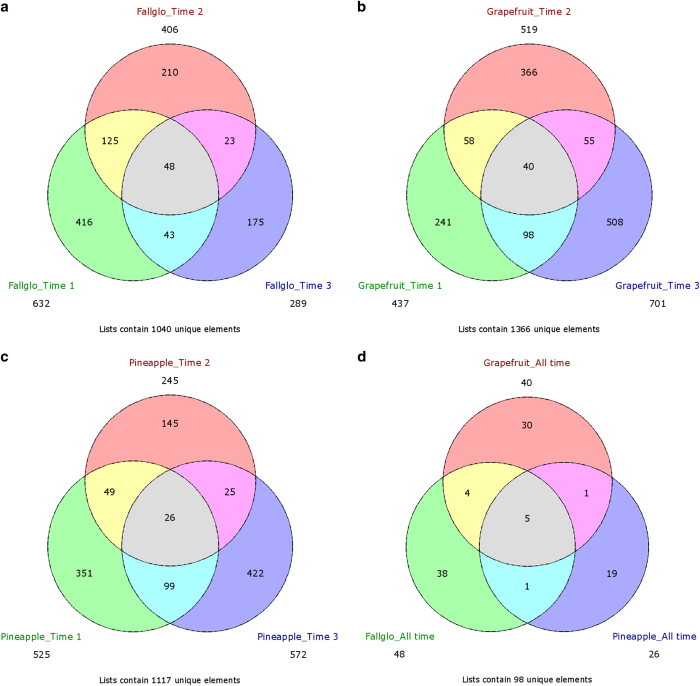
Venn diagrams indicating the overlaps of genes with differential transcript abundance (GDTA) in seedless versus seedy fruits between the three time points in Fallglo tangerine (**a**), grapefruit (**b**) and Pineapple sweet orange (**c**). Subsequently, GDTA obtained from each citrus genotype were used for further discovery of overlaps across genotypes (**d**).

**Figure 5:**
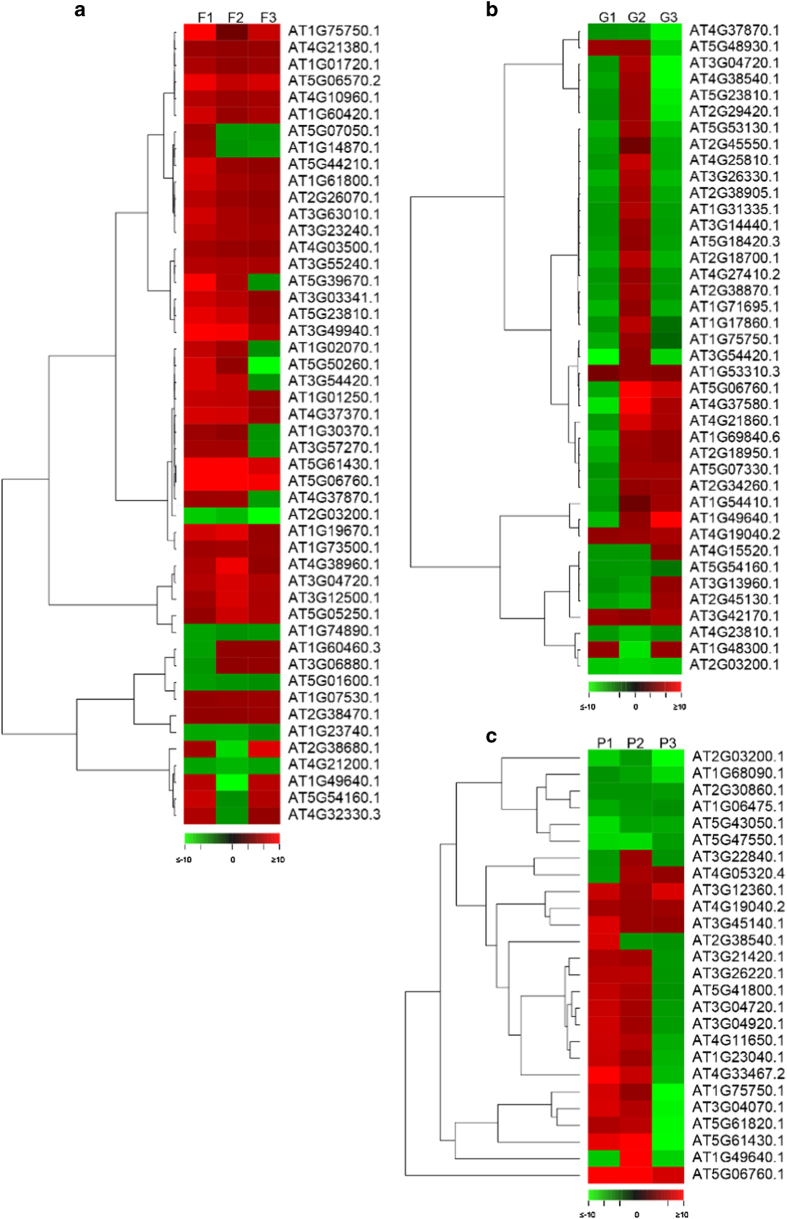
Expression heat maps of GDTA observed at all three time points in Fallglo tangerine (**a**), grapefruit (**b**) and Pineapple sweet orange (**c**). A hierarchical clustering analysis of GDTA observed at three time points of Fallglo (F1–F3), grapefruit (G1–G3) and Pineapple (P1–P3) using a Pearson correlation was computed by PermutMatrix v1.9.3. The abundance ratio of GDTA is displayed as illustrated in the color bar at the bottom of each panel. Green is for GDTA with lower transcript abundance and red is for GDTA with higher transcript abundance. White square means the corresponding gene didn’t exhibit differential transcript abundance at indicated time point.

The GDTA detected at each time point for each of the three citrus genotypes were imported into MapMan software for metabolic pathway analysis. The MapMan metabolism overview of each set of GDTA are shown in Supplementary Figures S1–S9. These genes were assigned as related to cell organization, development, hormone metabolism, protein metabolism, RNA regulation, secondary metabolism, signaling transduction, biotic stress and transporters. Venn diagram analysis showed that there were 48, 40 and 26 GDTA detected at all three time points in seedless versus seedy fruits of Fallglo, grapefruit and Pineapple, respectively (Figures 4a–c). MapMan metabolic pathway analysis of the above three sets of genes is presented in Supplementary Tables S12–S14. Gene sets associated with the same metabolic pathway within and across citrus genotypes were explored. For instance, there were 5, 3 and 2 GDTAs associated with hormone metabolism in Fallglo, Grapefruit and Pineapple, respectively (Supplementary Tables S12–S14), and one common GDTA encoding GAST1 protein homolog 1 was found among the three citrus genotypes. In addition, hierarchical clustering analysis of transcription profiles in seedless versus seedy fruits of each citrus genotype was computed by PermutMatrix 1.9.3. Heat maps of GDTA detected at all three time points in each citrus genotypes were shown in Figure 5. In Fallglo, 48 genes were grouped into 9 clusters (Figure 5a). Each cluster contains the genes with similar up- and down-regulation pattern over the three time points. In grapefruit and Pineapple, 7 and 5 gene clusters were grouped based on their expression patterns. The heat maps of GDTA detected at any two time points in each citrus genotype are presented in Supplementary Figures S10–S12. Taken together, these results showed that the majority of GDTA identified by Venn diagram are genotype-specific and showed differential expression profiles across the three time points studied.

However, 5 GDTAs were common across all three time points and across all three citrus genotypes (Figure 4d), and their transcript abundance ratio data are presented in Table 2. Noteworthy was that a gene homologous to the *Arabidopsis* aspartic protease gene (AT2G03200) exhibited lower transcript abundance at all time points and in all seedless genotypes compared with seedy varieties, strongly suggesting its involvement in seed development. A gene coding for a group 4 late embryogenesis abundant (LEA) domain-containing protein exhibited higher transcript abundance in all of the seedless genotypes at all tested time points and its expression peaked at time point 2, except for grapefruit where it showed lower transcript abundance at time point 1. Three genes have different expression patterns among different citrus, suggesting genetic variations between the genotypes may differentially regulate the timing of the gene induction related to seedless fruit development. These genes included a gene coding for GASA1 protein homolog 1, a gibberellic acid (GA)-responsive protein, which exhibited higher transcript abundance at all three time points in Fallglo, at time point 2 in Grapefruit and at time point 1 and 2 in Pineapple, respectively. A gene coding for a protein homologous to pathogenesis-related protein 4 (PR4) was found to have a regulation pattern identical to the GASA1 gene. Last, a gene coding for a hydrolase with unknown specific biological function showed differential transcript abundance at all time points.

### GDTA at each time point across all citrus genotypes

**Figure 6:**
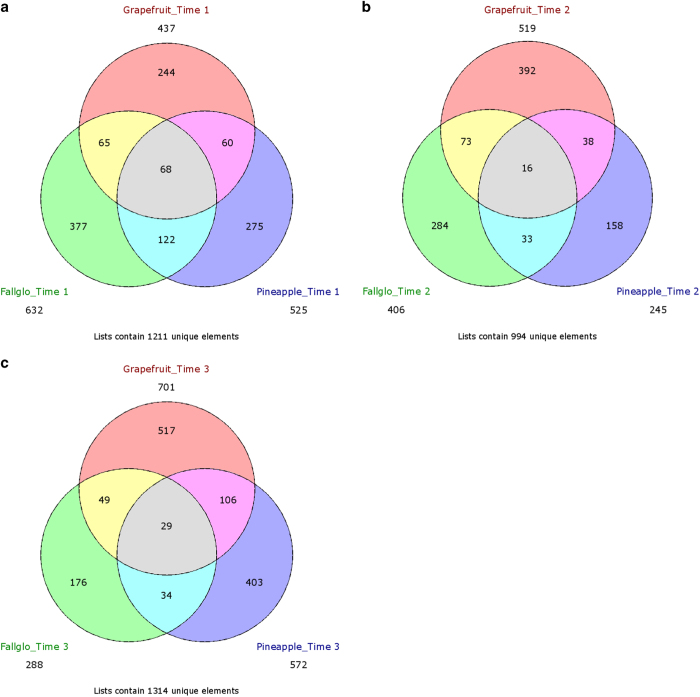
Venn diagrams indicating the overlaps of GDTA between each citrus genotype at Time point 1 (**a**), Time point 2 (**b**) and Time point 3 (**c**).

The genes associated with seedless fruit development might be strictly regulated at certain times during early fruit development. At other time points, they may not be differentially expressed in seedless versus seedy fruits. As a result, the GDTA at each time point shared by three citrus genotypes, especially those with the same expression trend, indicate a high probability of being associated with seedless fruit development. As shown in Figure 1, seed abortion was observed at time 2 in Pineapple sweet orange seedless fruit, as well as in Fallglo and grapefruit seedless fruits (data not shown). To retrieve the genes with differential transcript abundance at each time point in all three citrus genotypes, we conducted Venn diagram analysis using the gene list from the same time point in each citrus genotypes. There were 68, 16 and 29 GDTA shared by all three citrus genotypes at time point 1, 2 and 3 respectively (Figures 6a–c). Out of the GDTA at each time point respectively, there were 14, 9 and 12 GDTA with similar transcript abundance patterns (Table 3).

Among the 68 GDTA shared across all three genotypes at time point 1, 9 genes exhibited higher transcript abundance and 5 genes exhibited lower transcript abundance across all three seedless genotypes (Table 3). Of the higher transcript abundance class, genes were identified that code for proteins homologous to 1) nitrate reductase (NIA2), involved in nitrate assimilation; 2) a glycosyltransferase involved in flavonoid metabolism; 3) two proteins involved in hormone metabolism, LOX2 (LIPOXYGENASE 2), involved in jasmonic acid biosynthesis, and P-GLYCOPROTEIN 13, an ATPase involved in auxin transport; 4) a cysteine protease; and 5) a germin-family like protein representing a family of proteins with a broad array of functions.^40^ Three other identified genes encoded proteins of unknown function. The five lower transcript abundance genes encoded: 1) GA2OX8 (GIBBERELLIN 2-OXIDASE, involved in gibberellin degradation; 2) a UDP-glucosyl transferase family protein involved in indole-3-acetic acid (IAA) homeostasis; 3) an aspartic protease; 4) *PGY2* (*PIGGYBACK2*) coding for a ribosomal structural constituent involved in development/pattern regulation, and 5) a GDSL-motif lipase family protein from a large family of proteins involved in plant development and morphogenesis.^41^ The common differential transcript levels of these genes at time point 1 in all three citrus genotypes suggested an involvement of nutrients, secondary metabolites, development regulatory processes and hormones at the early stage of seed abortion.

At time point 2, 7 genes exhibited higher transcript abundance and 2 exhibited lower abundance in seedless versus seedy fruits across three citrus genotypes (Table 3). Those genes with higher abundance include 3 genes involved in development. Two genes code for NAC transcription factors (transcriptional factors regulating genes involved in stress response and development), with domains homologous to *Arabidopsis* protein 100 (ANAC100) and protein 72 (ANAC72) and one gene encodes late embryogenesis abundant (LEA) group 4 protein. A gene that codes for GA-responsive (GASA1) protein exhibited higher transcript abundance in all three seedless citrus genotypes. Only two genes exhibited lower transcript abundance across three citrus genotypes at time point 2. One encodes for a homolog to GLYOXALASE I (ATGLX1) and the other for an aspartyl protease involved in proteolysis. These genes with similar transcript patterns across three citrus genotypes at this time point might be associated with the development of seedless fruit, but are unlikely to be the cause of seedlessness.

Twenty-nine GDTA were identified at time point 3 in the three citrus genotypes and 12 genes had similar seedless/seedy abundance ratios (Table 3). Interestingly, eleven genes exhibited higher transcript abundance and only one gene with lower abundance. Three genes encoding proteins involved in secondary metabolism (Cinnamoyl-CoA reductase, 2OG-Fe (II) oxygenase and cytochrome P450 monooxygenase CYP706A6). The large group of cytochrome P450 enzymes are involved in oxidation reactions. Interestingly, overexpression of a gene encoding cytochrome P450, CYP78A9, induces large but seedless fruit in *Arabidopsis*.^42^ Three proteins encoding genes involved in protein metabolism all exhibited lower abundance in all three seedless citrus genotypes at this time point. The citrus gene homologous to *Arabidopsis* cysteine protease1 (CEP1) that exhibited higher transcript abundance at time point 1 (Table 3), exhibited lower abundance at time point 3 in all three seedless citrus genotypes. This gene is involved in tapetal programmed cell death and pollen development and was reported to be upregulated in developing seeds. The only shared gene exhibiting higher transcript abundance at this time point codes for a LEA4 domain-containing protein and it also exhibited higher transcript abundance at time point 2 in all three citrus genotypes.

## Discussion

Citrus fruits that have no seeds, have only traces of aborted seeds or have a much-reduced number of seeds (less than five seeds) are considered commercially seedless.^8^ In all cases, commercially seedless citrus cultivars require both inability to produce seeds under the specific growing conditions and the ability of these seedless fruit to develop to maturity. Many seedless cultivars produce very low yields,^40^ but the three seedless mutants used in this study all bear quite well, suggesting expression of specific genes and resulting changes of metabolism that permit a high degree of seedless fruit production. Cytogenetic analysis of some naturally-occurring seedless cultivars and irradiation-induced mutants revealed that chromosomal structural changes disrupted meiosis and contributed to ovule abortion.^28^ It is well documented that seedless citrus fruit production is genotype-dependent with some genotypes producing no fruit without cross-compatible pollination.^7^ Even within a cultivar, environmental conditions^27^ or application of exogenous growth regulators influence the set of seedless fruit.^43^ Regardless of the fundamental cause of seedlessness, cultivars that produce high yields of seedless fruit may display common gene expression that contributes to the set and development of seedless fruit.

In this study, we conducted a comparative transcriptome analysis of early fruit development between three seedy citrus genotypes and their seedless mutants. A similar strategy was previously applied in omics based research on apomixis in citrus.^44^ Seedless versus seedy fruit development in each genotype may exploit some different genes however it seems likely that some key differences in gene expression are shared by different low-seeded genotypes although possibly at somewhat different time points and with different magnitude. By collecting fruits at the same time points, we may have captured different developmental stages from each genotypes. Regardless, seed abortion was observed at time point 2 for all genotypes (Figure 1), indicating the time points we selected were appropriate to study transcriptional reprogramming before and after ovule abortion for these genotypes.

There were 245 to 701 genes with differential abundance at different time points among different citrus genotypes (Figure 4). Venn diagram analysis showed that 48, 40 and 26 GDTA were shared across all three time points in seedless versus seedy Fallglo, grapefruit and Pineapple, respectively (Figure 4, Supplementary Tables S12–S14). These GDTA are mostly genotype-specific, as only 5 genes were found to be common among genotypes at all three time points (Figure 4d and Table 2). Moreover, the abundance ratios of many of the GDTA discovered from each seedless citrus genotype varied at the three time points (Figure 5). Since certain genes relating to seedless fruit development might be strictly regulated at certain time points during early fruit development, we set out to study the GDTA at each time point shared by three citrus genotypes.

It has been shown that auxins, cytokinins or gibberellin, can induce parthenocarpy in plants and that parthenocarpic tomatoes have higher endogenous levels of auxin and gibberellin than seedy types.^18,19^ In this work, several genes involved in hormone metabolism exhibited differential transcript abundance between seedless and seedy clones at time point 1. A gene homologous to *A. thaliana* (AtGA2ox8) involved in gibberellin degradation, exhibited lower abundance in seedless versus seedy variants in all citrus types. Overexpression of AtGA2ox8 reduced gibberellin levels in tobacco plants.^45,46^ The suppression of this gene homolog in citrus at this time point suggests that these seedless citrus genotypes may have elevated endogenous GA levels associated with seedless fruit development. Increase in endogenous GA levels have been observed in parthenocarpic Fino clementine^47^ and exogenous GA applications during early fruit development have been used routinely to enhance set of seedless citrus.^43^ It is possible that silencing citrus *GA2ox8* would further enhance seedless citrus fruit development. The transcript abundance of a gene homologous to lipoxygenases (LOX2) increased at time point 1. Several roles have been suggested for lipoxygenases in seeds: fatty acid peroxidation in membranes, seed-storage lipids and production of growth regulators including jasmonates and abscisic acid (ABA).^48^ The balance between GA and ABA appears to play an important role in the regulation of fruit development in seedless mandarins.^47^ Plants regulate auxin levels through complex interactions including the formation and hydrolysis of amide-linked conjugates that act as storage or inactivation forms of the hormone.^49^ The transcripts of a gene homologous to UDP glucosyltransferase decreased at time point 1 in all citrus genotypes. The UDP-glucosyltransferase catalytic enzyme has been reported to act on indole-3-butyric acid (IBA) as a substrate to perturb indole-3-acetic acid (IAA) homeostasis in *Arabidopsis*.^50^ Moreover, the transcript of a gene homolog to PGP13 (P-GLYCOPROTEIN 13), an ATPase involved in auxin transport, increased at time point 1 in all three citrus genotypes. Taken together, reduced expression of GA2ox8 and UDP- glucosyltransferase, and increased expression of LOX2 and PGP13 at early stage of fruit development may permit a high degree of parthenocarpy via effects on hormone metabolism in seedless mutants.

Protein metabolism plays an important role in seed development. Several proteases are represented within the GDTA at time point 1, including a citrus transcript homologous to the *Arabidopsis* cysteine protease1 (CEP1) which has been reported to be upregulated in developing seeds and involved in programmed cell death (PCD).^51^ CEP1 knock-out mutants in *Arabidopsis* exhibited disrupted tapetal PCD and decreased pollen fertility with abnormal pollen exine, indicating that normal CEP1 expression is necessary for timely degeneration of tapetal cells and development of functional pollen.^52^ Notably, the citrus cysteine protease gene exhibited higher transcript abundance at time point 1, no abundance difference was found at time point 2 and lower transcript abundance was observed at time point 3 (Table 3), suggesting that this gene is strictly regulated during early fruit development and might be important for seedless fruit development. Interestingly, another GDTA involved in protein metabolism, an aspartic protease gene, exhibited lower transcript abundance at all time points in all three seedless citrus genotypes (Table 2). Aspartic proteases are a class of simple proteolytic enzymes found in a wide variety of plants and other organisms.^53^ It has been suggested that the seed aspartic proteases may initiate the hydrolysis of seed-storage proteins in germinating seeds before the massive *de novo* synthesis of cysteine proteases.^54^ It would be worthwhile to investigate citrus aspartic protease orthologs to study if transgenic manipulation of their expression has an effect on development of seedless fruit in both seedy and poorly parthenocarpic genotypes. Moreover, a gene coding 40 s ribosomal protein S24 exhibited higher transcript abundance in all three seedless versus seedy citrus genotypes at time point 2, but no differential transcript abundance found at time 1 or 3. These genes involved in protein metabolism might contribute to high levels of parthenocarpy.

In addition, two NAC transcription factors involved in development exhibited higher transcript abundance at time point 2 in seedless mutants of all three citrus genotypes. NAC transcription factors play essential roles in senescence^55^ and ANAC072 was reported to promote chlorophyll degradation during age-and dark-induced leaf senescence.^56^ Interestingly, several transcription factors including ANAC102 were downregulated in seedless Ponkan mandarin,^24^ suggesting that different citrus genotypes might exploit diverse transcription factors at different time points during early fruit development. There were several other genes that might be important to seedless fruit development.

The expression changes observed in this study do not distinguish between changes that are associated with the development of seedless fruit, and expression changes that may induce seedlessness. However, this work does provide a list of candidates that could be evaluated further for their ability to enhance seedless fruit development when their sequence is mutated or their regulation is altered. The transcriptional differences we present may reflect a number of inter-related processes such as triggering of ovule abortion, ovule degradation and loss of hormonal signals, disruption of seed development, and physiological responses compensating for loss of ovules that allow ongoing fruit development. Mimicking some of these transcriptional changes through biotechnology may enhance seedless fruit development by affecting one or several of these processes. However, this can only be confirmed by gene function experiments.

## Conclusions

**Figure 7:**
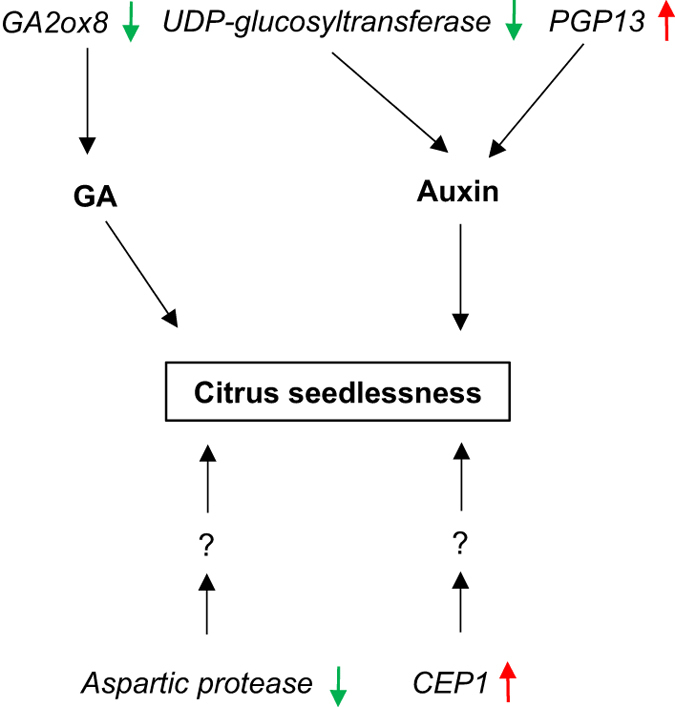
Illustration of suppression/activation of multiple pathways involved in citrus seedless fruit development. The green arrows indicate downregulation of the gene and red for upregulation of the gene besides them. The question marks indicate unknown component(s) of the signaling pathway.

By comparing transcriptome profiles of seedy Fallglo tangerine, grapefruit and Pineapple sweet oranges with their seedless mutants, we have identified a list of GDTA between seedy and near isogenic seedless citrus cultivars. The study of gene transcriptional abundance at three time points in early fruit development identified seedless versus seedy GDTA that were common in all three seedless citrus genotypes. It must be noted that a global assessment of transcriptomes will provide numerous potential relationships, with many having unclear function in the biological differences in the material studied. However, some of the GDTA revealed in this study are reasonable candidate genes where transcript level differences may be important for seedless fruit development, especially the genes shared by three citrus genotypes at time point 1 such as *GA2ox8*, *UDP-glucosyltransferase*, *PGP13*, *aspartic protease* and cysteine protease (*CEP1*). A network illustration showing the multiple pathways involved in citrus seedlessness was hypothesized (Figure 7).The seedless variants used for this study all have strong parthenocarpy with very similar yields as their seedy variants. Many genes with differential transcript levels between seedless and seedy variants may reflect physiological responses in these strongly parthenocarpic variants, which permit sustained fruit growth in the absence of seeds. It would be interesting to compare transcriptional differences between strongly parthenocarpic and weakly parthenocarpic genotypes during early fruit development. The list of candidate genes outlines future targets for characterization and functional studies, which may ultimately enhance development of new seedless citrus cultivars.

## Figures and Tables

**Table 1:** Summary of GDTA in seedless versus seedy fruits of three citrus genotypes

*Genotypes _Time point*	*Total PDTA*^a^	*PDTA with AtGID*^b^	*GDTA*^c^
Fallglo _Time 1	994	792	632
Fallglo _Time 2	593	465	406
Fallglo _Time 3	408	311	289
Grapefruit _Time 1	638	495	437
Grapefruit _Time 2	784	584	519
Grapefruit _Time 3	1077	825	701
Pineapple _Time 1	792	637	525
Pineapple _Time 2	359	261	245
Pineapple _Time 3	886	634	572

Abbreviations: GDTA, genes with differential transcript abundance; PDTA, probes with differential transcript abundance.

a Numbers of PDTA. Note that there were several probes for some genes.

b Numbers of PDTA having matches with annotated Arabidopsis Genome Initiative (AtGID) orthologs.

c Numbers of genes with differential transcript abundance (GDTA) after elimination of redundant PDTA specific for the same gene.

**Table 2:**
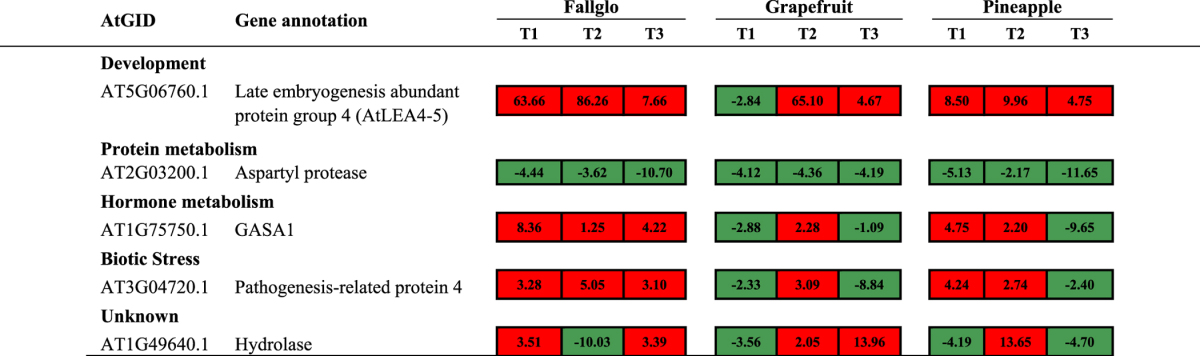
GDTA in seedless versus seedy fruits detected at all three time points across all three citrus genotypes

**Table 3:**
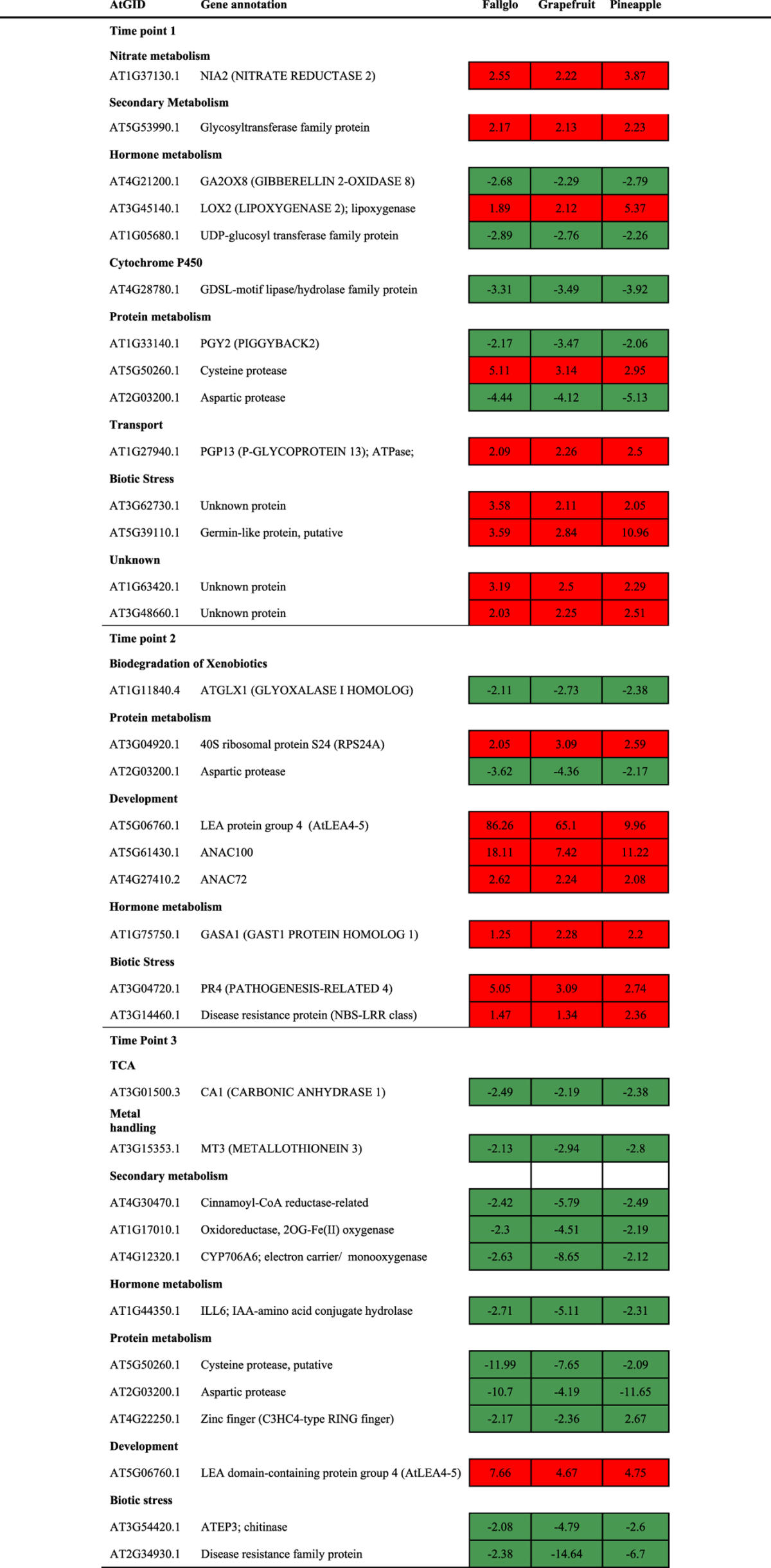
Shared GDTA by the three citrus genotypes at each time point
