# Supplementary material for: Comparative transcriptome analysis during early fruit development between three seedy citrus genotypes and their seedless mutants
Source: Hortic Res. 2017 Sep 13;4:17041–. doi: 10.1038/hortres.2017.41 (PMC5596110; doi:10.1038/hortres.2017.41)
Supplement: Supplementary Figures S1–S9 [file hortres201741-s1.pdf]

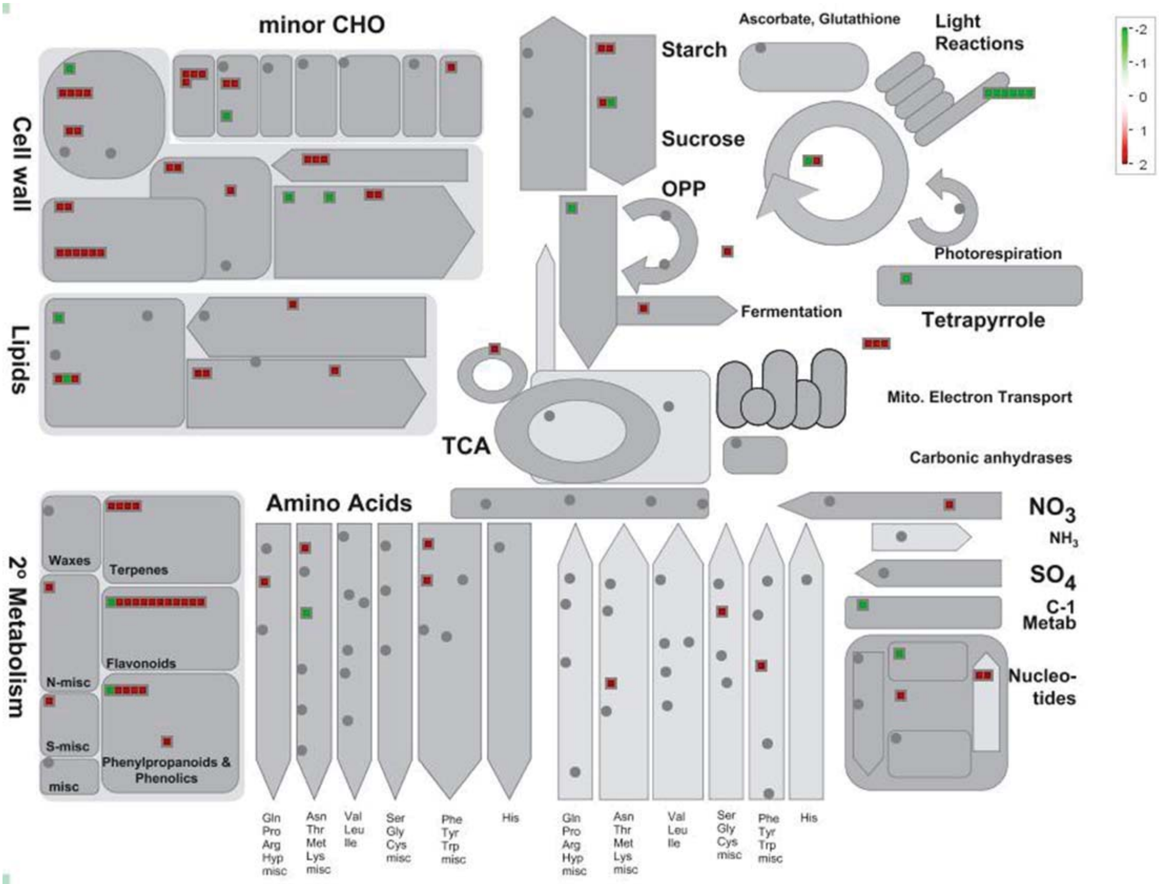

Supplementary Figure S1. MapMan metabolism overview of GDTA in seedless vs. seedy Fallglo fruits at time point 1. The abundance ratio of GDTA is displayed as illustrated in the color bar at upper right of each panel. Green is for GDTA with lower transcript abundance and red is for GDTA with higher transcript abundance.

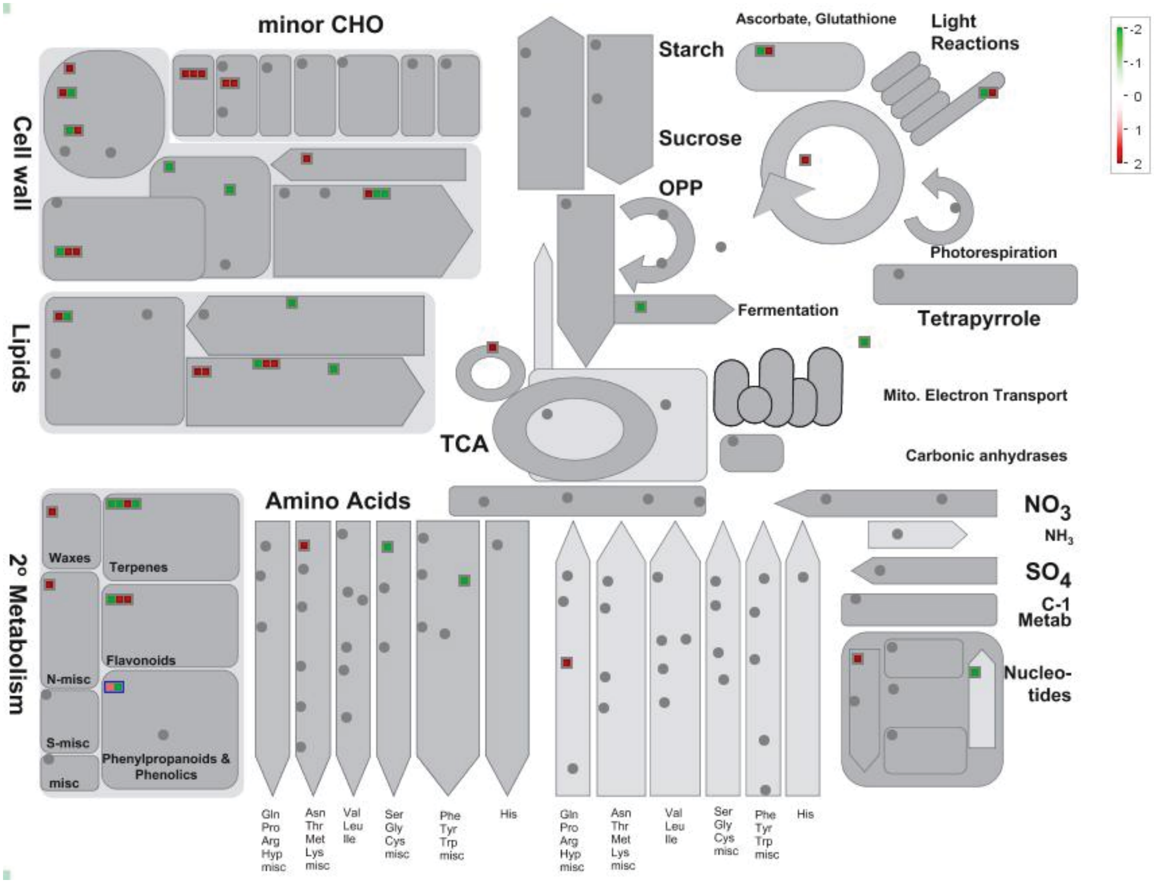

Supplementary Figure S2. MapMan metabolism overview of GDTA in seedless vs. seedy Fallglo fruits at time point 2. The abundance ratio of GDTA is displayed as illustrated in the color bar at upper right of each panel. Green is for GDTA with lower transcript abundance and red is for GDTA with higher transcript abundance.

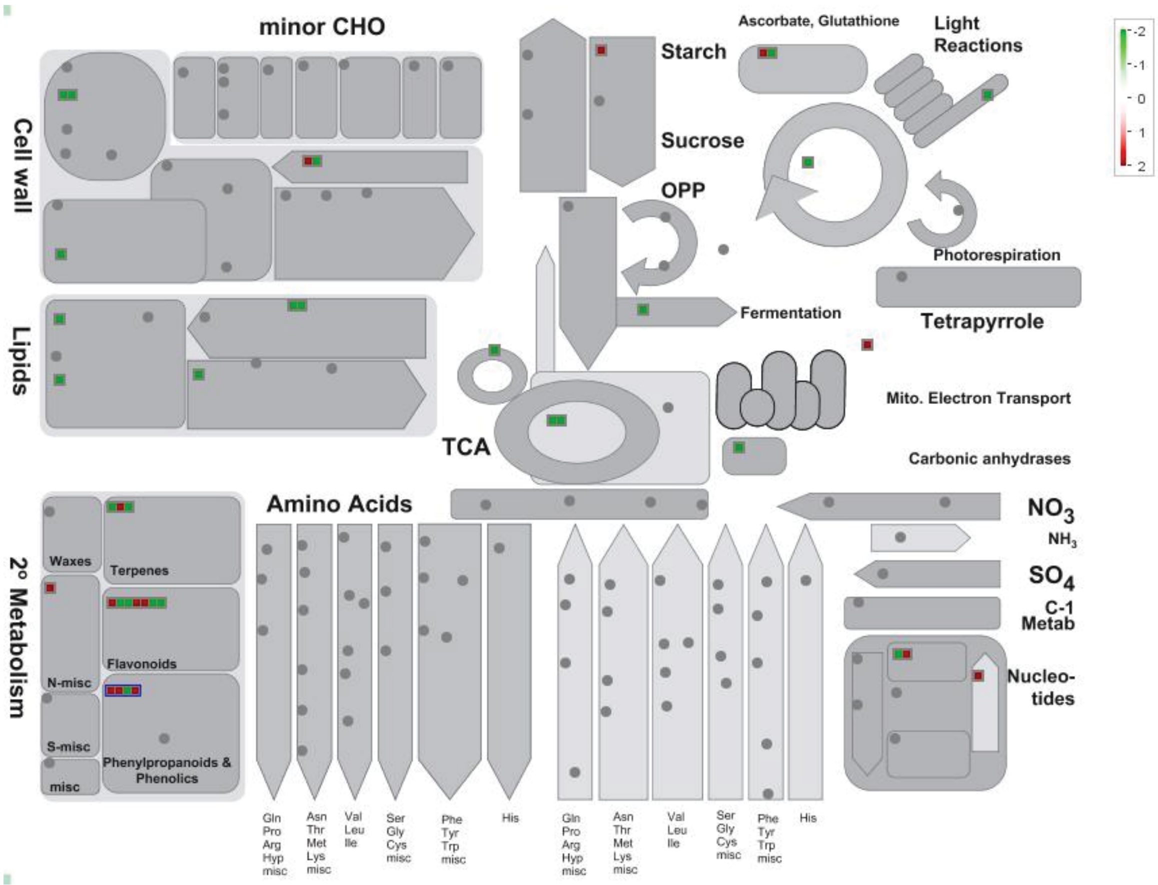

Supplementary Figure S3. MapMan metabolism overview of GDTA in seedless vs. seedy Fallglo fruits at time point 3. The abundance ratio of GDTA is displayed as illustrated in the color bar at upper right of each panel. Green is for GDTA with lower transcript abundance and red is for GDTA with higher transcript abundance.

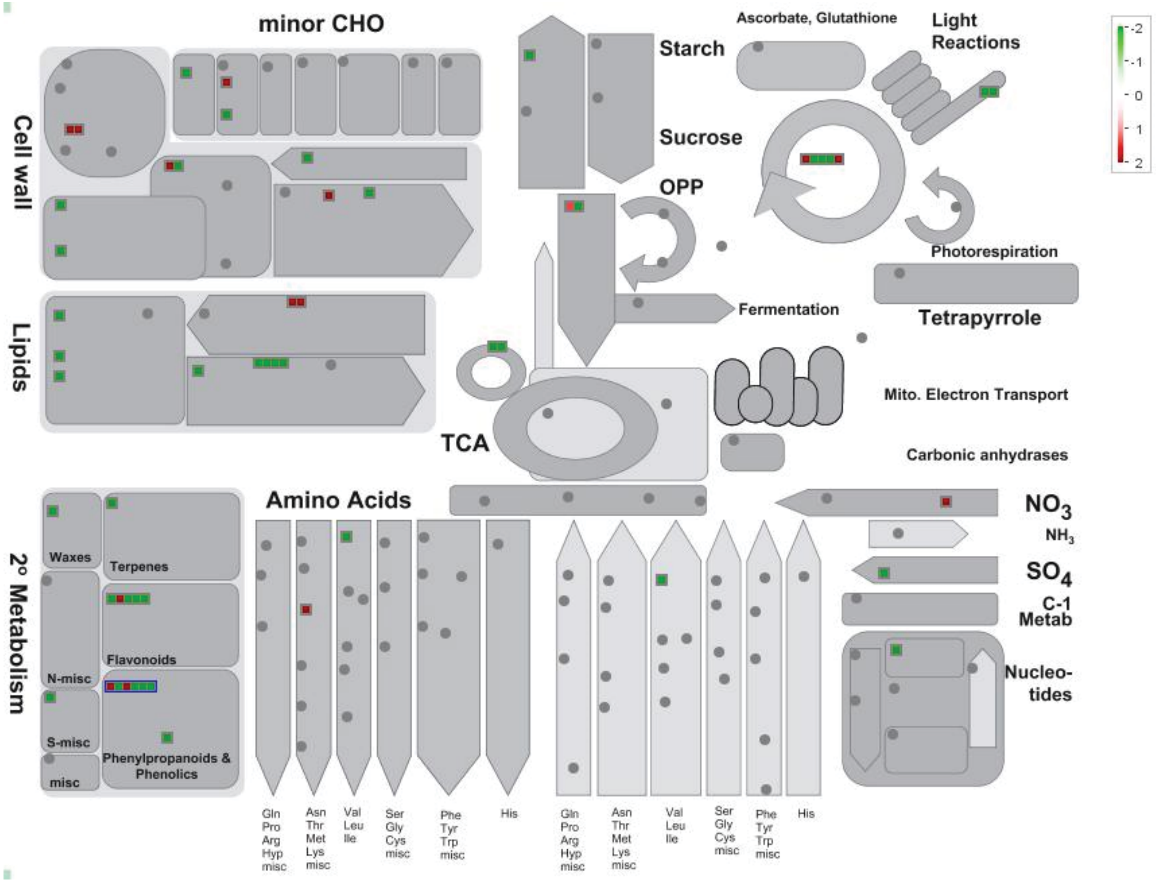

Supplementary **Figure S4. MapMan metabolism overview of GDTA in seedless vs. seedy grapefruits at time point 1.** The abundance ratio of GDTA is displayed as illustrated in the color bar at upper right of each panel. Green is for GDTA with lower transcript abundance and red is for GDTA with higher transcript abundance.

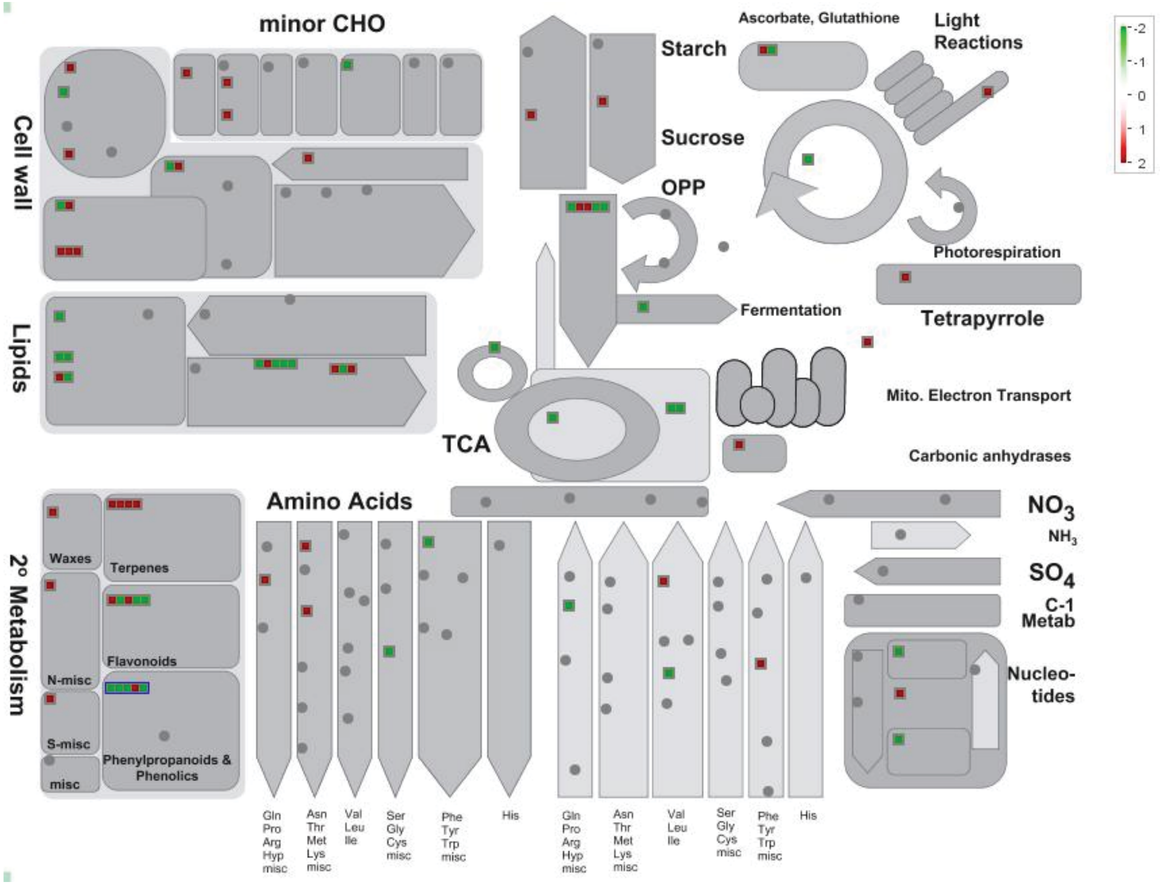

Supplementary Figure S5. MapMan metabolism overview of GDTA in seedless vs. seedy grapefruits at time point 2. The abundance ratio of GDTA is displayed as illustrated in the color bar at upper right of each panel. Green is for GDTA with lower transcript abundance and red is for GDTA with higher transcript abundance.

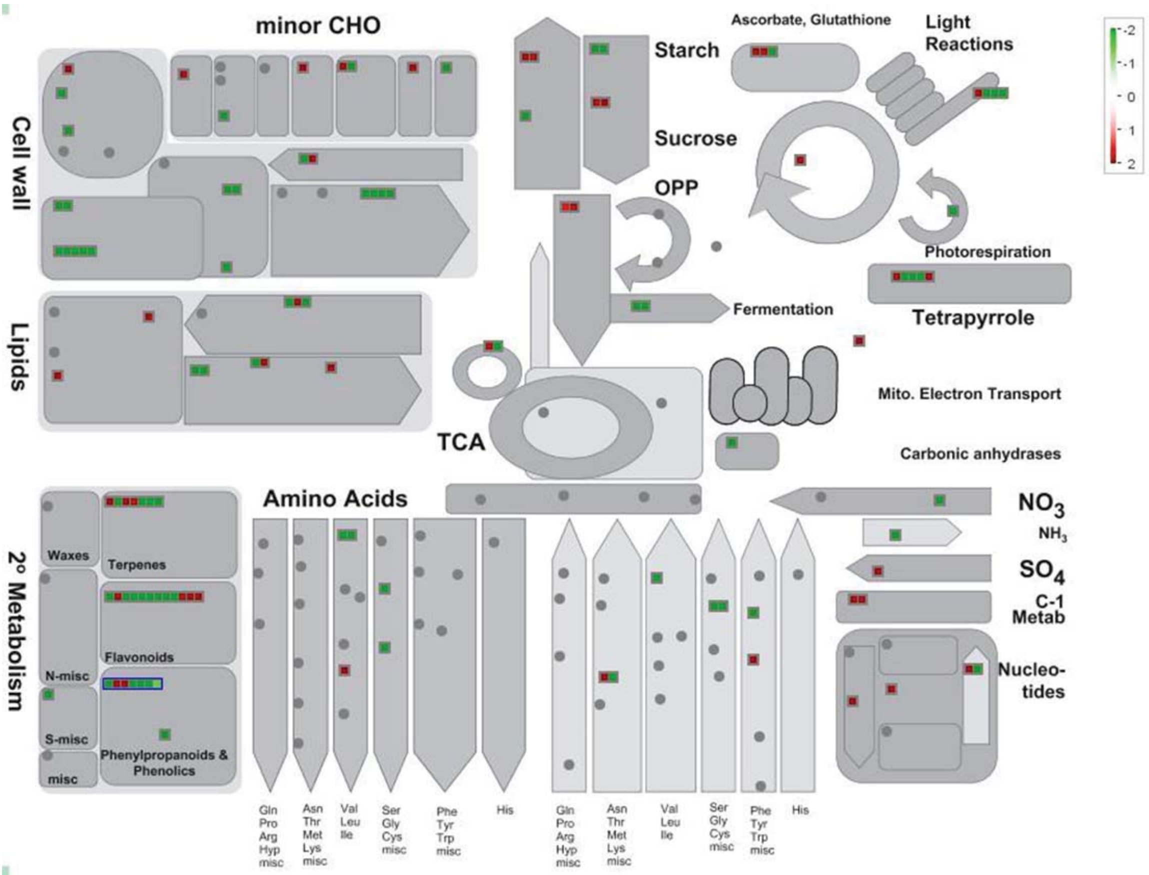

Supplementary Figure S6. MapMan metabolism overview of GDTA in seedless vs. seedy grapefruits at time point 3. The abundance ratio of GDTA is displayed as illustrated in the color bar at upper right of each panel. Green is for GDTA with lower transcript abundance and red is for GDTA with higher transcript abundance.

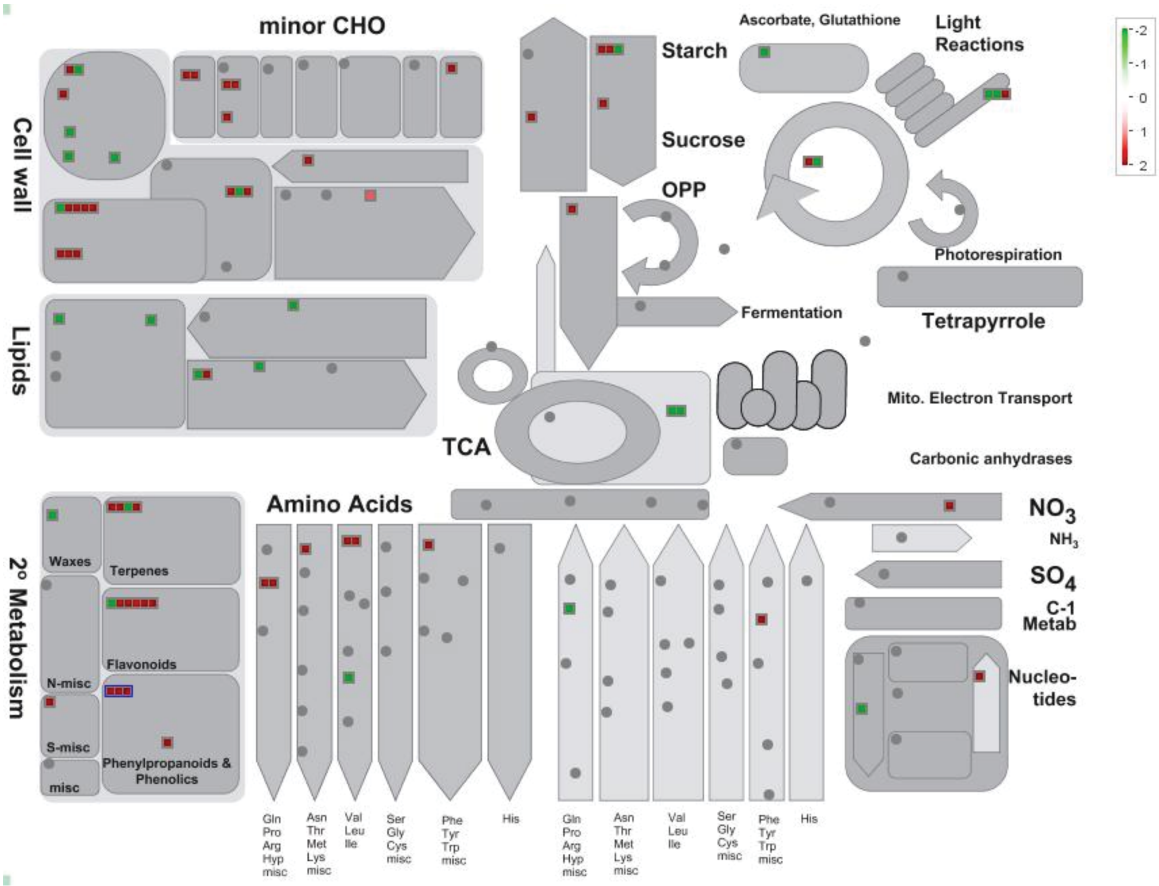

Supplementary Figure S7. MapMan metabolism overview of GDTA in seedless vs. seedy Pineapple sweet orange fruits at time point 1. The abundance ratio of GDTA is displayed as illustrated in the color bar at upper right of each panel. Green is for GDTA with lower transcript abundance and red is for GDTA with higher transcript abundance.

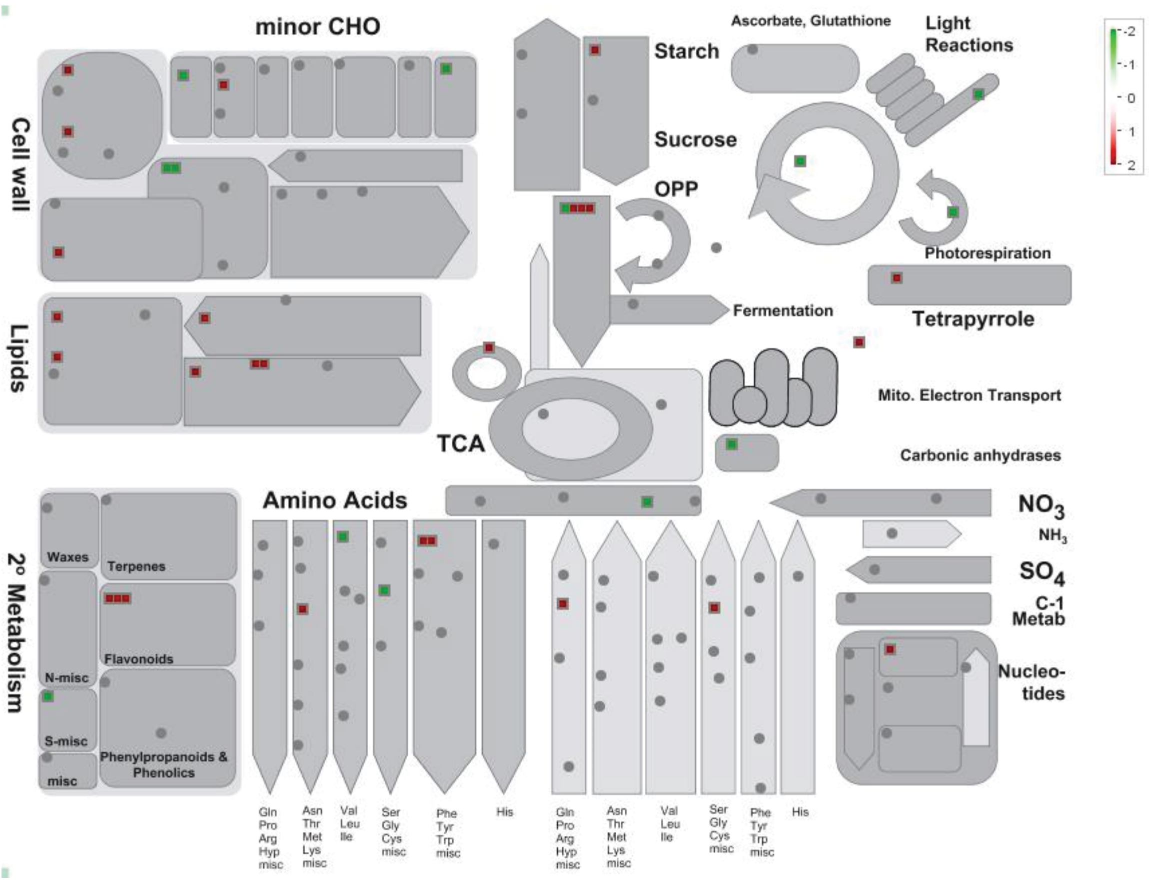

Supplementary Figure S8. MapMan metabolism overview of GDTA in seedless vs. seedy Pineapple sweet orange fruits at time point 2. The abundance ratio of GDTA is displayed as illustrated in the color bar at upper right of each panel. Green is for GDTA with lower transcript abundance and red is for GDTA with higher transcript abundance.

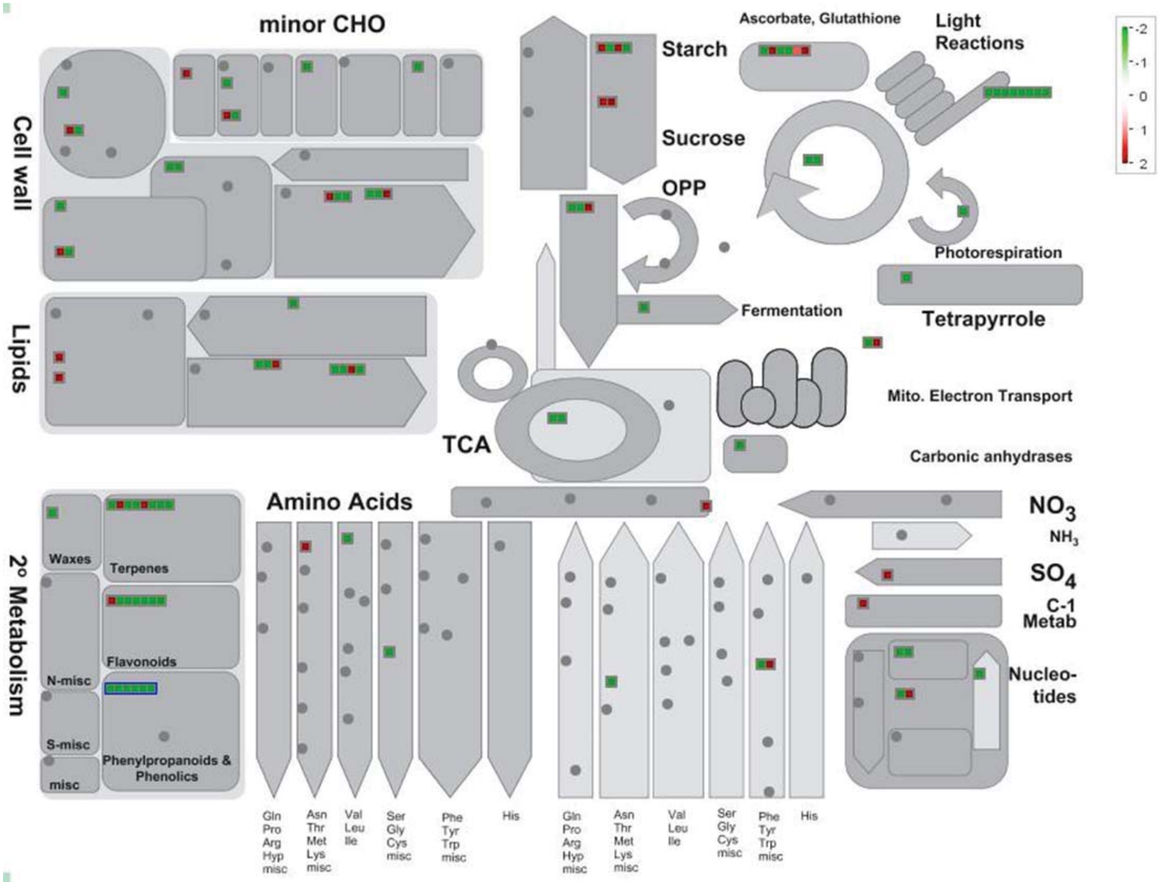

Supplementary Figure S9. MapMan metabolism overview of GDTA in seedless vs. seedy Pineapple sweet orange fruits at time 3. The abundance ratio of GDTA is displayed as illustrated in the color bar at upper right of each panel. Green is for GDTA with lower transcript abundance and red is for GDTA with higher transcript abundance.
