# Supplementary material for: Comparative transcriptome analysis during early fruit development between three seedy citrus genotypes and their seedless mutants
Source: Hortic Res. 2017 Sep 13;4:17041–. doi: 10.1038/hortres.2017.41 (PMC5596110; doi:10.1038/hortres.2017.41)
Supplement: Supplementary Figures S10–S12 [file hortres201741-s2.pdf]

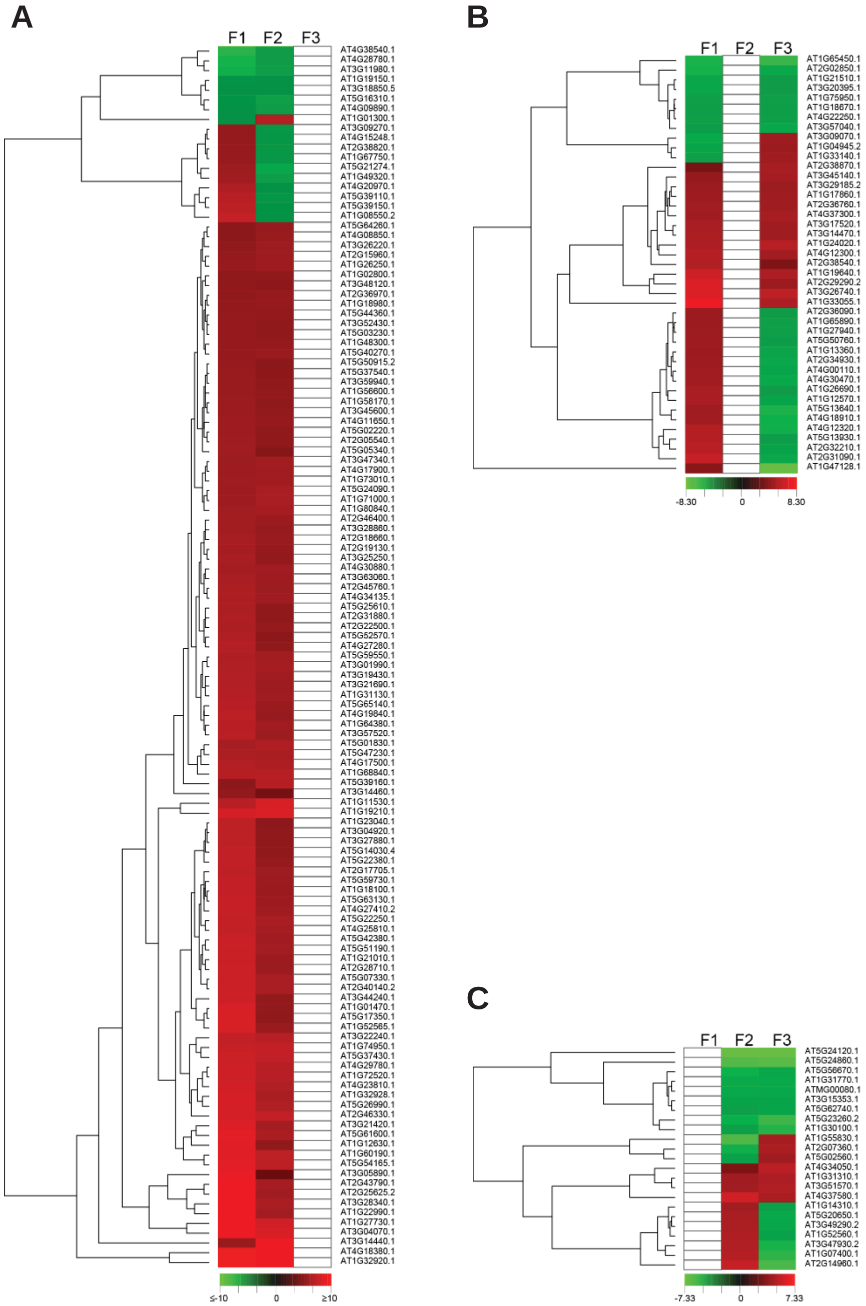

Supplementary Figure S10. Expression heat maps of GDTA detected at any two, but not all three time points in seedless vs. seedy Fallglo (F) fruits. A hierarchical clustering analysis of GDTA transcription profiles for Fallglo using a Pearson correlation was computed by using PermutMatrix v1.9.3. The abundance ratio of GDTA is displayed as illustrated in the color bar at the bottom of each panel. Green is for GDTA with lower transcript abundance and red is for GDTA with higher transcript abundance. White square means the corresponding gene didn't exhibit differential transcript abundance at indicated time point.

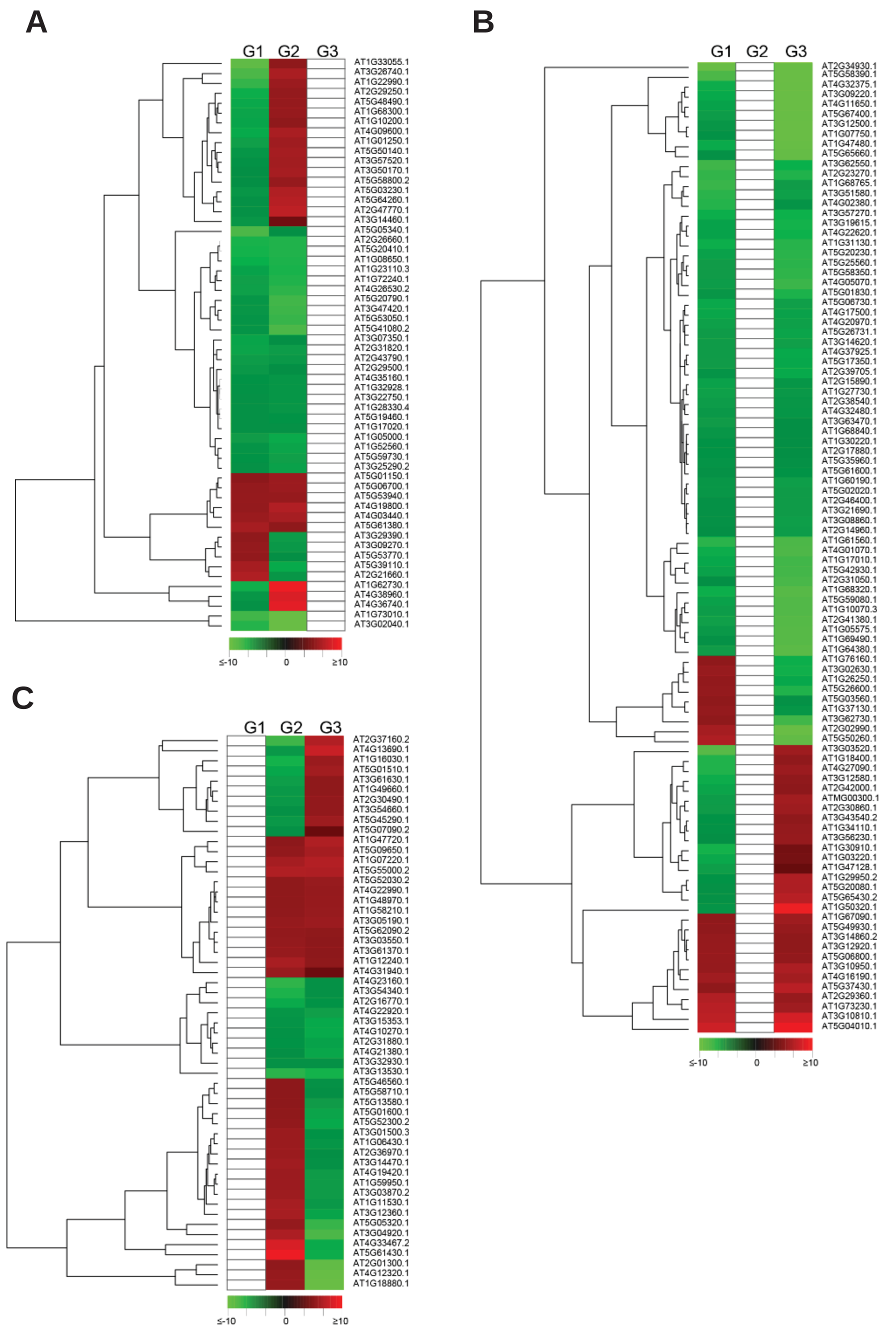

Supplementary Figure S11. Expression heat maps of GDTA detected at any two, but not all three points in seedless vs. seedy grapefruit (G). A hierarchical clustering analysis of GDTA transcription profiles for Fallglo using a Pearson correlation was computed by using PermutMatrix v1.9.3. The abundance ratio of GDTA is displayed as illustrated in the color bar at the bottom of each panel. Green is for GDTA with lower transcript abundance and red is for GDTA with higher transcript abundance. White square means the corresponding gene didn’t exhibit differential transcript abundance at indicated time point.

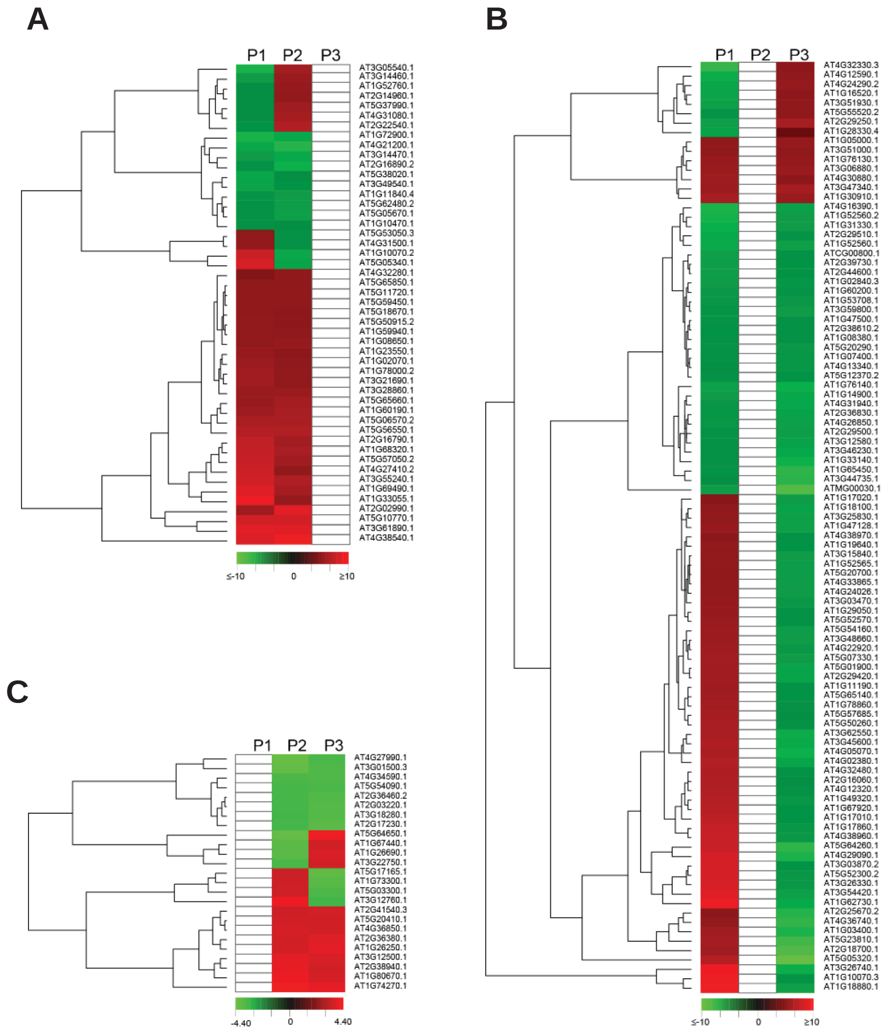

Supplementary Figure S12. Expression heat maps of GDTA detected at any two, but not all three points in seedless vs. seedy Pineapple (P) fruits. A hierarchical clustering analysis of GDTA transcription profiles for Fallglo using a Pearson correlation was computed by using PermutMatrix v1.9.3. The abundance ratio of GDTA is displayed as illustrated in the color bar at the bottom of each panel. Green is for GDTA with lower transcript abundance and red is for GDTA with higher transcript abundance. White square means the corresponding gene didn't exhibit differential transcript abundance at indicated time point.
