# Supplementary material for: Comparative transcriptome analysis during early fruit development between three seedy citrus genotypes and their seedless mutants
Source: Hortic Res. 2017 Sep 13;4:17041–. doi: 10.1038/hortres.2017.41 (PMC5596110; doi:10.1038/hortres.2017.41)
Supplement: Supplementary Tables [file hortres201741-s3.pdf]

Supplementary Table S1. Probes with differential transcript abundance (PDTA) in seedless vs. seedy Fallglo fruits at time point 1.

ProbeSet ID	Fallglo_Seedless	Fallglo_Seedy	Ratio	AtGID	E-Score	Arabidopsis.annotation
Cit.10894.1.S1_s_at	2168.16	34.0565	63.664	AT5G06760.1	1.00E-48	late embryogenesis abundant group 1 domain-containing protein /
Cit.2409.1.S1_s_at	3043.246	82.9109	36.705	AT3G09220.1	0	LAC7 (laccase 7); laccase
Cit.26593.1.S1_at	1097.362	34.6156	31.701		NA	
Cit.17090.1.S1_s_at	739.2175	34.7962	21.244	AT1G69490.1	4.00E-89	NAP (NAC-like, activated by AP3/PI); transcription factor
Cit.3665.1.S1_at	704.3483	41.1518	17.116	AT1G75750.1	2.00E-30	GASA1 (GAST1 PROTEIN HOMOLOG 1)
Cit.5970.1.S1_at	1143.177	77.5223	14.746	AT5G61430.1	6.00E-92	ANAC100 (ARABIDOPSIS NAC DOMAIN CONTAINING PROTEIN 100);
Cit.21810.1.S1_x_at	344.6234	24.1168	14.29		NA	
Cit.17294.1.S1_at	1320.069	97.1538	13.587		NA	
Cit.17243.1.S1_at	804.0527	66.3363	12.121	AT1G32920.1	0.0000003	unknown protein
Cit.12433.1.S1_at	307.6883	25.6371	12.002		NA	
Cit.30163.1.S1_at	371.3899	31.6382	11.739	AT1G72240.1	4.00E-12	unknown protein
Cit.1496.1.S1_s_at	469.6369	40.5883	11.571	AT3G04070.1	1.00E-98	anac047 (Arabidopsis NAC domain containing protein 47);
Cit.3195.1.S1_at	283.3329	24.5591	11.537	AT1G33055.1	7.00E-13	unknown protein
Cit.29547.1.S1_at	830.5537	74.7003	11.118	AT4G18380.1	6.00E-46	F-box family protein
Cit.15649.1.S1_at	617.0496	56.8242	10.859	AT4G25490.1	1.00E-53	CBF1 (C-REPEAT/DRE BINDING FACTOR 1); DNA binding / transcription
Cit.3665.1.S1_s_at	325.4422	31.652	10.282	AT1G75750.1	2.00E-30	GASA1 (GAST1 PROTEIN HOMOLOG 1)
Cit.9803.1.S1_s_at	237.5749	23.3595	10.17		NA	
Cit.13586.1.S1_at	603.3818	59.582	10.127	AT3G49940.1	2.00E-52	LBD38 (LOB DOMAIN-CONTAINING PROTEIN 38)
Cit.3236.1.S1_at	374.0923	41.2205	9.0754	AT5G39670.1	1.00E-40	calcium-binding EF hand family protein
Cit.17178.1.S1_x_at	308.138	33.9996	9.063		NA	
Cit.6076.1.S1_s_at	825.4907	92.1188	8.9612	AT1G22990.1	2.00E-51	heavy-metal-associated domain-containing protein / copper
Cit.35345.1.S1_s_at	787.8082	94.8706	8.304	AT3G28340.1	1.00E-139	GATL10 (Galacturonosyltransferase-like 10); polygalacturonate 4-alpha-galacturonosyltransferase/ transferase, transferring hexo
Cit.10594.1.S1_at	837.6155	102.976	8.1341	AT3G54420.1	3.00E-66	ATEP3; chitinase
Cit.31360.1.S1_at	399.4646	49.3854	8.0887		NA	
Cit.7334.1.S1_at	670.9878	84.7142	7.9206	AT5G17350.1	7.00E-37	unknown protein
Cit.22602.1.S1_at	450.592	59.0873	7.6259	AT1G61800.1	3.00E-32	GPT2; antiporter/ glucose-6-phosphate transmembrane transporter
Cit.5438.1.S1_at	193.703	25.6533	7.5508	AT2G25625.2	8.00E-20	unknown protein
Cit.37764.1.S1_s_at	553.8682	74.5914	7.4254	AT1G27730.1	7.00E-57	STZ (salt tolerance zinc finger); nucleic acid binding / transcription
Cit.34286.1.S1_at	380.6489	51.3303	7.4157		NA	

Cit.30629.1.S1_at	251.9323	33.9951	7.4108	AT2G43790.1	1.00E-143	ATMPK6 (ARABIDOPSIS THALIANA MAP KINASE 6); MAP kinase/
Cit.6765.1.S1_at	145.2855	20.0026	7.2633	AT3G26330.1	9.00E-69	CYP71B37; electron carrier/ heme binding / iron ion binding /
Cit.12779.1.S1_at	620.6314	86.5919	7.1673	AT4G23810.1	3.00E-57	WRKY53; DNA binding / protein binding / transcription activator/
Cit.15242.1.S1_at	220.5895	31.7617	6.9452	AT3G54420.1	2.00E-74	ATEP3; chitinase
Cit.9301.1.S1_s_at	521.3306	75.7635	6.881	AT5G06570.2	3.00E-47	hydrolase
Cit.31296.1.S1_at	926.0121	137.846	6.7177		NA	
Cit.23585.1.S1_at	316.911	47.7104	6.6424	AT1G01470.1	5.00E-31	LEA14 (LATE EMBRYOGENESIS ABUNDANT 14)
Cit.8718.1.S1_s_at	363.7329	54.8774	6.6281	5G54160.1	1.00E-105	ATOMT1 (O-METHYLTRANSFERASE 1); caffeate O-methyltransferase/ myricetin 3'-O-methyltransferase/ quercetin 3-O-methyltransferase
Cit.25075.1.S1_s_at	271.827	41.0276	6.6255	AT1G18880.1	4.00E-72	proton-dependent oligopeptide transport (POT) family protein
Cit.25942.1.S1_s_at	306.2342	46.4249	6.5963	AT5G06320.1	2.00E-66	NHL3
Cit.30437.1.S1_s_at	429.4482	65.1407	6.5926	AT3G05890.1	7.00E-21	RCI2B (RARE-COLD-INDUCIBLE 2B)
Cit.15458.1.S1_at	348.8965	53.5423	6.5163	AT1G18400.1	1.00E-45	BEE1 (BR Enhanced Expression 1); transcription factor
Cit.39642.1.S1_at	190.1754	29.3838	6.4721	AT5G26170.1	4.00E-32	WRKY50; transcription factor
Cit.2690.1.S1_s_at	246.7178	38.2403	6.4518	AT4G20820.1	1.00E-151	FAD-binding domain-containing protein
Cit.40496.1.S1_s_at	127.1919	20.1296	6.3187	AT4G25810.1	1.00E-129	XTR6 (XYLOGLUCAN ENDOTRANSGLYCOSYLASE 6); hydrolase, acting
Cit.1270.1.S1_s_at	2040.336	326.046	6.2578	AT5G61600.1	4.00E-45	ethylene-responsive element-binding family protein
Cit.13036.1.S1_at	586.0042	95.9168	6.1095	AT5G55620.1	2.00E-18	unknown protein
Cit.4752.1.S1_s_at	942.0451	155.583	6.0549	AT5G54165.1	3E-08	unknown protein
Cit.2927.1.S1_s_at	594.3709	98.2585	6.0491	AT5G23810.1	2.00E-96	AAP7; amino acid transmembrane transporter
Cit.12302.1.S1_at	250.9017	42.1684	5.95	AT1G42990.1	6.00E-30	ATBZIP60 (BASIC REGION/LEUCINE ZIPPER MOTIF 60); DNA binding /
Cit.29605.1.S1_at	365.3771	61.4831	5.9427		NA	
Cit.4030.1.S1_at	844.6681	143.988	5.8662	AT1G60190.1	4.00E-82	armadillo/beta-catenin repeat family protein / U-box domain-
Cit.18673.1.S1_at	531.4648	91.0253	5.8387	AT1G12630.1	2.00E-34	DNA binding / transcription activator/ transcription factor
Cit.4078.1.S1_at	238.523	41.1689	5.7938	AT5G50260.1	1.00E-158	cysteine proteinase, putative
Cit.32853.1.S1_at	138.4181	23.8926	5.7934	AT3G15760.1	3.00E-22	unknown protein
Cit.30695.1.S1_s_at	2045.958	362.334	5.6466	AT3G21420.1	1.00E-151	oxidoreductase, 2OG-Fe(II) oxygenase family protein
Cit.18008.1.S1_s_at	279.3604	49.598	5.6325		NA	
Cit.22427.1.S1_s_at	236.7086	42.4913	5.5708	AT4G15920.1	1.00E-64	INVOLVED IN: biological_process unknown; LOCATED IN:
Cit.17450.1.S1_s_at	2559.217	460.227	5.5608	AT2G46330.1	5.00E-13	AGP16 (ARABINOGALACTAN PROTEIN 16)
Cit.8663.1.S1_x_at	318.8037	57.5381	5.5407	AT1G68725.1	0.000005	AGP19 (ARABINOGALACTAN-PROTEIN 19)
Cit.21654.1.S1_s_at	416.3081	75.5713	5.5088	AT3G54420.1	3.00E-66	ATEP3; chitinase
Cit.1497.1.S1_s_at	480.208	87.3186	5.4995	AT3G04070.1	1.00E-98	anac047 (Arabidopsis NAC domain containing protein 47);
Cit.16392.1.S1_at	254.6889	46.7848	5.4438		NA	

Cit.25028.1.S1_at	255.5663	47.3583	5.3964	AT3G02840.1	2.00E-40	immediate-early fungal elicitor family protein
Cit.18677.1.S1_s_at	297.5533	55.5331	5.3581	AT3G54420.1	6.00E-76	ATEP3; chitinase
Cit.31360.1.S1_s_at	489.2974	92.2166	5.306		NA	
Cit.13963.1.S1_s_at	916.9544	174.879	5.2434	AT1G24130.1	1.00E-135	transducin family protein / WD-40 repeat family protein
Cit.3042.1.S1_s_at	1357.984	261.673	5.1896	AT1G52565.1	1.00E-31	unknown protein
Cit.18642.1.S1_s_at	437.0599	84.3601	5.1809	AT1G20030.2	3.00E-79	pathogenesis-related thaumatin family protein
Cit.2008.1.S1_s_at	103.8988	20.0627	5.1787		NA	
Cit.17142.1.S1_s_at	2577.451	503.442	5.1197	AT5G44210.1	8.00E-43	ERF9 (ERF DOMAIN PROTEIN 9); DNA binding / transcription factor/
Cit.37995.1.S1_at	121.179	23.7141	5.11	AT4G31290.1	1.00E-14	ChaC-like family protein
Cit.26086.1.S1_at	206.6905	40.474	5.1068	AT5G26990.1	0.00008	drought-responsive family protein
Cit.10258.1.S1_s_at	485.9557	95.6596	5.0801	AT1G32928.1	5E-09	unknown protein
Cit.11676.1.S1_at	444.7262	88.7414	5.0115		NA	
Cit.3778.1.S1_at	1762.697	352.108	5.0061	AT1G19210.1	1.00E-45	AP2 domain-containing transcription factor, putative
Cit.5377.1.S1_at	566.0367	113.716	4.9776	AT4G37370.1	1.00E-156	CYP81D8; electron carrier/ heme binding / iron ion binding /
Cit.13244.1.S1_at	438.2251	88.1308	4.9724	AT5G42380.1	1.00E-36	CML37 (CALMODULIN LIKE 37); calcium ion binding
Cit.1557.1.S1_s_at	2959.811	595.913	4.9669		NA	
Cit.8725.1.S1_at	425.8512	86.1011	4.9459	AT5G54160.1	1.00E-105	ATOMT1 (O-METHYLTRANSFERASE 1); caffeate O-methyltransferase/
Cit.28237.1.S1_at	302.4176	61.2385	4.9384		NA	
Cit.1820.1.S1_at	100.7493	20.7606	4.8529	AT2G28710.1	9.00E-34	zinc finger (C2H2 type) family protein
Cit.36207.1.S1_at	577.9795	119.803	4.8244		NA	
Cit.30494.1.S1_s_at	295.8359	61.9467	4.7757	AT2G40140.2	1.00E-147	CZF1; transcription factor
Cit.1039.1.S1_at	467.8357	98.4251	4.7532	AT3G03870.2	1.00E-37	unknown protein
Cit.12810.1.S1_at	475.1788	100.246	4.7401	AT1G19670.1	5.00E-65	ATCLH1 (ARABIDOPSIS THALIANA CORONATINE-INDUCED PROTEIN 1);
Cit.4810.1.S1_at	343.8706	72.8479	4.7204	AT3G23240.1	2.00E-64	ERF1 (ETHYLENE RESPONSE FACTOR 1); DNA binding / transcription
Cit.25990.1.S1_x_at	2421.961	517.42	4.6808	AT1G49640.1	0.000005	hydrolase
Cit.3523.1.S1_at	4556.2	979.166	4.6531	AT4G29780.1	0	unknown protein
Cit.18017.1.S1_s_at	406.8595	88.0384	4.6214	AT1G72520.1	0	lipoxygenase, putative
Cit.10152.1.S1_s_at	608.0159	132.066	4.6039	AT4G27410.2	1.00E-110	RD26 (RESPONSIVE TO DESICCATION 26); transcription activator/
Cit.21825.1.S1_at	198.3365	43.2949	4.5811	AT1G64380.1	8.00E-13	AP2 domain-containing transcription factor, putative
Cit.17309.1.S1_at	303.4498	66.3847	4.5711	AT3G03341.1	3.00E-28	unknown protein
Cit.10686.1.S1_at	708.1429	155.563	4.5521	AT1G60420.1	8.00E-86	DC1 domain-containing protein
Cit.38508.1.S1_at	337.7557	74.2807	4.547	AT3G44240.1	5.00E-47	CCR4-NOT transcription complex protein, putative
Cit.16807.1.S1_at	616.2209	135.565	4.5456	AT3G63010.1	9.00E-66	GID1B (GA INSENSITIVE DWARF1B); hydrolase
Cit.11563.1.S1_at	625.4323	137.945	4.5339	AT5G37430.1	0.00003	unknown protein
Cit.11666.1.S1_s_at	2711.374	598.354	4.5314	AT5G59550.1	9.00E-63	zinc finger (C3HC4-type RING finger) family protein

Cit.10278.1.S1_x_at	945.6307	209.328	4.5175	AT3G57520.1	5.00E-81	AtSIP2 (Arabidopsis thaliana seed imbibition 2); hydrolase,
Cit.29575.1.S1_s_at	697.7696	154.542	4.5151	AT4G29780.1	0	unknown protein
Cit.17450.1.S1_at	412.3239	91.4474	4.5089	AT2G46330.1	9E-09	AGP16 (ARABINOGALACTAN PROTEIN 16)
Cit.13663.1.S1_at	126.2437	28.046	4.5013	AT2G29290.2	8.00E-90	tropinone reductase, putative / tropine dehydrogenase, putative
Cit.31022.1.S1_at	226.907	50.4534	4.4974	AT2G32030.1	7.00E-51	GCN5-related N-acetyltransferase (GNAT) family protein
Cit.10673.1.S1_at	111.6695	24.8735	4.4895	AT3G26740.1	4.00E-29	CCL (CCR-LIKE)
Cit.26141.1.S1_s_at	1300.877	290.015	4.4855		NA	
Cit.18008.1.S1_at	184.6456	41.2489	4.4764		NA	
Cit.11086.1.S1_at	665.1804	148.775	4.4711	AT1G02070.1	2.00E-13	unknown protein
Cit.18482.1.S1_s_at	627.9087	140.596	4.4661	AT3G22240.1	7.00E-12	unknown protein
Cit.12814.1.S1_s_at	414.4556	92.9526	4.4588	AT5G07330.1	2.00E-36	unknown protein
Cit.29626.1.S1_s_at	113.292	25.5384	4.4361	AT5G50260.1	1.00E-158	cysteine proteinase, putative
Cit.4151.1.S1_s_at	119.8158	27.1364	4.4153	AT3G56710.1	5E-08	SIB1 (SIGMA FACTOR BINDING PROTEIN 1); binding / protein binding
Cit.29796.1.S1_at	524.9894	119.248	4.4025	AT1G21010.1	0.0000003	unknown protein
Cit.2675.1.S1_s_at	3853.43	881.476	4.3716	AT5G51190.1	2.00E-48	AP2 domain-containing transcription factor, putative
Cit.157.1.S1_x_at	704.8295	162.788	4.3297		NA	
Cit.5502.1.S1_at	282.6033	65.4212	4.3197	AT3G07600.1	8.00E-16	heavy-metal-associated domain-containing protein
Cit.14081.1.S1_at	957.0204	221.838	4.3141	AT2G31880.1	1.00E-118	leucine-rich repeat transmembrane protein kinase, putative
Cit.18211.1.S1_at	92.22247	21.4427	4.3009		NA	
Cit.10661.1.S1_at	1647.589	385.737	4.2713	AT1G12240.1	0	ATBETAFRUCT4; beta-fructofuranosidase/ hydrolase, hydrolyzing O-
Cit.9048.1.S1_s_at	3968.719	934.235	4.2481	AT2G40140.2	1.00E-110	CZF1; transcription factor
Cit.35355.1.S1_s_at	310.0194	73.7938	4.2012	AT1G18100.1	8.00E-55	E12A11; phosphatidylethanolamine binding
Cit.17228.1.S1_at	92.5834	22.0616	4.1966	AT4G34135.1	1.00E-131	UGT73B2 (UDP-GLUCOSYLTRANSFERASE 73B2); UDP-
Cit.26572.1.S1_s_at	103.2863	24.6448	4.191	AT5G42930.1	2.00E-44	triacylglycerol lipase
Cit.22998.1.S1_s_at	465.2986	111.173	4.1853	AT2G26070.1	2.00E-95	RTE1 (REVERSION-TO-ETHYLENE SENSITIVITY1)
Cit.1819.1.S1_s_at	1336.188	319.448	4.1828	AT2G28710.1	1.00E-34	zinc finger (C2H2 type) family protein
Cit.29507.1.S1_s_at	1887.106	451.778	4.1771		NA	
Cit.15404.1.S1_at	138.4764	33.2865	4.1601	AT4G33720.1	2.00E-58	pathogenesis-related protein, putative
Cit.30448.1.S1_s_at	120.6826	29.0129	4.1596	AT3G01990.1	9.00E-66	ACR6; amino acid binding
Cit.5512.1.S1_at	84.28468	20.2832	4.1554	AT1G12030.1	7.00E-56	unknown protein
Cit.9650.1.S1_at	567.8675	136.806	4.1509	AT1G74950.1	0.00004	TIFY10B
Cit.13924.1.S1_at	87.50175	21.1854	4.1303	AT1G08550.2	8.00E-92	NPQ1 (NON-PHOTOCHEMICAL QUENCHING 1); violaxanthin de-
Cit.2915.1.S1_s_at	1438.913	348.561	4.1282	AT5G63130.1	4.00E-43	octicosapeptide/Phox/Bem1p (PB1) domain-containing protein
Cit.8648.1.S1_at	177.4308	43.0067	4.1257		NA	
Cit.21825.1.S1_s_at	410.9952	100.908	4.073	AT1G64380.1	8.00E-13	AP2 domain-containing transcription factor, putative

Cit.18045.1.S1_s_at	608.619	149.795	4.063	AT1G01250.1	3.00E-26	AP2 domain-containing transcription factor, putative
Cit.10155.1.S1_s_at	4077.635	1004.46	4.0595	AT5G22250.1	1.00E-117	CCR4-NOT transcription complex protein, putative
Cit.10661.1.S1_s_at	1701.079	419.898	4.0512	AT1G12240.1	0	ATBETAFRUCT4; beta-fructofuranosidase/ hydrolase, hydrolyzing O-
Cit.17006.1.S1_s_at	1071.758	266.258	4.0253	AT3G03870.2	1.00E-37	unknown protein
Cit.19380.1.S1_at	183.3509	45.6451	4.0169		NA	
Cit.18572.1.S1_at	564.2197	140.8	4.0072	AT3G27880.1	2.00E-12	unknown protein
Cit.18482.1.S1_at	205.1721	51.264	4.0023	AT3G22240.1	7.00E-12	unknown protein
Cit.23912.1.S1_s_at	92.14581	23.0361	4.0001	AT5G59730.1	1.00E-54	ATEXO70H7 (EXOCYST SUBUNIT EXO70 FAMILY PROTEIN H7); protein
Cit.31377.1.S1_at	497.9517	124.817	3.9895	AT2G17040.1	2.00E-82	anac036 (Arabidopsis NAC domain containing protein 36);
Cit.18156.1.S1_at	95.72404	24.1029	3.9715	AT3G02840.1	3.00E-60	immediate-early fungal elicitor family protein
Cit.12539.1.S1_at	462.0897	116.866	3.954	AT1G18100.1	1.00E-54	E12A11; phosphatidylethanolamine binding
Cit.23299.1.S1_x_at	89.73354	22.7033	3.9524	AT5G14030.4	5.00E-33	translocon-associated protein beta (TRAPB) family protein
Cit.14905.1.S1_s_at	293.14	74.2746	3.9467	AT5G65140.1	1.00E-136	trehalose-6-phosphate phosphatase, putative
Cit.18912.1.S1_x_at	365.7907	92.7679	3.9431	AT5G39110.1	6E-09	germin-like protein, putative
Cit.37829.1.S1_s_at	260.9027	66.237	3.9389	AT5G22380.1	5.00E-67	anac090 (Arabidopsis NAC domain containing protein 90);
Cit.2959.1.S1_at	1492.916	379.499	3.9339	AT5G13000.2	0	ATGSL12 (glucan synthase-like 12); 1,3-beta-glucan synthase/
Cit.25795.1.S1_s_at	2593.129	663.409	3.9088	AT3G57520.1	0	AtSIP2 (Arabidopsis thaliana seed imbibition 2); hydrolase,
Cit.4660.1.S1_at	273.5802	70.4036	3.8859	AT1G61340.1	8.00E-37	F-box family protein
Cit.25021.1.S1_at	79.54259	20.4964	3.8808	AT1G63420.1	1.00E-29	INVOLVED IN: biological_process unknown; EXPRESSED IN: 22 plant
Cit.25808.1.S1_at	136.3046	35.1315	3.8798		NA	
Cit.6974.1.S1_at	152.1063	39.2099	3.8793		NA	
Cit.10381.1.S1_s_at	1491.309	386.409	3.8594	AT2G17705.1	2.00E-49	unknown protein
Cit.881.1.S1_s_at	199.2782	51.7711	3.8492	AT3G55240.1	4.00E-36	Overexpression leads to PEL (Pseudo-Etiolation in Light) phenotype.
Cit.18933.1.S1_s_at	186.1987	48.5301	3.8368	AT2G21180.1	6.00E-30	unknown protein
Cit.36935.1.S1_s_at	1871.325	490.786	3.8129	AT1G19640.1	2.00E-91	JMT (JASMONIC ACID CARBOXYL METHYLTRANSFERASE); jasmonate O-
Cit.12431.1.S1_s_at	1032.906	271.748	3.801	AT4G19840.1	1.00E-36	ATPP2-A1; carbohydrate binding
Cit.38996.1.S1_s_at	193.4334	50.8917	3.8009	AT3G03870.2	1.00E-37	unknown protein
Cit.3036.1.S1_s_at	1305.133	344.1	3.7929	AT3G04920.1	9.00E-63	40S ribosomal protein S24 (RPS24A)
Cit.8541.1.S1_at	105.5264	27.8576	3.7881	AT4G02380.1	4.00E-20	SAG21 (SENESCENCE-ASSOCIATED GENE 21)
Cit.14472.1.S1_s_at	1097.217	289.96	3.784		NA	
Cit.17018.1.S1_s_at	477.7991	126.312	3.7827	AT1G23040.1	3.00E-31	hydroxyproline-rich glycoprotein family protein
Cit.21295.1.S1_at	229.6241	60.9297	3.7687		NA	
Cit.18188.1.S1_at	98.72657	26.3601	3.7453		NA	
Cit.17886.1.S1_s_at	499.4943	133.59	3.739		NA	
Cit.29688.1.S1_s_at	148.6814	39.7946	3.7362	AT3G61890.1	5.00E-40	ATHB-12 (ARABIDOPSIS THALIANA HOMEOBOX 12); transcription

Cit.4938.1.S1_at	170.7264	45.8009	3.7276	AT4G01070.1	1.00E-142	GT72B1; UDP-glucosyltransferase/ UDP-glycosyltransferase/
Cit.1891.1.S1_at	233.7216	62.7476	3.7248	AT4G31290.1	2.00E-95	ChaC-like family protein
Cit.10277.1.S1_s_at	5478.436	1473.64	3.7176	AT3G57520.1	0	AtSIP2 (Arabidopsis thaliana seed imbibition 2); hydrolase,
Cit.18105.1.S1_s_at	575.1086	155.157	3.7066	AT5G42380.1	6.00E-29	CML37 (CALMODULIN LIKE 37); calcium ion binding
Cit.25840.1.S1_s_at	2439.723	658.513	3.7049	AT1G17840.1	0	WBC11 (WHITE-BROWN COMPLEX HOMOLOG PROTEIN 11); ATPase,
Cit.11391.1.S1_at	81.76111	22.0697	3.7047	AT2G29060.1	6.00E-84	scarecrow transcription factor family protein
Cit.12431.1.S1_at	642.6005	174.152	3.6899	AT4G19840.1	1.00E-26	ATPP2-A1; carbohydrate binding
Cit.8649.1.S1_x_at	413.7986	112.201	3.688	AT5G39150.1	2.00E-80	germin-like protein, putative
Cit.7568.1.S1_at	208.3426	56.6855	3.6754	AT5G04010.1	0.0000003	unknown protein
Cit.18662.1.S1_at	98.43554	26.8596	3.6648	AT1G63420.1	3.00E-20	INVOLVED IN: biological_process unknown; EXPRESSED IN: 22 plant
Cit.34205.1.S1_at	151.5008	41.4016	3.6593	AT4G17280.1	7.00E-24	INVOLVED IN: multicellular organismal development; LOCATED IN:
Cit.996.1.S1_s_at	1861.634	509.113	3.6566	AT1G01470.1	6.00E-54	LEA14 (LATE EMBRYOGENESIS ABUNDANT 14)
Cit.6860.1.S1_at	351.8975	96.3206	3.6534	AT3G21690.1	6.00E-64	MATE efflux family protein
Cit.22463.1.S1_s_at	751.901	206.216	3.6462	AT4G27410.2	1.00E-110	RD26 (RESPONSIVE TO DESICCATION 26); transcription activator/
Cit.28150.1.S1_s_at	235.2118	65.1819	3.6085	AT1G19300.1	1.00E-159	PARVUS (PARVUS); polygalacturonate 4-alpha-
Cit.14926.1.S1_at	328.799	91.4095	3.597	AT2G04520.1	1.00E-73	eukaryotic translation initiation factor 1A, putative / eIF-1A, putative /
Cit.5477.1.S1_s_at	327.8114	91.2354	3.593	AT1G31130.1	2.00E-36	unknown protein
Cit.21996.1.S1_at	276.9635	77.1222	3.5912		NA	
Cit.6793.1.S1_at	123.9138	34.5548	3.586	AT4G32280.1	2E-09	IAA29 (INDOLE-3-ACETIC ACID INDUCIBLE 29); transcription factor
Cit.31055.1.S1_at	633.5611	176.729	3.5849	AT3G27880.1	5.00E-21	unknown protein
Cit.3880.1.S1_at	143.0122	39.9296	3.5816	AT3G62730.1	5.00E-93	unknown protein
Cit.14499.1.S1_s_at	505.1751	141.918	3.5596	AT2G19130.1	1.00E-125	S-locus lectin protein kinase family protein
Cit.21476.1.S1_at	177.5798	49.9438	3.5556		NA	
Cit.31451.1.S1_s_at	146.2013	41.1952	3.549	AT1G11530.1	5.00E-37	ATCXXS1 (C-terminal cysteine residue is changed to a serine 1);
Cit.3520.1.S1_at	486.6317	137.323	3.5437	AT1G21550.1	5.00E-32	calcium-binding protein, putative
Cit.5456.1.S1_at	455.1245	128.512	3.5415		NA	
Cit.17565.1.S1_at	96.55363	27.3707	3.5276	AT3G10120.1	6.00E-30	unknown protein
Cit.16636.1.S1_at	85.88007	24.4	3.5197	AT4G17500.1	3.00E-48	ATERF-1 (ETHYLENE RESPONSIVE ELEMENT BINDING FACTOR 1); DNA
Cit.21233.1.S1_at	120.5844	34.2697	3.5187	AT2G31090.1	1.00E-30	unknown protein
Cit.18482.1.S1_x_at	200.2837	57.0078	3.5133	AT3G22240.1	7.00E-12	unknown protein
Cit.11548.1.S1_at	206.463	58.7801	3.5125	AT1G20030.2	3.00E-79	pathogenesis-related thaumatin family protein
Cit.26147.1.S1_at	69.98981	20.0769	3.4861		NA	
Cit.5367.1.S1_at	92.07452	26.4264	3.4842	AT5G15500.2	0.0000001	ankyrin repeat family protein
Cit.11903.1.S1_x_at	146.0392	41.9172	3.484	AT5G19940.1	2.00E-55	plastid-lipid associated protein PAP-related / fibrillin-related
Cit.20900.1.S1_at	2180.942	628.241	3.4715		NA	

Cit.31262.1.S1_at	351.827	101.676	3.4603		NA	
Cit.10032.1.S1_x_at	543.3983	158.34	3.4319	AT1G75750.1	3.00E-37	GASA1 (GAST1 PROTEIN HOMOLOG 1)
Cit.2809.1.S1_s_at	678.637	197.948	3.4284	AT1G68840.1	1.00E-122	RAV2 (REGULATOR OF THE ATPASE OF THE VACUOLAR MEMBRANE);
Cit.30280.1.S1_at	112.3207	32.8443	3.4198	AT4G19640.1	2.00E-49	ARA7; GTP binding
Cit.5116.1.S1_at	133.4221	39.078	3.4143	AT1G29290.1	4.00E-13	unknown protein
Cit.29533.1.S1_s_at	2161.429	633.541	3.4117	AT5G47230.1	2.00E-60	ERF5 (ETHYLENE RESPONSIVE ELEMENT BINDING FACTOR 5); DNA
Cit.29568.1.S1_s_at	192.6703	56.5272	3.4085	AT1G01720.1	1.00E-100	ATAF1; transcription activator/ transcription factor
Cit.20852.1.S1_s_at	819.5793	242.701	3.3769	AT5G65140.1	1.00E-136	trehalose-6-phosphate phosphatase, putative
Cit.28150.1.S1_at	137.9964	40.8773	3.3759	AT1G19300.1	6.00E-39	PARVUS (PARVUS); polygalacturonate 4-alpha-
Cit.16919.1.S1_s_at	239.6809	71.0941	3.3713	AT3G51630.1	1.00E-105	WNK5 (WITH NO LYSINE (K) KINASE 5); protein kinase
Cit.14906.1.S1_at	402.1256	119.938	3.3528	AT5G65140.1	1.00E-136	trehalose-6-phosphate phosphatase, putative
Cit.17724.1.S1_s_at	96.79334	28.886	3.3509	AT4G25810.1	1.00E-130	XTR6 (XYLOGLUCAN ENDOTRANSGLYCOSYLASE 6); hydrolase, acting
Cit.4026.1.S1_s_at	4252.807	1275.58	3.334	AT4G27280.1	3.00E-42	calcium-binding EF hand family protein
Cit.18491.1.S1_at	1196.612	359.352	3.3299		NA	
Cit.580.1.S1_x_at	1521.11	456.897	3.3292	AT3G04720.1	9.00E-47	PR4 (PATHOGENESIS-RELATED 4); chitin binding
Cit.10280.1.S1_x_at	4573.484	1380.13	3.3138	AT3G57520.1	0	AtSIP2 (Arabidopsis thaliana seed imbibition 2); hydrolase,
Cit.22963.1.S1_x_at	1791.3	542.316	3.3031	AT3G19430.1	6E-08	late embryogenesis abundant protein-related / LEA protein-related
Cit.8959.1.S1_at	192.4612	58.4814	3.291	AT4G10960.1	1.00E-164	UGE5 (UDP-D-glucose/UDP-D-galactose 4-epimerase 5); UDP-glucose
Cit.16903.1.S1_x_at	3371.384	1025.72	3.2868		NA	
Cit.5891.1.S1_at	340.2386	103.525	3.2865	AT4G17500.1	3.00E-75	ATERF-1 (ETHYLENE RESPONSIVE ELEMENT BINDING FACTOR 1); DNA
Cit.3224.1.S1_s_at	516.8524	157.434	3.283	AT1G52140.1	2.00E-37	unknown protein
Cit.35594.1.S1_at	115.2561	35.1257	3.2812	AT3G07870.1	9.00E-28	F-box family protein
Cit.17929.1.S1_s_at	532.067	162.7	3.2702		NA	
Cit.30497.1.S1_at	101.0342	30.902	3.2695		NA	
Cit.10927.1.S1_s_at	934.3585	285.906	3.2681	AT3G11660.1	2.00E-81	NHL1
Cit.29868.1.S1_s_at	734.6151	225.469	3.2582	AT1G31130.1	3.00E-26	unknown protein
Cit.17840.1.S1_s_at	204.9598	63.0994	3.2482	AT1G02070.1	2.00E-13	unknown protein
Cit.9345.1.S1_at	144.9337	44.7093	3.2417	AT5G52570.1	7.00E-98	BETA-OHASE 2 (BETA-CAROTENE HYDROXYLASE 2); carotene beta-ring
Cit.753.1.S1_x_at	2848.421	879.317	3.2394	AT3G04720.1	1.00E-46	PR4 (PATHOGENESIS-RELATED 4); chitin binding
Cit.12743.1.S1_at	80.7242	24.9222	3.239	AT2G29420.1	9.00E-55	ATGSTU7 (ARABIDOPSIS THALIANA GLUTATHIONE S-TRANSFERASE
Cit.21310.1.S1_s_at	998.9852	308.961	3.2334	AT4G17500.1	3.00E-75	ATERF-1 (ETHYLENE RESPONSIVE ELEMENT BINDING FACTOR 1); DNA
Cit.35435.1.S1_x_at	2710.775	838.744	3.2319	AT5G39110.1	6.00E-79	germin-like protein, putative
Cit.30596.1.S1_at	130.642	40.4869	3.2268	AT3G19615.1	3.00E-13	unknown protein
Cit.2900.1.S1_at	298.0916	93.3475	3.1934	AT1G73010.1	1.00E-110	phosphatase
Cit.32798.1.S1_at	79.63719	24.9527	3.1915	AT5G42900.3	0.0000005	unknown protein

Cit.20000.1.S1_at	198.213	62.2081	3.1863	AT5G07280.1	0.000001	EMS1 (EXCESS MICROSPOROCYTES1); kinase/ transmembrane
Cit.13957.1.S1_at	70.56537	22.2186	3.176	AT1G20925.1	1.00E-141	auxin efflux carrier family protein
Cit.9625.1.S1_s_at	2085.172	656.821	3.1746	AT1G61800.1	1.00E-179	GPT2; antiporter/ glucose-6-phosphate transmembrane transporter
Cit.17418.1.S1_s_at	812.2958	256.205	3.1705	AT1G17840.1	0	WBC11 (WHITE-BROWN COMPLEX HOMOLOG PROTEIN 11); ATPase,
Cit.13435.1.S1_s_at	80.49166	25.4411	3.1638	AT2G27510.1	4.00E-44	ATFD3 (ferredoxin 3); 2 iron, 2 sulfur cluster binding / electron
Cit.7047.1.S1_at	1298.544	411.414	3.1563	AT5G01710.1	1.00E-133	LOCATED IN: endomembrane system; EXPRESSED IN: sperm cell, male
Cit.15785.1.S1_at	302.5143	96.0678	3.149	AT4G20970.1	2.00E-22	basic helix-loop-helix (bHLH) family protein
Cit.27205.1.S1_at	81.53736	25.9497	3.1421	AT5G57560.1	4.00E-74	TCH4 (Touch 4); hydrolase, acting on glycosyl bonds /
Cit.22219.1.S1_s_at	379.1391	120.948	3.1347	AT3G45140.1	0	LOX2 (LIPOXYGENASE 2); lipoxygenase
Cit.6201.1.S1_at	1234.387	394.419	3.1296	AT3G21680.1	7.00E-12	unknown protein
Cit.14980.1.S1_s_at	67.35892	21.5424	3.1268		NA	
Cit.33093.1.S1_at	89.86018	28.8107	3.119		NA	
Cit.22023.1.S1_x_at	925.1531	296.827	3.1168		NA	
Cit.12233.1.S1_s_at	1036.533	332.596	3.1165	AT1G49320.1	3.00E-44	BURP domain-containing protein
Cit.25108.1.S1_at	144.1511	46.2585	3.1162	AT1G63420.1	7.00E-51	INVOLVED IN: biological_process unknown; EXPRESSED IN: 22 plant
Cit.18023.1.S1_at	519.4029	166.847	3.1131	AT1G77380.1	1.00E-100	AAP3; amino acid transmembrane transporter
Cit.2740.1.S1_at	79.87673	25.7081	3.1071		NA	
Cit.30692.1.S1_s_at	735.6823	237.12	3.1026	AT2G22500.1	1.00E-128	UCP5 (UNCOUPLING PROTEIN 5); binding
Cit.26572.1.S1_at	177.3721	57.2885	3.0961	AT5G42930.1	0.0000003	triacylglycerol lipase
Cit.18564.1.S1_at	898.559	290.634	3.0917		NA	
Cit.22653.1.S1_at	323.508	104.847	3.0855	AT3G57450.1	7.00E-16	unknown protein
Cit.2739.1.S1_at	685.5781	223.549	3.0668		NA	
Cit.2630.1.S1_at	3781.133	1236.24	3.0586		NA	
Cit.13693.1.S1_s_at	169.6111	55.5062	3.0557	AT4G38960.1	6.00E-28	zinc finger (B-box type) family protein
Cit.10057.1.S1_at	71.79879	23.5337	3.0509		NA	
Cit.21938.1.S1_s_at	409.3839	134.309	3.0481	AT3G57270.1	1.00E-109	BG1 (BETA-1,3-GLUCANASE 1); catalytic/ cation binding / hydrolase,
Cit.9240.1.S1_at	198.8998	65.3274	3.0447	AT5G25610.1	1.00E-118	RD22; nutrient reservoir
Cit.17388.1.S1_at	3133.257	1030.47	3.0406	AT2G45760.1	1.00E-25	BAP2 (BON ASSOCIATION PROTEIN 2)
Cit.8093.1.S1_x_at	859.3131	282.686	3.0398	AT5G59910.1	9.00E-69	HTB4; DNA binding
Cit.18536.1.S1_at	148.5288	48.8815	3.0385		NA	
Cit.4096.1.S1_at	96.1226	31.7115	3.0312	AT1G19300.1	1.00E-159	PARVUS (PARVUS); polygalacturonate 4-alpha-
Cit.17845.1.S1_s_at	2237.871	738.842	3.0289		NA	
Cit.19105.1.S1_at	69.84323	23.0711	3.0273	AT2G44840.1	3.00E-14	ERF13 (ETHYLENE-RESPONSIVE ELEMENT BINDING FACTOR 13); DNA
Cit.245.1.S1_at	62.31095	20.614	3.0227	AT5G02970.1	4.00E-58	hydrolase, alpha/beta fold family protein
Cit.9873.1.S1_at	172.9768	57.3011	3.0187	AT4G32330.3	4.00E-12	FUNCTIONS IN: molecular_function unknown; INVOLVED IN:

Cit.36841.1.S1_s_at	260.252	86.4604	3.0101	AT1G21550.1	2.00E-32	calcium-binding protein, putative
Cit.14889.1.S1_at	60.64881	20.2035	3.0019	AT2G23620.1	4.00E-83	MES1 (METHYL ESTERASE 1); hydrolase, acting on ester bonds /
Cit.26534.1.S1_s_at	237.3626	79.108	3.0005	AT3G21690.1	6.00E-64	MATE efflux family protein
Cit.20852.1.S1_at	116.8074	38.9644	2.9978	AT4G39770.1	2.00E-20	trehalose-6-phosphate phosphatase, putative
Cit.32003.1.S1_at	129.6359	43.3261	2.9921		NA	
Cit.12040.1.S1_s_at	788.3515	263.807	2.9884	AT4G08570.1	2.00E-60	heavy-metal-associated domain-containing protein / copper
Cit.16071.1.S1_at	203.3345	68.0495	2.988	AT3G21270.1	0.000003	ADOF2; DNA binding / transcription factor
Cit.12959.1.S1_at	92.47517	30.9569	2.9872	AT1G01200.1	8.00E-89	ATRABA3 (ARABIDOPSIS RAB GTPASE HOMOLOG A3); GTP binding
Cit.18933.1.S1_at	85.07391	28.5183	2.9831	AT2G21180.1	6.00E-30	unknown protein
Cit.16508.1.S1_at	86.52156	29.0072	2.9828	AT3G11760.1	2.00E-13	unknown protein
Cit.19843.1.S1_at	88.56589	29.7045	2.9816	AT1G65280.1	2.00E-25	heat shock protein binding
Cit.9029.1.S1_s_at	362.4153	121.931	2.9723	AT2G32210.1	1.00E-23	unknown protein
Cit.29307.1.S1_at	65.06738	21.933	2.9666		NA	
Cit.6308.1.S1_at	75.98299	25.6464	2.9627	AT2G29420.1	9.00E-60	ATGSTU7 (ARABIDOPSIS THALIANA GLUTATHIONE S-TRANSFERASE
Cit.5918.1.S1_at	625.3815	212.275	2.9461	AT4G04955.1	2.00E-89	ATALN (Arabidopsis allantoinase); allantoinase/ hydrolase
Cit.17596.1.S1_s_at	964.8016	327.831	2.943		NA	
Cit.30448.1.S1_x_at	105.1515	35.7511	2.9412	AT4G02380.1	5.00E-20	SAG21 (SENESCENCE-ASSOCIATED GENE 21)
Cit.1200.1.S1_s_at	854.2685	290.537	2.9403	AT4G11650.1	9.00E-81	ATOSM34 (osmotin 34)
Cit.8903.1.S1_x_at	81.89404	27.9384	2.9312		NA	
Cit.19313.1.S1_s_at	372.8081	127.356	2.9273	AT4G22505.1	0.0000002	INVOLVED IN: lipid transport; CONTAINS InterPro DOMAIN/s:
Cit.35249.1.S1_at	694.7366	237.752	2.9221	AT3G07360.1	5.00E-27	PUB9 (PLANT U-BOX 9); ubiquitin-protein ligase
Cit.23565.1.S1_at	267.7272	91.6518	2.9211	AT3G25250.1	2.00E-60	AGC2-1 (OXIDATIVE SIGNAL-INDUCIBLE1); kinase
Cit.17228.1.S1_x_at	154.0652	52.8061	2.9176	AT4G34135.1	1.00E-131	UGT73B2 (UDP-GLUCOSYLTRANSFERASE 73B2); UDP-
Cit.38467.1.S1_at	533.0372	182.895	2.9144	AT4G20880.1	2.00E-10	ethylene-responsive nuclear protein / ethylene-regulated nuclear
Cit.21486.1.S1_x_at	8451.495	2900.36	2.9139		NA	
Cit.6800.1.S1_at	95.43906	32.8	2.9097	AT1G15130.1	5.00E-97	hydroxyproline-rich glycoprotein family protein
Cit.10062.1.S1_at	224.6907	77.2881	2.9072	AT3G47340.1	1.00E-19	ASN1 (GLUTAMINE-DEPENDENT ASPARAGINE SYNTHASE 1);
Cit.5514.1.S1_at	334.4569	115.07	2.9065	AT3G63060.1	4.00E-56	EDL3 (EID1-like 3)
Cit.29403.1.S1_x_at	2102.686	724.595	2.9019	AT4G12300.1	5.00E-58	CYP706A4; electron carrier/ heme binding / iron ion binding /
Cit.2630.1.S1_a_at	5253.185	1813.02	2.8975		NA	
Cit.39006.1.S1_at	3134.374	1082.45	2.8956	AT3G61930.1	0.00008	unknown protein
Cit.20223.1.S1_at	121.0768	41.8499	2.8931	AT5G54160.1	6.00E-64	ATOMT1 (O-METHYLTRANSFERASE 1); caffeate O-methyltransferase/
Cit.5956.1.S1_s_at	1225.731	424.304	2.8888	AT2G18660.1	7.00E-33	EXLB3 (EXPANSIN-LIKE B3 PRECURSOR)
Cit.22319.1.S1_s_at	233.8834	81.0654	2.8851	AT1G55850.1	3.00E-66	ATCSLE1; cellulose synthase/ transferase, transferring glycosyl groups
Cit.28009.1.S1_at	77.27205	26.8909	2.8735	AT1G20860.1	2.00E-57	phosphate transporter family protein

Cit.4526.1.S1_at	109.8384	38.229	2.8732	AT4G34135.2	3.00E-86	UGT73B2 (UDP-GLUCOSYLTRANSFERASE 73B2); UDP-
Cit.14998.1.S1_at	277.1739	96.5296	2.8714	AT4G03500.1	1.00E-33	ankyrin repeat family protein
Cit.14499.1.S1_at	222.8825	77.7503	2.8666	AT2G19130.1	1.00E-125	S-locus lectin protein kinase family protein
Cit.31428.1.S1_at	102.6262	35.8349	2.8639		NA	
Cit.7343.1.S1_at	216.1527	75.4917	2.8633	AT5G01830.1	1.00E-131	armadillo/beta-catenin repeat family protein / U-box domain-
Cit.22764.1.S1_at	68.73488	24.0582	2.857		NA	
Cit.17025.1.S1_at	325.3884	113.914	2.8564	AT4G12320.1	1.00E-106	CYP706A6; electron carrier/ heme binding / iron ion binding /
Cit.10567.1.S1_s_at	1305.777	458.097	2.8504	AT4G16380.1	8.00E-78	metal ion binding
Cit.5945.1.S1_at	104.5589	36.7083	2.8484	AT4G31950.1	3.00E-49	CYP82C3; electron carrier/ heme binding / iron ion binding /
Cit.35730.1.S1_x_at	582.9443	204.697	2.8478	AT1G64230.2	5.00E-84	ubiquitin-conjugating enzyme, putative
Cit.17325.1.S1_at	1924.566	676.06	2.8467	AT2G18660.1	5.00E-32	EXLB3 (EXPANSIN-LIKE B3 PRECURSOR)
Cit.29385.1.S1_at	142.2095	49.9864	2.845	AT5G52450.1	1.00E-57	MATE efflux protein-related
Cit.9020.1.S1_s_at	265.5186	93.5194	2.8392	AT4G30880.1	5.00E-21	protease inhibitor/seed storage/lipid transfer protein (LTP) family
Cit.1966.1.S1_s_at	116.25	41.0229	2.8338	AT5G13930.1	1.00E-176	TT4 (TRANSPARENT TESTA 4); naringenin-chalcone synthase
Cit.14892.1.S1_at	228.1903	80.6886	2.828	AT3G03270.2	3.00E-72	universal stress protein (USP) family protein / early nodulin ENOD18
Cit.6849.1.S1_at	92.96262	32.9277	2.8232	AT5G41800.1	1.00E-65	amino acid transporter family protein
Cit.20606.1.S1_at	62.69158	22.2214	2.8212	AT1G05575.1	1.00E-12	unknown protein
Cit.9569.1.S1_at	462.2562	164.189	2.8154		NA	
Cit.26697.1.S1_s_at	238.8713	85.0196	2.8096		NA	
Cit.11255.1.S1_at	2570.169	915.585	2.8071		NA	
Cit.11757.1.S1_at	331.5086	118.501	2.7975	AT2G41870.1	6.00E-70	remorin family protein
Cit.28173.1.S1_s_at	128.4728	45.9587	2.7954	AT4G21380.1	1.00E-106	ARK3 (A. THALIANA RECEPTOR KINASE 3); kinase/ transmembrane
Cit.20011.1.S1_at	1935.413	693.358	2.7914	AT1G78070.1	7.00E-25	FUNCTIONS IN: molecular_function unknown; INVOLVED IN:
Cit.11854.1.S1_at	119.5139	42.8435	2.7895	AT5G05340.1	1.00E-103	peroxidase, putative
Cit.3002.1.S1_s_at	265.0416	95.0528	2.7884	AT4G12320.1	1.00E-135	CYP706A6; electron carrier/ heme binding / iron ion binding /
Cit.14643.1.S1_at	56.43521	20.2515	2.7867	AT4G23496.1	9.00E-26	SP1L5 (SPIRAL1-LIKE5)
Cit.28653.1.S1_s_at	73.08949	26.2855	2.7806		NA	
Cit.3086.1.S1_at	340.1808	122.625	2.7742	AT1G68320.1	2.00E-63	MYB62 (myb domain protein 62); DNA binding / transcription factor
Cit.12735.1.S1_at	246.8554	88.9847	2.7741		NA	
Cit.30907.1.S1_at	1009.652	364.091	2.7731	AT4G19800.1	7.00E-62	glycosyl hydrolase family 18 protein
Cit.37988.1.S1_at	91.80489	33.1106	2.7727		NA	
Cit.13519.1.S1_at	838.9637	302.664	2.7719	AT1G80840.1	3.00E-71	WRKY40; transcription factor
Cit.13694.1.S1_at	314.6606	113.776	2.7656	AT4G38960.1	6.00E-28	zinc finger (B-box type) family protein
Cit.696.1.S1_x_at	117.0991	42.3427	2.7655		NA	
Cit.8198.1.S1_x_at	2496.741	904.879	2.7592		NA	

Cit.9523.1.S1_s_at	1133.063	411.248	2.7552	AT2G47770.1	6.00E-43	benzodiazepine receptor-related
Cit.25037.1.S1_at	453.2586	164.574	2.7541	AT2G31880.1	1.00E-39	leucine-rich repeat transmembrane protein kinase, putative
Cit.3450.1.S1_at	2701.332	982.341	2.7499	AT1G01720.1	1.00E-100	ATAF1; transcription activator/ transcription factor
Cit.19751.1.S1_s_at	1532.107	557.749	2.7469	AT1G17840.1	0	WBC11 (WHITE-BROWN COMPLEX HOMOLOG PROTEIN 11); ATPase,
Cit.9300.1.S1_s_at	670.0322	244.016	2.7459	AT3G28860.1	7.00E-92	ABCB19; ATPase, coupled to transmembrane movement of
Cit.3757.1.S1_at	738.2262	269.456	2.7397	AT2G46400.1	0.000002	WRKY46; transcription factor
Cit.18460.1.S1_at	60.69403	22.1659	2.7382		NA	
Cit.30622.1.S1_s_at	3087.529	1132.69	2.7258		NA	
Cit.38821.1.S1_at	87.24268	32.0062	2.7258	AT2G28660.1	4.00E-16	copper-binding family protein
Cit.17398.1.S1_at	117.0217	43.0911	2.7157	AT5G04080.1	6.00E-10	unknown protein
Cit.21553.1.S1_at	414.0833	152.576	2.7139	AT5G17280.1	1.00E-19	unknown protein
Cit.11758.1.S1_at	155.3799	57.5225	2.7012	AT5G59550.1	1.00E-81	zinc finger (C3HC4-type RING finger) family protein
Cit.16960.1.S1_x_at	172.9144	64.0256	2.7007		NA	
Cit.16993.1.S1_s_at	254.2618	94.1982	2.6992	AT3G55240.1	4.00E-36	Overexpression leads to PEL (Pseudo-Etiolation in Light) phenotype.
Cit.4258.1.S1_s_at	713.8699	264.518	2.6988	AT1G14870.1	9.00E-53	FUNCTIONS IN: molecular_function unknown; INVOLVED IN: response
Cit.712.1.S1_x_at	1340.967	497.101	2.6976		NA	
Cit.5977.1.S1_at	407.139	151.367	2.6897	AT2G38680.1	1.00E-119	5'-nucleotidase/ magnesium ion binding
Cit.19280.1.S1_at	119.2854	44.4412	2.6841	AT4G21440.1	6.00E-16	ATMYB102 (ARABIDOPSIS MYB-LIKE 102); DNA binding / transcription
Cit.21982.1.S1_at	93.3205	34.7705	2.6839	AT2G17880.1	2.00E-32	DNAJ heat shock protein, putative
Cit.16035.1.S1_at	126.6291	47.2016	2.6827	AT2G17880.1	8.00E-11	DNAJ heat shock protein, putative
Cit.603.1.S1_x_at	79.71656	29.7242	2.6819	AT1G47128.1	1.00E-128	RD21 (responsive to dehydration 21); cysteine-type endopeptidase/
Cit.25290.1.S1_s_at	66.92151	24.9617	2.681	AT3G10910.1	4.00E-48	zinc finger (C3HC4-type RING finger) family protein
Cit.3061.1.S1_at	450.5155	168.292	2.677	AT5G47230.1	2.00E-60	ERF5 (ETHYLENE RESPONSIVE ELEMENT BINDING FACTOR 5); DNA
Cit.9620.1.S1_s_at	3167.613	1185.44	2.6721	AT1G61800.1	1.00E-95	GPT2; antiporter/ glucose-6-phosphate transmembrane transporter
Cit.32844.1.S1_s_at	79.37874	29.7072	2.672	AT1G10370.1	8.00E-47	ERD9 (EARLY-RESPONSIVE TO DEHYDRATION 9); glutathione
Cit.30484.1.S1_at	62.82433	23.5481	2.6679	AT3G14470.1	0.00001	disease resistance protein (NBS-LRR class), putative
Cit.7685.1.S1_at	60.80788	22.7998	2.667	AT5G02540.1	2.00E-37	short-chain dehydrogenase/reductase (SDR) family protein
Cit.3966.1.S1_s_at	109.1815	41.0151	2.662	AT2G37130.1	1.00E-134	peroxidase 21 (PER21) (P21) (PRXR5)
Cit.21244.1.S1_at	95.8331	36.0295	2.6598	AT1G07040.1	2E-09	unknown protein
Cit.38340.1.S1_at	138.0555	51.9238	2.6588		NA	
Cit.8697.1.S1_at	613.2082	231.111	2.6533		NA	
Cit.17845.1.S1_at	1041.421	392.564	2.6529		NA	
Cit.9702.1.S1_at	206.1605	77.7526	2.6515	AT5G42050.1	1.00E-106	FUNCTIONS IN: molecular_function unknown; EXPRESSED IN: 22 plant
Cit.60.1.S1_at	175.2768	66.2637	2.6451	AT2G38540.1	1.00E-16	LP1; calmodulin binding
Cit.29672.1.S1_at	61.71803	23.3557	2.6425	AT2G35240.1	8.00E-83	plastid developmental protein DAG, putative

Cit.20299.1.S1_at	57.46178	21.8027	2.6355		NA	
Cit.22187.1.S1_s_at	62.02736	23.5805	2.6304	AT1G12570.1	1.00E-152	glucose-methanol-choline (GMC) oxidoreductase family protein
Cit.28009.1.S1_s_at	166.26	63.2473	2.6287	AT1G20860.1	0	phosphate transporter family protein
Cit.3377.1.S1_at	349.5794	133.063	2.6272	AT5G20230.1	4.00E-30	ATBCB (ARABIDOPSIS BLUE-COPPER-BINDING PROTEIN); copper ion
Cit.10279.1.S1_at	83.52789	31.8206	2.625	AT3G57520.1	0	AtSIP2 (Arabidopsis thaliana seed imbibition 2); hydrolase,
Cit.20412.1.S1_s_at	710.3013	270.645	2.6245	AT3G12500.1	1.00E-127	ATHCHIB (ARABIDOPSIS THALIANA BASIC CHITINASE); chitinase
Cit.13688.1.S1_at	431.1556	164.296	2.6243	AT2G26070.1	2.00E-95	RTE1 (REVERSION-TO-ETHYLENE SENSITIVITY1)
Cit.9134.1.S1_s_at	3983.171	1518.67	2.6228	AT4G19420.1	1.00E-174	pectinacetylesterase family protein
Cit.22980.1.S1_s_at	149.2328	56.9113	2.6222	AT1G37130.1	0	NIA2 (NITRATE REDUCTASE 2); nitrate reductase (NADH)/ nitrate
Cit.9149.1.S1_s_at	2192.671	836.96	2.6198	AT2G24260.1	5.00E-37	basic helix-loop-helix (bHLH) family protein
Cit.18432.1.S1_at	121.1721	46.2786	2.6183	AT3G54420.1	9.00E-37	ATEP3; chitinase
Cit.7903.1.S1_at	71.50224	27.359	2.6135	AT5G67590.1	2.00E-35	FRO1 (FROSTBITE1); NADH dehydrogenase (ubiquinone)
Cit.9421.1.S1_s_at	55.35035	21.1832	2.6129	AT4G25810.1	1.00E-129	XTR6 (XYLOGLUCAN ENDOTRANSGLYCOSYLASE 6); hydrolase, acting
Cit.10032.1.S1_s_at	635.9827	243.49	2.6119	AT1G75750.1	3.00E-37	GASA1 (GAST1 PROTEIN HOMOLOG 1)
Cit.17794.1.S1_s_at	3966.683	1519.98	2.6097	AT5G54940.2	6.00E-42	eukaryotic translation initiation factor SUI1, putative
Cit.14471.1.S1_at	912.8835	349.909	2.6089		NA	
Cit.5888.1.S1_at	251.5544	96.4777	2.6074	AT3G16720.1	3.00E-28	ATL2; protein binding / zinc ion binding
Cit.3449.1.S1_s_at	1147.071	440.987	2.6011	AT1G01720.1	1.00E-100	ATAF1; transcription activator/ transcription factor
Cit.17264.1.S1_s_at	140.7716	54.1999	2.5973	AT2G05540.1	4.00E-16	glycine-rich protein
Cit.7483.1.S1_at	179.4288	69.1064	2.5964		NA	
Cit.16380.1.S1_at	196.2521	75.6412	2.5945	AT5G19690.1	6.00E-23	STT3A (STAUROSPORIN AND TEMPERATURE SENSITIVE 3-LIKE A);
Cit.11156.1.S1_s_at	156.6799	60.5123	2.5892	AT4G22070.1	1.00E-149	WRKY31; transcription factor
Cit.15330.1.S1_at	129.7051	50.1363	2.5871	AT5G62470.2	1.00E-102	MYB96 (myb domain protein 96); DNA binding / transcription factor
Cit.3805.1.S1_at	196.2556	75.922	2.585	AT1G71000.1	1.00E-39	heat shock protein binding
Cit.1007.1.S1_s_at	261.4395	101.159	2.5844	AT5G25560.1	1.00E-134	zinc finger (C3HC4-type RING finger) family protein
Cit.14381.1.S1_at	453.4755	175.533	2.5834	AT5G02220.1	4E-09	unknown protein
Cit.11209.1.S1_s_at	616.2734	238.878	2.5799	AT2G40000.1	1.00E-151	HSPRO2 (ARABIDOPSIS ORTHOLOG OF SUGAR BEET HS1 PRO-1 2)
Cit.30545.1.S1_at	153.2744	59.4758	2.5771	AT5G48150.2	1.00E-52	PAT1 (phytochrome a signal transduction 1); signal transducer/
Cit.20731.1.S1_at	69.77719	27.084	2.5763		NA	
Cit.29686.1.S1_at	3789.36	1472.11	2.5741	AT5G05340.1	1.00E-91	peroxidase, putative
Cit.14913.1.S1_s_at	2810.583	1093.97	2.5692	AT5G24090.1	2.00E-64	acidic endochitinase (CHIB1)
Cit.18730.1.S1_at	60.11907	23.4092	2.5682	AT4G13340.1	0.00005	leucine-rich repeat family protein / extensin family protein
Cit.17444.1.S1_at	880.2666	342.775	2.5681	AT5G10695.1	2.00E-21	unknown protein
Cit.22763.1.S1_s_at	122.7171	47.8289	2.5658	AT3G23240.1	8.00E-52	ERF1 (ETHYLENE RESPONSE FACTOR 1); DNA binding / transcription
Cit.9706.1.S1_s_at	123.2936	48.093	2.5636	AT3G57270.1	1.00E-106	BG1 (BETA-1,3-GLUCANASE 1); catalytic/ cation binding / hydrolase,

Cit.12163.1.S1_s_at	784.1552	306.025	2.5624	AT4G17900.1	4.00E-94	zinc-binding family protein
Cit.22298.1.S1_s_at	4194.377	1639.17	2.5588	AT1G06550.1	3.00E-30	enoyl-CoA hydratase/isomerase family protein
Cit.30024.1.S1_at	333.5654	130.358	2.5588	AT1G24020.1	0.000004	MLP423 (MLP-LIKE PROTEIN 423)
Cit.25122.1.S1_s_at	730.0255	285.393	2.558	AT3G57270.1	1.00E-109	BG1 (BETA-1,3-GLUCANASE 1); catalytic/ cation binding / hydrolase,
Cit.16828.1.S1_at	113.0939	44.2576	2.5554	AT3G10960.1	1.00E-44	xanthine/uracil permease family protein
Cit.38.1.S1_x_at	197.9134	77.5311	2.5527		NA	
Cit.30943.1.S1_s_at	488.4174	191.629	2.5488	AT1G79160.1	7.00E-27	unknown protein
Cit.21630.1.S1_at	486.9848	191.297	2.5457	AT5G25560.1	3.00E-34	zinc finger (C3HC4-type RING finger) family protein
Cit.7812.1.S1_at	88.09346	34.6419	2.543	AT2G15910.1	8.00E-74	CSL zinc finger domain-containing protein
Cit.7094.1.S1_at	90.69334	35.6803	2.5418	AT3G51580.1	7.00E-31	unknown protein
Cit.30542.1.S1_s_at	1851.403	728.404	2.5417	AT5G67360.1	0	ARA12; serine-type endopeptidase
Cit.16801.1.S1_at	361.9235	142.434	2.541	AT5G42610.1	3.00E-44	unknown protein
Cit.4069.1.S1_x_at	52.57941	20.7558	2.5332	AT1G59950.1	1.00E-105	aldo/keto reductase, putative
Cit.14453.1.S1_at	499.2302	197.087	2.533	AT3G25830.1	1.00E-138	ATTPS-CIN (terpene synthase-like sequence-1,8-cineole); (E)-beta-
Cit.17750.1.S1_x_at	1244.005	491.131	2.5329		NA	
Cit.17416.1.S1_at	110.5637	43.6618	2.5323		NA	
Cit.11014.1.S1_s_at	803.1512	317.508	2.5295	AT5G59845.1	1.00E-32	gibberellin-regulated family protein
Cit.7482.1.S1_at	71.11575	28.1842	2.5232	AT5G04860.1	4.00E-15	unknown protein
Cit.18273.1.S1_at	359.0198	142.364	2.5218		NA	
Cit.22275.1.S1_x_at	62.13609	24.6503	2.5207	AT5G21274.1	1.00E-69	CAM6 (CALMODULIN 6); calcium ion binding
Cit.302.1.S1_s_at	380.1665	151.151	2.5151	AT3G12500.1	1.00E-127	ATHCHIB (ARABIDOPSIS THALIANA BASIC CHITINASE); chitinase
Cit.1319.1.S1_s_at	532.8184	211.865	2.5149	AT5G65730.1	1.00E-132	xyloglucan:xyloglucosyl transferase, putative / xyloglucan
Cit.14156.1.S1_s_at	799.8732	318.111	2.5144	AT1G73500.1	1.00E-116	MKK9 (MAP KINASE KINASE 9); MAP kinase kinase/ kinase/ protein
Cit.3817.1.S1_at	181.4115	72.2199	2.5119	AT4G22920.1	1.00E-99	NYE1 (NON-YELLOWING 1)
Cit.33490.1.S1_at	244.0018	97.2015	2.5103	AT5G59450.1	6.00E-82	scarecrow-like transcription factor 11 (SCL11)
Cit.17896.1.S1_at	571.8046	228.142	2.5064	AT4G15160.2	4.00E-71	lipid binding / structural constituent of cell wall
Cit.25191.1.S1_s_at	301.8009	120.456	2.5055	AT5G10770.1	1.00E-160	chloroplast nucleoid DNA-binding protein, putative
Cit.26995.1.S1_at	64.77966	25.895	2.5016		NA	
Cit.24483.1.S1_s_at	131.0734	52.4682	2.4981	AT4G37870.1	1.00E-164	PCK1 (PHOSPHOENOLPYRUVATE CARBOXYKINASE 1); ATP binding /
Cit.6891.1.S1_at	388.9993	156.02	2.4933	AT1G56140.1	8.00E-65	leucine-rich repeat family protein / protein kinase family protein
Cit.26116.1.S1_at	61.74452	24.807	2.489	AT1G12740.1	3.00E-64	CYP87A2; electron carrier/ heme binding / iron ion binding /
Cit.34456.1.S1_at	320.8172	128.922	2.4885		NA	
Cit.29559.1.S1_s_at	427.1198	171.826	2.4858	AT3G05200.1	4.00E-83	ATL6; protein binding / zinc ion binding
Cit.1621.1.S1_at	119.0499	47.9537	2.4826	AT1G64660.1	0	ATMGL (ARABIDOPSIS THALIANA METHIONINE GAMMA-LYASE);
Cit.20561.1.S1_at	1218.925	491.137	2.4818		NA	

Cit.10334.1.S1_s_at	445.4808	179.555	2.481	AT1G37130.1	0	NIA2 (NITRATE REDUCTASE 2); nitrate reductase (NADH)/ nitrate
Cit.1711.1.S1_at	88.70466	35.7566	2.4808	AT1G62940.1	3.00E-89	ACOS5 (ACYL-COA SYNTHETASE 5); 4-coumarate-CoA ligase/ long-
Cit.30905.1.S1_at	2123.806	857.832	2.4758	AT5G17350.1	3.00E-27	unknown protein
Cit.35813.1.S1_at	112.6991	45.5394	2.4748	AT1G34420.1	2.00E-40	leucine-rich repeat family protein / protein kinase family protein
Cit.16929.1.S1_x_at	69.93323	28.2606	2.4746		NA	
Cit.29240.1.S1_x_at	109.1459	44.1476	2.4723	AT5G08640.1	3.00E-38	FLS (FLAVONOL SYNTHASE); flavonol synthase
Cit.33196.1.S1_at	54.99085	22.2768	2.4685	AT1G65690.1	8.00E-74	harpin-induced protein-related / HIN1-related / harpin-responsive
Cit.22941.1.S1_x_at	80.22264	32.499	2.4685	AT5G25170.1	2.00E-47	unknown protein
Cit.12739.1.S1_at	525.114	212.805	2.4676		NA	
Cit.12503.1.S1_at	1013.733	411.575	2.4631	AT3G45600.1	1.00E-133	TET3 (TETRASPANIN3)
Cit.7072.1.S1_at	72.2398	29.3562	2.4608	AT2G23220.1	7.00E-27	CYP81D6; electron carrier/ heme binding / iron ion binding /
Cit.21556.1.S1_x_at	10810.44	4402.55	2.4555	AT1G10310.1	6.00E-23	short-chain dehydrogenase/reductase (SDR) family protein
Cit.162.1.S1_x_at	10883.33	4445.36	2.4482		NA	
Cit.1648.1.S1_at	74.84479	30.605	2.4455	AT2G37940.1	2.00E-42	FUNCTIONS IN: molecular_function unknown; INVOLVED IN:
Cit.24426.1.S1_s_at	197.5756	80.8318	2.4443	AT1G73010.1	1.00E-110	phosphatase
Cit.11855.1.S1_s_at	2038.158	835.064	2.4407	AT5G05340.1	1.00E-103	peroxidase, putative
Cit.14222.1.S1_at	283.6819	116.314	2.4389	AT1G58170.1	8.00E-56	disease resistance-responsive protein-related / dirigent protein-
Cit.2860.1.S1_s_at	705.7013	289.366	2.4388	AT1G33060.2	3.00E-15	no apical meristem (NAM) family protein
Cit.29098.1.S1_at	53.68831	22.0286	2.4372	AT4G25310.1	4.00E-19	oxidoreductase, 2OG-Fe(II) oxygenase family protein
Cit.21111.1.S1_at	127.6185	52.3706	2.4368	AT1G28710.1	6.00E-51	unknown protein
Cit.12502.1.S1_s_at	1310.814	538.258	2.4353	AT3G45600.1	1.00E-133	TET3 (TETRASPANIN3)
Cit.14999.1.S1_s_at	561.8344	230.711	2.4352	AT4G03500.1	1.00E-33	ankyrin repeat family protein
Cit.25526.1.S1_s_at	850.946	350.244	2.4296	AT3G14620.1	3.00E-55	CYP72A8; electron carrier/ heme binding / iron ion binding /
Cit.594.1.S1_at	75.11725	30.9409	2.4278		NA	
Cit.36653.1.S1_at	96.54305	39.8264	2.4241		NA	
Cit.22363.1.S1_x_at	59.88145	24.7331	2.4211	AT1G52340.1	0.000005	ABA2 (ABA DEFICIENT 2); alcohol dehydrogenase/ oxidoreductase/
Cit.20765.1.S1_at	84.27192	34.808	2.421		NA	
Cit.30231.1.S1_at	87.98155	36.349	2.4205	AT5G65500.1	5.00E-54	ATP binding / kinase/ protein kinase/ protein serine/threonine
Cit.33104.1.S1_at	52.77032	21.8312	2.4172	AT5G62480.2	4.00E-12	ATGSTU9 (ARABIDOPSIS THALIANA GLUTATHIONE S-TRANSFERASE
Cit.33707.1.S1_at	648.9885	268.724	2.4151	AT4G27900.2	9.00E-26	FUNCTIONS IN: molecular_function unknown; INVOLVED IN:
Cit.36604.1.S1_at	56.17801	23.2917	2.4119	AT3G17520.1	2.00E-17	late embryogenesis abundant domain-containing protein / LEA
Cit.25956.1.S1_x_at	156.9224	65.0717	2.4115	AT1G26690.1	2E-08	emp24/gp25L/p24 family protein
Cit.14184.1.S1_at	344.231	142.858	2.4096	AT5G10930.1	2.00E-66	CIPK5 (CBL-INTERACTING PROTEIN KINASE 5); ATP binding / kinase/
Cit.20369.1.S1_at	75.4585	31.345	2.4074	AT2G47370.1	1.00E-22	FUNCTIONS IN: molecular_function unknown; INVOLVED IN:
Cit.4474.1.S1_s_at	152.6933	63.5058	2.4044	AT4G15610.1	2.00E-48	integral membrane family protein

Cit.6412.1.S1_at	289.3466	120.377	2.4037	AT2G33580.1	3.00E-32	protein kinase family protein / peptidoglycan-binding LysM domain-
Cit.23005.1.S1_at	476.7025	198.913	2.3965	AT5G19500.1	9.00E-26	tryptophan/tyrosine permease family protein
Cit.25298.1.S1_s_at	137.9438	57.6909	2.3911	AT3G05690.1	4E-08	NF-YA2 (NUCLEAR FACTOR Y, SUBUNIT A2); transcription factor
Cit.38213.1.S1_at	148.4545	62.2192	2.386	AT1G30370.1	3.00E-37	lipase class 3 family protein
Cit.26801.1.S1_x_at	107.6751	45.1544	2.3846	AT3G14470.1	8.00E-23	disease resistance protein (NBS-LRR class), putative
Cit.11296.1.S1_at	641.2142	269.189	2.382	AT5G37540.1	2.00E-87	aspartyl protease family protein
Cit.13915.1.S1_at	673.5831	282.8	2.3818	AT3G14440.1	0	NCED3 (NINE-CIS-EPOXYCAROTENOID DIOXYGENASE 3); 9-cis-
Cit.21326.1.S1_at	453.9254	190.927	2.3775		NA	
Cit.21516.1.S1_at	50.79017	21.3777	2.3758		NA	
Cit.30945.1.S1_at	57.74023	24.3061	2.3755	AT2G18950.1	1.00E-84	HPT1 (HOMOGENTISATE PHYTYLTRANSFERASE 1); homogentisate
Cit.6876.1.S1_at	261.5393	110.351	2.3701	AT1G35430.1	1.00E-27	unknown protein
Cit.29504.1.S1_s_at	65.0899	27.4643	2.37	AT4G20820.1	1.00E-151	FAD-binding domain-containing protein
Cit.14919.1.S1_at	789.6721	333.214	2.3699	AT3G44735.1	5.00E-13	PSK1; growth factor
Cit.1707.1.S1_s_at	3769.439	1590.67	2.3697	AT1G18740.1	1.00E-134	unknown protein
Cit.30469.1.S1_x_at	60.45779	25.514	2.3696	AT1G68725.1	0.000005	AGP19 (ARABINOGALACTAN-PROTEIN 19)
Cit.3195.1.S1_s_at	348.0738	147.038	2.3672	AT1G33055.1	7.00E-13	unknown protein
Cit.4682.1.S1_at	211.5621	89.6065	2.361	AT2G31880.1	0	leucine-rich repeat transmembrane protein kinase, putative
Cit.1775.1.S1_s_at	335.9491	142.374	2.3596	AT1G56600.1	1.00E-156	AtGolS2 (Arabidopsis thaliana galactinol synthase 2); transferase,
Cit.1005.1.S1_s_at	130.482	55.3383	2.3579	AT4G31500.1	1.00E-142	CYP83B1 (CYTOCHROME P450 MONOOXYGENASE 83B1);
Cit.24463.1.S1_s_at	113.2814	48.0586	2.3572	AT3G14680.1	1.00E-64	CYP72A14; electron carrier/ heme binding / iron ion binding /
Cit.15887.1.S1_at	154.5555	65.5925	2.3563	AT3G59940.1	2.00E-50	kelch repeat-containing F-box family protein
Cit.18411.1.S1_at	88.30513	37.4784	2.3562	AT5G20110.1	5.00E-45	dynein light chain, putative
Cit.7553.1.S1_at	112.8794	47.9166	2.3557	AT2G01300.1	5.00E-19	unknown protein
Cit.10672.1.S1_s_at	132.0737	56.1764	2.3511	AT5G07050.1	1.00E-155	LOCATED IN: membrane; CONTAINS InterPro DOMAIN/s: Protein of
Cit.3069.1.S1_at	137.443	58.5515	2.3474	AT5G50915.2	3.00E-44	basic helix-loop-helix (bHLH) family protein
Cit.11983.1.S1_s_at	89.07384	37.956	2.3468	AT3G44990.1	1.00E-126	XTR8 (XYLOGLUCAN ENDO-TRANSGLYCOSYLASE-RELATED 8);
Cit.17360.1.S1_s_at	65.7971	28.0715	2.3439		NA	
Cit.17567.1.S1_s_at	374.1106	159.627	2.3437	AT5G40800.1	7.00E-32	unknown protein
Cit.12819.1.S1_at	539.7656	230.364	2.3431	AT2G46210.1	1.00E-107	delta-8 sphingolipid desaturase, putative
Cit.26377.1.S1_x_at	240.2299	102.546	2.3427	AT5G17165.1	0.00002	unknown protein
Cit.28608.1.S1_at	53.62571	22.9038	2.3413	AT2G01170.1	7.00E-41	BAT1 (BIDIRECTIONAL AMINO ACID TRANSPORTER 1); amino acid
Cit.17675.1.S1_at	873.3774	373.465	2.3386	AT1G26250.1	2.00E-29	proline-rich extensin, putative
Cit.18251.1.S1_at	48.81052	20.872	2.3386	AT5G05300.1	0.000004	unknown protein
Cit.11178.1.S1_s_at	2016.932	862.671	2.338	AT5G35735.1	1.00E-132	auxin-responsive family protein
Cit.23393.1.S1_x_at	177.8107	76.1086	2.3363	AT5G21160.1	2.00E-24	La domain-containing protein / proline-rich family protein

Cit.23564.1.S1_at	137.2398	58.8085	2.3337	AT5G20250.3	2.00E-53	DIN10 (DARK INDUCIBLE 10); hydrolase, hydrolyzing O-glycosyl
Cit.25990.1.S1_at	695.4115	298.403	2.3304	AT1G49640.1	0.000005	hydrolase
Cit.29611.1.S1_at	205.9574	88.4909	2.3274	AT4G23810.1	4.00E-32	WRKY53; DNA binding / protein binding / transcription activator/
Cit.15995.1.S1_at	74.15351	31.8851	2.3257		NA	
Cit.39074.1.S1_at	156.3443	67.2404	2.3252	AT5G01015.1	2.00E-21	unknown protein
Cit.10595.1.S1_at	95.3342	41.0178	2.3242		NA	
Cit.2899.1.S1_s_at	169.7643	73.0482	2.324	AT1G73010.1	1.00E-110	phosphatase
Cit.39387.1.S1_at	55.21267	23.7789	2.3219	AT1G67750.1	1.00E-108	pectate lyase family protein
Cit.14989.1.S1_at	158.0835	68.0837	2.3219	AT2G16790.1	6.00E-56	shikimate kinase family protein
Cit.26830.1.S1_s_at	55.16554	23.7629	2.3215	AT4G10380.1	3.00E-67	NIP5;1; arsenite transmembrane transporter/ boron transporter/
Cit.29429.1.S1_s_at	1748.196	753.204	2.321	AT1G18740.1	1.00E-134	unknown protein
Cit.26491.1.S1_at	59.43643	25.6126	2.3206	AT2G38820.1	1.00E-12	unknown protein
Cit.11383.1.S1_s_at	1189.781	513.093	2.3188	AT4G38580.1	5.00E-67	ATFP6 (FARNESYLATED PROTEIN 6); metal ion binding
Cit.19898.1.S1_at	75.93768	32.7667	2.3175		NA	
Cit.24350.1.S1_at	47.7611	20.6091	2.3175	AT3G08510.2	2.00E-76	ATPLC2 (PHOSPHOLIPASE C 2); phospholipase C
Cit.14258.1.S1_at	1340.67	578.615	2.317		NA	
Cit.12067.1.S1_s_at	1670.594	721.59	2.3152	AT3G16510.1	5.00E-65	C2 domain-containing protein
Cit.30330.1.S1_at	61.07832	26.4651	2.3079	AT5G45140.1	1.00E-129	NRPC2; DNA binding / DNA-directed RNA polymerase/ ribonucleoside
Cit.19313.1.S1_at	315.9258	137.029	2.3055	AT5G19340.1	0.00002	unknown protein
Cit.26654.1.S1_x_at	58.58242	25.4386	2.3029		NA	
Cit.3339.1.S1_at	55.76256	24.2601	2.2985	AT1G12570.1	1.00E-152	glucose-methanol-choline (GMC) oxidoreductase family protein
Cit.23081.1.S1_x_at	48.10761	20.9534	2.2959	AT1G53280.1	3.00E-42	DJ-1 family protein
Cit.3215.1.S1_s_at	188.6319	82.1939	2.295	AT5G03230.1	8.00E-41	unknown protein
Cit.19339.1.S1_s_at	422.5528	184.235	2.2936	AT5G01750.2	5.00E-70	unknown protein
Cit.17907.1.S1_at	231.4538	100.931	2.2932	AT1G49320.1	3.00E-44	BURP domain-containing protein
Cit.35267.1.S1_at	50.49221	22.0426	2.2907	AT5G65790.1	4.00E-57	MYB68 (MYB DOMAIN PROTEIN 68); DNA binding / transcription
Cit.23237.1.S1_s_at	898.3488	392.233	2.2903	AT1G29670.1	1.00E-155	GDSL-motif lipase/hydrolase family protein
Cit.28375.1.S1_at	53.14675	23.2175	2.2891	AT5G01330.1	4.00E-55	PDC3 (pyruvate decarboxylase-3); carboxy-lyase/ catalytic/
Cit.10522.1.S1_s_at	3263.806	1425.86	2.289	AT5G67300.1	2.00E-87	MYBR1 (MYB DOMAIN PROTEIN R1); DNA binding / transcription
Cit.7639.1.S1_at	1226.593	536.129	2.2879	AT5G63905.1	2.00E-29	unknown protein
Cit.193.1.S1_s_at	238.7318	104.374	2.2873	AT5G53370.1	0	PMEPCRF (PECTIN METHYLESTERASE PCR FRAGMENT F);
Cit.14228.1.S1_at	54.0704	23.6582	2.2855	AT3G01820.1	5.00E-74	adenylate kinase family protein
Cit.29911.1.S1_at	113.0951	49.6139	2.2795	AT5G40270.1	1.00E-107	metal-dependent phosphohydrolase HD domain-containing protein
Cit.12639.1.S1_at	87.09502	38.2232	2.2786	AT3G27080.1	4.00E-67	TOM20-3 (TRANSLOCASE OF OUTER MEMBRANE 20 KDA SUBUNIT 3);
Cit.25526.1.S1_at	49.65922	21.8065	2.2773	AT3G14620.1	3.00E-55	CYP72A8; electron carrier/ heme binding / iron ion binding /

Cit.24884.1.S1_at	304.6562	133.959	2.2743	AT1G48300.1	1.00E-15	unknown protein
Cit.15080.1.S1_at	53.30455	23.4419	2.2739	AT1G25540.1	7.00E-10	PFT1 (PHYTOCHROME AND FLOWERING TIME 1); transcription
Cit.8326.1.S1_at	66.60153	29.3007	2.273	AT1G74270.1	1.00E-47	60S ribosomal protein L35a (RPL35aC)
Cit.39904.1.S1_at	52.04727	22.904	2.2724	AT3G48520.1	1.00E-16	CYP94B3; electron carrier/ heme binding / iron ion binding /
Cit.26009.1.S1_x_at	64.41547	28.4053	2.2677		NA	
Cit.28448.1.S1_at	53.33878	23.529	2.2669		NA	
Cit.21962.1.S1_at	140.618	62.0304	2.2669	AT2G36760.1	8.00E-59	UGT73C2 (UDP-glucosyl transferase 73C2); UDP-glycosyltransferase/
Cit.21700.1.S1_at	300.3086	132.667	2.2636	AT3G52430.1	8.00E-43	PAD4 (PHYTOALEXIN DEFICIENT 4); lipase/ protein binding /
Cit.26552.1.S1_at	61.12286	27.0174	2.2624	AT5G65380.1	6.00E-17	ripening-responsive protein, putative
Cit.3694.1.S1_s_at	394.6773	174.572	2.2608	AT1G07430.1	1.00E-122	protein phosphatase 2C, putative / PP2C, putative
Cit.10669.1.S1_s_at	2902.466	1284.32	2.2599	AT2G15960.1	7E-09	unknown protein
Cit.15681.1.S1_at	48.9648	21.6679	2.2598	AT4G13840.1	4.00E-93	transferase family protein
Cit.6160.1.S1_at	139.9131	61.9249	2.2594	AT2G32510.1	2.00E-86	MAPKKK17; ATP binding / kinase/ protein kinase/ protein
Cit.6364.1.S1_s_at	50.15793	22.2293	2.2564	AT2G15220.1	3.00E-83	secretory protein, putative
Cit.38990.1.S1_at	88.01788	39.0363	2.2548	AT1G21000.2	1.00E-100	zinc-binding family protein
Cit.9089.1.S1_s_at	404.717	179.606	2.2534	AT2G45550.1	1.00E-134	CYP76C4; electron carrier/ heme binding / iron ion binding /
Cit.1866.1.S1_s_at	1510.81	670.542	2.2531	AT2G22470.1	9.00E-24	AGP2 (ARABINOGALACTAN PROTEIN 2)
Cit.30989.1.S1_s_at	477.2587	211.91	2.2522	AT4G15248.1	2.00E-21	zinc ion binding
Cit.8537.1.S1_at	260.8354	115.829	2.2519	AT3G01990.1	9.00E-66	ACR6; amino acid binding
Cit.10366.1.S1_s_at	446.4958	198.349	2.2511	AT1G56300.1	3.00E-57	DNAJ heat shock N-terminal domain-containing protein
Cit.5518.1.S1_at	549.9943	244.483	2.2496	AT5G48150.2	7.00E-77	PAT1 (phytochrome a signal transduction 1); signal transducer/
Cit.11459.1.S1_s_at	351.7835	156.417	2.249	AT1G05300.1	1.00E-109	ZIP5; cation transmembrane transporter/ metal ion transmembrane
Cit.28954.1.S1_at	71.83363	31.9606	2.2476	AT3G26160.1	4.00E-39	CYP71B17; electron carrier/ heme binding / iron ion binding /
Cit.12926.1.S1_at	64.93336	28.9417	2.2436		NA	
Cit.35713.1.S1_at	365.7869	163.116	2.2425		NA	
Cit.20223.1.S1_x_at	126.3207	56.3795	2.2405	AT5G54160.1	6.00E-64	ATOMT1 (O-METHYLTRANSFERASE 1); caffeate O-methyltransferase/
Cit.3693.1.S1_at	950.4135	424.302	2.2399	AT2G29380.1	9.00E-68	protein phosphatase 2C, putative / PP2C, putative
Cit.40400.1.S1_at	489.2411	218.541	2.2387	AT3G57450.1	1.00E-14	unknown protein
Cit.11758.1.S1_s_at	2192.099	979.996	2.2368	AT5G59550.1	1.00E-81	zinc finger (C3HC4-type RING finger) family protein
Cit.10521.1.S1_at	430.5383	192.756	2.2336		NA	
Cit.9702.1.S1_x_at	219.0894	98.1102	2.2331	AT5G42050.1	1.00E-106	FUNCTIONS IN: molecular_function unknown; EXPRESSED IN: 22 plant
Cit.26053.1.S1_x_at	123.0457	55.109	2.2328		NA	
Cit.31510.1.S1_at	117.2758	52.5281	2.2326	AT5G44360.1	1.00E-60	FAD-binding domain-containing protein
Cit.30902.1.S1_at	95.59789	42.9161	2.2276	AT1G44191.1	7.00E-11	Encodes a ECA1 gametogenesis related family protein
Cit.5936.1.S1_at	164.9071	74.0964	2.2256	AT4G30470.1	1.00E-123	cinnamoyl-CoA reductase-related

Cit.40377.1.S1_s_at	83.18777	37.383	2.2253	AT3G09270.1	4.00E-50	ATGSTU8 (GLUTATHIONE S-TRANSFERASE TAU 8); glutathione
Cit.38354.1.S1_at	80.42962	36.1738	2.2234	AT4G00110.1	1.00E-31	GAE3 (UDP-D-GLUCURONATE 4-EPIMERASE 3); UDP-glucuronate 4-
Cit.17208.1.S1_at	1284.139	577.654	2.223	AT4G25000.1	1.00E-174	AMY1 (ALPHA-AMYLASE-LIKE); alpha-amylase
Cit.27935.1.S1_s_at	909.0344	409.299	2.221	AT2G47260.1	2.00E-70	WRKY23; transcription factor
Cit.2772.1.S1_at	85.42784	38.489	2.2195	AT4G37608.1	0.00002	unknown protein
Cit.18120.1.S1_at	97.85576	44.1089	2.2185	AT5G56550.1	2.00E-21	OXS3 (OXIDATIVE STRESS 3)
Cit.31971.1.S1_at	137.3695	61.9932	2.2159	AT5G06800.1	6.00E-21	myb family transcription factor
Cit.1320.1.S1_s_at	630.4462	285.151	2.2109	AT5G65730.1	1.00E-132	xyloglucan:xyloglucosyl transferase, putative / xyloglucan
Cit.31497.1.S1_at	121.1998	54.8278	2.2106	AT4G18910.1	3.00E-58	NIP1;2 (NOD26-LIKE INTRINSIC PROTEIN 1;2); arsenite
Cit.17896.1.S1_s_at	236.0154	106.797	2.2099	AT4G15160.2	4.00E-71	lipid binding / structural constituent of cell wall
Cit.23815.1.S1_s_at	93.40408	42.271	2.2097	AT3G05690.1	4E-08	NF-YA2 (NUCLEAR FACTOR Y, SUBUNIT A2); transcription factor
Cit.27926.1.S1_at	481.566	218.203	2.207		NA	
Cit.30110.1.S1_at	156.4442	70.9433	2.2052		NA	
Cit.1710.1.S1_s_at	2775.697	1259.7	2.2035	AT1G74450.1	3.00E-11	unknown protein
Cit.5651.1.S1_s_at	344.1436	156.256	2.2024	AT1G77210.1	2.00E-68	sugar transporter, putative
Cit.17961.1.S1_s_at	169.4128	76.932	2.2021	AT3G54040.1	8.00E-50	photoassimilate-responsive protein-related
Cit.32870.1.S1_at	70.56355	32.0572	2.2012		NA	
Cit.30276.1.S1_at	56.27753	25.5716	2.2008	AT4G37300.1	0.00007	MEE59 (maternal effect embryo arrest 59)
Cit.35109.1.S1_at	45.34817	20.6497	2.1961	AT5G66350.1	4.00E-24	SHI (SHORT INTERNODES); protein binding / protein
Cit.17342.1.S1_at	540.3525	246.244	2.1944	AT5G10870.1	7.00E-57	ATCM2 (chorismate mutase 2); chorismate mutase
Cit.38590.1.S1_at	614.3776	279.983	2.1943	AT1G70170.1	2.00E-72	MMP (MATRIX METALLOPROTEINASE); metalloendopeptidase/
Cit.4605.1.S1_at	464.9675	211.938	2.1939		NA	
Cit.22421.1.S1_x_at	815.2103	372.162	2.1905	AT1G17860.1	4.00E-25	trypsin and protease inhibitor family protein / Kunitz family protein
Cit.14158.1.S1_at	66.97415	30.5832	2.1899	AT1G61560.1	1.00E-113	MLO6 (MILDEW RESISTANCE LOCUS O 6); calmodulin binding
Cit.13750.1.S1_x_at	553.2974	253.011	2.1869	AT4G17030.1	8.00E-88	ATEXLB1 (ARABIDOPSIS THALIANA EXPANSIN-LIKE B1)
Cit.6803.1.S1_s_at	54.46478	24.9094	2.1865	AT5G42930.1	2.00E-44	triacylglycerol lipase
Cit.3817.1.S1_x_at	865.2856	396.044	2.1848	AT4G22920.1	1.00E-99	NYE1 (NON-YELLOWING 1)
Cit.15245.1.S1_at	327.9955	150.177	2.1841	AT2G26690.1	1.00E-129	nitrate transporter (NTP2)
Cit.34473.1.S1_at	45.7447	20.9521	2.1833		NA	
Cit.20749.1.S1_s_at	976.4572	447.388	2.1826	AT3G11410.1	8.00E-72	PP2CA (ARABIDOPSIS THALIANA PROTEIN PHOSPHATASE 2CA);
Cit.1866.1.S1_at	236.4221	108.36	2.1818	AT2G22470.1	7.00E-23	AGP2 (ARABINOGALACTAN PROTEIN 2)
Cit.6812.1.S1_at	55.04484	25.2313	2.1816	AT1G15520.1	7.00E-29	PDR12 (PLEIOTROPIC DRUG RESISTANCE 12); ATPase, coupled to
Cit.9700.1.S1_x_at	651.4276	298.783	2.1803	AT5G42050.1	1.00E-106	FUNCTIONS IN: molecular_function unknown; EXPRESSED IN: 22 plant
Cit.36656.1.S1_at	45.33053	20.7958	2.1798	AT5G07310.1	1.00E-32	AP2 domain-containing transcription factor, putative
Cit.7578.1.S1_at	139.617	64.1226	2.1773	AT1G18980.1	1.00E-71	germin-like protein, putative

Cit.5382.1.S1_at	224.7278	103.238	2.1768	AT4G38060.2	7.00E-17	unknown protein
Cit.12682.1.S1_at	613.2735	282.082	2.1741	AT3G14460.1	2.00E-18	disease resistance protein (NBS-LRR class), putative
Cit.22693.1.S1_x_at	269.6659	124.039	2.174		NA	
Cit.29899.1.S1_at	51.22415	23.565	2.1737	AT3G07250.1	4.00E-33	nuclear transport factor 2 (NTF2) family protein / RNA recognition
Cit.1727.1.S1_s_at	144.714	66.6381	2.1716	AT3G54420.1	6.00E-76	ATEP3; chitinase
Cit.4141.1.S1_at	92.01196	42.3915	2.1705	AT4G16520.2	2.00E-51	ATG8F (autophagy 8f); microtubule binding
Cit.29704.1.S1_s_at	253.8984	117.211	2.1662	AT4G00880.1	2.00E-37	auxin-responsive family protein
Cit.7705.1.S1_at	443.1993	204.61	2.1661	AT5G53990.1	2.00E-16	glycosyltransferase family protein
Cit.11390.1.S1_s_at	297.212	137.222	2.1659	AT1G07530.1	1.00E-149	SCL14 (SCARECROW-LIKE 14); transcription factor
Cit.17215.1.S1_s_at	1031.818	477.102	2.1627	AT3G17100.2	3.00E-56	transcription factor
Cit.3876.1.S1_s_at	81.05513	37.4898	2.1621	AT5G03170.1	1.00E-68	FLA11
Cit.4934.1.S1_at	1297.117	600.012	2.1618	AT1G22360.1	1.00E-155	AtUGT85A2 (UDP-glucosyl transferase 85A2); UDP-
Cit.10425.1.S1_s_at	2249.091	1041.53	2.1594	AT5G05250.1	1.00E-42	unknown protein
Cit.16322.1.S1_at	183.8986	85.1721	2.1591	AT3G62260.1	1.00E-34	protein phosphatase 2C, putative / PP2C, putative
Cit.18116.1.S1_at	126.6977	58.7119	2.158	AT5G48850.1	3.00E-39	ATSDI1 (SULPHUR DEFICIENCY-INDUCED 1); binding
Cit.1421.1.S1_at	48.15998	22.3177	2.1579	AT5G18670.1	1.00E-112	BMY3; beta-amylase/ catalytic/ cation binding
Cit.30370.1.S1_at	747.4706	346.655	2.1562		NA	
Cit.22710.1.S1_x_at	533.5166	247.517	2.1555	AT4G11650.1	9.00E-25	ATOSM34 (osmotin 34)
Cit.28737.1.S1_at	89.70747	41.6367	2.1545	AT5G13640.1	4.00E-44	ATPDAT; phosphatidylcholine-sterol O-acyltransferase
Cit.37714.1.S1_at	47.39872	22.0033	2.1542	AT5G15920.1	1.00E-55	structural maintenance of chromosomes (SMC) family protein (MSS2)
Cit.9580.1.S1_at	75.45798	35.0464	2.1531	AT3G55610.1	7.00E-16	P5CS2 (DELTA 1-PYRROLINE-5-CARBOXYLATE SYNTHASE 2); catalytic/
Cit.18295.1.S1_at	452.4117	210.167	2.1526		NA	
Cit.23609.1.S1_at	54.68026	25.4197	2.1511	AT2G29670.1	3.00E-35	binding
Cit.22023.1.S1_s_at	3021.705	1405.13	2.1505	AT5G01710.1	1.00E-133	LOCATED IN: endomembrane system; EXPRESSED IN: sperm cell, male
Cit.6309.1.S1_at	148.5214	69.0974	2.1495	AT3G60890.1	6.00E-11	ZPR2 (LITTLE ZIPPER 2); protein binding
Cit.1751.1.S1_s_at	95.90309	44.7105	2.145	AT1G67920.1	2.00E-13	unknown protein
Cit.11650.1.S1_at	71.85733	33.5173	2.1439	AT5G22460.2	2.00E-93	esterase/lipase/thioesterase family protein
Cit.6413.1.S1_at	320.5896	149.606	2.1429		NA	
Cit.29386.1.S1_at	335.1202	156.414	2.1425	AT3G44735.1	2.00E-11	PSK1; growth factor
Cit.24967.1.S1_at	107.4323	50.171	2.1413	AT3G04730.1	4.00E-17	IAA16; transcription factor
Cit.4721.1.S1_at	49.38739	23.0749	2.1403	AT2G36970.1	2.00E-22	UDP-glucoronosyl/UDP-glucosyl transferase family protein
Cit.5712.1.S1_s_at	214.3419	100.163	2.1399	AT5G27710.1	1.00E-121	unknown protein
Cit.21424.1.S1_at	46.60881	21.8037	2.1377	AT3G48120.1	3.00E-27	unknown protein
Cit.38640.1.S1_at	51.88642	24.2744	2.1375	AT3G22600.1	7.00E-37	protease inhibitor/seed storage/lipid transfer protein (LTP) family
Cit.9822.1.S1_s_at	1324.921	619.901	2.1373	AT2G47140.1	6.00E-41	short-chain dehydrogenase/reductase (SDR) family protein

Cit.2890.1.S1_at	186.3017	87.2318	2.1357	AT3G51240.1	1.00E-177	F3H (FLAVANONE 3-HYDROXYLASE); naringenin 3-dioxygenase
Cit.4392.1.S1_s_at	520.1045	243.63	2.1348	AT1G01490.2	3.00E-41	heavy-metal-associated domain-containing protein
Cit.2237.1.S1_at	674.2772	316.152	2.1328	AT1G64980.1	1.00E-127	unknown protein
Cit.30707.1.S1_s_at	70.79585	33.207	2.132	AT5G50760.1	7.00E-31	auxin-responsive family protein
Cit.38584.1.S1_at	96.16541	45.1151	2.1316		NA	
Cit.2690.1.S1_at	45.14796	21.1953	2.1301	AT4G20820.1	1.00E-151	FAD-binding domain-containing protein
Cit.10057.1.S1_s_at	245.2199	115.213	2.1284	AT3G47340.1	1.00E-19	ASN1 (GLUTAMINE-DEPENDENT ASPARAGINE SYNTHASE 1);
Cit.37078.1.S1_s_at	351.3435	165.075	2.1284	AT5G39600.1	9.00E-49	unknown protein
Cit.2861.1.S1_at	72.21381	34.0344	2.1218	AT1G33060.2	2.00E-14	no apical meristem (NAM) family protein
Cit.28534.1.S1_s_at	528.2526	249.368	2.1184	AT3G18710.1	1.00E-33	PUB29 (PLANT U-BOX 29); ubiquitin-protein ligase
Cit.23149.1.S1_at	44.4392	20.9793	2.1182		NA	
Cit.22589.1.S1_s_at	5507.513	2600.4	2.118	AT2G38870.1	3.00E-18	protease inhibitor, putative
Cit.34251.1.S1_at	69.52987	32.8289	2.1179		NA	
Cit.29835.1.S1_at	85.47582	40.419	2.1147	AT3G23990.1	1.00E-41	HSP60 (HEAT SHOCK PROTEIN 60); ATP binding
Cit.11704.1.S1_s_at	1700.75	804.694	2.1135	AT3G15210.1	4.00E-40	ERF4 (ETHYLENE RESPONSIVE ELEMENT BINDING FACTOR 4); DNA
Cit.25113.1.S1_at	91.79803	43.4597	2.1123	AT2G19130.1	7.00E-61	S-locus lectin protein kinase family protein
Cit.26711.1.S1_at	197.0562	93.353	2.1109	AT3G04450.1	0.0000006	transcription factor
Cit.15680.1.S1_at	49.69398	23.5435	2.1107	AT5G04660.1	7.00E-99	CYP77A4; electron carrier/ heme binding / iron ion binding /
Cit.30300.1.S1_at	52.22704	24.7467	2.1105		NA	
Cit.18696.1.S1_at	1191.536	564.636	2.1103		NA	
Cit.4061.1.S1_s_at	652.4408	309.86	2.1056	AT1G07160.1	9.00E-75	protein phosphatase 2C, putative / PP2C, putative
Cit.1861.1.S1_s_at	4740.045	2252.18	2.1046	AT2G27080.2	2.00E-70	harpin-induced protein-related / HIN1-related / harpin-responsive
Cit.6332.1.S1_at	85.70369	40.7684	2.1022	AT2G34930.1	5.00E-35	disease resistance family protein
Cit.19606.1.S1_at	287.905	136.996	2.1016		NA	
Cit.13750.1.S1_at	704.1193	335.069	2.1014	AT4G17030.1	8.00E-88	ATEXLB1 (ARABIDOPSIS THALIANA EXPANSIN-LIKE B1)
Cit.5682.1.S1_s_at	672.5598	320.17	2.1006	AT5G12840.4	2.00E-37	NF-YA1 (NUCLEAR FACTOR Y, SUBUNIT A1); transcription factor
Cit.22221.1.S1_at	43.38485	20.6582	2.1001	AT3G07970.1	3.00E-55	QRT2 (QUARTET 2); polygalacturonase
Cit.27897.1.S1_at	87.80132	41.839	2.0986	AT1G29670.1	4.00E-38	GDSL-motif lipase/hydrolase family protein
Cit.21130.1.S1_s_at	517.6261	246.808	2.0973	AT3G28210.1	7.00E-67	PMZ; zinc ion binding
Cit.31848.1.S1_s_at	2860.066	1364.46	2.0961	AT2G23810.1	1.00E-116	TET8 (TETRASPANIN8)
Cit.28794.1.S1_at	46.18785	22.053	2.0944		NA	
Cit.24477.1.S1_s_at	1160.272	554.026	2.0943	AT5G26230.1	7.00E-74	unknown protein
Cit.25439.1.S1_at	66.06151	31.5743	2.0923	AT3G02050.1	1.00E-16	KUP3 (K+ UPTAKE TRANSPORTER 3); potassium ion transmembrane
Cit.6831.1.S1_at	61.76293	29.5487	2.0902	AT1G27940.1	1.00E-168	PGP13 (P-GLYCOPROTEIN 13); ATPase, coupled to transmembrane
Cit.32400.1.S1_at	46.9347	22.4776	2.0881		NA	

Cit.29884.1.S1_s_at	59.11395	28.3137	2.0878	AT1G63420.1	7.00E-51	INVOLVED IN: biological_process unknown; EXPRESSED IN: 22 plant
Cit.28253.1.S1_at	90.3321	43.2935	2.0865	AT3G26220.1	9.00E-54	CYP71B3; electron carrier/ heme binding / iron ion binding /
Cit.19193.1.S1_at	44.23011	21.2009	2.0862		NA	
Cit.20174.1.S1_x_at	144.3934	69.2173	2.0861		NA	
Cit.17629.1.S1_x_at	3377.638	1619.29	2.0859		NA	
Cit.20640.1.S1_at	71.51546	34.2941	2.0854	AT1G64380.1	2E-08	AP2 domain-containing transcription factor, putative
Cit.21853.1.S1_at	53.81849	25.809	2.0853	AT5G42830.1	7.00E-24	transferase family protein
Cit.16156.1.S1_at	1473.814	707.159	2.0841	AT5G41330.1	2.00E-53	potassium channel tetramerisation domain-containing protein
Cit.16112.1.S1_at	49.81073	23.9097	2.0833	AT3G22370.1	4.00E-18	AOX1A (ALTERNATIVE OXIDASE 1A); alternative oxidase
Cit.11226.1.S1_at	61.04298	29.3031	2.0832	AT3G26330.1	1.00E-150	CYP71B37; electron carrier/ heme binding / iron ion binding /
Cit.19647.1.S1_at	45.52184	21.8542	2.083	AT4G22910.1	4E-08	FZR2 (FIZZY-RELATED 2); signal transducer
Cit.2351.1.S1_at	187.7456	90.206	2.0813	AT2G38470.1	1.00E-124	WRKY33; transcription factor
Cit.3432.1.S1_at	506.4628	243.955	2.0761	AT2G20370.1	0	MUR3 (MURUS 3); catalytic/ transferase, transferring glycosyl groups
Cit.30026.1.S1_at	94.06519	45.3421	2.0746	AT3G48270.1	2.00E-41	CYP71A26; electron carrier/ heme binding / iron ion binding /
Cit.32508.1.S1_at	80.06468	38.6412	2.072		NA	
Cit.29379.1.S1_s_at	5745.2	2775.66	2.0698	AT2G27830.1	8.00E-49	FUNCTIONS IN: molecular_function unknown; EXPRESSED IN: 22 plant
Cit.35389.1.S1_at	1428.201	690.342	2.0688		NA	
Cit.4939.1.S1_at	46.8215	22.6324	2.0688		NA	
Cit.16165.1.S1_at	61.30169	29.6358	2.0685	AT1G77380.1	4.00E-95	AAP3; amino acid transmembrane transporter
Cit.6333.1.S1_at	885.8032	428.378	2.0678	AT4G17030.1	3.00E-83	ATEXLB1 (ARABIDOPSIS THALIANA EXPANSIN-LIKE B1)
Cit.19461.1.S1_s_at	1590.551	769.425	2.0672	AT3G44735.1	9.00E-12	PSK1; growth factor
Cit.24453.1.S1_at	57.90153	28.0187	2.0665	AT5G10690.1	2.00E-32	pentatricopeptide (PPR) repeat-containing protein / CBS domain-
Cit.5351.1.S1_x_at	244.5446	118.399	2.0654		NA	
Cit.2392.1.S1_at	213.051	103.208	2.0643	AT1G02800.1	0	ATCEL2; cellulase/ hydrolase, hydrolyzing O-glycosyl compounds
Cit.34961.1.S1_at	85.90496	41.6181	2.0641	AT4G21490.1	1.00E-47	NDB3; NADH dehydrogenase
Cit.13696.1.S1_at	62.14579	30.1086	2.0641	AT5G11810.1	9.00E-85	unknown protein
Cit.19678.1.S1_s_at	177.6713	86.0873	2.0638	AT2G27830.1	2.00E-48	FUNCTIONS IN: molecular_function unknown; EXPRESSED IN: 22 plant
Cit.30701.1.S1_s_at	646.2033	313.191	2.0633	AT4G39700.1	3.00E-60	heavy-metal-associated domain-containing protein / copper
Cit.7691.1.S1_at	69.19141	33.5346	2.0633		NA	
Cit.28249.1.S1_at	279.6013	135.595	2.062	AT1G13360.1	6.00E-22	unknown protein
Cit.57.1.S1_at	12426.08	6026.58	2.0619	AT1G17860.1	2.00E-27	trypsin and protease inhibitor family protein / Kunitz family protein
Cit.18509.1.S1_at	496.937	241.675	2.0562		NA	
Cit.31493.1.S1_at	104.9152	51.0777	2.054		NA	
Cit.28233.1.S1_at	153.2257	74.6361	2.053		NA	
Cit.19674.1.S1_s_at	1809.258	881.439	2.0526	AT3G45140.1	1.00E-109	LOX2 (LIPOXYGENASE 2); lipoxygenase

Cit.12954.1.S1_at	978.0884	476.973	2.0506	AT1G29670.1	1.00E-155	GDSL-motif lipase/hydrolase family protein
Cit.17307.1.S1_at	405.195	197.826	2.0482	AT4G14305.1	2.00E-72	FUNCTIONS IN: molecular_function unknown; INVOLVED IN:
Cit.3915.1.S1_s_at	99.87271	48.7816	2.0473	AT5G36110.1	1.00E-104	CYP716A1; electron carrier/ heme binding / iron ion binding /
Cit.4526.1.S1_s_at	56.58899	27.642	2.0472	AT4G34135.1	1.00E-131	UGT73B2 (UDP-GLUCOSYLTRANSFERASE 73B2); UDP-
Cit.1816.1.S1_s_at	169.3463	82.7722	2.0459	AT5G38410.1	4.00E-49	ribulose bisphosphate carboxylase small chain 3B / RuBisCO small
Cit.4456.1.S1_at	275.7358	134.834	2.045	AT1G65890.1	3.00E-73	AAE12 (ACYL ACTIVATING ENZYME 12); catalytic
Cit.21581.1.S1_x_at	50.03039	24.4871	2.0431		NA	
Cit.6084.1.S1_at	262.4242	128.491	2.0424	AT3G29360.2	0	UDP-glucose 6-dehydrogenase, putative
Cit.17552.1.S1_at	47.48224	23.286	2.0391	AT4G21380.1	1.00E-49	ARK3 (A. THALIANA RECEPTOR KINASE 3); kinase/ transmembrane
Cit.2725.1.S1_s_at	413.1652	202.647	2.0388	AT1G08630.4	1.00E-152	THA1 (Threonine Aldolase 1); aldehyde-lyase/ threonine aldolase
Cit.18624.1.S1_at	131.2948	64.4105	2.0384		NA	
Cit.25085.1.S1_at	56.15622	27.5825	2.0359	AT2G24640.1	7.00E-79	UBP19 (UBIQUITIN-SPECIFIC PROTEASE 19); cysteine-type
Cit.17589.1.S1_x_at	3802.582	1869.55	2.034	AT4G02380.1	8.00E-15	SAG21 (SENESCENCE-ASSOCIATED GENE 21)
Cit.28142.1.S1_s_at	113.0969	55.6189	2.0334	AT3G13730.1	8.00E-87	CYP90D1; oxidoreductase, acting on paired donors, with
Cit.4569.1.S1_at	269.3191	132.473	2.033	AT5G53750.1	1.00E-115	FUNCTIONS IN: molecular_function unknown; INVOLVED IN: response
Cit.18907.1.S1_at	53.82978	26.4794	2.0329		NA	
Cit.30467.1.S1_at	7677.531	3777.33	2.0325	AT5G39160.1	7.00E-74	germin-like protein (GLP2a) (GLP5a)
Cit.39988.1.S1_at	121.4895	59.797	2.0317	AT1G53860.1	1E-08	remorin family protein
Cit.4977.1.S1_s_at	722.8934	355.981	2.0307	AT4G31940.1	3.00E-77	CYP82C4; electron carrier/ heme binding / iron ion binding /
Cit.14406.1.S1_at	224.7478	110.733	2.0296	AT3G48660.1	4.00E-27	unknown protein
Cit.2305.1.S1_s_at	237.7715	117.195	2.0289	AT2G36090.1	3.00E-53	F-box family protein
Cit.14558.1.S1_at	1248.915	616.434	2.026	AT5G27320.1	1.00E-161	GID1C (GA INSENSITIVE DWARF1C); hydrolase
Cit.33162.1.S1_at	261.0252	128.874	2.0254	AT1G05280.1	9.00E-80	fringe-related protein
Cit.38606.1.S1_at	94.66123	46.7569	2.0245	AT3G29185.2	0.00001	unknown protein
Cit.5973.1.S1_s_at	72.40327	35.7898	2.023		NA	
Cit.13936.1.S1_s_at	102.9022	50.8672	2.023		NA	
Cit.591.1.S1_x_at	70.45567	34.8285	2.0229		NA	
Cit.2925.1.S1_x_at	735.3224	363.618	2.0222	AT2G46490.1	8.00E-18	unknown protein
Cit.31081.1.S1_at	47.16144	23.3312	2.0214		NA	
Cit.23834.1.S1_at	47.98662	23.7488	2.0206		NA	
Cit.18435.1.S1_at	331.4062	164.045	2.0202	AT4G08850.1	7.00E-18	kinase
Cit.28589.1.S1_x_at	89.02714	44.074	2.0199		NA	
Cit.5085.1.S1_at	4196.868	2079.05	2.0186	AT5G63350.1	8.00E-71	unknown protein
Cit.17368.1.S1_s_at	4633.202	2296.23	2.0177		NA	
Cit.26653.1.S1_at	226.2764	112.182	2.017	AT4G13880.1	7.00E-15	AtRLP48 (Receptor Like Protein 48); protein binding

Cit.30851.1.S1_at	101.4236	50.3026	2.0163	AT1G77380.1	1.00E-116	AAP3; amino acid transmembrane transporter
Cit.12943.1.S1_s_at	454.6278	225.513	2.016	AT5G14570.1	1.00E-179	ATNRT2.7 (Arabidopsis thaliana high affinity nitrate transporter 2.7);
Cit.12990.1.S1_at	1606.212	796.865	2.0157	AT5G63905.1	4.00E-30	unknown protein
Cit.9568.1.S1_s_at	6334.459	3146.75	2.013	AT5G64260.1	1.00E-125	EXL2 (EXORDIUM LIKE 2)
Cit.14919.1.S1_s_at	1490.432	740.577	2.0125	AT3G44735.1	5.00E-13	PSK1; growth factor
Cit.16228.1.S1_at	264.9024	131.872	2.0088		NA	
Cit.15606.1.S1_at	193.0199	96.1617	2.0072	AT1G80130.1	1.00E-56	Tetratricopeptide repeat (TPR)-like superfamily protein; FUNCTIONS
Cit.15696.1.S1_s_at	181.8178	90.5823	2.0072	AT3G05975.1	1.00E-35	FUNCTIONS IN: molecular_function unknown; INVOLVED IN:
Cit.12944.1.S1_s_at	140.4921	70.0094	2.0068	AT5G14570.1	1.00E-179	ATNRT2.7 (Arabidopsis thaliana high affinity nitrate transporter 2.7);
Cit.25038.1.S1_at	65.82707	32.8056	2.0066	AT2G01300.1	3.00E-29	unknown protein
Cit.13374.1.S1_s_at	1200.296	598.35	2.006	AT1G26800.1	2.00E-49	zinc finger (C3HC4-type RING finger) family protein
Cit.37965.1.S1_at	78.99478	39.385	2.0057	AT4G17250.1	2.00E-40	unknown protein
Cit.21713.1.S1_x_at	142.8473	71.2299	2.0054		NA	
Cit.7749.1.S1_at	243.2385	121.334	2.0047	AT1G78070.1	1.00E-68	FUNCTIONS IN: molecular_function unknown; INVOLVED IN:
Cit.17554.1.S1_at	144.388	72.0479	2.0041		NA	
Cit.29267.1.S1_s_at	199.1847	99.4049	2.0038	AT3G44735.1	2.00E-11	PSK1; growth factor
Cit.11486.1.S1_at	43.19473	21.5746	2.0021		NA	
Cit.9701.1.S1_s_at	3226.084	1611.8	2.0015	AT5G42050.1	1.00E-106	FUNCTIONS IN: molecular_function unknown; EXPRESSED IN: 22 plant
Cit.28969.1.S1_at	46.14266	23.0558	2.0013		NA	
Cit.27327.1.S1_at	48.07668	24.0252	2.0011		NA	
Cit.30139.1.S1_at	326.3217	652.648	-2	AT5G53870.1	2.00E-19	plastocyanin-like domain-containing protein
Cit.7504.1.S1_at	217.2248	434.664	-2.001	AT2G39470.1	1.00E-69	PPL2 (PsbP-like protein 2); calcium ion binding
Cit.18267.1.S1_at	41.97015	84.0169	-2.002		NA	
Cit.30981.1.S1_s_at	26.14364	52.3875	-2.004	AT1G19870.1	3.00E-35	iqd32 (IQ-domain 32); calmodulin binding
Cit.1870.1.S1_s_at	127.9081	256.452	-2.005	AT2G17880.1	3.00E-24	DNAJ heat shock protein, putative
Cit.19375.1.S1_s_at	75.74789	152.077	-2.008	AT2G01570.1	4.00E-26	RGA1 (REPRESSOR OF GA1-3 1); protein binding / transcription factor
Cit.1293.1.S1_at	32.28797	64.8623	-2.009	AT5G59840.1	6.00E-22	Ras-related GTP-binding family protein
Cit.27996.1.S1_at	25.22699	50.7096	-2.01	AT2G29410.1	0.00006	MTPB1 (METAL TOLERANCE PROTEIN B1); efflux transmembrane
Cit.23747.1.S1_at	33.85958	68.1506	-2.013		NA	
Cit.26738.1.S1_at	20.16338	40.5864	-2.013	AT5G66550.1	6.00E-17	Maf family protein
Cit.28591.1.S1_at	137.3847	276.601	-2.013	AT1G75500.1	2.00E-40	nodulin MtN21 family protein
Cit.19161.1.S1_at	25.29954	50.9821	-2.015	AT4G19040.2	8.00E-59	EDR2; lipid binding
Cit.35443.1.S1_at	41.42934	83.4879	-2.015	AT3G48380.2	5.00E-62	FUNCTIONS IN: molecular_function unknown; INVOLVED IN:
Cit.21518.1.S1_at	79.59566	160.755	-2.02		NA	
Cit.28560.1.S1_at	66.92474	135.178	-2.02	AT3G14860.2	1.00E-19	NHL repeat-containing protein

Cit.28885.1.S1_at	91.67629	185.238	-2.021		NA	
Cit.6888.1.S1_at	92.77523	187.567	-2.022	AT2G18280.2	3.00E-60	AtTLP2 (TUBBY LIKE PROTEIN 2); phosphoric diester hydrolase/
Cit.18861.1.S1_x_at	257.9918	522.263	-2.024		NA	
Cit.17047.1.S1_at	45.53867	92.1885	-2.024		NA	
Cit.16546.1.S1_at	23.8807	48.354	-2.025	AT1G76405.2	9.00E-34	unknown protein
Cit.31079.1.S1_at	135.4827	274.428	-2.026	AT4G37925.1	9.00E-41	NDH-M (subunit NDH-M of NAD(P)H:plastoquinone dehydrogenase
Cit.34573.1.S1_at	22.31065	45.2307	-2.027		NA	
Cit.34934.1.S1_at	111.1719	225.447	-2.028		NA	
Cit.32855.1.S1_at	29.1706	59.212	-2.03	AT4G26330.1	6.00E-81	UNE17 (UNFERTILIZED EMBRYO SAC 17); identical protein binding /
Cit.22278.1.S1_s_at	1694.237	3446.86	-2.034	AT1G75500.1	1.00E-165	nodulin MtN21 family protein
Cit.32460.1.S1_at	198.488	404.014	-2.035	AT3G17360.1	0.00004	POK1 (PHRAGMOPLAST ORIENTING KINESIN 1); ATP binding /
Cit.6513.1.S1_at	577.9684	1178.1	-2.038	AT1G20610.1	4.00E-94	CYCB2;3 (Cyclin B2;3); cyclin-dependent protein kinase regulator
Cit.23187.1.S1_x_at	24.44999	49.9097	-2.041		NA	
Cit.30658.1.S1_at	34.29506	70.0275	-2.042		NA	
Cit.10012.1.S1_at	28.41219	58.0418	-2.043		NA	
Cit.4555.1.S1_at	277.1714	566.618	-2.044	AT5G13050.1	2.00E-91	5-FCL (5-FORMYLTETRAHYDROFOLATE CYCLOLIGASE); 5-
Cit.31058.1.S1_at	24.3575	49.7958	-2.044		NA	
Cit.12875.1.S1_at	34.07408	69.7072	-2.046	AT1G20810.1	1.00E-78	immunophilin / FKBP-type peptidyl-prolyl cis-trans isomerase family
Cit.30868.1.S1_s_at	161.4142	330.438	-2.047	AT3G46780.1	6.00E-80	PTAC16 (PLASTID TRANSCRIPTIONALLY ACTIVE 16); binding / catalytic
Cit.20267.1.S1_x_at	754.4675	1551.06	-2.056	AT5G48490.1	3.00E-18	protease inhibitor/seed storage/lipid transfer protein (LTP) family
Cit.12700.1.S1_x_at	226.3701	465.878	-2.058	AT1G20030.1	1.00E-65	pathogenesis-related thaumatin family protein
Cit.20252.1.S1_s_at	913.4503	1882.21	-2.061	AT1G31335.1	3.00E-19	unknown protein
Cit.20567.1.S1_at	544.8947	1124.36	-2.063	AT2G36985.1	1.00E-15	ROT4 (ROTUNDIFOLIA4)
Cit.7647.1.S1_at	449.3389	927.203	-2.063	AT3G57040.1	4.00E-72	ARR9 (RESPONSE REGULATOR 9); transcription regulator/ two-
Cit.16756.1.S1_s_at	184.2558	380.36	-2.064	AT3G45140.1	0	LOX2 (LIPOXYGENASE 2); lipoxygenase
Cit.6145.1.S1_at	112.2461	231.761	-2.065	AT1G32100.1	1.00E-113	PRR1 (PINORESINOL REDUCTASE 1); pinoresinol reductase
Cit.13168.1.S1_at	33.91531	70.1578	-2.069	AT1G78700.1	1.00E-109	brassinosteroid signalling positive regulator-related
Cit.37818.1.S1_at	24.6621	51.0817	-2.071	AT4G18590.1	2.00E-22	FUNCTIONS IN: molecular_function unknown; INVOLVED IN:
Cit.542.1.S1_at	56.2399	116.589	-2.073		NA	
Cit.20232.1.S1_s_at	78.91686	163.712	-2.074	AT4G35750.1	6.00E-77	Rho-GTPase-activating protein-related
Cit.1871.1.S1_at	130.3678	270.968	-2.078	AT4G36040.1	1.00E-24	DNAJ heat shock N-terminal domain-containing protein (J11)
Cit.5392.1.S1_s_at	294.9988	613.307	-2.079	AT1G03600.1	9.00E-53	photosystem II family protein
Cit.6178.1.S1_at	106.9042	222.366	-2.08	AT4G09890.1	1.00E-20	unknown protein
Cit.30185.1.S1_at	41.38433	86.1895	-2.083	AT1G60140.1	6.00E-52	ATTPS10 (trehalose phosphate synthase); transferase, transferring
Cit.6443.1.S1_at	28.96346	60.4859	-2.088	AT1G65900.1	1.00E-149	unknown protein

Cit.8515.1.S1_s_at	79.27435	165.616	-2.089	AT5G06720.1	1.00E-116	peroxidase, putative
Cit.1842.1.S1_at	310.8216	649.771	-2.09	AT2G20260.1	6.00E-41	PSAE-2 (photosystem I subunit E-2); catalytic
Cit.35924.1.S1_at	30.39279	63.5639	-2.091	AT5G13960.1	1.00E-22	SUVH4 (SU(VAR)3-9 HOMOLOG 4); double-stranded methylated DNA
Cit.27079.1.S1_at	41.49279	86.8122	-2.092	AT2G40490.1	2.00E-33	HEME2; uroporphyrinogen decarboxylase
Cit.14975.1.S1_at	148.0278	309.781	-2.093	AT3G17030.1	1.00E-141	INVOLVED IN: biological_process unknown; LOCATED IN:
Cit.18669.1.S1_at	25.32609	53.0436	-2.094	AT1G70830.5	0.000003	MLP28 (MLP-LIKE PROTEIN 28)
Cit.11401.1.S1_at	622.4598	1304.44	-2.096		NA	
Cit.20546.1.S1_at	54.75388	115.033	-2.101	AT3G01670.1	1.00E-10	unknown protein
Cit.24179.1.S1_at	89.93012	188.986	-2.101	AT3G18850.5	3.00E-81	LPAT5; acyltransferase
Cit.28947.1.S1_at	166.0376	349.449	-2.105	AT5G64410.1	1.00E-100	OPT4 (OLIGOPEPTIDE TRANSPORTER 4); oligopeptide transporter
Cit.7430.1.S1_at	29.12128	61.3399	-2.106	AT4G16380.2	1.00E-15	LOCATED IN: cellular_component unknown; BEST Arabidopsis thaliana
Cit.25177.1.S1_s_at	79.8278	168.185	-2.107	AT4G09890.1	1.00E-20	unknown protein
Cit.31051.1.S1_at	27.9287	59.0327	-2.114	AT1G18670.1	0	IBS1 (IMPAIRED IN BABA-INDUCED STERILITY 1); ATP binding / kinase/
Cit.28940.1.S1_x_at	50.34005	106.518	-2.116	AT1G01300.1	3.00E-57	aspartyl protease family protein
Cit.21905.1.S1_s_at	374.5699	793.6	-2.119		NA	
Cit.37306.1.S1_at	104.7947	222.179	-2.12	AT4G22250.1	2.00E-10	zinc finger (C3HC4-type RING finger) family protein
Cit.29798.1.S1_at	45.23938	96.2322	-2.127	AT3G54670.1	1.00E-118	TTN8 (TITAN8); ATP binding / transporter
Cit.7247.1.S1_at	128.2527	272.833	-2.127	AT1G75950.1	9.00E-15	SKP1 (S PHASE KINASE-ASSOCIATED PROTEIN 1); protein binding /
Cit.16134.1.S1_at	637.5215	1356.26	-2.127		NA	
Cit.15468.1.S1_at	86.5437	184.177	-2.128	AT4G38070.1	6.00E-22	bHLH family protein
Cit.6202.1.S1_at	148.04	315.107	-2.129		NA	
Cit.6780.1.S1_at	256.8712	547.112	-2.13	AT3G63088.1	2E-08	RTFL14 (ROTUNDIFOLIA LIKE 14)
Cit.23941.1.S1_at	79.40873	169.146	-2.13	AT2G34410.2	5.00E-11	O-acetyltransferase family protein
Cit.15810.1.S1_s_at	654.4623	1394.07	-2.13	AT3G02120.1	5.00E-19	hydroxyproline-rich glycoprotein family protein
Cit.12755.1.S1_at	22.30264	47.6484	-2.136	AT5G08580.1	1.00E-153	calcium-binding EF hand family protein
Cit.39003.1.S1_s_at	40.00766	85.6249	-2.14	AT1G19150.1	1.00E-116	LHCA6; chlorophyll binding
Cit.22614.1.S1_at	101.4409	217.223	-2.141	AT1G66150.1	2.00E-31	TMK1 (TRANSMEMBRANE KINASE 1); transmembrane receptor
Cit.21403.1.S1_at	35.92535	77.0577	-2.145		NA	
Cit.8522.1.S1_at	78.61464	168.641	-2.145	AT5G08350.1	2.00E-45	GRAM domain-containing protein / ABA-responsive protein-related
Cit.6681.1.S1_s_at	128.1391	275.039	-2.146	AT4G14490.1	3.00E-42	forkhead-associated domain-containing protein / FHA domain-
Cit.24383.1.S1_x_at	128.4243	275.697	-2.147	AT3G55430.1	5.00E-28	glycosyl hydrolase family 17 protein / beta-1,3-glucanase, putative
Cit.18604.1.S1_at	50.65016	109.093	-2.154	AT4G06536.1	2.00E-15	FUNCTIONS IN: molecular_function unknown; INVOLVED IN:
Cit.23470.1.S1_s_at	82.7823	178.642	-2.158	AT3G01670.1	5.00E-51	unknown protein
Cit.34743.1.S1_s_at	35.75128	77.1855	-2.159		NA	
Cit.22234.1.S1_s_at	2244.373	4851.26	-2.162	AT5G20950.2	0	glycosyl hydrolase family 3 protein

Cit.1843.1.S1_at	97.36727	210.664	-2.164		NA	
Cit.9990.1.S1_x_at	835.392	1813.29	-2.171	AT1G33140.1	4.00E-89	PGY2 (PIGGYBACK2); structural constituent of ribosome
Cit.28417.1.S1_s_at	199.3788	433.942	-2.176	AT3G06880.1	1.00E-10	nucleotide binding
Cit.29571.1.S1_at	25.03313	54.5674	-2.18	AT1G02850.2	1.00E-115	BGLU11 (BETA GLUCOSIDASE 11); hydrolase, hydrolyzing O-glycosyl
Cit.26584.1.S1_at	33.08693	72.1637	-2.181	AT5G16310.1	2.00E-14	UCH1; ubiquitin thiolesterase
Cit.6393.1.S1_at	32.16141	70.1819	-2.182	AT4G28030.1	6.00E-86	GCN5-related N-acetyltransferase (GNAT) family protein
Cit.13501.1.S1_at	1114.493	2432.99	-2.183	AT1G78890.1	1.00E-37	unknown protein
Cit.22067.1.S1_at	48.81923	107.2	-2.196	AT3G58110.1	4.00E-46	unknown protein
Cit.15007.1.S1_at	103.4133	227.457	-2.199	AT1G51400.1	2.00E-16	photosystem II 5 kD protein
Cit.7003.1.S1_at	485.913	1070.46	-2.203	AT5G17160.1	0.00007	unknown protein
Cit.4552.1.S1_at	27.96565	61.6283	-2.204	AT2G27880.1	1.00E-119	AGO5 (ARGONAUTE 5); nucleic acid binding
Cit.28316.1.S1_s_at	1297.118	2861.52	-2.206		NA	
Cit.20232.1.S1_at	30.304	67.0632	-2.213	AT4G35750.1	4.00E-24	Rho-GTPase-activating protein-related
Cit.27775.1.S1_x_at	35.45546	78.566	-2.216	AT3G42170.1	3.00E-10	DNA binding
Cit.18458.1.S1_at	66.31771	147.052	-2.217		NA	
Cit.7921.1.S1_at	34.5976	76.7982	-2.22		NA	
Cit.19333.1.S1_s_at	118.4175	263.276	-2.223	AT3G48200.1	9.00E-64	unknown protein
Cit.16594.1.S1_at	93.74113	208.813	-2.228	AT4G39640.2	1.00E-143	GGT1 (GAMMA-GLUTAMYL TRANSPEPTIDASE 1); gamma-
Cit.15749.1.S1_at	28.1572	62.8946	-2.234		NA	
Cit.34097.1.S1_at	24.86823	55.5565	-2.234	AT5G38260.1	1.00E-53	serine/threonine protein kinase, putative
Cit.25071.1.S1_at	22.8288	51.0263	-2.235	AT3G07020.1	7.00E-66	UDP-glucose:sterol glucosyltransferase (UGT80A2)
Cit.15018.1.S1_at	209.3634	469.081	-2.241	AT4G10270.1	2.00E-22	wound-responsive family protein
Cit.14612.1.S1_s_at	29.42759	66.0365	-2.244	AT1G67050.1	2.00E-54	unknown protein
Cit.20090.1.S1_at	23.71665	53.2689	-2.246		NA	
Cit.3768.1.S1_at	23.28448	52.345	-2.248		NA	
Cit.5112.1.S1_at	34.87507	78.5667	-2.253	AT1G47128.1	1.00E-118	RD21 (responsive to dehydration 21); cysteine-type endopeptidase/
Cit.21939.1.S1_at	71.46506	162.082	-2.268	AT5G20660.1	0.0000003	24 kDa vacuolar protein, putative
Cit.38764.1.S1_at	78.10275	177.621	-2.274	AT2G43820.1	4.00E-51	UGT74F2 (UDP-GLUCOSYLTRANSFERASE 74F2); UDP-glucose:4-
Cit.13270.1.S1_x_at	21.73117	49.5125	-2.278	AT5G58380.1	1.00E-39	SIP1 (SOS3-INTERACTING PROTEIN 1); ATP binding / kinase/ protein
Cit.6105.1.S1_at	32.30966	73.6482	-2.279	AT1G04945.2	1E-09	FUNCTIONS IN: molecular_function unknown; INVOLVED IN:
Cit.13849.1.S1_at	25.89842	59.0868	-2.281	AT3G04910.3	1.00E-12	WNK1 (WITH NO LYSINE (K) 1); kinase/ protein kinase/ protein
Cit.6780.1.S1_s_at	384.3886	878.933	-2.287	AT3G63088.1	2E-08	RTFL14 (ROTUNDIFOLIA LIKE 14)
Cit.4286.1.S1_at	42.32218	97.0373	-2.293	AT1G60550.1	8.00E-74	ECHID (ENOYL-COA HYDRATASE/ISOMERASE D); catalytic/ naphthoate
Cit.8463.1.S1_at	317.5776	729.213	-2.296	AT2G38870.1	1.00E-17	protease inhibitor, putative
Cit.9606.1.S1_x_at	351.586	808.811	-2.3	AT1G53280.1	1.00E-165	DJ-1 family protein

Cit.16367.1.S1_at	37.01232	85.2215	-2.303		NA	
Cit.30924.1.S1_at	155.6327	358.506	-2.304	AT3G06880.1	1.00E-10	nucleotide binding
Cit.31118.1.S1_at	121.7964	280.753	-2.305	AT1G03495.1	1.00E-21	transferase/ transferase, transferring acyl groups other than amino-
Cit.8839.1.S1_x_at	28.92242	66.68	-2.305		NA	
Cit.8685.1.S1_at	20.9327	48.3587	-2.31	AT5G03300.1	1.00E-170	ADK2 (ADENOSINE KINASE 2); adenosine kinase/ copper ion binding /
Cit.37449.1.S1_at	123.7554	286.472	-2.315	AT5G67460.1	1.00E-19	glycosyl hydrolase family protein 17
Cit.31281.1.S1_at	27.0231	62.5718	-2.315	AT5G06580.1	1.00E-69	FAD linked oxidase family protein
Cit.16542.1.S1_at	42.592	98.625	-2.316	AT1G13220.2	1.00E-110	LINC2 (LITTLE NUCLEI2)
Cit.5057.1.S1_at	33.7512	78.2732	-2.319	AT1G19150.1	1.00E-114	LHCA6; chlorophyll binding
Cit.27408.1.S1_at	41.26555	95.7728	-2.321		NA	
Cit.7728.1.S1_at	178.772	414.959	-2.321	AT3G62980.1	0.0000003	TIR1 (TRANSPORT INHIBITOR RESPONSE 1); auxin binding / protein
Cit.39050.1.S1_at	33.80975	78.5305	-2.323	AT3G10200.1	4.00E-81	dehydration-responsive protein-related
Cit.18037.1.S1_at	176.1273	409.441	-2.325	AT3G61510.1	0	ACS1 (ACC SYNTHASE 1); 1-aminocyclopropane-1-carboxylate
Cit.8578.1.S1_at	155.6656	362.627	-2.33	AT1G67090.1	2.00E-32	RBCS1A (RIBULOSE BISPHOSPHATE CARBOXYLASE SMALL CHAIN 1A);
Cit.14911.1.S1_at	717.9753	1672.67	-2.33	AT1G52720.1	6.00E-22	unknown protein
Cit.5064.1.S1_at	102.5187	239.687	-2.338	AT3G20395.1	4.00E-42	protein binding / zinc ion binding
Cit.30192.1.S1_at	43.22561	101.173	-2.341	AT1G21510.1	1.00E-24	unknown protein
Cit.38029.1.S1_at	28.46377	66.69	-2.343	AT1G20870.1	1.00E-12	LOCATED IN: cellular_component unknown; EXPRESSED IN: 15 plant
Cit.20592.1.S1_x_at	466.9171	1100.26	-2.356		NA	
Cit.12450.1.S1_at	224.2964	530.407	-2.365		NA	
Cit.233.1.S1_at	395.8207	939.047	-2.372		NA	
Cit.26229.1.S1_at	21.91126	52.0916	-2.377		NA	
Cit.8403.1.S1_s_at	806.9077	1921.76	-2.382	AT4G27450.1	1.00E-122	unknown protein
Cit.24643.1.S1_s_at	160.4521	383.173	-2.388	AT3G09980.1	2.00E-51	unknown protein
Cit.30506.1.S1_s_at	750.5219	1792.71	-2.389	AT5G01600.1	3.00E-92	ATFER1; ferric iron binding / iron ion binding
Cit.6471.1.S1_at	138.1765	330.564	-2.392		NA	
Cit.22018.1.S1_at	20.09613	48.2409	-2.401		NA	
Cit.26904.1.S1_at	240.828	580.269	-2.409		NA	
Cit.18242.1.S1_at	173.2895	418.429	-2.415		NA	
Cit.21552.1.S1_at	198.2348	480.12	-2.422	AT1G31335.1	3.00E-19	unknown protein
Cit.16639.1.S1_at	133.643	323.726	-2.422	AT1G49870.2	5.00E-17	EXPRESSED IN: 10 plant structures; EXPRESSED DURING: F mature
Cit.36686.1.S1_at	29.48994	72.0894	-2.445	AT5G61580.2	6.00E-93	PFK4 (PHOSPHOFRUCTOKINASE 4); 6-phosphofructokinase
Cit.14638.1.S1_at	20.17821	49.5468	-2.455		NA	
Cit.20280.1.S1_at	179.5089	442.301	-2.464		NA	
Cit.21680.1.S1_at	332.6417	824.071	-2.477	AT1G04030.1	3E-09	unknown protein

Cit.4211.1.S1_at	52.43237	130.397	-2.487	AT1G02640.1	0	BXL2 (BETA-XYLOSIDASE 2); hydrolase, hydrolyzing O-glycosyl
Cit.24701.1.S1_s_at	81.23471	202.221	-2.489		NA	
Cit.29930.1.S1_at	32.02058	79.83	-2.493	AT3G09070.1	4.00E-42	glycine-rich protein
Cit.448.1.S1_at	23.53094	59.0516	-2.51	AT1G23820.1	1.00E-147	SPDS1 (spermidine synthase 1); spermidine synthase
Cit.22486.1.S1_x_at	36.99685	93.3868	-2.524	AT4G16440.1	1.00E-25	ferredoxin hydrogenase
Cit.30007.1.S1_at	58.17092	148.29	-2.549	AT3G48760.1	2.00E-37	zinc finger (DHHC type) family protein
Cit.11346.1.S1_at	184.6319	470.999	-2.551	AT5G17920.2	6.00E-73	ATMS1; 5-methyltetrahydropteroyltriglutamate-homocysteine S-
Cit.13706.1.S1_at	336.8359	860.943	-2.556	AT4G27450.1	4.00E-71	unknown protein
Cit.40290.1.S1_at	21.79027	55.7038	-2.556	AT1G60460.3	5.00E-40	unknown protein
Cit.32783.1.S1_at	42.77652	109.775	-2.566	AT1G31817.1	0.00007	NFD3 (NUCLEAR FUSION DEFECTIVE 3); structural constituent of
Cit.6073.1.S1_s_at	129.2748	331.853	-2.567	AT5G47000.1	3.00E-91	peroxidase, putative
Cit.7299.1.S1_s_at	2198.022	5675.87	-2.582	AT4G22505.1	2.00E-27	INVOLVED IN: lipid transport; CONTAINS InterPro DOMAIN/s:
Cit.13500.1.S1_at	24.63495	63.7329	-2.587	AT4G02590.2	1.00E-110	UNE12 (unfertilized embryo sac 12); DNA binding / transcription
Cit.26718.1.S1_at	22.70693	58.977	-2.597	AT1G56070.1	3.00E-58	LOS1; copper ion binding / translation elongation factor/ translation
Cit.4159.1.S1_s_at	76.76971	200.582	-2.613	AT1G74890.1	1.00E-55	ARR15 (RESPONSE REGULATOR 15); transcription regulator/ two-
Cit.25025.1.S1_s_at	24.92052	66.776	-2.68	AT1G73370.1	1.00E-116	SUS6 (SUCROSE SYNTHASE 6); UDP-glycosyltransferase/ sucrose
Cit.26276.1.S1_at	27.45868	73.6899	-2.684	AT4G21200.1	2.00E-22	GA2OX8 (GIBBERELLIN 2-OXIDASE 8); gibberellin 2-beta-dioxygenase
Cit.21007.1.S1_at	20.91324	56.2894	-2.692		NA	
Cit.8902.1.S1_at	43.72465	120.75	-2.762	AT1G23740.1	1.00E-124	oxidoreductase, zinc-binding dehydrogenase family protein
Cit.15375.1.S1_at	29.37022	81.1865	-2.764		NA	
Cit.8519.1.S1_x_at	148.5403	412.414	-2.776	AT2G38380.1	1.00E-109	peroxidase 22 (PER22) (P22) (PRXEA) / basic peroxidase E
Cit.16203.1.S1_at	70.38966	195.497	-2.777	AT4G39710.1	2.00E-67	immunophilin, putative / FKBP-type peptidyl-prolyl cis-trans
Cit.19378.1.S1_s_at	322.5768	899.552	-2.789	AT2G02850.1	4.00E-37	ARPN (PLANTACYANIN); copper ion binding / electron carrier
Cit.13706.1.S1_s_at	1296.45	3633.49	-2.803	AT4G27450.1	1.00E-100	unknown protein
Cit.10697.1.S1_s_at	802.1516	2251.77	-2.807	AT5G20820.1	5.00E-30	auxin-responsive protein-related
Cit.8902.1.S1_s_at	50.36788	141.736	-2.814	AT1G23740.1	1.00E-124	oxidoreductase, zinc-binding dehydrogenase family protein
Cit.9009.1.S1_at	25.04011	71.2822	-2.847	AT2G43970.1	8.00E-80	La domain-containing protein
Cit.20400.1.S1_s_at	122.1608	348.154	-2.85	AT5G61440.1	9.00E-60	ACHT5 (ATYPICAL CYS HIS RICH THIOREDOXIN 5)
Cit.18006.1.S1_at	304.6966	868.721	-2.851	AT4G27450.1	1.00E-100	unknown protein
Cit.20210.1.S1_at	33.03134	94.2754	-2.854		NA	
Cit.29982.1.S1_at	91.47431	262.093	-2.865	AT3G11340.1	5.00E-74	UDP-glucoronosyl/UDP-glucosyl transferase family protein
Cit.39331.1.S1_at	64.83852	186.722	-2.88		NA	
Cit.18687.1.S1_at	35.31792	102.104	-2.891	AT1G05680.1	3.00E-35	UDP-glucoronosyl/UDP-glucosyl transferase family protein
Cit.7227.1.S1_at	41.89216	121.42	-2.898	AT2G42680.1	3.00E-15	MBF1A (MULTIPROTEIN BRIDGING FACTOR 1A); DNA binding /
Cit.11911.1.S1_at	21.80593	63.4444	-2.91	AT1G65450.1	1.00E-100	transferase family protein

Cit.15664.1.S1_at	40.42979	118.159	-2.923	AT3G11980.1	2.00E-95	MS2 (MALE STERILITY 2); fatty acyl-CoA reductase (alcohol-forming)/
Cit.19325.1.S1_at	373.6801	1149.69	-3.077		NA	
Cit.20185.1.S1_x_at	26.46508	81.8551	-3.093		NA	
Cit.24348.1.S1_at	82.2046	254.343	-3.094		NA	
Cit.34811.1.S1_at	60.09723	189.442	-3.152	AT4G34950.1	3.00E-41	nodulin family protein
Cit.13836.1.S1_at	88.52342	283.473	-3.202	AT5G19120.1	1.00E-73	aspartic-type endopeptidase
Cit.27555.1.S1_at	65.64743	211.077	-3.215	AT5G56970.1	1.00E-58	CKX3 (CYTOKININ OXIDASE 3); amine oxidase/ cytokinin
Cit.24063.1.S1_at	25.54434	82.3177	-3.223	AT3G22440.1	3.00E-18	hydroxyproline-rich glycoprotein family protein
Cit.6665.1.S1_at	29.72401	96.691	-3.253	AT3G10360.1	2.00E-64	APUM4 (Arabidopsis Pumilio 4); RNA binding / binding
Cit.541.1.S1_at	79.67655	262.091	-3.289		NA	
Cit.14365.1.S1_at	69.22871	229.01	-3.308		NA	
Cit.15637.1.S1_at	52.14392	172.615	-3.31	AT4G28780.1	9.00E-91	GDSL-motif lipase/hydrolase family protein
Cit.29760.1.S1_at	27.69178	93.2094	-3.366		NA	
Cit.4667.1.S1_at	96.01096	325.576	-3.391	AT3G11980.1	8.00E-23	MS2 (MALE STERILITY 2); fatty acyl-CoA reductase (alcohol-forming)/
Cit.26968.1.S1_at	25.53718	87.3679	-3.421	AT4G39900.1	8.00E-30	unknown protein
Cit.7688.1.S1_at	105.6554	370.534	-3.507	AT5G40030.1	5.00E-77	protein kinase, putative
Cit.15006.1.S1_at	66.77699	253.276	-3.793	AT4G38540.1	2.00E-81	monooxygenase, putative (MO2)
Cit.15073.1.S1_at	87.49518	339.148	-3.876	AT2G03200.1	6.00E-48	aspartyl protease family protein
Cit.20445.1.S1_at	23.69058	103.177	-4.355		NA	
Cit.22753.1.S1_x_at	199.123	925.567	-4.648		NA	
Cit.13787.1.S1_s_at	111.4458	558.25	-5.009	AT2G03200.1	1.00E-50	aspartyl protease family protein
Cit.8501.1.S1_at	204.2397	1102.81	-5.4		NA	

Supplementary Table 2. PDTA in seedless vs. seedy Fallglo fruits at time point 2.

ProbeSet ID	Fallglo_ Seedless	Fallglo_S eedy	Ratio	AtGID	E-Score	Arabidopsis.annotation
Cit.10894.1.S1_s_at	3227.242	37.4137	86.25825	AT5G06760.1	1.00E-48	late embryogenesis abundant group 1 domain-containing protein / l
Cit.29507.1.S1_s_at	1832.219	77.3031	23.70176		NA	
Cit.5970.1.S1_at	508.4922	28.0815	18.10776	AT5G61430.1	6.00E-92	ANAC100 (ARABIDOPSIS NAC DOMAIN CONTAINING PROTEIN 100);
Cit.21810.1.S1_x_at	291.013	24.7262	11.76942		NA	
Cit.8027.1.S1_s_at	1020.072	89.7158	11.37004	AT3G12580.1	0	HSP70 (heat shock protein 70); ATP binding
Cit.17243.1.S1_at	367.3534	40.8085	9.001878	AT1G32920.1	3.00E-07	unknown protein
Cit.21717.1.S1_at	401.3795	45.5685	8.808269	AT3G04720.1	2.00E-43	PR4 (PATHOGENESIS-RELATED 4); chitin binding
Cit.185.1.S1_s_at	1904.139	238.714	7.976661	AT4G10250.1	4.00E-71	ATHSP22.0
Cit.13586.1.S1_at	317.5426	40.1473	7.909431	AT3G49940.1	2.00E-52	LBD38 (LOB DOMAIN-CONTAINING PROTEIN 38)
Cit.34286.1.S1_at	175.3945	22.5569	7.775661		NA	
Cit.13915.1.S1_at	354.5393	45.8447	7.733485	AT3G14440.1	0	NCED3 (NINE-CIS-EPOXYCAROTENOID DIOXYGENASE 3); 9-cis-epoxy
Cit.13693.1.S1_s_at	254.8874	36.1132	7.058004	AT4G38960.1	6.00E-28	zinc finger (B-box type) family protein
Cit.29547.1.S1_at	447.0851	64.391	6.943284	AT4G18380.1	6.00E-46	F-box family protein
Cit.1496.1.S1_s_at	201.2682	30.0705	6.693207	AT3G04070.1	1.00E-98	anac047 (Arabidopsis NAC domain containing protein 47); transcript
Cit.2739.1.S1_at	479.1454	73.7793	6.494311		NA	
Cit.13694.1.S1_at	498.5662	76.7817	6.493293	AT4G38960.1	6.00E-28	zinc finger (B-box type) family protein
Cit.12810.1.S1_at	213.892	37.163	5.755506	AT1G19670.1	5.00E-65	ATCLH1 (ARABIDOPSIS THALIANA CORONATINE-INDUCED PROTEIN
Cit.13558.1.S1_s_at	904.5673	164.059	5.513664	AT1G16030.1	1.00E-111	Hsp70b (heat shock protein 70B); ATP binding
Cit.3778.1.S1_at	713.7197	132.522	5.385686	AT1G19210.1	1.00E-45	AP2 domain-containing transcription factor, putative
Cit.20412.1.S1_s_at	159.6204	30.2357	5.279201	AT3G12500.1	1.00E-127	ATHCHIB (ARABIDOPSIS THALIANA BASIC CHITINASE); chitinase
Cit.31451.1.S1_s_at	146.5143	27.9957	5.233459	AT1G11530.1	5.00E-37	ATCXXS1 (C-terminal cysteine residue is changed to a serine 1); prot
Cit.5516.1.S1_at	323.921	63.2047	5.124952	AT3G10020.1	4.00E-39	unknown protein
Cit.21497.1.S1_at	231.9361	45.6276	5.083238	AT2G38905.1	5.00E-10	hydrophobic protein, putative / low temperature and salt responsiv
Cit.302.1.S1_s_at	99.58868	20.0176	4.975061	AT3G12500.1	1.00E-127	ATHCHIB (ARABIDOPSIS THALIANA BASIC CHITINASE); chitinase
Cit.2906.1.S1_s_at	115.9998	23.4651	4.943508		NA	
Cit.17450.1.S1_at	198.9463	40.9249	4.861259	AT2G46330.1	9.00E-09	AGP16 (ARABINOGALACTAN PROTEIN 16)
Cit.37764.1.S1_s_at	242.0168	50.1554	4.825343	AT1G27730.1	7.00E-57	STZ (salt tolerance zinc finger); nucleic acid binding / transcription fa
Cit.5377.1.S1_at	282.3131	59.7785	4.722653	AT4G37370.1	1.00E-156	CYP81D8; electron carrier/ heme binding / iron ion binding / monoc
Cit.17294.1.S1_at	342.7165	73.7328	4.648089		NA	
Cit.2928.1.S1_s_at	101.3698	21.8837	4.632216	AT5G23810.1	1.00E-103	AAP7; amino acid transmembrane transporter
Cit.4671.1.S1_at	2080.489	455.263	4.569858	AT1G12060.1	6.00E-17	ATBAG5 (ARABIDOPSIS THALIANA BCL-2-ASSOCIATED ATHANOGENE

Cit.31296.1.S1_at	358.8793	78.5576	4.568361		NA	
Cit.2927.1.S1_s_at	554.5132	122.852	4.513654	AT5G23810.1	2.00E-96	AAP7; amino acid transmembrane transporter
Cit.10594.1.S1_at	618.7908	142.027	4.356859	AT3G54420.1	3.00E-66	ATEP3; chitinase
Cit.10425.1.S1_s_at	875.1885	201.998	4.332661	AT5G05250.1	1.00E-42	unknown protein
Cit.5201.1.S1_at	154.2541	36.36	4.242415	AT2G14960.1	0	GH3.1
Cit.17309.1.S1_at	108.7375	26.0564	4.173162	AT3G03341.1	3.00E-28	unknown protein
Cit.9650.1.S1_at	372.3148	92.2079	4.037774	AT1G74950.1	4.00E-05	TIFY10B
Cit.11563.1.S1_at	387.6682	98.0526	3.953677	AT5G37430.1	3.00E-05	unknown protein
Cit.9301.1.S1_s_at	245.9319	62.303	3.947351	AT5G06570.2	3.00E-47	hydrolase
Cit.30494.1.S1_s_at	153.8006	39.5187	3.891848	AT2G40140.2	1.00E-147	CZF1; transcription factor
Cit.36207.1.S1_at	167.3225	43.1773	3.875245		NA	
Cit.4030.1.S1_at	354.6196	92.2967	3.842171	AT1G60190.1	4.00E-82	armadillo/beta-catenin repeat family protein / U-box domain-conta
Cit.18045.1.S1_s_at	371.8212	97.3406	3.819794	AT1G01250.1	3.00E-26	AP2 domain-containing transcription factor, putative
Cit.21310.1.S1_s_at	402.4677	106.599	3.775544	AT4G17500.1	3.00E-75	ATERF-1 (ETHYLENE RESPONSIVE ELEMENT BINDING FACTOR 1); DN
Cit.4752.1.S1_s_at	503.88	134.501	3.746286	AT5G54165.1	3.00E-08	unknown protein
Cit.14471.1.S1_at	735.2689	197.95	3.714423		NA	
Cit.1497.1.S1_s_at	318.5692	86.2051	3.695479	AT3G04070.1	1.00E-98	anac047 (Arabidopsis NAC domain containing protein 47); transcript
Cit.4566.1.S1_at	501.1548	135.728	3.692336	AT4G37580.1	6.00E-70	HLS1 (HOOKLESS 1); N-acetyltransferase
Cit.12814.1.S1_s_at	628.2499	170.948	3.675085	AT5G07330.1	2.00E-36	unknown protein
Cit.580.1.S1_x_at	594.1006	163.305	3.637986	AT3G04720.1	9.00E-47	PR4 (PATHOGENESIS-RELATED 4); chitin binding
Cit.18482.1.S1_at	82.11193	22.6934	3.618319	AT3G22240.1	7.00E-12	unknown protein
Cit.29801.1.S1_at	1011.57	281.901	3.588387	AT1G54050.1	2.00E-46	17.4 kDa class III heat shock protein (HSP17.4-CIII)
Cit.24173.1.S1_at	80.47869	22.4828	3.57957	AT1G07220.1	4.00E-64	FUNCTIONS IN: molecular_function unknown; INVOLVED IN: biologi
Cit.2809.1.S1_s_at	258.5512	73.0337	3.540162	AT1G68840.1	1.00E-122	RAV2 (REGULATOR OF THE ATPASE OF THE VACUOLAR MEMBRANE)
Cit.29575.1.S1_s_at	338.574	95.863	3.531854	AT4G29780.1	0	unknown protein
Cit.30568.1.S1_x_at	818.5267	233.06	3.512084	AT1G63830.1	2.00E-73	proline-rich family protein
Cit.21654.1.S1_s_at	307.0087	87.6076	3.504361	AT3G54420.1	3.00E-66	ATEP3; chitinase
Cit.30657.1.S1_s_at	1875.698	536.601	3.495515	AT4G36740.1	1.00E-50	ATHB40 (ARABIDOPSIS THALIANA HOMEOBOX PROTEIN 40); DNA bi
Cit.3523.1.S1_at	2579.647	742.473	3.474399	AT4G29780.1	0	unknown protein
Cit.18017.1.S1_s_at	156.895	45.3949	3.456229	AT1G72520.1	0	lipoxygenase, putative
Cit.30467.1.S1_at	1768.427	513.457	3.444158	AT5G39160.1	7.00E-74	germin-like protein (GLP2a) (GLP5a)
Cit.6647.1.S1_s_at	339.8363	99.331	3.421252	AT5G41080.2	1.00E-106	glycerophosphoryl diester phosphodiesterase family protein
Cit.28940.1.S1_x_at	82.28891	24.3682	3.376899	AT1G01300.1	3.00E-57	aspartyl protease family protein
Cit.18482.1.S1_s_at	277.6333	82.4319	3.368031	AT3G22240.1	7.00E-12	unknown protein
Cit.19380.1.S1_at	85.26698	25.8607	3.297166		NA	

Cit.17450.1.S1_s_at	1430.333	435.262	3.286141	AT2G46330.1	5.00E-13	AGP16 (ARABINOGALACTAN PROTEIN 16)
Cit.29686.1.S1_at	494.0578	151.804	3.254577	AT5G05340.1	1.00E-91	peroxidase, putative
Cit.22763.1.S1_s_at	110.6268	34.078	3.24628	AT3G23240.1	8.00E-52	ERF1 (ETHYLENE RESPONSE FACTOR 1); DNA binding / transcription
Cit.996.1.S1_s_at	817.848	252.985	3.232798	AT1G01470.1	6.00E-54	LEA14 (LATE EMBRYOGENESIS ABUNDANT 14)
Cit.26086.1.S1_at	79.3485	24.5764	3.228644	AT5G26990.1	8.00E-05	drought-responsive family protein
Cit.20928.1.S1_at	657.7989	204.34	3.219138		NA	
Cit.10595.1.S1_at	82.90783	25.8036	3.21304		NA	
Cit.12779.1.S1_at	159.451	49.9567	3.191787	AT4G23810.1	3.00E-57	WRKY53; DNA binding / protein binding / transcription activator/ tra
Cit.23036.1.S1_s_at	179.2122	56.2718	3.184758	AT2G14960.1	0	GH3.1
Cit.5456.1.S1_at	149.4129	46.9929	3.179477		NA	
Cit.7343.1.S1_at	163.5055	51.6771	3.163984	AT5G01830.1	1.00E-131	armadillo/beta-catenin repeat family protein / U-box domain-conta
Cit.29334.1.S1_at	459.3796	147.63	3.111687	AT5G28540.1	9.00E-75	BIP1; ATP binding
Cit.9464.1.S1_at	370.5475	119.718	3.095175	AT2G29500.1	1.00E-56	17.6 kDa class I small heat shock protein (HSP17.6B-CI)
Cit.26427.1.S1_x_at	82.01123	26.7291	3.068234		NA	
Cit.1557.1.S1_s_at	2268.865	740.257	3.064968		NA	
Cit.24426.1.S1_s_at	271.1029	89.2423	3.03783	AT1G73010.1	1.00E-110	phosphatase
Cit.881.1.S1_s_at	93.09341	30.6891	3.03344	AT3G55240.1	4.00E-36	Overexpression leads to PEL (Pseudo-Etiolation in Light) phenotype.
Cit.34844.1.S1_at	125.8053	41.6148	3.023092		NA	
Cit.35345.1.S1_s_at	295.8974	98.1215	3.015622	AT3G28340.1	1.00E-139	GATL10 (Galacturonosyltransferase-like 10); polygalacturonate 4-alp
Cit.13969.1.S1_s_at	3724.047	1235.34	3.014593	AT5G52640.1	0	ATHSP90.1 (HEAT SHOCK PROTEIN 90.1); ATP binding / unfolded pro
Cit.29533.1.S1_s_at	1265.314	420.584	3.008467	AT5G47230.1	2.00E-60	ERF5 (ETHYLENE RESPONSIVE ELEMENT BINDING FACTOR 5); DNA b
Cit.1270.1.S1_s_at	1170.734	392.487	2.982858	AT5G61600.1	4.00E-45	ethylene-responsive element-binding family protein
Cit.3226.1.S1_at	143.5901	48.393	2.967167	AT1G65020.1	3.00E-75	FUNCTIONS IN: molecular_function unknown; INVOLVED IN: biologi
Cit.3236.1.S1_at	87.80038	29.5913	2.967099	AT5G39670.1	1.00E-40	calcium-binding EF hand family protein
Cit.10155.1.S1_s_at	2422.245	821.422	2.948845	AT5G22250.1	1.00E-117	CCR4-NOT transcription complex protein, putative
Cit.14792.1.S1_at	118.2676	40.1202	2.947833	AT5G52230.1	4.00E-33	MBD13; methyl-CpG binding
Cit.8248.1.S1_x_at	1265.578	431.77	2.931142	AT5G09810.1	1.00E-71	ACT7 (ACTIN 7); structural constituent of cytoskeleton
Cit.29605.1.S1_at	148.1247	50.5781	2.928634		NA	
Cit.3805.1.S1_at	539.011	184.987	2.913771	AT1G71000.1	1.00E-39	heat shock protein binding
Cit.14913.1.S1_s_at	509.3535	174.87	2.912752	AT5G24090.1	2.00E-64	acidic endochitinase (CHIB1)
Cit.10278.1.S1_x_at	704.9589	242.299	2.909456	AT3G57520.1	5.00E-81	AtSIP2 (Arabidopsis thaliana seed imbibition 2); hydrolase, hydrolyzi
Cit.10258.1.S1_s_at	219.5039	75.5019	2.907264	AT1G32928.1	5.00E-09	unknown protein
Cit.21825.1.S1_at	71.39606	24.589	2.903572	AT1G64380.1	8.00E-13	AP2 domain-containing transcription factor, putative
Cit.26255.1.S1_at	71.74101	24.9048	2.880616		NA	
Cit.9569.1.S1_at	272.7871	94.7108	2.880212		NA	

Cit.9638.1.S1_s_at	3667.198	1273.33	2.880015	AT4G27670.1	9.00E-71	HSP21 (HEAT SHOCK PROTEIN 21)
Cit.13519.1.S1_at	498.7383	174.489	2.858278	AT1G80840.1	3.00E-71	WRKY40; transcription factor
Cit.30695.1.S1_s_at	730.3541	255.977	2.853204	AT3G21420.1	1.00E-151	oxidoreductase, 2OG-Fe(II) oxygenase family protein
Cit.30629.1.S1_at	106.5081	37.3929	2.848352	AT2G43790.1	1.00E-143	ATMPK6 (ARABIDOPSIS THALIANA MAP KINASE 6); MAP kinase/ kina
Cit.26141.1.S1_s_at	937.1971	329.203	2.846866		NA	
Cit.1779.1.S1_s_at	1439.003	507.818	2.833698	AT2G29500.1	4.00E-59	17.6 kDa class I small heat shock protein (HSP17.6B-CI)
Cit.17724.1.S1_s_at	66.17113	23.5047	2.815236	AT4G25810.1	1.00E-130	XTR6 (XYLOGLUCAN ENDOTRANSGLYCOSYLASE 6); hydrolase, acting
Cit.30448.1.S1_s_at	107.5038	38.3366	2.804211	AT3G01990.1	9.00E-66	ACR6; amino acid binding
Cit.16807.1.S1_at	259.5596	93.0116	2.790616	AT3G63010.1	9.00E-66	GID1B (GA INSENSITIVE DWARF1B); hydrolase
Cit.26534.1.S1_s_at	249.3201	89.4938	2.785891	AT3G21690.1	6.00E-64	MATE efflux family protein
Cit.2675.1.S1_s_at	2141.412	769.544	2.782703	AT5G51190.1	2.00E-48	AP2 domain-containing transcription factor, putative
Cit.13969.1.S1_at	415.7337	149.542	2.780056	AT5G52640.1	0	ATHSP90.1 (HEAT SHOCK PROTEIN 90.1); ATP binding / unfolded pro
Cit.2900.1.S1_at	323.6551	116.437	2.779659	AT1G73010.1	1.00E-110	phosphatase
Cit.31077.1.S1_at	56.57598	20.3589	2.778932	AT5G52430.1	3.00E-73	hydroxyproline-rich glycoprotein family protein
Cit.14472.1.S1_s_at	667.158	240.231	2.777157		NA	
Cit.22602.1.S1_at	106.5199	38.3694	2.776169	AT1G61800.1	3.00E-32	GPT2; antiporter/ glucose-6-phosphate transmembrane transporter
Cit.23585.1.S1_at	112.7476	40.6456	2.773921	AT1G01470.1	5.00E-31	LEA14 (LATE EMBRYOGENESIS ABUNDANT 14)
Cit.15923.1.S1_at	75.00955	27.0921	2.768692	AT1G58420.1	7.00E-26	FUNCTIONS IN: molecular_function unknown; INVOLVED IN: biologi
Cit.29868.1.S1_s_at	334.5817	120.912	2.767155	AT1G31130.1	3.00E-26	unknown protein
Cit.18536.1.S1_at	189.7378	68.617	2.765172		NA	
Cit.11666.1.S1_s_at	1388.989	503.594	2.758151	AT5G59550.1	9.00E-63	zinc finger (C3HC4-type RING finger) family protein
Cit.30648.1.S1_s_at	6149.544	2234.28	2.752356	AT4G27670.1	2.00E-32	HSP21 (HEAT SHOCK PROTEIN 21)
Cit.6076.1.S1_s_at	483.9482	176.01	2.749548	AT1G22990.1	2.00E-51	heavy-metal-associated domain-containing protein / copper chaper
Cit.11676.1.S1_at	139.2137	50.958	2.731929		NA	
Cit.4047.1.S1_at	65.96131	24.2072	2.724869	AT2G02990.1	7.00E-96	RNS1 (RIBONUCLEASE 1); endoribonuclease/ ribonuclease
Cit.10057.1.S1_s_at	91.42631	33.603	2.720781	AT3G47340.1	1.00E-19	ASN1 (GLUTAMINE-DEPENDENT ASPARAGINE SYNTHASE 1); asparag
Cit.17840.1.S1_s_at	123.2109	45.3626	2.716133	AT1G02070.1	2.00E-13	unknown protein
Cit.37368.1.S1_at	56.54435	20.8433	2.712832	AT1G23090.1	2.00E-71	AST91 (SULFATE TRANSPORTER 91); sulfate transmembrane transpo
Cit.10062.1.S1_at	67.91696	25.0689	2.709214	AT3G47340.1	1.00E-19	ASN1 (GLUTAMINE-DEPENDENT ASPARAGINE SYNTHASE 1); asparag
Cit.39099.1.S1_at	73.00797	26.9564	2.708372	AT3G57170.1	1.00E-31	N-acetylglucosaminyl transferase component family protein / Gpi1 f
Cit.21476.1.S1_at	71.22156	26.3767	2.700172		NA	
Cit.22463.1.S1_s_at	424.505	157.244	2.699661	AT4G27410.2	1.00E-110	RD26 (RESPONSIVE TO DESICCATION 26); transcription activator/ tra
Cit.753.1.S1_x_at	1173.591	435.485	2.694904	AT3G04720.1	1.00E-46	PR4 (PATHOGENESIS-RELATED 4); chitin binding
Cit.30576.1.S1_s_at	1733.248	644.463	2.689445	AT2G26150.1	1.00E-105	ATHSFA2; DNA binding / transcription factor
Cit.12163.1.S1_s_at	442.6287	164.64	2.688459	AT4G17900.1	4.00E-94	zinc-binding family protein

Cit.11083.1.S1_s_at	311.3331	116.633	2.669335	AT3G02040.1	1.00E-141	SRG3 (senescence-related gene 3); glycerophosphodiester phospho
Cit.28237.1.S1_at	311.3853	116.793	2.666137		NA	
Cit.13244.1.S1_at	195.4219	73.3716	2.663455	AT5G42380.1	1.00E-36	CML37 (CALMODULIN LIKE 37); calcium ion binding
Cit.22963.1.S1_x_at	944.5298	357.169	2.644491	AT3G19430.1	6.00E-08	late embryogenesis abundant protein-related / LEA protein-related
Cit.14156.1.S1_s_at	311.072	117.773	2.641296	AT1G73500.1	1.00E-116	MKK9 (MAP KINASE KINASE 9); MAP kinase kinase/ kinase/ protein k
Cit.9020.1.S1_s_at	66.15484	25.0879	2.63692	AT4G30880.1	5.00E-21	protease inhibitor/seed storage/lipid transfer protein (LTP) family p
Cit.15726.1.S1_at	60.89809	23.1014	2.636119	AT3G14460.1	8.00E-06	disease resistance protein (NBS-LRR class), putative
Cit.5438.1.S1_at	69.78198	26.4826	2.635011	AT2G25625.2	8.00E-20	unknown protein
Cit.32906.1.S1_at	101.9973	38.732	2.633412	AT1G21600.2	1.00E-79	PTAC6 (PLASTID TRANSCRIPTIONALLY ACTIVE6)
Cit.16146.1.S1_at	53.8716	20.4736	2.631278	AT2G34470.2	4.00E-07	UREG (UREASE ACCESSORY PROTEIN G); ATP binding / metal ion bin
Cit.9705.1.S1_at	64.65701	24.5731	2.631216	AT3G57270.1	1.00E-109	BG1 (BETA-1,3-GLUCANASE 1); catalytic/ cation binding / hydrolase,
Cit.3098.1.S1_at	52.64035	20.0234	2.628942	AT5G19860.1	5.00E-27	unknown protein
Cit.5793.1.S1_s_at	458.5871	174.527	2.627603	AT3G03341.1	3.00E-28	unknown protein
Cit.1779.1.S1_at	1808.322	690.662	2.618246	AT2G29500.1	4.00E-59	17.6 kDa class I small heat shock protein (HSP17.6B-CI)
Cit.25906.1.S1_at	315.662	120.615	2.617095	AT1G07400.1	3.00E-12	17.8 kDa class I heat shock protein (HSP17.8-CI)
Cit.23502.1.S1_at	52.45472	20.0473	2.616554	AT2G17420.1	3.00E-13	NTRA (NADPH-DEPENDENT THIOREDOXIN REDUCTASE A); thioredox
Cit.4526.1.S1_s_at	59.35397	22.7616	2.607636	AT4G34135.1	1.00E-131	UGT73B2 (UDP-GLUCOSYLTRANSFERASE 73B2); UDP-glucosyltransfe
Cit.19496.1.S1_at	394.624	152.422	2.589023	AT5G37670.1	2.00E-44	15.7 kDa class I-related small heat shock protein-like (HSP15.7-CI)
Cit.31840.1.S1_at	675.6536	261.075	2.587965	AT4G38500.1	5.00E-61	unknown protein
Cit.32389.1.S1_at	774.5414	300.216	2.579951	AT3G63300.1	5.00E-43	phosphoinositide binding
Cit.19482.1.S1_at	1544.804	599.602	2.576381	AT1G26250.1	8.00E-28	proline-rich extensin, putative
Cit.5514.1.S1_at	92.41304	35.9216	2.572631	AT3G63060.1	4.00E-56	EDL3 (EID1-like 3)
Cit.20512.1.S1_x_at	115.0602	44.7659	2.570265		NA	
Cit.16710.1.S1_at	59.41591	23.3526	2.544292	AT2G28590.1	1.00E-49	protein kinase family protein
Cit.10152.1.S1_s_at	295.2917	116.118	2.543033	AT4G27410.2	1.00E-110	RD26 (RESPONSIVE TO DESICCATION 26); transcription activator/ tra
Cit.10381.1.S1_s_at	1229.843	483.974	2.541136	AT2G17705.1	2.00E-49	unknown protein
Cit.28253.1.S1_at	58.38182	23.0072	2.537548	AT3G26220.1	9.00E-54	CYP71B3; electron carrier/ heme binding / iron ion binding / monoo
Cit.17388.1.S1_at	2926.455	1153.51	2.537002	AT2G45760.1	1.00E-25	BAP2 (BON ASSOCIATION PROTEIN 2)
Cit.11086.1.S1_at	515.9026	203.668	2.533055	AT1G02070.1	2.00E-13	unknown protein
Cit.14905.1.S1_s_at	78.52132	31.0859	2.525943	AT5G65140.1	1.00E-136	trehalose-6-phosphate phosphatase, putative
Cit.24483.1.S1_s_at	64.03828	25.4035	2.520849	AT4G37870.1	1.00E-164	PCK1 (PHOSPHOENOLPYRUVATE CARBOXYKINASE 1); ATP binding /
Cit.10669.1.S1_s_at	3699.643	1468.31	2.519654	AT2G15960.1	7.00E-09	unknown protein
Cit.19733.1.S1_x_at	63.03938	25.0736	2.514172		NA	
Cit.22551.1.S1_at	137.169	54.6495	2.509976	AT1G34220.2	5.00E-10	unknown protein
Cit.29796.1.S1_at	111.139	44.309	2.508273	AT1G21010.1	3.00E-07	unknown protein

Cit.23912.1.S1_s_at	65.84014	26.2915	2.504235	AT5G59730.1	1.00E-54	ATEXO70H7 (EXOCYST SUBUNIT EXO70 FAMILY PROTEIN H7); protei
Cit.20851.1.S1_s_at	150.96	60.5529	2.493025	AT2G01590.1	2.00E-19	CRR3 (CHLORORESPIRATORY REDUCTION 3)
Cit.31025.1.S1_at	541.8508	217.994	2.485626	AT4G39250.1	4.00E-26	ATRL1 (ARABIDOPSIS RAD-LIKE 1); DNA binding / transcription facto
Cit.40276.1.S1_at	79.54237	32.0706	2.480226	AT3G47930.2	4.00E-49	ATGLDH (L-GALACTONO-1,4-LACTONE DEHYDOROGENASE); L-gulon
Cit.2915.1.S1_s_at	617.1327	248.852	2.479915	AT5G63130.1	4.00E-43	octicosapeptide/Phox/Bem1p (PB1) domain-containing protein
Cit.5477.1.S1_s_at	116.0279	46.953	2.471149	AT1G31130.1	2.00E-36	unknown protein
Cit.5184.1.S1_at	66.79814	27.0727	2.467363	AT4G12010.1	1.00E-117	disease resistance protein (TIR-NBS-LRR class), putative
Cit.22998.1.S1_s_at	318.0079	128.928	2.466562	AT2G26070.1	2.00E-95	RTE1 (REVERSION-TO-ETHYLENE SENSITIVITY1)
Cit.1819.1.S1_s_at	1036.48	420.336	2.465838	AT2G28710.1	1.00E-34	zinc finger (C2H2 type) family protein
Cit.24884.1.S1_at	90.19179	36.593	2.464731	AT1G48300.1	1.00E-15	unknown protein
Cit.15528.1.S1_at	4294.548	1742.71	2.464294	AT1G74310.1	1.00E-132	ATHSP101 (ARABIDOPSIS THALIANA HEAT SHOCK PROTEIN 101); AT
Cit.31333.1.S1_at	372.2104	151.042	2.464287	AT3G22830.1	4.00E-78	AT-HSFA6B; DNA binding / transcription factor
Cit.5891.1.S1_at	150.0098	60.8931	2.463495	AT4G17500.1	3.00E-75	ATERF-1 (ETHYLENE RESPONSIVE ELEMENT BINDING FACTOR 1); DN
Cit.4810.1.S1_at	69.83714	28.3492	2.463459	AT3G23240.1	2.00E-64	ERF1 (ETHYLENE RESPONSE FACTOR 1); DNA binding / transcription
Cit.30095.1.S1_at	56.76612	23.1205	2.455226	AT1G29340.1	8.00E-22	PUB17 (PLANT U-BOX 17); ubiquitin-protein ligase
Cit.3757.1.S1_at	363.3571	148.159	2.452477	AT2G46400.1	2.00E-06	WRKY46; transcription factor
Cit.14173.1.S1_at	527.1614	215.059	2.451236		NA	
Cit.15485.1.S1_at	70.82983	28.9078	2.450202	AT5G05140.1	5.00E-56	transcription elongation factor-related
Cit.26425.1.S1_at	151.2337	61.7246	2.450136		NA	
Cit.29475.1.S1_at	88.40646	36.2033	2.441942		NA	
Cit.26433.1.S1_s_at	516.8413	212.385	2.433511	AT1G52560.1	4.00E-73	26.5 kDa class I small heat shock protein-like (HSP26.5-P)
Cit.12593.1.S1_at	168.1789	69.2254	2.429438	AT1G52560.1	4.00E-73	26.5 kDa class I small heat shock protein-like (HSP26.5-P)
Cit.22035.1.S1_at	59.54433	24.5294	2.427465		NA	
Cit.2729.1.S1_at	232.0547	95.6407	2.426317	AT3G12760.1	1.00E-116	FUNCTIONS IN: molecular_function unknown; INVOLVED IN: biologi
Cit.35355.1.S1_s_at	221.8793	91.4652	2.425834	AT1G18100.1	8.00E-55	E12A11; phosphatidylethanolamine binding
Cit.12739.1.S1_at	686.0395	283.386	2.420868		NA	
Cit.37084.1.S1_at	59.05798	24.4863	2.41188		NA	
Cit.32671.1.S1_at	116.4612	48.4118	2.405636	AT1G55270.1	3.00E-90	kelch repeat-containing F-box family protein
Cit.18219.1.S1_x_at	75.20738	31.2651	2.405472		NA	
Cit.38045.1.S1_at	48.44115	20.1435	2.404799	AT5G18730.1	2.00E-26	FUNCTIONS IN: molecular_function unknown; INVOLVED IN: biologi
Cit.18435.1.S1_at	61.06514	25.4172	2.402516	AT4G08850.1	7.00E-18	kinase
Cit.37986.1.S1_at	68.20107	28.436	2.398405		NA	
Cit.29911.1.S1_at	59.14367	24.6878	2.395661	AT5G40270.1	1.00E-107	metal-dependent phosphohydrolase HD domain-containing protein
Cit.38057.1.S1_at	71.22424	29.7974	2.390288		NA	
Cit.5956.1.S1_s_at	430.7426	180.722	2.383457	AT2G18660.1	7.00E-33	EXLB3 (EXPANSIN-LIKE B3 PRECURSOR)

Cit.8959.1.S1_at	95.66409	40.1542	2.382417	AT4G10960.1	1.00E-164	UGE5 (UDP-D-glucose/UDP-D-galactose 4-epimerase 5); UDP-glucos
Cit.30804.1.S1_s_at	48.19688	20.2309	2.382335		NA	
Cit.4318.1.S1_s_at	419.6964	176.9	2.372512	AT2G36400.1	1.00E-108	AtGRF3 (GROWTH-REGULATING FACTOR 3); transcription activator
Cit.28270.1.S1_at	87.6249	36.9656	2.370446	AT1G32160.1	3.00E-34	unknown protein
Cit.3042.1.S1_s_at	633.3943	267.872	2.364545	AT1G52565.1	1.00E-31	unknown protein
Cit.21825.1.S1_s_at	161.9526	68.533	2.363132	AT1G64380.1	8.00E-13	AP2 domain-containing transcription factor, putative
Cit.11240.1.S1_at	54.34597	23.0085	2.361992	AT1G02190.1	5.00E-62	CER1 protein, putative
Cit.36225.1.S1_s_at	56.23159	23.8739	2.355355	AT1G09155.1	5.00E-57	AtPP2-B15 (Phloem protein 2-B15); carbohydrate binding
Cit.20174.1.S1_x_at	61.8435	26.2584	2.355192		NA	
Cit.35040.1.S1_at	94.53815	40.1526	2.354473		NA	
Cit.12431.1.S1_s_at	934.9904	397.959	2.349464	AT4G19840.1	1.00E-36	ATPP2-A1; carbohydrate binding
Cit.9568.1.S1_s_at	3760.414	1602.09	2.347196	AT5G64260.1	1.00E-125	EXL2 (EXORDIUM LIKE 2)
Cit.19319.1.S1_at	55.27887	23.6145	2.340885	AT3G49290.2	8.00E-17	ABIL2 (ABL INTERACTOR-LIKE PROTEIN 2)
Cit.18040.1.S1_at	8035.775	3443.89	2.333346	AT1G21310.1	9.00E-27	ATEXT3 (EXTENSIN 3); structural constituent of cell wall
Cit.17142.1.S1_s_at	1113.714	477.875	2.330557	AT5G44210.1	8.00E-43	ERF9 (ERF DOMAIN PROTEIN 9); DNA binding / transcription factor/
Cit.9300.1.S1_s_at	433.8609	186.239	2.329598	AT3G28860.1	7.00E-92	ABCB19; ATPase, coupled to transmembrane movement of substan
Cit.7921.1.S1_at	48.83773	20.9804	2.32778		NA	
Cit.6740.1.S1_at	133.2679	57.3013	2.325738	AT3G54460.1	2.00E-53	SNF2 domain-containing protein / helicase domain-containing prote
Cit.11255.1.S1_at	2080.211	896.944	2.319221		NA	
Cit.15137.1.S1_at	75.45338	32.7668	2.302741	AT2G41380.1	1.00E-68	embryo-abundant protein-related
Cit.21771.1.S1_x_at	55.3518	24.0689	2.299722	AT5G20650.1	3.00E-11	COPT5; copper ion transmembrane transporter/ high affinity coppe
Cit.2778.1.S1_at	320.6083	139.486	2.298496	AT3G44400.2	1.00E-08	disease resistance protein (TIR-NBS-LRR class), putative
Cit.7387.1.S1_at	122.8793	53.5748	2.293602	AT4G01860.2	3.00E-28	transducin family protein / WD-40 repeat family protein
Cit.6373.1.S1_at	68.12839	29.8066	2.285681	AT5G48800.1	2.00E-81	phototropic-responsive NPH3 family protein
Cit.15796.1.S1_at	60.74276	26.6271	2.281241	AT1G07530.1	2.00E-37	SCL14 (SCARECROW-LIKE 14); transcription factor
Cit.14665.1.S1_at	1722.984	756.051	2.278926	AT2G32120.2	2.00E-94	HSP70T-2 (HEAT-SHOCK PROTEIN 70T-2); ATP binding
Cit.37829.1.S1_s_at	120.2451	52.7885	2.277865	AT5G22380.1	5.00E-67	anac090 (Arabidopsis NAC domain containing protein 90); transcript
Cit.1200.1.S1_s_at	293.9787	129.589	2.268541	AT4G11650.1	9.00E-81	ATOSM34 (osmotin 34)
Cit.38457.1.S1_at	68.68005	30.2809	2.268096	AT4G33520.3	7.00E-34	PAA1 (P-TYPE ATP-ASE 1); ATPase, coupled to transmembrane move
Cit.10465.1.S1_s_at	6331.816	2794.36	2.265927	AT2G26150.1	1.00E-105	ATHSFA2; DNA binding / transcription factor
Cit.14998.1.S1_at	161.273	71.1904	2.265375	AT4G03500.1	1.00E-33	ankyrin repeat family protein
Cit.34952.1.S1_at	45.45993	20.0704	2.265029		NA	
Cit.30692.1.S1_s_at	462.1987	204.098	2.264593	AT2G22500.1	1.00E-128	UCP5 (UNCOUPLING PROTEIN 5); binding
Cit.14906.1.S1_at	153.0365	67.6039	2.263725	AT5G65140.1	1.00E-136	trehalose-6-phosphate phosphatase, putative
Cit.6860.1.S1_at	220.2825	97.5621	2.25787	AT3G21690.1	6.00E-64	MATE efflux family protein

Cit.4721.1.S1_at	51.63823	22.8727	2.257633	AT2G36970.1	2.00E-22	UDP-glucoronosyl/UDP-glucosyl transferase family protein
Cit.10686.1.S1_at	351.9774	155.919	2.257433	AT1G60420.1	8.00E-86	DC1 domain-containing protein
Cit.39721.1.S1_x_at	8563.297	3799.39	2.25386	AT3G46230.1	7.00E-69	ATHSP17.4
Cit.12502.1.S1_s_at	498.4634	221.503	2.250369	AT3G45600.1	1.00E-133	TET3 (TETRASPANIN3)
Cit.29148.1.S1_at	65.15757	29.1723	2.23354	AT1G35190.1	3.00E-42	oxidoreductase, 2OG-Fe(II) oxygenase family protein
Cit.7578.1.S1_at	85.83076	38.5407	2.227019	AT1G18980.1	1.00E-71	germin-like protein, putative
Cit.37988.1.S1_at	54.54158	24.4909	2.227013		NA	
Cit.17957.1.S1_at	52.55517	23.6072	2.226237	AT5G07330.1	4.00E-35	unknown protein
Cit.394.1.S1_at	53.81438	24.1817	2.225415	AT5G01300.1	2.00E-63	phosphatidylethanolamine-binding family protein
Cit.11999.1.S1_at	59.14948	26.5806	2.22529	ATMG00560.1	1.00E-108	encodes a mitochondrial ribosomal protein L2, a constituent of the l
Cit.12252.1.S1_at	95.55598	43.04	2.220166	AT2G14960.1	0	GH3.1
Cit.13688.1.S1_at	333.0664	150.148	2.218257	AT2G26070.1	2.00E-95	RTE1 (REVERSION-TO-ETHYLENE SENSITIVITY1)
Cit.14499.1.S1_at	117.1644	52.985	2.211274	AT2G19130.1	1.00E-125	S-locus lectin protein kinase family protein
Cit.30394.1.S1_at	76.38958	34.8656	2.190973	AT5G46880.1	1.00E-147	HB-7 (HOMEOBOX-7); DNA binding / transcription factor
Cit.14755.1.S1_at	1517.12	692.577	2.190543	AT1G17870.1	2.00E-63	EGY3 (ETHYLENE-DEPENDENT GRAVITROPISM-DEFICIENT AND YELL(
Cit.18491.1.S1_at	691.7198	316.185	2.187708		NA	
Cit.23565.1.S1_at	131.4179	60.3989	2.175834	AT3G25250.1	2.00E-60	AGC2-1 (OXIDATIVE SIGNAL-INDUCIBLE1); kinase
Cit.18784.1.S1_at	82.85077	38.0927	2.17498	AT1G02460.1	3.00E-24	glycoside hydrolase family 28 protein / polygalacturonase (pectinas(
Cit.26397.1.S1_s_at	206.3892	94.925	2.174235	AT3G43540.2	4.00E-13	unknown protein
Cit.21774.1.S1_at	195.7935	90.2193	2.170195	AT1G52560.2	7.00E-06	26.5 kDa class I small heat shock protein-like (HSP26.5-P)
Cit.32832.1.S1_at	442.5897	204.223	2.167185	AT4G02120.1	2.00E-69	CTP synthase, putative / UTP--ammonia ligase, putative
Cit.26004.1.S1_at	44.85029	20.6997	2.166713		NA	
Cit.20509.1.S1_at	52.73866	24.3479	2.166049		NA	
Cit.9500.1.S1_s_at	4940.078	2281.06	2.165697	AT5G59720.1	4.00E-56	HSP18.2 (heat shock protein 18.2)
Cit.31510.1.S1_at	66.57922	30.7522	2.165025	AT5G44360.1	1.00E-60	FAD-binding domain-containing protein
Cit.38.1.S1_x_at	142.3465	65.7682	2.164367		NA	
Cit.1775.1.S1_s_at	120.5591	55.7519	2.16242	AT1G56600.1	1.00E-156	AtGolS2 (Arabidopsis thaliana galactinol synthase 2); transferase, tra
Cit.38508.1.S1_at	108.6883	50.268	2.162178	AT3G44240.1	5.00E-47	CCR4-NOT transcription complex protein, putative
Cit.3215.1.S1_s_at	102.3203	47.4959	2.154296	AT5G03230.1	8.00E-41	unknown protein
Cit.12388.1.S1_at	160.7285	74.7209	2.151051	AT2G40095.1	9.00E-66	unknown protein
Cit.6136.1.S1_at	84.69527	39.3958	2.149854	AT3G51570.1	3.00E-05	disease resistance protein (TIR-NBS-LRR class), putative
Cit.38018.1.S1_at	167.3	77.8243	2.149713		NA	
Cit.29568.1.S1_s_at	121.9408	56.7795	2.147621	AT1G01720.1	1.00E-100	ATAF1; transcription activator/ transcription factor
Cit.10279.1.S1_at	68.18769	31.7541	2.147364	AT3G57520.1	0	AtSIP2 (Arabidopsis thaliana seed imbibition 2); hydrolase, hydrolyzi
Cit.18030.1.S1_at	69.88857	32.5487	2.147201	AT1G10490.1	3.00E-39	unknown protein

Cit.14884.1.S1_at	52.21845	24.3302	2.146242	AT3G23100.2	2.00E-85	XRCC4; protein C-terminus binding
Cit.24446.1.S1_s_at	46.67532	21.7764	2.143393	AT1G44910.2	2.00E-21	protein binding
Cit.25037.1.S1_at	257.3205	120.447	2.136381	AT2G31880.1	1.00E-39	leucine-rich repeat transmembrane protein kinase, putative
Cit.5159.1.S1_at	56.3909	26.4489	2.13207	AT1G14310.1	4.00E-11	haloacid dehalogenase-like hydrolase family protein
Cit.9239.1.S1_at	95.44036	44.8296	2.128957	AT5G25610.1	1.00E-117	RD22; nutrient reservoir
Cit.21700.1.S1_at	108.1812	50.8597	2.127054	AT3G52430.1	8.00E-43	PAD4 (PHYTOALEXIN DEFICIENT 4); lipase/ protein binding / triacylg
Cit.16619.1.S1_at	86.62968	40.7393	2.126443	AT3G63380.1	1.00E-127	calcium-transporting ATPase, plasma membrane-type, putative / Ca
Cit.2392.1.S1_at	57.67593	27.1311	2.125823	AT1G02800.1	0	ATCEL2; cellulase/ hydrolase, hydrolyzing O-glycosyl compounds
Cit.2554.1.S1_s_at	1902.444	894.951	2.125752	AT1G48300.1	2.00E-45	unknown protein
Cit.35397.1.S1_at	88.19701	41.5052	2.124962	AT3G48190.1	1.00E-100	ATM (ATAXIA-TELANGIECTASIA MUTATED); 1-phosphatidylinositol-3
Cit.33130.1.S1_at	89.02492	41.9106	2.124163		NA	
Cit.8444.1.S1_s_at	12522.61	5896.43	2.12376	AT3G46230.1	8.00E-70	ATHSP17.4
Cit.7334.1.S1_at	172.5542	81.2754	2.123081	AT5G17350.1	7.00E-37	unknown protein
Cit.35055.1.S1_s_at	51.99083	24.4907	2.122881	AT2G38470.1	1.00E-124	WRKY33; transcription factor
Cit.20663.1.S1_at	42.70801	20.1279	2.121831	AT2G39760.1	3.00E-08	BPM3; protein binding
Cit.31454.1.S1_at	54.49398	25.7062	2.119874	AT4G08850.2	2.00E-29	kinase
Cit.24760.1.S1_at	73.27313	34.5671	2.119735	AT4G21380.1	4.00E-56	ARK3 (A. THALIANA RECEPTOR KINASE 3); kinase/ transmembrane r
Cit.12777.1.S1_at	1037.133	489.448	2.118987	AT4G36850.1	1.00E-143	INVOLVED IN: biological_process unknown; LOCATED IN: membrane
Cit.30622.1.S1_s_at	2733.411	1291	2.117277		NA	
Cit.29626.1.S1_s_at	52.43725	24.9006	2.105863	AT5G50260.1	1.00E-158	cysteine proteinase, putative
Cit.14908.1.S1_at	57.71023	27.4192	2.10474	AT2G27150.2	1.00E-142	AAO3 (Abscisic ALDEHYDE OXIDASE 3); abscisic aldehyde oxidase/ al
Cit.7675.1.S1_at	627.2515	298.407	2.101998		NA	
Cit.30204.1.S1_at	297.2804	141.661	2.09854	AT4G12430.1	3.00E-23	trehalose-6-phosphate phosphatase, putative
Cit.14381.1.S1_at	287.8945	137.31	2.096682	AT5G02220.1	4.00E-09	unknown protein
Cit.14222.1.S1_at	277.7669	132.744	2.092505	AT1G58170.1	8.00E-56	disease resistance-responsive protein-related / dirigent protein-rela
Cit.38213.1.S1_at	117.671	56.2446	2.09213	AT1G30370.1	3.00E-37	lipase class 3 family protein
Cit.12503.1.S1_at	301.8121	144.518	2.088402	AT3G45600.1	1.00E-133	TET3 (TETRASPANIN3)
Cit.12180.1.S1_at	46.7522	22.3953	2.08759	AT2G46225.2	1.00E-21	ABIL1 (Abi-1-like 1)
Cit.17938.1.S1_at	140.717	67.43	2.086862	AT3G49780.1	8.00E-14	ATPSK4 (PHYTOSULFOKINE 4 PRECURSOR); growth factor
Cit.17845.1.S1_s_at	1932.579	927.491	2.083663		NA	
Cit.15887.1.S1_at	80.695	38.7461	2.08266	AT3G59940.1	2.00E-50	kelch repeat-containing F-box family protein
Cit.39714.1.S1_at	266.034	127.74	2.082623	AT4G01580.1	2.00E-23	transcriptional factor B3 family protein
Cit.39512.1.S1_at	138.5209	66.6921	2.07702	AT2G40700.1	1.00E-94	DEAD/DEAH box helicase, putative (RH17)
Cit.4026.1.S1_s_at	3073.031	1480.35	2.075887	AT4G27280.1	3.00E-42	calcium-binding EF hand family protein
Cit.8395.1.S1_x_at	304.048	146.516	2.075188	AT3G05890.1	5.00E-20	RCI2B (RARE-COLD-INDUCIBLE 2B)

Cit.2899.1.S1_s_at	174.9601	84.3568	2.074048	AT1G73010.1	1.00E-110	phosphatase
Cit.21856.1.S1_at	54.49191	26.3019	2.071787		NA	
Cit.6261.1.S1_at	285.281	137.731	2.071288	AT4G39680.2	1.00E-40	SAP domain-containing protein
Cit.13425.1.S1_at	563.1919	272.224	2.068855	AT2G39730.1	1.00E-169	RCA (RUBISCO ACTIVASE); ADP binding / ATP binding / enzyme regu
Cit.13667.1.S1_s_at	105.1349	50.8696	2.066754	AT4G05070.1	3.00E-09	unknown protein
Cit.21610.1.S1_at	145.3523	70.5036	2.06163	AT2G29260.1	7.00E-70	tropinone reductase, putative / tropine dehydrogenase, putative
Cit.17258.1.S1_at	249.9715	121.392	2.059216	AT1G12810.1	4.00E-21	proline-rich family protein
Cit.23299.1.S1_x_at	83.9226	40.7786	2.058008	AT5G14030.4	5.00E-33	translocon-associated protein beta (TRAPB) family protein
Cit.17264.1.S1_s_at	152.7214	74.2253	2.057538	AT2G05540.1	4.00E-16	glycine-rich protein
Cit.29381.1.S1_at	50.4201	24.5115	2.056996	AT3G53970.1	3.00E-89	proteasome inhibitor-related
Cit.9048.1.S1_s_at	1887.55	917.878	2.056429	AT2G40140.2	1.00E-110	CZF1; transcription factor
Cit.3036.1.S1_s_at	642.7108	312.781	2.054828	AT3G04920.1	9.00E-63	40S ribosomal protein S24 (RPS24A)
Cit.36305.1.S1_s_at	72.90926	35.5271	2.052217	AT1G20693.3	5.00E-40	HMGB2 (HIGH MOBILITY GROUP B 2); DNA binding / chromatin binc
Cit.14011.1.S1_at	3085.949	1504.67	2.050909	AT3G16050.1	1.00E-122	A37; protein heterodimerization
Cit.16105.1.S1_at	92.58147	45.2101	2.047803	AT4G11130.1	2.00E-72	RDR2 (RNA-DEPENDENT RNA POLYMERASE 2); RNA-directed RNA pc
Cit.14216.1.S1_at	247.1352	120.688	2.047725	AT1G31310.1	2.00E-88	hydroxyproline-rich glycoprotein family protein
Cit.31055.1.S1_at	243.1682	118.797	2.046931	AT3G27880.1	5.00E-21	unknown protein
Cit.31257.1.S1_at	51.36586	25.113	2.04539	AT1G09280.1	3.00E-70	FUNCTIONS IN: molecular_function unknown; INVOLVED IN: biologi
Cit.40290.1.S1_at	57.73526	28.2274	2.045363	AT1G60460.3	5.00E-40	unknown protein
Cit.18673.1.S1_at	69.20434	33.8376	2.045191	AT1G12630.1	2.00E-34	DNA binding / transcription activator/ transcription factor
Cit.29915.1.S1_at	48.3023	23.6318	2.043952		NA	
Cit.30067.1.S1_at	392.3867	192.232	2.041212	AT3G49950.1	1.00E-42	scarecrow transcription factor family protein
Cit.280.1.S1_at	776.1348	380.348	2.040589	AT1G74310.1	1.00E-143	ATHSP101 (ARABIDOPSIS THALIANA HEAT SHOCK PROTEIN 101); AT
Cit.12050.1.S1_s_at	64.92393	31.8422	2.03893	AT5G14180.1	1.00E-119	MPL1 (MYZUS PERSICAE-INDUCED LIPASE 1); catalytic
Cit.5189.1.S1_at	41.99173	20.6009	2.038346	AT1G08830.2	3.00E-42	CSD1 (COPPER/ZINC SUPEROXIDE DISMUTASE 1); superoxide dismu
Cit.31072.1.S1_at	73.25816	36.009	2.034439	AT3G06880.1	6.00E-07	nucleotide binding
Cit.17018.1.S1_s_at	343.7345	169.035	2.033512	AT1G23040.1	3.00E-31	hydroxyproline-rich glycoprotein family protein
Cit.18445.1.S1_at	100.6387	49.5269	2.031999	AT4G12680.1	3.00E-36	unknown protein
Cit.11296.1.S1_at	466.7387	229.962	2.029631	AT5G37540.1	2.00E-87	aspartyl protease family protein
Cit.29255.1.S1_at	59.35962	29.2853	2.026945		NA	
Cit.9345.1.S1_at	81.91392	40.4593	2.024603	AT5G52570.1	7.00E-98	BETA-OHASE 2 (BETA-CAROTENE HYDROXYLASE 2); carotene beta-ri
Cit.36875.1.S1_at	44.12446	21.8086	2.023262	AT5G56660.1	2.00E-76	ILL2; IAA-Ala conjugate hydrolase/ IAA-amino acid conjugate hydrol
Cit.1854.1.S1_at	98.04984	48.5215	2.02075		NA	
Cit.20049.1.S1_x_at	4274.663	2115.77	2.020378		NA	
Cit.21579.1.S1_s_at	1043.393	516.496	2.02014	AT4G39250.1	4.00E-26	ATRL1 (ARABIDOPSIS RAD-LIKE 1); DNA binding / transcription facto

Cit.21424.1.S1_at	43.50453	21.5442	2.019316	AT3G48120.1	3.00E-27	unknown protein
Cit.23534.1.S1_at	156.1103	77.3301	2.018752	AT1G15110.1	3.00E-68	phosphatidyl serine synthase family protein
Cit.22186.1.S1_s_at	113.5991	56.2852	2.018275	AT4G34050.1	1.00E-101	caffeoyl-CoA 3-O-methyltransferase, putative
Cit.10032.1.S1_x_at	223.232	110.845	2.01392	AT1G75750.1	3.00E-37	GASA1 (GAST1 PROTEIN HOMOLOG 1)
Cit.25482.1.S1_at	78.09297	38.7927	2.013085	AT1G18070.2	2.00E-90	EF-1-alpha-related GTP-binding protein, putative
Cit.3513.1.S1_at	409.012	203.285	2.012018	AT3G06190.1	1.00E-127	BPM2 (BTB-POZ AND MATH DOMAIN 2); protein binding
Cit.3069.1.S1_at	74.14382	36.9156	2.00847	AT5G50915.2	3.00E-44	basic helix-loop-helix (bHLH) family protein
Cit.4608.1.S1_at	68.15147	33.9796	2.005657	AT1G23550.1	3.00E-36	SRO2 (SIMILAR TO RCD ONE 2); NAD+ ADP-ribosyltransferase
Cit.15301.1.S1_at	5767.589	2880.55	2.002256	AT1G74310.1	2.00E-29	ATHSP101 (ARABIDOPSIS THALIANA HEAT SHOCK PROTEIN 101); AT
Cit.32370.1.S1_at	152.6674	76.3221	2.000303	AT2G20330.1	2.00E-92	transducin family protein / WD-40 repeat family protein
Cit.2942.1.S1_at	22.87509	45.7332	-2.0006		NA	
Cit.29207.1.S1_at	421.7378	843.713	-2.00056	AT2G22795.1	2.00E-16	unknown protein
Cit.595.1.S1_s_at	42.6151	85.3084	-2.00184	AT3G02885.1	3.00E-36	GASA5 (GAST1 PROTEIN HOMOLOG 5)
Cit.2486.1.S1_at	54.10672	108.364	-2.00279	AT5G64920.1	2.00E-85	CIP8 (COP1-INTERACTING PROTEIN 8); protein binding / zinc ion bin
Cit.34196.1.S1_at	94.14648	188.82	-2.00559	AT1G71140.1	2.00E-26	MATE efflux family protein
Cit.8152.1.S1_x_at	33.0704	66.3485	-2.00628	AT4G34050.1	2.00E-94	caffeoyl-CoA 3-O-methyltransferase, putative
Cit.35139.1.S1_at	117.6503	236.688	-2.0118	AT4G35800.1	2.00E-05	NRPB1 (RNA POLYMERASE II LARGE SUBUNIT); DNA binding / DNA-c
Cit.25808.1.S1_at	24.25949	48.8311	-2.01287		NA	
Cit.14966.1.S1_at	79.06924	159.199	-2.01342	AT5G62740.1	1.00E-140	band 7 family protein
Cit.38722.1.S1_at	25.18351	50.7351	-2.01462	AT2G28480.1	1.00E-28	RNA binding
Cit.19619.1.S1_s_at	292.7964	589.947	-2.01487		NA	
Cit.17235.1.S1_s_at	58.36454	117.643	-2.01566	AT1G30100.1	1.00E-69	NCED5 (NINE-CIS-EPOXYCAROTENOID DIOXYGENASE 5); 9-cis-epoxy
Cit.8423.1.S1_s_at	285.174	575.085	-2.01661	AT5G20700.1	2.00E-37	senescence-associated protein-related
Cit.30974.1.S1_at	87.5879	176.711	-2.01753	AT1G12040.1	9.00E-33	LRX1 (LEUCINE-RICH REPEAT/EXTENSIN 1); histidine phosphotransfe
Cit.6481.1.S1_at	63.35734	127.847	-2.01786	AT5G14440.2	3.00E-73	FUNCTIONS IN: molecular_function unknown; INVOLVED IN: biologi
Cit.19388.1.S1_at	27.80922	56.2022	-2.02099		NA	
Cit.28058.1.S1_at	55.21193	111.744	-2.02391	AT4G15320.1	6.00E-17	ATCSLB06 (CELLULOSE SYNTHASE LIKE B6); cellulose synthase/ trans
Cit.8720.1.S1_at	126.2915	255.766	-2.02521	AT5G54160.1	7.00E-35	ATOMT1 (O-METHYLTRANSFERASE 1); caffeate O-methyltransferase
Cit.11854.1.S1_at	66.02227	133.799	-2.02658	AT5G05340.1	1.00E-103	peroxidase, putative
Cit.40475.1.S1_at	199.694	405.068	-2.02844		NA	
Cit.37671.1.S1_at	97.64713	198.216	-2.02992	AT5G15330.1	3.00E-66	SPX4 (SPX DOMAIN GENE 4)
Cit.1020.1.S1_at	53.74604	109.111	-2.03013	AT4G35860.1	1.00E-109	ATGB2 (GTP-BINDING 2); GTP binding
Cit.15198.1.S1_at	35.04058	71.2126	-2.03229	AT5G01510.1	3.00E-22	FUNCTIONS IN: molecular_function unknown; INVOLVED IN: biologi
Cit.21713.1.S1_x_at	40.07437	81.5091	-2.03395		NA	
Cit.12981.1.S1_x_at	777.4709	1582.54	-2.0355	AT2G17230.1	1.00E-149	EXL5 (EXORDIUM LIKE 5)

Cit.18232.1.S1_x_at	21.65692	44.0873	-2.03571		NA	
Cit.24960.1.S1_at	41.9369	85.4503	-2.03759		NA	
Cit.16773.1.S1_at	137.6367	280.639	-2.03898	AT3G55040.1	5.00E-68	GSTL2
Cit.2849.1.S1_at	93.12405	190.064	-2.04098	AT3G20820.1	1.00E-159	leucine-rich repeat family protein
Cit.10123.1.S1_at	20.93156	42.7517	-2.04245	AT5G02560.1	6.00E-35	HTA12; DNA binding
Cit.20082.1.S1_x_at	133.0512	272.329	-2.04679	AT1G75750.1	7.00E-33	GASA1 (GAST1 PROTEIN HOMOLOG 1)
Cit.29120.1.S1_at	23.06486	47.2237	-2.04743	AT2G25737.1	5.00E-24	unknown protein
Cit.19954.1.S1_s_at	2345.066	4813.91	-2.05278		NA	
Cit.4989.1.S1_at	75.90131	155.829	-2.05305		NA	
Cit.4258.1.S1_s_at	263.648	541.335	-2.05325	AT1G14870.1	9.00E-53	FUNCTIONS IN: molecular_function unknown; INVOLVED IN: respon
Cit.15654.1.S1_at	431.6372	887.618	-2.0564	AT2G39220.1	2.00E-36	PLP6 (PATATIN-LIKE PROTEIN 6); nutrient reservoir
Cit.23869.1.S1_at	122.7007	252.749	-2.05988	AT1G59960.1	5.00E-14	aldo/keto reductase, putative
Cit.21331.1.S1_x_at	87.00395	179.309	-2.06093		NA	
Cit.22378.1.S1_x_at	36.81341	75.8903	-2.06148	AT5G19290.1	4.00E-61	esterase/lipase/thioesterase family protein
Cit.12980.1.S1_x_at	1530.819	3157.01	-2.0623	AT2G17230.1	8.00E-79	EXL5 (EXORDIUM LIKE 5)
Cit.8025.1.S1_at	164.0824	338.732	-2.0644	AT1G72020.1	3.00E-34	unknown protein
Cit.5730.1.S1_s_at	32.96659	68.0833	-2.06522		NA	
Cit.24435.1.S1_s_at	43.14742	89.1743	-2.06674	AT5G28237.1	1.00E-106	tryptophan synthase, beta subunit, putative
Cit.25310.1.S1_x_at	259.647	537.432	-2.06986	AT2G46420.1	1.00E-39	unknown protein
Cit.13059.1.S1_s_at	242.7675	502.771	-2.071	AT1G11545.1	1.00E-152	xyloglucan:xyloglucosyl transferase, putative / xyloglucan endotrans
Cit.4748.1.S1_at	33.62225	69.7695	-2.0751		NA	
Cit.15391.1.S1_at	31.6517	65.7175	-2.07627		NA	
Cit.26628.1.S1_at	38.57381	80.1004	-2.07655	AT5G58230.1	7.00E-81	MSI1 (MULTICOPY SUPRESSOR OF IRA1); protein binding
Cit.10009.1.S1_s_at	2736.884	5685.99	-2.07754		NA	
Cit.26586.1.S1_at	37.46225	77.87	-2.07862		NA	
Cit.3768.1.S1_s_at	62.02946	129.285	-2.08424		NA	
Cit.36654.1.S1_at	40.97283	85.6562	-2.09056	AT2G18196.1	2.00E-67	metal ion binding
Cit.5057.1.S1_at	67.1208	140.395	-2.09167	AT1G19150.1	1.00E-114	LHCA6; chlorophyll binding
Cit.21445.1.S1_x_at	486.8617	1018.59	-2.09214		NA	
Cit.29383.1.S1_at	34.94577	73.1566	-2.09343	AT1G01470.1	1.00E-46	LEA14 (LATE EMBRYOGENESIS ABUNDANT 14)
Cit.19884.1.S1_at	31.89212	66.7949	-2.0944	AT5G37810.1	1.00E-14	NIP4;1 (NOD26-LIKE INTRINSIC PROTEIN 4;1); water channel
Cit.22413.1.S1_x_at	76.63734	160.567	-2.09515	AT1G06850.1	6.00E-21	AtbZIP52 (Arabidopsis thaliana basic leucine zipper 52); DNA binding
Cit.14635.1.S1_at	187.3335	392.536	-2.09538	AT1G74210.1	4.00E-69	glycerophosphoryl diester phosphodiesterase family protein
Cit.15773.1.S1_at	41.53218	87.0267	-2.0954		NA	
Cit.17457.1.S1_at	32.22598	67.6863	-2.10036		NA	

Cit.21508.1.S1_x_at	64.16769	134.812	-2.10093		NA	
Cit.8001.1.S1_at	212.1613	445.92	-2.1018	AT1G01360.1	4.00E-74	unknown protein
Cit.24466.1.S1_x_at	20.19087	42.4493	-2.1024	AT5G02500.2	4.00E-16	HSC70-1 (HEAT SHOCK COGNATE PROTEIN 70-1); ATP binding
Cit.18861.1.S1_x_at	192.4578	405.057	-2.10465		NA	
Cit.17531.1.S1_at	32.8157	69.1051	-2.10585		NA	
Cit.6407.1.S1_at	158.6363	334.124	-2.10622	AT5G59030.1	5.00E-44	COPT1 (copper transporter 1); copper ion transmembrane transport
Cit.9343.1.S1_x_at	22.66799	47.7957	-2.10851	AT1G11840.4	3.00E-90	ATGLX1 (GLYOXALASE I HOMOLOG); lactoylglutathione lyase/ metal
Cit.18653.1.S1_at	213.9501	451.134	-2.1086		NA	
Cit.4160.1.S1_at	28.70617	60.5494	-2.10928	AT1G74890.1	2.00E-10	ARR15 (RESPONSE REGULATOR 15); transcription regulator/ two-co
Cit.32450.1.S1_at	151.1833	319.656	-2.11436		NA	
Cit.13924.1.S1_at	22.29439	47.2292	-2.11843	AT1G08550.2	8.00E-92	NPQ1 (NON-PHOTOCHEMICAL QUENCHING 1); violaxanthin de-epo
Cit.20592.1.S1_x_at	282.5285	599.365	-2.12143		NA	
Cit.28962.1.S1_at	28.58522	60.8989	-2.13043	AT4G31840.1	4.00E-48	plastocyanin-like domain-containing protein
Cit.11850.1.S1_at	408.6722	870.83	-2.13088		NA	
Cit.21506.1.S1_s_at	849.145	1811.42	-2.13323		NA	
Cit.6480.1.S1_at	60.67769	129.549	-2.13503	AT5G60210.1	3.00E-65	LOCATED IN: plasma membrane; EXPRESSED IN: 22 plant structures
Cit.15547.1.S1_at	20.32003	43.404	-2.13602	AT4G20970.1	1.00E-37	basic helix-loop-helix (bHLH) family protein
Cit.21592.1.S1_at	66.23438	141.708	-2.13949	AT5G07050.1	2.00E-14	LOCATED IN: membrane; CONTAINS InterPro DOMAIN/s: Protein of
Cit.14512.1.S1_at	27.58935	59.0596	-2.14067		NA	
Cit.8564.1.S1_at	153.9022	329.472	-2.14079	AT5G62350.1	9.00E-38	invertase/pectin methylesterase inhibitor family protein / DC 1.2 ho
Cit.16956.1.S1_x_at	27.17264	58.2635	-2.1442	AT3G15353.1	1.00E-13	MT3 (METALLOTHIONEIN 3); copper ion binding
Cit.24179.1.S1_at	148.8388	319.84	-2.1489	AT3G18850.5	3.00E-81	LPAT5; acyltransferase
Cit.18353.1.S1_x_at	2402.097	5162.79	-2.14928	AT3G15353.1	7.00E-18	MT3 (METALLOTHIONEIN 3); copper ion binding
Cit.14205.1.S1_at	174.9667	376.278	-2.15057	AT5G14450.1	1.00E-161	GDSL-motif lipase/hydrolase family protein
Cit.6094.1.S1_s_at	510.1844	1099.32	-2.15474	AT3G28857.1	1.00E-32	transcription regulator
Cit.30506.1.S1_s_at	806.7209	1738.5	-2.15502	AT5G01600.1	3.00E-92	ATFER1; ferric iron binding / iron ion binding
Cit.4757.1.S1_s_at	319.3107	688.221	-2.15533	AT5G11590.1	7.00E-70	TINY2 (TINY2); DNA binding / transcription factor
Cit.18043.1.S1_x_at	68.24998	147.407	-2.15981	AT1G48380.1	5.00E-30	RHL1 (ROOT HAIRLESS 1); DNA binding / protein binding
Cit.9873.1.S1_at	52.30463	113.125	-2.16281	AT4G32330.3	4.00E-12	FUNCTIONS IN: molecular_function unknown; INVOLVED IN: biologi
Cit.8649.1.S1_x_at	471.1835	1020.07	-2.16492	AT5G39150.1	2.00E-80	germin-like protein, putative
Cit.21222.1.S1_x_at	312.6909	677.025	-2.16516	AT3G15353.1	2.00E-17	MT3 (METALLOTHIONEIN 3); copper ion binding
Cit.1746.1.S1_at	35.00249	75.9291	-2.16925	AT1G08970.2	9.00E-31	NF-YC9 (NUCLEAR FACTOR Y, SUBUNIT C9); DNA binding / transcript
Cit.22410.1.S1_at	27.2928	59.2875	-2.17228	AT1G56220.4	6.00E-12	dormancy/auxin associated family protein
Cit.7736.1.S1_at	390.2705	848.325	-2.17368	AT1G63310.1	1.00E-26	FUNCTIONS IN: molecular_function unknown; INVOLVED IN: biologi
Cit.15140.1.S1_at	37.92609	82.6457	-2.17913	AT5G54690.1	0	GAUT12 (GALACTURONOSYLTRANSFERASE 12); polygalacturonate 4

Cit.31938.1.S1_s_at	34.83338	75.9178	-2.17945	AT5G52030.2	1.00E-107	TraB protein-related
Cit.17714.1.S1_at	36.76332	80.2202	-2.18207	AT4G34350.1	9.00E-13	HDR (4-HYDROXY-3-METHYLBUT-2-ENYL DIPHOSPHATE REDUCTASE
Cit.28809.1.S1_at	31.99292	70.0802	-2.19049	AT1G11440.1	4.00E-21	FUNCTIONS IN: molecular_function unknown; INVOLVED IN: biologi
Cit.31441.1.S1_at	50.87193	111.603	-2.1938		NA	
Cit.24523.1.S1_at	42.88583	94.5021	-2.20357	AT5G05080.2	2.00E-36	UBC22 (ubiquitin-conjugating enzyme 22); ubiquitin-protein ligase
Cit.6704.1.S1_at	26.29988	58.0926	-2.20886	AT5G28237.1	1.00E-106	tryptophan synthase, beta subunit, putative
Cit.1516.1.S1_s_at	42.29675	93.4427	-2.20922	AT1G76010.1	6.00E-93	nucleic acid binding
Cit.23516.1.S1_at	22.79422	50.3744	-2.20996		NA	
Cit.30719.1.S1_at	296.7	655.816	-2.21037		NA	
Cit.19642.1.S1_s_at	244.5903	542.12	-2.21644		NA	
Cit.5883.1.S1_at	28.93802	64.2735	-2.22107	AT1G64950.1	1.00E-128	CYP89A5; electron carrier/ heme binding / iron ion binding / monoo
Cit.14449.1.S1_at	132.5904	295.032	-2.22514	AT2G38310.1	1.00E-69	unknown protein
Cit.3241.1.S1_at	257.3991	574.794	-2.23308		NA	
Cit.22667.1.S1_at	75.40551	168.444	-2.23384	AT2G36800.1	6.00E-67	DOGT1 (DON-GLUCOSYLTRANSFERASE 1); UDP-glycosyltransferase/
Cit.10194.1.S1_x_at	71.59787	159.952	-2.23403	AT1G30910.1	1.00E-123	molybdenum cofactor sulfurase family protein
Cit.18453.1.S1_at	174.957	391.112	-2.23547	AT2G47930.1	2.00E-24	AGP26 (ARABINOGALACTAN PROTEIN 26)
Cit.25382.1.S1_at	39.55682	88.5159	-2.23769		NA	
Cit.29173.1.S1_s_at	22.79854	51.1082	-2.24173	AT3G09270.1	2.00E-49	ATGSTU8 (GLUTATHIONE S-TRANSFERASE TAU 8); glutathione trans
Cit.21499.1.S1_at	250.3827	563.367	-2.25002	ATMG00480.1	4.00E-22	Encodes subunit 8 of the mitochondrial F(O) ATP synthase complex.
Cit.24831.1.S1_at	482.4234	1087.96	-2.2552		NA	
Cit.13308.1.S1_at	21.5751	48.6971	-2.2571	AT5G53060.1	1.00E-29	KH domain-containing protein
Cit.14507.1.S1_at	97.01654	219.039	-2.25774	AT4G38840.1	7.00E-28	auxin-responsive protein, putative
Cit.32755.1.S1_at	95.76399	216.326	-2.25895	ATMG00080.1	1.00E-66	encodes a mitochondrial ribosomal protein L16, which is a constitue
Cit.3283.1.S1_s_at	708.4762	1601.24	-2.26011	AT1G67750.1	0	pectate lyase family protein
Cit.26491.1.S1_at	26.14825	59.1173	-2.26085	AT2G38820.1	1.00E-12	unknown protein
Cit.21389.1.S1_at	246.242	556.975	-2.2619	AT3G20362.1	5.00E-10	unknown protein
Cit.22748.1.S1_x_at	153.4307	347.905	-2.2675	AT2G29250.1	3.00E-11	lectin protein kinase, putative
Cit.19774.1.S1_at	45.55254	103.379	-2.26945		NA	
Cit.7622.1.S1_at	55.95708	127.131	-2.27194	AT1G10000.1	3.00E-11	nucleic acid binding / ribonuclease H
Cit.4757.1.S1_at	476.0845	1081.72	-2.27212	AT5G11590.1	7.00E-70	TINY2 (TINY2); DNA binding / transcription factor
Cit.18912.1.S1_x_at	170.5533	388.814	-2.27972	AT5G39110.1	6.00E-09	germin-like protein, putative
Cit.23000.1.S1_at	20.63573	47.0711	-2.28105	AT1G16670.1	1.00E-31	protein kinase family protein
Cit.28733.1.S1_at	21.69484	49.5424	-2.2836	AT2G41705.2	9.00E-57	camphor resistance CrcB family protein
Cit.8052.1.S1_x_at	20.48099	46.8956	-2.28971	AT5G63620.1	1.00E-177	oxidoreductase, zinc-binding dehydrogenase family protein
Cit.8786.1.S1_at	37.29986	85.5463	-2.29348	AT1G05850.1	1.00E-150	POM1 (POM-POM1); chitinase

Cit.30990.1.S1_at	55.56067	127.465	-2.29415	AT4G15248.1	7.00E-21	zinc ion binding
Cit.6534.1.S1_at	72.82437	167.126	-2.29492	AT1G31770.1	1.00E-91	ABC transporter family protein
Cit.4667.1.S1_at	249.2445	572.308	-2.29617	AT3G11980.1	8.00E-23	MS2 (MALE STERILITY 2); fatty acyl-CoA reductase (alcohol-forming)
Cit.30043.1.S1_at	40.84239	93.9523	-2.30036	AT3G17380.1	1.00E-48	meprin and TRAF homology domain-containing protein / MATH don
Cit.20076.1.S1_x_at	108.5638	250.442	-2.30686		NA	
Cit.4541.1.S1_s_at	186.3775	430.25	-2.30849	AT3G10020.2	6.00E-05	unknown protein
Cit.16027.1.S1_at	24.68781	57.0223	-2.30974		NA	
Cit.21331.1.S1_at	39.49142	91.3314	-2.31269		NA	
Cit.24496.1.S1_at	63.80607	147.749	-2.3156	AT2G37160.2	5.00E-78	transducin family protein / WD-40 repeat family protein
Cit.30421.1.S1_x_at	63.45508	147.049	-2.31737	AT5G47550.1	7.00E-27	cysteine protease inhibitor, putative / cystatin, putative
Cit.36091.1.S1_at	22.03793	51.3864	-2.33173		NA	
Cit.24938.1.S1_at	20.01994	46.7032	-2.33283	AT1G01110.2	3.00E-09	IQD18 (IQ-domain 18)
Cit.24745.1.S1_x_at	39.79363	92.9702	-2.33631	AT5G54960.1	2.00E-07	PDC2 (pyruvate decarboxylase-2); carboxy-lyase/ catalytic/ magnesi
Cit.5108.1.S1_at	34.52201	80.8061	-2.34071		NA	
Cit.32379.1.S1_at	27.28383	64.0022	-2.34579	AT5G38640.1	1.00E-11	eukaryotic translation initiation factor 2B family protein / eIF-2B fan
Cit.18672.1.S1_at	41.044	96.6171	-2.35399		NA	
Cit.17637.1.S1_at	109.9728	259.355	-2.35836	AT3G05890.1	8.00E-16	RCI2B (RARE-COLD-INDUCIBLE 2B)
Cit.3888.1.S1_at	57.74889	136.899	-2.37059	AT3G11910.1	6.00E-65	UBP13 (UBIQUITIN-SPECIFIC PROTEASE 13); ubiquitin thiolesterase/
Cit.24646.1.S1_s_at	445.7212	1057.15	-2.37177	AT1G11545.1	1.00E-152	xyloglucan:xyloglucosyl transferase, putative / xyloglucan endotrans
Cit.19869.1.S1_at	20.23348	48.0311	-2.37384		NA	
Cit.15637.1.S1_at	108.796	258.433	-2.37539	AT4G28780.1	9.00E-91	GDSL-motif lipase/hydrolase family protein
Cit.17921.1.S1_at	25.89457	61.673	-2.3817	AT1G63980.2	1.00E-06	D111/G-patch domain-containing protein
Cit.25177.1.S1_s_at	97.60957	232.602	-2.38298	AT4G09890.1	1.00E-20	unknown protein
Cit.4322.1.S1_at	35.33249	84.2382	-2.38416	AT5G32450.1	1.00E-102	RNA recognition motif (RRM)-containing protein
Cit.24046.1.S1_x_at	35.97584	85.8735	-2.38698	AT1G14900.1	5.00E-50	HMGA (HIGH MOBILITY GROUP A); DNA binding
Cit.21515.1.S1_at	20.25355	48.3718	-2.38831		NA	
Cit.7954.1.S1_at	98.81597	236.056	-2.38884	AT3G01472.1	1.00E-10	CPuORF33 (Conserved peptide upstream open reading frame 33)
Cit.24525.1.S1_s_at	165.4422	395.7	-2.39177	AT5G11590.1	7.00E-70	TINY2 (TINY2); DNA binding / transcription factor
Cit.15644.1.S1_at	29.0134	69.5097	-2.39578	AT1G63440.1	1.00E-108	HMA5 (HEAVY METAL ATPASE 5); ATPase, coupled to transmembrar
Cit.20353.1.S1_s_at	35.15344	84.3521	-2.39954	AT5G55550.2	2.00E-43	RNA recognition motif (RRM)-containing protein
Cit.5513.1.S1_at	60.01215	144.16	-2.40218	AT3G48420.1	2.00E-35	haloacid dehalogenase-like hydrolase family protein
Cit.15006.1.S1_at	88.63542	213.191	-2.40526	AT4G38540.1	2.00E-81	monooxygenase, putative (MO2)
Cit.3613.1.S1_at	142.3636	342.558	-2.40622		NA	
Cit.23228.1.S1_x_at	21.13767	51.1127	-2.41809	AT2G35110.2	1.00E-07	GRL (GNARLED); transcription activator
Cit.7373.1.S1_at	23.29728	56.5706	-2.42821	AT5G60530.1	2.00E-11	late embryogenesis abundant protein-related / LEA protein-related

Cit.1932.1.S1_at	64.96835	158.505	-2.43973	AT1G54690.1	7.00E-33	GAMMA-H2AX (GAMMA HISTONE VARIANT H2AX); DNA binding
Cit.4972.1.S1_s_at	25.77111	62.9496	-2.44264	AT5G07050.1	1.00E-143	LOCATED IN: membrane; CONTAINS InterPro DOMAIN/s: Protein of
Cit.29589.1.S1_at	24.39344	59.6149	-2.44389	AT2G07360.1	4.00E-37	SH3 domain-containing protein
Cit.104.1.S1_at	37.59537	92.2975	-2.45502		NA	
Cit.17784.1.S1_at	57.56379	142.082	-2.46826	AT1G31812.1	3.00E-33	ACBP6 (acyl-CoA-binding protein 6); acyl-CoA binding / phosphatidy
Cit.24017.1.S1_at	21.69091	53.815	-2.48099	AT3G48310.1	2.00E-30	CYP71A22; electron carrier/ heme binding / iron ion binding / mono
Cit.13233.1.S1_s_at	25.23325	62.7005	-2.48484	AT5G23260.2	7.00E-50	TT16 (TRANSPARENT TESTA16); transcription factor
Cit.16174.1.S1_at	86.82906	216.293	-2.49102	AT3G52500.1	3.00E-51	aspartyl protease family protein
Cit.1983.1.S1_x_at	212.1972	528.775	-2.4919	AT5G56670.1	1.00E-29	40S ribosomal protein S30 (RPS30C)
Cit.13169.1.S1_at	26.64709	66.4077	-2.49212		NA	
Cit.13635.1.S1_at	24.31498	60.7278	-2.49755	AT1G13570.1	1.00E-75	F-box family protein
Cit.17907.1.S1_at	242.6278	610.332	-2.51551	AT1G49320.1	3.00E-44	BURP domain-containing protein
Cit.25781.1.S1_at	72.8456	183.449	-2.51833	AT5G17230.3	3.00E-53	phytoene synthase (PSY) / geranylgeranyl-diphosphate geranylgerar
Cit.7643.1.S1_at	20.55983	52.1967	-2.53877		NA	
Cit.25910.1.S1_x_at	542.1643	1377.02	-2.53986		NA	
Cit.26584.1.S1_at	28.53472	72.7621	-2.54995	AT5G16310.1	2.00E-14	UCH1; ubiquitin thiolesterase
Cit.26184.1.S1_at	435.9379	1118.4	-2.56549		NA	
Cit.23904.1.S1_at	53.52273	137.618	-2.57121		NA	
Cit.18341.1.S1_at	110.4908	284.506	-2.57493		NA	
Cit.22350.1.S1_x_at	23.89351	61.5908	-2.57772	AT1G64230.4	5.00E-38	ubiquitin-conjugating enzyme, putative
Cit.6363.1.S1_at	24.51633	63.2169	-2.57856	AT5G16000.1	9.00E-13	NIK1 (NSP-INTERACTING KINASE 1); kinase
Cit.14663.1.S1_s_at	144.9334	374.801	-2.58602	AT3G28050.1	8.00E-12	nodulin MtN21 family protein
Cit.20636.1.S1_at	161.7698	418.805	-2.58889		NA	
Cit.17839.1.S1_at	25.33316	66.0267	-2.60633		NA	
Cit.15604.1.S1_at	39.86253	104.154	-2.61282	AT4G33790.1	2.00E-68	CER4 (ECERIFERUM 4); fatty acyl-CoA reductase (alcohol-forming)/ c
Cit.1843.1.S1_at	118.7353	310.473	-2.61483		NA	
Cit.20157.1.S1_at	29.1801	76.6817	-2.62788		NA	
Cit.24299.1.S1_at	46.59117	124.989	-2.68268	AT1G02260.1	1.00E-54	transmembrane protein, putative
Cit.26152.1.S1_at	31.73182	85.2044	-2.68514		NA	
Cit.15313.1.S1_at	46.40415	125.135	-2.69663	AT4G24050.1	7.00E-67	short-chain dehydrogenase/reductase (SDR) family protein
Cit.29301.1.S1_s_at	65.71637	178.482	-2.71595	AT3G13227.1	5.00E-07	serine-rich protein-related
Cit.35405.1.S1_at	20.50152	55.9239	-2.72779	AT1G18640.2	1.00E-34	PSP (3-PHOSPHOSERINE PHOSPHATASE); phosphoserine phosphata:
Cit.6471.1.S1_at	147.2062	403.691	-2.74235		NA	
Cit.15664.1.S1_at	81.43901	227.617	-2.79494	AT3G11980.1	2.00E-95	MS2 (MALE STERILITY 2); fatty acyl-CoA reductase (alcohol-forming)
Cit.9342.1.S1_x_at	50.92244	142.625	-2.80082	AT1G67280.1	1.00E-167	lactoylglutathione lyase, putative / glyoxalase I, putative

Cit.22275.1.S1_x_at	25.55561	72.3994	-2.83301	AT5G21274.1	1.00E-69	CAM6 (CALMODULIN 6); calcium ion binding
Cit.8149.1.S1_at	574.291	1636.44	-2.84949		NA	
Cit.5342.1.S1_at	42.64472	122.42	-2.8707	AT5G61780.1	1.00E-103	tudor domain-containing protein / nuclease family protein
Cit.30460.1.S1_s_at	53.43396	153.814	-2.87858	AT4G34290.1	7.00E-41	SWIB complex BAF60b domain-containing protein
Cit.7907.1.S1_at	20.00499	58.1401	-2.90628		NA	
Cit.16075.1.S1_at	37.85198	110.127	-2.90941	AT4G24120.1	1.00E-140	YSL1 (YELLOW STRIPE LIKE 1); oligopeptide transporter
Cit.8902.1.S1_s_at	98.16148	287.415	-2.92798	AT1G23740.1	1.00E-124	oxidoreductase, zinc-binding dehydrogenase family protein
Cit.30834.1.S1_at	20.99222	61.87	-2.94728	AT1G20780.1	3.00E-10	SAUL1 (SENESCENCE-ASSOCIATED E3 UBIQUITIN LIGASE 1); ubiquiti
Cit.29223.1.S1_at	27.62486	81.9529	-2.96664	AT5G35200.1	5.00E-07	epsin N-terminal homology (ENTH) domain-containing protein
Cit.29732.1.S1_at	21.3955	64.6621	-3.02223	AT3G12550.1	2.00E-22	XH/XS domain-containing protein / XS zinc finger domain-containin
Cit.29134.1.S1_s_at	117.2983	354.604	-3.02309	AT5G45350.2	5.00E-24	proline-rich family protein
Cit.8866.1.S1_x_at	26.72193	80.7843	-3.02315	AT4G26850.1	0	VTC2 (vitamin c defective 2); GDP-D-glucose phosphorylase/ GDP-g
Cit.37364.1.S1_at	52.29683	158.316	-3.02726	AT3G24670.1	4.00E-48	pectate lyase family protein
Cit.21905.1.S1_s_at	385.0158	1167.43	-3.03215		NA	
Cit.29361.1.S1_s_at	43.91266	135.772	-3.09185		NA	
Cit.12520.1.S1_at	248.056	783.878	-3.16008	AT4G36910.1	5.00E-83	LEJ2 (LOSS OF THE TIMING OF ET AND JA BIOSYNTHESIS 2)
Cit.20410.1.S1_at	34.55122	109.195	-3.16037		NA	
Cit.29791.1.S1_at	320.1819	1012	-3.16069	AT5G11420.1	1.00E-146	FUNCTIONS IN: molecular_function unknown; INVOLVED IN: biologi
Cit.4142.1.S1_at	20.16862	66.7743	-3.3108	AT3G60640.1	5.00E-24	ATG8G (AUTOPHAGY 8G); microtubule binding
Cit.26643.1.S1_at	20.80622	69.183	-3.32511	AT3G14460.1	2.00E-15	disease resistance protein (NBS-LRR class), putative
Cit.36807.1.S1_s_at	28.79765	96.5174	-3.35157	AT4G21200.1	2.00E-74	GA2OX8 (GIBBERELLIN 2-OXIDASE 8); gibberellin 2-beta-dioxygenas
Cit.20076.1.S1_at	87.29588	295.874	-3.38933		NA	
Cit.13787.1.S1_s_at	63.28788	216.915	-3.42743	AT2G03200.1	1.00E-50	aspartyl protease family protein
Cit.25808.1.S1_x_at	29.48284	105.31	-3.57189		NA	
Cit.15073.1.S1_at	64.98821	247.894	-3.81444	AT2G03200.1	6.00E-48	aspartyl protease family protein
Cit.26053.1.S1_x_at	21.51747	85.0484	-3.95253		NA	
Cit.16640.1.S1_at	90.41008	391.046	-4.32525	AT1G55830.1	8.00E-49	unknown protein
Cit.14150.1.S1_s_at	34.72169	157.319	-4.53086	AT5G24860.1	2.00E-33	FPF1 (FLOWERING PROMOTING FACTOR 1)
Cit.16117.1.S1_x_at	23.89378	110.239	-4.61373		NA	
Cit.5977.1.S1_at	170.7289	870.019	-5.09591	AT2G38680.1	1.00E-119	5'-nucleotidase/ magnesium ion binding
Cit.14898.1.S1_s_at	33.0591	176.922	-5.35169	AT5G59030.1	3.00E-35	COPT1 (copper transporter 1); copper ion transmembrane transpor
Cit.8501.1.S1_at	75.54948	483.73	-6.40283		NA	
Cit.21094.1.S1_s_at	96.45015	745.862	-7.73314	AT5G24120.1	9.00E-50	SIGE (SIGMA FACTOR E); DNA binding / DNA-directed RNA polymer
Cit.25990.1.S1_x_at	433.4384	3985.17	-9.19431	AT1G49640.1	5.00E-06	hydrolase
Cit.25990.1.S1_at	167.9719	1851.59	-11.0232	AT1G49640.1	5.00E-06	hydrolase

Cit.12433.1.S1_at	21.43432	494.776	-23.0833	NA
Cit.17596.1.S1_s_at	52.51731	1738.27	-33.099	NA

Supplementary Table 3. PDTA in seedless vs. seedy Fallglo fruits at time point 3.

ProbeSet ID	Fallglo_ Seedless	Fallglo_ Seedy	Ratio	AtGID	E-Score	Arabidopsis.annotation
Cit.10894.1.S1_s_at	298.3677	38.95467	7.659356	AT5G06760.1	1.00E-48	late embryogenesis abundant group 1 domain-containin
Cit.5421.1.S1_at	390.1534	55.99879	6.967176		NA	
Cit.5977.1.S1_at	296.5967	54.87757	5.404698	AT2G38680.1	1.00E-119	5'-nucleotidase/ magnesium ion binding
Cit.11619.1.S1_at	3023.585	576.2541	5.246965	AT1G24020.1	5.00E-10	MLP423 (MLP-LIKE PROTEIN 423)
Cit.5970.1.S1_at	211.0111	41.80473	5.047541	AT5G61430.1	6.00E-92	ANAC100 (ARABIDOPSIS NAC DOMAIN CONTAINING PRO
Cit.9301.1.S1_s_at	223.2093	45.37141	4.919602	AT5G06570.2	3.00E-47	hydrolase
Cit.25808.1.S1_at	629.6157	138.2829	4.553099		NA	
Cit.25808.1.S1_x_at	213.5698	48.82437	4.374246		NA	
Cit.3665.1.S1_at	240.3034	56.87527	4.225095	AT1G75750.1	2.00E-30	GASA1 (GAST1 PROTEIN HOMOLOG 1)
Cit.3665.1.S1_s_at	187.0918	44.30176	4.223123	AT1G75750.1	2.00E-30	GASA1 (GAST1 PROTEIN HOMOLOG 1)
Cit.4748.1.S1_at	114.5434	28.30413	4.046879		NA	
Cit.21990.1.S1_at	81.00927	21.4764	3.772013		NA	
Cit.25990.1.S1_at	2938.464	865.8562	3.393709	AT1G49640.1	5.00E-06	hydrolase
Cit.22916.1.S1_at	100.1817	29.79779	3.362051		NA	
Cit.1799.1.S1_at	84.57984	25.18993	3.357685		NA	
Cit.13586.1.S1_at	140.4493	43.36413	3.238836	AT3G49940.1	2.00E-52	LBD38 (LOB DOMAIN-CONTAINING PROTEIN 38)
Cit.24043.1.S1_at	73.85652	22.81162	3.237671		NA	
Cit.5904.1.S1_at	90.24069	27.88394	3.236296	AT4G28450.1	1.00E-121	nucleotide binding / protein binding
Cit.10673.1.S1_at	159.9013	50.16743	3.187353	AT3G26740.1	4.00E-29	CCL (CCR-LIKE)
Cit.23109.1.S1_x_at	64.13759	20.15693	3.181913	AT5G26570.1	4.00E-14	ATGWD3; carbohydrate kinase/ catalytic/ phosphogluca
Cit.8725.1.S1_at	211.293	66.6151	3.171848	AT5G54160.1	1.00E-105	ATOMT1 (O-METHYLTRANSFERASE 1); caffeate O-methy
Cit.26512.1.S1_at	84.3362	27.0258	3.120581	AT4G11740.1	2.00E-67	SAY1
Cit.21717.1.S1_at	135.7685	43.85246	3.096029	AT3G04720.1	2.00E-43	PR4 (PATHOGENESIS-RELATED 4); chitin binding
Cit.12818.1.S1_at	68.5787	22.34084	3.069656	AT5G48930.1	1.00E-121	HCT (HYDROXYCINNAMOYL-COA SHIKIMATE/QUINATE H
Cit.10425.1.S1_s_at	384.2945	126.9298	3.027614	AT5G05250.1	1.00E-42	unknown protein
Cit.24698.1.S1_x_at	130.885	44.18806	2.961999		NA	
Cit.30428.1.S1_at	213.1775	72.99339	2.920504	AT3G12500.1	7.00E-65	ATHCHIB (ARABIDOPSIS THALIANA BASIC CHITINASE); ch
Cit.23660.1.S1_x_at	63.32249	22.07054	2.869096		NA	
Cit.16993.1.S1_s_at	128.6625	44.87789	2.866946	AT3G55240.1	4.00E-36	Overexpression leads to PEL (Pseudo-Etiolation in Light)
Cit.14399.1.S1_at	67.84486	23.94878	2.832915	AT1G60420.1	2.00E-41	DC1 domain-containing protein
Cit.22186.1.S1_s_at	57.6032	20.43546	2.818787	AT4G34050.1	1.00E-101	caffeoyl-CoA 3-O-methyltransferase, putative

Cit.17596.1.S1_s_at	3419.946	1217.588	2.808788		NA	
Cit.881.1.S1_s_at	91.14725	32.46884	2.807222	AT3G55240.1	4.00E-36	Overexpression leads to PEL (Pseudo-Etiolation in Light)
Cit.31519.1.S1_at	85.54259	30.51175	2.803595		NA	
Cit.26188.1.S1_at	58.548	20.93372	2.796827	AT2G18030.1	3.00E-23	peptide methionine sulfoxide reductase family protein
Cit.1557.1.S1_s_at	864.3521	311.2159	2.777339		NA	
Cit.9620.1.S1_s_at	1418.66	510.9514	2.776507	AT1G61800.1	1.00E-95	GPT2; antiporter/ glucose-6-phosphate transmembrane
Cit.27960.1.S1_at	68.6513	24.85216	2.762388	AT2G13840.1	7.00E-86	PHP domain-containing protein
Cit.23162.1.S1_at	55.7279	20.30198	2.744949	AT3G10150.1	2.00E-32	PAP16 (PURPLE ACID PHOSPHATASE 16); acid phosphata
Cit.3455.1.S1_at	57.7383	21.15661	2.72909	AT2G44950.1	1.00E-128	HUB1 (HISTONE MONO-UBIQUITINATION 1); protein bin
Cit.13589.1.S1_at	87.51889	32.48396	2.694219	AT3G19240.1	1.00E-109	FUNCTIONS IN: molecular_function unknown; INVOLVED
Cit.8959.1.S1_at	186.0094	69.31167	2.683666	AT4G10960.1	1.00E-164	UGE5 (UDP-D-glucose/UDP-D-galactose 4-epimerase 5);
Cit.30401.1.S1_at	62.1197	23.33204	2.66242	AT3G11240.1	3.00E-52	ATE2 (ARGININE-TRNA PROTEIN TRANSFERASE 2); arginy
Cit.6467.1.S1_s_at	53.80452	20.31327	2.648738		NA	
Cit.22763.1.S1_s_at	62.33109	23.94128	2.603499	AT3G23240.1	8.00E-52	ERF1 (ETHYLENE RESPONSE FACTOR 1); DNA binding / tr
Cit.18624.1.S1_at	95.77543	36.80479	2.602254		NA	
Cit.18282.1.S1_s_at	443.9967	171.2393	2.592843		NA	
Cit.12768.1.S1_at	70.27776	27.11527	2.591815	AT2G34790.1	4.00E-75	MEE23 (MATERNAL EFFECT EMBRYO ARREST 23); FAD bi
Cit.20565.1.S1_at	55.74903	21.52959	2.589414		NA	
Cit.36935.1.S1_s_at	90.90368	35.36735	2.570271	AT1G19640.1	2.00E-91	JMT (JASMONIC ACID CARBOXYL METHYLTRANSFERASE)
Cit.29940.1.S1_at	204.5228	79.955	2.557974	AT5G57050.2	1.00E-119	ABI2 (ABA INSENSITIVE 2); protein serine/threonine pho
Cit.3195.1.S1_at	217.6937	85.19434	2.55526	AT1G33055.1	7.00E-13	unknown protein
Cit.32792.1.S1_at	77.05709	30.28122	2.544716		NA	
Cit.15851.1.S1_at	53.80157	21.28973	2.527114	AT1G15780.1	4.00E-29	unknown protein
Cit.9625.1.S1_s_at	940.1227	374.6318	2.509458	AT1G61800.1	1.00E-179	GPT2; antiporter/ glucose-6-phosphate transmembrane
Cit.18469.1.S1_at	89.07269	35.51905	2.507744	AT4G18530.1	2.00E-08	unknown protein
Cit.6136.1.S1_at	117.3111	47.54432	2.467405	AT3G51570.1	3.00E-05	disease resistance protein (TIR-NBS-LRR class), putative
Cit.28780.1.S1_at	52.63288	21.51594	2.446227	AT5G05190.1	1.00E-05	unknown protein
Cit.23649.1.S1_x_at	98.12736	40.15903	2.443469	AT5G56000.1	6.00E-36	heat shock protein 81-4 (HSP81-4)
Cit.16347.1.S1_at	129.1503	52.87402	2.442604	AT2G44190.1	1.00E-45	EDE1 (ENDOSPERM DEFECTIVE 1); microtubule binding
Cit.5281.1.S1_at	56.95779	23.37029	2.437188	AT5G05600.1	2.00E-90	oxidoreductase, 2OG-Fe(II) oxygenase family protein
Cit.29568.1.S1_s_at	96.54282	39.64381	2.435256	AT1G01720.1	1.00E-100	ATAF1; transcription activator/ transcription factor
Cit.26375.1.S1_at	84.47935	34.94588	2.417434	AT3G16730.1	6.00E-48	unknown protein
Cit.10596.1.S1_at	102.5069	42.50489	2.41165	AT1G05170.1	0	galactosyltransferase family protein
Cit.5377.1.S1_at	312.1111	129.7799	2.404926	AT4G37370.1	1.00E-156	CYP81D8; electron carrier/ heme binding / iron ion bindi
Cit.30276.1.S1_at	57.44378	24.06031	2.387491	AT4G37300.1	7.00E-05	MEE59 (maternal effect embryo arrest 59)

Cit.28377.1.S1_at	214.0395	89.71809	2.385689	AT2G36290.1	5.00E-55	hydrolase, alpha/beta fold family protein
Cit.22589.1.S1_s_at	2336.434	982.1132	2.378986	AT2G38870.1	3.00E-18	protease inhibitor, putative
Cit.14216.1.S1_at	64.3288	27.05366	2.377822	AT1G31310.1	2.00E-88	hydroxyproline-rich glycoprotein family protein
Cit.22227.1.S1_at	61.21493	25.74612	2.377637	AT2G43970.2	2.00E-18	La domain-containing protein
Cit.7597.1.S1_at	52.00668	21.90842	2.373822	ATMG00860.1	8.00E-25	hypothetical protein
Cit.27584.1.S1_at	53.0113	22.34536	2.372363	AT2G16365.4	7.00E-23	F-box family protein
Cit.1877.1.S1_at	353.7125	149.204	2.370664		NA	
Cit.9569.1.S1_at	777.7059	328.7005	2.366002		NA	
Cit.29661.1.S1_at	85.45822	36.1447	2.364336	AT4G38460.1	2.00E-34	GGR (geranylgeranyl reductase); farnesyltranstransferas
Cit.22219.1.S1_s_at	154.9826	65.72056	2.358206	AT3G45140.1	0	LOX2 (LIPOXYGENASE 2); lipoxygenase
Cit.15796.1.S1_at	76.69608	32.565	2.355169	AT1G07530.1	2.00E-37	SCL14 (SCARECROW-LIKE 14); transcription factor
Cit.5713.1.S1_at	149.2197	63.43065	2.352486		NA	
Cit.5847.1.S1_a_at	205.898	87.64902	2.349119	AT3G08950.1	2.00E-74	electron transport SCO1/SenC family protein
Cit.28173.1.S1_s_at	68.64628	29.24282	2.347458	AT4G21380.1	1.00E-106	ARK3 (A. THALIANA RECEPTOR KINASE 3); kinase/ transm
Cit.6031.1.S1_at	68.03	29.03817	2.342778	AT2G36380.1	1.00E-101	PDR6; ATPase, coupled to transmembrane movement of
Cit.39750.1.S1_at	115.1715	49.32596	2.334906	AT2G38025.1	4.00E-06	FUNCTIONS IN: molecular_function unknown; INVOLVED
Cit.2351.1.S1_at	92.13165	39.4872	2.333203	AT2G38470.1	1.00E-124	WRKY33; transcription factor
Cit.60.1.S1_at	570.4435	245.7372	2.321356	AT2G38540.1	1.00E-16	LP1; calmodulin binding
Cit.2927.1.S1_s_at	309.7827	133.4617	2.321136	AT5G23810.1	2.00E-96	AAP7; amino acid transmembrane transporter
Cit.39466.1.S1_at	63.80412	27.50443	2.319776		NA	
Cit.17413.1.S1_s_at	96.4454	41.57715	2.319673	AT4G33467.2	1.00E-09	unknown protein
Cit.16807.1.S1_at	265.3294	114.4999	2.317289	AT3G63010.1	9.00E-66	GID1B (GA INSENSITIVE DWARF1B); hydrolase
Cit.38641.1.S1_at	60.77719	26.22995	2.317091	AT2G28070.1	6.00E-19	ABC transporter family protein
Cit.4566.1.S1_at	415.3028	179.2516	2.316871	AT4G37580.1	6.00E-70	HLS1 (HOOKLESS 1); N-acetyltransferase
Cit.8235.1.S1_at	88.68821	38.3022	2.315486	AT1G07890.8	1.00E-124	APX1 (ascorbate peroxidase 1); L-ascorbate peroxidase
Cit.29589.1.S1_at	77.77556	33.6833	2.309024	AT2G07360.1	4.00E-37	SH3 domain-containing protein
Cit.14941.1.S1_at	90.37397	39.16392	2.307582	AT3G49200.1	1.00E-102	unknown protein
Cit.29403.1.S1_x_at	853.4432	371.3413	2.298272	AT4G12300.1	5.00E-58	CYP706A4; electron carrier/ heme binding / iron ion binc
Cit.28815.1.S1_x_at	323.7386	140.8672	2.298183		NA	
Cit.15414.1.S1_at	68.71509	29.90618	2.297689	AT5G61520.1	1.00E-174	hexose transporter, putative
Cit.29073.1.S1_at	46.8532	20.42302	2.294137	AT3G62290.1	9.00E-18	ATARFA1E (ADP-ribosylation factor A1E); GTP binding / p
Cit.14156.1.S1_s_at	192.8673	84.16251	2.291606	AT1G73500.1	1.00E-116	MKK9 (MAP KINASE KINASE 9); MAP kinase kinase/ kinas
Cit.18045.1.S1_s_at	302.0954	132.4115	2.281489	AT1G01250.1	3.00E-26	AP2 domain-containing transcription factor, putative
Cit.12810.1.S1_at	288.3556	128.4285	2.245262	AT1G19670.1	5.00E-65	ATCLH1 (ARABIDOPSIS THALIANA CORONATINE-INDUCEI
Cit.4999.1.S1_s_at	143.3924	63.8951	2.244185	AT4G32480.1	4.00E-47	unknown protein

Cit.16789.1.S1_at	87.46828	39.04992	2.239909		NA	
CitAffx.13.1.S1_at	45.18196	20.23434	2.232935	NA	NA	NA
Cit.19245.1.S1_at	66.81216	29.94604	2.231085		NA	
Cit.3746.1.S1_s_at	535.2963	240.1002	2.22947	AT5G17540.1	1.00E-114	transferase family protein
Cit.20967.1.S1_at	70.22374	31.53876	2.226585		NA	
Cit.31463.1.S1_at	99.0857	44.51091	2.226099		NA	
Cit.34720.1.S1_s_at	101.3897	45.65462	2.220798	AT5G03780.1	5.00E-22	TRFL10 (TRF-LIKE 10); DNA binding
Cit.36604.1.S1_at	70.97865	31.96852	2.220267	AT3G17520.1	2.00E-17	late embryogenesis abundant domain-containing protei
Cit.30249.1.S1_at	85.44483	38.53683	2.217225		NA	
Cit.16640.1.S1_at	277.4091	125.6716	2.207413	AT1G55830.1	8.00E-49	unknown protein
Cit.16349.1.S1_at	44.36123	20.13979	2.202666	AT4G11980.1	9.00E-27	ATNUDX14 (ARABIDOPSIS THALIANA NUDIX HYDROLASE
Cit.18765.1.S1_at	273.2212	124.3053	2.197985		NA	
Cit.31072.1.S1_at	117.0453	53.53279	2.186423	AT3G06880.1	6.00E-07	nucleotide binding
Cit.25016.1.S1_at	45.88271	21.0875	2.175825	AT3G14470.1	3.00E-47	disease resistance protein (NBS-LRR class), putative
Cit.24422.1.S1_at	51.38327	23.63712	2.173838	AT3G18760.1	1.00E-15	ribosomal protein S6 family protein
Cit.17581.1.S1_x_at	117.8526	54.25726	2.172107	AT1G17860.1	1.00E-29	trypsin and protease inhibitor family protein / Kunitz fan
Cit.9990.1.S1_x_at	1401.243	646.1188	2.168708	AT1G33140.1	4.00E-89	PGY2 (PIGGYBACK2); structural constituent of ribosome
Cit.25231.1.S1_at	110.4252	51.08068	2.16178		NA	
Cit.28775.1.S1_s_at	142.1071	65.74548	2.161473		NA	
Cit.30261.1.S1_at	47.2891	21.88292	2.161005	AT4G31390.1	1.00E-176	ABC1 family protein
Cit.15052.1.S1_at	451.9841	209.2071	2.160463	AT3G24800.1	2.00E-97	PRT1 (PROTEOLYSIS 1); ubiquitin-protein ligase
Cit.21962.1.S1_at	101.6362	47.23501	2.151713	AT2G36760.1	8.00E-59	UGT73C2 (UDP-glucosyl transferase 73C2); UDP-glycosyl
Cit.14471.1.S1_at	831.7822	386.7538	2.150676		NA	
Cit.29341.1.S1_at	159.3004	74.1103	2.149504		NA	
Cit.11332.1.S1_at	53.53069	24.9232	2.147826		NA	
Cit.13693.1.S1_s_at	183.1293	85.40961	2.14413	AT4G38960.1	6.00E-28	zinc finger (B-box type) family protein
Cit.18564.1.S1_at	207.0421	96.66221	2.141914		NA	
Cit.14341.1.S1_at	168.0394	78.61142	2.137595		NA	
Cit.9873.1.S1_at	154.7124	72.47871	2.134591	AT4G32330.3	4.00E-12	FUNCTIONS IN: molecular_function unknown; INVOLVEI
Cit.20174.1.S1_x_at	91.32982	42.83715	2.132024		NA	
Cit.14472.1.S1_s_at	848.1997	399.1576	2.124974		NA	
Cit.17552.1.S1_at	43.94083	20.68153	2.124641	AT4G21380.1	1.00E-49	ARK3 (A. THALIANA RECEPTOR KINASE 3); kinase/ transm
Cit.29930.1.S1_at	45.2326	21.29327	2.124267	AT3G09070.1	4.00E-42	glycine-rich protein
Cit.20343.1.S1_x_at	477.3314	224.8256	2.123119		NA	
Cit.30755.1.S1_at	82.36176	38.82247	2.121497	AT5G03740.1	3.00E-27	HD2C (HISTONE DEACETYLASE 2C); histone deacetylase/

Cit.16635.1.S1_at	91.967	43.4368	2.11726	AT3G47180.1	5.00E-42	zinc finger (C3HC4-type RING finger) family protein
Cit.24314.1.S1_s_at	116.0106	55.10602	2.105226	AT2G22840.1	1.00E-114	AtGRF1 (GROWTH-REGULATING FACTOR 1); transcriptio
Cit.11352.1.S1_at	55.11787	26.18323	2.105083	AT5G57990.1	2.00E-17	UBP23 (UBIQUITIN-SPECIFIC PROTEASE 23); ubiquitin thi
Cit.22998.1.S1_s_at	375.3968	178.4651	2.103475	AT2G26070.1	2.00E-95	RTE1 (REVERSION-TO-ETHYLENE SENSITIVITY1)
Cit.20833.1.S1_at	209.9053	99.85054	2.102195	AT3G10950.1	1.00E-06	60S ribosomal protein L37a (RPL37aB)
Cit.21721.1.S1_s_at	121.7791	57.93603	2.101958	AT5G63950.1	1.00E-101	CHR24 (chromatin remodeling 24); ATP binding / DNA bi
Cit.18282.1.S1_at	208.4347	99.40199	2.096887		NA	
Cit.40290.1.S1_at	49.30386	23.51897	2.096344	AT1G60460.3	5.00E-40	unknown protein
Cit.31635.1.S1_at	113.5787	54.21843	2.094836	AT5G27650.1	2.00E-27	PWWP domain-containing protein
Cit.1928.1.S1_s_at	8423.724	4021.398	2.094725	AT1G78860.1	1.00E-117	curculin-like (mannose-binding) lectin family protein
Cit.22649.1.S1_x_at	401.5093	191.806	2.093309	AT4G25150.1	6.00E-50	acid phosphatase, putative
Cit.22602.1.S1_at	77.23795	36.9658	2.089443	AT1G61800.1	3.00E-32	GPT2; antiporter/ glucose-6-phosphate transmembrane
Cit.38606.1.S1_at	84.60279	40.50603	2.088647	AT3G29185.2	1.00E-05	unknown protein
Cit.23787.1.S1_x_at	49.97436	23.9699	2.08488	AT5G44210.1	4.00E-05	ERF9 (ERF DOMAIN PROTEIN 9); DNA binding / transcript
Cit.19626.1.S1_at	80.56424	38.69125	2.082234		NA	
Cit.19987.1.S1_s_at	151.4481	72.78786	2.080678	AT2G17390.1	1.00E-125	AKR2B (ANKYRIN REPEAT-CONTAINING 2B); protein bind
Cit.13694.1.S1_at	334.9966	161.1422	2.078888	AT4G38960.1	6.00E-28	zinc finger (B-box type) family protein
Cit.5457.1.S1_at	501.7426	241.534	2.077317	AT5G06280.3	3.00E-15	unknown protein
Cit.7847.1.S1_a_at	52.17679	25.12691	2.07653		NA	
Cit.13101.1.S1_at	536.8023	258.5606	2.076118	AT3G12685.1	4.00E-52	FUNCTIONS IN: molecular_function unknown; INVOLVED
Cit.7106.1.S1_at	80.61682	38.94263	2.070143	AT5G61520.2	3.00E-60	hexose transporter, putative
Cit.16836.1.S1_at	81.64957	39.44366	2.07003		NA	
Cit.30987.1.S1_at	144.4458	69.96301	2.064602	AT3G51480.1	5.00E-57	ATGLR3.6 (GLUTAMATE RECEPTOR 3.6); intracellular liga
Cit.12400.1.S1_s_at	274.8582	133.1771	2.063855	AT5G60580.4	1.00E-19	zinc finger (C3HC4-type RING finger) family protein
Cit.20040.1.S1_at	152.9192	74.19627	2.061009		NA	
Cit.23571.1.S1_x_at	80.45907	39.11405	2.057038		NA	
Cit.14998.1.S1_at	95.92207	46.6376	2.056754	AT4G03500.1	1.00E-33	ankyrin repeat family protein
Cit.17309.1.S1_at	70.59071	34.32621	2.056467	AT3G03341.1	3.00E-28	unknown protein
Cit.11443.1.S1_at	58.77758	28.59934	2.055208		NA	
Cit.10123.1.S1_at	46.44116	22.6008	2.054846	AT5G02560.1	6.00E-35	HTA12; DNA binding
Cit.8664.1.S1_x_at	294.5928	143.5447	2.052272	AT1G33100.1	2.00E-28	MATE efflux family protein
Cit.10595.1.S1_at	96.07275	46.8232	2.051819		NA	
Cit.5261.1.S1_at	43.71761	21.31228	2.051287	AT3G16300.1	7.00E-47	integral membrane family protein
Cit.26046.1.S1_x_at	86.0248	41.99708	2.048352		NA	
Cit.16049.1.S1_at	50.45692	24.63909	2.04784	AT5G61340.1	8.00E-80	unknown protein

Cit.7235.1.S1_at	408.2847	199.5877	2.045641		NA	
Cit.8877.1.S1_s_at	45.20117	22.14681	2.040979	AT4G01100.1	1.00E-164	ADNT1 (ADENINE NUCLEOTIDE TRANSPORTER 1); ADP tr
Cit.37160.1.S1_at	59.71377	29.28564	2.039012		NA	
Cit.30339.1.S1_at	90.80666	44.59204	2.036387	AT1G76680.1	1.00E-121	OPR1; 12-oxophytodienoate reductase
Cit.13663.1.S1_at	175.9502	86.43759	2.035575	AT2G29290.2	8.00E-90	tropinone reductase, putative / tropine dehydrogenase,
Cit.24776.1.S1_s_at	152.6272	75.09677	2.032407	AT3G15810.1	9.00E-70	unknown protein
Cit.29844.1.S1_at	51.17145	25.1958	2.030952	AT1G04945.2	1.00E-08	FUNCTIONS IN: molecular_function unknown; INVOLVEI
Cit.3807.1.S1_at	427.1867	210.8617	2.025909	AT2G38640.1	2.00E-53	unknown protein
Cit.28003.1.S1_at	50.28567	24.84588	2.023904	AT3G24420.1	8.00E-45	hydrolase, alpha/beta fold family protein
Cit.11731.1.S1_s_at	219.4686	108.6704	2.01958	AT5G26340.1	0	MSS1; carbohydrate transmembrane transporter/ hexos
Cit.38755.1.S1_at	41.91297	20.78736	2.016272	AT5G58890.1	3.00E-16	AGL82 (AGAMOUS-LIKE 82); DNA binding / transcription
Cit.34601.1.S1_at	40.51488	20.10312	2.015353	AT3G44050.1	3.00E-26	kinesin motor protein-related
Cit.28072.1.S1_at	119.5176	59.38559	2.012569	AT5G42800.1	1.00E-05	DFR (DIHYDROFLAVONOL 4-REDUCTASE); dihydrokaemp
Cit.31131.1.S1_at	1958.577	975.1311	2.008527	AT2G37870.1	2.00E-36	protease inhibitor/seed storage/lipid transfer protein (L
Cit.26480.1.S1_at	153.0263	76.21497	2.007825	AT5G65495.1	6.00E-17	unknown protein
Cit.24776.1.S1_at	51.78601	25.85591	2.002869	AT3G15810.1	2.00E-68	unknown protein
Cit.33335.1.S1_s_at	99.38949	198.3818	-2	AT3G22120.1	6.00E-36	CWLP (CELL WALL-PLASMA MEMBRANE LINKER PROTEIN
Cit.21222.1.S1_x_at	1139.664	2280.122	-2.0007	AT3G15353.1	2.00E-17	MT3 (METALLOTHIONEIN 3); copper ion binding
Cit.17840.1.S1_s_at	84.60603	169.4084	-2.00232	AT1G02070.1	2.00E-13	unknown protein
Cit.15833.1.S1_at	82.31422	164.9345	-2.00372		NA	
Cit.30506.1.S1_s_at	1024.711	2053.241	-2.00373	AT5G01600.1	3.00E-92	ATFER1; ferric iron binding / iron ion binding
Cit.12748.1.S1_s_at	186.1328	373.1284	-2.00464	AT4G26530.2	1.00E-172	fructose-bisphosphate aldolase, putative
Cit.34418.1.S1_at	25.76084	51.74744	-2.00876	AT3G22930.1	9.00E-21	calmodulin, putative
Cit.4145.1.S1_at	110.7002	222.446	-2.00945	AT1G43800.1	1.00E-166	acyl-(acyl-carrier-protein) desaturase, putative / stearoy
Cit.21713.1.S1_x_at	77.63784	156.1965	-2.01186		NA	
Cit.26923.1.S1_at	27.74146	55.8945	-2.01484	AT1G12800.1	3.00E-05	S1 RNA-binding domain-containing protein
Cit.30192.1.S1_at	20.59172	41.5117	-2.01594	AT1G21510.1	1.00E-24	unknown protein
Cit.34936.1.S1_at	40.99594	82.71784	-2.01771		NA	
Cit.2304.1.S1_at	32.3175	65.35565	-2.0223	AT2G36090.1	5.00E-11	F-box family protein
Cit.8902.1.S1_s_at	241.7469	489.3136	-2.02407	AT1G23740.1	1.00E-124	oxidoreductase, zinc-binding dehydrogenase family prot
Cit.30768.1.S1_at	25.56077	51.76313	-2.0251	AT2G42880.1	8.00E-30	ATMPK20; MAP kinase
Cit.25956.1.S1_at	45.22975	91.64806	-2.02628	AT1G26690.1	2.00E-08	emp24/gp25L/p24 family protein
Cit.20005.1.S1_at	30.37362	61.57712	-2.02732		NA	
Cit.21033.1.S1_at	99.80601	202.4001	-2.02793	AT5G24580.3	2.00E-24	copper-binding family protein
Cit.15251.1.S1_at	221.136	448.6855	-2.029	AT4G36610.1	2.00E-87	hydrolase, alpha/beta fold family protein

Cit.5745.1.S1_s_at	120.4908	244.6254	-2.03024		NA	
Cit.1727.1.S1_s_at	70.7258	143.8689	-2.03418	AT3G54420.1	6.00E-76	ATEP3; chitinase
Cit.32108.1.S1_at	25.40897	51.69026	-2.03433	AT3G08030.2	2.00E-21	unknown protein
Cit.30024.1.S1_at	247.8607	504.5474	-2.03561	AT1G24020.1	4.00E-06	MLP423 (MLP-LIKE PROTEIN 423)
Cit.7773.1.S1_at	21.3993	43.57521	-2.03629		NA	
Cit.5064.1.S1_at	599.1856	1221.572	-2.03872	AT3G20395.1	4.00E-42	protein binding / zinc ion binding
Cit.4844.1.S1_at	25.1984	51.41991	-2.0406	AT1G04590.1	4.00E-79	FUNCTIONS IN: molecular_function unknown; EXPRESSE
Cit.37130.1.S1_at	20.39753	41.64209	-2.04153		NA	
Cit.4397.1.S1_at	37.15167	75.87012	-2.04217	AT2G44710.1	6.00E-41	RNA recognition motif (RRM)-containing protein
Cit.35180.1.S1_at	89.37003	183.0105	-2.04778	AT5G43822.1	5.00E-41	unknown protein
Cit.13366.1.S1_at	91.42755	187.6169	-2.05208	AT5G13930.1	1.00E-153	TT4 (TRANSPARENT TESTA 4); naringenin-chalcone synth
Cit.22243.1.S1_at	39.72408	81.54769	-2.05285	AT1G18800.1	2.00E-28	NRP2 (NAP1-RELATED PROTEIN 2); DNA binding / chrom
Cit.24823.1.S1_at	22.37615	45.93775	-2.05298	AT3G30390.2	6.00E-11	amino acid transporter family protein
Cit.5159.1.S1_at	32.0924	66.15499	-2.06139	AT1G14310.1	4.00E-11	haloacid dehalogenase-like hydrolase family protein
Cit.38359.1.S1_at	30.18543	62.24848	-2.0622		NA	
Cit.30707.1.S1_s_at	80.40043	166.4806	-2.07064	AT5G50760.1	7.00E-31	auxin-responsive family protein
Cit.5150.1.S1_at	87.18912	180.9163	-2.07499		NA	
Cit.37085.1.S1_at	20.32632	42.22377	-2.0773	AT3G50790.1	8.00E-40	late embryogenesis abundant protein, putative / LEA prc
Cit.4248.1.S1_at	24.32354	50.53661	-2.07768	AT1G06470.2	4.00E-17	phosphate translocator-related
Cit.26433.1.S1_s_at	105.0183	218.2074	-2.0778	AT1G52560.1	4.00E-73	26.5 kDa class I small heat shock protein-like (HSP26.5-P
Cit.13228.1.S1_s_at	689.5103	1434.148	-2.07995	AT4G09960.3	1.00E-94	STK (SEEDSTICK); protein binding / transcription factor
Cit.37295.1.S1_at	30.27877	63.16649	-2.08616	AT5G02390.2	2.00E-07	unknown protein
Cit.25891.1.S1_x_at	20.37548	42.53424	-2.08752	AT2G21660.2	4.00E-49	CCR2 (COLD, CIRCADIAN RHYTHM, AND RNA BINDING 2)
Cit.25187.1.S1_at	43.84501	91.83467	-2.09453	AT1G28400.1	8.00E-28	unknown protein
Cit.31051.1.S1_at	25.24229	53.05951	-2.10201	AT1G18670.1	0	IBS1 (IMPAIRED IN BABA-INDUCED STERILITY 1); ATP bin
Cit.18232.1.S1_x_at	20.03213	42.12865	-2.10305		NA	
Cit.17065.1.S1_at	151.3818	318.9553	-2.10696	AT1G23800.1	2.00E-25	ALDH2B7; 3-chloroallyl aldehyde dehydrogenase/ aldehy
Cit.30937.1.S1_at	44.00079	92.8887	-2.11107	AT1G11200.1	1.00E-134	unknown protein
Cit.40088.1.S1_at	148.5631	313.9916	-2.11352	ATCG00580.1	8.00E-42	PSII cytochrome b559. There have been many speculatic
Cit.4456.1.S1_at	47.94107	101.4671	-2.1165	AT1G65890.1	3.00E-73	AAE12 (ACYL ACTIVATING ENZYME 12); catalytic
Cit.22550.1.S1_s_at	25.9692	54.98144	-2.11718	AT1G03220.1	1.00E-121	extracellular dermal glycoprotein, putative / EDGP, puta
Cit.20668.1.S1_s_at	97.9092	207.7501	-2.12186	AT1G27940.1	1.00E-168	PGP13 (P-GLYCOPROTEIN 13); ATPase, coupled to transn
Cit.2461.1.S1_at	22.55072	47.90762	-2.12444	AT1G54340.1	0	ICDH (ISOCITRATE DEHYDROGENASE); isocitrate dehydrc
Cit.15242.1.S1_at	36.29557	77.15083	-2.12563	AT3G54420.1	2.00E-74	ATEP3; chitinase
Cit.17868.1.S1_s_at	37.60253	79.98579	-2.12714	AT1G30220.1	0	INT2 (INOSITOL TRANSPORTER 2); carbohydrate transme

Cit.3500.1.S1_s_at	76.1303	162.0003	-2.12793	AT5G04500.1	1.00E-05	glycosyltransferase family protein 47
Cit.7247.1.S1_at	105.2637	224.5231	-2.13296	AT1G75950.1	9.00E-15	SKP1 (S PHASE KINASE-ASSOCIATED PROTEIN 1); protein
Cit.3236.1.S1_at	26.39069	56.34812	-2.13515	AT5G39670.1	1.00E-40	calcium-binding EF hand family protein
Cit.29307.1.S1_at	27.12259	57.94769	-2.13651		NA	
Cit.15572.1.S1_at	157.8849	337.5429	-2.1379	AT1G01900.1	1.00E-34	SBTI1.1; serine-type endopeptidase
Cit.8587.1.S1_s_at	54.38065	116.2877	-2.1384		NA	
Cit.10114.1.S1_s_at	1045.23	2243.886	-2.14679	AT5G33370.1	1.00E-149	GDSL-motif lipase/hydrolase family protein
Cit.21441.1.S1_at	465.8814	1000.509	-2.14756	ATCG00160.1	5.00E-68	Chloroplast ribosomal protein S2
Cit.21592.1.S1_at	107.4523	231.0203	-2.14998	AT5G07050.1	2.00E-14	LOCATED IN: membrane; CONTAINS InterPro DOMAIN/s
Cit.14966.1.S1_at	227.3914	488.9143	-2.1501	AT5G62740.1	1.00E-140	band 7 family protein
Cit.39084.1.S1_s_at	163.4794	352.3893	-2.15556	AT5G11160.1	5.00E-84	APT5 (Adenine phosphoribosyltransferase 5); adenine pl
Cit.21573.1.S1_at	60.54574	130.5552	-2.15631		NA	
Cit.38213.1.S1_at	32.29383	69.70094	-2.15834	AT1G30370.1	3.00E-37	lipase class 3 family protein
Cit.29310.1.S1_at	48.83782	105.6388	-2.16305	AT2G38540.1	1.00E-05	LP1; calmodulin binding
Cit.24660.1.S1_at	23.9793	51.87082	-2.16315	AT4G27090.1	4.00E-11	60S ribosomal protein L14 (RPL14B)
Cit.37306.1.S1_at	36.93394	80.02454	-2.16669	AT4G22250.1	2.00E-10	zinc finger (C3HC4-type RING finger) family protein
Cit.19319.1.S1_at	75.71017	164.2668	-2.16968	AT3G49290.2	8.00E-17	ABIL2 (ABL INTERACTOR-LIKE PROTEIN 2)
Cit.1983.1.S1_x_at	128.4387	279.3706	-2.17513	AT5G56670.1	1.00E-29	40S ribosomal protein S30 (RPS30C)
Cit.15014.1.S1_at	21.98385	48.00751	-2.18376	AT5G41370.1	9.00E-41	XPB1 (ARABIDOPSIS HOMOLOG OF XERODERMA PIGMEI
Cit.22187.1.S1_s_at	214.3541	468.4698	-2.18549	AT1G12570.1	1.00E-152	glucose-methanol-choline (GMC) oxidoreductase family
Cit.104.1.S1_at	48.38087	105.8667	-2.18819		NA	
Cit.32755.1.S1_at	61.97183	135.6915	-2.18957	ATMG00080.1	1.00E-66	encodes a mitochondrial ribosomal protein L16, which is
Cit.38319.1.S1_s_at	115.4655	253.0181	-2.19129	AT5G23960.1	1.00E-121	TPS21 (TERPENE SYNTHASE 21); (-)-E-beta-caryophyllene
Cit.7498.1.S1_at	22.94525	50.38227	-2.19576	AT5G22120.1	3.00E-17	unknown protein
Cit.37255.1.S1_at	43.69447	96.0016	-2.19711	AT4G21570.1	3.00E-17	unknown protein
Cit.22978.1.S1_s_at	24.94652	54.88207	-2.19999	AT1G58030.1	6.00E-24	CAT2 (CATIONIC AMINO ACID TRANSPORTER 2); amino a
Cit.8839.1.S1_x_at	58.3483	128.752	-2.20661		NA	
Cit.37016.1.S1_s_at	29.67102	65.47809	-2.2068	AT3G55370.2	2.00E-64	OBP3 (OBF-BINDING PROTEIN 3); DNA binding / transcri
Cit.5613.1.S1_s_at	266.8835	590.3024	-2.21184	AT1G72970.1	2.00E-85	HTH (HOTHEAD); FAD binding / aldehyde-lyase/ mandel
Cit.9029.1.S1_s_at	56.74179	125.5637	-2.2129	AT2G32210.1	1.00E-23	unknown protein
Cit.17366.1.S1_at	50.31164	111.4991	-2.21617		NA	
Cit.15657.1.S1_at	33.49396	74.29166	-2.21806	AT3G50560.1	1.00E-106	short-chain dehydrogenase/reductase (SDR) family prote
Cit.21845.1.S1_x_at	49.44218	109.7912	-2.2206		NA	
Cit.18360.1.S1_s_at	24.51308	54.59152	-2.22704	AT4G11385.1	2.00E-06	unknown protein
Cit.16416.1.S1_at	27.05726	60.27641	-2.22774		NA	

Cit.37098.1.S1_at	24.26232	54.11757	-2.23052		NA	
Cit.29852.1.S1_at	41.38704	92.41728	-2.233	AT5G16030.1	1.00E-49	unknown protein
Cit.19019.1.S1_at	338.7274	760.1751	-2.24421		NA	
Cit.20330.1.S1_at	25.9281	58.27344	-2.2475		NA	
Cit.2700.1.S1_s_at	811.3641	1823.673	-2.24766	AT5G60490.1	1.00E-74	FLA12
Cit.16703.1.S1_at	20.48107	46.09629	-2.25068		NA	
Cit.6286.1.S1_at	20.04429	45.12101	-2.25107	AT4G36230.1	6.00E-11	unknown protein
Cit.30051.1.S1_at	43.96602	98.99416	-2.25161		NA	
Cit.29369.1.S1_x_at	2394.826	5394.6	-2.25261	AT1G72150.1	8.00E-07	PATL1 (PATELLIN 1); transporter
Cit.28249.1.S1_at	50.7024	114.4083	-2.25647	AT1G13360.1	6.00E-22	unknown protein
Cit.21771.1.S1_x_at	25.35816	57.35348	-2.26174	AT5G20650.1	3.00E-11	COPT5; copper ion transmembrane transporter/ high aff
Cit.6534.1.S1_at	126.652	286.5582	-2.26256	AT1G31770.1	1.00E-91	ABC transporter family protein
Cit.190.1.S1_at	23.21269	52.5523	-2.26395	AT5G01150.1	2.00E-05	unknown protein
Cit.9706.1.S1_s_at	137.473	311.269	-2.26422	AT3G57270.1	1.00E-106	BG1 (BETA-1,3-GLUCANASE 1); catalytic/ cation binding /
Cit.30386.1.S1_at	24.45048	55.44747	-2.26775	AT4G20400.2	1.00E-29	transcription factor jumonji (jmj) family protein / zinc fin
Cit.7304.1.S1_at	47.19772	107.0548	-2.26822	AT1G72210.1	1.00E-79	basic helix-loop-helix (bHLH) family protein (bHLH096)
Cit.354.1.S1_at	47.9292	108.7483	-2.26894	AT3G15353.1	1.00E-17	MT3 (METALLOTHIONEIN 3); copper ion binding
Cit.20273.1.S1_at	59.53133	135.0872	-2.26918	AT4G15460.1	1.00E-09	glycine-rich protein
Cit.23140.1.S1_at	22.42127	51.01272	-2.27519		NA	
Cit.36781.1.S1_s_at	220.8591	503.1565	-2.27818	AT3G61430.2	2.00E-23	PIP1A (PLASMA MEMBRANE INTRINSIC PROTEIN 1A); wa
Cit.18706.1.S1_x_at	30.09918	68.78254	-2.2852		NA	
Cit.29361.1.S1_s_at	154.461	353.0255	-2.28553		NA	
Cit.25906.1.S1_at	54.14334	123.8177	-2.28685	AT1G07400.1	3.00E-12	17.8 kDa class I heat shock protein (HSP17.8-CI)
Cit.27344.1.S1_at	32.23609	73.73428	-2.28732	AT5G42905.1	1.00E-27	nucleic acid binding / ribonuclease H
Cit.7687.1.S1_at	52.17725	119.679	-2.2937	AT1G05950.1	2.00E-07	unknown protein
Cit.38354.1.S1_at	39.65627	90.99646	-2.29463	AT4G00110.1	1.00E-31	GAE3 (UDP-D-GLUCURONATE 4-EPIMERASE 3); UDP-gluc
Cit.14962.1.S1_at	27.79998	63.80397	-2.29511	AT5G63020.1	3.00E-87	disease resistance protein (CC-NBS-LRR class), putative
Cit.25747.1.S1_s_at	36.3973	83.56888	-2.29602	AT5G07050.1	1.00E-155	LOCATED IN: membrane; CONTAINS InterPro DOMAIN/s
Cit.4159.1.S1_s_at	47.24868	108.5434	-2.29728	AT1G74890.1	1.00E-55	ARR15 (RESPONSE REGULATOR 15); transcription regulat
Cit.6827.1.S1_x_at	44.43777	102.2511	-2.301	AT1G17010.1	7.00E-81	oxidoreductase, 2OG-Fe(II) oxygenase family protein
Cit.21852.1.S1_s_at	41.1785	95.01566	-2.30741		NA	
Cit.843.1.S1_at	22.75314	52.55042	-2.30959		NA	
Cit.23129.1.S1_at	57.15892	132.319	-2.31493	AT5G66590.1	1.00E-52	allergen V5/Tpx-1-related family protein
Cit.2990.1.S1_x_at	27.58171	64.51961	-2.33922	AT3G49570.1	7.00E-16	LSU3 (RESPONSE TO LOW SULFUR 3)
Cit.13862.1.S1_at	47.4588	111.1461	-2.34195	AT3G12750.1	6.00E-78	ZIP1 (ZINC TRANSPORTER 1 PRECURSOR); zinc ion transn

Cit.16792.1.S1_at	422.0356	994.7507	-2.35703	AT3G57040.1	2.00E-15	ARR9 (RESPONSE REGULATOR 9); transcription regulator
Cit.24458.1.S1_at	27.80925	65.64651	-2.3606	AT4G15560.1	9.00E-44	CLA1 (CLOROPLASTOS ALTERADOS 1); 1-deoxy-D-xylulos
Cit.23844.1.S1_at	46.0097	109.191	-2.37322	AT5G23250.1	2.00E-88	succinyl-CoA ligase (GDP-forming) alpha-chain, mitochor
Cit.6332.1.S1_at	34.25804	81.43658	-2.37715	AT2G34930.1	5.00E-35	disease resistance family protein
Cit.7403.1.S1_at	25.69622	61.15239	-2.37982		NA	
Cit.11284.1.S1_at	23.84999	56.77463	-2.38049	AT3G60900.1	1.00E-56	FLA10
Cit.3339.1.S1_at	215.0646	513.2797	-2.38663	AT1G12570.1	1.00E-152	glucose-methanol-choline (GMC) oxidoreductase family
Cit.21233.1.S1_at	34.58347	82.93658	-2.39816	AT2G31090.1	1.00E-30	unknown protein
Cit.19378.1.S1_s_at	480.2574	1155.014	-2.40499	AT2G02850.1	4.00E-37	ARPN (PLANTACYANIN); copper ion binding / electron ca
Cit.5936.1.S1_at	53.52954	129.4846	-2.41894	AT4G30470.1	1.00E-123	cinnamoyl-CoA reductase-related
Cit.35675.1.S1_at	141.6571	344.6831	-2.43322	AT1G69850.1	1.00E-115	ATNRT1:2 (ARABIDOPSIS THALIANA NITRATE TRANSPOR
Cit.14903.1.S1_s_at	30.37688	74.35428	-2.44773	AT4G11385.1	2.00E-06	unknown protein
Cit.33920.1.S1_at	44.04175	107.8112	-2.44793		NA	
Cit.21651.1.S1_at	20.17426	49.39063	-2.4482		NA	
Cit.7634.1.S1_at	96.76	237.5794	-2.45535		NA	
Cit.17438.1.S1_at	117.4494	288.4868	-2.45626	AT1G14870.1	1.00E-55	FUNCTIONS IN: molecular_function unknown; INVOLVED
Cit.17739.1.S1_at	95.11752	233.7072	-2.45704		NA	
CitAffx.1.1.S1_at	21.94426	53.92881	-2.45754	NA	NA	NA
Cit.38446.1.S1_at	32.82495	80.71364	-2.45891	AT2G25620.1	2.00E-34	protein phosphatase 2C, putative / PP2C, putative
Cit.30628.1.S1_s_at	355.507	877.5291	-2.46839	AT1G69840.6	1.00E-136	band 7 family protein
Cit.26276.1.S1_at	293.7896	725.4938	-2.46943	AT4G21200.1	2.00E-22	GA2OX8 (GIBBERELLIN 2-OXIDASE 8); gibberellin 2-beta-
Cit.36548.1.S1_at	24.50071	60.56338	-2.4719	AT1G62830.1	1.00E-126	LDL1 (LSD1-LIKE1); amine oxidase/ electron carrier/ oxid
Cit.12714.1.S1_at	27.40775	67.87518	-2.4765		NA	
Cit.17344.1.S1_x_at	24.87709	61.98757	-2.49175		NA	
Cit.814.1.S1_s_at	310.9391	775.0035	-2.49246	AT3G01500.3	1.00E-134	CA1 (CARBONIC ANHYDRASE 1); carbonate dehydratase/
Cit.24483.1.S1_s_at	160.825	401.4274	-2.49605	AT4G37870.1	1.00E-164	PCK1 (PHOSPHOENOLPYRUVATE CARBOXYKINASE 1); AT
Cit.16342.1.S1_at	32.06931	80.11947	-2.49832	AT2G01275.2	2.00E-75	zinc finger (C3HC4-type RING finger) family protein
Cit.16532.1.S1_at	24.49003	61.25425	-2.50119		NA	
Cit.21518.1.S1_at	31.90126	79.85211	-2.5031		NA	
Cit.10418.1.S1_x_at	27.11498	68.0463	-2.50955	AT2G23070.1	0	casein kinase II alpha chain, putative
Cit.34392.1.S1_at	127.1706	319.3739	-2.51138	AT5G03190.3	4.00E-08	CPUORF47 (CONSERVED PEPTIDE UPSTREAM OPEN REAI
Cit.3978.1.S1_x_at	21.93539	55.32573	-2.52221	AT5G66030.1	1.00E-31	ATGRIP; protein binding
Cit.7806.1.S1_at	40.39007	102.1428	-2.52891	AT4G35390.1	2.00E-80	AGF1 (AT-hook protein of GA feedback 1); transcription
Cit.11683.1.S1_s_at	193.1622	488.7566	-2.53029	AT2G38110.1	0	GPAT6 (GLYCEROL-3-PHOSPHATE ACYLTRANSFERASE 6);
Cit.16803.1.S1_at	34.40604	87.27704	-2.53668	AT3G23600.1	4.00E-56	dienelactone hydrolase family protein

Cit.23062.1.S1_at	70.61285	181.4222	-2.56925		NA	
Cit.22853.1.S1_x_at	20.21864	52.31543	-2.58749	AT2G46540.1	2.00E-19	unknown protein
Cit.40276.1.S1_at	37.87459	98.85297	-2.61001	AT3G47930.2	4.00E-49	ATGLDH (L-GALACTONO-1,4-LACTONE DEHYDROGENA
Cit.13148.1.S1_at	33.83288	88.61673	-2.61925	AT5G18840.1	2.00E-68	sugar transporter, putative
Cit.8501.1.S1_at	189.0112	496.6339	-2.62754		NA	
Cit.3002.1.S1_s_at	111.7641	293.6819	-2.62769	AT4G12320.1	1.00E-135	CYP706A6; electron carrier/ heme binding / iron ion binc
Cit.233.1.S1_at	687.1501	1820.241	-2.64897		NA	
Cit.21250.1.S1_at	35.15543	94.31898	-2.68291	AT5G39240.1	1.00E-10	unknown protein
Cit.13893.1.S1_at	23.29001	62.72722	-2.69331	AT1G27290.2	2.00E-38	unknown protein
Cit.10547.1.S1_s_at	83.99095	227.7293	-2.71136	AT1G44350.1	5.00E-97	ILL6; IAA-amino acid conjugate hydrolase/ metallopeptic
Cit.16287.1.S1_at	22.07617	59.90311	-2.71347	AT4G39230.1	1.00E-105	isoflavone reductase, putative
Cit.31497.1.S1_at	52.05757	141.4988	-2.71812	AT4G18910.1	3.00E-58	NIP1;2 (NOD26-LIKE INTRINSIC PROTEIN 1;2); arsenite tr
Cit.10687.1.S1_at	25.25059	68.86245	-2.72716	AT2G39700.1	1.00E-131	ATEXPA4 (ARABIDOPSIS THALIANA EXPANSIN A4)
Cit.36807.1.S1_s_at	88.73756	243.1211	-2.73978	AT4G21200.1	2.00E-74	GA2OX8 (GIBBERELLIN 2-OXIDASE 8); gibberellin 2-beta-
Cit.542.1.S1_x_at	30.68239	85.88164	-2.79905	AT3G49540.1	1.00E-08	unknown protein
Cit.23498.1.S1_s_at	51.59502	145.7718	-2.82531	AT3G12750.1	6.00E-78	ZIP1 (ZINC TRANSPORTER 1 PRECURSOR); zinc ion transn
Cit.14797.1.S1_s_at	136.9668	387.9904	-2.83273	AT3G26040.1	2.00E-54	transferase family protein
Cit.17235.1.S1_s_at	45.95026	130.8717	-2.84812	AT1G30100.1	1.00E-69	NCED5 (NINE-CIS-EPOXYCAROTENOID DIOXYGENASE 5);
Cit.8794.1.S1_x_at	21.21352	60.69997	-2.86138	AT1G14320.1	1.00E-46	SAC52 (SUPPRESSOR OF ACAULIS 52); structural constitu
Cit.26358.1.S1_at	21.68583	62.11491	-2.86431	AT2G46640.2	5.00E-21	unknown protein
Cit.8500.1.S1_s_at	1376	3941.588	-2.86453	AT5G09510.1	2.00E-73	40S ribosomal protein S15 (RPS15D)
Cit.28737.1.S1_at	46.01764	132.9187	-2.88843	AT5G13640.1	4.00E-44	ATPDAT; phosphatidylcholine-sterol O-acyltransferase
Cit.27436.1.S1_at	23.26636	67.41367	-2.89747	AT2G27730.1	7.00E-27	unknown protein
Cit.165.1.S1_s_at	158.1567	465.5273	-2.94346	AT3G22840.1	3.00E-56	ELIP1 (EARLY LIGHT-INDUCABLE PROTEIN); chlorophyll b
Cit.21549.1.S1_s_at	149.3214	443.3376	-2.96902		NA	
Cit.29901.1.S1_at	36.83745	111.4011	-3.02413	AT2G39560.1	2.00E-08	unknown protein
Cit.7503.1.S1_at	84.2302	256.9995	-3.05116	AT3G13540.1	5.00E-28	ATMYB5 (MYB DOMAIN PROTEIN 5); DNA binding / trans
Cit.26367.1.S1_at	45.22541	139.6104	-3.08699	AT4G33820.1	4.00E-73	glycosyl hydrolase family 10 protein
Cit.36672.1.S1_s_at	83.01461	257.4972	-3.10183	AT1G65450.1	1.00E-100	transferase family protein
Cit.3126.1.S1_at	29.59279	91.85352	-3.10392	AT5G66730.1	1.00E-42	zinc finger (C2H2 type) family protein
Cit.829.1.S1_at	183.0884	571.0068	-3.11875	AT3G18280.1	2.00E-25	protease inhibitor/seed storage/lipid transfer protein (LT
Cit.20185.1.S1_x_at	21.79117	69.72597	-3.19974		NA	
Cit.26336.1.S1_at	47.86967	153.3163	-3.20279	AT5G49800.1	1.00E-55	FUNCTIONS IN: molecular_function unknown; INVOLVED
Cit.10075.1.S1_at	61.66485	198.8897	-3.22533	AT2G20870.1	3.00E-11	cell wall protein precursor, putative
Cit.15991.1.S1_at	753.9475	2474.707	-3.28233	AT3G53400.1	1.00E-15	FUNCTIONS IN: molecular_function unknown; INVOLVED

Cit.11911.1.S1_s_at	229.2829	755.7	-3.29593	AT1G65450.1	1.00E-100	transferase family protein
Cit.13233.1.S1_s_at	32.55296	109.3997	-3.36067	AT5G23260.2	7.00E-50	TT16 (TRANSPARENT TESTA16); transcription factor
Cit.22753.1.S1_x_at	101.534	345.7697	-3.40546		NA	
Cit.10014.1.S1_s_at	245.2806	862.3901	-3.51593	AT4G10265.1	7.00E-22	wound-responsive protein, putative
Cit.11367.1.S1_x_at	173.9469	653.4698	-3.75672	AT1G69840.6	1.00E-126	band 7 family protein
Cit.12252.1.S1_at	25.5754	96.27971	-3.76454	AT2G14960.1	0	GH3.1
Cit.12090.1.S1_at	88.31	350.0439	-3.96381		NA	
Cit.7483.1.S1_at	83.62318	346.1117	-4.13894		NA	
Cit.11911.1.S1_at	51.76117	219.4174	-4.23903	AT1G65450.1	1.00E-100	transferase family protein
Cit.9461.1.S1_at	34.86281	148.3828	-4.25619	AT1G07400.1	4.00E-63	17.8 kDa class I heat shock protein (HSP17.8-CI)
Cit.9662.1.S1_s_at	87.61066	381.4846	-4.35432	AT5G16050.1	1.00E-126	GRF5 (GENERAL REGULATORY FACTOR 5); ATP binding /
Cit.34778.1.S1_at	31.4138	145.9291	-4.64538	AT5G59190.1	6.00E-32	subtilase family protein
Cit.425.1.S1_s_at	37.92333	177.9445	-4.69222	AT1G22480.1	1.00E-41	plastocyanin-like domain-containing protein
Cit.15355.1.S1_at	65.91405	312.1526	-4.73575	AT4G10490.1	5.00E-80	oxidoreductase, 2OG-Fe(II) oxygenase family protein
Cit.14150.1.S1_s_at	31.25838	157.0553	-5.02442	AT5G24860.1	2.00E-33	FPF1 (FLOWERING PROMOTING FACTOR 1)
Cit.9662.1.S1_at	122.2522	615.3913	-5.03379	AT5G16050.1	1.00E-126	GRF5 (GENERAL REGULATORY FACTOR 5); ATP binding /
Cit.31516.1.S1_at	114.117	605.7681	-5.30831	AT3G53400.1	4.00E-05	FUNCTIONS IN: molecular_function unknown; INVOLVEI
Cit.38702.1.S1_at	29.39074	170.3976	-5.79766	AT3G49260.2	5.00E-23	iqd21 (IQ-domain 21); calmodulin binding
Cit.6130.1.S1_s_at	21.37137	128.7175	-6.02289	AT1G17260.1	5.00E-94	AHA10 (Autoinhibited H(+)-ATPase isoform 10); ATPase/
Cit.13049.1.S1_at	57.80939	423.0659	-7.31829	AT5G62180.1	1.00E-80	AtCXE20 (Arabidopsis thaliana carboxyesterase 20); carb
Cit.21094.1.S1_s_at	201.8176	1535.074	-7.60624	AT5G24120.1	9.00E-50	SIGE (SIGMA FACTOR E); DNA binding / DNA-directed RN
Cit.5112.1.S1_at	36.92023	306.3113	-8.29657	AT1G47128.1	1.00E-118	RD21 (responsive to dehydration 21); cysteine-type endo
Cit.15728.1.S1_at	23.30105	230.5109	-9.89273	AT3G63470.1	6.00E-47	scpl40 (serine carboxypeptidase-like 40); serine-type car
Cit.13787.1.S1_s_at	80.38371	804.7041	-10.0108	AT2G03200.1	1.00E-50	aspartyl protease family protein
Cit.15073.1.S1_at	130.4168	1486.072	-11.3948	AT2G03200.1	6.00E-48	aspartyl protease family protein
Cit.4078.1.S1_at	21.14193	253.4942	-11.9901	AT5G50260.1	1.00E-158	cysteine proteinase, putative
Cit.14555.1.S1_at	22.94328	371.203	-16.1792	AT2G25940.1	1.00E-146	ALPHA-VPE (alpha-vacuolar processing enzyme); cystein

Supplementary Table 4. PDTA in seedless vs. seedy grapefruits at time point 1.

ProbeSet ID	Grapefruit_ Seedless	Grapefruit_ Seedy	Ratio	AtGID	E-Score	Arabidopsis.annotation
Cit.5776.1.S1_at	134.3946	26.91214	4.993828	AT3G10810.1	5.00E-06	zinc finger (C3HC4-type RING finger) family protein
Cit.146.1.S1_x_at	150.5525	30.14889	4.993633		NA	
Cit.7568.1.S1_at	714.4391	157.0501	4.549116	AT5G04010.1	3.00E-07	unknown protein
Cit.6263.1.S1_at	116.6705	26.09958	4.470206	AT1G73090.1	1.00E-104	unknown protein
Cit.29134.1.S1_s_at	103.7085	28.14854	3.68433	AT5G45350.2	5.00E-24	proline-rich family protein
Cit.244.1.S1_x_at	94.43535	25.93742	3.640892	AT5G17920.2	0	ATMS1; 5-methyltetrahydropteroyltriglutamate-homocy
Cit.749.1.S1_x_at	123.3419	34.49959	3.57517	AT1G73230.1	1.00E-69	nascent polypeptide-associated complex (NAC) domain-[illegible]
Cit.13696.1.S1_at	92.3625	25.89022	3.567467	AT5G11810.1	9.00E-85	unknown protein
Cit.28480.1.S1_s_at	1225.071	362.3339	3.381055	AT5G49360.1	1.00E-173	BXL1 (BETA-XYLOSIDASE 1); hydrolase, hydrolyzing O-gly
Cit.17344.1.S1_x_at	77.89981	23.25487	3.349828		NA	
Cit.19443.1.S1_at	139.9188	42.49818	3.292348	AT2G29360.1	1.00E-18	tropinone reductase, putative / tropine dehydrogenase,
Cit.19868.1.S1_x_at	96.34501	30.12198	3.198495	AT2G21660.1	7.00E-63	CCR2 (COLD, CIRCADIAN RHYTHM, AND RNA BINDING 2)
Cit.7941.1.S1_at	424.0673	133.6674	3.172556		NA	
Cit.29626.1.S1_s_at	133.1223	42.43124	3.137365	AT5G50260.1	1.00E-158	cysteine proteinase, putative
Cit.32370.1.S1_at	100.2264	32.96979	3.039947	AT2G20330.1	2.00E-92	transducin family protein / WD-40 repeat family protein
Cit.32673.1.S1_at	62.42619	21.06832	2.963036	AT1G28690.1	2.00E-13	pentatricopeptide (PPR) repeat-containing protein
Cit.26187.1.S1_at	69.10859	23.67328	2.919266		NA	
Cit.935.1.S1_at	159.5643	55.24824	2.888134	AT1G18980.1	9.00E-46	germin-like protein, putative
Cit.35435.1.S1_x_at	95.89341	33.71265	2.844434	AT5G39110.1	6.00E-79	germin-like protein, putative
Cit.13406.1.S1_at	95.05849	33.5962	2.829442	AT5G61380.1	2.00E-28	TOC1 (TIMING OF CAB EXPRESSION 1); transcription regu
Cit.17707.1.S1_x_at	60.16484	21.36341	2.816256	AT1G15270.1	1.00E-28	FUNCTIONS IN: molecular_function unknown; INVOLVED
Cit.23649.1.S1_x_at	64.0662	22.76035	2.814816	AT5G56000.1	6.00E-36	heat shock protein 81-4 (HSP81-4)
Cit.4047.1.S1_at	218.3305	77.61457	2.813009	AT2G02990.1	7.00E-96	RNS1 (RIBONUCLEASE 1); endoribonuclease/ ribonuclea
Cit.23374.1.S1_at	65.25121	23.48979	2.777854	AT1G05500.1	1.00E-56	NTMC2T2.1
Cit.14288.1.S1_at	76.44125	27.5608	2.77355	AT5G06930.1	2.00E-10	LOCATED IN: chloroplast; EXPRESSED IN: 15 plant structu
Cit.24533.1.S1_at	76.2856	27.71767	2.752237		NA	
Cit.33821.1.S1_at	88.31216	33.01671	2.674772	AT5G24970.1	3.00E-12	ABC1 family protein
Cit.17334.1.S1_s_at	585.8802	220.3319	2.65908		NA	
Cit.8769.1.S1_x_at	96.93153	36.50193	2.655518	AT4G16190.1	1.00E-159	cysteine proteinase, putative
Cit.29778.1.S1_at	54.81997	20.66347	2.65299		NA	
Cit.299.1.S1_at	57.93872	22.06332	2.62602		NA	

Cit.32397.1.S1_at	94.15043	36.58663	2.573356	AT2G31260.1	3.00E-09	APG9 (autophagy 9)
Cit.27062.1.S1_at	57.73755	22.45721	2.571003	AT4G25630.1	1.00E-10	FIB2 (FIBRILLARIN 2); snoRNA binding
Cit.16577.1.S1_at	53.04415	20.70791	2.56154		NA	
Cit.29517.1.S1_at	52.10621	20.49529	2.54235		NA	
Cit.5776.1.S1_s_at	218.7956	86.79795	2.520746	AT3G10810.1	5.00E-06	zinc finger (C3HC4-type RING finger) family protein
Cit.11976.1.S1_at	452.3183	180.0242	2.512542	AT1G11680.1	0	CYP51G1 (CYTOCHROME P450 51G1); oxygen binding / s
Cit.29884.1.S1_s_at	116.1352	46.39673	2.50309	AT1G63420.1	7.00E-51	INVOLVED IN: biological_process unknown; EXPRESSED I
Cit.22258.1.S1_at	52.99449	21.21926	2.497471		NA	
Cit.24193.1.S1_at	85.6841	34.6684	2.471533	AT5G51460.1	1.00E-58	ATTPPA; trehalose-phosphatase
Cit.5456.1.S1_at	647.7651	263.5636	2.457718		NA	
Cit.25652.1.S1_at	106.3269	43.27478	2.457018	AT5G40140.1	6.00E-41	armadillo/beta-catenin repeat family protein / U-box do
Cit.26543.1.S1_at	62.32764	25.53979	2.440413	AT3G42170.1	1.00E-05	DNA binding
Cit.38551.1.S1_at	75.85195	31.14682	2.435303	AT2G17900.1	6.00E-95	SDG37; zinc ion binding
Cit.37994.1.S1_at	93.91029	38.70171	2.426515		NA	
Cit.28006.1.S1_at	84.48399	34.90311	2.420529	AT4G03440.1	1.00E-08	ankyrin repeat family protein
Cit.34164.1.S1_at	71.95196	29.75566	2.418093	AT1G69970.2	1.00E-08	CLE26 (CLAVATA3/ESR-RELATED 26); protein binding / re
Cit.23718.1.S1_x_at	70.72135	29.2468	2.418088	AT1G53310.3	6.00E-24	ATPPC1 (PHOSPHOENOLPYRUVATE CARBOXYLASE 1); cat
Cit.30495.1.S1_at	56.5807	23.52774	2.404851	AT2G20320.1	4.00E-38	FUNCTIONS IN: molecular_function unknown; INVOLVED
Cit.31961.1.S1_at	73.42172	30.5805	2.400933		NA	
Cit.31211.1.S1_at	111.5885	46.52837	2.398289		NA	
Cit.21579.1.S1_at	281.9069	118.2636	2.383717	AT2G18328.1	4.00E-09	ATRL4 (ARABIDOPSIS RAD-LIKE 4); DNA binding
Cit.38018.1.S1_at	193.0894	81.04092	2.382616		NA	
Cit.12818.1.S1_at	217.5679	91.33379	2.382118	AT5G48930.1	1.00E-121	HCT (HYDROXYCINNAMOYL-COA SHIKIMATE/QUINATE H
Cit.249.1.S1_at	87.84427	36.93363	2.378436		NA	
Cit.14913.1.S1_at	77.09184	32.42128	2.377816	AT5G24090.1	2.00E-64	acidic endochitinase (CHIB1)
Cit.30987.1.S1_at	87.79913	37.00007	2.372945	AT3G51480.1	5.00E-57	ATGLR3.6 (GLUTAMATE RECEPTOR 3.6); intracellular liga
Cit.13425.1.S1_at	112.9453	47.69796	2.367927	AT2G39730.1	1.00E-169	RCA (RUBISCO ACTIVASE); ADP binding / ATP binding / e
Cit.24519.1.S1_at	63.92334	27.0984	2.358934	AT4G14110.1	1.00E-53	COP9 (CONSTITUTIVE PHOTOMORPHOGENIC 9); metallo
Cit.6070.1.S1_at	139.0768	58.96605	2.358591	AT3G52060.2	1.00E-121	unknown protein
Cit.16254.1.S1_at	81.0616	34.4703	2.351636	AT3G02630.1	5.00E-79	acyl-(acyl-carrier-protein) desaturase, putative / stearoy
Cit.29440.1.S1_at	96.53766	41.17486	2.344578		NA	
Cit.31577.1.S1_at	50.23417	21.43593	2.343457	AT1G52080.1	9.00E-26	AR791; actin binding
Cit.29656.1.S1_at	49.22425	21.03943	2.339619	AT3G50860.1	5.00E-52	clathrin adaptor complex small chain family protein
Cit.33962.1.S1_at	69.2923	29.6387	2.337899	AT3G55060.1	4.00E-56	unknown protein
Cit.30735.1.S1_at	65.99651	28.29733	2.332252		NA	

Cit.38254.1.S1_at	50.52647	21.69903	2.328513	AT3G17850.1	4.00E-19	protein kinase, putative
Cit.36815.1.S1_at	69.84015	30.14142	2.317082	AT1G73177.1	2.00E-14	BNS (BONSAI)
Cit.13914.1.S1_at	99.21288	42.84235	2.315767	AT1G06220.1	1.00E-157	MEE5 (MATERNAL EFFECT EMBRYO ARREST 5); GTP bind
Cit.25665.1.S1_at	61.19453	26.47106	2.311752	AT3G12920.1	1.00E-33	protein binding / zinc ion binding
Cit.20784.1.S1_at	523.8166	229.1577	2.285835		NA	
Cit.21959.1.S1_at	70.40434	30.88287	2.279721	AT3G10920.2	2.00E-23	MSD1 (MANGANESE SUPEROXIDE DISMUTASE 1); metal
Cit.31971.1.S1_at	224.0361	98.29299	2.279268	AT5G06800.1	6.00E-21	myb family transcription factor
Cit.29608.1.S1_at	507.8865	223.1911	2.275568		NA	
Cit.28560.1.S1_at	69.90192	30.78217	2.270857	AT3G14860.2	1.00E-19	NHL repeat-containing protein
Cit.7131.1.S1_at	103.4062	45.65326	2.265034	AT1G65070.1	1.00E-27	DNA mismatch repair MutS family protein
Cit.18374.1.S1_at	209.1514	92.5425	2.260058	AT1G80410.1	6.00E-59	EMB2753 (EMBRYO DEFECTIVE 2753); binding
Cit.6831.1.S1_at	61.02086	27.0018	2.259881	AT1G27940.1	1.00E-168	PGP13 (P-GLYCOPROTEIN 13); ATPase, coupled to transm
Cit.6447.1.S1_at	51.9623	23.05588	2.253755	AT3G48660.1	2.00E-29	unknown protein
Cit.25459.1.S1_at	50.02496	22.23964	2.24936	AT3G10950.1	1.00E-18	60S ribosomal protein L37a (RPL37aB)
Cit.17305.1.S1_at	228.2178	101.4805	2.248883		NA	
Cit.24536.1.S1_at	71.69387	31.94197	2.244504	AT5G53940.1	1.00E-08	yippee family protein
Cit.31428.1.S1_x_at	110.6654	49.60501	2.230932		NA	
Cit.6776.1.S1_at	530.3133	237.7173	2.230857	AT5G51560.1	1.00E-92	leucine-rich repeat transmembrane protein kinase, puta
Cit.20538.1.S1_at	170.8579	76.69138	2.227863	AT3G09270.1	3.00E-09	ATGSTU8 (GLUTATHIONE S-TRANSFERASE TAU 8); glutat
Cit.372.1.S1_s_at	84.65308	38.02269	2.226383	AT5G26600.1	1.00E-176	catalytic/ pyridoxal phosphate binding
Cit.31354.1.S1_at	53.57986	24.09118	2.224045	AT1G20510.1	1.00E-14	OPCL1 (OPC-8:0 COA LIGASE1); 4-coumarate-CoA ligase
Cit.15551.1.S1_at	56.70246	25.50083	2.223554	AT1G10390.2	3.00E-87	nucleoporin family protein
Cit.6208.1.S1_s_at	60.37967	27.17324	2.222027	AT3G02830.1	1.00E-123	ZFN1 (ZINC FINGER PROTEIN 1); DNA binding / nuclease/
Cit.27957.1.S1_x_at	119.394	53.7349	2.221908		NA	
Cit.22980.1.S1_s_at	59.57354	26.81631	2.221541	AT1G37130.1	0	NIA2 (NITRATE REDUCTASE 2); nitrate reductase (NADH)
Cit.21289.1.S1_at	49.63433	22.37598	2.218197		NA	
Cit.23312.1.S1_at	181.8322	82.05581	2.215958	AT3G58690.1	3.00E-59	protein kinase family protein
Cit.39051.1.S1_at	78.9134	35.62772	2.214944	AT2G28250.2	4.00E-81	NCRK; kinase
Cit.11139.1.S1_at	52.27761	23.65016	2.210455	AT5G06700.1	2.00E-76	unknown protein
Cit.30907.1.S1_at	288.0442	130.7215	2.203495	AT4G19800.1	7.00E-62	glycosyl hydrolase family 18 protein
Cit.13063.1.S1_at	178.4125	81.04697	2.201347		NA	
Cit.23550.1.S1_x_at	80.08236	36.43971	2.197667		NA	
Cit.4353.1.S1_at	1411.503	642.7197	2.196141	AT5G09530.1	9.00E-74	hydroxyproline-rich glycoprotein family protein
Cit.25152.1.S1_at	85.06436	38.84875	2.189629	AT4G13980.1	2.00E-38	AT-HSFA5; DNA binding / transcription factor
Cit.17675.1.S1_at	387.1144	177.0444	2.186539	AT1G26250.1	2.00E-29	proline-rich extensin, putative

Cit.19482.1.S1_at	2011.297	921.9777	2.181503	AT1G26250.1	8.00E-28	proline-rich extensin, putative
Cit.15562.1.S1_at	76.27242	34.97451	2.1808	AT3G22800.1	1.00E-138	leucine-rich repeat family protein / extensin family prote
Cit.28524.1.S1_at	83.31639	38.25194	2.178096		NA	
Cit.35759.1.S1_at	76.81609	35.28613	2.176949	AT3G02540.2	3.00E-48	RAD23-3 (PUTATIVE DNA REPAIR PROTEIN RAD23-3)
Cit.15131.1.S1_at	46.94191	21.58613	2.174633		NA	
Cit.36303.1.S1_at	66.97644	30.84276	2.171545	AT2G17210.1	1.00E-73	pentatricopeptide (PPR) repeat-containing protein
Cit.23116.1.S1_at	46.85249	21.62442	2.166647	AT5G53770.1	5.00E-24	nucleotidyltransferase family protein
Cit.28943.1.S1_x_at	60.60776	27.98161	2.165985		NA	
Cit.32885.1.S1_at	44.6911	20.67609	2.161487	AT4G11720.1	1.00E-96	HAP2 (HAPLESS 2)
Cit.24043.1.S1_at	69.23875	32.04109	2.160936		NA	
Cit.18947.1.S1_at	160.4786	74.42439	2.156264		NA	
Cit.17496.1.S1_at	67.08463	31.11854	2.155777		NA	
Cit.24884.1.S1_at	115.2476	53.552	2.152069	AT1G48300.1	1.00E-15	unknown protein
Cit.4589.1.S1_at	71.09136	33.05221	2.150881	AT2G28630.1	0	KCS12 (3-KETOACYL-COA SYNTHASE 12); acyltransferase
Cit.28030.1.S1_at	62.35399	28.99027	2.150859	AT5G49930.1	3.00E-22	emb1441 (embryo defective 1441); nucleic acid binding
Cit.12944.1.S1_s_at	151.0347	70.32094	2.147791	AT5G14570.1	1.00E-179	ATNRT2.7 (Arabidopsis thaliana high affinity nitrate trans
Cit.14245.1.S1_at	111.3718	51.98561	2.142358	AT3G07180.1	1.00E-109	GPI transamidase component PIG-S-related
Cit.24367.1.S1_at	77.22077	36.05005	2.142043	AT4G08170.1	2.00E-14	inositol 1,3,4-trisphosphate 5/6-kinase family protein
Cit.16010.1.S1_at	86.38908	40.39685	2.13851	AT5G44400.1	6.00E-21	FAD-binding domain-containing protein
Cit.31312.1.S1_at	131.4202	61.45601	2.138443	AT1G51965.1	6.00E-88	pentatricopeptide (PPR) repeat-containing protein
Cit.25095.1.S1_at	139.7017	65.42873	2.135174	AT4G39170.1	4.00E-72	SEC14 cytosolic factor, putative / phosphoglyceride trans
Cit.7705.1.S1_at	172.6837	81.03471	2.130984	AT5G53990.1	2.00E-16	glycosyltransferase family protein
Cit.7607.1.S1_at	62.70736	29.4359	2.130302	AT3G29390.1	8.00E-11	RIK (RS2-Interacting KH protein); RNA binding
Cit.23975.1.S1_at	423.7014	199.0817	2.128279	AT1G02090.1	6.00E-07	FUS5 (FUSCA 5); MAP kinase kinase
Cit.21579.1.S1_s_at	980.9236	461.5969	2.125065	AT4G39250.1	4.00E-26	ATRL1 (ARABIDOPSIS RAD-LIKE 1); DNA binding / transcr
Cit.16756.1.S1_s_at	86.74117	40.94624	2.118416	AT3G45140.1	0	LOX2 (LIPOXYGENASE 2); lipoxygenase
Cit.35014.1.S1_s_at	123.4246	58.41771	2.112794	AT5G19130.1	1.00E-84	GPI transamidase component family protein / Gaa1-like
Cit.6728.1.S1_s_at	88.99476	42.12331	2.11272	AT1G56230.1	1.00E-138	unknown protein
Cit.3880.1.S1_at	285.7421	135.6087	2.107107	AT3G62730.1	5.00E-93	unknown protein
Cit.29911.1.S1_at	57.13932	27.15365	2.104296	AT5G40270.1	1.00E-107	metal-dependent phosphohydrolase HD domain-contain
Cit.21697.1.S1_x_at	42.57995	20.24317	2.103423		NA	
Cit.32778.1.S1_at	67.39882	32.08401	2.100698	AT1G77310.1	2.00E-12	FUNCTIONS IN: molecular_function unknown; INVOLVED
Cit.6896.1.S1_at	225.5443	107.4815	2.098448	AT2G30370.1	8.00E-29	allergen-related
Cit.40014.1.S1_at	149.9339	71.58231	2.094566	AT5G27560.1	3.00E-50	unknown protein
Cit.11563.1.S1_at	507.2829	242.3324	2.093335	AT5G37430.1	3.00E-05	unknown protein

Cit.25132.1.S1_at	191.2603	91.53107	2.089567	AT3G16830.1	4.00E-98	TPR2 (TOPLESS-RELATED 2)
Cit.30632.1.S1_at	154.4174	74.03819	2.085645	AT5G27030.1	4.00E-77	TPR3 (TOPLESS-RELATED 3)
Cit.38671.1.S1_at	165.2665	79.40672	2.081266	AT3G10525.1	7.00E-08	FUNCTIONS IN: molecular_function unknown; INVOLVEL
Cit.15039.1.S1_at	187.9516	90.30812	2.081226	AT1G15320.1	1.00E-66	unknown protein
Cit.25527.1.S1_at	81.8309	39.33962	2.080114	AT3G23410.1	1.00E-46	alcohol oxidase-related
Cit.1411.1.S1_at	51.79512	24.92986	2.077634	AT5G52510.1	1.00E-154	scarecrow-like transcription factor 8 (SCL8)
Cit.31076.1.S1_at	67.16113	32.34592	2.07634	AT5G03560.1	2.00E-15	nucleobase:cation symporter
Cit.2172.1.S1_s_at	1127.204	543.4749	2.074068	AT2G25970.1	3.00E-65	KH domain-containing protein
Cit.32970.1.S1_at	50.95372	24.60028	2.071266	AT1G71696.2	2.00E-12	SOL1 (SUPPRESSOR OF LLP1 1); carboxypeptidase/ meta
Cit.24659.1.S1_at	649.4244	313.5852	2.070966	AT3G61220.1	1.00E-54	short-chain dehydrogenase/reductase (SDR) family prote
Cit.29042.1.S1_at	43.13868	20.85056	2.068946	AT1G74640.1	1.00E-94	unknown protein
Cit.19161.1.S1_x_at	55.11288	26.67456	2.066121	AT4G19040.2	8.00E-59	EDR2; lipid binding
Cit.13279.1.S1_at	102.628	49.67988	2.065786	AT2G38410.1	3.00E-37	VHS domain-containing protein / GAT domain-containin
Cit.14237.1.S1_at	54.82683	26.55022	2.065024		NA	
Cit.30272.1.S1_at	47.85926	23.22297	2.060859	AT1G76160.1	1.00E-108	sks5 (SKU5 Similar 5); copper ion binding / oxidoreducta
Cit.38208.1.S1_at	54.85917	26.62239	2.06064		NA	
Cit.31025.1.S1_at	364.0905	176.6893	2.060626	AT4G39250.1	4.00E-26	ATRL1 (ARABIDOPSIS RAD-LIKE 1); DNA binding / transcr
Cit.18258.1.S1_at	164.4199	80.00928	2.05501	AT2G01400.1	3.00E-09	unknown protein
Cit.31891.1.S1_at	61.75165	30.11003	2.050866	AT5G13520.1	6.00E-33	peptidase M1 family protein
Cit.30737.1.S1_at	196.9401	96.03655	2.050679		NA	
Cit.31932.1.S1_at	140.7466	68.63644	2.05061	AT1G26230.1	3.00E-73	chaperonin, putative
Cit.30327.1.S1_at	89.66231	43.76854	2.048556	AT3G06020.1	1.00E-13	unknown protein
Cit.28783.1.S1_at	88.94798	43.47081	2.046154	AT1G67090.1	1.00E-52	RBCS1A (RIBULOSE BISPHOSPHATE CARBOXYLASE SMALI
Cit.29667.1.S1_at	210.8536	103.1523	2.0441	AT5G40270.1	1.00E-58	metal-dependent phosphohydrolase HD domain-contain
Cit.6258.1.S1_x_at	60.19456	29.46505	2.042914		NA	
Cit.7578.1.S1_at	99.84237	48.91213	2.04126	AT1G18980.1	1.00E-71	germin-like protein, putative
Cit.2555.1.S1_at	984.7079	482.5192	2.040764	AT1G48300.1	2.00E-45	unknown protein
Cit.25669.1.S1_x_at	44.08494	21.60876	2.040142	AT4G24270.2	5.00E-28	RNA recognition motif (RRM)-containing protein
Cit.14964.1.S1_at	100.3538	49.21542	2.039072	AT3G49180.1	1.00E-137	RID3 (ROOT INITIATION DEFECTIVE 3); nucleotide bindin
Cit.21378.1.S1_at	226.813	111.391	2.036188		NA	
Cit.33152.1.S1_at	179.0473	88.03311	2.033863	AT3G03710.1	5.00E-06	RIF10 (resistant to inhibition with FSM 10); 3'-5'-exoribo
Cit.190.1.S1_at	125.4958	61.77372	2.03154	AT5G01150.1	2.00E-05	unknown protein
Cit.15783.1.S1_at	77.72437	38.26646	2.031136	AT5G16890.1	1.00E-13	exostosin family protein
Cit.24558.1.S1_at	61.9288	30.50226	2.030302	AT1G43190.2	4.00E-05	polypyrimidine tract-binding protein, putative / heterog
Cit.30262.1.S1_at	104.6471	51.6461	2.026234	AT5G10790.1	7.00E-54	UBP22 (UBIQUITIN-SPECIFIC PROTEASE 22); ubiquitin thi

Cit.32265.1.S1_at	40.55957	20.0274	2.025204		NA	
Cit.30525.1.S1_at	2641.801	1306.306	2.022345	AT2G29350.3	1.00E-51	SAG13; alcohol dehydrogenase/ oxidoreductase
Cit.15776.1.S1_at	221.5951	109.6365	2.02118	AT5G64530.1	5.00E-35	XND1; transcription factor
Cit.28071.1.S1_at	65.81116	32.57379	2.020372	AT5G48380.1	3.00E-54	leucine-rich repeat family protein / protein kinase family
Cit.37335.1.S1_at	72.39626	35.89973	2.016624	AT4G24940.1	3.00E-51	SAE1A (SUMO-ACTIVATING ENZYME 1A); SUMO activatir
Cit.1588.1.S1_at	69.21829	34.32787	2.016388	AT3G49310.1	2.00E-08	LOCATED IN: endomembrane system; EXPRESSED IN: 22
Cit.2476.1.S1_at	234.2576	116.5405	2.010096	AT5G06900.1	1.00E-115	CYP93D1; electron carrier/ heme binding / iron ion bindi
Cit.31724.1.S1_at	127.2168	63.29587	2.009875	AT4G01130.1	4.00E-35	acetylesterase, putative
Cit.23061.1.S1_at	52.69087	26.2162	2.009859		NA	
Cit.36744.1.S1_s_at	69.01195	34.41117	2.00551	AT3G26100.2	1.00E-130	regulator of chromosome condensation (RCC1) family pr
Cit.38530.1.S1_at	43.68871	21.79366	2.004652	AT1G08800.2	6.00E-22	unknown protein
Cit.29644.1.S1_at	41.99894	20.95257	2.004477	AT3G59240.1	2.00E-15	F-box family protein
Cit.32517.1.S1_s_at	108.7044	217.4295	-2.00019	AT5G20190.1	1.00E-49	binding
Cit.30448.1.S1_s_at	45.39168	90.82381	-2.00089	AT3G01990.1	9.00E-66	ACR6; amino acid binding
Cit.19616.1.S1_s_at	33.64722	67.36195	-2.00201	AT5G58800.2	1.00E-87	quinone reductase family protein
Cit.2927.1.S1_s_at	1722.503	3452.053	-2.00409	AT5G23810.1	2.00E-96	AAP7; amino acid transmembrane transporter
Cit.16390.1.S1_at	41.82342	83.85966	-2.00509	AT3G56230.1	2.00E-59	speckle-type POZ protein-related
Cit.1870.1.S1_s_at	69.2896	139.0628	-2.00698	AT2G17880.1	3.00E-24	DNAJ heat shock protein, putative
Cit.30648.1.S1_s_at	349.1571	700.8607	-2.00729	AT4G27670.1	2.00E-32	HSP21 (HEAT SHOCK PROTEIN 21)
Cit.9944.1.S1_x_at	21.02978	42.21932	-2.0076	AT4G37990.1	1.00E-156	ELI3-2 (ELICITOR-ACTIVATED GENE 3-2); aryl-alcohol deh
Cit.11806.1.S1_at	76.17902	153.4273	-2.01404	AT2G31050.1	4.00E-26	plastocyanin-like domain-containing protein
Cit.14643.1.S1_at	42.59464	85.81405	-2.01467	AT4G23496.1	9.00E-26	SP1L5 (SPIRAL1-LIKE5)
Cit.9817.1.S1_at	325.5875	657.2968	-2.0188		NA	
Cit.27939.1.S1_at	23.82326	48.11109	-2.0195	AT3G01180.1	1.00E-06	AtSS2 (starch synthase 2); transferase, transferring glyco
Cit.29034.1.S1_at	21.67846	43.80836	-2.02082	AT3G13960.1	1.00E-10	AtGRF5 (GROWTH-REGULATING FACTOR 5); transcriptio
Cit.6160.1.S1_at	41.21016	83.29795	-2.0213	AT2G32510.1	2.00E-86	MAPKKK17; ATP binding / kinase/ protein kinase/ protei
Cit.5575.1.S1_at	243.6056	493.3335	-2.02513	AT3G50170.1	9.00E-50	unknown protein
Cit.4945.1.S1_at	30.18844	61.17122	-2.02631	AT4G31200.1	3.00E-82	SWAP (Suppressor-of-White-APricot)/surp domain-conta
Cit.12252.1.S1_at	732.8117	1486.42	-2.02838	AT2G14960.1	0	GH3.1
Cit.1869.1.S1_at	2042.184	4144.768	-2.02958		NA	
Cit.22186.1.S1_s_at	40.72239	82.67322	-2.03017	AT4G34050.1	1.00E-101	caffeoyl-CoA 3-O-methyltransferase, putative
Cit.10749.1.S1_at	24.56255	49.9003	-2.03156	AT3G16520.3	1.00E-114	UDP-glucoronosyl/UDP-glucosyl transferase family prote
Cit.24469.1.S1_at	21.14807	42.97448	-2.03208	AT3G05700.1	9.00E-09	INVOLVED IN: response to water deprivation; CONTAINS
Cit.15404.1.S1_at	31.41311	63.85343	-2.0327	AT4G33720.1	2.00E-58	pathogenesis-related protein, putative
Cit.25795.1.S1_s_at	1985.367	4037.606	-2.03368	AT3G57520.1	0	AtSIP2 (Arabidopsis thaliana seed imbibition 2); hydrolas

Cit.26276.1.S1_at	56.59858	115.185	-2.03512	AT4G21200.1	2.00E-22	GA2OX8 (GIBBERELLIN 2-OXIDASE 8); gibberellin 2-beta-
Cit.29115.1.S1_at	25.5071	51.91818	-2.03544	AT5G12380.1	1.00E-24	annexin, putative
Cit.30366.1.S1_at	22.1095	45.0193	-2.0362		NA	
Cit.1779.1.S1_at	117.3429	238.9451	-2.0363	AT2G29500.1	4.00E-59	17.6 kDa class I small heat shock protein (HSP17.6B-CI)
Cit.20532.1.S1_at	54.11301	110.225	-2.03694	AT4G35985.1	3.00E-18	senescence/dehydration-associated protein-related
Cit.30921.1.S1_at	45.00817	91.68755	-2.03713		NA	
Cit.30233.1.S1_at	29.68463	60.47831	-2.03736		NA	
Cit.9379.1.S1_x_at	22.07842	45.01545	-2.03889	AT5G14040.1	1.00E-169	mitochondrial phosphate transporter
Cit.29674.1.S1_s_at	172.3675	351.5543	-2.03956	AT5G24890.1	1.00E-33	unknown protein
Cit.9568.1.S1_s_at	5473.95	11177.1	-2.04187	AT5G64260.1	1.00E-125	EXL2 (EXORDIUM LIKE 2)
Cit.12140.1.S1_at	665.1412	1358.439	-2.04233	AT3G08860.1	3.00E-98	alanine--glyoxylate aminotransferase, putative / beta-ala
Cit.38593.1.S1_at	24.57696	50.19565	-2.04239	AT4G19440.1	1.00E-40	pentatricopeptide (PPR) repeat-containing protein
Cit.7887.1.S1_at	24.74667	50.59133	-2.04437	AT5G65430.2	2.00E-14	GRF8 (GENERAL REGULATORY FACTOR 8); protein bindin
Cit.30380.1.S1_at	36.48226	74.61391	-2.04521	AT3G18950.1	2.00E-80	transducin family protein / WD-40 repeat family protein
Cit.753.1.S1_x_at	2145.452	4390.267	-2.04631	AT3G04720.1	1.00E-46	PR4 (PATHOGENESIS-RELATED 4); chitin binding
Cit.7004.1.S1_at	29.22086	59.80096	-2.04652	AT5G19460.1	7.00E-14	atnudt20 (Arabidopsis thaliana Nudix hydrolase homolog
Cit.31341.1.S1_at	39.00084	79.85172	-2.04744	AT2G29420.1	1.00E-32	ATGSTU7 (ARABIDOPSIS THALIANA GLUTATHIONE S-TRA
Cit.23166.1.S1_x_at	26.51109	54.30925	-2.04855	AT2G38920.2	3.00E-08	SPX (SYG1/Pho81/XPR1) domain-containing protein / zin
Cit.34999.1.S1_at	54.1011	110.8753	-2.04941		NA	
Cit.15868.1.S1_at	26.53033	54.38554	-2.04994	AT1G30840.1	1.00E-126	ATPUP4; purine transmembrane transporter
Cit.26534.1.S1_s_at	240.5442	493.7165	-2.0525	AT3G21690.1	6.00E-64	MATE efflux family protein
Cit.17228.1.S1_x_at	34.75286	71.35558	-2.05323	AT4G34135.1	1.00E-131	UGT73B2 (UDP-GLUCOSYLTRANSFERASE 73B2); UDP-glu
Cit.19110.1.S1_at	32.36997	66.46707	-2.05336	AT1G09630.1	6.00E-06	ATRAB11C (ARABIDOPSIS RAB GTPASE 11C); GTP binding
Cit.3757.1.S1_at	145.3966	298.5517	-2.05336	AT2G46400.1	2.00E-06	WRKY46; transcription factor
Cit.16703.1.S1_at	28.00309	57.61864	-2.05758		NA	
Cit.30790.1.S1_at	37.77085	77.77808	-2.05921	AT1G70180.2	6.00E-31	sterile alpha motif (SAM) domain-containing protein
Cit.4238.1.S1_at	389.9166	803.2291	-2.06	AT1G78110.1	5.00E-94	unknown protein
Cit.23712.1.S1_x_at	32.46099	66.88322	-2.06042	AT1G17860.1	6.00E-12	trypsin and protease inhibitor family protein / Kunitz fan
Cit.21816.1.S1_at	21.24838	43.78094	-2.06044		NA	
Cit.10496.1.S1_at	96.95839	200.1721	-2.06452	AT1G30380.1	3.00E-53	PSAK (photosystem I subunit K)
Cit.10894.1.S1_x_at	5709.248	11790.02	-2.06507		NA	
Cit.22423.1.S1_x_at	461.0745	953.0154	-2.06694	AT1G54410.1	3.00E-15	dehydrin family protein
Cit.9523.1.S1_s_at	1076.829	2225.784	-2.06698	AT2G47770.1	6.00E-43	benzodiazepine receptor-related
Cit.35082.1.S1_at	44.45983	91.95115	-2.06818	AT1G09820.1	4.00E-74	pentatricopeptide (PPR) repeat-containing protein
Cit.13868.1.S1_at	79.21143	163.8576	-2.06861	AT4G14550.1	3.00E-81	IAA14 (INDOLE-3-ACETIC ACID INDUCIBLE 14); protein bi

Cit.30410.1.S1_at	30.06585	62.21368	-2.06925	AT1G28330.4	2.00E-30	DYL1 (DORMANCY-ASSOCIATED PROTEIN-LIKE 1)
Cit.1270.1.S1_s_at	196.4228	406.5544	-2.06979	AT5G61600.1	4.00E-45	ethylene-responsive element-binding family protein
Cit.17868.1.S1_at	233.537	483.4109	-2.06995	AT1G30220.1	7.00E-75	INT2 (INOSITOL TRANSPORTER 2); carbohydrate transme
Cit.7113.1.S1_at	20.68918	42.90475	-2.07378	AT3G18930.2	3.00E-84	zinc finger (C3HC4-type RING finger) family protein
Cit.19849.1.S1_at	55.46238	115.0973	-2.07523	AT5G15380.1	2.00E-07	DRM1 (domains rearranged methylase 1); DNA (cytosine
Cit.31478.1.S1_at	29.90771	62.09716	-2.07629	AT2G04039.1	1.00E-49	unknown protein
Cit.23133.1.S1_at	22.40052	46.52995	-2.07718	AT4G39630.1	3.00E-14	unknown protein
Cit.7063.1.S1_at	28.28908	58.76971	-2.07747	AT2G43465.1	2.00E-16	FUNCTIONS IN: molecular_function unknown; INVOLVED
Cit.29998.1.S1_at	202.2535	420.3799	-2.07848	AT3G63470.1	1.00E-88	scpl40 (serine carboxypeptidase-like 40); serine-type car
Cit.8683.1.S1_s_at	405.0672	841.9821	-2.07862	AT2G16060.1	4.00E-66	AHB1 (ARABIDOPSIS HEMOGLOBIN 1); oxygen binding /
Cit.22158.1.S1_x_at	186.9249	388.5839	-2.07882	AT1G17860.1	3.00E-12	trypsin and protease inhibitor family protein / Kunitz fan
Cit.15391.1.S1_at	36.39828	75.73123	-2.08063		NA	
Cit.28162.1.S1_at	62.85079	131.0723	-2.08545	AT1G17020.1	4.00E-63	SRG1 (SENESCENCE-RELATED GENE 1); oxidoreductase, a
Cit.21485.1.S1_at	156.3107	326.6342	-2.08965		NA	
Cit.24389.1.S1_at	28.05105	58.63401	-2.09026	AT5G17620.1	9.00E-13	FUNCTIONS IN: molecular_function unknown; INVOLVED
Cit.6374.1.S1_at	34.17477	71.43747	-2.09036	AT5G20080.1	1.00E-142	NADH-cytochrome b5 reductase, putative
Cit.11107.1.S1_at	40.30638	84.26814	-2.09069	AT3G25290.2	1.00E-142	auxin-responsive family protein
Cit.48.1.S1_x_at	32.09343	67.10921	-2.09106		NA	
Cit.6217.1.S1_at	29.22892	61.13298	-2.09152	AT5G14120.1	1.00E-107	nodulin family protein
Cit.34850.1.S1_s_at	1029.275	2152.896	-2.09166	AT3G47420.1	0	glycerol-3-phosphate transporter, putative / glycerol 3-p
Cit.17090.1.S1_s_at	543.3516	1136.719	-2.09205	AT1G69490.1	4.00E-89	NAP (NAC-like, activated by AP3/PI); transcription factor
Cit.11795.1.S1_at	30.34127	63.55022	-2.09451	AT3G22750.1	4.00E-84	protein kinase, putative
Cit.16554.1.S1_at	62.8737	131.8316	-2.09677	AT1G28050.1	8.00E-17	zinc finger (B-box type) family protein
Cit.20780.1.S1_at	31.2411	65.5246	-2.09738		NA	
Cit.13579.1.S1_s_at	419.2002	879.8401	-2.09885	AT5G65660.1	3.00E-48	hydroxyproline-rich glycoprotein family protein
Cit.7969.1.S1_x_at	193.9831	407.1827	-2.09906	AT1G17860.1	8.00E-31	trypsin and protease inhibitor family protein / Kunitz fan
Cit.39345.1.S1_at	36.94945	77.57458	-2.09948		NA	
Cit.2848.1.S1_s_at	1138.955	2391.585	-2.09981	AT3G20820.1	1.00E-159	leucine-rich repeat family protein
Cit.25504.1.S1_at	24.44887	51.34602	-2.10014	AT4G36740.1	1.00E-06	ATHB40 (ARABIDOPSIS THALIANA HOMEOBOX PROTEIN
Cit.19678.1.S1_s_at	674.624	1417.144	-2.10064	AT2G27830.1	2.00E-48	FUNCTIONS IN: molecular_function unknown; EXPRESSE
Cit.20219.1.S1_at	33.50495	70.39866	-2.10114		NA	
Cit.35862.1.S1_at	81.23285	171.1879	-2.10737	AT5G04220.1	1.00E-64	ATSYTC/NTMC2T1.3/NTMC2TYPE1.3/SYTC
Cit.5733.1.S1_at	43.35767	91.37954	-2.10757	AT2G31180.1	5.00E-56	MYB14 (MYB DOMAIN PROTEIN 14); DNA binding / trans
Cit.445.1.S1_s_at	74.59413	157.7687	-2.11503	AT5G01300.1	3.00E-69	phosphatidylethanolamine-binding family protein
Cit.2305.1.S1_s_at	659.626	1395.787	-2.11603	AT2G36090.1	3.00E-53	F-box family protein

Cit.13512.1.S1_at	225.8261	478.042	-2.11686	AT5G20790.1	1.00E-25	unknown protein
Cit.11961.1.S1_at	23.5888	49.95333	-2.11767	AT4G38260.1	9.00E-36	unknown protein
Cit.30560.1.S1_at	51.63482	109.4949	-2.12056	AT1G32928.1	7.00E-08	unknown protein
Cit.28604.1.S1_at	28.41939	60.30857	-2.12209		NA	
Cit.27992.1.S1_s_at	32.68456	69.40783	-2.12357	AT3G26040.1	1.00E-66	transferase family protein
Cit.35939.1.S1_at	20.71055	43.99347	-2.12421	AT4G11970.2	1.00E-61	YT521-B-like family protein
Cit.24724.1.S1_x_at	23.84811	50.71366	-2.12653		NA	
Cit.17404.1.S1_at	29.99946	64.04072	-2.13473		NA	
Cit.12090.1.S1_at	307.6864	656.8776	-2.13489		NA	
Cit.3086.1.S1_at	352.916	754.1787	-2.13699	AT1G68320.1	2.00E-63	MYB62 (myb domain protein 62); DNA binding / transcri
Cit.20225.1.S1_at	289.0576	617.9716	-2.13788	AT1G07750.1	6.00E-14	cupin family protein
Cit.1983.1.S1_x_at	256.8926	549.68	-2.13973	AT5G56670.1	1.00E-29	40S ribosomal protein S30 (RPS30C)
Cit.12814.1.S1_at	486.3869	1041.768	-2.14185	AT5G07330.1	2.00E-36	unknown protein
Cit.12814.1.S1_s_at	533.9671	1143.976	-2.14241	AT5G07330.1	2.00E-36	unknown protein
Cit.14741.1.S1_at	25.48604	54.60696	-2.14262	AT4G05320.4	7.00E-30	UBQ10 (POLYUBIQUITIN 10); protein binding
Cit.2690.1.S1_s_at	44.99681	96.44894	-2.14346	AT4G20820.1	1.00E-151	FAD-binding domain-containing protein
Cit.24046.1.S1_x_at	87.03015	186.587	-2.14394	AT1G14900.1	5.00E-50	HMGA (HIGH MOBILITY GROUP A); DNA binding
Cit.27139.1.S1_s_at	22.12514	47.46975	-2.14551	AT5G65210.5	1.00E-144	TGA1; DNA binding / calmodulin binding / transcription f
Cit.9817.1.S1_s_at	2045.599	4391.904	-2.147	AT3G07910.1	3.00E-29	FUNCTIONS IN: molecular_function unknown; INVOLVED
Cit.27634.1.S1_x_at	20.34644	43.70661	-2.14812	AT1G34110.1	3.00E-47	leucine-rich repeat transmembrane protein kinase, puta
Cit.31355.1.S1_at	34.14619	73.38448	-2.14913		NA	
Cit.22600.1.S1_at	49.68618	106.8737	-2.15097	AT1G55490.2	1.00E-48	CPN60B (CHAPERONIN 60 BETA); ATP binding / protein b
Cit.22046.1.S1_at	32.35443	69.62057	-2.15181		NA	
Cit.36556.1.S1_s_at	63.51652	136.7012	-2.15221		NA	
Cit.15744.1.S1_at	41.08154	88.46658	-2.15344		NA	
Cit.30385.1.S1_at	28.28743	61.00263	-2.15653	AT5G15700.1	3.00E-84	DNA-directed RNA polymerase (RPOT2)
Cit.22463.1.S1_s_at	947.9744	2046.62	-2.15894	AT4G27410.2	1.00E-110	RD26 (RESPONSIVE TO DESICCATION 26); transcription a
Cit.1816.1.S1_s_at	100.8887	218.1618	-2.1624	AT5G38410.1	4.00E-49	ribulose bisphosphate carboxylase small chain 3B / RuBis
Cit.28045.1.S1_at	74.84228	161.9174	-2.16345	AT5G16620.1	2.00E-61	TIC40
Cit.28614.1.S1_at	34.27032	74.18339	-2.16465	AT2G21520.2	1.00E-08	transporter
Cit.17868.1.S1_s_at	114.5638	247.999	-2.16472	AT1G30220.1	0	INT2 (INOSITOL TRANSPORTER 2); carbohydrate transme
Cit.36282.1.S1_at	24.09928	52.25077	-2.16815		NA	
Cit.23912.1.S1_s_at	54.38701	117.9489	-2.1687	AT5G59730.1	1.00E-54	ATEXO70H7 (EXOCYST SUBUNIT EXO70 FAMILY PROTEIN
Cit.14618.1.S1_at	23.60082	51.30586	-2.1739	AT5G47670.1	1.00E-55	NF-YB6 (NUCLEAR FACTOR Y, SUBUNIT B6); transcription
Cit.1883.1.S1_at	26.16536	56.88403	-2.17402	AT4G35160.1	5.00E-45	O-methyltransferase family 2 protein

Cit.9462.1.S1_x_at	284.5043	619.3627	-2.17699	AT1G07400.1	2.00E-62	17.8 kDa class I heat shock protein (HSP17.8-CI)
Cit.11683.1.S1_s_at	29.77991	64.85222	-2.17772	AT2G38110.1	0	GPAT6 (GLYCEROL-3-PHOSPHATE ACYLTRANSFERASE 6);
Cit.2868.1.S1_at	81.31615	177.1144	-2.1781	AT1G29950.2	3.00E-27	transcription factor/ transcription regulator
Cit.8718.1.S1_s_at	22.99694	50.0919	-2.1782	AT5G54160.1	1.00E-105	ATOMT1 (O-METHYLTRANSFERASE 1); caffeate O-methy
Cit.33490.1.S1_at	58.59881	127.6799	-2.17888	AT5G59450.1	6.00E-82	scarecrow-like transcription factor 11 (SCL11)
Cit.3448.1.S1_s_at	1101.3	2402.589	-2.18159	AT2G38120.1	1.00E-173	AUX1 (AUXIN RESISTANT 1); amino acid transmembrane
Cit.26965.1.S1_at	38.46945	84.08906	-2.18587	AT1G64230.4	3.00E-52	ubiquitin-conjugating enzyme, putative
Cit.24483.1.S1_s_at	558.731	1221.546	-2.18629	AT4G37870.1	1.00E-164	PCK1 (PHOSPHOENOLPYRUVATE CARBOXYKINASE 1); AT
Cit.28240.1.S1_at	21.49042	47.08502	-2.19098	AT3G43540.2	1.00E-12	unknown protein
Cit.18630.1.S1_at	39.82021	87.30833	-2.19256		NA	
Cit.22659.1.S1_at	27.23921	59.7277	-2.19271		NA	
Cit.5060.1.S1_at	21.034	46.14267	-2.19372	AT3G07040.1	6.00E-18	RPM1 (RESISTANCE TO P. SYRINGAE PV MACULICOLA 1);
Cit.37281.1.S1_at	33.3516	73.21506	-2.19525	AT1G24100.1	9.00E-33	UGT74B1 (UDP-glucosyl transferase 74B1); UDP-glycosyl
Cit.13915.1.S1_at	818.873	1799.42	-2.19743	AT3G14440.1	0	NCED3 (NINE-CIS-EPOXYCAROTENOID DIOXYGENASE 3);
Cit.26406.1.S1_at	47.31527	103.9989	-2.198	AT5G35960.1	5.00E-62	protein kinase, putative
Cit.9461.1.S1_at	774.6643	1703.216	-2.19865	AT1G07400.1	4.00E-63	17.8 kDa class I heat shock protein (HSP17.8-CI)
Cit.24942.1.S1_x_at	23.41823	51.63194	-2.20478		NA	
Cit.13667.1.S1_s_at	142.9666	315.5418	-2.2071	AT4G05070.1	3.00E-09	unknown protein
Cit.17346.1.S1_s_at	50.41621	111.3265	-2.20815	AT1G31335.1	2.00E-06	unknown protein
Cit.38321.1.S1_at	33.40611	73.80276	-2.20926	AT2G24765.2	6.00E-18	ARF3 (ADP-RIBOSYLATION FACTOR 3); protein binding
Cit.8541.1.S1_at	39.72828	87.81539	-2.2104	AT4G02380.1	4.00E-20	SAG21 (SENESCENCE-ASSOCIATED GENE 21)
Cit.26433.1.S1_s_at	139.4573	308.345	-2.21104	AT1G52560.1	4.00E-73	26.5 kDa class I small heat shock protein-like (HSP26.5-P
Cit.9569.1.S1_at	238.6889	527.9639	-2.21193		NA	
Cit.8482.1.S1_x_at	1006.2	2227.526	-2.2138	AT2G47730.1	1.00E-65	ATGSTF8 (ARABIDOPSIS THALIANA GLUTATHIONE S-TRAI
Cit.6647.1.S1_s_at	173.9572	385.3891	-2.21542	AT5G41080.2	1.00E-106	glycerophosphoryl diester phosphodiesterase family pro
Cit.5478.1.S1_at	80.93093	179.4464	-2.21728	AT5G53050.1	1.00E-15	hydrolase, alpha/beta fold family protein
Cit.10566.1.S1_at	65.04553	144.269	-2.21797	AT1G52690.2	2.00E-25	late embryogenesis abundant protein, putative / LEA prc
Cit.30846.1.S1_at	71.31903	158.4061	-2.22109	AT1G50320.1	4.00E-48	ATHX; enzyme activator
Cit.30276.1.S1_at	27.37639	60.87127	-2.2235	AT4G37300.1	7.00E-05	MEE59 (maternal effect embryo arrest 59)
Cit.5493.1.S1_s_at	452.3904	1006.287	-2.22438	AT2G39705.1	2.00E-14	RTFL8 (ROTUNDIFOLIA LIKE 8)
Cit.4752.1.S1_s_at	133.6277	297.24	-2.22439	AT5G54165.1	3.00E-08	unknown protein
Cit.10277.1.S1_s_at	3822.566	8503.869	-2.22465	AT3G57520.1	0	AtSIP2 (Arabidopsis thaliana seed imbibition 2); hydrolas
Cit.4950.1.S1_at	22.19029	49.48985	-2.23025		NA	
Cit.8720.1.S1_x_at	144.0326	321.3687	-2.23122	AT5G54160.1	7.00E-35	ATOMT1 (O-METHYLTRANSFERASE 1); caffeate O-methy
Cit.28589.1.S1_x_at	34.82446	77.82212	-2.2347		NA	

Cit.38647.1.S1_at	48.11628	107.6301	-2.23687	AT5G56860.1	2.00E-09	GNC (GATA, nitrate-inducible, carbon metabolism-involv
Cit.3215.1.S1_s_at	243.9842	545.7944	-2.23701	AT5G03230.1	8.00E-41	unknown protein
Cit.21027.1.S1_at	24.2406	54.27581	-2.23905	AT2G17550.1	2.00E-24	unknown protein
Cit.13694.1.S1_at	122.0526	273.2929	-2.23914	AT4G38960.1	6.00E-28	zinc finger (B-box type) family protein
Cit.2809.1.S1_s_at	216.7502	485.4086	-2.23948	AT1G68840.1	1.00E-122	RAV2 (REGULATOR OF THE ATPASE OF THE VACUOLAR M
Cit.29096.1.S1_at	20.86561	46.76685	-2.24134	AT4G22505.1	2.00E-05	INVOLVED IN: lipid transport; CONTAINS InterPro DOMA
Cit.23261.1.S1_at	108.4412	243.298	-2.24359		NA	
Cit.10916.1.S1_at	108.4611	243.5147	-2.24518	AT5G02020.1	1.00E-37	unknown protein
Cit.20355.1.S1_at	24.27734	54.52183	-2.24579	AT4G21860.1	7.00E-62	MSRB2 (methionine sulfoxide reductase B 2); peptide-m
Cit.1779.1.S1_s_at	98.45179	221.2121	-2.24691	AT2G29500.1	4.00E-59	17.6 kDa class I small heat shock protein (HSP17.6B-CI)
Cit.28954.1.S1_at	21.3182	47.90226	-2.24701	AT3G26160.1	4.00E-39	CYP71B17; electron carrier/ heme binding / iron ion binc
Cit.7966.1.S1_x_at	82.47527	185.3825	-2.24773	AT1G17860.1	2.00E-31	trypsin and protease inhibitor family protein / Kunitz fan
Cit.22642.1.S1_at	43.31937	97.46753	-2.24998	AT4G15520.1	2.00E-18	tRNA/rRNA methyltransferase (SpoU) family protein
Cit.30128.1.S1_at	71.82532	161.6878	-2.25113	AT4G27360.1	2.00E-37	dynein light chain, putative
Cit.35652.1.S1_at	27.74946	62.47141	-2.25127		NA	
Cit.7343.1.S1_s_at	31.87902	71.77023	-2.25133	AT5G01830.1	1.00E-131	armadillo/beta-catenin repeat family protein / U-box do
Cit.15923.1.S1_at	57.20374	128.8933	-2.25323	AT1G58420.1	7.00E-26	FUNCTIONS IN: molecular_function unknown; INVOLVED
Cit.25008.1.S1_at	20.96173	47.25238	-2.25422	AT3G14460.1	1.00E-14	disease resistance protein (NBS-LRR class), putative
Cit.18238.1.S1_at	44.75279	100.9023	-2.25466		NA	
Cit.24339.1.S1_at	20.86184	47.04688	-2.25516	AT3G12500.1	1.00E-32	ATHCHIB (ARABIDOPSIS THALIANA BASIC CHITINASE); ch
Cit.8152.1.S1_x_at	21.47953	48.4758	-2.25684	AT4G34050.1	2.00E-94	caffeoyl-CoA 3-O-methyltransferase, putative
Cit.30699.1.S1_at	24.66979	55.69553	-2.25764	AT4G35310.1	3.00E-75	CPK5 (calmodulin-domain protein kinase 5); ATP binding
Cit.25526.1.S1_s_at	854.5361	1929.632	-2.2581	AT3G14620.1	3.00E-55	CYP72A8; electron carrier/ heme binding / iron ion bindi
Cit.34286.1.S1_at	314.1291	709.5532	-2.25879		NA	
Cit.37653.1.S1_at	29.6136	66.90854	-2.25939	AT5G52910.1	4.00E-42	ATIM (TIMELESS)
Cit.23998.1.S1_at	20.59807	46.65726	-2.26513	AT4G30720.1	5.00E-30	electron carrier/ oxidoreductase
Cit.2849.1.S1_at	67.82308	153.7294	-2.26662	AT3G20820.1	1.00E-159	leucine-rich repeat family protein
Cit.32002.1.S1_at	265.8353	602.6526	-2.26701	AT1G49210.1	1.00E-51	zinc finger (C3HC4-type RING finger) family protein
Cit.4030.1.S1_at	129.4496	294.2265	-2.2729	AT1G60190.1	4.00E-82	armadillo/beta-catenin repeat family protein / U-box do
Cit.40496.1.S1_s_at	319.4102	726.2801	-2.27382	AT4G25810.1	1.00E-129	XTR6 (XYLOGLUCAN ENDOTRANSGLYCOSYLASE 6); hydrc
Cit.34379.1.S1_at	34.87944	79.31499	-2.27398	AT2G30580.1	2.00E-16	DRIP2 (DREB2A-INTERACTING PROTEIN 2); protein bindi
Cit.23861.1.S1_at	38.46249	87.50821	-2.27516		NA	
Cit.1729.1.S1_s_at	433.5423	987.5057	-2.27776	AT5G20860.1	7.00E-87	pectinesterase family protein
Cit.11240.1.S1_at	23.47815	53.57551	-2.28193	AT1G02190.1	5.00E-62	CER1 protein, putative
Cit.21825.1.S1_at	140.4388	320.6221	-2.283	AT1G64380.1	8.00E-13	AP2 domain-containing transcription factor, putative

Cit.22632.1.S1_at	211.6208	483.4297	-2.28441	AT5G40460.1	5.00E-26	unknown protein
Cit.24444.1.S1_x_at	27.79285	63.66388	-2.29066		NA	
Cit.19573.1.S1_x_at	27.86672	64.17603	-2.30296		NA	
Cit.23964.1.S1_x_at	20.07412	46.24564	-2.30374	AT3G08580.2	3.00E-17	AAC1 (ADP/ATP CARRIER 1); ATP:ADP antiporter/ binding
Cit.8446.1.S1_at	45.06139	103.9676	-2.30724	AT1G53210.1	0	sodium/calcium exchanger family protein / calcium-bind
Cit.16361.1.S1_at	26.52929	61.28266	-2.31		NA	
Cit.20841.1.S1_at	70.97751	164.1485	-2.31268	AT5G50140.1	1.00E-05	ankyrin repeat family protein
Cit.16616.1.S1_at	20.45049	47.30503	-2.31315	AT2G48130.1	2.00E-17	protease inhibitor/seed storage/lipid transfer protein (LT
Cit.17187.1.S1_at	65.55026	151.8042	-2.31584	AT1G05575.1	4.00E-17	unknown protein
Cit.23055.1.S1_at	31.95115	74.00752	-2.31627		NA	
Cit.30598.1.S1_at	36.45063	84.54269	-2.31938	AT2G37025.2	6.00E-38	TRFL8 (TRF-LIKE 8); DNA binding
Cit.4999.1.S1_s_at	411.5313	955.3965	-2.32156	AT4G32480.1	4.00E-47	unknown protein
Cit.21717.1.S1_at	718.9478	1669.324	-2.3219	AT3G04720.1	2.00E-43	PR4 (PATHOGENESIS-RELATED 4); chitin binding
Cit.31434.1.S1_a_at	70.07445	163.2933	-2.33028		NA	
Cit.12779.1.S1_at	131.9118	308.9197	-2.34187	AT4G23810.1	3.00E-57	WRKY53; DNA binding / protein binding / transcription a
Cit.18713.1.S1_at	35.16968	82.44537	-2.34422		NA	
Cit.22603.1.S1_s_at	300.3396	704.8168	-2.34673	AT2G17880.1	3.00E-24	DNAJ heat shock protein, putative
Cit.23133.1.S1_x_at	28.63829	67.24862	-2.34821	AT4G39630.1	3.00E-14	unknown protein
Cit.22710.1.S1_x_at	499.9064	1174.567	-2.34957	AT4G11650.1	9.00E-25	ATOSM34 (osmotin 34)
Cit.1868.1.S1_s_at	2510.262	5906.504	-2.35294		NA	
Cit.37481.1.S1_at	63.66838	149.8182	-2.3531	AT3G15550.1	7.00E-79	unknown protein
Cit.21150.1.S1_at	40.24881	94.77148	-2.35464		NA	
Cit.7334.1.S1_at	358.505	846.5064	-2.36121	AT5G17350.1	7.00E-37	unknown protein
Cit.10152.1.S1_s_at	605.0816	1432.959	-2.36821	AT4G27410.2	1.00E-110	RD26 (RESPONSIVE TO DESICCATION 26); transcription a
Cit.17518.1.S1_at	30.64153	72.57364	-2.36847		NA	
Cit.11577.1.S1_s_at	1126.437	2669.592	-2.36994	AT1G30220.1	0	INT2 (INOSITOL TRANSPORTER 2); carbohydrate transme
Cit.2730.1.S1_at	125.5396	297.7905	-2.37208	AT5G58350.1	0	WNK4 (WITH NO K (=LYSINE) 4); kinase/ protein kinase
Cit.1007.1.S1_s_at	54.37813	129.0175	-2.3726	AT5G25560.1	1.00E-134	zinc finger (C3HC4-type RING finger) family protein
Cit.2928.1.S1_s_at	193.6583	460.9599	-2.38027	AT5G23810.1	1.00E-103	AAP7; amino acid transmembrane transporter
Cit.3524.1.S1_at	24.67512	58.76818	-2.38168	AT4G33000.2	2.00E-69	CBL10 (CALCINEURIN B-LIKE 10); calcium ion binding
Cit.28396.1.S1_at	46.0164	109.6139	-2.38206		NA	
Cit.7991.1.S1_x_at	580.2446	1382.676	-2.38292	AT1G17860.1	8.00E-33	trypsin and protease inhibitor family protein / Kunitz fan
Cit.37136.1.S1_s_at	210.0297	500.8872	-2.38484	AT1G05000.1	6.00E-85	tyrosine specific protein phosphatase family protein
Cit.5651.1.S1_s_at	395.3802	943.2314	-2.38563	AT1G77210.1	2.00E-68	sugar transporter, putative
Cit.16130.1.S1_at	87.34648	208.4014	-2.38592		NA	

Cit.2874.1.S1_at	33.8096	80.7828	-2.38935	AT5G18420.3	2.00E-23	unknown protein
Cit.36076.1.S1_at	185.503	445.3398	-2.40071	AT2G05910.1	2.00E-58	unknown protein
Cit.7274.1.S1_at	23.70776	56.95944	-2.40257	AT5G01410.1	5.00E-11	RSR4 (REDUCED SUGAR RESPONSE 4); protein heterodim
Cit.5891.1.S1_at	22.22879	53.4865	-2.40618	AT4G17500.1	3.00E-75	ATERF-1 (ETHYLENE RESPONSIVE ELEMENT BINDING FAC
Cit.21047.1.S1_s_at	299.117	720.2408	-2.40789	AT5G20790.1	1.00E-25	unknown protein
Cit.21195.1.S1_at	45.90686	110.9956	-2.41784	AT2G38870.1	2.00E-12	protease inhibitor, putative
Cit.29751.1.S1_at	92.45282	223.5881	-2.4184	AT5G20790.1	7.00E-17	unknown protein
Cit.7951.1.S1_s_at	20.00496	48.489	-2.42385	AT2G34260.1	1.00E-127	transducin family protein / WD-40 repeat family protein
Cit.8210.1.S1_x_at	35.73055	86.65056	-2.42511	AT1G54410.1	1.00E-19	dehydrin family protein
Cit.12285.1.S1_x_at	51.25278	124.3244	-2.42571	AT1G53310.3	1.00E-88	ATPPC1 (PHOSPHOENOLPYRUVATE CARBOXYLASE 1); cat
Cit.34169.1.S1_at	24.79525	60.14999	-2.42587		NA	
Cit.13701.1.S1_at	44.6122	108.5807	-2.43388	AT5G64400.1	2.00E-37	FUNCTIONS IN: molecular_function unknown; INVOLVED
Cit.17595.1.S1_x_at	74.04684	180.5773	-2.43869		NA	
Cit.10032.1.S1_s_at	501.17	1224.147	-2.44258	AT1G75750.1	3.00E-37	GASA1 (GAST1 PROTEIN HOMOLOG 1)
Cit.24864.1.S1_s_at	22.7491	55.72355	-2.44948	AT1G76880.1	8.00E-58	trihelix DNA-binding protein, putative
Cit.13689.1.S1_s_at	415.4402	1021.062	-2.45778	AT5G26731.1	3.00E-17	unknown protein
Cit.23371.1.S1_at	28.39166	69.78781	-2.45804	AT2G30860.1	9.00E-30	ATGSTF9 (GLUTATHIONE S-TRANSFERASE PHI 9); copper
Cit.31079.1.S1_at	103.8634	255.7636	-2.4625	AT4G37925.1	9.00E-41	NDH-M (subunit NDH-M of NAD(P)H:plastoquinone dehy
Cit.522.1.S1_s_at	151.9829	374.4064	-2.46348	AT4G05320.4	0	UBQ10 (POLYUBIQUITIN 10); protein binding
Cit.11606.1.S1_s_at	431.8576	1064.279	-2.46442	AT3G47420.1	0	glycerol-3-phosphate transporter, putative / glycerol 3-p
Cit.25712.1.S1_at	66.15563	163.349	-2.46916		NA	
Cit.60.1.S1_at	55.05711	136.0249	-2.47061	AT2G38540.1	1.00E-16	LP1; calmodulin binding
Cit.16237.1.S1_at	25.04921	61.97184	-2.474	ATMG00300.1	2.00E-10	hypothetical protein
Cit.7327.1.S1_at	26.37846	65.27747	-2.47465		NA	
Cit.15140.1.S1_at	36.82653	91.25371	-2.47793	AT5G54690.1	0	GAUT12 (GALACTURONOSYLTRANSFERASE 12); polygala
Cit.30629.1.S1_at	54.12493	134.2433	-2.48025	AT2G43790.1	1.00E-143	ATMPK6 (ARABIDOPSIS THALIANA MAP KINASE 6); MAP
Cit.26455.1.S1_at	61.51472	152.6645	-2.48176	AT5G67400.1	1.00E-60	peroxidase 73 (PER73) (P73) (PRXR11)
Cit.31144.1.S1_at	164.8066	409.8035	-2.48657	AT1G10070.3	1.00E-119	ATBCAT-2 (ARABIDOPSIS THALIANA BRANCHED-CHAIN A
Cit.10076.1.S1_at	29.34895	73.11499	-2.49123	AT5G04590.1	3.00E-27	SIR; sulfite reductase (ferredoxin)/ sulfite reductase
Cit.7468.1.S1_at	22.94677	57.29676	-2.49694	AT5G23750.2	4.00E-25	remorin family protein
Cit.32798.1.S1_at	29.53715	73.75452	-2.49701	AT5G42900.3	5.00E-07	unknown protein
Cit.13625.1.S1_at	25.93959	64.83563	-2.49949	AT1G14180.2	3.00E-52	protein binding / zinc ion binding
Cit.1200.1.S1_s_at	848.0539	2135.59	-2.51822	AT4G11650.1	9.00E-81	ATOSM34 (osmotin 34)
Cit.24920.1.S1_at	23.71162	59.79715	-2.52185		NA	
Cit.5112.1.S1_at	35.0145	88.36853	-2.52377	AT1G47128.1	1.00E-118	RD21 (responsive to dehydration 21); cysteine-type endo

Cit.21497.1.S1_at	211.416	534.3488	-2.52748	AT2G38905.1	5.00E-10	hydrophobic protein, putative / low temperature and sa
Cit.2307.1.S1_at	63.54168	160.857	-2.53152		NA	
Cit.24434.1.S1_at	21.81184	55.40459	-2.54012		NA	
Cit.20640.1.S1_at	61.91061	157.4297	-2.54285	AT1G64380.1	2.00E-08	AP2 domain-containing transcription factor, putative
Cit.38045.1.S1_at	71.98752	183.1137	-2.54369	AT5G18730.1	2.00E-26	FUNCTIONS IN: molecular_function unknown; INVOLVED
Cit.36745.1.S1_at	21.6999	55.23082	-2.54521		NA	
Cit.821.1.S1_at	26.11239	66.60628	-2.55075	AT2G45550.1	1.00E-77	CYP76C4; electron carrier/ heme binding / iron ion bindi
Cit.15137.1.S1_at	91.32589	233.2038	-2.55353	AT2G41380.1	1.00E-68	embryo-abundant protein-related
Cit.2630.1.S1_at	126.52	323.4444	-2.55647		NA	
Cit.18045.1.S1_s_at	270.1851	691.3738	-2.55889	AT1G01250.1	3.00E-26	AP2 domain-containing transcription factor, putative
Cit.34436.1.S1_at	26.33474	67.39917	-2.55933		NA	
Cit.5440.1.S1_s_at	218.6293	559.5858	-2.55952	AT2G45130.1	2.00E-76	SPX3 (SPX DOMAIN GENE 3)
Cit.30163.1.S1_at	49.98632	128.0623	-2.56195	AT1G72240.1	4.00E-12	unknown protein
Cit.7775.1.S1_at	48.04989	123.3792	-2.56773	AT1G17010.1	8.00E-11	oxidoreductase, 2OG-Fe(II) oxygenase family protein
Cit.6386.1.S1_at	64.85312	166.9543	-2.57434	AT1G27461.1	3.00E-85	unknown protein
Cit.23522.1.S1_at	54.03896	139.4563	-2.58066		NA	
Cit.2304.1.S1_at	32.79442	84.9906	-2.59162	AT2G36090.1	5.00E-11	F-box family protein
Cit.37764.1.S1_s_at	116.3959	303.0441	-2.60356	AT1G27730.1	7.00E-57	STZ (salt tolerance zinc finger); nucleic acid binding / tra
Cit.36807.1.S1_s_at	23.51845	61.2916	-2.60611	AT4G21200.1	2.00E-74	GA2OX8 (GIBBERELLIN 2-OXIDASE 8); gibberellin 2-beta-
Cit.36569.1.S1_at	28.55042	74.62735	-2.61388	AT2G40400.2	7.00E-29	unknown protein
Cit.24705.1.S1_at	25.83153	67.64906	-2.61886	AT5G46250.3	4.00E-48	RNA recognition motif (RRM)-containing protein
Cit.12748.1.S1_s_at	23.47897	61.49419	-2.61912	AT4G26530.2	1.00E-172	fructose-bisphosphate aldolase, putative
Cit.20992.1.S1_at	31.49129	82.49062	-2.61947		NA	
Cit.10669.1.S1_s_at	1954.791	5127.834	-2.62321	AT2G15960.1	7.00E-09	unknown protein
Cit.15785.1.S1_at	56.80077	149.1545	-2.62592	AT4G20970.1	2.00E-22	basic helix-loop-helix (bHLH) family protein
Cit.3377.1.S1_at	120.6491	317.6952	-2.63322	AT5G20230.1	4.00E-30	ATBCB (ARABIDOPSIS BLUE-COPPER-BINDING PROTEIN);
Cit.7295.1.S1_at	271.6582	715.3989	-2.63345	AT5G59080.1	3.00E-26	unknown protein
Cit.38354.1.S1_at	28.18724	74.36751	-2.63834	AT4G00110.1	1.00E-31	GAE3 (UDP-D-GLUCURONATE 4-EPIMERASE 3); UDP-gluc
Cit.25526.1.S1_at	28.25262	74.75819	-2.64606	AT3G14620.1	3.00E-55	CYP72A8; electron carrier/ heme binding / iron ion bindi
Cit.26572.1.S1_at	71.13889	188.3323	-2.64739	AT5G42930.1	3.00E-07	triacylglycerol lipase
Cit.26593.1.S1_at	1576.084	4176.418	-2.64987		NA	
Cit.18537.1.S1_at	82.10896	217.8081	-2.65267	AT2G15890.1	5.00E-18	MEE14 (maternal effect embryo arrest 14)
Cit.27183.1.S1_at	186.8814	496.5791	-2.65719		NA	
Cit.2906.1.S1_s_at	278.6035	743.1291	-2.66734		NA	
Cit.23845.1.S1_at	21.3207	56.93371	-2.67035	AT4G31130.1	3.00E-36	unknown protein

Cit.35421.1.S1_at	36.47972	97.42189	-2.67058	AT4G17610.1	4.00E-87	tRNA/rRNA methyltransferase (SpoU) family protein
Cit.20502.1.S1_at	33.58029	89.85841	-2.67593		NA	
Cit.13636.1.S1_s_at	503.6619	1349.437	-2.67925	AT1G23110.3	2.00E-44	unknown protein
Cit.17757.1.S1_at	24.772	66.38602	-2.67988	AT4G23690.1	3.00E-50	disease resistance-responsive family protein / dirigent fa
Cit.15006.1.S1_at	123.0825	329.8724	-2.68009	AT4G38540.1	2.00E-81	monooxygenase, putative (MO2)
Cit.35499.1.S1_at	61.66245	165.3753	-2.68195	AT4G22620.1	5.00E-44	auxin-responsive family protein
Cit.30596.1.S1_at	99.91694	268.8712	-2.69095	AT3G19615.1	3.00E-13	unknown protein
Cit.23345.1.S1_at	34.01295	91.72366	-2.69673		NA	
Cit.17188.1.S1_at	49.79105	134.3555	-2.69839	AT3G55646.1	2.00E-18	unknown protein
Cit.33184.1.S1_at	57.28646	154.7023	-2.7005		NA	
Cit.26693.1.S1_at	20.94169	56.67516	-2.70633	AT1G47500.1	3.00E-39	ATRBP47C' (RNA-binding protein 47C'); RNA binding
Cit.22036.1.S1_at	32.43432	88.00693	-2.71339	AT1G23780.1	1.00E-21	F-box family protein
Cit.6847.1.S1_at	25.26019	68.64162	-2.71738	AT3G04720.1	5.00E-44	PR4 (PATHOGENESIS-RELATED 4); chitin binding
Cit.6952.1.S1_at	23.55392	64.09668	-2.72127	AT2G42000.1	1.00E-15	plant EC metallothionein-like family 15 protein
Cit.38466.1.S1_at	20.66146	56.28456	-2.72413		NA	
Cit.23578.1.S1_x_at	50.27343	137.0043	-2.72518	AT1G68300.1	6.00E-06	universal stress protein (USP) family protein
Cit.24094.1.S1_at	233.9472	638.8372	-2.73069	AT2G31820.1	4.00E-28	ankyrin repeat family protein
Cit.26132.1.S1_at	24.64363	67.46465	-2.73761		NA	
Cit.31037.1.S1_at	24.83641	68.2043	-2.74614	AT5G16040.1	1.00E-108	regulator of chromosome condensation (RCC1) family pr
Cit.10152.1.S1_at	78.26735	214.9408	-2.74624		NA	
Cit.26654.1.S1_x_at	124.3027	341.4423	-2.74686		NA	
Cit.10213.1.S1_at	23.82275	65.462	-2.74788	AT1G10200.1	2.00E-87	WLIM1; transcription factor/ zinc ion binding
Cit.37293.1.S1_at	21.08269	57.93378	-2.74793	AT5G35740.1	6.00E-46	glycosyl hydrolase family protein 17
Cit.18687.1.S1_at	32.81029	90.40593	-2.75541	AT1G05680.1	3.00E-35	UDP-glucoronosyl/UDP-glucosyl transferase family prote
Cit.12839.1.S1_s_at	114.5502	317.0971	-2.76819	AT3G07350.1	1.00E-78	unknown protein
Cit.15311.1.S1_at	22.10118	61.40405	-2.77832		NA	
Cit.18687.1.S1_s_at	212.9626	594.7491	-2.79274	AT3G02100.1	6.00E-93	UDP-glucoronosyl/UDP-glucosyl transferase family prote
Cit.36616.1.S1_at	34.49089	96.35851	-2.79374		NA	
Cit.5819.1.S1_at	64.23972	180.9219	-2.81636	AT2G28900.1	1.00E-48	ATOEP16-1 (OUTER PLASTID ENVELOPE PROTEIN 16-1); [illegible]
Cit.39178.1.S1_s_at	2800.239	7957.968	-2.84189	AT5G06760.1	1.00E-48	late embryogenesis abundant group 1 domain-containin
Cit.1201.1.S1_at	126.6782	360.3014	-2.84423	AT4G11650.1	9.00E-81	ATOSM34 (osmotin 34)
Cit.13668.1.S1_at	37.6323	107.1811	-2.84811	AT4G05070.1	4.00E-09	unknown protein
Cit.1479.1.S1_s_at	294.2227	846.6816	-2.87769	AT5G48490.1	3.00E-18	protease inhibitor/seed storage/lipid transfer protein (L[illegible]
Cit.14918.1.S1_at	34.81188	100.2938	-2.88102	AT1G03220.1	1.00E-113	extracellular dermal glycoprotein, putative / EDGP, puta[illegible]
Cit.24922.1.S1_at	22.4129	64.79112	-2.8908		NA	

Cit.8515.1.S1_x_at	60.79895	175.9135	-2.89336	AT5G06730.1	1.00E-105	peroxidase, putative
Cit.28237.1.S1_at	44.96718	130.3951	-2.89978		NA	
Cit.1780.1.S1_at	270.4339	784.2732	-2.90006	AT2G29500.1	2.00E-58	17.6 kDa class I small heat shock protein (HSP17.6B-CI)
Cit.15728.1.S1_at	20.2636	58.76579	-2.90007	AT3G63470.1	6.00E-47	scpl40 (serine carboxypeptidase-like 40); serine-type car
Cit.2409.1.S1_s_at	1498.888	4366.456	-2.91313	AT3G09220.1	0	LAC7 (laccase 7); laccase
Cit.2942.1.S1_at	41.06905	119.708	-2.9148		NA	
Cit.3665.1.S1_at	258.9873	756.236	-2.91997	AT1G75750.1	2.00E-30	GASA1 (GAST1 PROTEIN HOMOLOG 1)
Cit.12037.1.S1_at	376.0383	1098.301	-2.92072		NA	
Cit.8903.1.S1_x_at	24.86975	73.00063	-2.93532		NA	
Cit.8661.1.S1_x_at	20.73309	60.85957	-2.93538	AT4G35800.1	3.00E-05	NRPB1 (RNA POLYMERASE II LARGE SUBUNIT); DNA bind
Cit.31285.1.S1_at	511.9719	1504.652	-2.93893	AT2G18700.1	7.00E-51	ATTPS11; transferase, transferring glycosyl groups
Cit.4377.1.S1_at	77.21613	227.687	-2.9487	AT1G47480.1	8.00E-79	hydrolase
Cit.20926.1.S1_s_at	233.4952	693.6338	-2.97066	AT4G32375.1	7.00E-07	glycoside hydrolase family 28 protein / polygalacturonas
Cit.22710.1.S1_at	986.0067	2929.734	-2.97131		NA	
Cit.21988.1.S1_at	39.08194	116.5978	-2.98342	AT5G20410.1	4.00E-66	MGD2; 1,2-diacylglycerol 3-beta-galactosyltransferase/ U
Cit.10328.1.S1_s_at	833.0273	2494.226	-2.99417	AT4G09600.1	2.00E-23	GASA3 (GAST1 PROTEIN HOMOLOG 3)
Cit.10672.1.S1_s_at	182.0187	545.152	-2.99503	AT5G07050.1	1.00E-155	LOCATED IN: membrane; CONTAINS InterPro DOMAIN/s
Cit.14889.1.S1_at	71.01724	212.9769	-2.99895	AT2G23620.1	4.00E-83	MES1 (METHYL ESTERASE 1); hydrolase, acting on ester b
Cit.8697.1.S1_at	200.0061	600.0538	-3.00018		NA	
Cit.23168.1.S1_at	225.4879	677.459	-3.00441		NA	
Cit.25735.1.S1_at	27.60812	82.97942	-3.00562		NA	
Cit.17074.1.S1_s_at	37.49657	112.9907	-3.01336	AT2G18950.1	1.00E-71	HPT1 (HOMOGENTISATE PHYTYLTRANSFERASE 1); homo
Cit.10033.1.S1_x_at	279.6682	845.4835	-3.02317	AT1G75750.1	2.00E-36	GASA1 (GAST1 PROTEIN HOMOLOG 1)
Cit.8027.1.S1_s_at	22.62121	68.46491	-3.02658	AT3G12580.1	0	HSP70 (heat shock protein 70); ATP binding
Cit.15760.1.S1_at	126.2176	383.6269	-3.03941	AT4G01070.1	6.00E-90	GT72B1; UDP-glucosyltransferase/ UDP-glycosyltransfera
Cit.5745.1.S1_s_at	35.26699	107.3389	-3.04361		NA	
Cit.26052.1.S1_s_at	52.47982	160.4875	-3.05808	AT3G55646.1	3.00E-29	unknown protein
Cit.4621.1.S1_at	34.1553	104.5033	-3.05965		NA	
Cit.21833.1.S1_x_at	61.55779	188.5794	-3.06345	AT5G53130.1	6.00E-08	CNGC1 (CYCLIC NUCLEOTIDE GATED CHANNEL 1); calmo
Cit.36604.1.S1_at	40.72066	124.7794	-3.06428	AT3G17520.1	2.00E-17	late embryogenesis abundant domain-containing protein
Cit.5477.1.S1_s_at	73.04675	226.3326	-3.09846	AT1G31130.1	2.00E-36	unknown protein
Cit.22748.1.S1_x_at	23.27005	72.17614	-3.10168	AT2G29250.1	3.00E-11	lectin protein kinase, putative
Cit.28507.1.S1_at	21.31327	66.32748	-3.11203	AT2G28840.1	1.00E-30	ankyrin repeat family protein
Cit.6765.1.S1_at	130.3594	406.1852	-3.11589	AT3G26330.1	9.00E-69	CYP71B37; electron carrier/ heme binding / iron ion binc
Cit.21833.1.S1_at	58.3478	181.9982	-3.1192	AT5G53130.1	6.00E-08	CNGC1 (CYCLIC NUCLEOTIDE GATED CHANNEL 1); calmo

Cit.11963.1.S1_at	153.4997	479.3845	-3.12303	AT4G32375.1	7.00E-07	glycoside hydrolase family 28 protein / polygalacturonas
Cit.9703.1.S1_at	75.28751	235.6556	-3.13008	AT3G57270.1	1.00E-113	BG1 (BETA-1,3-GLUCANASE 1); catalytic/ cation binding /
Cit.29796.1.S1_at	74.68909	234.1821	-3.13543	AT1G21010.1	3.00E-07	unknown protein
Cit.21182.1.S1_s_at	125.4319	395.5944	-3.15386	AT1G62730.1	1.00E-48	transferase
Cit.25990.1.S1_x_at	830.8309	2626.116	-3.16083	AT1G49640.1	5.00E-06	hydrolase
Cit.14674.1.S1_s_at	21.06225	66.66694	-3.16523	AT5G10930.1	5.00E-68	CIPK5 (CBL-INTERACTING PROTEIN KINASE 5); ATP bindir
Cit.13417.1.S1_s_at	78.70335	250.3982	-3.18154	AT1G08650.1	4.00E-77	PPCK1 (PHOSPHOENOLPYRUVATE CARBOXYLASE KINASE
Cit.29822.1.S1_at	51.54784	164.2978	-3.18729	AT1G77210.1	7.00E-75	sugar transporter, putative
Cit.6827.1.S1_x_at	118.6118	378.4686	-3.19082	AT1G17010.1	7.00E-81	oxidoreductase, 2OG-Fe(II) oxygenase family protein
Cit.25991.1.S1_at	21.40063	68.54557	-3.20297		NA	
Cit.14576.1.S1_s_at	21.53665	69.52706	-3.22831	AT1G01490.2	4.00E-32	heavy-metal-associated domain-containing protein
Cit.3665.1.S1_s_at	1392.539	4525.361	-3.24972	AT1G75750.1	2.00E-30	GASA1 (GAST1 PROTEIN HOMOLOG 1)
Cit.24784.1.S1_x_at	27.50894	89.60546	-3.25732	AT1G71695.1	5.00E-57	peroxidase 12 (PER12) (P12) (PRXR6)
Cit.36207.1.S1_at	447.1276	1463.191	-3.27242		NA	
Cit.2116.1.S1_s_at	401.1137	1318.146	-3.28622	AT4G11650.1	2.00E-87	ATOSM34 (osmotin 34)
Cit.27382.1.S1_s_at	29.28057	96.60185	-3.29918	AT4G14147.1	6.00E-07	ARPC4; protein binding
Cit.21310.1.S1_s_at	45.98948	152.7521	-3.32146	AT4G17500.1	3.00E-75	ATERF-1 (ETHYLENE RESPONSIVE ELEMENT BINDING FAC
Cit.10194.1.S1_x_at	21.9256	73.04649	-3.33156	AT1G30910.1	1.00E-123	molybdenum cofactor sulfurase family protein
Cit.30672.1.S1_x_at	942.2199	3142.844	-3.33557		NA	
Cit.3018.1.S1_at	28.91526	96.51332	-3.3378	AT5G09660.1	1.00E-164	PMDH2 (peroxisomal NAD-malate dehydrogenase 2); ma
Cit.30130.1.S1_at	25.91552	86.81442	-3.3499		NA	
Cit.6491.1.S1_at	24.05934	81.2942	-3.3789	AT2G42560.1	5.00E-22	late embryogenesis abundant domain-containing protei
Cit.10471.1.S1_s_at	341.7859	1156.297	-3.3831	AT2G26660.1	1.00E-108	SPX2 (SPX DOMAIN GENE 2)
Cit.25965.1.S1_x_at	250.6267	858.7298	-3.42633		NA	
Cit.30672.1.S1_at	1080.083	3741.443	-3.46403		NA	
Cit.12794.1.S1_at	23.24966	80.64594	-3.46869	AT1G33140.1	2.00E-90	PGY2 (PIGGYBACK2); structural constituent of ribosome
Cit.15637.1.S1_at	39.17924	136.7444	-3.49023	AT4G28780.1	9.00E-91	GDSL-motif lipase/hydrolase family protein
Cit.29385.1.S1_at	27.83623	98.02084	-3.52134	AT5G52450.1	1.00E-57	MATE efflux protein-related
Cit.8587.1.S1_s_at	256.0144	902.9907	-3.52711		NA	
Cit.104.1.S1_at	28.60913	101.016	-3.5309		NA	
Cit.20561.1.S1_at	555.3547	1969.849	-3.54701		NA	
Cit.24660.1.S1_at	30.53488	109.6635	-3.59142	AT4G27090.1	4.00E-11	60S ribosomal protein L14 (RPL14B)
Cit.15458.1.S1_at	187.4063	677.6744	-3.61607	AT1G18400.1	1.00E-45	BEE1 (BR Enhanced Expression 1); transcription factor
Cit.16048.1.S1_at	44.35498	162.2507	-3.658	AT1G75490.1	8.00E-46	DNA binding / transcription factor
Cit.448.1.S1_at	20.98426	77.01991	-3.67037	AT1G23820.1	1.00E-147	SPDS1 (spermidine synthase 1); spermidine synthase

Cit.11083.1.S1_s_at	454.3946	1686.411	-3.71134	AT3G02040.1	1.00E-141	SRG3 (senescence-related gene 3); glycerophosphodiest
Cit.22079.1.S1_x_at	30.99422	115.7354	-3.7341	AT1G69840.6	2.00E-33	band 7 family protein
Cit.18120.1.S1_at	474.3336	1781.028	-3.7548	AT5G56550.1	2.00E-21	OXS3 (OXIDATIVE STRESS 3)
Cit.14158.1.S1_at	27.98048	105.7114	-3.77804	AT1G61560.1	1.00E-113	MLO6 (MILDEW RESISTANCE LOCUS O 6); calmodulin bir
Cit.27555.1.S1_at	56.44405	213.9856	-3.79111	AT5G56970.1	1.00E-58	CKX3 (CYTOKININ OXIDASE 3); amine oxidase/ cytokinin
Cit.13787.1.S1_s_at	42.71057	162.7254	-3.80996	AT2G03200.1	1.00E-50	aspartyl protease family protein
Cit.10454.1.S1_at	35.26855	134.7769	-3.82145		NA	
Cit.7094.1.S1_at	22.05566	84.47325	-3.83	AT3G51580.1	7.00E-31	unknown protein
Cit.21988.1.S1_s_at	54.62344	209.9463	-3.84352	AT5G20410.1	8.00E-74	MGD2; 1,2-diacylglycerol 3-beta-galactosyltransferase/ U
Cit.19639.1.S1_at	28.75675	112.4032	-3.90876		NA	
Cit.3920.1.S1_s_at	45.79854	180.3421	-3.93773	AT1G80760.1	1.00E-138	NIP6;1 (NOD26-LIKE INTRINSIC PROTEIN 6;1); boron tran
Cit.18299.1.S1_at	24.52553	97.48328	-3.97477	AT2G23270.1	1.00E-06	unknown protein
Cit.40196.1.S1_at	22.55127	89.70082	-3.97764		NA	
Cit.6076.1.S1_s_at	508.5383	2050.529	-4.0322	AT1G22990.1	2.00E-51	heavy-metal-associated domain-containing protein / cop
Cit.26629.1.S1_at	28.58824	115.6284	-4.04461		NA	
Cit.3571.1.S1_at	20.82122	84.5542	-4.06096	AT2G21490.1	1.00E-36	LEA (DEHYDRIN LEA)
Cit.25990.1.S1_at	268.3309	1093.662	-4.0758	AT1G49640.1	5.00E-06	hydrolase
Cit.28048.1.S1_at	23.37016	95.69081	-4.09457	AT5G11060.1	5.00E-24	KNAT4 (KNOTTED1-LIKE HOMEOBOX GENE 4); transcript
Cit.29637.1.S1_at	47.09073	193.1255	-4.10114		NA	
Cit.20042.1.S1_at	39.77541	163.8284	-4.11884	AT1G68765.1	6.00E-06	IDA (INFLORESCENCE DEFICIENT IN ABSCISSION); recepto
Cit.34473.1.S1_at	53.69595	222.5622	-4.14486		NA	
Cit.24426.1.S1_s_at	510.4246	2154.209	-4.22043	AT1G73010.1	1.00E-110	phosphatase
Cit.7539.1.S1_at	38.87637	167.9132	-4.31916	AT3G24310.1	5.00E-44	MYB305 (myb domain protein 305); DNA binding / trans
Cit.5117.1.S1_at	181.4427	795.0229	-4.38167	AT3G62550.1	1.00E-53	universal stress protein (USP) family protein
Cit.15073.1.S1_at	22.44649	99.33392	-4.42537	AT2G03200.1	6.00E-48	aspartyl protease family protein
Cit.20302.1.S1_s_at	24.1068	107.0348	-4.44003	AT5G66780.1	9.00E-29	unknown protein
Cit.23385.1.S1_x_at	21.74474	96.9755	-4.45972	AT3G48120.1	4.00E-30	unknown protein
Cit.10521.1.S1_at	88.39391	408.4141	-4.62039		NA	
Cit.2899.1.S1_s_at	373.9508	1752.766	-4.68716	AT1G73010.1	1.00E-110	phosphatase
Cit.2900.1.S1_at	367.8781	1731.109	-4.70566	AT1G73010.1	1.00E-110	phosphatase
Cit.10673.1.S1_at	241.9865	1239.804	-5.12344	AT3G26740.1	4.00E-29	CCL (CCR-LIKE)
Cit.30798.1.S1_at	515.8552	2673.274	-5.18222	AT5G05340.1	1.00E-95	peroxidase, putative
Cit.13606.1.S1_at	609.917	3195.852	-5.23981	AT5G58390.1	5.00E-83	peroxidase, putative
Cit.11676.1.S1_at	71.81473	404.6088	-5.63406		NA	
Cit.6376.1.S1_at	229.576	1293.835	-5.63576	AT1G68320.1	2.00E-78	MYB62 (myb domain protein 62); DNA binding / transcri

Cit.4566.1.S1_at	266.3389	1528.273	-5.73808	AT4G37580.1	6.00E-70	HLS1 (HOOKLESS 1); N-acetyltransferase
Cit.19346.1.S1_at	245.7474	1442.344	-5.86921		NA	
Cit.2635.1.S1_at	20.58381	130.4888	-6.33939	AT3G03520.1	1.00E-78	phosphoesterase family protein
Cit.10453.1.S1_s_at	100.7814	682.472	-6.77181		NA	
Cit.4361.1.S1_at	26.93407	192.6125	-7.15126		NA	
Cit.3195.1.S1_at	139.3073	1022.7	-7.34132	AT1G33055.1	7.00E-13	unknown protein
Cit.1727.1.S1_s_at	151.2855	1252.75	-8.2807	AT3G54420.1	6.00E-76	ATEP3; chitinase
Cit.25956.1.S1_x_at	27.36308	242.8329	-8.87447	AT1G26690.1	2.00E-08	emp24/gp25L/p24 family protein
Cit.6332.1.S1_at	68.08031	707.9664	-10.399	AT2G34930.1	5.00E-35	disease resistance family protein
Cit.17589.1.S1_x_at	32.4498	363.243	-11.194	AT4G02380.1	8.00E-15	SAG21 (SENESCENCE-ASSOCIATED GENE 21)
CitAffx.1.1.S1_at	65.2971	739.7766	-11.3294	NA	NA	NA
Cit.18677.1.S1_s_at	185.9122	2120.24	-11.4045	AT3G54420.1	6.00E-76	ATEP3; chitinase

Supplementary Table S5. PDTA in seedless vs. seedy grapefruits at time point 2.

ProbeSet ID	Grapefruit_Seedless	Grapefruit_Seedy	Ratio	AtGID	E-Score	Arabidopsis.annotation
Cit.10894.1.S1_s_at	6104.588	81.35658	75.03496	AT5G06760.1	1.00E-48	late embryogenesis abundant group 1 domain-containin
Cit.10894.1.S1_x_at	3387.979	55.62599	60.9064		NA	
Cit.39178.1.S1_s_at	1962.666	35.57423	55.171	AT5G06760.1	1.00E-48	late embryogenesis abundant group 1 domain-containin
Cit.11619.1.S1_at	667.2596	63.70122	10.47483	AT1G24020.1	5.00E-10	MLP423 (MLP-LIKE PROTEIN 423)
Cit.21182.1.S1_s_at	1143.265	124.8961	9.153729	AT1G62730.1	1.00E-48	transferase
Cit.4566.1.S1_at	431.7103	48.46674	8.907352	AT4G37580.1	6.00E-70	HLS1 (HOOKLESS 1); N-acetyltransferase
Cit.5970.1.S1_at	162.1226	21.84181	7.422581	AT5G61430.1	6.00E-92	ANAC100 (ARABIDOPSIS NAC DOMAIN CONTAINING PRO
Cit.21182.1.S1_at	318.4098	46.86289	6.794498		NA	
Cit.25064.1.S1_s_at	876.0129	133.533	6.560273	AT1G17860.1	2.00E-27	trypsin and protease inhibitor family protein / Kunitz fan
Cit.30657.1.S1_s_at	1496.512	241.6125	6.193852	AT4G36740.1	1.00E-50	ATHB40 (ARABIDOPSIS THALIANA HOMEOBOX PROTEIN
Cit.57.1.S1_at	843.6548	140.2355	6.015986	AT1G17860.1	2.00E-27	trypsin and protease inhibitor family protein / Kunitz fan
Cit.9569.1.S1_at	166.251	28.15279	5.905312		NA	
Cit.57.1.S1_x_at	969.2918	167.4864	5.787287	AT1G17860.1	2.00E-27	trypsin and protease inhibitor family protein / Kunitz fan
Cit.20129.1.S1_at	126.5047	22.07846	5.729779		NA	
Cit.4875.1.S1_at	118.5432	22.00706	5.386599		NA	
Cit.5421.1.S1_at	180.6221	33.62717	5.371314		NA	
Cit.22170.1.S1_x_at	112.9646	21.117	5.349463	AT5G37990.1	3.00E-12	S-adenosylmethionine-dependent methyltransferase/ m
Cit.13694.1.S1_at	337.7144	63.25948	5.338558	AT4G38960.1	6.00E-28	zinc finger (B-box type) family protein
Cit.17413.1.S1_s_at	187.8715	36.11513	5.202016	AT4G33467.2	1.00E-09	unknown protein
Cit.20355.1.S1_at	262.7011	51.51194	5.09981	AT4G21860.1	7.00E-62	MSRB2 (methionine sulfoxide reductase B 2); peptide-m
Cit.36207.1.S1_at	346.9995	68.69796	5.051089		NA	
Cit.9568.1.S1_s_at	5220.773	1068.204	4.887431	AT5G64260.1	1.00E-125	EXL2 (EXORDIUM LIKE 2)
Cit.21345.1.S1_at	151.9221	32.43967	4.68322		NA	
Cit.24022.1.S1_s_at	1926.728	415.2917	4.639457	AT3G53670.1	9.00E-23	unknown protein
Cit.31428.1.S1_at	94.90338	20.94092	4.531958		NA	
Cit.22158.1.S1_x_at	1999.832	498.9973	4.007701	AT1G17860.1	3.00E-12	trypsin and protease inhibitor family protein / Kunitz fan
Cit.40496.1.S1_s_at	203.6938	50.98946	3.994822	AT4G25810.1	1.00E-129	XTR6 (XYLOGLUCAN ENDOTRANSGLYCOSYLASE 6); hydro
Cit.11918.1.S1_x_at	119.1899	30.14118	3.954387	AT4G38540.1	2.00E-78	monooxygenase, putative (MO2)
Cit.14222.1.S1_at	749.0733	194.5238	3.850805	AT1G58170.1	8.00E-56	disease resistance-responsive protein-related / dirigent p
Cit.9523.1.S1_s_at	1404.671	368.3847	3.813055	AT2G47770.1	6.00E-43	benzodiazepine receptor-related
Cit.22077.1.S1_at	109.4185	28.89826	3.786335		NA	

Cit.20573.1.S1_at	139.6025	37.73239	3.699805		NA	
Cit.24877.1.S1_x_at	79.41759	21.49186	3.69524		NA	
Cit.17346.1.S1_at	131.9111	35.85069	3.679458	AT1G31335.1	2.00E-06	unknown protein
Cit.36935.1.S1_s_at	421.816	116.1973	3.63017	AT1G19640.1	2.00E-91	JMT (JASMONIC ACID CARBOXYL METHYLTRANSFERASE)
Cit.39551.1.S1_at	72.66451	20.15425	3.605419	AT4G00500.2	1.00E-12	lipase class 3 family protein / calmodulin-binding heat-sł
Cit.13258.1.S1_at	75.18143	21.03282	3.574482	AT4G34215.2	2.00E-86	hydrolase
Cit.17309.1.S1_at	121.7888	34.51238	3.528844	AT3G03341.1	3.00E-28	unknown protein
Cit.31285.1.S1_at	200.1891	57.23273	3.497808	AT2G18700.1	7.00E-51	ATTPS11; transferase, transferring glycosyl groups
Cit.23685.1.S1_at	155.6729	44.61658	3.489127	AT3G06850.2	4.00E-62	BCE2; acetyltransferase/ alpha-ketoacid dehydrogenase,
Cit.22157.1.S1_at	87.79295	25.33742	3.464952	AT5G15780.1	1.00E-14	pollen Ole e 1 allergen and extensin family protein
Cit.15781.1.S1_at	82.45414	23.86708	3.454723		NA	
Cit.7991.1.S1_x_at	5781.634	1678.994	3.443511	AT1G17860.1	8.00E-33	trypsin and protease inhibitor family protein / Kunitz fan
Cit.197.1.S1_x_at	10250.09	2994.982	3.422421	AT5G20620.1	0	UBQ4; protein binding
Cit.5128.1.S1_at	79.28571	23.17651	3.420951	AT2G35660.1	1.00E-177	CTF2A; monooxygenase/ oxidoreductase
Cit.8185.1.S1_s_at	80.86162	24.15143	3.348109	AT2G25490.1	8.00E-22	EBF1 (EIN3-BINDING F BOX PROTEIN 1); protein binding ,
Cit.23504.1.S1_at	344.8967	103.1267	3.344398		NA	
Cit.2659.1.S1_x_at	82.78653	24.9746	3.314829	AT1G56290.1	1.00E-137	CwfJ-like family protein
Cit.30907.1.S1_at	84.43598	25.63862	3.293312	AT4G19800.1	7.00E-62	glycosyl hydrolase family 18 protein
Cit.3215.1.S1_s_at	198.7798	61.12648	3.251943	AT5G03230.1	8.00E-41	unknown protein
Cit.23753.1.S1_at	377.8091	117.5941	3.212824		NA	
Cit.10278.1.S1_x_at	376.0375	117.7642	3.193139	AT3G57520.1	5.00E-81	AtSIP2 (Arabidopsis thaliana seed imbibition 2); hydrolas
Cit.17523.1.S1_at	81.57381	25.61022	3.185205	AT1G06475.1	4.00E-06	unknown protein
Cit.27351.1.S1_at	83.87196	26.37864	3.179541	AT5G55000.2	2.00E-09	FIP2; protein binding / voltage-gated potassium channel
Cit.8648.1.S1_at	82.93055	26.11212	3.175941		NA	
Cit.753.1.S1_x_at	596.0211	187.8426	3.172982	AT3G04720.1	1.00E-46	PR4 (PATHOGENESIS-RELATED 4); chitin binding
Cit.31463.1.S1_at	89.72596	28.36299	3.163487		NA	
Cit.26512.1.S1_at	74.92855	23.81369	3.146449	AT4G11740.1	2.00E-67	SAY1
Cit.10277.1.S1_s_at	4645.96	1478.097	3.143204	AT3G57520.1	0	AtSIP2 (Arabidopsis thaliana seed imbibition 2); hydrolas
Cit.35955.1.S1_at	112.6369	35.9269	3.135169	AT2G02340.1	6.90E-21	AtPP2-B8 (Phloem protein 2-B8); carbohydrate binding
Cit.37379.1.S1_at	89.04298	28.46609	3.128037	AT1G25500.2	2.00E-46	choline transporter-related
Cit.7567.1.S1_at	76.4502	24.61759	3.105511		NA	
Cit.11918.1.S1_at	99.42187	32.02064	3.104931	AT4G38540.1	2.00E-78	monooxygenase, putative (MO2)
Cit.3036.1.S1_s_at	668.4255	216.0784	3.09344	AT3G04920.1	9.00E-63	40S ribosomal protein S24 (RPS24A)
Cit.26865.1.S1_at	81.31808	26.49841	3.068791	AT5G07630.1	1.00E-19	lipid transporter
Cit.31260.1.S1_at	180.2663	58.77956	3.066819		NA	

Cit.15523.1.S1_at	137.3578	44.84593	3.062882	AT3G26330.1	5.00E-71	CYP71B37; electron carrier/ heme binding / iron ion binc
Cit.18043.1.S1_x_at	132.5235	43.28827	3.061418	AT1G48380.1	5.00E-30	RHL1 (ROOT HAIRLESS 1); DNA binding / protein binding
Cit.20561.1.S1_at	178.3226	58.3398	3.05662		NA	
Cit.2550.1.S1_at	115.7068	37.9145	3.051782	AT1G51600.2	1.00E-101	TIFY2A; sequence-specific DNA binding / transcription fa
Cit.26938.1.S1_at	212.8337	70.00176	3.040405	AT4G38220.2	2.00E-44	aminoacylase, putative / N-acyl-L-amino-acid amidohydr
Cit.30233.1.S1_at	71.47371	23.61651	3.02643		NA	
Cit.5641.1.S1_at	102.5157	34.00365	3.014844	AT1G06620.1	1.00E-102	2-oxoglutarate-dependent dioxygenase, putative
Cit.10673.1.S1_at	162.0188	53.86831	3.007683	AT3G26740.1	4.00E-29	CCL (CCR-LIKE)
Cit.580.1.S1_x_at	180.7886	60.23022	3.001626	AT3G04720.1	9.00E-47	PR4 (PATHOGENESIS-RELATED 4); chitin binding
Cit.4522.1.S1_at	224.522	74.8359	3.000191	AT5G38050.1	3.00E-71	unknown protein
Cit.27225.1.S1_at	103.9441	34.78094	2.988536	AT1G61010.3	3.00E-26	CPSF73-I (CLEAVAGE AND POLYADENYLATION SPECIFICIT
Cit.29107.1.S1_at	75.6424	25.39728	2.978366	AT4G18820.1	7.00E-32	ATP binding / DNA binding / DNA-directed DNA polymer
Cit.10328.1.S1_s_at	301.0879	101.3817	2.969845	AT4G09600.1	2.00E-23	GASA3 (GAST1 PROTEIN HOMOLOG 3)
Cit.25795.1.S1_s_at	1909.615	648.132	2.946337	AT3G57520.1	0	AtSIP2 (Arabidopsis thaliana seed imbibition 2); hydrolas
Cit.18174.1.S1_at	408.9254	139.7414	2.926301	AT5G49550.1	1.00E-28	unknown protein
Cit.16558.1.S1_at	98.14068	33.67804	2.914085	AT3G24495.1	1.00E-91	MSH7 (MUTS HOMOLOG 7); ATP binding / damaged DN/
Cit.35443.1.S1_at	103.9021	35.68714	2.911472	AT3G48380.2	5.00E-62	FUNCTIONS IN: molecular_function unknown; INVOLVEL
Cit.10661.1.S1_at	143.8342	49.41442	2.910774	AT1G12240.1	0	ATBETAFRUCT4; beta-fructofuranosidase/ hydrolase, hy
Cit.7966.1.S1_x_at	790.8743	271.8096	2.909663	AT1G17860.1	2.00E-31	trypsin and protease inhibitor family protein / Kunitz fan
Cit.1557.1.S1_s_at	5638.521	1945.029	2.898939		NA	
Cit.31451.1.S1_s_at	198.5485	68.54006	2.896824	AT1G11530.1	5.00E-37	ATCXXS1 (C-terminal cysteine residue is changed to a ser
Cit.3549.1.S1_at	432.381	149.5659	2.890906	AT3G12360.1	9.00E-55	ITN1 (INCREASED TOLERANCE TO NACL); protein binding
Cit.10060.1.S1_at	411.6037	142.7182	2.884031	AT3G47340.1	0	ASN1 (GLUTAMINE-DEPENDENT ASPARAGINE SYNTHASE
Cit.14472.1.S1_s_at	259.757	90.11813	2.882406		NA	
Cit.3006.1.S1_at	813.988	282.7112	2.879221	AT5G10140.2	4.00E-35	FLC (FLOWERING LOCUS C); specific transcriptional repre
Cit.16782.1.S1_at	468.1904	162.9294	2.873578	AT4G15236.1	6.00E-36	ATP binding / ATPase/ nucleoside-triphosphatase/ nucle
Cit.19812.1.S1_at	68.87768	23.99233	2.870821		NA	
Cit.5793.1.S1_s_at	451.0289	157.2854	2.867583	AT3G03341.1	3.00E-28	unknown protein
Cit.25344.1.S1_at	90.53979	31.59436	2.865695	AT3G42170.1	2.00E-15	DNA binding
Cit.28006.1.S1_at	224.138	78.22964	2.865129	AT4G03440.1	1.00E-08	ankyrin repeat family protein
Cit.23637.1.S1_x_at	86.21786	30.1103	2.863401		NA	
Cit.5575.1.S1_at	237.9091	83.6668	2.843531	AT3G50170.1	9.00E-50	unknown protein
Cit.18335.1.S1_at	61.71141	21.74371	2.838127	AT4G11670.1	9.00E-39	FUNCTIONS IN: molecular_function unknown; INVOLVEL
Cit.27488.1.S1_at	75.10065	26.56793	2.826741		NA	
Cit.39578.1.S1_at	235.0063	83.23165	2.823521	AT3G42170.1	3.00E-08	DNA binding

Cit.6727.1.S1_at	118.3813	42.03323	2.816374	AT2G45690.1	2.00E-24	SSE1 (SHRUNKEN SEED 1)
Cit.21289.1.S1_at	67.49337	24.08627	2.802151		NA	
Cit.21627.1.S1_at	69.91354	24.96883	2.800033	AT5G66240.2	3.00E-07	transducin family protein / WD-40 repeat family protein
Cit.25271.1.S1_at	62.32653	22.28398	2.796921		NA	
Cit.20841.1.S1_at	72.32526	25.91123	2.791271	AT5G50140.1	1.00E-05	ankyrin repeat family protein
Cit.24173.1.S1_at	63.19423	22.64841	2.790228	AT1G07220.1	4.00E-64	FUNCTIONS IN: molecular_function unknown; INVOLVED
Cit.6582.1.S1_at	620.0923	222.3565	2.78873	AT4G15545.1	2.00E-87	unknown protein
Cit.27344.1.S1_at	85.06349	30.55844	2.783633	AT5G42905.1	1.00E-27	nucleic acid binding / ribonuclease H
Cit.1928.1.S1_s_at	537.2969	194.7338	2.759135	AT1G78860.1	1.00E-117	curculin-like (mannose-binding) lectin family protein
Cit.1554.1.S1_at	78.22797	28.36793	2.75762	AT1G27330.1	3.00E-29	FUNCTIONS IN: molecular_function unknown; INVOLVED
Cit.8426.1.S1_at	329.1994	119.9681	2.744058	AT5G47080.1	1.00E-118	CKB1; protein kinase regulator
Cit.21497.1.S1_at	271.9829	99.36304	2.737264	AT2G38905.1	5.00E-10	hydrophobic protein, putative / low temperature and sa
Cit.38085.1.S1_at	72.20811	26.58159	2.716471		NA	
Cit.33267.1.S1_at	55.58996	20.48826	2.713259	AT4G21870.1	5.00E-28	26.5 kDa class P-related heat shock protein (HSP26.5-P)
Cit.13978.1.S1_at	173.4887	63.94964	2.712896	AT4G16510.1	3.00E-92	YbaK/prolyl-tRNA synthetase-related
Cit.40196.1.S1_at	61.13079	22.59896	2.705027		NA	
Cit.7685.1.S1_at	122.9633	45.64893	2.693673	AT5G02540.1	2.00E-37	short-chain dehydrogenase/reductase (SDR) family prote
Cit.10058.1.S1_s_at	418.3869	155.457	2.691335	AT3G47340.1	0	ASN1 (GLUTAMINE-DEPENDENT ASPARAGINE SYNTHASE
Cit.26141.1.S1_s_at	2125.021	790.7535	2.687337		NA	
Cit.12580.1.S1_at	57.12759	21.33844	2.677215	AT1G47640.1	1.00E-114	unknown protein
Cit.15399.1.S1_at	101.1258	37.98289	2.662404	AT1G12600.1	3.00E-64	FUNCTIONS IN: molecular_function unknown; INVOLVED
Cit.12814.1.S1_s_at	751.5507	283.3604	2.652279	AT5G07330.1	2.00E-36	unknown protein
Cit.25022.1.S1_at	63.57953	23.99037	2.65021	AT4G01470.1	5.00E-27	TIP1;3 (TONOPLAST INTRINSIC PROTEIN 1;3); urea transp
Cit.5916.1.S1_at	305.8601	115.5215	2.647647		NA	
Cit.9580.1.S1_at	81.75474	30.92966	2.643247	AT3G55610.1	7.00E-16	P5CS2 (DELTA 1-PYRROLINE-5-CARBOXYLATE SYNTHASE
Cit.18706.1.S1_x_at	80.03487	30.33046	2.638762		NA	
Cit.31171.1.S1_at	54.658	20.72778	2.636944	AT3G51290.1	1.00E-81	proline-rich family protein
Cit.7969.1.S1_x_at	2315.269	878.8865	2.634321	AT1G17860.1	8.00E-31	trypsin and protease inhibitor family protein / Kunitz fan
Cit.38531.1.S1_at	127.7076	48.49732	2.633292	AT5G55760.1	2.00E-49	SRT1 (sirtuin 1); DNA binding / NAD or NADH binding / N
Cit.19369.1.S1_at	93.85591	35.64276	2.633239	AT3G14470.1	2.00E-12	disease resistance protein (NBS-LRR class), putative
Cit.30060.1.S1_at	68.71215	26.10022	2.632627		NA	
Cit.27349.1.S1_at	125.9595	47.98945	2.624733		NA	
Cit.5266.1.S1_at	77.80105	29.66593	2.622572	AT4G16380.1	4.00E-56	metal ion binding
Cit.26788.1.S1_at	82.95865	31.64679	2.621392	AT2G25450.1	3.00E-36	2-oxoglutarate-dependent dioxygenase, putative
Cit.18045.1.S1_s_at	129.7389	49.50098	2.620936	AT1G01250.1	3.00E-26	AP2 domain-containing transcription factor, putative

Cit.26629.1.S1_at	142.7271	54.52365	2.61771		NA	
Cit.7478.1.S1_at	126.9516	48.87861	2.597283	AT2G30120.2	2.00E-47	unknown protein
Cit.22079.1.S1_x_at	53.27233	20.55334	2.591906	AT1G69840.6	2.00E-33	band 7 family protein
Cit.18311.1.S1_at	93.79523	36.32216	2.582314		NA	
Cit.22067.1.S1_s_at	1248.319	483.4691	2.582004	AT3G58110.1	4.00E-46	unknown protein
Cit.1536.1.S1_x_at	546.3096	211.6767	2.580868	AT3G52590.1	5.00E-69	UBQ1 (UBIQUITIN EXTENSION PROTEIN 1); protein bindi
Cit.24947.1.S1_at	76.16351	29.521	2.579977		NA	
Cit.21542.1.S1_at	58.64513	22.78728	2.573591		NA	
Cit.4810.1.S1_at	64.00303	24.89141	2.57129	AT3G23240.1	2.00E-64	ERF1 (ETHYLENE RESPONSE FACTOR 1); DNA binding / tr
Cit.21833.1.S1_at	93.95313	36.58606	2.568003	AT5G53130.1	6.00E-08	CNGC1 (CYCLIC NUCLEOTIDE GATED CHANNEL 1); calmo
Cit.37994.1.S1_at	108.2885	42.18998	2.566688		NA	
Cit.36527.1.S1_at	54.76133	21.33855	2.56631	AT1G80880.1	4.00E-62	pentatricopeptide (PPR) repeat-containing protein
Cit.16519.1.S1_at	139.3675	54.39507	2.562135	AT2G31060.2	3.00E-63	elongation factor family protein
Cit.26518.1.S1_at	59.78335	23.34541	2.560818		NA	
Cit.24874.1.S1_x_at	59.2334	23.16149	2.557409		NA	
Cit.31355.1.S1_at	109.3303	42.75401	2.557194		NA	
Cit.25808.1.S1_at	95.03091	37.2092	2.553963		NA	
Cit.8463.1.S1_at	92.2664	36.14373	2.552764	AT2G38870.1	1.00E-17	protease inhibitor, putative
Cit.16732.1.S1_at	54.61361	21.40163	2.551843	AT2G26890.2	9.00E-16	GRV2 (GRAVITROPISM DEFECTIVE 2); binding / heat shoc
Cit.17929.1.S1_s_at	217.9841	85.57449	2.547302		NA	
Cit.3995.1.S1_x_at	64.17367	25.20838	2.545728		NA	
Cit.30951.1.S1_at	55.56033	21.88951	2.538217		NA	
Cit.38996.1.S1_s_at	84.8263	33.42472	2.537831	AT3G03870.2	1.00E-37	unknown protein
Cit.8608.1.S1_at	1159.163	456.8838	2.537107	AT5G06740.1	4.00E-21	lectin protein kinase family protein
Cit.3327.1.S1_at	51.43082	20.30796	2.532545	AT1G15860.2	9.00E-71	INVOLVED IN: biological_process unknown; EXPRESSED I
Cit.17346.1.S1_s_at	71.86742	28.38181	2.532165	AT1G31335.1	2.00E-06	unknown protein
Cit.36161.1.S1_at	52.53224	20.81498	2.523771	AT2G28760.3	4.00E-08	NAD-dependent epimerase/dehydratase family protein
Cit.9809.1.S1_x_at	345.2925	137.3122	2.514653	AT3G16080.1	6.00E-47	60S ribosomal protein L37 (RPL37C)
Cit.10060.1.S1_s_at	1412.619	563.1976	2.508212	AT3G47340.1	0	ASN1 (GLUTAMINE-DEPENDENT ASPARAGINE SYNTHASE
Cit.190.1.S1_at	145.4052	58.15246	2.500414	AT5G01150.1	2.00E-05	unknown protein
Cit.3905.1.S1_at	1578.368	631.9801	2.497496	AT2G18950.1	8.00E-59	HPT1 (HOMOGENTISATE PHYTYLTRANSFERASE 1); homo
Cit.22324.1.S1_at	53.87988	21.60207	2.4942	AT3G22740.1	1.00E-61	HMT3; homocysteine S-methyltransferase
Cit.27263.1.S1_at	66.25337	26.7084	2.480619	AT5G06700.1	2.00E-16	unknown protein
Cit.5721.1.S1_s_at	89.32734	36.05735	2.477368	AT3G48690.1	1.00E-95	CXE12; carboxylesterase
Cit.157.1.S1_x_at	349.1976	140.9701	2.477104		NA	

Cit.30633.1.S1_at	305.6845	123.4605	2.47597	AT5G28830.1	1.00E-57	calcium-binding EF hand family protein
Cit.16557.1.S1_at	171.8186	69.40303	2.475664	AT1G04280.1	2.00E-53	unknown protein
Cit.12743.1.S1_at	59.15546	23.95275	2.469673	AT2G29420.1	9.00E-55	ATGSTU7 (ARABIDOPSIS THALIANA GLUTATHIONE S-TRA
Cit.31032.1.S1_at	267.426	108.3548	2.468059	AT3G52280.1	9.00E-73	GTE6 (GENERAL TRANSCRIPTION FACTOR GROUP E6); DI
Cit.29391.1.S1_s_at	98.33721	39.85519	2.467363	AT1G59950.1	3.00E-99	aldo/keto reductase, putative
Cit.23067.1.S1_x_at	240.3339	97.42985	2.466738		NA	
Cit.35188.1.S1_at	55.56689	22.532	2.466132	AT1G69935.1	3.00E-36	SHW1 (SHORT HYPOCOTYL IN WHITE LIGHT1)
Cit.2854.1.S1_at	126.1373	51.42528	2.452827	AT1G15120.1	1.00E-29	ubiquinol-cytochrome C reductase complex 7.8 kDa prot
Cit.10906.1.S1_at	83.3773	34.00367	2.452009	AT1G43670.1	1.00E-178	fructose-1,6-bisphosphatase, putative / D-fructose-1,6-b
Cit.1320.1.S1_s_at	1008.609	411.9749	2.448229	AT5G65730.1	1.00E-132	xyloglucan:xyloglucosyl transferase, putative / xyloglucan
Cit.6076.1.S1_s_at	395.2684	162.0036	2.439874	AT1G22990.1	2.00E-51	heavy-metal-associated domain-containing protein / cop
Cit.9132.1.S1_s_at	135.7244	55.65897	2.4385	AT4G19420.1	1.00E-156	pectinacetylesterase family protein
Cit.25808.1.S1_x_at	82.69458	33.92249	2.437751		NA	
Cit.16098.1.S1_at	54.26077	22.28558	2.434793	AT2G43150.1	3.00E-64	proline-rich extensin-like family protein
Cit.11964.1.S1_s_at	49.72469	20.42298	2.434742	AT2G27690.1	0	CYP94C1; fatty acid (omega-1)-hydroxylase/ oxygen binc
Cit.5514.1.S1_at	311.7514	128.0536	2.434538	AT3G63060.1	4.00E-56	EDL3 (EID1-like 3)
Cit.3005.1.S1_at	91.48019	37.63051	2.431011	AT5G10140.2	6.00E-31	FLC (FLOWERING LOCUS C); specific transcriptional repre
Cit.27444.1.S1_at	49.8081	20.5592	2.422667		NA	
Cit.33200.1.S1_at	65.53337	27.06174	2.421624	AT3G62310.1	4.00E-12	RNA helicase, putative
Cit.38374.1.S1_at	158.356	65.4361	2.42001	AT3G55610.1	4.00E-15	P5CS2 (DELTA 1-PYRROLINE-5-CARBOXYLATE SYNTHASE
Cit.10053.1.S1_at	795.3626	328.6619	2.420002	AT4G15560.1	0	CLA1 (CLOROPLASTOS ALTERADOS 1); 1-deoxy-D-xylulos
Cit.9803.1.S1_s_at	1300.943	538.0231	2.418006		NA	
Cit.28703.1.S1_at	210.8278	87.23907	2.416667	AT5G53220.3	3.00E-11	unknown protein
Cit.4721.1.S1_at	81.69548	33.90189	2.409762	AT2G36970.1	2.00E-22	UDP-glucoronosyl/UDP-glucosyl transferase family prote
Cit.22961.1.S1_x_at	63.21409	26.33057	2.400787	AT1G03250.1	7.00E-07	unknown protein
Cit.2576.1.S1_at	64.78377	27.0674	2.393424	AT4G13360.1	1.00E-173	catalytic
Cit.2927.1.S1_s_at	299.0662	124.9819	2.392876	AT5G23810.1	2.00E-96	AAP7; amino acid transmembrane transporter
Cit.2778.1.S1_at	288.4749	120.658	2.390848	AT3G44400.2	1.00E-08	disease resistance protein (TIR-NBS-LRR class), putative
Cit.39470.1.S1_at	70.9583	29.75818	2.384497	AT2G34730.1	2.00E-20	myosin heavy chain-related
Cit.38717.1.S1_at	83.20321	34.93825	2.381436		NA	
Cit.37714.1.S1_at	53.8513	22.61705	2.381005	AT5G15920.1	1.00E-55	structural maintenance of chromosomes (SMC) family pr
Cit.23750.1.S1_at	58.36454	24.5132	2.380943	AT5G18820.1	2.00E-42	EMB3007 (embryo defective 3007); ATP binding / protei
Cit.16374.1.S1_at	955.1524	401.6237	2.378227	AT3G53690.1	2.00E-66	zinc finger (C3HC4-type RING finger) family protein
Cit.10280.1.S1_x_at	3310.835	1395.264	2.372909	AT3G57520.1	0	AtSIP2 (Arabidopsis thaliana seed imbibition 2); hydrolas
Cit.23578.1.S1_x_at	65.57104	27.70406	2.366839	AT1G68300.1	6.00E-06	universal stress protein (USP) family protein

Cit.29327.1.S1_x_at	990.9475	419.1197	2.364354	AT1G69530.4	1.00E-58	ATEXPA1 (ARABIDOPSIS THALIANA EXPANSIN A1)
Cit.21514.1.S1_at	77.95354	32.97202	2.364233		NA	
Cit.18043.1.S1_at	53.53442	22.67585	2.360856	AT1G48380.1	5.00E-30	RHL1 (ROOT HAIRLESS 1); DNA binding / protein binding
Cit.8464.1.S1_s_at	13275.46	5631.771	2.357244	AT1G06430.1	1.00E-55	FTSH8; ATP-dependent peptidase/ ATPase/ metallopept
Cit.814.1.S1_s_at	52.4034	22.27027	2.353065	AT3G01500.3	1.00E-134	CA1 (CARBONIC ANHYDRASE 1); carbonate dehydratase/
Cit.22463.1.S1_s_at	279.1355	118.6612	2.352374	AT4G27410.2	1.00E-110	RD26 (RESPONSIVE TO DESICCATION 26); transcription a
Cit.29859.1.S1_s_at	391.6184	166.8522	2.347098	AT5G16510.2	6.00E-52	reversibly glycosylated polypeptide, putative
Cit.18605.1.S1_at	69.32899	29.588	2.343146		NA	
Cit.12282.1.S1_at	120.7026	51.5219	2.342744	AT2G25670.2	4.00E-70	unknown protein
Cit.37467.1.S1_at	54.44985	23.27682	2.339231	AT3G10370.1	2.00E-07	SDP6 (SUGAR-DEPENDENT 6); glycerol-3-phosphate deh
Cit.38971.1.S1_s_at	54.88781	23.50239	2.335414	AT4G01520.1	4.00E-08	anac067 (Arabidopsis NAC domain containing protein 67
Cit.22696.1.S1_at	1674.591	717.1425	2.335088	AT4G12610.2	4.00E-11	transcription initiation factor IIF alpha subunit (TFIIF-alp
Cit.2474.1.S1_at	354.5632	151.8508	2.334945	AT4G31940.1	1.00E-26	CYP82C4; electron carrier/ heme binding / iron ion bindi
Cit.30979.1.S1_at	286.7761	122.8968	2.333471	AT4G18390.2	3.00E-36	TCP family transcription factor, putative
Cit.12875.1.S1_at	58.92503	25.30491	2.328601	AT1G20810.1	1.00E-78	immunophilin / FKBP-type peptidyl-prolyl cis-trans isom
Cit.19646.1.S1_at	46.66272	20.04396	2.328019		NA	
Cit.23948.1.S1_x_at	55.0928	23.66791	2.327743		NA	
Cit.16124.1.S1_at	61.11172	26.26614	2.326635		NA	
Cit.28446.1.S1_at	57.44257	24.69061	2.326495	AT2G46910.1	2.00E-06	plastid-lipid associated protein PAP / fibrillin family prot
Cit.28107.1.S1_at	71.32198	30.67941	2.324751	AT2G31955.2	1.00E-118	CNX2 (COFACTOR OF NITRATE REDUCTASE AND XANTHI
Cit.6081.1.S1_at	55.74374	24.03448	2.319324	AT2G42490.1	3.00E-84	copper amine oxidase, putative
Cit.29892.1.S1_at	56.35995	24.33656	2.315855		NA	
Cit.19697.1.S1_at	295.7802	127.7229	2.315796		NA	
Cit.634.1.S1_at	59.9283	25.94994	2.309381	AT3G56510.2	5.00E-65	TBP-binding protein, putative
Cit.5334.1.S1_at	57.05344	24.71244	2.308693	AT5G58800.2	1.00E-87	quinone reductase family protein
Cit.4770.1.S1_at	83.26846	36.11278	2.305789	AT4G24340.1	8.00E-55	phosphorylase family protein
Cit.24536.1.S1_at	77.73792	33.7461	2.303612	AT5G53940.1	1.00E-08	yippee family protein
Cit.2563.1.S1_at	55.43684	24.07918	2.302273	AT5G48930.1	5.00E-24	HCT (HYDROXYCINNAMOYL-COA SHIKIMATE/QUINATE H
Cit.29404.1.S1_x_at	95.88893	41.65916	2.301749	AT4G12320.1	1.00E-84	CYP706A6; electron carrier/ heme binding / iron ion bin
Cit.32070.1.S1_at	115.8502	50.42074	2.29767	AT3G12340.1	2.00E-36	FK506 binding / peptidyl-prolyl cis-trans isomerase
Cit.28521.1.S1_at	51.95744	22.61847	2.297124	AT5G62890.3	5.00E-79	permease, putative
Cit.14664.1.S1_at	92.93284	40.60629	2.288632	AT1G71692.1	3.00E-70	AGL12 (AGAMOUS-LIKE 12); transcription factor
Cit.32360.1.S1_at	202.9245	88.68064	2.288262		NA	
Cit.30991.1.S1_at	176.1171	77.00711	2.287024	AT3G18240.2	2.00E-72	unknown protein
Cit.21642.1.S1_at	80.3551	35.17167	2.284654	AT4G08280.1	2.00E-18	FUNCTIONS IN: molecular_function unknown; INVOLVE

Cit.13824.1.S1_at	61.27877	26.82368	2.284503	AT1G64500.1	8.00E-84	glutaredoxin family protein
Cit.20999.1.S1_at	117.4191	51.41466	2.283767		NA	
Cit.10753.1.S1_at	72.6096	31.80204	2.283174	AT4G19040.2	1.00E-60	EDR2; lipid binding
Cit.35779.1.S1_at	66.36284	29.08927	2.281351	AT3G61370.1	2.00E-25	unknown protein
Cit.10033.1.S1_x_at	206.7167	90.67667	2.279712	AT1G75750.1	2.00E-36	GASA1 (GAST1 PROTEIN HOMOLOG 1)
Cit.15543.1.S1_at	348.3698	153.1134	2.27524	AT5G44390.1	5.00E-23	FAD-binding domain-containing protein
Cit.35729.1.S1_at	99.10445	43.56854	2.274679		NA	
Cit.26889.1.S1_at	53.63392	23.59014	2.273574	AT3G19500.1	3.00E-39	ethylene-responsive protein -related
Cit.31535.1.S1_at	92.90799	40.86472	2.27355	AT3G66658.1	4.00E-75	ALDH22a1 (Aldehyde Dehydrogenase 22a1); 3-chloroally
Cit.29368.1.S1_x_at	1917.936	844.5298	2.27101	AT1G17860.1	4.00E-28	trypsin and protease inhibitor family protein / Kunitz fan
Cit.37369.1.S1_at	78.30775	34.51025	2.269116	AT5G43560.2	2.00E-33	meprin and TRAF homology domain-containing protein /
Cit.4050.1.S1_at	383.0124	168.8214	2.268743	AT5G23040.1	1.00E-106	CDF1 (CELL GROWTH DEFECT FACTOR 1)
Cit.31164.1.S1_at	93.70258	41.39738	2.263491		NA	
Cit.25076.1.S1_at	45.99486	20.32911	2.262512	AT1G18880.1	2.00E-10	proton-dependent oligopeptide transport (POT) family p
Cit.30330.1.S1_at	55.90631	24.76015	2.257915	AT5G45140.1	1.00E-129	NRPC2; DNA binding / DNA-directed RNA polymerase/ ri
Cit.11673.1.S1_at	98.64993	43.78791	2.252903	AT3G05190.1	7.00E-12	aminotransferase class IV family protein
Cit.30156.1.S1_at	203.7172	90.45827	2.252057		NA	
Cit.12105.1.S1_at	73.07778	32.46053	2.251281	AT5G16820.2	1.00E-128	HSF3 (HEAT SHOCK FACTOR 3); DNA binding / transcripti
Cit.32549.1.S1_at	45.87973	20.39153	2.249941		NA	
Cit.21484.1.S1_at	60.15283	26.74885	2.248801		NA	
Cit.5234.1.S1_at	999.4378	444.4737	2.248587		NA	
Cit.17907.1.S1_at	1499.954	667.4789	2.247193	AT1G49320.1	3.00E-44	BURP domain-containing protein
Cit.23069.1.S1_x_at	46.5407	20.73786	2.244238	AT5G48490.1	9.00E-07	protease inhibitor/seed storage/lipid transfer protein (LT
Cit.22580.1.S1_at	69.25438	30.86488	2.243792	AT3G26230.1	7.00E-53	CYP71B24; electron carrier/ heme binding / iron ion binc
Cit.19083.1.S1_at	56.01927	24.97571	2.24295	AT4G39200.2	6.00E-06	40S ribosomal protein S25 (RPS25E)
Cit.7948.1.S1_at	133.7664	59.80524	2.2367	AT1G22460.1	5.00E-15	unknown protein
Cit.23284.1.S1_x_at	82.39793	36.86917	2.234873	AT1G68300.1	4.00E-30	universal stress protein (USP) family protein
Cit.13103.1.S1_at	60.44844	27.09807	2.230729	AT1G79280.1	1.00E-10	NUA (NUCLEAR PORE ANCHOR)
Cit.2167.1.S1_at	76.00903	34.07748	2.230477	AT1G49410.1	9.00E-19	TOM6 (translocase of the outer mitochondrial membran
Cit.26543.1.S1_at	74.54733	33.45045	2.22859	AT3G42170.1	1.00E-05	DNA binding
Cit.20503.1.S1_at	46.20844	20.79591	2.221997		NA	
Cit.22748.1.S1_x_at	233.1336	104.9476	2.221429	AT2G29250.1	3.00E-11	lectin protein kinase, putative
Cit.14792.1.S1_at	72.70767	32.76683	2.218941	AT5G52230.1	4.00E-33	MBD13; methyl-CpG binding
Cit.29803.1.S1_at	166.7439	75.20348	2.217236	AT5G28300.1	4.00E-11	trihelix DNA-binding protein, putative
Cit.1319.1.S1_s_at	889.4493	401.7143	2.214134	AT5G65730.1	1.00E-132	xyloglucan:xyloglucosyl transferase, putative / xyloglucar

Cit.22984.1.S1_x_at	62.98525	28.44887	2.213981	AT5G05320.1	6.00E-20	monooxygenase, putative (MO3)
Cit.7399.1.S1_at	1028.813	464.8932	2.213009	AT1G35350.1	1.00E-157	LOCATED IN: integral to membrane; EXPRESSED IN: 18 p
Cit.24040.1.S1_at	66.94459	30.27699	2.211072		NA	
Cit.13915.1.S1_at	452.9154	204.8734	2.210709	AT3G14440.1	0	NCED3 (NINE-CIS-EPOXYCAROTENOID DIOXYGENASE 3);
Cit.22877.1.S1_at	45.30536	20.4959	2.21046	AT1G07670.1	6.00E-19	calcium-transporting ATPase
Cit.27874.1.S1_at	47.83971	21.7	2.204595	AT5G17880.1	5.00E-14	CSA1 (constitutive shade-avoidance1); ATP binding / trar
Cit.32879.1.S1_at	46.05483	20.89748	2.203846		NA	
Cit.32458.1.S1_at	135.1562	61.3922	2.201521	AT1G02110.1	5.00E-11	proline-rich family protein
Cit.37328.1.S1_at	115.147	52.35535	2.199336	AT3G03550.1	2.00E-60	zinc finger (C3HC4-type RING finger) family protein
Cit.28072.1.S1_at	100.6036	45.746	2.199178	AT5G42800.1	1.00E-05	DFR (DIHYDROFLAVONOL 4-REDUCTASE); dihydrokaemp
Cit.16070.1.S1_at	67.80849	30.89182	2.195031	AT2G38290.1	1.00E-137	ATAMT2 (AMMONIUM TRANSPORTER 2); ammonium tra
Cit.17433.1.S1_at	372.386	169.7108	2.194239		NA	
Cit.38075.1.S1_at	46.34423	21.12592	2.193714	AT5G62090.2	1.00E-08	FUNCTIONS IN: molecular_function unknown; INVOLVED
Cit.34962.1.S1_at	75.4166	34.3854	2.193274		NA	
Cit.37277.1.S1_at	269.4605	123.005	2.190647	AT3G14460.1	3.00E-07	disease resistance protein (NBS-LRR class), putative
Cit.27780.1.S1_at	88.69652	40.59333	2.185002	AT5G09650.1	8.00E-11	AtPPa6 (Arabidopsis thaliana pyrophosphorylase 6); inor
Cit.30031.1.S1_at	131.7259	60.30087	2.184478	AT3G45100.2	0	SETH2; transferase, transferring glycosyl groups
Cit.12824.1.S1_s_at	283.0787	129.5891	2.184433	AT1G01540.2	1.00E-124	protein kinase family protein
Cit.32471.1.S1_at	112.8313	51.76082	2.179859	AT4G18140.2	2.00E-05	phosphatase
Cit.31154.1.S1_at	106.3394	48.78621	2.179702		NA	
Cit.32121.1.S1_at	92.59914	42.52822	2.177358	AT4G21300.1	2.00E-70	pentatricopeptide (PPR) repeat-containing protein
Cit.20520.1.S1_at	115.9569	53.27511	2.176568		NA	
Cit.3978.1.S1_x_at	43.65638	20.07987	2.174137	AT5G66030.1	1.00E-31	ATGRIP; protein binding
Cit.11985.1.S1_at	118.85	54.77853	2.169646	AT5G52300.2	2.00E-66	LTI65 (LOW-TEMPERATURE-INDUCED 65)
Cit.26894.1.S1_at	43.97424	20.27478	2.168913	AT3G25070.1	5.00E-05	RIN4 (RPM1 INTERACTING PROTEIN 4); protein binding
Cit.11329.1.S1_s_at	128.5293	59.26292	2.168798	AT1G27950.1	2.00E-13	LTPG1 (GLYCOSYLPHOSPHATIDYLINOSITOL-ANCHORED L
Cit.4189.1.S1_x_at	84.74582	39.11018	2.166848	AT2G28110.1	1.00E-58	FRA8 (FRAGILE FIBER 8); glucuronosyltransferase/ transf
Cit.31069.1.S1_at	46.43362	21.43225	2.16653	AT4G39050.1	3.00E-65	kinesin-related protein (MKRP2)
Cit.40326.1.S1_at	147.3743	68.07241	2.164964	AT3G25710.1	6.00E-17	BHLH32 (BASIC HELIX-LOOP-HELIX 32); DNA binding / tra
Cit.26995.1.S1_at	92.47014	42.73281	2.163914		NA	
Cit.30324.1.S1_at	75.2911	34.80821	2.163027	AT2G33560.1	4.00E-65	spindle checkpoint protein-related
Cit.26735.1.S1_at	44.67506	20.67319	2.161014	AT4G07410.2	2.00E-42	transducin family protein / WD-40 repeat family protein
Cit.29276.1.S1_at	74.1544	34.32067	2.160634	AT5G15090.1	5.00E-07	VDAC3 (VOLTAGE DEPENDENT ANION CHANNEL 3); volta
Cit.7516.1.S1_at	163.4698	75.72683	2.158677	AT5G55160.1	5.00E-16	SUMO2 (SMALL UBIQUITIN-LIKE MODIFIER 2); protein bi
Cit.23254.1.S1_x_at	51.17097	23.73853	2.155608	AT2G17800.2	1.00E-30	ARAC1; GTP binding

Cit.28667.1.S1_at	324.7468	150.6678	2.155383		NA	
Cit.26432.1.S1_s_at	48.36469	22.46951	2.152459	AT1G29810.1	6.00E-47	dehydratase family
Cit.6794.1.S1_s_at	72.83139	33.83752	2.152386	AT4G35790.1	4.00E-78	ATPLDDELTA; phospholipase D
Cit.32267.1.S1_at	94.77313	44.13416	2.147387	AT1G58210.1	1.00E-25	EMB1674 (EMBRYO DEFECTIVE 1674)
Cit.13518.1.S1_at	830.0017	386.7423	2.146136	AT2G27880.1	5.00E-16	AGO5 (ARGONAUTE 5); nucleic acid binding
Cit.17223.1.S1_s_at	813.3109	379.2404	2.144579		NA	
Cit.22941.1.S1_at	151.2557	70.54298	2.144164	AT5G25170.1	2.00E-47	unknown protein
Cit.27576.1.S1_at	44.10048	20.59725	2.141086		NA	
Cit.14254.1.S1_at	52.63986	24.61362	2.138648		NA	
Cit.29940.1.S1_at	206.4818	96.55579	2.138471	AT5G57050.2	1.00E-119	ABI2 (ABA INSENSITIVE 2); protein serine/threonine pho
Cit.21510.1.S1_at	78.68668	36.82285	2.136898	AT2G29580.1	6.00E-16	zinc finger (CCCH-type) family protein / RNA recognition
Cit.7951.1.S1_s_at	61.55972	28.83937	2.134572	AT2G34260.1	1.00E-127	transducin family protein / WD-40 repeat family protein
Cit.35044.1.S1_at	55.79511	26.14225	2.134289		NA	
Cit.7553.1.S1_at	98.60172	46.20605	2.133957	AT2G01300.1	5.00E-19	unknown protein
Cit.25116.1.S1_at	60.97041	28.62136	2.130242	AT4G12640.1	2.00E-75	RNA recognition motif (RRM)-containing protein
Cit.5296.1.S1_s_at	100.6366	47.34435	2.125631	AT2G28930.3	1.00E-171	APK1B; ATP binding / kinase/ protein kinase/ protein ser
Cit.12095.1.S1_at	93.76522	44.1213	2.125169	AT5G56170.1	4.00E-54	FUNCTIONS IN: molecular_function unknown; INVOLVED
Cit.31019.1.S1_at	54.54568	25.6967	2.122673	AT3G17040.1	3.00E-66	HCF107 (HIGH CHLOROPHYLL FLUORESCENT 107); bindir
Cit.31635.1.S1_at	101.7045	47.93167	2.121864	AT5G27650.1	2.00E-27	PWWP domain-containing protein
Cit.39409.1.S1_at	51.87565	24.45013	2.121692	AT5G11450.1	1.00E-65	oxygen-evolving complex-related
Cit.25474.1.S1_x_at	1672.464	788.556	2.12092	AT5G60390.3	1.00E-108	elongation factor 1-alpha / EF-1-alpha
Cit.21407.1.S1_at	44.32716	20.90061	2.120855	AT3G53480.1	5.00E-46	PDR9 (PLEIOTROPIC DRUG RESISTANCE 9); ATPase, coup
Cit.16210.1.S1_at	107.9472	50.90078	2.120738	AT1G31240.1	3.00E-37	DNA binding
Cit.26361.1.S1_at	45.03835	21.24719	2.119732	AT3G57062.1	5.00E-10	unknown protein
Cit.31374.1.S1_at	208.4467	98.34997	2.119438	AT2G45550.1	1.00E-34	CYP76C4; electron carrier/ heme binding / iron ion bindi
Cit.361.1.S1_at	209.125	98.71582	2.118455	AT1G15670.1	1.00E-108	kelch repeat-containing F-box family protein
Cit.10152.1.S1_s_at	216.0629	102.0189	2.117871	AT4G27410.2	1.00E-110	RD26 (RESPONSIVE TO DESICCATION 26); transcription a
Cit.21905.1.S1_s_at	457.8172	216.2791	2.116789		NA	
Cit.14453.1.S1_at	323.7848	153.2094	2.113348	AT3G25830.1	1.00E-138	ATTPS-CIN (terpene synthase-like sequence-1,8-cineole)
Cit.30387.1.S1_at	536.5223	254.0305	2.112039	AT1G03530.1	1.00E-16	NAF1 (NUCLEAR ASSEMBLY FACTOR 1)
Cit.18491.1.S1_at	431.6967	204.4581	2.111419		NA	
Cit.16135.1.S1_at	48.3786	22.93017	2.109823	AT1G23980.1	1.00E-33	zinc finger (C3HC4-type RING finger) family protein
Cit.15517.1.S1_at	99.12898	46.98949	2.109599	AT1G48970.1	8.00E-61	GTP binding / translation initiation factor
Cit.21541.1.S1_s_at	44.57698	21.13438	2.109216		NA	
Cit.30130.1.S1_at	54.36244	25.78375	2.108399		NA	

Cit.15956.1.S1_at	88.95488	42.21142	2.107365	AT3G54190.1	1.00E-141	FUNCTIONS IN: molecular_function unknown; INVOLVED
Cit.21071.1.S1_x_at	72.76025	34.53204	2.107036		NA	
Cit.640.1.S1_s_at	282.1977	133.9724	2.106387	AT4G24280.1	0	cpHsc70-1 (chloroplast heat shock protein 70-1); ATP bir
Cit.30204.1.S1_at	103.1167	48.97691	2.105415	AT4G12430.1	3.00E-23	trehalose-6-phosphate phosphatase, putative
Cit.24328.1.S1_x_at	46.76638	22.2132	2.105342	AT2G05790.1	7.00E-39	glycosyl hydrolase family 17 protein
Cit.37919.1.S1_at	42.62747	20.25053	2.105005	AT3G13060.1	4.00E-37	ECT5; FUNCTIONS IN: molecular_function unknown; INV
Cit.35968.1.S1_s_at	63.1927	30.02115	2.104939	AT1G23390.1	4.00E-66	kelch repeat-containing F-box family protein
Cit.5722.1.S1_at	43.3024	20.57476	2.104637	AT3G48690.1	1.00E-95	CXE12; carboxylesterase
Cit.6191.1.S1_x_at	479.8465	228.0559	2.104074	AT3G48425.1	1.00E-164	endonuclease/exonuclease/phosphatase family protein
Cit.48.1.S1_x_at	62.46201	29.70881	2.102474		NA	
Cit.39984.1.S1_at	118.1944	56.2335	2.10185		NA	
Cit.38047.1.S1_at	115.3946	54.90483	2.10172		NA	
Cit.17226.1.S1_at	420.4537	200.1142	2.101069	AT1G73320.1	3.00E-99	FUNCTIONS IN: molecular_function unknown; INVOLVED
Cit.29579.1.S1_at	48.215	22.97141	2.098913	AT4G25650.2	1.00E-56	ACD1-LIKE (ACD1-LIKE); 2 iron, 2 sulfur cluster binding /
Cit.30302.1.S1_at	64.55301	30.75867	2.098693	AT5G59500.1	5.00E-22	unknown protein
Cit.22526.1.S1_at	47.94204	22.85667	2.097508	AT2G36460.2	8.00E-17	fructose-bisphosphate aldolase, putative
Cit.10454.1.S1_at	81.85104	39.02369	2.097471		NA	
Cit.21098.1.S1_s_at	75.47066	36.01686	2.095426	AT4G23840.1	4.00E-88	leucine-rich repeat family protein
Cit.16236.1.S1_at	45.83897	21.90936	2.092209	AT4G22990.1	1.00E-156	SPX (SYG1/Pho81/XPR1) domain-containing protein
Cit.26748.1.S1_s_at	739.4346	353.4956	2.091779	AT1G15750.4	0	TPL (TOPLESS); protein binding / protein homodimerizat
Cit.38994.1.S1_at	147.6646	70.73	2.087722	AT1G44446.3	1.00E-123	CH1 (CHLORINA 1); chlorophyllide a oxygenase
Cit.9570.1.S1_at	43.35767	20.77039	2.087475	AT5G64260.1	1.00E-125	EXL2 (EXORDIUM LIKE 2)
Cit.24380.1.S1_at	53.45928	25.61005	2.087434	AT5G16970.1	1.00E-46	AT-AER (alkenal reductase); 2-alkenal reductase
Cit.40145.1.S1_at	686.4992	328.961	2.086871		NA	
Cit.13406.1.S1_at	57.22919	27.43282	2.086158	AT5G61380.1	2.00E-28	TOC1 (TIMING OF CAB EXPRESSION 1); transcription regu
Cit.1746.1.S1_at	67.25781	32.31399	2.081384	AT1G08970.2	9.00E-31	NF-YC9 (NUCLEAR FACTOR Y, SUBUNIT C9); DNA binding
Cit.13202.1.S1_at	57.28627	27.56182	2.078465	AT1G77300.1	1.00E-24	EFS (EARLY FLOWERING IN SHORT DAYS); histone methy
Cit.24784.1.S1_x_at	56.46468	27.17817	2.077575	AT1G71695.1	5.00E-57	peroxidase 12 (PER12) (P12) (PRXR6)
Cit.28584.1.S1_at	115.1786	55.47926	2.076066	AT3G23900.2	4.00E-40	RNA recognition motif (RRM)-containing protein
Cit.594.1.S1_at	129.3711	62.33559	2.075397		NA	
Cit.10345.1.S1_x_at	6796.189	3276.488	2.07423	AT2G30290.1	4.00E-58	vacuolar sorting receptor, putative
Cit.37488.1.S1_at	92.95808	44.85205	2.072549		NA	
Cit.8750.1.S1_at	68.9061	33.24835	2.072467	AT3G61110.1	4.00E-45	ARS27A (ARABIDOPSIS RIBOSOMAL PROTEIN S27); struct
Cit.3195.1.S1_s_at	275.6997	133.0397	2.072311	AT1G33055.1	7.00E-13	unknown protein
Cit.18565.1.S1_at	150.8486	72.89188	2.069484	AT2G38630.1	1.00E-19	FUNCTIONS IN: molecular_function unknown; INVOLVED

Cit.2874.1.S1_at	92.57858	44.7537	2.068624	AT5G18420.3	2.00E-23	unknown protein
Cit.30506.1.S1_s_at	618.1838	298.841	2.068604	AT5G01600.1	3.00E-92	ATFER1; ferric iron binding / iron ion binding
Cit.20636.1.S1_at	171.2558	82.82307	2.067731		NA	
Cit.15993.1.S1_at	47.90688	23.17049	2.067582	AT1G25390.1	1.00E-86	protein kinase family protein
Cit.10279.1.S1_at	67.12412	32.48424	2.06636	AT3G57520.1	0	AtSIP2 (Arabidopsis thaliana seed imbibition 2); hydrolas
Cit.29784.1.S1_s_at	227.6169	110.3057	2.06351	AT5G07120.1	8.00E-80	SNX2b (SORTING NEXIN 2b); phosphoinositide binding /
Cit.32765.1.S1_at	126.4832	61.43694	2.058748	AT1G79790.1	1.00E-29	haloacid dehalogenase-like hydrolase family protein
Cit.28132.1.S1_at	175.3647	85.19002	2.058512	AT5G09920.1	2.00E-11	NRPB4; DNA-directed RNA polymerase
Cit.25708.1.S1_s_at	64.03764	31.11486	2.058105		NA	
Cit.35448.1.S1_at	56.56119	27.50414	2.056461		NA	
Cit.24347.1.S1_x_at	61.14248	29.7386	2.055997	AT4G14360.2	3.00E-26	dehydration-responsive protein-related
Cit.3224.1.S1_s_at	156.6571	76.34682	2.051914	AT1G52140.1	2.00E-37	unknown protein
Cit.20449.1.S1_at	110.4716	53.91027	2.049175		NA	
Cit.16427.1.S1_at	46.94127	22.90797	2.049124	AT1G78955.1	6.00E-90	CAMS1 (Camelliol C synthase 1); beta-amyrin synthase
Cit.25990.1.S1_at	843.2372	411.7461	2.047954	AT1G49640.1	5.00E-06	hydrolase
Cit.32846.1.S1_at	100.0456	48.91499	2.045295		NA	
Cit.31938.1.S1_s_at	126.7277	61.99431	2.044183	AT5G52030.2	1.00E-107	TraB protein-related
Cit.15311.1.S1_at	67.82117	33.20803	2.042312		NA	
Cit.38329.1.S1_at	49.48288	24.23373	2.041901	AT5G10600.1	5.00E-39	CYP81K2; electron carrier/ heme binding / iron ion bindi
Cit.10213.1.S1_at	59.77427	29.27408	2.041884	AT1G10200.1	2.00E-87	WLIM1; transcription factor/ zinc ion binding
Cit.11641.1.S1_at	51.26745	25.15143	2.038351	AT5G58710.1	7.00E-83	ROC7; peptidyl-prolyl cis-trans isomerase
Cit.38589.1.S1_at	206.188	101.1708	2.038019	AT5G44280.1	5.00E-70	RING1A (RING 1A); protein binding / zinc ion binding
Cit.3195.1.S1_at	112.6527	55.28268	2.037758	AT1G33055.1	7.00E-13	unknown protein
Cit.17405.1.S1_s_at	132.1735	64.86234	2.037754	AT5G03540.1	3.00E-74	ATEXO70A1 (exocyst subunit EXO70 family protein A1); p
Cit.8786.1.S1_at	124.981	61.35646	2.036966	AT1G05850.1	1.00E-150	POM1 (POM-POM1); chitinase
Cit.25226.1.S1_at	45.13447	22.17461	2.035412	AT3G42170.1	2.00E-11	DNA binding
Cit.14774.1.S1_at	72.82762	35.81894	2.033215	AT2G31340.1	9.00E-88	emb1381 (embryo defective 1381)
Cit.3005.1.S1_s_at	797.3472	392.2568	2.032717	AT5G10140.2	4.00E-35	FLC (FLOWERING LOCUS C); specific transcriptional repre
Cit.7916.1.S1_at	65.11386	32.05141	2.031544	AT4G12600.1	7.00E-53	ribosomal protein L7Ae/L30e/S12e/Gadd45 family prote
Cit.28686.1.S1_at	79.47354	39.13935	2.030528	AT3G23600.2	1.00E-13	dienelactone hydrolase family protein
Cit.35081.1.S1_s_at	90.47189	44.55978	2.030349	AT4G16380.1	3.00E-11	metal ion binding
Cit.1727.1.S1_s_at	44.25795	21.81064	2.029191	AT3G54420.1	6.00E-76	ATEP3; chitinase
Cit.21931.1.S1_at	109.3182	53.88321	2.028799	AT3G19670.1	1.00E-26	protein binding
Cit.17438.1.S1_at	63.67311	31.38809	2.028575	AT1G14870.1	1.00E-55	FUNCTIONS IN: molecular_function unknown; INVOLVED
Cit.8210.1.S1_x_at	186.6147	92.0181	2.028022	AT1G54410.1	1.00E-19	dehydrin family protein

Cit.35626.1.S1_at	110.1268	54.34856	2.026306	AT1G14370.1	3.00E-81	APK2A (PROTEIN KINASE 2A); ATP binding / kinase/ prot
Cit.39140.1.S1_at	311.4316	153.7016	2.026209	AT1G18030.2	7.00E-41	protein phosphatase 2C, putative / PP2C, putative
Cit.15535.1.S1_at	54.26553	26.78262	2.026147	AT3G59820.2	5.00E-48	calcium-binding mitochondrial protein-related
Cit.34156.1.S1_at	128.9658	63.71705	2.024039		NA	
Cit.26918.1.S1_at	56.18369	27.76532	2.02352	AT2G45010.1	2.00E-10	unknown protein
Cit.3290.1.S1_at	167.0975	82.59486	2.023098	AT1G06290.1	1.00E-61	ACX3 (ACYL-COA OXIDASE 3); acyl-CoA oxidase
Cit.25772.1.S1_at	123.9059	61.25517	2.022783	AT5G49220.1	1.00E-11	unknown protein
Cit.23978.1.S1_x_at	42.07975	20.80993	2.0221	AT4G24220.2	1.00E-47	VEP1 (VEIN PATTERNING 1); binding / catalytic
Cit.17517.1.S1_at	47.33899	23.4141	2.021815	AT5G13580.1	8.00E-77	ABC transporter family protein
Cit.17999.1.S1_s_at	166.8248	82.5312	2.021354	AT4G15630.1	2.00E-49	integral membrane family protein
Cit.31458.1.S1_at	154.9022	76.63409	2.021322	AT1G47720.1	3.00E-11	OSB1 (Organellar Single-stranded); single-stranded DNA
Cit.38878.1.S1_at	47.35937	23.43262	2.021087		NA	
Cit.21508.1.S1_x_at	268.5483	132.8842	2.02092		NA	
Cit.16592.1.S1_at	57.79343	28.59838	2.020864		NA	
Cit.11148.1.S1_s_at	80.21587	39.71307	2.019886	AT1G68530.1	0	KCS6 (3-KETOACYL-COA SYNTHASE 6); catalytic/ transfer
Cit.23187.1.S1_x_at	53.08175	26.37399	2.012655		NA	
Cit.14138.1.S1_at	75.76388	37.66615	2.011458	AT4G17830.1	2.00E-23	peptidase M20/M25/M40 family protein
Cit.23718.1.S1_x_at	64.1498	31.8926	2.011432	AT1G53310.3	6.00E-24	ATPPC1 (PHOSPHOENOLPYRUVATE CARBOXYLASE 1); ca
Cit.26238.1.S1_x_at	66.52313	33.09649	2.009975		NA	
Cit.19584.1.S1_at	115.5568	57.55615	2.007723	AT3G18350.1	4.00E-54	unknown protein
Cit.6784.1.S1_at	106.1695	52.9067	2.006731	AT5G46560.1	1.00E-103	unknown protein
Cit.25921.1.S1_at	47.49131	23.69048	2.004658	AT5G43470.2	1.00E-11	RPP8 (RECOGNITION OF PERONOSPORA PARASITICA 8);
Cit.30041.1.S1_at	65.78871	32.82161	2.004433	AT3G57300.1	1.00E-149	INO80 (INO80 ORTHOLOG); ATP binding / DNA binding /
Cit.30964.1.S1_at	81.68549	40.82041	2.001094	AT5G63640.1	2.00E-45	VHS domain-containing protein / GAT domain-containin
Cit.25803.1.S1_at	20.19313	40.34504	-1.99796	AT5G44180.1	1.00E-53	homeobox transcription factor, putative
Cit.11854.1.S1_at	78.90768	157.9416	-2.0016	AT5G05340.1	1.00E-103	peroxidase, putative
Cit.38876.1.S1_at	73.63036	147.5295	-2.00365	AT3G51930.1	2.00E-24	transducin family protein / WD-40 repeat family protein
Cit.14916.1.S1_at	36.77697	73.73529	-2.00493	AT1G17020.1	3.00E-90	SRG1 (SENESCENCE-RELATED GENE 1); oxidoreductase,
Cit.20287.1.S1_at	582.9764	1171.067	-2.00877		NA	
Cit.14533.1.S1_at	95.47974	191.9515	-2.01039	AT1G20823.1	1.00E-56	zinc finger (C3HC4-type RING finger) family protein
Cit.25635.1.S1_at	40.05983	80.56779	-2.01119	AT3G15353.1	8.00E-18	MT3 (METALLOTHIONEIN 3); copper ion binding
Cit.6686.1.S1_at	29.51233	59.39705	-2.01262	AT5G47740.1	1.00E-34	FUNCTIONS IN: molecular_function unknown; INVOLVE
Cit.12839.1.S1_s_at	153.0441	308.1142	-2.01324	AT3G07350.1	1.00E-78	unknown protein
Cit.24915.1.S1_at	28.71391	57.8524	-2.01479		NA	
Cit.26549.1.S1_at	25.46707	51.3714	-2.01717	AT5G43330.1	3.00E-45	malate dehydrogenase, cytosolic, putative

Cit.21496.1.S1_at	338.1537	682.2084	-2.01745		NA	
Cit.28487.1.S1_at	29.93414	60.40722	-2.018	AT3G14460.1	2.00E-05	disease resistance protein (NBS-LRR class), putative
Cit.1710.1.S1_s_at	725.4114	1463.999	-2.01816	AT1G74450.1	3.00E-11	unknown protein
Cit.25576.1.S1_s_at	35.03849	70.76293	-2.01958	AT3G23580.1	1.00E-162	RNR2A (RIBONUCLEOTIDE REDUCTASE 2A); ribonucleosi
Cit.23198.1.S1_at	43.50667	87.8743	-2.01979	AT5G25820.1	7.00E-52	exostosin family protein
Cit.28173.1.S1_s_at	36.56322	73.8883	-2.02084	AT4G21380.1	1.00E-106	ARK3 (A. THALIANA RECEPTOR KINASE 3); kinase/ transm
Cit.14258.1.S1_at	222.4552	450.5319	-2.02527		NA	
Cit.5398.1.S1_at	64.02357	129.7578	-2.02672	AT4G01280.1	4.00E-29	myb family transcription factor
Cit.24953.1.S1_at	65.6895	133.1521	-2.02699	AT4G33460.1	1.00E-79	ATNAP13; transporter
Cit.24414.1.S1_at	44.76788	90.74601	-2.02703	AT5G38710.1	4.00E-36	proline oxidase, putative / osmotic stress-responsive pro
Cit.27441.1.S1_at	26.11734	52.94241	-2.0271		NA	
Cit.16606.1.S1_at	71.31573	144.6161	-2.02783	AT5G57070.1	3.00E-05	hydroxyproline-rich glycoprotein family protein
Cit.9464.1.S1_at	169.7498	344.8816	-2.03171	AT2G29500.1	1.00E-56	17.6 kDa class I small heat shock protein (HSP17.6B-CI)
Cit.6619.1.S1_at	25.17932	51.167	-2.0321	AT3G48470.1	1.00E-101	EMB2423 (EMBRYO DEFECTIVE 2423)
Cit.29856.1.S1_at	32.64585	66.36313	-2.03282	AT5G53770.1	2.00E-41	nucleotidyltransferase family protein
Cit.2598.1.S1_at	219.6598	446.5514	-2.03292	AT1G75510.1	5.00E-97	transcription initiation factor IIF beta subunit (TFIIF-beta
Cit.758.1.S1_at	107.2906	218.407	-2.03566		NA	
Cit.17492.1.S1_at	29.34076	59.83789	-2.03941		NA	
Cit.7941.1.S1_at	175.8561	359.0173	-2.04154		NA	
Cit.18579.1.S1_at	47.80729	97.60435	-2.04162		NA	
Cit.29729.1.S1_at	24.74312	50.51805	-2.0417	AT2G16700.1	3.00E-68	ADF5 (ACTIN DEPOLYMERIZING FACTOR 5); actin binding
Cit.8302.1.S1_x_at	44.68585	91.29591	-2.04306		NA	
Cit.27433.1.S1_at	31.40864	64.17189	-2.04313	AT1G29040.3	7.00E-19	unknown protein
Cit.29444.1.S1_at	108.5835	222.0139	-2.04464	AT4G31180.2	1.00E-55	aspartyl-tRNA synthetase, putative / aspartate--tRNA lig
Cit.14175.1.S1_x_at	25.44916	52.07111	-2.04608	AT4G26570.2	4.00E-39	ATCBL3 (ARABIDOPSIS THALIANA CALCINEURIN B-LIKE 3)
Cit.5425.1.S1_at	273.7991	560.2224	-2.04611	AT3G19970.1	2.00E-62	unknown protein
Cit.15006.1.S1_at	141.7066	290.4478	-2.04964	AT4G38540.1	2.00E-81	monooxygenase, putative (MO2)
Cit.1814.1.S1_s_at	29.33726	60.13329	-2.04972	AT3G26210.1	1.00E-108	CYP71B23; electron carrier/ heme binding / iron ion binc
Cit.31416.1.S1_at	84.97749	174.2206	-2.0502		NA	
Cit.6664.1.S1_at	189.2985	388.5267	-2.05246	AT4G03110.1	1.00E-172	RNA-binding protein, putative
Cit.7076.1.S1_at	28.0814	57.6507	-2.05299	AT2G26160.1	4.00E-12	FUNCTIONS IN: molecular_function unknown; INVOLVED
Cit.22023.1.S1_x_at	502.2801	1031.242	-2.05312		NA	
Cit.6097.1.S1_at	46.20002	94.87904	-2.05366	AT2G42010.1	0	PLDBETA1 (PHOSPHOLIPASE D BETA 1); phospholipase D
Cit.30535.1.S1_s_at	42.4335	87.18905	-2.05472	AT1G05010.1	1.00E-148	EFE (ETHYLENE-FORMING ENZYME); 1-aminocyclopropa
Cit.7660.1.S1_at	25.97758	53.43985	-2.05715	AT1G12290.2	7.00E-14	disease resistance protein (CC-NBS-LRR class), putative

Cit.38882.1.S1_at	60.64014	125.2293	-2.06512		NA	
Cit.4136.1.S1_at	124.629	257.4227	-2.06551	AT4G23630.1	8.00E-92	BTI1 (VIRB2-INTERACTING PROTEIN 1)
Cit.19606.1.S1_at	85.79323	177.3657	-2.06736		NA	
Cit.22154.1.S1_at	21.13509	43.72986	-2.06906	AT1G27330.1	2.00E-18	FUNCTIONS IN: molecular_function unknown; INVOLVEI
Cit.7237.1.S1_at	161.3735	334.0312	-2.06993	AT5G01210.1	8.00E-74	transferase family protein
Cit.22286.1.S1_x_at	1628.901	3380.168	-2.07512	AT5G07090.2	2.00E-10	40S ribosomal protein S4 (RPS4B)
Cit.23417.1.S1_at	55.46952	115.116	-2.0753	AT3G32930.1	4.00E-58	unknown protein
Cit.24033.1.S1_at	41.08781	85.31477	-2.0764	AT5G19875.1	6.00E-32	unknown protein
Cit.3442.1.S1_at	22.62353	46.9886	-2.07698		NA	
Cit.18512.1.S1_at	32.71026	67.95233	-2.0774		NA	
Cit.23610.1.S1_at	286.4233	595.2152	-2.0781		NA	
Cit.1802.1.S1_at	48.02429	99.96877	-2.08163	AT4G12590.1	1.00E-124	unknown protein
Cit.24185.1.S1_s_at	204.7374	426.7084	-2.08417	AT3G54660.1	0	GR (GLUTATHIONE REDUCTASE); ATP binding / glutathio
Cit.12819.1.S1_at	153.5096	319.954	-2.08426	AT2G46210.1	1.00E-107	delta-8 sphingolipid desaturase, putative
Cit.26445.1.S1_at	75.64655	157.7603	-2.08549	AT4G17420.1	3.00E-45	FUNCTIONS IN: molecular_function unknown; INVOLVEI
Cit.12535.1.S1_at	767.0111	1601.372	-2.08781	AT5G59010.2	6.00E-94	protein kinase-related
Cit.14989.1.S1_at	21.86632	45.66815	-2.08852	AT2G16790.1	6.00E-56	shikimate kinase family protein
Cit.18281.1.S1_at	132.3631	276.4466	-2.08855		NA	
Cit.24582.1.S1_at	74.83742	156.3453	-2.08913		NA	
Cit.34811.1.S1_at	24.55421	51.31124	-2.08971	AT4G34950.1	3.00E-41	nodulin family protein
Cit.14635.1.S1_at	179.541	375.52	-2.09156	AT1G74210.1	4.00E-69	glycerophosphoryl diester phosphodiesterase family pro
Cit.3236.1.S1_at	79.96539	167.2831	-2.09194	AT5G39670.1	1.00E-40	calcium-binding EF hand family protein
Cit.2151.1.S1_at	81.35719	170.5371	-2.09615		NA	
Cit.18017.1.S1_s_at	60.5124	127.1623	-2.10143	AT1G72520.1	0	lipoxygenase, putative
Cit.13936.1.S1_at	49.60981	104.2896	-2.1022		NA	
Cit.16673.1.S1_at	137.6612	289.5146	-2.1031		NA	
Cit.27211.1.S1_at	36.07893	75.96249	-2.10545	AT1G25350.1	4.00E-63	OVA9 (ovule abortion 9); glutamine-tRNA ligase
Cit.17294.1.S1_at	86.33691	181.9421	-2.10735		NA	
Cit.6026.1.S1_at	29.22341	61.64516	-2.10944	AT1G65420.1	5.00E-56	unknown protein
Cit.29007.1.S1_at	20.18141	42.57539	-2.10963	AT3G02885.1	3.00E-36	GASA5 (GAST1 PROTEIN HOMOLOG 5)
Cit.17308.1.S1_at	91.00047	191.9843	-2.10971		NA	
Cit.1861.1.S1_s_at	805.1543	1701.049	-2.1127	AT2G27080.2	2.00E-70	harpin-induced protein-related / HIN1-related / harpin-r
Cit.23023.1.S1_at	118.8975	251.297	-2.11356	AT1G75660.1	2.00E-05	XRN3; 5'-3' exoribonuclease
Cit.26086.1.S1_at	31.89212	67.41608	-2.11388	AT5G26990.1	8.00E-05	drought-responsive family protein
Cit.15474.1.S1_at	31.64915	67.17275	-2.12242	AT3G27930.1	5.00E-65	unknown protein

Cit.18105.1.S1_s_at	279.9489	594.5616	-2.12382	AT5G42380.1	6.00E-29	CML37 (CALMODULIN LIKE 37); calcium ion binding
Cit.26711.1.S1_at	58.88657	125.0875	-2.12421	AT3G04450.1	6.00E-07	transcription factor
Cit.18283.1.S1_at	49.14019	104.4125	-2.12479		NA	
Cit.15773.1.S1_at	27.24485	57.91159	-2.1256		NA	
Cit.25997.1.S1_x_at	178.1769	378.9716	-2.12694	AT5G05270.2	7.00E-13	chalcone-flavanone isomerase family protein
Cit.4718.1.S1_at	81.3747	173.2904	-2.12954	AT1G18580.1	7.00E-68	GAUT11 (Galacturonosyltransferase 11); polygalacturona
Cit.17520.1.S1_at	336.113	715.8159	-2.12969	AT2G30490.1	0	C4H (CINNAMATE-4-HYDROXYLASE); trans-cinnamate 4-
Cit.29596.1.S1_at	183.917	391.7098	-2.12982		NA	
Cit.542.1.S1_at	71.31078	151.8904	-2.12998		NA	
Cit.31294.1.S1_at	74.23038	158.1654	-2.13074	AT1G11700.1	1.00E-13	unknown protein
Cit.22434.1.S1_at	148.2114	316.5397	-2.13573		NA	
Cit.21789.1.S1_at	53.54807	114.6026	-2.14018		NA	
Cit.26228.1.S1_at	28.16001	60.36009	-2.14347	AT1G75580.1	2.00E-37	auxin-responsive protein, putative
Cit.18728.1.S1_x_at	59.58825	127.727	-2.14349		NA	
Cit.12843.1.S1_at	63.41637	136.0872	-2.14593		NA	
Cit.11042.1.S1_at	38.53914	82.89449	-2.15092	AT1G10430.1	1.00E-179	PP2A-2; protein serine/threonine phosphatase
Cit.11055.1.S1_s_at	2199.947	4734.328	-2.15202	AT4G35750.1	9.00E-87	Rho-GTPase-activating protein-related
Cit.32102.1.S1_at	86.08079	185.2548	-2.1521	AT5G63020.1	1.00E-07	disease resistance protein (CC-NBS-LRR class), putative
Cit.21169.1.S1_at	45.53857	98.05875	-2.15331		NA	
Cit.17958.1.S1_at	115.04	247.7677	-2.15375	AT5G09830.1	2.00E-33	BolA-like family protein
Cit.29721.1.S1_at	256.6095	552.9552	-2.15485	AT4G10270.1	3.00E-21	wound-responsive family protein
Cit.24745.1.S1_at	30.99618	66.94677	-2.15984	AT5G54960.1	2.00E-07	PDC2 (pyruvate decarboxylase-2); carboxy-lyase/ catalyt
Cit.16885.1.S1_at	138.9114	300.5158	-2.16336	AT1G66400.1	1.00E-43	calmodulin-related protein, putative
Cit.25910.1.S1_x_at	907.8419	1966.075	-2.16566		NA	
Cit.7227.1.S1_at	22.98593	49.86622	-2.16942	AT2G42680.1	3.00E-15	MBF1A (MULTIPROTEIN BRIDGING FACTOR 1A); DNA bir
Cit.14081.1.S1_at	213.2151	462.6121	-2.1697	AT2G31880.1	1.00E-118	leucine-rich repeat transmembrane protein kinase, puta
Cit.17845.1.S1_at	49.13896	106.7458	-2.17233		NA	
Cit.19326.1.S1_at	624.5177	1356.855	-2.17264	AT1G27650.2	8.00E-09	ATU2AF35A; RNA binding / nucleic acid binding / nucleo
Cit.12237.1.S1_at	434.9232	946.584	-2.17644	AT2G17410.1	2.00E-87	ARID/BRIGHT DNA-binding domain-containing protein
Cit.28144.1.S1_at	177.7547	388.5969	-2.18614	AT1G17020.1	3.00E-63	SRG1 (SENESCENCE-RELATED GENE 1); oxidoreductase, a
Cit.11296.1.S1_at	232.4689	508.3324	-2.18667	AT5G37540.1	2.00E-87	aspartyl protease family protein
Cit.17243.1.S1_at	115.1326	251.7581	-2.18668	AT1G32920.1	3.00E-07	unknown protein
Cit.28077.1.S1_at	143.7993	314.7823	-2.18904	AT5G19460.1	1.00E-22	atnudt20 (Arabidopsis thaliana Nudix hydrolase homolog
Cit.33468.1.S1_at	166.3077	364.9987	-2.19472	AT5G54760.2	9.00E-10	eukaryotic translation initiation factor SUI1, putative
Cit.22642.1.S1_at	33.20168	72.91422	-2.1961	AT4G15520.1	2.00E-18	tRNA/rRNA methyltransferase (SpoU) family protein

Cit.4259.1.S1_at	85.67736	188.3102	-2.1979	AT1G14870.1	3.00E-52	FUNCTIONS IN: molecular_function unknown; INVOLVED
Cit.18535.1.S1_at	545.1255	1200.331	-2.20194	AT2G34250.2	2.00E-13	protein transport protein sec61, putative
Cit.25373.1.S1_s_at	376.685	829.5624	-2.20227	AT4G05150.1	1.00E-104	octicosapeptide/Phox/Bem1p (PB1) domain-containing p
Cit.14040.1.S1_at	25.59831	56.39516	-2.20308	AT5G59540.1	4.00E-46	oxidoreductase, 2OG-Fe(II) oxygenase family protein
Cit.30908.1.S1_at	24.90085	54.88213	-2.20403		NA	
Cit.272.1.S1_at	69.35123	153.0529	-2.20692	AT2G33120.1	1.00E-104	SAR1 (SYNAPTOBREVIN-RELATED PROTEIN 1)
Cit.12938.1.S1_at	53.31026	117.706	-2.20794	AT3G51670.1	0	SEC14 cytosolic factor family protein / phosphoglyceride
Cit.30058.1.S1_at	53.25014	117.6175	-2.20877		NA	
Cit.20124.1.S1_at	120.173	265.6427	-2.2105		NA	
Cit.27996.1.S1_at	23.20269	51.44344	-2.21713	AT2G29410.1	6.00E-05	MTPB1 (METAL TOLERANCE PROTEIN B1); efflux transme
Cit.271.1.S1_x_at	157.1118	348.5769	-2.21866	AT2G36460.1	0	fructose-bisphosphate aldolase, putative
Cit.26060.1.S1_at	76.07378	169.1559	-2.22358	AT3G22840.1	3.00E-15	ELIP1 (EARLY LIGHT-INDUCABLE PROTEIN); chlorophyll b
Cit.11448.1.S1_at	168.2923	374.8613	-2.22744	AT1G28200.1	4.00E-95	FIP1 (FH INTERACTING PROTEIN 1)
Cit.7862.1.S1_at	29.6846	66.30821	-2.23376		NA	
Cit.3523.1.S1_at	763.3273	1705.438	-2.23422	AT4G29780.1	0	unknown protein
Cit.11802.1.S1_at	38.40821	85.87264	-2.23579	AT5G24690.1	5.00E-54	INVOLVED IN: biological_process unknown; LOCATED IN
Cit.3114.1.S1_at	167.5013	374.6455	-2.23667	AT3G13930.1	1.00E-121	dihydrolipoamide S-acetyltransferase, putative
Cit.25939.1.S1_at	59.0471	132.205	-2.23898	AT2G21660.1	2.00E-49	CCR2 (COLD, CIRCADIAN RHYTHM, AND RNA BINDING 2)
Cit.3420.1.S1_at	784.5016	1758.01	-2.24093	AT2G26660.1	6.00E-87	SPX2 (SPX DOMAIN GENE 2)
Cit.19882.1.S1_x_at	102.6261	230.5528	-2.24653	AT1G28330.4	6.00E-48	DYL1 (DORMANCY-ASSOCIATED PROTEIN-LIKE 1)
Cit.5954.1.S1_at	76.19057	171.3114	-2.24846	AT3G06810.1	5.00E-70	IBR3 (IBA-RESPONSE 3); acyl-CoA dehydrogenase/ oxidor
Cit.21827.1.S1_at	105.4818	237.3789	-2.25043		NA	
Cit.23992.1.S1_at	76.00405	171.179	-2.25224	AT1G51310.1	1.00E-71	tRNA (5-methylaminomethyl-2-thiouridylate)-methyltran
Cit.13320.1.S1_at	109.6817	247.3864	-2.25549	AT3G42170.1	1.00E-98	DNA binding
Cit.32009.1.S1_at	40.9314	92.45946	-2.25889		NA	
Cit.1249.1.S1_at	22.88751	51.70672	-2.25917	AT5G43830.1	1.00E-102	unknown protein
Cit.1854.1.S1_at	40.29801	91.22136	-2.26367		NA	
Cit.9873.1.S1_at	74.28339	168.4682	-2.26791	AT4G32330.3	4.00E-12	FUNCTIONS IN: molecular_function unknown; INVOLVED
Cit.2677.1.S1_at	219.58	500.4244	-2.27901	AT5G51190.1	2.00E-48	AP2 domain-containing transcription factor, putative
Cit.23215.1.S1_at	21.87206	49.89495	-2.28122	AT1G65840.1	6.00E-35	ATPAO4 (ARABIDOPSIS THALIANA POLYAMINE OXIDASE
Cit.17450.1.S1_s_at	565.4639	1291.544	-2.28404	AT2G46330.1	5.00E-13	AGP16 (ARABINOGALACTAN PROTEIN 16)
Cit.23534.1.S1_at	82.80775	189.6873	-2.2907	AT1G15110.1	3.00E-68	phosphatidyl serine synthase family protein
Cit.1883.1.S1_at	22.45582	51.44556	-2.29097	AT4G35160.1	5.00E-45	O-methyltransferase family 2 protein
Cit.8720.1.S1_at	69.81171	160.3404	-2.29676	AT5G54160.1	7.00E-35	ATOMT1 (O-METHYLTRANSFERASE 1); caffeate O-methy
Cit.4694.1.S1_at	28.43005	65.29904	-2.29683	AT3G46130.1	7.00E-76	MYB111 (MYB DOMAIN PROTEIN 111); DNA binding / tra

Cit.11796.1.S1_s_at	81.6647	187.6807	-2.29819	AT3G22750.1	4.00E-84	protein kinase, putative
Cit.5550.1.S1_at	24.19099	55.64743	-2.30034		NA	
Cit.3817.1.S1_at	111.3463	256.2777	-2.30163	AT4G22920.1	1.00E-99	NYE1 (NON-YELLOWING 1)
Cit.10258.1.S1_s_at	70.74204	163.3037	-2.30844	AT1G32928.1	5.00E-09	unknown protein
Cit.5470.1.S1_at	22.86518	52.79651	-2.30904		NA	
Cit.28591.1.S1_at	195.826	452.3628	-2.31002	AT1G75500.1	2.00E-40	nodulin MtN21 family protein
Cit.4633.1.S1_at	252.8115	584.338	-2.31136	AT3G22260.1	2.00E-97	OTU-like cysteine protease family protein
Cit.26571.1.S1_at	124.0841	286.8185	-2.31148		NA	
Cit.37513.1.S1_at	71.09881	164.5049	-2.31375	AT3G05410.2	4.00E-81	calcium ion binding
Cit.32256.1.S1_at	20.2276	46.85952	-2.31661	AT4G09680.1	5.00E-09	unknown protein
Cit.6967.1.S1_at	31.61604	73.39576	-2.32147	AT1G38065.1	5.00E-11	unknown protein
Cit.15055.1.S1_at	35.997	83.69971	-2.32519	AT4G13690.1	1.00E-31	unknown protein
Cit.19543.1.S1_at	187.5742	436.1571	-2.32525	AT4G37870.1	3.00E-18	PCK1 (PHOSPHOENOLPYRUVATE CARBOXYKINASE 1); AT
Cit.21392.1.S1_at	123.8866	288.2964	-2.3271		NA	
Cit.21043.1.S1_at	69.23151	161.2468	-2.3291		NA	
Cit.2638.1.S1_x_at	36.07867	84.1191	-2.33155		NA	
Cit.23049.1.S1_at	222.3637	518.4764	-2.33166		NA	
Cit.21723.1.S1_s_at	57.15314	133.526	-2.33628	AT1G05010.1	1.00E-148	EFE (ETHYLENE-FORMING ENZYME); 1-aminocyclopropa
Cit.14030.1.S1_at	39.69078	92.8447	-2.3392	AT5G45290.1	2.00E-49	zinc finger (C3HC4-type RING finger) family protein
Cit.22023.1.S1_s_at	322.2843	755.0744	-2.34288	AT5G01710.1	1.00E-133	LOCATED IN: endomembrane system; EXPRESSED IN: sp
Cit.335.1.S1_at	20.37778	47.80568	-2.34597	AT1G49660.1	1.00E-65	AtCXE5 (Arabidopsis thaliana carboxyesterase 5); carbox
Cit.30629.1.S1_at	20.91107	49.14057	-2.34998	AT2G43790.1	1.00E-143	ATMPK6 (ARABIDOPSIS THALIANA MAP KINASE 6); MAP
Cit.29173.1.S1_s_at	31.53395	74.15654	-2.35164	AT3G09270.1	2.00E-49	ATGSTU8 (GLUTATHIONE S-TRANSFERASE TAU 8); glutat
Cit.9788.1.S1_at	52.84604	124.7572	-2.36077	AT2G14910.1	1.00E-121	unknown protein
Cit.9662.1.S1_at	28.91964	68.27524	-2.36086	AT5G16050.1	1.00E-126	GRF5 (GENERAL REGULATORY FACTOR 5); ATP binding /
Cit.19642.1.S1_s_at	526.4222	1246.301	-2.36749		NA	
Cit.32782.1.S1_at	24.25089	57.44921	-2.36895	AT3G10300.2	9.00E-26	calcium-binding EF hand family protein
Cit.30768.1.S1_at	32.40719	76.79117	-2.36957	AT2G42880.1	8.00E-30	ATMPK20; MAP kinase
Cit.9052.1.S1_at	28.27376	67.30481	-2.38047	AT3G46020.1	9.00E-27	RNA-binding protein, putative
Cit.4550.1.S1_at	343.0085	817.4229	-2.3831	AT1G05000.1	6.00E-85	tyrosine specific protein phosphatase family protein
Cit.6154.1.S1_at	21.26028	50.70174	-2.38481		NA	
Cit.15428.1.S1_at	21.74428	51.87355	-2.38562	AT1G18940.1	6.00E-46	nodulin family protein
Cit.7869.1.S1_at	38.01136	90.68125	-2.38564	AT1G48050.1	1.00E-11	KU80; double-stranded DNA binding / protein binding
Cit.17706.1.S1_at	25.18547	60.67435	-2.4091	AT5G58240.1	2.00E-22	bis(5'-adenosyl)-triphosphatase, putative
Cit.31495.1.S1_at	40.11378	97.37435	-2.42745	AT2G28290.1	1.00E-07	SYD (SPLAYED); ATPase/ chromatin binding

Cit.20542.1.S1_at	57.24516	138.9935	-2.42804	AT3G59280.1	1.00E-26	TXR1 (THAXTOMIN A RESISTANT 1)
Cit.22493.1.S1_at	348.4484	847.1711	-2.43127	AT1G09020.1	5.00E-36	SNF4 (HOMOLOG OF YEAST SUCROSE NONFERMENTING
Cit.26596.1.S1_x_at	490.7793	1194.281	-2.43344	AT3G15353.1	8.00E-18	MT3 (METALLOTHIONEIN 3); copper ion binding
Cit.9086.1.S1_at	135.3729	330.7108	-2.44296	AT2G45550.1	1.00E-128	CYP76C4; electron carrier/ heme binding / iron ion bindi
Cit.30830.1.S1_at	21.16422	51.87833	-2.45123		NA	
Cit.25461.1.S1_at	28.90214	70.91953	-2.45378	AT1G73930.2	2.00E-52	unknown protein
Cit.15649.1.S1_at	50.75529	124.6256	-2.45542	AT4G25490.1	1.00E-53	CBF1 (C-REPEAT/DRE BINDING FACTOR 1); DNA binding ,
Cit.36666.1.S1_at	32.31129	79.38334	-2.45683		NA	
Cit.19105.1.S1_at	47.03608	115.8387	-2.46276	AT2G44840.1	3.00E-14	ERF13 (ETHYLENE-RESPONSIVE ELEMENT BINDING FACT
Cit.20328.1.S1_x_at	43.56147	107.3016	-2.46322		NA	
Cit.12432.1.S1_at	27.04535	66.73377	-2.46748	AT4G19840.1	1.00E-36	ATPP2-A1; carbohydrate binding
Cit.170.1.S1_x_at	1337.248	3307.399	-2.47329	AT1G13950.1	3.00E-77	ELF5A-1 (EUKARYOTIC ELONGATION FACTOR 5A-1); tran
Cit.21068.1.S1_at	25.08896	62.0879	-2.47471	AT2G31820.1	4.00E-27	ankyrin repeat family protein
Cit.29255.1.S1_at	25.26458	62.52469	-2.4748		NA	
Cit.20021.1.S1_at	123.1275	305.3068	-2.4796		NA	
Cit.36375.1.S1_at	24.89003	61.78182	-2.48219		NA	
Cit.40444.1.S1_at	160.8826	399.5849	-2.4837		NA	
Cit.10196.1.S1_at	34.59115	86.06751	-2.48814	AT1G48410.1	0	AGO1 (ARGONAUTE 1); endoribonuclease/ miRNA bindi
Cit.6543.1.S1_at	24.03685	60.15293	-2.50253	AT1G76810.1	2.00E-27	eukaryotic translation initiation factor 2 family protein /
Cit.7607.1.S1_at	26.4019	66.23853	-2.50885	AT3G29390.1	8.00E-11	RIK (RS2-Interacting KH protein); RNA binding
Cit.1018.1.S1_x_at	81.74158	206.2897	-2.52368	AT2G10940.2	3.00E-42	protease inhibitor/seed storage/lipid transfer protein (L
Cit.34204.1.S1_at	26.09531	65.94531	-2.52709	AT1G78660.3	5.00E-09	gamma-glutamyl hydrolase, putative / gamma-Glu-X car
Cit.6820.1.S1_at	46.64926	118.1425	-2.53257	AT5G18475.1	5.00E-87	pentatricopeptide (PPR) repeat-containing protein
Cit.32593.1.S1_at	191.2044	484.2585	-2.53267		NA	
Cit.7047.1.S1_at	79.26158	200.8666	-2.53422	AT5G01710.1	1.00E-133	LOCATED IN: endomembrane system; EXPRESSED IN: sp
Cit.15962.1.S1_at	28.58996	72.65884	-2.54141	AT4G38660.1	1.00E-101	thaumatin, putative
Cit.15093.1.S1_at	28.22592	71.79387	-2.54354	AT3G61630.1	3.00E-42	CRF6 (CYTOKININ RESPONSE FACTOR 6); DNA binding / t
Cit.31086.1.S1_at	21.12647	53.84796	-2.54884	AT5G10540.1	1.00E-17	peptidase M3 family protein / thimet oligopeptidase fan
Cit.7396.1.S1_at	22.17831	56.55376	-2.54996	AT1G01110.2	7.00E-12	IQD18 (IQ-domain 18)
Cit.4657.1.S1_at	48.29572	123.1559	-2.55004	AT3G14770.1	1.00E-85	nodulin MtN3 family protein
Cit.23912.1.S1_s_at	49.24277	125.7495	-2.55366	AT5G59730.1	1.00E-54	ATEXO70H7 (EXOCYST SUBUNIT EXO70 FAMILY PROTEIN
Cit.27240.1.S1_at	25.67883	65.606	-2.55487	AT5G48390.1	6.00E-65	ATZIP4; binding
Cit.7623.1.S1_at	20.0193	51.2925	-2.56215		NA	
Cit.26066.1.S1_at	23.22717	59.59798	-2.56587	AT2G30490.1	4.00E-05	C4H (CINNAMATE-4-HYDROXYLASE); trans-cinnamate 4-
Cit.16058.1.S1_at	142.5408	366.561	-2.57162	AT3G55370.2	2.00E-64	OBP3 (OBF-BINDING PROTEIN 3); DNA binding / transcri

Cit.26085.1.S1_at	23.85222	61.38433	-2.57353		NA	
Cit.16701.1.S1_at	42.8397	110.4461	-2.57812		NA	
Cit.24979.1.S1_x_at	246.8298	636.8793	-2.58024		NA	
Cit.16031.1.S1_at	39.39939	101.7387	-2.58224	AT3G13960.1	1.00E-12	AtGRF5 (GROWTH-REGULATING FACTOR 5); transcriptio
Cit.28472.1.S1_at	126.1665	327.5076	-2.59584	AT4G37680.1	5.00E-95	HHP4 (heptahelical protein 4); receptor
Cit.8203.1.S1_x_at	196.4973	511.2612	-2.60187	AT1G54410.1	2.00E-22	dehydrin family protein
Cit.29381.1.S1_at	24.68673	64.30623	-2.60489	AT3G53970.1	3.00E-89	proteasome inhibitor-related
Cit.21765.1.S1_s_at	339.1439	885.0483	-2.60965		NA	
Cit.12867.1.S1_at	128.7457	336.455	-2.61333	AT1G61570.1	3.00E-25	TIM13 (TRANSLOCASE OF THE INNER MITOCHONDRIAL M
Cit.19380.1.S1_at	35.61182	93.28677	-2.61955		NA	
Cit.5526.1.S1_s_at	173.8604	456.393	-2.62505	AT3G13110.1	1.00E-125	ATSERAT2;2 (SERINE ACETYLTRANSFERASE 2;2); serine O
Cit.11107.1.S1_at	33.96343	89.37244	-2.63143	AT3G25290.2	1.00E-142	auxin-responsive family protein
Cit.24408.1.S1_at	62.56613	164.7063	-2.63252	AT5G56630.1	9.00E-45	PFK7 (PHOSPHOFRUCTOKINASE 7); 6-phosphofructokina
Cit.8231.1.S1_s_at	2474.019	6558.409	-2.65091	AT2G01890.1	1.00E-131	PAP8 (PURPLE ACID PHOSPHATASE 8); acid phosphatase
Cit.24533.1.S1_at	49.27097	130.821	-2.65513		NA	
Cit.2245.1.S1_at	135.0162	359.3474	-2.66151	AT2G14170.2	1.00E-90	ALDH6B2; 3-chloroallyl aldehyde dehydrogenase/ methy
Cit.30953.1.S1_at	28.01516	74.71175	-2.66683	AT2G03210.1	5.00E-77	FUT2 (FUCOSYLTRANSFERASE 2); fucosyltransferase/ tra
Cit.30437.1.S1_s_at	182.0287	486.3909	-2.67206	AT3G05890.1	7.00E-21	RCI2B (RARE-COLD-INDUCIBLE 2B)
Cit.24979.1.S1_at	263.6285	705.2533	-2.67518		NA	
Cit.31441.1.S1_at	33.06569	88.9468	-2.69		NA	
Cit.9373.1.S1_x_at	70.32129	189.272	-2.69153	AT4G39230.1	1.00E-138	isoflavone reductase, putative
Cit.3098.1.S1_at	45.404	122.3733	-2.69521	AT5G19860.1	5.00E-27	unknown protein
Cit.28707.1.S1_at	25.49767	68.7482	-2.69625	AT1G56090.1	1.00E-19	tetratricopeptide repeat (TPR)-containing protein
Cit.27732.1.S1_at	22.58152	61.00448	-2.70152		NA	
Cit.21844.1.S1_s_at	68.96246	186.5087	-2.7045		NA	
Cit.21857.1.S1_at	100.0548	271.7956	-2.71647	AT2G45130.1	4.00E-29	SPX3 (SPX DOMAIN GENE 3)
Cit.12593.1.S1_at	245.1402	667.2275	-2.72182	AT1G52560.1	4.00E-73	26.5 kDa class I small heat shock protein-like (HSP26.5-P
Cit.9343.1.S1_x_at	38.4198	104.7112	-2.72545	AT1G11840.4	3.00E-90	ATGLX1 (GLYOXALASE I HOMOLOG); lactoylglutathione l
Cit.14057.1.S1_at	174.7523	478.3781	-2.73746	AT5G20040.1	7.00E-21	ATIPT9; ATP binding / tRNA isopentenyltransferase
Cit.18086.1.S1_at	164.283	450.7792	-2.74392	AT3G23240.1	5.00E-31	ERF1 (ETHYLENE RESPONSE FACTOR 1); DNA binding / tr
Cit.29563.1.S1_at	27.23628	74.97238	-2.75267	AT2G44600.1	6.00E-56	unknown protein
Cit.29902.1.S1_at	30.26016	83.60149	-2.76276	AT3G25950.1	8.00E-68	FUNCTIONS IN: molecular_function unknown; INVOLVED
Cit.30405.1.S1_at	20.71346	57.53738	-2.77778	AT3G43590.1	5.00E-49	zinc knuckle (CCHC-type) family protein
Cit.165.1.S1_s_at	182.3123	506.8671	-2.78021	AT3G22840.1	3.00E-56	ELIP1 (EARLY LIGHT-INDUCABLE PROTEIN); chlorophyll b
Cit.12315.1.S1_s_at	20.32321	56.52223	-2.78117	AT5G03770.1	1.00E-171	3-deoxy-D-manno-octulosonic acid transferase-related

Cit.22486.1.S1_x_at	41.71081	116.7894	-2.79998	AT4G16440.1	1.00E-25	ferredoxin hydrogenase
Cit.23567.1.S1_s_at	483.2586	1354.433	-2.80271	AT5G01220.1	0	SQD2 (sulfoquinovosyldiacylglycerol 2); UDP-glycosyltrar
Cit.24732.1.S1_at	52.92067	148.8822	-2.81331	AT5G65530.1	6.00E-06	protein kinase, putative
Cit.29608.1.S1_at	302.2613	851.3411	-2.81657		NA	
Cit.19086.1.S1_at	174.7819	494.6125	-2.82988		NA	
Cit.15198.1.S1_at	25.4774	72.12222	-2.83083	AT5G01510.1	3.00E-22	FUNCTIONS IN: molecular_function unknown; INVOLVEL
Cit.2915.1.S1_s_at	259.934	742.0435	-2.85474	AT5G63130.1	4.00E-43	octicosapeptide/Phox/Bem1p (PB1) domain-containing ı
Cit.20410.1.S1_at	54.59111	157.0737	-2.87728		NA	
Cit.7403.1.S1_at	32.02794	92.811	-2.89781		NA	
Cit.29266.1.S1_at	22.27974	64.62975	-2.90083	AT3G24160.1	4.00E-12	PMP (PUTATIVE TYPE 1 MEMBRANE PROTEIN)
Cit.21368.1.S1_at	53.24097	155.7053	-2.92454		NA	
Cit.29338.1.S1_x_at	22.13021	64.78287	-2.92735	AT3G22840.1	2.00E-14	ELIP1 (EARLY LIGHT-INDUCABLE PROTEIN); chlorophyll b
Cit.18057.1.S1_s_at	222.521	652.5595	-2.93257	AT5G11070.1	4.00E-09	unknown protein
Cit.12979.1.S1_at	23.34767	68.6164	-2.9389	AT5G66430.1	2.00E-63	S-adenosyl-L-methionine:carboxyl methyltransferase far
Cit.21033.1.S1_at	244.815	720.7944	-2.94424	AT5G24580.3	2.00E-24	copper-binding family protein
Cit.21981.1.S1_x_at	20.85756	61.66456	-2.95646	AT2G16770.1	5.00E-16	DNA binding / transcription factor
Cit.17450.1.S1_at	73.57919	218.1886	-2.96536	AT2G46330.1	9.00E-09	AGP16 (ARABINOGALACTAN PROTEIN 16)
Cit.39737.1.S1_at	20.22818	60.17162	-2.97464		NA	
Cit.4179.1.S1_at	20.23398	60.91859	-3.01071	AT1G45249.1	5.00E-49	ABF2 (ABSCISIC ACID RESPONSIVE ELEMENTS-BINDING F
Cit.35435.1.S1_x_at	1601.503	4829.816	-3.0158	AT5G39110.1	6.00E-79	germin-like protein, putative
Cit.25381.1.S1_at	49.2725	148.958	-3.02315	AT1G29820.2	1.00E-105	unknown protein
Cit.31296.1.S1_at	93.21767	281.8543	-3.02361		NA	
Cit.19362.1.S1_x_at	438.8456	1332.798	-3.03705	AT1G17860.1	8.00E-23	trypsin and protease inhibitor family protein / Kunitz fan
Cit.7624.1.S1_at	31.63802	97.02747	-3.0668		NA	
Cit.15680.1.S1_at	21.61219	67.20232	-3.10946	AT5G04660.1	7.00E-99	CYP77A4; electron carrier/ heme binding / iron ion bindi
Cit.19718.1.S1_s_at	44.68798	139.9052	-3.13071	AT2G30540.1	3.00E-40	glutaredoxin family protein
Cit.11055.1.S1_at	1018.494	3189.771	-3.13185	AT4G35750.1	9.00E-87	Rho-GTPase-activating protein-related
Cit.16962.1.S1_x_at	1467.454	4596.271	-3.13214	AT1G17860.1	1.00E-22	trypsin and protease inhibitor family protein / Kunitz fan
Cit.15121.1.S1_at	32.67744	103.3705	-3.16336	AT1G61310.1	5.00E-07	ATP binding / protein binding
Cit.20232.1.S1_s_at	184.2014	586.0125	-3.18137	AT4G35750.1	6.00E-77	Rho-GTPase-activating protein-related
Cit.13417.1.S1_s_at	78.38233	252.2716	-3.21848	AT1G08650.1	4.00E-77	PPCK1 (PHOSPHOENOLPYRUVATE CARBOXYLASE KINASE
Cit.12690.1.S1_at	32.55191	105.1493	-3.2302	AT3G13800.1	1.00E-119	metallo-beta-lactamase family protein
Cit.38274.1.S1_at	26.66344	87.47944	-3.28088		NA	
Cit.13558.1.S1_s_at	143.5185	472.7751	-3.29418	AT1G16030.1	1.00E-111	Hsp70b (heat shock protein 70B); ATP binding
Cit.6381.1.S1_at	36.02381	119.2486	-3.31027	AT4G35750.1	6.00E-77	Rho-GTPase-activating protein-related

Cit.17368.1.S1_at	49.44611	165.7156	-3.35144		NA	
Cit.4549.1.S1_s_at	83.72152	280.6124	-3.35174	AT1G05000.1	2.00E-86	tyrosine specific protein phosphatase family protein
Cit.6373.1.S1_at	47.84325	160.8259	-3.36152	AT5G48800.1	2.00E-81	phototropic-responsive NPH3 family protein
Cit.5874.1.S1_s_at	92.38711	311.486	-3.37153	AT1G08650.1	7.00E-87	PPCK1 (PHOSPHOENOLPYRUVATE CARBOXYLASE KINASE
Cit.37136.1.S1_s_at	300.72	1021.618	-3.39724	AT1G05000.1	6.00E-85	tyrosine specific protein phosphatase family protein
Cit.13636.1.S1_s_at	405.4898	1380.865	-3.40542	AT1G23110.3	2.00E-44	unknown protein
Cit.12038.1.S1_at	160.2865	547.3167	-3.41462		NA	
Cit.24933.1.S1_at	30.32696	105.5403	-3.48008	AT3G54340.1	3.00E-27	AP3 (APETALA 3); DNA binding / transcription factor
Cit.29751.1.S1_at	81.10155	285.601	-3.52152	AT5G20790.1	7.00E-17	unknown protein
Cit.19530.1.S1_at	72.05421	255.8275	-3.55049		NA	
Cit.21988.1.S1_s_at	95.41283	340.6695	-3.57048	AT5G20410.1	8.00E-74	MGD2; 1,2-diacylglycerol 3-beta-galactosyltransferase/ U
Cit.12779.1.S1_at	32.07491	115.0776	-3.58778	AT4G23810.1	3.00E-57	WRKY53; DNA binding / protein binding / transcription a
Cit.25685.1.S1_at	21.95369	79.33277	-3.61364	AT5G25880.1	2.00E-32	ATNADP-ME3 (NADP-malic enzyme 3); malate dehydrog
Cit.30163.1.S1_at	23.1425	83.76428	-3.6195	AT1G72240.1	4.00E-12	unknown protein
Cit.19566.1.S1_at	61.1194	225.6133	-3.69135		NA	
Cit.5478.1.S1_at	119.3688	443.5266	-3.7156	AT5G53050.1	1.00E-15	hydrolase, alpha/beta fold family protein
Cit.17538.1.S1_at	31.07041	116.9336	-3.7635		NA	
Cit.12647.1.S1_at	101.5397	384.8369	-3.79001	AT3G13530.1	1.00E-121	MAPKKK7; ATP binding / binding / kinase/ protein kinase
Cit.12748.1.S1_s_at	20.57195	79.58486	-3.86861	AT4G26530.2	1.00E-172	fructose-bisphosphate aldolase, putative
Cit.19007.1.S1_at	24.27651	95.8157	-3.94685	AT4G23160.1	2.00E-15	protein kinase family protein
Cit.34850.1.S1_s_at	1121.785	4455.771	-3.97204	AT3G47420.1	0	glycerol-3-phosphate transporter, putative / glycerol 3-p
Cit.29547.1.S1_at	30.50396	121.5116	-3.98347	AT4G18380.1	6.00E-46	F-box family protein
Cit.18673.1.S1_at	28.05121	112.5408	-4.01198	AT1G12630.1	2.00E-34	DNA binding / transcription activator/ transcription facto
Cit.5440.1.S1_s_at	227.4013	919.7148	-4.04446	AT2G45130.1	2.00E-76	SPX3 (SPX DOMAIN GENE 3)
Cit.13787.1.S1_s_at	17.42147	70.81545	-4.06484	AT2G03200.1	1.00E-50	aspartyl protease family protein
Cit.4524.1.S1_at	29.46002	121.7877	-4.134	AT5G40530.1	1.00E-112	EXPRESSED IN: 22 plant structures; EXPRESSED DURING:
Cit.5875.1.S1_at	80.5111	342.8669	-4.25863	AT1G08650.1	7.00E-87	PPCK1 (PHOSPHOENOLPYRUVATE CARBOXYLASE KINASE
Cit.19291.1.S1_at	79.86832	342.3085	-4.28591		NA	
Cit.523.1.S1_at	23.10847	99.40835	-4.30181		NA	
Cit.21047.1.S1_s_at	137.3595	591.847	-4.30874	AT5G20790.1	1.00E-25	unknown protein
Cit.24496.1.S1_at	20.19856	88.38753	-4.37593	AT2G37160.2	5.00E-78	transducin family protein / WD-40 repeat family protein
Cit.14365.1.S1_at	85.99367	383.0171	-4.45402		NA	
Cit.27362.1.S1_s_at	144.6833	661.3499	-4.57102	AT5G53050.1	1.00E-15	hydrolase, alpha/beta fold family protein
Cit.15073.1.S1_at	33.60175	156.5089	-4.65776	AT2G03200.1	6.00E-48	aspartyl protease family protein
Cit.11606.1.S1_s_at	458.1826	2135.069	-4.65986	AT3G47420.1	0	glycerol-3-phosphate transporter, putative / glycerol 3-p

Cit.12433.1.S1_at	136.8747	656.7159	-4.79793		NA	
Cit.1868.1.S1_s_at	1875.193	9060.661	-4.83186		NA	
Cit.9817.1.S1_at	360.7487	1793.013	-4.97025		NA	
Cit.9059.1.S1_at	57.33984	288.0199	-5.02303	AT3G14310.1	0	ATPME3; pectinesterase
Cit.6647.1.S1_s_at	148.7036	756.1788	-5.08514	AT5G41080.2	1.00E-106	glycerophosphoryl diester phosphodiesterase family pro
Cit.12037.1.S1_at	318.5461	1625.783	-5.10376		NA	
Cit.28940.1.S1_x_at	20.57848	106.0307	-5.1525	AT1G01300.1	3.00E-57	aspartyl protease family protein
Cit.2555.1.S1_at	212.5603	1210.859	-5.69654	AT1G48300.1	2.00E-45	unknown protein
Cit.13512.1.S1_at	67.71579	458.9785	-6.77801	AT5G20790.1	1.00E-25	unknown protein
Cit.30672.1.S1_at	783.3015	5630.13	-7.18769		NA	
Cit.30672.1.S1_x_at	652.4564	5117.21	-7.84299		NA	
Cit.8587.1.S1_s_at	270.8061	2395.218	-8.84477		NA	
Cit.10471.1.S1_s_at	219.2524	2046.381	-9.33345	AT2G26660.1	1.00E-108	SPX2 (SPX DOMAIN GENE 2)
Cit.24426.1.S1_s_at	230.6912	2424.755	-10.5108	AT1G73010.1	1.00E-110	phosphatase
Cit.2900.1.S1_at	146.3833	1928.851	-13.1767	AT1G73010.1	1.00E-110	phosphatase
Cit.19454.1.S1_x_at	23.85767	320.4539	-13.4319	AT4G08685.1	4.00E-55	SAH7
Cit.11083.1.S1_s_at	230.1616	3347.72	-14.5451	AT3G02040.1	1.00E-141	SRG3 (senescence-related gene 3); glycerophosphodiest
Cit.2899.1.S1_s_at	151.1624	2286.585	-15.1267	AT1G73010.1	1.00E-110	phosphatase

Supplementary Table S6. PDTA in seedless vs. seedy grapefruits at time point 3.

ProbeSet ID	Grapefruit_ Seedless	Grapefruit_ Seedy	Ratio	AtGID	E-Score	Arabidopsis.annotation
Cit.26140.1.S1_at	496.7246	26.07872	19.04712		NA	
Cit.25990.1.S1_x_at	3247.442	232.6083	13.96099	AT1G49640.1	5.00E-06	hydrolase
Cit.30846.1.S1_at	569.4154	53.21327	10.70063	AT1G50320.1	4.00E-48	ATHX; enzyme activator
Cit.11676.1.S1_at	729.5507	75.49277	9.663849		NA	
Cit.12433.1.S1_at	594.2383	64.52944	9.208794		NA	
Cit.17596.1.S1_s_at	2000.43	229.8582	8.702887		NA	
Cit.7568.1.S1_at	639.6319	83.24293	7.683919	AT5G04010.1	3.00E-07	unknown protein
Cit.21773.1.S1_at	150.2885	21.07694	7.130471		NA	
Cit.29242.1.S1_at	131.7624	22.43574	5.87288	AT1G67440.1	9.00E-45	emb1688 (embryo defective 1688); GTP binding / GTPas
Cit.39178.1.S1_s_at	835.5909	151.2625	5.524111	AT5G06760.1	1.00E-48	late embryogenesis abundant group 1 domain-containin
Cit.17790.1.S1_at	190.5788	36.75074	5.185713		NA	
Cit.5776.1.S1_s_at	216.4873	42.68505	5.071736	AT3G10810.1	5.00E-06	zinc finger (C3HC4-type RING finger) family protein
Cit.5776.1.S1_at	99.43875	20.09361	4.948775	AT3G10810.1	5.00E-06	zinc finger (C3HC4-type RING finger) family protein
Cit.17595.1.S1_x_at	173.821	35.7594	4.860848		NA	
Cit.48.1.S1_x_at	125.8388	27.40288	4.592174		NA	
Cit.16835.1.S1_at	95.97462	21.48444	4.467169		NA	
Cit.39331.1.S1_at	227.2116	51.34899	4.42485		NA	
Cit.25692.1.S1_at	234.1252	53.32511	4.390524		NA	
Cit.28730.1.S1_at	107.8066	24.66697	4.370484	AT4G16190.1	3.00E-31	cysteine proteinase, putative
Cit.10894.1.S1_x_at	2737.846	630.2819	4.343844		NA	
Cit.17255.1.S1_at	228.2173	52.63254	4.33605	AT4G21110.1	1.00E-77	G10 family protein
Cit.15055.1.S1_at	287.6444	66.34706	4.335451	AT4G13690.1	1.00E-31	unknown protein
Cit.26531.1.S1_at	120.103	28.09592	4.274749		NA	
Cit.24063.1.S1_at	103.6916	25.14108	4.124389	AT3G22440.1	3.00E-18	hydroxyproline-rich glycoprotein family protein
Cit.22516.1.S1_at	100.6132	24.56067	4.096517		NA	
Cit.6971.1.S1_at	134.522	34.2509	3.927546	AT1G77590.1	5.00E-90	LACS9 (LONG CHAIN ACYL-COA SYNTHETASE 9); long-cha
Cit.6620.1.S1_at	92.48819	23.58162	3.922046	AT3G13810.1	1.00E-103	AtIDD11 (Arabidopsis thaliana Indeterminate(ID)-Domai
Cit.5456.1.S1_at	1238.267	320.0245	3.869288		NA	
Cit.26379.1.S1_x_at	84.26245	22.04046	3.82308	AT4G16520.2	3.00E-20	ATG8F (autophagy 8f); microtubule binding
Cit.10894.1.S1_s_at	4055.558	1061.056	3.82219	AT5G06760.1	1.00E-48	late embryogenesis abundant group 1 domain-containin

Cit.17750.1.S1_x_at	568.8589	154.804	3.674704		NA	
Cit.31137.1.S1_at	113.395	31.08919	3.647409	AT3G51280.1	1.00E-61	male sterility MS5, putative
Cit.27900.1.S1_s_at	184.2274	50.51225	3.647183	AT4G24480.1	2.00E-59	serine/threonine protein kinase, putative
Cit.11563.1.S1_at	763.575	209.7096	3.641107	AT5G37430.1	3.00E-05	unknown protein
Cit.15081.1.S1_at	105.6449	29.14259	3.625103	AT1G18910.1	5.00E-53	protein binding / zinc ion binding
Cit.28730.1.S1_x_at	86.592	24.2011	3.578019	AT4G16190.1	3.00E-31	cysteine proteinase, putative
Cit.23704.1.S1_at	100.3338	28.15537	3.563576	AT5G33340.1	1.00E-34	CDR1 (CONSTITUTIVE DISEASE RESISTANCE 1); aspartic-t
Cit.7887.1.S1_at	74.86784	21.04083	3.558217	AT5G65430.2	2.00E-14	GRF8 (GENERAL REGULATORY FACTOR 8); protein bindin
Cit.281.1.S1_x_at	182.6017	51.78916	3.525867	AT5G11330.1	8.00E-84	monooxygenase family protein
Cit.26543.1.S1_at	98.27969	29.1687	3.369354	AT3G42170.1	1.00E-05	DNA binding
Cit.24294.1.S1_at	131.0962	39.12984	3.350287		NA	
Cit.24173.1.S1_at	80.66205	24.1051	3.346265	AT1G07220.1	4.00E-64	FUNCTIONS IN: molecular_function unknown; INVOLVED
Cit.17780.1.S1_at	121.547	36.6978	3.312106	AT3G12203.1	5.00E-28	scpl17 (serine carboxypeptidase-like 17); serine-type car
Cit.24809.1.S1_x_at	170.9338	51.83224	3.297828		NA	
Cit.28352.1.S1_at	71.68391	21.78063	3.291177	AT2G19540.1	3.00E-23	transducin family protein / WD-40 repeat family protein
Cit.25781.1.S1_at	104.1088	31.70518	3.283653	AT5G17230.3	3.00E-53	phytoene synthase (PSY) / geranylgeranyl-diphosphate g
Cit.24496.1.S1_at	108.3079	33.27348	3.255082	AT2G37160.2	5.00E-78	transducin family protein / WD-40 repeat family protein
Cit.20369.1.S1_at	76.75507	23.74812	3.232048	AT2G47370.1	1.00E-22	FUNCTIONS IN: molecular_function unknown; INVOLVED
Cit.3724.1.S1_at	66.53442	20.83291	3.193717	AT1G47720.1	6.00E-18	OSB1 (Organellar Single-stranded); single-stranded DNA
Cit.25452.1.S1_x_at	101.213	31.72038	3.190788	AT5G55950.1	4.00E-11	transporter-related
Cit.25960.1.S1_at	67.54576	21.23212	3.181301		NA	
Cit.2868.1.S1_at	484.2224	152.7717	3.169582	AT1G29950.2	3.00E-27	transcription factor/ transcription regulator
Cit.27351.1.S1_at	97.70344	30.85023	3.167025	AT5G55000.2	2.00E-09	FIP2; protein binding / voltage-gated potassium channel
Cit.19552.1.S1_at	125.958	39.84056	3.161552	AT1G75290.1	4.00E-35	oxidoreductase, acting on NADH or NADPH
Cit.23718.1.S1_x_at	117.0632	37.02769	3.161504	AT1G53310.3	6.00E-24	ATPPC1 (PHOSPHOENOLPYRUVATE CARBOXYLASE 1); ca
Cit.6374.1.S1_at	137.8344	43.99574	3.132903	AT5G20080.1	1.00E-142	NADH-cytochrome b5 reductase, putative
Cit.21990.1.S1_at	117.9918	37.76002	3.124781		NA	
Cit.21239.1.S1_at	395.6034	126.7811	3.120366		NA	
Cit.11562.1.S1_at	74.26145	23.8218	3.117374	AT5G57580.1	1.00E-162	calmodulin-binding protein
Cit.321.1.S1_at	66.69707	21.49388	3.103073		NA	
Cit.23697.1.S1_at	131.5486	42.4245	3.10077	AT4G16390.1	1.00E-19	LOCATED IN: chloroplast; EXPRESSED IN: 22 plant structu
Cit.25459.1.S1_at	63.8565	20.74421	3.078281	AT3G10950.1	1.00E-18	60S ribosomal protein L37a (RPL37aB)
Cit.35358.1.S1_at	62.1149	20.2118	3.0732	AT3G55920.1	1.00E-36	peptidyl-prolyl cis-trans isomerase, putative / cyclophilin
Cit.22677.1.S1_x_at	89.78257	29.28175	3.066161	AT5G13930.1	2.00E-48	TT4 (TRANSPARENT TESTA 4); naringenin-chalcone synth
Cit.21291.1.S1_at	65.61012	21.47159	3.055671		NA	

Cit.6587.1.S1_at	91.05109	29.85145	3.05014	AT1G75170.2	9.00E-72	SEC14 cytosolic factor family protein / phosphoglyceride
Cit.23742.1.S1_at	82.12247	27.07846	3.03276		NA	
Cit.36715.1.S1_at	87.4026	28.82026	3.032679		NA	
Cit.26185.1.S1_at	165.5439	55.03391	3.008035	AT3G10310.1	4.00E-20	ATP binding / microtubule motor
Cit.29508.1.S1_at	112.1268	37.42455	2.996076	AT4G31580.2	1.00E-74	SRZ-22; protein binding
Cit.26071.1.S1_x_at	75.66357	25.47485	2.970128		NA	
Cit.29072.1.S1_at	792.3351	268.2568	2.953644	AT1G56720.3	4.00E-31	protein kinase family protein
Cit.21363.1.S1_at	77.15858	26.19613	2.945419		NA	
Cit.30830.1.S1_at	78.67509	26.71238	2.945267		NA	
Cit.13144.1.S1_at	60.67716	20.68543	2.933328	AT2G48010.1	1.00E-86	RKF3 (RECEPTOR-LIKE KINASE IN IN FLOWERS 3); kinase/
Cit.24404.1.S1_s_at	73.93848	25.24398	2.928955	AT1G34210.1	0	SERK2 (SOMATIC EMBRYOGENESIS RECEPTOR-LIKE KINA
Cit.22728.1.S1_x_at	79.12705	27.05044	2.925167		NA	
Cit.22723.1.S1_x_at	76.26318	26.11955	2.919774	AT3G46620.1	2.00E-05	zinc finger (C3HC4-type RING finger) family protein
Cit.13941.1.S1_at	67.30982	23.12389	2.910835		NA	
Cit.25838.1.S1_at	82.01509	28.19369	2.908987	AT4G38740.1	2.00E-47	ROC1 (ROTAMASE CYP 1); peptidyl-prolyl cis-trans isome
Cit.25808.1.S1_x_at	121.9718	41.9359	2.908529		NA	
Cit.37925.1.S1_at	75.97116	26.16214	2.903859	AT3G20640.1	3.00E-08	ethylene-responsive protein -related
Cit.14181.1.S1_at	115.9353	39.94382	2.902459	AT4G11080.1	1.00E-128	high mobility group (HMG1/2) family protein
Cit.20355.1.S1_at	58.19223	20.11862	2.892456	AT4G21860.1	7.00E-62	MSRB2 (methionine sulfoxide reductase B 2); peptide-m
Cit.20592.1.S1_x_at	202.5088	70.10957	2.888462		NA	
Cit.17921.1.S1_at	67.68114	23.45039	2.886141	AT1G63980.2	1.00E-06	D111/G-patch domain-containing protein
Cit.16640.1.S1_at	333.3499	115.8918	2.876389	AT1G55830.1	8.00E-49	unknown protein
Cit.4566.1.S1_at	292.2056	101.6401	2.874905	AT4G37580.1	6.00E-70	HLS1 (HOOKLESS 1); N-acetyltransferase
Cit.29910.1.S1_at	61.40067	21.39194	2.870271		NA	
Cit.32558.1.S1_at	101.9295	35.72161	2.853441	AT2G13440.1	2.00E-78	glucose-inhibited division family A protein
Cit.29243.1.S1_at	86.43335	30.40123	2.843087	AT1G30400.2	4.00E-41	MRP1 (ARABIDOPSIS THALIANA MULTIDRUG RESISTANC
Cit.19070.1.S1_at	57.91087	20.38799	2.84044	AT5G20400.1	3.00E-19	oxidoreductase, 2OG-Fe(II) oxygenase family protein
Cit.7588.1.S1_at	152.3686	53.68578	2.838156	AT5G06150.1	5.00E-42	CYC1BAT; cyclin-dependent protein kinase regulator
Cit.8815.1.S1_x_at	182.0664	64.20819	2.835564		NA	
Cit.23141.1.S1_at	63.97947	22.58228	2.833171	AT2G26900.1	3.00E-76	bile acid:sodium symporter family protein
Cit.21865.1.S1_at	125.9646	44.49922	2.830715	AT4G23620.1	2.00E-52	50S ribosomal protein-related
Cit.17425.1.S1_s_at	80.26218	28.38809	2.827319	AT1G28380.1	4.00E-86	NSL1 (necrotic spotted lesions 1)
Cit.35198.1.S1_at	91.73758	32.48015	2.82442	AT4G02195.1	2.00E-50	SYP42 (SYNTAXIN OF PLANTS 42); SNAP receptor
Cit.27780.1.S1_at	58.11519	20.63315	2.816593	AT5G09650.1	8.00E-11	AtPPa6 (Arabidopsis thaliana pyrophosphorylase 6); inor
Cit.14975.1.S1_at	124.27	44.33266	2.803125	AT3G17030.1	1.00E-141	INVOLVED IN: biological_process unknown; LOCATED IN

Cit.4819.1.S1_at	62.62226	22.34589	2.802406	AT3G57680.1	0	peptidase S41 family protein
Cit.19161.1.S1_at	72.89477	26.11281	2.791533	AT4G19040.2	8.00E-59	EDR2; lipid binding
Cit.28588.1.S1_at	257.1151	92.42084	2.782003	AT3G23810.1	3.00E-12	SAHH2 (S-ADENOSYL-L-HOMOCYSTEINE (SAH) HYDROLA
Cit.22710.1.S1_at	2884.288	1038.972	2.776098		NA	
Cit.6594.1.S1_at	71.13535	25.86017	2.750769	AT4G34540.1	2.00E-96	isoflavone reductase family protein
Cit.26923.1.S1_at	55.8727	20.35567	2.744822	AT1G12800.1	3.00E-05	S1 RNA-binding domain-containing protein
Cit.16237.1.S1_at	99.38644	36.28302	2.7392	ATMG00300.1	2.00E-10	hypothetical protein
Cit.15198.1.S1_at	104.5635	38.31952	2.728727	AT5G01510.1	3.00E-22	FUNCTIONS IN: molecular_function unknown; INVOLVED
Cit.12037.1.S1_at	472.151	174.2266	2.709982		NA	
Cit.4532.1.S1_at	192.9976	71.29648	2.706972	AT4G16800.1	2.00E-90	enoyl-CoA hydratase, putative
Cit.4578.1.S1_at	78.79058	29.153	2.702658		NA	
Cit.27781.1.S1_at	125.2367	46.38567	2.699901	AT5G40850.1	7.00E-11	UPM1 (UROPHORPHYRIN METHYLASE 1); uroporphyrin-I
Cit.33104.1.S1_at	70.76247	26.23022	2.697746	AT5G62480.2	4.00E-12	ATGSTU9 (ARABIDOPSIS THALIANA GLUTATHIONE S-TRA
Cit.22742.1.S1_at	124.3673	46.13288	2.695849	AT5G63660.1	6.00E-19	PDF2.5
Cit.29477.1.S1_at	54.5051	20.25535	2.690899	AT1G48170.1	3.00E-10	unknown protein
Cit.27379.1.S1_at	57.80896	21.50558	2.688091	AT1G75430.1	3.00E-45	BLH11 (BEL1-LIKE HOMEODOMAIN 11); transcription fac
Cit.5988.1.S1_at	105.8583	39.47332	2.681768	AT1G49510.1	8.00E-12	emb1273 (embryo defective 1273)
Cit.23861.1.S1_at	110.8387	41.33648	2.681377		NA	
Cit.29098.1.S1_at	62.12412	23.22505	2.674876	AT4G25310.1	4.00E-19	oxidoreductase, 2OG-Fe(II) oxygenase family protein
Cit.16705.1.S1_at	63.38818	23.70767	2.673741	AT1G47710.1	2.00E-64	serpin, putative / serine protease inhibitor, putative
Cit.27820.1.S1_s_at	78.96614	29.53706	2.67346	AT4G25140.1	5.00E-45	OLEO1 (OLEOSIN 1)
Cit.22564.1.S1_x_at	68.882	25.81198	2.668606		NA	
Cit.12863.1.S1_at	61.7028	23.20979	2.658482	AT1G25380.1	1.00E-129	mitochondrial substrate carrier family protein
Cit.22515.1.S1_x_at	875.0891	331.7206	2.638031		NA	
Cit.34953.1.S1_at	54.99615	20.8635	2.635998	AT1G79830.2	1.00E-09	GC5 (golgin candidate 5); protein binding
Cit.17957.1.S1_at	62.78672	23.82939	2.634844	AT5G07330.1	4.00E-35	unknown protein
Cit.24324.1.S1_x_at	642.4562	244.3299	2.629462	AT5G13930.1	3.00E-09	TT4 (TRANSPARENT TESTA 4); naringenin-chalcone synth
Cit.30234.1.S1_at	79.87562	30.39438	2.627973	AT3G20290.2	5.00E-51	ATEHD1 (EPS15 HOMOLOGY DOMAIN 1); GTP binding / (
Cit.34684.1.S1_at	62.42403	23.85066	2.617287	AT1G16670.1	2.00E-17	protein kinase family protein
Cit.28609.1.S1_at	257.3809	98.44913	2.614354		NA	
Cit.40086.1.S1_at	60.36148	23.21537	2.600065	AT5G04900.1	2.00E-58	short-chain dehydrogenase/reductase (SDR) family prote
Cit.17196.1.S1_at	59.65589	22.94958	2.599433	AT1G54570.1	9.00E-83	esterase/lipase/thioesterase family protein
Cit.749.1.S1_x_at	253.4108	97.48921	2.599373	AT1G73230.1	1.00E-69	nascent polypeptide-associated complex (NAC) domain-c
Cit.12150.1.S1_at	73.33955	28.2258	2.598316	AT1G22170.1	6.00E-16	phosphoglycerate/bisphosphoglycerate mutase family p
Cit.34430.1.S1_at	57.42005	22.10443	2.597672	AT3G06550.3	9.00E-18	FUNCTIONS IN: molecular_function unknown; INVOLVED

Cit.23733.1.S1_x_at	54.42918	21.04469	2.586362	AT1G67090.1	2.00E-32	RBCS1A (RIBULOSE BISPHOSPHATE CARBOXYLASE SMALL
Cit.25989.1.S1_at	152.7443	59.06083	2.58622	AT2G34190.1	2.00E-50	xanthine/uracil permease family protein
Cit.22550.1.S1_s_at	103.5364	40.07568	2.583522	AT1G03220.1	1.00E-121	extracellular dermal glycoprotein, putative / EDGP, puta
Cit.2635.1.S1_at	181.9389	70.56532	2.578305	AT3G03520.1	1.00E-78	phosphoesterase family protein
Cit.22423.1.S1_x_at	867.5468	336.6153	2.577265	AT1G54410.1	3.00E-15	dehydrin family protein
Cit.15924.1.S1_at	58.25977	22.64974	2.572205	AT3G01680.1	7.00E-09	unknown protein
Cit.14210.1.S1_at	166.4434	64.74122	2.570903	AT1G03220.1	1.00E-121	extracellular dermal glycoprotein, putative / EDGP, puta
Cit.23564.1.S1_at	116.522	45.32807	2.570637	AT5G20250.3	2.00E-53	DIN10 (DARK INDUCIBLE 10); hydrolase, hydrolyzing O-g
Cit.28519.1.S1_at	76.8111	29.93723	2.565738		NA	
Cit.939.1.S1_s_at	154.2209	60.11676	2.565356	AT3G13610.1	1.00E-134	oxidoreductase, 2OG-Fe(II) oxygenase family protein
Cit.36665.1.S1_at	109.6903	42.89907	2.556939	AT3G50790.1	5.00E-74	late embryogenesis abundant protein, putative / LEA pro
Cit.23242.1.S1_at	74.08054	29.08805	2.546769	AT3G08820.1	2.00E-58	pentatricopeptide (PPR) repeat-containing protein
Cit.36690.1.S1_at	69.69792	27.37089	2.546425		NA	
Cit.23371.1.S1_x_at	77.62112	30.5128	2.543887	AT2G30860.1	9.00E-30	ATGSTF9 (GLUTATHIONE S-TRANSFERASE PHI 9); copper
Cit.24920.1.S1_at	56.25812	22.12044	2.543264		NA	
Cit.23686.1.S1_at	60.21679	23.75597	2.534807		NA	
Cit.30804.1.S1_s_at	70.15438	27.71202	2.531551		NA	
Cit.14030.1.S1_at	269.9881	106.8373	2.527096	AT5G45290.1	2.00E-49	zinc finger (C3HC4-type RING finger) family protein
Cit.29785.1.S1_at	117.0607	46.40895	2.522373	AT1G19600.1	7.00E-70	pfkB-type carbohydrate kinase family protein
Cit.22983.1.S1_x_at	62.15174	24.74117	2.512078		NA	
Cit.32435.1.S1_at	94.3562	37.59856	2.50957	AT3G20550.1	8.00E-15	DDL (DAWDLE)
Cit.21857.1.S1_at	314.7839	125.4808	2.508622	AT2G45130.1	4.00E-29	SPX3 (SPX DOMAIN GENE 3)
Cit.24660.1.S1_at	68.5015	27.50012	2.490953	AT4G27090.1	4.00E-11	60S ribosomal protein L14 (RPL14B)
Cit.26559.1.S1_at	54.00099	21.68494	2.490253		NA	
Cit.37645.1.S1_at	78.43057	31.53219	2.487318	AT3G06030.1	2.00E-44	ANP3 (ARABIDOPSIS NPK1-RELATED PROTEIN KINASE 3);
Cit.14788.1.S1_at	97.23314	39.09504	2.487097	AT5G63260.1	6.00E-26	zinc finger (CCCH-type) family protein
Cit.22458.1.S1_at	60.98921	24.57297	2.481963		NA	
Cit.11817.1.S1_at	466.989	188.2944	2.4801	AT3G04830.1	1.00E-133	binding
Cit.28583.1.S1_x_at	49.57705	20.01029	2.477578		NA	
Cit.14712.1.S1_at	456.863	184.4914	2.476338	AT2G17250.1	7.00E-85	EMB2762 (EMBRYO DEFECTIVE 2762)
Cit.30690.1.S1_at	77.30466	31.24822	2.47389	AT1G54260.1	7.00E-06	histone H1/H5 family protein
Cit.7688.1.S1_at	68.35004	27.69301	2.468133	AT5G40030.1	5.00E-77	protein kinase, putative
Cit.28017.1.S1_at	73.15058	29.6591	2.466379	AT2G41350.2	2.00E-33	unknown protein
Cit.25344.1.S1_at	63.38566	25.70685	2.465711	AT3G42170.1	2.00E-15	DNA binding
Cit.28447.1.S1_x_at	60.29271	24.48971	2.461961	AT5G14200.1	3.00E-78	3-isopropylmalate dehydrogenase, chloroplast, putative

Cit.8522.1.S1_at	75.91004	30.90594	2.456163	AT5G08350.1	2.00E-45	GRAM domain-containing protein / ABA-responsive prot
Cit.29641.1.S1_s_at	167.9324	68.46148	2.452947	AT5G14790.1	1.00E-110	binding
Cit.38821.1.S1_at	62.52779	25.49629	2.452427	AT2G28660.1	4.00E-16	copper-binding family protein
Cit.28929.1.S1_at	70.00783	28.56069	2.451195		NA	
Cit.6522.1.S1_at	111.9088	45.65994	2.450919	AT5G63090.4	2.00E-60	LOB (LATERAL ORGAN BOUNDARIES)
Cit.37794.1.S1_s_at	243.2338	99.39076	2.447248	AT1G80860.1	3.00E-76	PLMT (PHOSPHOLIPID N-METHYLTRANSFERASE); phosph
Cit.11673.1.S1_at	93.98776	38.42142	2.446233	AT3G05190.1	7.00E-12	aminotransferase class IV family protein
Cit.19530.1.S1_at	186.1351	76.10052	2.445911		NA	
Cit.15971.1.S1_at	189.6615	77.59043	2.444393		NA	
Cit.22431.1.S1_at	263.5486	107.8258	2.444207	AT5G52780.1	3.00E-26	unknown protein
Cit.29740.1.S1_at	105.2512	43.16296	2.438461		NA	
Cit.23129.1.S1_at	210.2083	86.20602	2.438441	AT5G66590.1	1.00E-52	allergen V5/Tpx-1-related family protein
Cit.5351.1.S1_x_at	148.9276	61.0942	2.437672		NA	
Cit.23947.1.S1_at	55.80172	22.90151	2.436596	AT1G29200.3	3.00E-64	unknown protein
Cit.19200.1.S1_at	84.61603	34.73291	2.436192		NA	
Cit.37059.1.S1_x_at	65.64897	26.98999	2.432345	AT2G37210.1	4.00E-28	Encodes a protein of unknown function. It has been crys
Cit.21562.1.S1_x_at	50.3666	20.72737	2.429956	ATCG00070.1	5.00E-18	PSII K protein
Cit.38830.1.S1_at	69.01544	28.41166	2.429124	AT1G72300.1	1.00E-82	leucine-rich repeat transmembrane protein kinase, puta
Cit.15391.1.S1_at	119.4331	49.20251	2.427378		NA	
Cit.10603.1.S1_at	51.11412	21.09659	2.422862		NA	
Cit.22003.1.S1_at	413.1873	170.6897	2.420693	AT1G64230.2	1.00E-63	ubiquitin-conjugating enzyme, putative
Cit.24444.1.S1_x_at	68.80875	28.48506	2.415608		NA	
Cit.10194.1.S1_x_at	187.1799	77.61225	2.411731	AT1G30910.1	1.00E-123	molybdenum cofactor sulfurase family protein
Cit.28775.1.S1_at	63.49136	26.32644	2.411696		NA	
Cit.7951.1.S1_s_at	48.40436	20.08715	2.409718	AT2G34260.1	1.00E-127	transducin family protein / WD-40 repeat family protein
Cit.30365.1.S1_at	70.74021	29.45803	2.40139	AT5G49650.1	1.00E-05	xylulose kinase, putative
Cit.32934.1.S1_at	50.26106	20.96397	2.397497		NA	
Cit.30249.1.S1_at	66.8196	27.88906	2.395907		NA	
Cit.21073.1.S1_at	70.23749	29.31722	2.395776		NA	
Cit.37795.1.S1_at	158.1236	66.0667	2.393393		NA	
Cit.40152.1.S1_at	96.65584	40.39434	2.392807	AT3G51870.1	4.00E-37	binding / transporter
Cit.18713.1.S1_at	115.0094	48.17437	2.387357		NA	
Cit.18162.1.S1_s_at	51.68456	21.68118	2.383844	AT3G09100.2	8.00E-29	mRNA capping enzyme family protein
Cit.37452.1.S1_at	87.83403	36.88566	2.381251	AT4G03100.1	5.00E-58	rac GTPase activating protein, putative
Cit.16802.1.S1_at	246.8189	103.7974	2.377891	AT4G21820.1	7.00E-24	binding / calmodulin binding

Cit.11903.1.S1_x_at	81.02908	34.07771	2.377774	AT5G19940.1	2.00E-55	plastid-lipid associated protein PAP-related / fibrillin-rela
Cit.35173.1.S1_at	67.6535	28.45332	2.377701	AT2G37000.1	3.00E-38	TCP family transcription factor, putative
Cit.46.1.S1_at	83.43156	35.09075	2.377594	AT2G24570.1	1.00E-123	WRKY17; calmodulin binding / transcription factor
Cit.19559.1.S1_at	95.19637	40.06681	2.375941		NA	
Cit.13558.1.S1_s_at	115.5356	48.77802	2.3686	AT1G16030.1	1.00E-111	Hsp70b (heat shock protein 70B); ATP binding
Cit.26584.1.S1_at	71.57753	30.22557	2.368112	AT5G16310.1	2.00E-14	UCH1; ubiquitin thiolesterase
Cit.36004.1.S1_at	51.45973	21.74166	2.366872	AT4G35450.1	1.00E-27	AKR2 (ANKYRIN REPEAT-CONTAINING PROTEIN 2); prote
Cit.16661.1.S1_at	223.2453	94.61596	2.359489	AT5G12930.1	3.00E-49	unknown protein
Cit.16635.1.S1_at	87.99685	37.30699	2.358723	AT3G47180.1	5.00E-42	zinc finger (C3HC4-type RING finger) family protein
Cit.19025.1.S1_at	53.05276	22.51372	2.356464	AT5G54160.1	4.00E-22	ATOMT1 (O-METHYLTRANSFERASE 1); caffeate O-methy
Cit.25713.1.S1_at	392.4503	166.6071	2.355544	AT4G10180.1	4.00E-74	DET1 (DE-ETIOLATED 1); catalytic
Cit.35019.1.S1_at	50.41784	21.41191	2.354663		NA	
Cit.13692.1.S1_at	95.44302	40.54046	2.354266	AT3G26430.1	1.00E-122	GDSL-motif lipase/hydrolase family protein
Cit.21445.1.S1_x_at	359.6553	152.9127	2.35203		NA	
Cit.25758.1.S1_x_at	118.2424	50.28794	2.351307	AT5G51080.3	2.00E-24	RNase H domain-containing protein
Cit.23460.1.S1_at	438.7403	186.6901	2.350099	AT4G33260.1	1.00E-109	CDC20.2; signal transducer
Cit.2990.1.S1_x_at	930.9658	396.7799	2.346303	AT3G49570.1	7.00E-16	LSU3 (RESPONSE TO LOW SULFUR 3)
Cit.682.1.S1_at	82.01368	34.99264	2.343741	AT3G62290.1	1.00E-100	ATARFA1E (ADP-ribosylation factor A1E); GTP binding / p
Cit.28337.1.S1_at	86.35066	36.93464	2.337932		NA	
Cit.32267.1.S1_at	69.00945	29.52576	2.337262	AT1G58210.1	1.00E-25	EMB1674 (EMBRYO DEFECTIVE 1674)
Cit.6895.1.S1_at	59.51492	25.46462	2.337161	AT4G39880.1	7.00E-08	ribosomal protein L23 family protein
Cit.31938.1.S1_s_at	51.64862	22.17509	2.329128	AT5G52030.2	1.00E-107	TraB protein-related
Cit.37972.1.S1_at	184.4797	79.28121	2.326903		NA	
Cit.35262.1.S1_s_at	111.2408	47.8569	2.324446	AT3G03750.1	4.00E-96	SET domain-containing protein
Cit.24402.1.S1_at	127.5261	54.88473	2.323526		NA	
Cit.6513.1.S1_at	336.1283	144.9122	2.319531	AT1G20610.1	4.00E-94	CYCB2;3 (Cyclin B2;3); cyclin-dependent protein kinase r
Cit.27069.1.S1_at	47.26387	20.39884	2.316988	AT5G17440.1	6.00E-30	LUC7 N_terminus domain-containing protein
Cit.29497.1.S1_at	103.2544	44.56575	2.3169	AT2G38610.2	7.00E-67	KH domain-containing protein
Cit.16441.1.S1_at	77.39724	33.43732	2.314696	AT4G36710.1	5.00E-70	transcription factor
Cit.20427.1.S1_at	117.9316	50.97422	2.313554		NA	
Cit.25712.1.S1_at	253.0388	109.8814	2.302836		NA	
Cit.6169.1.S1_at	54.07651	23.48651	2.30245	AT5G43710.1	5.00E-41	glycoside hydrolase family 47 protein
Cit.29082.1.S1_s_at	109.338	47.49506	2.302092		NA	
Cit.16339.1.S1_at	78.01925	33.94141	2.298645		NA	
Cit.26502.1.S1_at	62.11383	27.05621	2.295733	AT2G35360.1	2.00E-09	ubiquitin family protein

Cit.28737.1.S1_at	109.6836	47.85363	2.292064	AT5G13640.1	4.00E-44	ATPDAT; phosphatidylcholine-sterol O-acyltransferase
Cit.2265.1.S1_at	81.06745	35.43317	2.287897		NA	
Cit.38868.1.S1_at	125.5012	54.85611	2.287825	AT1G50240.2	7.00E-53	FU (FUSED); protein serine/threonine kinase
Cit.36618.1.S1_at	94.3046	41.24566	2.286413	AT3G48040.1	9.00E-11	ROP10 (RHO-RELATED PROTEIN FROM PLANTS 10); GTP
Cit.38616.1.S1_at	134.7445	58.9988	2.283852	AT3G22880.1	2.00E-67	DMC1 (DISRUPTION OF MEIOTIC CONTROL 1); ATP bindi
Cit.15539.1.S1_at	59.75325	26.16437	2.283764	AT1G77720.1	1.00E-54	protein kinase family protein
Cit.25431.1.S1_s_at	3196.339	1400.206	2.282763	AT1G06470.2	3.00E-19	phosphate translocator-related
Cit.15517.1.S1_at	75.59115	33.17434	2.278603	AT1G48970.1	8.00E-61	GTP binding / translation initiation factor
Cit.16390.1.S1_at	93.86111	41.19663	2.278369	AT3G56230.1	2.00E-59	speckle-type POZ protein-related
Cit.31773.1.S1_at	68.19775	29.93384	2.278283	AT5G17690.1	5.00E-15	TFL2 (TERMINAL FLOWER 2); DNA binding / methylated
Cit.35847.1.S1_at	62.65108	27.50574	2.277746	AT5G40740.1	3.00E-90	unknown protein
Cit.7155.1.S1_at	248.3423	109.0779	2.276743	AT2G39930.1	9.00E-05	ISA1 (ISOAMYLASE 1); alpha-amylase/ isoamylase
Cit.9571.1.S1_at	55.31937	24.31551	2.275065	AT2G29360.1	1.00E-85	tropinone reductase, putative / tropine dehydrogenase,
Cit.16627.1.S1_at	91.9428	40.4391	2.273611		NA	
Cit.38620.1.S1_at	102.883	45.26954	2.272676		NA	
Cit.12436.1.S1_at	99.40871	43.77655	2.270821	AT4G35730.1	2.00E-16	unknown protein
Cit.26187.1.S1_at	96.66747	42.58796	2.269831		NA	
Cit.23651.1.S1_at	60.12201	26.49023	2.269592		NA	
Cit.35111.1.S1_at	90.57207	39.91224	2.269281	AT3G25660.1	1.00E-101	glutamyl-tRNA(Gln) amidotransferase, putative
Cit.24942.1.S1_at	90.54897	39.90546	2.269087		NA	
Cit.7655.1.S1_at	126.4901	55.77496	2.267865	AT2G31240.1	1.00E-165	tetratricopeptide repeat (TPR)-containing protein
Cit.32804.1.S1_at	58.30098	25.71025	2.267616	AT4G30210.2	2.00E-10	ATR2 (ARABIDOPSIS P450 REDUCTASE 2); NADPH-hemo
Cit.16236.1.S1_at	65.68882	28.97146	2.267363	AT4G22990.1	1.00E-156	SPX (SYG1/Pho81/XPR1) domain-containing protein
Cit.23061.1.S1_at	77.95139	34.40041	2.266002		NA	
Cit.33271.1.S1_at	161.7199	71.37949	2.265635		NA	
Cit.21219.1.S1_at	423.6696	187.0306	2.265242		NA	
Cit.7830.1.S1_at	318.9064	140.9022	2.263317	AT3G25980.1	1.00E-104	mitotic spindle checkpoint protein, putative (MAD2)
Cit.28030.1.S1_at	75.71915	33.46106	2.262904	AT5G49930.1	3.00E-22	emb1441 (embryo defective 1441); nucleic acid binding
Cit.16817.1.S1_at	133.9185	59.20221	2.262052	AT5G43490.1	6.00E-07	unknown protein
Cit.24706.1.S1_x_at	59.57906	26.39945	2.25683		NA	
Cit.23051.1.S1_at	59.62197	26.43111	2.25575		NA	
Cit.15136.1.S1_s_at	315.2056	140.0475	2.250705	AT4G15140.1	3.00E-32	unknown protein
Cit.38240.1.S1_s_at	59.86419	26.60696	2.249945	AT1G69220.1	0	SIK1; ATP binding / kinase/ protein kinase/ protein serin
Cit.37962.1.S1_at	64.77198	28.80451	2.248675	AT1G31910.1	9.00E-53	GHMP kinase family protein
Cit.10425.1.S1_s_at	1692.545	752.9045	2.248021	AT5G05250.1	1.00E-42	unknown protein

Cit.10885.1.S1_at	53.54182	23.8378	2.246089	AT3G19760.1	0	eukaryotic translation initiation factor 4A, putative / eIF-
Cit.24444.1.S1_at	75.52542	33.63015	2.245765		NA	
Cit.36102.1.S1_at	67.79578	30.19688	2.245125		NA	
Cit.19483.1.S1_at	105.9222	47.26103	2.241216		NA	
Cit.38834.1.S1_at	88.29369	39.41713	2.239983		NA	
Cit.29003.1.S1_at	60.62728	27.1223	2.23533	AT4G32870.1	9.00E-45	unknown protein
Cit.20503.1.S1_at	86.15082	38.56179	2.234098		NA	
Cit.611.1.S1_x_at	48.97588	21.928	2.233486	AT3G09790.1	1.00E-19	UBQ8; protein binding
Cit.26238.1.S1_x_at	59.39297	26.62062	2.231089		NA	
Cit.38040.1.S1_at	57.75027	25.89678	2.230017	AT2G44660.1	1.00E-23	transferase, transferring glycosyl groups / transferase, tr
Cit.3456.1.S1_at	54.65219	24.51291	2.229527	AT1G02850.2	1.00E-88	BGLU11 (BETA GLUCOSIDASE 11); hydrolase, hydrolyzing
Cit.5267.1.S1_at	60.37978	27.0922	2.228678	AT1G08830.2	2.00E-17	CSD1 (COPPER/ZINC SUPEROXIDE DISMUTASE 1); superc
Cit.29870.1.S1_at	90.26462	40.54716	2.226164	AT2G41900.1	4.00E-67	zinc finger (CCCH-type) family protein
Cit.7307.1.S1_at	58.11075	26.19172	2.218669	AT4G37050.1	2.00E-09	PLP4 (PATATIN-LIKE PROTEIN 4); nutrient reservoir
Cit.26995.1.S1_at	64.37615	29.05253	2.215853		NA	
Cit.28939.1.S1_at	337.1494	152.3763	2.21261		NA	
Cit.27634.1.S1_x_at	67.34502	30.46732	2.210402	AT1G34110.1	3.00E-47	leucine-rich repeat transmembrane protein kinase, puta
Cit.21878.1.S1_at	75.69788	34.31934	2.205692	AT5G09660.2	3.00E-32	PMDH2 (peroxisomal NAD-malate dehydrogenase 2); ma
Cit.31046.1.S1_s_at	67.95023	30.85262	2.202414	AT5G03406.1	7.00E-93	ATP binding / aminoacyl-tRNA ligase/ histidine-tRNA liga
Cit.21580.1.S1_at	65.07041	29.58031	2.199788		NA	
Cit.6722.1.S1_at	63.21747	28.76063	2.198056	AT5G17250.1	1.00E-21	catalytic/ transferase
Cit.36343.1.S1_s_at	329.7645	150.0425	2.197807	AT5G08690.1	4.00E-13	ATP synthase beta chain 2, mitochondrial
Cit.33439.1.S1_at	63.2631	28.78643	2.197671		NA	
Cit.3536.1.S1_at	131.3164	59.86136	2.193676	AT5G26780.1	5.00E-97	SHM2 (SERINE HYDROXYMETHYLTRANSFERASE 2); cataly
Cit.34208.1.S1_at	80.19483	36.55798	2.193634		NA	
Cit.36930.1.S1_at	55.49133	25.3151	2.192025	AT2G36890.1	2.00E-65	RAX2 (REGULATOR OF AXILLARY MERISTEMS 2); DNA bir
Cit.25473.1.S1_at	60.98383	27.82141	2.191975	AT5G02400.1	8.00E-10	PLL2; catalytic/ protein serine/threonine phosphatase
Cit.28374.1.S1_at	52.71664	24.06397	2.190688	AT5G14430.2	4.00E-19	dehydration-responsive protein-related
Cit.35567.1.S1_at	45.01204	20.55074	2.190288		NA	
Cit.38556.1.S1_at	74.97819	34.23457	2.190131	AT1G52160.1	2.00E-58	TRZ3 (TRNASE Z 3); 3'-tRNA processing endoribonuclease
Cit.5726.1.S1_s_at	289.4642	132.2118	2.189398	AT2G32590.1	1.00E-102	INVOLVED IN: mitosis, mitotic cell cycle; LOCATED IN: nu
Cit.38325.1.S1_at	58.39524	26.67225	2.189363		NA	
Cit.22960.1.S1_at	55.35175	25.29102	2.188593	AT2G30490.1	2.00E-09	C4H (CINNAMATE-4-HYDROXYLASE); trans-cinnamate 4-
Cit.31524.1.S1_at	302.7311	138.3653	2.187912		NA	
Cit.18445.1.S1_at	122.6123	56.13226	2.184346	AT4G12680.1	3.00E-36	unknown protein

Cit.23084.1.S1_at	52.76273	24.16833	2.183135		NA	
Cit.16313.1.S1_at	46.72344	21.40307	2.183025	AT1G62360.1	1.00E-118	STM (SHOOT MERISTEMLESS); transcription factor
Cit.38019.1.S1_at	114.0037	52.23347	2.182579	AT1G03310.1	1.00E-42	isoamylase, putative / starch debranching enzyme, putat
Cit.13719.1.S1_at	137.8966	63.20021	2.181901		NA	
Cit.2740.1.S1_at	113.0592	51.84898	2.180548		NA	
Cit.22018.1.S1_at	74.61828	34.30068	2.175417		NA	
Cit.37370.1.S1_at	54.92242	25.25042	2.175109	AT3G13960.1	6.00E-10	AtGRF5 (GROWTH-REGULATING FACTOR 5); transcriptio
Cit.24884.1.S1_at	126.1745	58.01324	2.174926	AT1G48300.1	1.00E-15	unknown protein
Cit.13288.1.S1_at	49.3539	22.697	2.174468	AT1G16350.1	1.00E-159	inosine-5'-monophosphate dehydrogenase, putative
Cit.13867.1.S1_at	483.5682	222.7105	2.171286	AT4G21270.1	1.00E-114	ATK1 (ARABIDOPSIS THALIANA KINESIN 1); microtubule r
Cit.21439.1.S1_s_at	80.16009	36.91979	2.171196	AT1G15130.1	3.00E-41	hydroxyproline-rich glycoprotein family protein
Cit.31299.1.S1_at	118.9151	54.77536	2.17096	AT5G48170.1	4.00E-37	SLY2 (SLEEPY2)
Cit.24942.1.S1_x_at	53.51814	24.6609	2.170162		NA	
Cit.2748.1.S1_at	74.99667	34.56784	2.16955	AT5G19590.1	1.00E-54	unknown protein
Cit.22046.1.S1_at	60.89271	28.07626	2.168833		NA	
Cit.24185.1.S1_s_at	307.0798	141.6046	2.168572	AT3G54660.1	0	GR (GLUTATHIONE REDUCTASE); ATP binding / glutathio
Cit.20082.1.S1_x_at	1161.251	535.7349	2.167585	AT1G75750.1	7.00E-33	GASA1 (GAST1 PROTEIN HOMOLOG 1)
Cit.28693.1.S1_at	136.402	62.95293	2.16673	AT3G09780.1	9.00E-20	CCR1 (ARABIDOPSIS THALIANA CRINKLY4 RELATED 1); AT
Cit.31961.1.S1_at	71.6623	33.07489	2.166668		NA	
Cit.31971.1.S1_at	67.60714	31.20518	2.166536	AT5G06800.1	6.00E-21	myb family transcription factor
Cit.17568.1.S1_x_at	45.65868	21.09083	2.164859	AT2G02240.1	2.00E-09	MEE66 (maternal effect embryo arrest 66)
Cit.22214.1.S1_at	72.33192	33.43103	2.163616	AT1G24030.2	1.00E-105	protein kinase family protein
Cit.29718.1.S1_at	87.24331	40.34961	2.162185	AT4G17430.1	2.00E-62	unknown protein
Cit.26992.1.S1_at	216.2357	100.0134	2.162067	AT1G72880.1	1.00E-05	acid phosphatase survival protein SurE, putative
Cit.16081.1.S1_at	57.94646	26.81977	2.160588	AT4G30610.1	7.00E-66	BRS1 (BRI1 SUPPRESSOR 1); serine-type carboxypeptidas
Cit.37182.1.S1_at	62.36589	28.90386	2.157701	AT5G16610.2	4.00E-35	unknown protein
Cit.38178.1.S1_at	116.3448	53.93005	2.157328	AT1G27460.1	2.00E-44	NPGR1 (NO POLLEN GERMINATION RELATED 1); calmodu
Cit.19496.1.S1_at	74.95762	34.75311	2.156861	AT5G37670.1	2.00E-44	15.7 kDa class I-related small heat shock protein-like (HS
Cit.15458.1.S1_at	52.89821	24.52847	2.156605	AT1G18400.1	1.00E-45	BEE1 (BR Enhanced Expression 1); transcription factor
Cit.19596.1.S1_at	593.1185	275.1768	2.155409	AT3G01130.1	3.00E-16	unknown protein
Cit.22306.1.S1_at	69.2054	32.12833	2.15403	AT1G60800.1	8.00E-89	NIK3 (NSP-INTERACTING KINASE 3); kinase
Cit.13967.1.S1_at	136.47	63.51969	2.148468	AT5G08020.1	1.00E-110	RPA70B (RPA70-KDA SUBUNIT B); DNA binding / nucleic
Cit.30956.1.S1_at	78.92165	36.74351	2.147907	AT1G77660.1	8.00E-08	MORN (Membrane Occupation and Recognition Nexus) r
Cit.2474.1.S1_at	450.7326	210.0132	2.146211	AT4G31940.1	1.00E-26	CYP82C4; electron carrier/ heme binding / iron ion bindi
Cit.23136.1.S1_x_at	94.65191	44.11126	2.145754		NA	

Cit.7373.1.S1_at	48.00273	22.39847	2.143125	AT5G60530.1	2.00E-11	late embryogenesis abundant protein-related / LEA prot
Cit.29589.1.S1_at	51.56974	24.10159	2.139682	AT2G07360.1	4.00E-37	SH3 domain-containing protein
Cit.36931.1.S1_at	45.9443	21.48544	2.138392	AT5G41270.1	3.00E-08	FUNCTIONS IN: molecular_function unknown; INVOLVED
Cit.16018.1.S1_at	101.6195	47.53132	2.137948	AT3G02820.1	8.00E-68	zinc knuckle (CCHC-type) family protein
Cit.24560.1.S1_at	133.1634	62.29901	2.137488		NA	
Cit.14433.1.S1_s_at	430.0782	201.2081	2.13748	AT4G20430.1	1.00E-165	subtilase family protein
Cit.5783.1.S1_at	94.58121	44.25914	2.136987	AT2G23360.1	4.00E-28	transport protein-related
Cit.7037.1.S1_at	66.38617	31.09964	2.134628		NA	
Cit.5745.1.S1_s_at	105.79	49.58595	2.133467		NA	
Cit.9671.1.S1_at	314.9363	147.9359	2.12887	AT5G63660.1	4.00E-23	PDF2.5
Cit.34740.1.S1_at	52.68142	24.74864	2.128659		NA	
Cit.22431.1.S1_x_at	177.9982	83.64424	2.128039	AT5G52780.1	3.00E-26	unknown protein
Cit.1799.1.S1_at	53.45253	25.15886	2.124601		NA	
Cit.23391.1.S1_x_at	56.81491	26.7471	2.124152	AT4G14342.1	4.00E-09	pre-mRNA splicing factor 10 kDa subunit, putative
Cit.28860.1.S1_at	141.8859	66.87286	2.121726	AT3G12580.1	7.00E-13	HSP70 (heat shock protein 70); ATP binding
Cit.38075.1.S1_at	47.78927	22.57637	2.116783	AT5G62090.2	1.00E-08	FUNCTIONS IN: molecular_function unknown; INVOLVED
Cit.19406.1.S1_at	60.08476	28.42115	2.114086	AT2G36350.1	2.00E-32	protein kinase, putative
Cit.24319.1.S1_at	72.21704	34.20181	2.111498		NA	
Cit.30780.1.S1_s_at	201.7354	95.54395	2.111441	AT2G39080.1	1.00E-125	binding / catalytic
Cit.20526.1.S1_at	71.73294	34	2.109792		NA	
Cit.6154.1.S1_at	68.61675	32.54649	2.108269		NA	
Cit.15155.1.S1_at	79.9459	37.92801	2.107833	AT5G19130.2	3.00E-54	GPI transamidase component family protein / Gaa1-like
Cit.29652.1.S1_at	46.66153	22.17132	2.10459	AT2G04550.1	8.00E-85	IBR5 (INDOLE-3-BUTYRIC ACID RESPONSE 5); MAP kinase
Cit.26152.1.S1_at	144.2266	68.55366	2.10385		NA	
Cit.28240.1.S1_at	49.73277	23.64178	2.103597	AT3G43540.2	1.00E-12	unknown protein
Cit.8180.1.S1_x_at	46.76924	22.24836	2.102143		NA	
Cit.19573.1.S1_x_at	81.60026	38.83441	2.101236		NA	
Cit.5418.1.S1_s_at	146.9638	69.95599	2.100804	AT5G49190.1	1.00E-160	SUS2 (SUCROSE SYNTHASE 2); UDP-glycosyltransferase/
Cit.16564.1.S1_at	137.9965	65.70514	2.100239	AT5G48620.1	2.00E-11	disease resistance protein (CC-NBS-LRR class), putative
Cit.18278.1.S1_at	42.51144	20.2414	2.100222	AT5G65005.1	1.00E-14	nucleic acid binding
Cit.15093.1.S1_at	78.18216	37.25535	2.098549	AT3G61630.1	3.00E-42	CRF6 (CYTOKININ RESPONSE FACTOR 6); DNA binding / t
Cit.19955.1.S1_at	79.27254	37.81102	2.096546		NA	
Cit.23669.1.S1_at	205.8799	98.2195	2.09612	AT2G37420.1	2.00E-33	kinesin motor protein-related
RPTR-Cit-M57289-1_s	48.05329	22.92922	2.095723	NA	NA	NA
Cit.39068.1.S1_at	63.85762	30.47388	2.095487		NA	

Cit.27197.1.S1_at	152.6611	72.92113	2.09351	AT4G23180.1	2.00E-63	CRK10 (CYSTEINE-RICH RLK10); ATP binding / kinase/ pro
Cit.4950.1.S1_at	78.5672	37.52896	2.093509		NA	
Cit.40244.1.S1_s_at	153.1484	73.25619	2.090586	AT4G05050.3	8.00E-21	UBQ11 (UBIQUITIN 11); protein binding
Cit.6842.1.S1_at	606.1406	289.9545	2.090468	AT5G60930.1	4.00E-45	chromosome-associated kinesin, putative
Cit.32062.1.S1_at	58.04589	27.77212	2.090078		NA	
Cit.23712.1.S1_x_at	51.88317	24.83392	2.089206	AT1G17860.1	6.00E-12	trypsin and protease inhibitor family protein / Kunitz fan
Cit.31802.1.S1_at	48.89774	23.40499	2.089201		NA	
Cit.7595.1.S1_at	45.61222	21.83772	2.08869	AT1G17890.1	2.00E-14	GER2; binding / catalytic/ coenzyme binding
Cit.20242.1.S1_at	250.1812	119.8258	2.087874		NA	
Cit.23345.1.S1_a_at	134.1096	64.28851	2.086059		NA	
Cit.16245.1.S1_at	47.0358	22.54842	2.085991		NA	
Cit.6968.1.S1_at	185.288	88.83662	2.085716	AT1G26840.1	1.00E-101	ORC6 (ORIGIN RECOGNITION COMPLEX PROTEIN 6); DN/
Cit.7231.1.S1_at	61.99217	29.72551	2.085487		NA	
Cit.10661.1.S1_at	132.0929	63.42441	2.082682	AT1G12240.1	0	ATBETAFRUCT4; beta-fructofuranosidase/ hydrolase, hy
Cit.18116.1.S1_at	593.8352	285.498	2.079998	AT5G48850.1	3.00E-39	ATSDI1 (SULPHUR DEFICIENCY-INDUCED 1); binding
Cit.5856.1.S1_at	42.38601	20.38063	2.07972	AT5G06690.1	1.00E-55	WCRKC1 (WCRKC THIOREDOXIN 1)
Cit.31386.1.S1_at	57.01243	27.41624	2.079513	AT4G23660.2	2.00E-79	AtPPT1 (Arabidopsis thaliana polyprenyltransferase 1); 4
Cit.39737.1.S1_at	70.20091	33.78389	2.07794		NA	
Cit.25665.1.S1_at	83.17166	40.02669	2.077905	AT3G12920.1	1.00E-33	protein binding / zinc ion binding
Cit.1468.1.S1_at	53.07339	25.54983	2.07725	AT1G69840.6	1.00E-145	band 7 family protein
Cit.32165.1.S1_at	43.28096	20.84752	2.076072	AT3G22930.1	7.00E-56	calmodulin, putative
Cit.38521.1.S1_s_at	231.2906	111.5073	2.074219	AT2G38000.1	1.00E-163	chaperone protein dnaJ-related
Cit.18521.1.S1_at	64.23055	30.97784	2.073435	AT3G19000.1	4.00E-36	oxidoreductase, 2OG-Fe(II) oxygenase family protein
Cit.23299.1.S1_x_at	151.792	73.22503	2.072952	AT5G14030.4	5.00E-33	translocon-associated protein beta (TRAPB) family prote
Cit.22727.1.S1_at	404.4873	195.1566	2.072629	AT3G59970.3	5.00E-90	MTHFR1 (METHYLENETETRAHYDROFOLATE REDUCTASE
Cit.28560.1.S1_at	71.69686	34.59283	2.072593	AT3G14860.2	1.00E-19	NHL repeat-containing protein
Cit.37603.1.S1_at	49.63442	23.97294	2.070435	AT2G38560.1	5.00E-13	TFIIS (TRANSCRIPT ELONGATION FACTOR IIS); DNA bindi
Cit.38848.1.S1_at	48.79264	23.58377	2.068908		NA	
Cit.5045.1.S1_s_at	342.0812	165.6915	2.064567	AT2G23540.1	1.00E-103	GDSL-motif lipase/hydrolase family protein
Cit.6857.1.S1_x_at	190.7477	92.40794	2.064192	AT3G59660.1	1.00E-141	C2 domain-containing protein / GRAM domain-containir
Cit.21576.1.S1_at	136.2993	66.03947	2.063907		NA	
Cit.32887.1.S1_at	44.70434	21.66117	2.063801	AT4G33060.1	2.00E-15	peptidyl-prolyl cis-trans isomerase cyclophilin-type famil
Cit.15745.1.S1_at	167.3772	81.14888	2.062594		NA	
Cit.35483.1.S1_at	70.86597	34.36223	2.062322	AT5G27395.2	4.00E-27	P-P-bond-hydrolysis-driven protein transmembrane tran
Cit.16655.1.S1_at	130.5605	63.31723	2.062006	AT4G33990.1	5.00E-62	EMB2758 (embryo defective 2758)

Cit.24325.1.S1_at	46.25278	22.43321	2.061799	AT1G80260.1	6.00E-39	emb1427 (embryo defective 1427); tubulin binding
Cit.18315.1.S1_at	62.7404	30.45053	2.060404	AT5G60450.1	5.00E-39	ARF4 (AUXIN RESPONSE FACTOR 4); transcription factor
Cit.30945.1.S1_at	89.4283	43.41035	2.060069	AT2G18950.1	1.00E-84	HPT1 (HOMOGENTISATE PHYTYLTRANSFERASE 1); homo
Cit.20139.1.S1_x_at	628.6118	305.2482	2.059346		NA	
Cit.7899.1.S1_at	248.9247	120.9727	2.057693	AT3G27150.1	2.00E-89	kelch repeat-containing F-box family protein
Cit.40149.1.S1_at	136.7174	66.47529	2.056665	AT5G37630.1	4.00E-20	EMB2656 (EMBRYO DEFECTIVE 2656); binding
Cit.14612.1.S1_s_at	45.6116	22.18073	2.056362	AT1G67050.1	2.00E-54	unknown protein
Cit.7597.1.S1_at	62.54654	30.46991	2.052731	ATMG00860.1	8.00E-25	hypothetical protein
Cit.35543.1.S1_at	70.59644	34.40154	2.05213	AT4G19470.1	3.00E-16	disease resistance protein-related
Cit.34886.1.S1_at	48.75378	23.7816	2.050063		NA	
Cit.31959.1.S1_at	109.6158	53.49606	2.049044	AT4G15215.1	1.00E-42	PDR13; ATP binding / ATPase/ nucleoside-triphosphatas
Cit.21433.1.S1_x_at	55.84621	27.26923	2.047957		NA	
Cit.38717.1.S1_at	58.81392	28.72249	2.047661		NA	
Cit.10440.1.S1_at	88.09742	43.04666	2.046556	AT1G62040.1	8.00E-53	ATG8C (autophagy 8c); microtubule binding
Cit.21016.1.S1_at	46.42545	22.68784	2.04627		NA	
Cit.335.1.S1_at	45.81632	22.39742	2.045607	AT1G49660.1	1.00E-65	AtCXE5 (Arabidopsis thaliana carboxyesterase 5); carbox
Cit.20591.1.S1_at	219.2889	107.2318	2.044999		NA	
Cit.22554.1.S1_at	274.2577	134.1743	2.04404		NA	
Cit.30472.1.S1_at	88.87507	43.58415	2.03916	AT1G21110.1	3.00E-08	O-methyltransferase, putative
Cit.6952.1.S1_at	78.22814	38.37563	2.038485	AT2G42000.1	1.00E-15	plant EC metallothionein-like family 15 protein
Cit.17739.1.S1_at	146.8099	72.03925	2.037915		NA	
Cit.18938.1.S1_at	47.70891	23.41295	2.037715		NA	
Cit.36481.1.S1_at	74.13609	36.47234	2.032666	AT3G48140.1	4.00E-10	senescence-associated protein, putative
Cit.36470.1.S1_at	113.2989	55.74706	2.032374		NA	
Cit.24590.1.S1_x_at	74.71552	36.77698	2.031584	AT4G09750.1	1.00E-45	short-chain dehydrogenase/reductase (SDR) family prot
Cit.17133.1.S1_at	49.46341	24.35651	2.030809	AT4G08230.1	9.00E-40	glycine-rich protein
Cit.6910.1.S1_at	111.38	54.89408	2.028998	AT1G13030.1	6.00E-11	sphere organelles protein-related
Cit.29546.1.S1_at	41.84642	20.64376	2.027074	AT3G03900.1	1.00E-63	adenylylsulfate kinase, putative
Cit.16144.1.S1_at	1083.229	534.9567	2.024891	AT2G27035.1	1.00E-32	plastocyanin-like domain-containing protein
Cit.5918.1.S1_at	142.2516	70.31413	2.023087	AT4G04955.1	2.00E-89	ATALN (Arabidopsis allantoinase); allantoinase/ hydrolas
Cit.37328.1.S1_at	76.81606	38.03186	2.019782	AT3G03550.1	2.00E-60	zinc finger (C3HC4-type RING finger) family protein
Cit.29795.1.S1_at	60.1728	29.7928	2.019709	AT4G39120.1	4.00E-54	IMPL2 (MYO-INOSITOL MONOPHOSPHATASE LIKE 2); 3'(
Cit.25282.1.S1_at	47.84516	23.73265	2.016006	AT5G07090.2	4.00E-34	40S ribosomal protein S4 (RPS4B)
Cit.35779.1.S1_at	69.64487	34.5524	2.01563	AT3G61370.1	2.00E-25	unknown protein
Cit.29123.1.S1_at	270.569	134.2864	2.014865		NA	

Cit.7140.1.S1_at	114.2708	56.75284	2.013482		NA	
Cit.33920.1.S1_at	55.15875	27.42777	2.011055		NA	
Cit.30308.1.S1_at	62.9182	31.29213	2.010672	AT5G06350.1	3.00E-56	binding
Cit.11987.1.S1_s_at	4638.66	2307.252	2.01047	AT1G65060.1	0	4CL3; 4-coumarate-CoA ligase
Cit.5512.1.S1_at	414.2153	206.0363	2.0104	AT1G12030.1	7.00E-56	unknown protein
Cit.19005.1.S1_at	76.5496	38.09636	2.009368	AT1G47128.1	2.00E-16	RD21 (responsive to dehydration 21); cysteine-type endo
Cit.31126.1.S1_at	63.04053	31.37672	2.00915	AT1G08440.1	1.00E-95	CONTAINS InterPro DOMAIN/s: Uncharacterised protein
Cit.29815.1.S1_at	64.95577	32.38459	2.005762	AT1G13180.1	7.00E-73	DIS1 (DISTORTED TRICHOMES 1); ATP binding / actin bin
Cit.14993.1.S1_at	124.3209	61.98542	2.005647	AT4G15520.1	4.00E-91	tRNA/rRNA methyltransferase (SpoU) family protein
Cit.24043.1.S1_at	106.2606	53.0482	2.003095		NA	
Cit.5726.1.S1_at	467.4219	233.396	2.002699	AT2G32590.1	1.00E-102	INVOLVED IN: mitosis, mitotic cell cycle; LOCATED IN: nu
Cit.31319.1.S1_at	57.8264	28.9	2.000913	AT1G17840.1	4.00E-86	WBC11 (WHITE-BROWN COMPLEX HOMOLOG PROTEIN
Cit.15073.1.S1_at	357.5522	709.4796	-1.98427	AT2G03200.1	6.00E-48	aspartyl protease family protein
Cit.20398.1.S1_x_at	30.53787	61.09141	-2.00051		NA	
Cit.2809.1.S1_s_at	138.3214	277.0436	-2.0029	AT1G68840.1	1.00E-122	RAV2 (REGULATOR OF THE ATPASE OF THE VACUOLAR M
Cit.28805.1.S1_at	79.67775	159.8684	-2.00644		NA	
Cit.12693.1.S1_at	485.5418	974.9987	-2.00806	AT4G17370.1	1.00E-122	oxidoreductase family protein
Cit.25910.1.S1_x_at	1251.544	2514.115	-2.00881		NA	
Cit.15728.1.S1_at	74.47006	149.9075	-2.01299	AT3G63470.1	6.00E-47	scpl40 (serine carboxypeptidase-like 40); serine-type car
Cit.15241.1.S1_at	46.76015	94.20798	-2.01471	AT5G01070.2	4.00E-05	zinc finger (C3HC4-type RING finger) family protein
Cit.9706.1.S1_s_at	74.63794	150.5163	-2.01662	AT3G57270.1	1.00E-106	BG1 (BETA-1,3-GLUCANASE 1); catalytic/ cation binding /
Cit.6660.1.S1_at	76.5619	154.7475	-2.02121	AT5G07440.2	1.00E-100	GDH2 (GLUTAMATE DEHYDROGENASE 2); ATP binding /
Cit.21326.1.S1_at	133.6055	270.1142	-2.02173		NA	
Cit.23168.1.S1_at	385.3926	779.3094	-2.02212		NA	
Cit.17310.1.S1_s_at	64.11691	129.6731	-2.02245	AT1G32170.1	1.00E-130	XTR4 (XYLOGLUCAN ENDOTRANSGLYCOSYLASE 4); hydro
Cit.10087.1.S1_s_at	385.4087	779.7836	-2.02326	AT3G16770.1	2.00E-53	ATEBP (ETHYLENE-RESPONSIVE ELEMENT BINDING PROT
Cit.14156.1.S1_s_at	289.7029	586.4626	-2.02436	AT1G73500.1	1.00E-116	MKK9 (MAP KINASE KINASE 9); MAP kinase kinase/ kinas
Cit.11576.1.S1_at	568.0242	1149.952	-2.02448	AT1G30220.1	3.00E-87	INT2 (INOSITOL TRANSPORTER 2); carbohydrate transme
Cit.4720.1.S1_at	1075.744	2177.899	-2.02455	AT2G36970.1	1.00E-107	UDP-glucoronosyl/UDP-glucosyl transferase family prote
Cit.30622.1.S1_s_at	303.6679	615.0642	-2.02545		NA	
Cit.9552.1.S1_at	20.08569	40.69172	-2.02591	AT5G58710.1	1.00E-95	ROC7; peptidyl-prolyl cis-trans isomerase
Cit.22603.1.S1_s_at	431.6042	874.772	-2.02679	AT2G17880.1	3.00E-24	DNAJ heat shock protein, putative
Cit.25156.1.S1_x_at	21.54778	43.74567	-2.03017		NA	
Cit.29250.1.S1_s_at	333.2059	676.7353	-2.03098	AT5G18670.1	0	BMY3; beta-amylase/ catalytic/ cation binding
Cit.40392.1.S1_at	40.01586	81.40398	-2.03429	AT4G29210.2	2.00E-91	GGT4 (GAMMA-GLUTAMYL TRANSPEPTIDASE 4); gamma

Cit.8741.1.S1_s_at	519.7685	1057.771	-2.03508		NA	
Cit.12100.1.S1_at	505.8312	1029.801	-2.03586		NA	
Cit.3970.1.S1_s_at	35.23548	71.73986	-2.03601	AT3G53340.1	1.00E-67	NF-YB10 (NUCLEAR FACTOR Y, SUBUNIT B10); transcripti
Cit.17845.1.S1_at	46.16516	94.03996	-2.03703		NA	
Cit.17444.1.S1_at	183.5326	374.0813	-2.03823	AT5G10695.1	2.00E-21	unknown protein
Cit.36077.1.S1_s_at	52.58401	107.2671	-2.03992	AT2G45550.1	2.00E-44	CYP76C4; electron carrier/ heme binding / iron ion bindi
Cit.13639.1.S1_at	27.05341	55.19533	-2.04024	AT4G21192.2	1.00E-35	FUNCTIONS IN: molecular_function unknown; INVOLVED
Cit.24933.1.S1_at	32.54065	66.41483	-2.04098	AT3G54340.1	3.00E-27	AP3 (APETALA 3); DNA binding / transcription factor
Cit.7372.1.S1_at	84.72116	172.9546	-2.04146	AT4G24060.1	5.00E-05	Dof-type zinc finger domain-containing protein
Cit.23660.1.S1_x_at	22.08863	45.10339	-2.04193		NA	
Cit.17006.1.S1_s_at	1182.41	2415.076	-2.0425	AT3G03870.2	1.00E-37	unknown protein
Cit.996.1.S1_s_at	409.555	837.9836	-2.04608	AT1G01470.1	6.00E-54	LEA14 (LATE EMBRYOGENESIS ABUNDANT 14)
Cit.20174.1.S1_x_at	66.68995	136.4993	-2.04677		NA	
Cit.9940.1.S1_at	145.1467	297.2579	-2.04798	AT5G54190.1	0	PORA; oxidoreductase/ protochlorophyllide reductase
Cit.34418.1.S1_at	72.33515	148.1546	-2.04817	AT3G22930.1	9.00E-21	calmodulin, putative
Cit.25708.1.S1_s_at	47.96143	98.25123	-2.04855		NA	
Cit.15586.1.S1_at	88.24905	180.8459	-2.04927	AT4G27310.1	8.00E-44	zinc finger (B-box type) family protein
Cit.31007.1.S1_at	33.93285	69.54795	-2.04958	AT5G12120.1	5.00E-79	ubiquitin-associated (UBA)/TS-N domain-containing prot
Cit.20021.1.S1_at	124.2675	254.7191	-2.04976		NA	
Cit.6484.1.S1_at	20.08931	41.19374	-2.05053	AT3G14470.1	2.00E-56	disease resistance protein (NBS-LRR class), putative
Cit.17868.1.S1_at	77.18222	158.42	-2.05255	AT1G30220.1	7.00E-75	INT2 (INOSITOL TRANSPORTER 2); carbohydrate transme
Cit.13924.1.S1_s_at	54.70639	112.359	-2.05386	AT1G08550.2	8.00E-92	NPQ1 (NON-PHOTOCHEMICAL QUENCHING 1); violaxant
Cit.21913.1.S1_at	161.0852	331.0394	-2.05506	AT2G44970.2	5.00E-29	lipase-related
Cit.8767.1.S1_at	595.5118	1225.952	-2.05865	AT5G23760.1	3.00E-42	heavy-metal-associated domain-containing protein
Cit.22463.1.S1_s_at	205.4563	423.2757	-2.06017	AT4G27410.2	1.00E-110	RD26 (RESPONSIVE TO DESICCATION 26); transcription a
Cit.29611.1.S1_at	44.53359	91.76031	-2.06047	AT4G23810.1	4.00E-32	WRKY53; DNA binding / protein binding / transcription a
Cit.22386.1.S1_s_at	132.7239	273.5699	-2.0612	AT3G05540.1	3.00E-75	INVOLVED IN: biological_process unknown; LOCATED IN
Cit.30934.1.S1_s_at	36.07336	74.41747	-2.06295	AT2G37770.2	1.00E-133	aldo/keto reductase family protein
Cit.21254.1.S1_at	39.90219	82.3537	-2.06389	AT1G77180.2	3.00E-23	chromatin protein family
Cit.13460.1.S1_at	77.07018	159.0747	-2.06402	AT2G20142.1	1.00E-35	transmembrane receptor
Cit.40461.1.S1_s_at	34.02259	70.24284	-2.06459	AT5G16000.1	3.00E-80	NIK1 (NSP-INTERACTING KINASE 1); kinase
Cit.23349.1.S1_at	24.32648	50.25063	-2.06568	AT4G23160.1	2.00E-24	protein kinase family protein
Cit.30002.1.S1_at	28.95144	59.85708	-2.0675	AT3G15410.1	1.00E-137	leucine-rich repeat family protein
Cit.14768.1.S1_at	190.6778	394.262	-2.06769	AT1G30910.1	1.00E-106	molybdenum cofactor sulfurase family protein
Cit.25606.1.S1_at	140.7506	291.0823	-2.06807	AT5G43190.1	1.00E-77	F-box family protein (FBX6)

Cit.22527.1.S1_at	24.33711	50.36867	-2.06962	AT1G41830.1	8.00E-30	SKS6 (SKU5-SIMILAR 6); pectinesterase
Cit.18066.1.S1_at	432.1762	896.6891	-2.07482	AT1G15960.1	1.00E-111	NRAMP6; inorganic anion transmembrane transporter/ r
Cit.6850.1.S1_at	24.85382	51.57063	-2.07496	AT5G09320.1	2.00E-57	VPS9B
Cit.20798.1.S1_at	43.88273	91.06588	-2.07521		NA	
Cit.29750.1.S1_at	66.27848	137.5914	-2.07596	AT5G42680.1	8.00E-90	unknown protein
Cit.29313.1.S1_s_at	291.0065	604.5619	-2.07749	AT5G54770.1	1.00E-159	THI1; protein homodimerization
Cit.13851.1.S1_at	410.2883	853.8009	-2.08098	AT2G26670.1	1.00E-113	TED4 (REVERSAL OF THE DET PHENOTYPE 4); heme oxyg
Cit.37941.1.S1_at	56.43155	117.5272	-2.08265	AT1G76280.2	2.00E-23	pentatricopeptide (PPR) repeat-containing protein
Cit.4715.1.S1_s_at	133.2883	277.6222	-2.08287	AT4G37550.1	0	formamidase, putative / formamide amidohydrolase, pu
Cit.23417.1.S1_at	48.27237	100.641	-2.08486	AT3G32930.1	4.00E-58	unknown protein
Cit.33020.1.S1_s_at	30.32515	63.22678	-2.08496	AT5G02500.1	1.00E-51	HSC70-1 (HEAT SHOCK COGNATE PROTEIN 70-1); ATP bi
Cit.7100.1.S1_x_at	152.9297	318.9995	-2.08592		NA	
Cit.17418.1.S1_s_at	388.9862	812.1706	-2.08792	AT1G17840.1	0	WBC11 (WHITE-BROWN COMPLEX HOMOLOG PROTEIN
Cit.23598.1.S1_s_at	194.3636	406.1969	-2.08988	AT5G15500.2	1.00E-07	ankyrin repeat family protein
Cit.22158.1.S1_x_at	3104.326	6499.874	-2.09381	AT1G17860.1	3.00E-12	trypsin and protease inhibitor family protein / Kunitz fan
Cit.26406.1.S1_at	37.97782	79.67316	-2.09789	AT5G35960.1	5.00E-62	protein kinase, putative
Cit.20929.1.S1_at	215.0196	451.147	-2.09817	AT5G03560.1	1.00E-10	nucleobase:cation symporter
Cit.1828.1.S1_at	35.83118	75.29874	-2.10149	AT5G06730.1	1.00E-117	peroxidase, putative
Cit.22474.1.S1_at	25.11813	52.85112	-2.1041	AT5G63190.2	5.00E-51	MA3 domain-containing protein
Cit.6784.1.S1_at	31.36374	66.01425	-2.1048	AT5G46560.1	1.00E-103	unknown protein
Cit.35257.1.S1_at	83.21461	175.6588	-2.11091		NA	
Cit.21866.1.S1_x_at	28.68804	60.60376	-2.11251		NA	
Cit.15284.1.S1_at	532.7784	1125.929	-2.11332	AT2G31090.1	6.00E-34	unknown protein
Cit.32593.1.S1_at	124.0568	262.3257	-2.11456		NA	
Cit.28009.1.S1_at	31.90847	67.49152	-2.11516	AT1G20860.1	2.00E-57	phosphate transporter family protein
Cit.29301.1.S1_s_at	139.0519	294.2256	-2.11594	AT3G13227.1	5.00E-07	serine-rich protein-related
Cit.18624.1.S1_at	50.25869	106.375	-2.11655		NA	
Cit.826.1.S1_s_at	54.40003	115.2102	-2.11783	AT1G30270.1	0	CIPK23 (CBL-INTERACTING PROTEIN KINASE 23); kinase/
Cit.3896.1.S1_at	70.40036	149.215	-2.11952	AT1G27340.1	5.00E-70	F-box family protein
Cit.15578.1.S1_at	23.43666	49.73149	-2.12195	AT5G19210.2	1.00E-164	DEAD/DEAH box helicase, putative
Cit.4683.1.S1_at	43.53212	92.38211	-2.12216	AT1G07030.1	1.00E-127	mitochondrial substrate carrier family protein
Cit.5624.1.S1_at	58.83103	125.0481	-2.12555	AT5G40240.1	1.00E-104	nodulin MtN21 family protein
Cit.6707.1.S1_at	64.08326	136.22	-2.12567	AT1G64770.1	1.00E-128	NDF2 (NDH-DEPENDENT CYCLIC ELECTRON FLOW 1); car
Cit.20228.1.S1_s_at	44.34507	94.26729	-2.12577	AT1G01220.1	4.00E-91	GHMP kinase-related
Cit.15523.1.S1_at	218.2045	463.8569	-2.12579	AT3G26330.1	5.00E-71	CYP71B37; electron carrier/ heme binding / iron ion binc

Cit.40221.1.S1_s_at	37.47299	79.76449	-2.12859		NA	
Cit.14471.1.S1_at	306.974	654.0621	-2.13068		NA	
Cit.12569.1.S1_s_at	333.1132	710.2679	-2.13221	AT3G16350.1	1.00E-109	myb family transcription factor
Cit.9324.1.S1_at	150.5647	321.5823	-2.13584	AT3G14680.1	1.00E-64	CYP72A14; electron carrier/ heme binding / iron ion binc
Cit.2959.1.S1_at	558.0472	1193.118	-2.13802	AT5G13000.2	0	ATGSL12 (glucan synthase-like 12); 1,3-beta-glucan syntl
Cit.36841.1.S1_s_at	48.91817	104.6177	-2.13863	AT1G21550.1	2.00E-32	calcium-binding protein, putative
Cit.10700.1.S1_s_at	428.0629	916.378	-2.14076	AT1G01720.1	1.00E-116	ATAF1; transcription activator/ transcription factor
Cit.28003.1.S1_at	31.48502	67.53298	-2.14492	AT3G24420.1	8.00E-45	hydrolase, alpha/beta fold family protein
Cit.28138.1.S1_at	26.58874	57.10115	-2.14757	AT5G24330.1	2.00E-83	ATXR6; DNA binding / protein binding
Cit.5228.1.S1_s_at	240.4175	516.525	-2.14845	AT3G51550.1	4.00E-45	FER (FERONIA); kinase/ protein kinase
Cit.15506.1.S1_at	59.75276	128.617	-2.15249	AT2G36750.1	6.00E-77	UGT73C1 (UDP-GLUCOSYL TRANSFERASE 73C1); UDP-glu
Cit.29416.1.S1_at	239.422	515.597	-2.15351	AT5G14000.1	9.00E-42	anac084 (Arabidopsis NAC domain containing protein 84
Cit.5127.1.S1_at	202.1713	435.7902	-2.15555	AT3G51000.1	1.00E-106	epoxide hydrolase, putative
Cit.15676.1.S1_at	305.1675	658.4053	-2.15752	AT3G08720.2	6.00E-75	S6K2 (ARABIDOPSIS THALIANA SERINE/THREONINE PRO
Cit.5961.1.S1_at	20.02581	43.22586	-2.15851	AT2G38905.1	1.00E-25	hydrophobic protein, putative / low temperature and sa
Cit.17629.1.S1_x_at	1255.7	2713.437	-2.1609		NA	
Cit.12739.1.S1_at	184.2608	398.4667	-2.16251		NA	
Cit.7312.1.S1_at	234.8772	508.0032	-2.16285	AT2G36630.1	2.00E-23	unknown protein
Cit.2902.1.S1_at	29.54941	64.1937	-2.17242	AT1G16720.1	0	HCF173 (high chlorophyll fluorescence phenotype 173);
Cit.26004.1.S1_at	29.70736	64.53708	-2.17243		NA	
Cit.11872.1.S1_at	784.8235	1705.454	-2.17304	AT3G19990.1	0	unknown protein
Cit.24477.1.S1_s_at	1077.958	2343.158	-2.1737	AT5G26230.1	7.00E-74	unknown protein
Cit.18118.1.S1_at	40.37991	87.80596	-2.1745	AT5G62150.1	1.00E-27	peptidoglycan-binding LysM domain-containing protein
Cit.21040.1.S1_s_at	258.4071	563.1721	-2.1794	AT5G11720.1	6.00E-73	alpha-glucosidase 1 (AGLU1)
Cit.1718.1.S1_s_at	32.5483	71.10734	-2.18467	AT2G19590.1	1.00E-127	ACO1 (ACC OXIDASE 1); 1-aminocyclopropane-1-carboxy
Cit.17047.1.S1_at	20.82249	45.50016	-2.18515		NA	
Cit.38799.1.S1_at	24.78105	54.20155	-2.18722		NA	
Cit.8514.1.S1_x_at	347.6231	760.3519	-2.18729	AT2G38380.1	1.00E-109	peroxidase 22 (PER22) (P22) (PRXEA) / basic peroxidase
Cit.21705.1.S1_s_at	40.07718	87.66792	-2.18748	AT3G25400.1	1.00E-47	FUNCTIONS IN: molecular_function unknown; INVOLVED
Cit.18242.1.S1_at	172.351	377.1031	-2.18799		NA	
Cit.21981.1.S1_x_at	20.94997	45.88894	-2.19041	AT2G16770.1	5.00E-16	DNA binding / transcription factor
Cit.1270.1.S1_s_at	101.7996	223.0692	-2.19126	AT5G61600.1	4.00E-45	ethylene-responsive element-binding family protein
Cit.20637.1.S1_at	41.75358	91.50052	-2.19144	AT5G40800.1	6.00E-28	unknown protein
Cit.814.1.S1_s_at	39.16122	85.88326	-2.19307	AT3G01500.3	1.00E-134	CA1 (CARBONIC ANHYDRASE 1); carbonate dehydratase/
Cit.6100.1.S1_at	93.98643	206.3306	-2.19532	AT1G01180.1	1.00E-111	unknown protein

Cit.4886.1.S1_at	110.315	242.1794	-2.19534	AT2G43760.3	6.00E-73	molybdopterin biosynthesis MoaE family protein
Cit.5457.1.S1_at	118.6525	260.692	-2.1971	AT5G06280.3	3.00E-15	unknown protein
Cit.29048.1.S1_x_at	596.0098	1310.777	-2.19925		NA	
Cit.21258.1.S1_at	22.0146	48.44437	-2.20056		NA	
Cit.20007.1.S1_at	38.20741	84.09644	-2.20105		NA	
Cit.1501.1.S1_at	27.75952	61.1235	-2.20189	AT5G20570.1	2.00E-64	RBX1 (RING-BOX 1); protein binding
Cit.39201.1.S1_s_at	58.14543	128.1948	-2.20473	AT1G21000.2	1.00E-100	zinc-binding family protein
Cit.17371.1.S1_at	490.2476	1080.866	-2.20473	AT3G25590.1	5.00E-29	unknown protein
Cit.26217.1.S1_at	129.57	285.7757	-2.20557	AT5G60710.1	5.00E-28	zinc finger (C3HC4-type RING finger) family protein
Cit.18295.1.S1_at	35.56761	78.54118	-2.20822		NA	
Cit.5008.1.S1_at	20.21929	44.67983	-2.20976	AT3G17970.1	9.00E-67	atToc64-III (Arabidopsis thaliana translocon at the outer
Cit.34356.1.S1_at	29.03879	64.33697	-2.21555		NA	
Cit.3863.1.S1_x_at	163.0891	361.7408	-2.21806	AT4G14342.1	8.00E-45	pre-mRNA splicing factor 10 kDa subunit, putative
Cit.21700.1.S1_at	230.0898	510.5292	-2.21883	AT3G52430.1	8.00E-43	PAD4 (PHYTOALEXIN DEFICIENT 4); lipase/ protein bindi
Cit.23657.1.S1_at	26.97717	59.88253	-2.21975		NA	
Cit.7233.1.S1_at	35.30339	78.38332	-2.22028	AT3G18830.1	1.00E-152	ATPLT5 (POLYOL TRANSPORTER 5); D-ribose transmemb
Cit.20015.1.S1_at	261.4671	581.069	-2.22234	AT1G74250.1	7.00E-11	DNAJ heat shock N-terminal domain-containing protein
Cit.17589.1.S1_x_at	252.2012	560.9507	-2.22422	AT4G02380.1	8.00E-15	SAG21 (SENESCENCE-ASSOCIATED GENE 21)
Cit.18353.1.S1_x_at	4860.774	10817.1	-2.22539	AT3G15353.1	7.00E-18	MT3 (METALLOTHIONEIN 3); copper ion binding
Cit.39452.1.S1_at	29.74916	66.20438	-2.22542	AT2G03050.1	3.00E-30	mitochondrial transcription termination factor-related /
Cit.3693.1.S1_at	802.5059	1785.945	-2.22546	AT2G29380.1	9.00E-68	protein phosphatase 2C, putative / PP2C, putative
Cit.14271.1.S1_at	71.1635	158.4647	-2.22677	AT2G38870.1	7.00E-05	protease inhibitor, putative
Cit.17360.1.S1_s_at	104.1101	232.2608	-2.23092		NA	
Cit.23915.1.S1_at	59.79917	133.5342	-2.23304	AT1G02260.1	3.00E-40	transmembrane protein, putative
Cit.26945.1.S1_s_at	23.98595	53.60261	-2.23475		NA	
Cit.19129.1.S1_at	74.88435	167.4137	-2.23563	AT1G03220.1	9.00E-36	extracellular dermal glycoprotein, putative / EDGP, puta
Cit.12141.1.S1_s_at	257.1752	578.6985	-2.25021	AT3G08860.1	0	alanine--glyoxylate aminotransferase, putative / beta-ala
Cit.32752.1.S1_at	20.42929	46.02	-2.25265		NA	
Cit.21968.1.S1_at	36.76884	82.90663	-2.25481	AT4G00230.1	3.00E-64	XSP1 (xylem serine peptidase 1); identical protein bindin
Cit.9345.1.S1_at	48.77634	110.1483	-2.25823	AT5G52570.1	7.00E-98	BETA-OHASE 2 (BETA-CAROTENE HYDROXYLASE 2); caro
Cit.12543.1.S1_at	183.6957	415.8656	-2.26388	AT1G15380.2	6.00E-70	lactoylglutathione lyase family protein / glyoxalase I fam
Cit.17204.1.S1_at	27.26788	61.76584	-2.26515	AT3G56710.1	1.00E-07	SIB1 (SIGMA FACTOR BINDING PROTEIN 1); binding / pro
Cit.29554.1.S1_at	33.43964	75.83121	-2.2677	AT3G50670.2	6.00E-79	U1-70K (U1 SMALL NUCLEAR RIBONUCLEOPROTEIN-70K
Cit.60.1.S1_at	192.5267	436.74	-2.26846	AT2G38540.1	1.00E-16	LP1; calmodulin binding
Cit.17150.1.S1_at	125.2994	284.2928	-2.26891	AT3G03470.1	4.00E-33	CYP89A9; electron carrier/ heme binding / iron ion bindi

Cit.4525.1.S1_at	107.5646	244.3383	-2.27155	AT4G34131.1	7.00E-30	UGT73B3 (UDP-glucosyl transferase 73B3); UDP-glycosyl
Cit.9723.1.S1_s_at	627.2628	1426.295	-2.27384	AT4G20260.3	5.00E-57	DREPP plasma membrane polypeptide family protein
Cit.17981.1.S1_at	376.0373	855.4324	-2.27486	AT3G61440.1	1.00E-168	CYSC1 (CYSTEINE SYNTHASE C1); L-3-cyanoalanine synth
Cit.27186.1.S1_at	25.01687	56.99657	-2.27833	AT2G39940.1	3.00E-57	COI1 (CORONATINE INSENSITIVE 1); protein binding / ub
Cit.1113.1.S1_s_at	471.1482	1073.48	-2.27843	AT1G59950.1	3.00E-99	aldo/keto reductase, putative
Cit.10334.1.S1_at	205.1299	467.5904	-2.27948	AT1G37130.1	0	NIA2 (NITRATE REDUCTASE 2); nitrate reductase (NADH)
Cit.11423.1.S1_at	45.49938	103.7224	-2.27964	AT4G20070.1	1.00E-111	ATAAH (Arabidopsis thaliana Allantoate Amidohydrolase
Cit.18325.1.S1_s_at	779.3229	1776.584	-2.27965	AT1G06570.1	2.00E-63	PDS1 (PHYTOENE DESATURATION 1); 4-hydroxyphenylpy
Cit.29732.1.S1_at	40.9854	93.47171	-2.28061	AT3G12550.1	2.00E-22	XH/XS domain-containing protein / XS zinc finger domair
Cit.6364.1.S1_s_at	51.53042	117.7843	-2.28572	AT2G15220.1	3.00E-83	secretory protein, putative
Cit.17337.1.S1_at	112.2041	256.634	-2.28721	AT5G20230.1	5.00E-19	ATBCB (ARABIDOPSIS BLUE-COPPER-BINDING PROTEIN);
Cit.39832.1.S1_s_at	44.47234	101.7389	-2.28769	AT1G06430.1	0	FTSH8; ATP-dependent peptidase/ ATPase/ metallopept
Cit.3817.1.S1_x_at	716.0349	1640.747	-2.29143	AT4G22920.1	1.00E-99	NYE1 (NON-YELLOWING 1)
Cit.6420.1.S1_at	314.2408	721.3666	-2.29559	AT1G44446.1	2.00E-74	CH1 (CHLORINA 1); chlorophyllide a oxygenase
Cit.10453.1.S1_s_at	356.2624	819.1005	-2.29915		NA	
Cit.22776.1.S1_s_at	398.8281	917.178	-2.29968	AT4G32480.1	5.00E-71	unknown protein
Cit.18537.1.S1_at	41.87538	96.30228	-2.29974	AT2G15890.1	5.00E-18	MEE14 (maternal effect embryo arrest 14)
Cit.35756.1.S1_at	21.13246	48.70468	-2.30473	AT3G59850.1	3.00E-94	polygalacturonase, putative / pectinase, putative
Cit.31923.1.S1_at	25.24755	58.19915	-2.30514		NA	
Cit.12768.1.S1_at	56.18879	129.9348	-2.31247	AT2G34790.1	4.00E-75	MEE23 (MATERNAL EFFECT EMBRYO ARREST 23); FAD bi
Cit.927.1.S1_at	21.89904	50.6978	-2.31507	AT1G56340.1	0	CRT1 (CALRETICULIN 1); calcium ion binding / unfolded p
Cit.31451.1.S1_s_at	551.3301	1277.18	-2.31654	AT1G11530.1	5.00E-37	ATCXXS1 (C-terminal cysteine residue is changed to a ser
Cit.30123.1.S1_at	114.0772	264.6323	-2.31977		NA	
Cit.24213.1.S1_s_at	21.00734	48.73682	-2.31999	AT1G68570.1	3.00E-74	proton-dependent oligopeptide transport (POT) family p
Cit.18986.1.S1_s_at	627.0824	1454.907	-2.32012	AT5G18840.1	2.00E-68	sugar transporter, putative
Cit.18450.1.S1_at	54.41023	126.4715	-2.32441		NA	
Cit.38924.1.S1_at	23.41856	54.4763	-2.3262	AT4G33440.1	1.00E-37	glycoside hydrolase family 28 protein / polygalacturonas
Cit.523.1.S1_at	95.59684	222.7485	-2.33008		NA	
Cit.28127.1.S1_at	59.51415	138.7012	-2.33056	AT3G47570.1	1.00E-53	leucine-rich repeat transmembrane protein kinase, puta
Cit.13671.1.S1_s_at	151.0626	352.0668	-2.3306	AT3G51895.1	5.00E-97	SULTR3;1 (SULFATE TRANSPORTER 3;1); secondary activ
Cit.16192.1.S1_at	185.8451	433.1548	-2.33073	AT2G04495.1	7.00E-21	unknown protein
Cit.37764.1.S1_s_at	99.88866	232.9196	-2.33179	AT1G27730.1	7.00E-57	STZ (salt tolerance zinc finger); nucleic acid binding / trai
Cit.14133.1.S1_at	118.5933	277.4489	-2.3395	AT5G15120.1	3.00E-49	unknown protein
Cit.16208.1.S1_at	24.90445	58.26665	-2.33961	AT3G50930.1	9.00E-32	BCS1 (CYTOCHROME BC1 SYNTHESIS); ATP binding / ATP
Cit.39466.1.S1_at	26.71766	62.61628	-2.34363		NA	

Cit.17724.1.S1_s_at	92.60017	217.2653	-2.34627	AT4G25810.1	1.00E-130	XTR6 (XYLOGLUCAN ENDOTRANSGLYCOSYLASE 6); hydro
Cit.7192.1.S1_at	921.399	2165.228	-2.34994	AT3G25140.1	3.00E-22	QUA1 (QUASIMODO 1); polygalacturonate 4-alpha-galac
Cit.36616.1.S1_at	26.16125	61.54217	-2.35242		NA	
Cit.13250.1.S1_s_at	680.1361	1600.402	-2.35306	AT4G12290.1	0	amine oxidase/ copper ion binding / quinone binding
Cit.352.1.S1_s_at	1249.741	2940.869	-2.35318	AT2G23250.1	1.00E-110	UGT84B2 (UDP-glucosyl transferase 84B2); UDP-glycosyl
Cit.38398.1.S1_at	21.45606	50.52409	-2.35477	AT1G68185.1	4.00E-11	ubiquitin-related
Cit.14926.1.S1_at	193.5171	456.4372	-2.35864	AT2G04520.1	1.00E-73	eukaryotic translation initiation factor 1A, putative / eIF-
Cit.11209.1.S1_s_at	89.59924	211.3344	-2.35866	AT2G40000.1	1.00E-151	HSPRO2 (ARABIDOPSIS ORTHOLOG OF SUGAR BEET HS1
Cit.39488.1.S1_at	23.24012	54.82512	-2.35907		NA	
Cit.20397.1.S1_at	34.53413	81.48791	-2.35963		NA	
Cit.13724.1.S1_s_at	146.2823	345.36	-2.36091	AT5G60900.1	1.00E-106	RLK1 (RECEPTOR-LIKE PROTEIN KINASE 1); ATP binding /
Cit.37306.1.S1_at	31.7106	74.97669	-2.3644	AT4G22250.1	2.00E-10	zinc finger (C3HC4-type RING finger) family protein
Cit.15267.1.S1_at	143.7085	339.8783	-2.36505	AT1G72190.1	1.00E-70	oxidoreductase family protein
Cit.25318.1.S1_at	23.82923	56.41439	-2.36744	AT2G16710.2	6.00E-21	hesB-like domain-containing protein
Cit.39117.1.S1_at	54.93674	130.1397	-2.3689	AT2G44800.1	2.00E-22	oxidoreductase, 2OG-Fe(II) oxygenase family protein
Cit.11764.1.S1_s_at	160.5745	380.5793	-2.37011	AT3G11690.1	3.00E-32	unknown protein
Cit.17065.1.S1_s_at	962.7527	2282.115	-2.37041	AT1G23800.1	1.00E-112	ALDH2B7; 3-chloroallyl aldehyde dehydrogenase/ aldehy
Cit.12316.1.S1_s_at	75.7561	179.6435	-2.37134	AT1G31200.1	2.00E-41	ATPP2-A9 (Phloem protein 2-A9); carbohydrate binding
Cit.25296.1.S1_at	209.3865	496.6704	-2.37203	AT5G13580.1	6.00E-45	ABC transporter family protein
Cit.37460.1.S1_at	32.29637	76.65844	-2.37359	AT1G75620.1	2.00E-64	glyoxal oxidase-related
Cit.6412.1.S1_at	98.01566	232.7577	-2.3747	AT2G33580.1	3.00E-32	protein kinase family protein / peptidoglycan-binding Ly:
Cit.17346.1.S1_s_at	53.0323	126.2994	-2.38156	AT1G31335.1	2.00E-06	unknown protein
Cit.20042.1.S1_at	35.40173	84.96697	-2.40008	AT1G68765.1	6.00E-06	IDA (INFLORESCENCE DEFICIENT IN ABSCISSION); recepto
Cit.7500.1.S1_at	43.89544	105.3736	-2.40056	AT1G20270.1	2.00E-07	oxidoreductase, 2OG-Fe(II) oxygenase family protein
Cit.4868.1.S1_at	297.2694	713.7169	-2.40091	AT3G15840.1	3.00E-99	PIFI (post-illumination chlorophyll fluorescence increase
Cit.29252.1.S1_at	27.66301	66.4423	-2.40185	AT3G48090.2	9.00E-39	EDS1 (enhanced disease susceptibility 1); lipase/ signal t
Cit.5855.1.S1_at	398.2986	958.7598	-2.40714	AT1G30110.1	4.00E-85	ATNUDX25 (ARABIDOPSIS THALIANA NUDIX HYDROLASE
Cit.30976.1.S1_at	34.08673	82.0555	-2.40726	AT3G12650.1	8.00E-45	unknown protein
Cit.5941.1.S1_at	36.12833	87.05357	-2.40957		NA	
Cit.10380.1.S1_at	31.01792	74.81695	-2.41206	AT1G78680.1	1.00E-138	ATGGH2 (gamma-glutamyl hydrolase 2); omega peptidas
Cit.23393.1.S1_x_at	94.98652	229.3978	-2.41506	AT5G21160.1	2.00E-24	La domain-containing protein / proline-rich family protei
Cit.11286.1.S1_at	486.7425	1176.073	-2.41621	AT1G63840.1	1.00E-11	zinc finger (C3HC4-type RING finger) family protein
Cit.14342.1.S1_at	54.48215	131.6525	-2.41643	AT3G62150.1	9.00E-65	PGP21 (P-GLYCOPROTEIN 21); ATPase, coupled to transr
Cit.24588.1.S1_at	26.08094	63.04915	-2.41744		NA	
CitAffx.1.1.S1_at	65.0128	157.508	-2.42272	NA	NA	NA

Cit.20630.1.S1_at	31.25185	75.98725	-2.43145	AT3G48000.1	1.00E-22	ALDH2B4 (ALDEHYDE DEHYDROGENASE 2B4); 3-chloroal
Cit.14346.1.S1_at	39.98579	97.269	-2.43259	AT5G43630.1	2.00E-38	TZP; DNA binding / nucleic acid binding / zinc ion binding
Cit.19293.1.S1_at	24.29317	59.11144	-2.43325	AT5G02020.1	3.00E-05	unknown protein
Cit.13915.1.S1_at	448.5142	1092.217	-2.43519	AT3G14440.1	0	NCED3 (NINE-CIS-EPOXYCAROTENOID DIOXYGENASE 3);
Cit.38659.1.S1_at	23.29679	56.73911	-2.43549		NA	
Cit.32038.1.S1_at	26.91114	65.63896	-2.4391	AT4G29060.1	4.00E-56	emb2726 (embryo defective 2726); RNA binding / transl
Cit.24514.1.S1_at	20.30657	49.54252	-2.43973	AT3G22990.1	7.00E-56	LFR (LEAF AND FLOWER RELATED); binding
Cit.16418.1.S1_at	57.66511	140.6929	-2.43983	AT5G41040.2	1.00E-60	transferase family protein
Cit.20434.1.S1_x_at	27.35933	66.87507	-2.44432	AT4G23400.1	1.00E-144	PIP1;5 (PLASMA MEMBRANE INTRINSIC PROTEIN 1;5); w
Cit.29625.1.S1_at	23.76567	58.19978	-2.4489	AT2G30020.1	1.00E-63	protein phosphatase 2C, putative / PP2C, putative
Cit.6860.1.S1_at	29.60572	72.6319	-2.45331	AT3G21690.1	6.00E-64	MATE efflux family protein
Cit.22887.1.S1_at	274.5867	674.4666	-2.4563		NA	
Cit.8401.1.S1_at	574.8199	1412.802	-2.45782	AT3G14420.2	1.00E-179	(S)-2-hydroxy-acid oxidase, peroxisomal, putative / glyco
Cit.4690.1.S1_at	524.4738	1289.251	-2.45818	AT5G07990.1	1.00E-109	TT7 (TRANSPARENT TESTA 7); flavonoid 3'-monooxygena
Cit.11683.1.S1_at	47.73069	117.637	-2.4646		NA	
Cit.5538.1.S1_s_at	41.7428	102.9194	-2.46556	AT4G21215.2	5.00E-17	unknown protein
Cit.9132.1.S1_s_at	146.8384	362.3705	-2.46782	AT4G19420.1	1.00E-156	pectinacetylesterase family protein
Cit.3757.1.S1_at	119.1561	294.3563	-2.47034	AT2G46400.1	2.00E-06	WRKY46; transcription factor
Cit.16767.1.S1_at	38.06171	94.06533	-2.47139	AT1G80160.1	2.00E-27	lactoylglutathione lyase family protein / glyoxalase I fam
Cit.34473.1.S1_at	31.27627	77.39111	-2.47444		NA	
Cit.1866.1.S1_s_at	951.601	2355.913	-2.47574	AT2G22470.1	9.00E-24	AGP2 (ARABINOGALACTAN PROTEIN 2)
Cit.7343.1.S1_s_at	34.57864	85.66774	-2.47748	AT5G01830.1	1.00E-131	armadillo/beta-catenin repeat family protein / U-box do
Cit.9650.1.S1_at	28.87614	71.54578	-2.47768	AT1G74950.1	4.00E-05	TIFY10B
Cit.19737.1.S1_x_at	488.3535	1211.329	-2.48043	AT3G15353.1	6.00E-17	MT3 (METALLOTHIONEIN 3); copper ion binding
Cit.30769.1.S1_at	30.12014	74.80701	-2.48362		NA	
Cit.29267.1.S1_s_at	271.1964	674.2847	-2.48633	AT3G44735.1	2.00E-11	PSK1; growth factor
Cit.29831.1.S1_at	26.16859	65.07681	-2.48683	AT1G28510.1	6.00E-59	FUNCTIONS IN: molecular_function unknown; INVOLVED
Cit.14749.1.S1_at	139.8624	348.3997	-2.49102	AT1G22360.1	3.00E-94	AtUGT85A2 (UDP-glucosyl transferase 85A2); UDP-glyco
Cit.3665.1.S1_s_at	658.3901	1642.243	-2.49433	AT1G75750.1	2.00E-30	GASA1 (GAST1 PROTEIN HOMOLOG 1)
Cit.16960.1.S1_x_at	92.65069	231.4115	-2.49768		NA	
Cit.12252.1.S1_at	224.8453	564.0458	-2.5086	AT2G14960.1	0	GH3.1
Cit.22731.1.S1_s_at	2126.561	5344.12	-2.51303	AT1G61080.1	3.00E-23	proline-rich family protein
Cit.13671.1.S1_at	971.9364	2442.958	-2.5135	AT3G51895.1	5.00E-97	SULTR3;1 (SULFATE TRANSPORTER 3;1); secondary activ
Cit.10014.1.S1_s_at	92.16019	232.0472	-2.51787	AT4G10265.1	7.00E-22	wound-responsive protein, putative
Cit.24388.1.S1_s_at	373.4438	940.7201	-2.51904	AT5G11580.1	1.00E-148	UVB-resistance protein-related / regulator of chromosor

Cit.9240.1.S1_at	217.183	548.108	-2.52372	AT5G25610.1	1.00E-118	RD22; nutrient reservoir
Cit.4030.1.S1_at	126.1649	318.4079	-2.52374	AT1G60190.1	4.00E-82	armadillo/beta-catenin repeat family protein / U-box do
Cit.15901.1.S1_at	28.58227	72.18136	-2.52539		NA	
Cit.15433.1.S1_at	399.9059	1011.47	-2.52927	AT5G11720.1	6.00E-73	alpha-glucosidase 1 (AGLU1)
Cit.8351.1.S1_s_at	37.00224	93.60409	-2.52969	AT4G35090.1	0	CAT2 (CATALASE 2); catalase
Cit.22783.1.S1_at	42.59224	107.7704	-2.53028	AT1G27752.2	3.00E-31	FUNCTIONS IN: molecular_function unknown; INVOLVED
Cit.18432.1.S1_at	45.30999	114.8785	-2.53539	AT3G54420.1	9.00E-37	ATEP3; chitinase
Cit.24463.1.S1_s_at	177.1645	449.5148	-2.53727	AT3G14680.1	1.00E-64	CYP72A14; electron carrier/ heme binding / iron ion binc
Cit.3042.1.S1_s_at	344.8614	875.4963	-2.53869	AT1G52565.1	1.00E-31	unknown protein
Cit.17291.1.S1_at	74.03452	187.9558	-2.53876	AT5G54160.1	1.00E-100	ATOMT1 (O-METHYLTRANSFERASE 1); caffeate O-methy
Cit.11882.1.S1_at	22.10922	56.14996	-2.53966		NA	
Cit.30834.1.S1_at	20.79716	52.86601	-2.54198	AT1G20780.1	3.00E-10	SAUL1 (SENESCENCE-ASSOCIATED E3 UBIQUITIN LIGASE
Cit.32003.1.S1_at	49.81606	126.7977	-2.54532		NA	
Cit.10457.1.S1_s_at	2202.708	5607.434	-2.5457	AT3G25180.1	1.00E-121	CYP82G1; electron carrier/ heme binding / iron ion bindi
Cit.10152.1.S1_s_at	157.2518	400.719	-2.54826	AT4G27410.2	1.00E-110	RD26 (RESPONSIVE TO DESICCATION 26); transcription a
Cit.1866.1.S1_at	164.6123	419.7223	-2.54976	AT2G22470.1	7.00E-23	AGP2 (ARABINOGALACTAN PROTEIN 2)
Cit.25526.1.S1_s_at	507.8903	1295.403	-2.55056	AT3G14620.1	3.00E-55	CYP72A8; electron carrier/ heme binding / iron ion bindi
Cit.22352.1.S1_at	48.57299	123.9687	-2.55221	AT1G02070.1	6.00E-13	unknown protein
Cit.3140.1.S1_x_at	288.1572	735.6335	-2.55289	AT1G59710.1	3.00E-45	FUNCTIONS IN: molecular_function unknown; INVOLVED
Cit.39779.1.S1_at	27.32221	70.02305	-2.56286	AT4G28530.1	8.00E-73	anac074 (Arabidopsis NAC domain containing protein 74
Cit.17413.1.S1_at	49.93974	128.0463	-2.56402	AT4G33467.2	5.00E-09	unknown protein
Cit.18642.1.S1_s_at	157.1214	403.1954	-2.56614	AT1G20030.2	3.00E-79	pathogenesis-related thaumatin family protein
Cit.696.1.S1_x_at	71.05876	182.4653	-2.56781		NA	
Cit.40231.1.S1_s_at	675.6241	1737.865	-2.57224	AT5G59030.1	3.00E-46	COPT1 (copper transporter 1); copper ion transmembrar
Cit.20176.1.S1_at	208.3862	536.2667	-2.57343		NA	
Cit.21853.1.S1_at	82.91721	213.532	-2.57524	AT5G42830.1	7.00E-24	transferase family protein
Cit.10093.1.S1_at	148.1062	382.1468	-2.58022	AT3G18830.1	9.00E-90	ATPLT5 (POLYOL TRANSPORTER 5); D-ribose transmemb
Cit.30656.1.S1_s_at	68.67345	177.273	-2.58139	AT3G19990.1	0	unknown protein
Cit.20509.1.S1_at	21.75128	56.16264	-2.58204		NA	
Cit.1039.1.S1_at	320.5512	828.5773	-2.58485	AT3G03870.2	1.00E-37	unknown protein
Cit.7094.1.S1_at	46.52516	120.5264	-2.59056	AT3G51580.1	7.00E-31	unknown protein
Cit.28396.1.S1_at	69.878	181.3355	-2.59503		NA	
Cit.18720.1.S1_s_at	32.06803	83.26312	-2.59645		NA	
Cit.30200.1.S1_at	22.13606	57.47722	-2.59654	AT5G60870.1	5.00E-45	regulator of chromosome condensation (RCC1) family pr
Cit.10686.1.S1_at	509.045	1322.505	-2.59801	AT1G60420.1	8.00E-86	DC1 domain-containing protein

Cit.10457.1.S1_at	878.5825	2283.888	-2.59951	AT3G25180.1	6.00E-81	CYP82G1; electron carrier/ heme binding / iron ion bindi
Cit.2131.1.S1_s_at	415.7572	1083.414	-2.60588	AT5G19440.1	1.00E-152	cinnamyl-alcohol dehydrogenase, putative (CAD)
Cit.21736.1.S1_at	157.1397	409.5207	-2.60609	AT3G57790.1	8.00E-33	glycoside hydrolase family 28 protein / polygalacturonas
Cit.15850.1.S1_at	29.73378	77.6731	-2.61228	AT4G33950.1	2.00E-82	OST1 (OPEN STOMATA 1); calcium-dependent protein se
Cit.1422.1.S1_at	63.47057	165.8235	-2.6126	AT5G18670.1	0	BMY3; beta-amylase/ catalytic/ cation binding
Cit.14472.1.S1_s_at	224.6352	586.9031	-2.61269		NA	
Cit.24178.1.S1_at	54.56383	142.7448	-2.61611	AT3G52190.1	2.00E-34	PHF1 (PHOSPHATE TRANSPORTER TRAFFIC FACILITATOR
Cit.20000.1.S1_at	81.49403	213.2457	-2.6167	AT5G07280.1	1.00E-06	EMS1 (EXCESS MICROSPOROCYTES1); kinase/ transmem
Cit.15785.1.S1_at	87.03323	228.2262	-2.62229	AT4G20970.1	2.00E-22	basic helix-loop-helix (bHLH) family protein
Cit.2875.1.S1_at	21.88341	57.4437	-2.62499	AT5G18420.3	3.00E-23	unknown protein
Cit.31963.1.S1_at	46.28778	121.6282	-2.62765	AT4G02050.1	2.00E-52	sugar transporter, putative
Cit.2753.1.S1_s_at	469.0796	1236.143	-2.63525	AT4G35220.1	2.00E-96	cyclase family protein
Cit.32755.1.S1_at	26.36981	69.63096	-2.64056	ATMG00080.1	1.00E-66	encodes a mitochondrial ribosomal protein L16, which is
Cit.21032.1.S1_at	60.98384	161.0453	-2.64079	AT5G04760.1	5.00E-27	myb family transcription factor
Cit.5891.1.S1_at	43.05399	113.8944	-2.64539	AT4G17500.1	3.00E-75	ATERF-1 (ETHYLENE RESPONSIVE ELEMENT BINDING FAC
Cit.11548.1.S1_at	76.08962	201.6915	-2.65071	AT1G20030.2	3.00E-79	pathogenesis-related thaumatin family protein
Cit.22286.1.S1_x_at	1294.732	3434.588	-2.65274	AT5G07090.2	2.00E-10	40S ribosomal protein S4 (RPS4B)
Cit.18366.1.S1_x_at	111.8234	297.1412	-2.65724		NA	
Cit.21891.1.S1_at	43.90647	116.6997	-2.65792	AT1G53530.2	4.00E-22	signal peptidase I family protein
Cit.17264.1.S1_s_at	108.2211	288.4338	-2.66523	AT2G05540.1	4.00E-16	glycine-rich protein
Cit.21925.1.S1_s_at	109.4513	293.2973	-2.67971	AT3G07700.2	2.00E-93	ABC1 family protein
Cit.29391.1.S1_s_at	114.5968	307.5857	-2.68407	AT1G59950.1	3.00E-99	aldo/keto reductase, putative
Cit.7775.1.S1_at	46.29592	124.5537	-2.69038	AT1G17010.1	8.00E-11	oxidoreductase, 2OG-Fe(II) oxygenase family protein
Cit.4682.1.S1_at	165.0141	444.3923	-2.69306	AT2G31880.1	0	leucine-rich repeat transmembrane protein kinase, puta
Cit.22451.1.S1_x_at	29.18847	78.82303	-2.70049	AT2G45550.1	6.00E-60	CYP76C4; electron carrier/ heme binding / iron ion bindi
Cit.17346.1.S1_at	111.0778	300.7407	-2.70748	AT1G31335.1	2.00E-06	unknown protein
Cit.5269.1.S1_at	25.37598	68.83205	-2.71249		NA	
Cit.13689.1.S1_s_at	210.0242	570.0424	-2.71417	AT5G26731.1	3.00E-17	unknown protein
Cit.30894.1.S1_at	25.1181	68.39654	-2.723	AT3G15290.1	1.00E-107	3-hydroxybutyryl-CoA dehydrogenase, putative
Cit.30506.1.S1_s_at	398.605	1087.508	-2.72828	AT5G01600.1	3.00E-92	ATFER1; ferric iron binding / iron ion binding
Cit.39931.1.S1_at	24.50419	66.90794	-2.73047	AT3G22060.1	2.00E-36	receptor protein kinase-related
Cit.12234.1.S1_at	329.536	900.9593	-2.73402	AT2G32540.1	3.00E-96	ATCSLB04; cellulose synthase/ transferase/ transferase,
Cit.18704.1.S1_at	26.78438	73.23631	-2.73429		NA	
Cit.3795.1.S1_at	311.0387	854.7733	-2.74813	AT2G24580.1	1.00E-90	sarcosine oxidase family protein
Cit.12843.1.S1_at	45.91303	126.5389	-2.75606		NA	

Cit.15944.1.S1_x_at	38.30923	105.7062	-2.75929	AT1G17860.1	5.00E-09	trypsin and protease inhibitor family protein / Kunitz fan
Cit.3549.1.S1_at	223.9631	620.6043	-2.77101	AT3G12360.1	9.00E-55	ITN1 (INCREASED TOLERANCE TO NACL); protein binding
Cit.16700.1.S1_at	205.8852	572.0358	-2.77842	AT3G07700.2	2.00E-93	ABC1 family protein
Cit.28815.1.S1_x_at	54.32609	151.3052	-2.78513		NA	
Cit.14998.1.S1_at	96.65543	269.3124	-2.78631	AT4G03500.1	1.00E-33	ankyrin repeat family protein
Cit.8519.1.S1_x_at	39.79035	111.8119	-2.81003	AT2G38380.1	1.00E-109	peroxidase 22 (PER22) (P22) (PRXEA) / basic peroxidase I
Cit.28173.1.S1_s_at	69.83521	196.5533	-2.81453	AT4G21380.1	1.00E-106	ARK3 (A. THALIANA RECEPTOR KINASE 3); kinase/ transm
Cit.12388.1.S1_at	42.20545	119.0503	-2.82073	AT2G40095.1	9.00E-66	unknown protein
Cit.5493.1.S1_s_at	207.6788	586.4824	-2.82399	AT2G39705.1	2.00E-14	RTFL8 (ROTUNDIFOLIA LIKE 8)
Cit.19672.1.S1_at	58.28079	164.7134	-2.8262		NA	
Cit.23218.1.S1_at	25.45604	72.06213	-2.83085		NA	
Cit.14133.1.S1_s_at	49.68515	141.1836	-2.84157	AT5G15120.1	6.00E-61	unknown protein
Cit.35594.1.S1_at	55.8647	159.112	-2.84817	AT3G07870.1	9.00E-28	F-box family protein
Cit.2350.1.S1_at	70.29469	200.4682	-2.85183	AT2G38470.1	2.00E-42	WRKY33; transcription factor
Cit.17675.1.S1_at	73.94147	211.035	-2.85408	AT1G26250.1	2.00E-29	proline-rich extensin, putative
Cit.9384.1.S1_x_at	137.7642	393.351	-2.85525	AT5G61820.1	1.00E-147	FUNCTIONS IN: molecular_function unknown; INVOLVED
Cit.18460.1.S1_at	40.1973	114.8784	-2.85786		NA	
Cit.7334.1.S1_at	176.0984	503.5093	-2.85925	AT5G17350.1	7.00E-37	unknown protein
Cit.29929.1.S1_at	23.14612	66.19469	-2.85986	AT5G67370.1	1.00E-77	unknown protein
Cit.858.1.S1_s_at	411.7225	1178.872	-2.86327	AT1G71695.1	1.00E-118	peroxidase 12 (PER12) (P12) (PRXR6)
Cit.12140.1.S1_at	520.5223	1494.93	-2.87198	AT3G08860.1	3.00E-98	alanine--glyoxylate aminotransferase, putative / beta-ala
Cit.38996.1.S1_s_at	161.4981	464.4279	-2.87575	AT3G03870.2	1.00E-37	unknown protein
Cit.3817.1.S1_at	140.0354	403.2786	-2.87983	AT4G22920.1	1.00E-99	NYE1 (NON-YELLOWING 1)
Cit.38990.1.S1_at	41.9507	121.4321	-2.89464	AT1G21000.2	1.00E-100	zinc-binding family protein
Cit.11985.1.S1_at	84.65129	245.9573	-2.90554	AT5G52300.2	2.00E-66	LTI65 (LOW-TEMPERATURE-INDUCED 65)
Cit.29386.1.S1_at	253.3622	736.2572	-2.90595	AT3G44735.1	2.00E-11	PSK1; growth factor
Cit.31079.1.S1_at	99.08822	288.9807	-2.9164	AT4G37925.1	9.00E-41	NDH-M (subunit NDH-M of NAD(P)H:plastoquinone deh
Cit.7634.1.S1_at	101.0486	294.7316	-2.91673		NA	
Cit.21321.1.S1_x_at	2865.083	8359.929	-2.91787	AT3G09390.1	4.00E-23	MT2A (METALLOTHIONEIN 2A); copper ion binding
Cit.9384.1.S1_s_at	3068.79	8957.928	-2.91904	AT5G61820.1	1.00E-147	FUNCTIONS IN: molecular_function unknown; INVOLVED
Cit.10594.1.S1_at	66.06531	192.9299	-2.92029	AT3G54420.1	3.00E-66	ATEP3; chitinase
Cit.4453.1.S1_at	915.7973	2681.238	-2.92776	AT2G22660.2	3.00E-19	FUNCTIONS IN: molecular_function unknown; INVOLVED
Cit.603.1.S1_x_at	60.44295	177.4986	-2.93663	AT1G47128.1	1.00E-128	RD21 (responsive to dehydration 21); cysteine-type endo
Cit.14161.1.S1_at	65.89198	193.8284	-2.94161	AT1G20560.2	1.00E-122	AAE1 (ACYL ACTIVATING ENZYME 1); AMP binding
Cit.4977.1.S1_s_at	333.6031	982.6369	-2.94553	AT4G31940.1	3.00E-77	CYP82C4; electron carrier/ heme binding / iron ion bindi

Cit.374.1.S1_s_at	317.4726	936.9636	-2.95132	AT1G69530.2	1.00E-123	ATEXPA1 (ARABIDOPSIS THALIANA EXPANSIN A1)
Cit.31351.1.S1_at	52.72	156.7526	-2.9733	AT5G01750.2	1.00E-36	unknown protein
Cit.6258.1.S1_x_at	31.73522	94.45496	-2.97634		NA	
Cit.21331.1.S1_x_at	33.13772	98.718	-2.97902		NA	
Cit.26572.1.S1_s_at	47.4228	141.3386	-2.98039	AT5G42930.1	2.00E-44	triacylglycerol lipase
Cit.20865.1.S1_at	233.2183	695.1389	-2.98064	AT1G32170.1	2.00E-46	XTR4 (XYLOGLUCAN ENDOTRANSGLYCOSYLASE 4); hydrc
Cit.15009.1.S1_at	84.20674	251.4734	-2.98638	AT4G10120.2	1.00E-160	ATSPS4F; transferase, transferring glycosyl groups
Cit.5492.1.S1_at	50.38663	150.6734	-2.99034	AT2G39705.1	5.00E-11	RTFL8 (ROTUNDIFOLIA LIKE 8)
Cit.15018.1.S1_at	238.4017	715.9567	-3.00315	AT4G10270.1	2.00E-22	wound-responsive family protein
Cit.8595.1.S1_s_at	2173.238	6561.104	-3.01905	AT4G32940.1	0	GAMMA-VPE (GAMMA VACUOLAR PROCESSING ENZYM
Cit.20743.1.S1_at	198.1127	598.9373	-3.02322	AT5G65500.1	3.00E-34	ATP binding / kinase/ protein kinase/ protein serine/thre
Cit.29438.1.S1_s_at	59.89452	181.513	-3.03054	AT1G65480.1	5.00E-76	FT (FLOWERING LOCUS T); phosphatidylethanolamine bi
Cit.18435.1.S1_at	75.67184	229.468	-3.03241	AT4G08850.1	7.00E-18	kinase
Cit.13886.1.S1_s_at	495.049	1502.895	-3.03585	AT4G13250.1	2.00E-95	short-chain dehydrogenase/reductase (SDR) family prote
Cit.6015.1.S1_at	49.12645	149.1974	-3.03701	AT2G39450.1	1.00E-165	MTP11; cation transmembrane transporter/ manganese
Cit.9301.1.S1_s_at	86.91537	263.9889	-3.03731	AT5G06570.2	3.00E-47	hydrolase
Cit.5970.1.S1_at	332.7077	1013.912	-3.04746	AT5G61430.1	6.00E-92	ANAC100 (ARABIDOPSIS NAC DOMAIN CONTAINING PRC
Cit.31374.1.S1_at	39.07986	120.112	-3.0735	AT2G45550.1	1.00E-34	CYP76C4; electron carrier/ heme binding / iron ion bindi
Cit.5502.1.S1_at	152.3478	468.368	-3.07433	AT3G07600.1	8.00E-16	heavy-metal-associated domain-containing protein
Cit.3862.1.S1_at	605.3165	1862.301	-3.07657		NA	
Cit.21206.1.S1_x_at	60.07151	185.1287	-3.08181		NA	
Cit.7987.1.S1_x_at	139.8929	433.8099	-3.10101	AT3G09390.1	4.00E-23	MT2A (METALLOTHIONEIN 2A); copper ion binding
Cit.2725.1.S1_s_at	252.2475	782.5446	-3.10229	AT1G08630.4	1.00E-152	THA1 (Threonine Aldolase 1); aldehyde-lyase/ threonine
Cit.5172.1.S1_at	42.44267	132.0271	-3.11072	AT4G28025.1	1.00E-33	unknown protein
Cit.9822.1.S1_s_at	686.363	2137.374	-3.11406	AT2G47140.1	6.00E-41	short-chain dehydrogenase/reductase (SDR) family prote
Cit.21833.1.S1_x_at	130.9686	408.2764	-3.11736	AT5G53130.1	6.00E-08	CNGC1 (CYCLIC NUCLEOTIDE GATED CHANNEL 1); calmo
Cit.31490.1.S1_at	51.45267	160.4589	-3.11857		NA	
Cit.30272.1.S1_at	41.79863	130.4113	-3.11999	AT1G76160.1	1.00E-108	sks5 (SKU5 Similar 5); copper ion binding / oxidoreducta
Cit.35761.1.S1_at	22.3735	69.88407	-3.12352	AT1G69850.1	2.00E-28	ATNRT1:2 (ARABIDOPSIS THALIANA NITRATE TRANSPOR
Cit.12598.1.S1_s_at	37.86568	118.7317	-3.1356	AT2G45550.1	3.00E-80	CYP76C4; electron carrier/ heme binding / iron ion bindi
Cit.2041.1.S1_s_at	80.1288	252.0629	-3.14572	AT3G02630.1	1.00E-118	acyl-(acyl-carrier-protein) desaturase, putative / stearoy
Cit.31285.1.S1_at	242.5826	764.856	-3.15297	AT2G18700.1	7.00E-51	ATTPS11; transferase, transferring glycosyl groups
Cit.7824.1.S1_at	20.51086	65.28133	-3.18277	AT1G09540.1	1.00E-23	MYB61 (MYB DOMAIN PROTEIN 61); DNA binding / trans
Cit.5117.1.S1_at	111.2334	354.4077	-3.18616	AT3G62550.1	1.00E-53	universal stress protein (USP) family protein
Cit.39791.1.S1_s_at	151.0696	481.9876	-3.1905	AT3G14420.2	1.00E-179	(S)-2-hydroxy-acid oxidase, peroxisomal, putative / glycc

Cit.1497.1.S1_s_at	685.9099	2205.806	-3.21588	AT3G04070.1	1.00E-98	anac047 (Arabidopsis NAC domain containing protein 47
Cit.39542.1.S1_at	20.85655	67.34684	-3.22905	AT5G11430.1	1.00E-12	zinc ion binding
Cit.16807.1.S1_at	276.9954	900.6693	-3.25157	AT3G63010.1	9.00E-66	GID1B (GA INSENSITIVE DWARF1B); hydrolase
Cit.19461.1.S1_s_at	1503.076	4888.479	-3.25232	AT3G44735.1	9.00E-12	PSK1; growth factor
Cit.8930.1.S1_s_at	45.0748	147.2025	-3.26574	AT5G54160.1	4.00E-96	ATOMT1 (O-METHYLTRANSFERASE 1); caffeate O-methy
Cit.35499.1.S1_at	71.63476	235.3246	-3.28506	AT4G22620.1	5.00E-44	auxin-responsive family protein
Cit.4666.1.S1_at	31.63283	104.1466	-3.29236	AT2G27920.1	0	SCPL51 (SERINE CARBOXYPEPTIDASE-LIKE 51); serine-typ
Cit.12647.1.S1_at	132.7885	438.1456	-3.29957	AT3G13530.1	1.00E-121	MAPKKK7; ATP binding / binding / kinase/ protein kinase
Cit.14337.1.S1_at	22.09866	73.02995	-3.30472	AT5G48930.1	6.00E-41	HCT (HYDROXYCINNAMOYL-COA SHIKIMATE/QUINATE H
Cit.8515.1.S1_x_at	27.17843	89.84012	-3.30557	AT5G06730.1	1.00E-105	peroxidase, putative
Cit.8933.1.S1_s_at	22.56957	74.69563	-3.30957	AT5G54160.1	1.00E-95	ATOMT1 (O-METHYLTRANSFERASE 1); caffeate O-methy
Cit.30596.1.S1_at	48.27892	162.253	-3.36074	AT3G19615.1	3.00E-13	unknown protein
Cit.3746.1.S1_s_at	81.66128	278.6868	-3.41272	AT5G17540.1	1.00E-114	transferase family protein
Cit.9722.1.S1_x_at	24.82526	85.23959	-3.43358	AT4G20260.3	5.00E-41	DREPP plasma membrane polypeptide family protein
Cit.7966.1.S1_x_at	1046.48	3593.836	-3.43421	AT1G17860.1	2.00E-31	trypsin and protease inhibitor family protein / Kunitz fan
Cit.17413.1.S1_s_at	151.2316	526.9858	-3.48463	AT4G33467.2	1.00E-09	unknown protein
Cit.9029.1.S1_x_at	37.16271	131.3051	-3.53325	AT2G32210.1	3.00E-22	unknown protein
Cit.18299.1.S1_at	28.24131	100.9663	-3.57513	AT2G23270.1	1.00E-06	unknown protein
Cit.22649.1.S1_x_at	60.4825	216.5181	-3.57985	AT4G25150.1	6.00E-50	acid phosphatase, putative
Cit.11156.1.S1_s_at	32.9816	118.6516	-3.59751	AT4G22070.1	1.00E-149	WRKY31; transcription factor
Cit.6320.1.S1_at	203.0195	732.2994	-3.60704	AT1G01320.1	1.00E-17	tetratricopeptide repeat (TPR)-containing protein
Cit.19686.1.S1_s_at	28.97522	104.8156	-3.61742	AT1G65480.1	5.00E-76	FT (FLOWERING LOCUS T); phosphatidylethanolamine bi
Cit.372.1.S1_s_at	72.75961	263.2065	-3.61748	AT5G26600.1	1.00E-176	catalytic/ pyridoxal phosphate binding
Cit.31262.1.S1_at	63.19451	228.8728	-3.62172		NA	
Cit.9421.1.S1_s_at	47.3678	172.2975	-3.63744	AT4G25810.1	1.00E-129	XTR6 (XYLOGLUCAN ENDOTRANSGLYCOSYLASE 6); hydrc
Cit.14918.1.S1_at	62.1372	226.6259	-3.64719	AT1G03220.1	1.00E-113	extracellular dermal glycoprotein, putative / EDGP, puta
Cit.31369.1.S1_at	22.25269	81.47224	-3.66123	AT1G11330.1	2.00E-70	S-locus lectin protein kinase family protein
Cit.9383.1.S1_at	143.0535	528.9217	-3.69737	AT5G61820.1	7.00E-31	FUNCTIONS IN: molecular_function unknown; INVOLVED
Cit.5899.1.S1_at	31.08438	115.3352	-3.71039	AT3G10910.1	4.00E-48	zinc finger (C3HC4-type RING finger) family protein
Cit.25191.1.S1_s_at	650.6637	2414.522	-3.71086	AT5G10770.1	1.00E-160	chloroplast nucleoid DNA-binding protein, putative
Cit.5477.1.S1_s_at	67.29745	251.2045	-3.73275	AT1G31130.1	2.00E-36	unknown protein
Cit.26654.1.S1_x_at	48.475	181.5523	-3.74528		NA	
Cit.9300.1.S1_s_at	117.8407	442.4232	-3.75442	AT3G28860.1	7.00E-92	ABCB19; ATPase, coupled to transmembrane movement
Cit.3500.1.S1_s_at	34.76445	131.0667	-3.77014	AT5G04500.1	1.00E-05	glycosyltransferase family protein 47
Cit.1007.1.S1_s_at	31.64558	120.041	-3.79329	AT5G25560.1	1.00E-134	zinc finger (C3HC4-type RING finger) family protein

Cit.20780.1.S1_at	60.41481	229.9862	-3.80679		NA	
Cit.20194.1.S1_at	49.90477	191.4249	-3.8358		NA	
Cit.18228.1.S1_at	178.278	692.5502	-3.88466	AT5G13800.1	3.00E-27	hydrolase, alpha/beta fold family protein
Cit.9386.1.S1_x_at	158.7681	617.2922	-3.88801	AT5G61820.1	3.00E-54	FUNCTIONS IN: molecular_function unknown; INVOLVEC
Cit.2730.1.S1_at	146.0295	568.9135	-3.89588	AT5G58350.1	0	WNK4 (WITH NO K (=LYSINE) 4); kinase/ protein kinase
Cit.15258.1.S1_at	54.64937	213.4278	-3.9054	AT5G03795.1	1.00E-120	LOCATED IN: membrane; EXPRESSED IN: embryo, sepal,
Cit.24963.1.S1_at	84.41702	329.6833	-3.90541	AT4G01870.1	5.00E-33	tolB protein-related
Cit.24963.1.S1_s_at	257.936	1016.398	-3.9405	AT4G01870.1	1.00E-115	tolB protein-related
Cit.12269.1.S1_at	20.63501	81.65765	-3.95724	AT2G27230.2	2.00E-48	LHW (LONESOME HIGHWAY); protein homodimerization
Cit.12285.1.S1_x_at	25.09273	100.5967	-4.009	AT1G53310.3	1.00E-88	ATPPC1 (PHOSPHOENOLPYRUVATE CARBOXYLASE 1); ca
Cit.478.1.S1_at	450.2279	1815.915	-4.03332	AT2G23250.1	1.00E-109	UGT84B2 (UDP-glucosyl transferase 84B2); UDP-glycosyl
Cit.13424.1.S1_at	21.52558	87.48406	-4.06419	AT4G19230.1	0	CYP707A1; (+)-abscisic acid 8'-hydroxylase/ oxygen bindi
Cit.6308.1.S1_at	143.2743	585.0632	-4.08352	AT2G29420.1	9.00E-60	ATGSTU7 (ARABIDOPSIS THALIANA GLUTATHIONE S-TRA
Cit.22984.1.S1_x_at	79.96287	326.6179	-4.08462	AT5G05320.1	6.00E-20	monooxygenase, putative (MO3)
Cit.10927.1.S1_s_at	659.323	2706.299	-4.10466	AT3G11660.1	2.00E-81	NHL1
Cit.24670.1.S1_x_at	29.61493	122.1525	-4.12469	AT4G34050.2	3.00E-25	caffeoyl-CoA 3-O-methyltransferase, putative
Cit.34286.1.S1_at	120.2763	497.6834	-4.13783		NA	
Cit.13667.1.S1_s_at	34.49958	144.187	-4.17938	AT4G05070.1	3.00E-09	unknown protein
Cit.13787.1.S1_s_at	52.44014	219.6181	-4.18798	AT2G03200.1	1.00E-50	aspartyl protease family protein
Cit.22666.1.S1_at	54.20988	227.3556	-4.19399	AT1G78960.1	4.00E-66	ATLUP2; beta-amyrin synthase/ lupeol synthase
Cit.21787.1.S1_at	58.08252	243.771	-4.19698	AT3G28580.1	6.00E-46	AAA-type ATPase family protein
Cit.21497.1.S1_at	73.66477	309.5904	-4.20269	AT2G38905.1	5.00E-10	hydrophobic protein, putative / low temperature and sa
Cit.2130.1.S1_x_at	565.816	2390.436	-4.22476	AT5G19440.1	1.00E-151	cinnamyl-alcohol dehydrogenase, putative (CAD)
Cit.20662.1.S1_at	407.7292	1724.25	-4.22891	AT2G32540.1	8.00E-20	ATCSLB04; cellulose synthase/ transferase/ transferase,
Cit.18008.1.S1_at	120.7034	513.8355	-4.25701		NA	
Cit.14999.1.S1_s_at	136.9592	583.0486	-4.2571	AT4G03500.1	1.00E-33	ankyrin repeat family protein
Cit.15242.1.S1_at	55.98788	238.7692	-4.26466	AT3G54420.1	2.00E-74	ATEP3; chitinase
Cit.21150.1.S1_at	20.15273	86.91425	-4.31278		NA	
Cit.6164.1.S1_at	22.07895	95.36626	-4.31933		NA	
Cit.32004.1.S1_at	110.0863	475.6314	-4.32053		NA	
Cit.18482.1.S1_s_at	42.81096	185.2353	-4.32682	AT3G22240.1	7.00E-12	unknown protein
Cit.12817.1.S1_at	40.1433	174.8996	-4.35688	AT5G48930.1	7.00E-45	HCT (HYDROXYCINNAMOYL-COA SHIKIMATE/QUINATE H
Cit.6231.1.S1_s_at	41.29387	182.5851	-4.4216	AT4G23180.1	6.00E-77	CRK10 (CYSTEINE-RICH RLK10); ATP binding / kinase/ prc
Cit.11340.1.S1_s_at	399.202	1773.504	-4.44262	AT5G04080.1	9.00E-15	unknown protein
Cit.2691.1.S1_at	60.58678	270.9785	-4.47257	AT4G20860.1	2.00E-42	FAD-binding domain-containing protein

Cit.3880.1.S1_at	101.6698	461.7432	-4.5416	AT3G62730.1	5.00E-93	unknown protein
Cit.15866.1.S1_at	139.2202	632.3668	-4.54221	AT3G47780.1	4.00E-23	ATATH6; ATPase, coupled to transmembrane movement
Cit.13680.1.S1_at	343.8971	1592.081	-4.62953	AT5G54390.1	1.00E-137	AHL (ARABIDOPSIS HAL2-LIKE); 3'(2'),5'-bisphosphate nu
Cit.8769.1.S1_x_at	38.72335	181.1733	-4.67866	AT4G16190.1	1.00E-159	cysteine proteinase, putative
Cit.18491.1.S1_at	532.677	2538.362	-4.76529		NA	
Cit.13148.1.S1_at	95.49456	461.7985	-4.83586	AT5G18840.1	2.00E-68	sugar transporter, putative
Cit.16993.1.S1_s_at	62.06757	300.5999	-4.84311	AT3G55240.1	4.00E-36	Overexpression leads to PEL (Pseudo-Etiolation in Light)
Cit.21833.1.S1_at	100.7168	491.6637	-4.88165	AT5G53130.1	6.00E-08	CNGC1 (CYCLIC NUCLEOTIDE GATED CHANNEL 1); calmo
Cit.13816.1.S1_s_at	51.60469	255.6373	-4.95376	AT5G06060.1	2.00E-89	tropinone reductase, putative / tropine dehydrogenase,
Cit.11806.1.S1_at	83.43438	413.5187	-4.95621	AT2G31050.1	4.00E-26	plastocyanin-like domain-containing protein
Cit.35871.1.S1_at	33.54356	166.4463	-4.96209	AT4G13440.1	5.00E-16	calcium-binding EF hand family protein
Cit.21842.1.S1_s_at	173.4384	865.1946	-4.98848	AT1G01320.1	1.00E-17	tetratricopeptide repeat (TPR)-containing protein
Cit.17208.1.S1_at	450.7426	2264.167	-5.02319	AT4G25000.1	1.00E-174	AMY1 (ALPHA-AMYLASE-LIKE); alpha-amylase
Cit.30695.1.S1_s_at	383.1352	1937.207	-5.0562	AT3G21420.1	1.00E-151	oxidoreductase, 2OG-Fe(II) oxygenase family protein
Cit.17325.1.S1_at	139.948	708.1812	-5.06032	AT2G18660.1	5.00E-32	EXLB3 (EXPANSIN-LIKE B3 PRECURSOR)
Cit.3036.1.S1_s_at	349.4042	1774.531	-5.07873	AT3G04920.1	9.00E-63	40S ribosomal protein S24 (RPS24A)
Cit.1496.1.S1_s_at	231.6758	1182.297	-5.10324	AT3G04070.1	1.00E-98	anac047 (Arabidopsis NAC domain containing protein 47
Cit.10547.1.S1_s_at	193.1672	986.2004	-5.10542	AT1G44350.1	5.00E-97	ILL6; IAA-amino acid conjugate hydrolase/ metallopeptic
Cit.9089.1.S1_s_at	279.4626	1426.814	-5.10556	AT2G45550.1	1.00E-134	CYP76C4; electron carrier/ heme binding / iron ion bindi
Cit.3665.1.S1_at	113.3223	578.951	-5.10889	AT1G75750.1	2.00E-30	GASA1 (GAST1 PROTEIN HOMOLOG 1)
Cit.8931.1.S1_at	23.75474	121.6787	-5.12229	AT5G54160.1	4.00E-96	ATOMT1 (O-METHYLTRANSFERASE 1); caffeate O-methy
Cit.16929.1.S1_x_at	135.7772	697.8986	-5.14003		NA	
Cit.14158.1.S1_at	20.99941	108.6849	-5.17562	AT1G61560.1	1.00E-113	MLO6 (MILDEW RESISTANCE LOCUS O 6); calmodulin bir
Cit.20005.1.S1_at	83.34885	431.8366	-5.18107		NA	
Cit.6376.1.S1_at	41.77021	218.2212	-5.22433	AT1G68320.1	2.00E-78	MYB62 (myb domain protein 62); DNA binding / transcri
Cit.1621.1.S1_at	41.40527	216.8584	-5.23746	AT1G64660.1	0	ATMGL (ARABIDOPSIS THALIANA METHIONINE GAMMA-
Cit.30511.1.S1_s_at	369.712	1949.519	-5.27307	AT5G61820.1	1.00E-147	FUNCTIONS IN: molecular_function unknown; INVOLVED
Cit.22319.1.S1_s_at	92.83633	493.6425	-5.31734	AT1G55850.1	3.00E-66	ATCSLE1; cellulose synthase/ transferase, transferring gl
Cit.17258.1.S1_at	60.39408	321.3585	-5.32103	AT1G12810.1	4.00E-21	proline-rich family protein
Cit.15760.1.S1_at	56.18095	299.6274	-5.33326	AT4G01070.1	6.00E-90	GT72B1; UDP-glucosyltransferase/ UDP-glycosyltransfera
Cit.9029.1.S1_s_at	111.0941	599.5426	-5.39671	AT2G32210.1	1.00E-23	unknown protein
Cit.27205.1.S1_at	28.44005	156.0787	-5.48799	AT5G57560.1	4.00E-74	TCH4 (Touch 4); hydrolase, acting on glycosyl bonds / xyl
Cit.10311.1.S1_s_at	55.17162	303.6065	-5.50295	AT1G21890.1	1.00E-135	nodulin MtN21 family protein
Cit.21160.1.S1_s_at	88.39069	486.5332	-5.50435	AT5G13200.1	1.00E-90	GRAM domain-containing protein / ABA-responsive prot
Cit.2926.1.S1_at	40.76267	225.4745	-5.5314	AT5G23810.1	6.00E-62	AAP7; amino acid transmembrane transporter

Cit.9025.1.S1_x_at	289.1347	1601.708	-5.53966	AT1G03400.1	1.00E-95	2-oxoglutarate-dependent dioxygenase, putative
Cit.38.1.S1_x_at	117.5093	663.3699	-5.64525		NA	
Cit.12810.1.S1_at	138.3186	782.7784	-5.65924	AT1G19670.1	5.00E-65	ATCLH1 (ARABIDOPSIS THALIANA CORONATINE-INDUCE
Cit.7295.1.S1_at	81.15619	462.3782	-5.69739	AT5G59080.1	3.00E-26	unknown protein
Cit.21825.1.S1_at	62.23929	355.9669	-5.71933	AT1G64380.1	8.00E-13	AP2 domain-containing transcription factor, putative
Cit.16490.1.S1_at	27.50389	157.9195	-5.74172	AT1G11190.1	1.00E-130	BFN1 (BIFUNCTIONAL NUCLEASE I); T/G mismatch-speci
Cit.7343.1.S1_at	99.87006	573.5068	-5.74253	AT5G01830.1	1.00E-131	armadillo/beta-catenin repeat family protein / U-box do
Cit.31144.1.S1_at	28.7547	165.2154	-5.74568	AT1G10070.3	1.00E-119	ATBCAT-2 (ARABIDOPSIS THALIANA BRANCHED-CHAIN A
Cit.32400.1.S1_at	41.34178	237.8683	-5.7537		NA	
Cit.28253.1.S1_at	51.40811	296.2939	-5.76356	AT3G26220.1	9.00E-54	CYP71B3; electron carrier/ heme binding / iron ion bindi
Cit.5377.1.S1_at	141.8769	819.4627	-5.77587	AT4G37370.1	1.00E-156	CYP81D8; electron carrier/ heme binding / iron ion bindi
Cit.12189.1.S1_s_at	37.81094	218.5032	-5.77884	AT2G35940.3	2.00E-10	BLH1 (BEL1-LIKE HOMEODOMAIN 1); DNA binding / prot
Cit.5936.1.S1_at	61.23833	354.3157	-5.78585	AT4G30470.1	1.00E-123	cinnamoyl-CoA reductase-related
Cit.14020.1.S1_at	205.3929	1203.315	-5.8586	AT5G53970.1	1.00E-180	aminotransferase, putative
Cit.16956.1.S1_x_at	29.96847	177.9592	-5.93821	AT3G15353.1	1.00E-13	MT3 (METALLOTHIONEIN 3); copper ion binding
Cit.15137.1.S1_at	66.2832	403.7271	-6.09094	AT2G41380.1	1.00E-68	embryo-abundant protein-related
Cit.13579.1.S1_s_at	265.648	1626.488	-6.12272	AT5G65660.1	3.00E-48	hydroxyproline-rich glycoprotein family protein
Cit.22861.1.S1_at	36.6042	225.0804	-6.14903	AT5G04760.1	1.00E-32	myb family transcription factor
Cit.21908.1.S1_x_at	71.43414	441.1541	-6.17568		NA	
Cit.12510.1.S1_at	191.0917	1181.327	-6.18199	AT4G01870.1	1.00E-115	tolB protein-related
Cit.17090.1.S1_s_at	100.0322	619.0166	-6.18817	AT1G69490.1	4.00E-89	NAP (NAC-like, activated by AP3/PI); transcription factor
Cit.17187.1.S1_at	36.36605	227.4856	-6.25544	AT1G05575.1	4.00E-17	unknown protein
Cit.21233.1.S1_at	35.8554	226.5473	-6.31836	AT2G31090.1	1.00E-30	unknown protein
Cit.22710.1.S1_x_at	240.5636	1523.489	-6.333	AT4G11650.1	9.00E-25	ATOSM34 (osmotin 34)
Cit.20662.1.S1_s_at	138.196	886.4492	-6.41443	AT2G32540.1	4.00E-57	ATCSLB04; cellulose synthase/ transferase/ transferase,
Cit.15355.1.S1_at	51.34017	330.6654	-6.44068	AT4G10490.1	5.00E-80	oxidoreductase, 2OG-Fe(II) oxygenase family protein
Cit.25942.1.S1_s_at	93.82063	608.5457	-6.48627	AT5G06320.1	2.00E-66	NHL3
Cit.13166.1.S1_at	49.64692	327.6411	-6.59942	AT5G13200.1	1.00E-90	GRAM domain-containing protein / ABA-responsive prot
Cit.17018.1.S1_s_at	355.8999	2372.798	-6.66704	AT1G23040.1	3.00E-31	hydroxyproline-rich glycoprotein family protein
Cit.21654.1.S1_s_at	20.51941	137.1296	-6.68292	AT3G54420.1	3.00E-66	ATEP3; chitinase
Cit.16919.1.S1_s_at	150.6367	1033.715	-6.86231	AT3G51630.1	1.00E-105	WNK5 (WITH NO LYSINE (K) KINASE 5); protein kinase
Cit.12090.1.S1_at	237.9969	1638.891	-6.88619		NA	
Cit.11086.1.S1_at	379.843	2620.132	-6.89793	AT1G02070.1	2.00E-13	unknown protein
Cit.30583.1.S1_at	45.41187	318.2118	-7.00724	AT4G33865.1	7.00E-27	40S ribosomal protein S29 (RPS29C)
Cit.17451.1.S1_x_at	306.8778	2162.766	-7.04765		NA	

Cit.24483.1.S1_s_at	350.1738	2468.705	-7.04994	AT4G37870.1	1.00E-164	PCK1 (PHOSPHOENOLPYRUVATE CARBOXYKINASE 1); AT
Cit.10569.1.S1_s_at	141.9352	1001.263	-7.05437	AT4G12390.1	3.00E-59	PME1 (PECTIN METHYLESTERASE INHIBITOR 1); enzyme
Cit.11918.1.S1_x_at	313.4026	2218.066	-7.07737	AT4G38540.1	2.00E-78	monooxygenase, putative (MO2)
Cit.157.1.S1_x_at	421.7865	3040.037	-7.20753		NA	
Cit.21825.1.S1_s_at	100.09	723.2665	-7.22616	AT1G64380.1	8.00E-13	AP2 domain-containing transcription factor, putative
Cit.29626.1.S1_s_at	29.81787	217.2673	-7.28648	AT5G50260.1	1.00E-158	cysteine proteinase, putative
Cit.9388.1.S1_at	109.8367	802.8576	-7.30956	AT5G61820.1	7.00E-24	FUNCTIONS IN: molecular_function unknown; INVOLVED
Cit.2927.1.S1_s_at	337.6413	2489.11	-7.37205	AT5G23810.1	2.00E-96	AAP7; amino acid transmembrane transporter
Cit.753.1.S1_x_at	560.1631	4130.308	-7.3734	AT3G04720.1	1.00E-46	PR4 (PATHOGENESIS-RELATED 4); chitin binding
Cit.2928.1.S1_s_at	33.02243	246.1447	-7.45386	AT5G23810.1	1.00E-103	AAP7; amino acid transmembrane transporter
Cit.21295.1.S1_at	58.22176	434.4563	-7.46209		NA	
Cit.17840.1.S1_s_at	73.76325	550.8292	-7.46753	AT1G02070.1	2.00E-13	unknown protein
Cit.25118.1.S1_at	41.07017	309.0967	-7.52606	AT5G48930.1	8.00E-33	HCT (HYDROXYCINNAMOYL-COA SHIKIMATE/QUINATE H
Cit.580.1.S1_x_at	232.9208	1754.496	-7.53259	AT3G04720.1	9.00E-47	PR4 (PATHOGENESIS-RELATED 4); chitin binding
Cit.3086.1.S1_at	81.32067	613.4789	-7.54395	AT1G68320.1	2.00E-63	MYB62 (myb domain protein 62); DNA binding / transcri
Cit.7553.1.S1_at	61.91616	480.6893	-7.76355	AT2G01300.1	5.00E-19	unknown protein
Cit.20640.1.S1_at	22.9958	181.0282	-7.87223	AT1G64380.1	2.00E-08	AP2 domain-containing transcription factor, putative
Cit.9703.1.S1_at	366.9583	2950.066	-8.03924	AT3G57270.1	1.00E-113	BG1 (BETA-1,3-GLUCANASE 1); catalytic/ cation binding /
Cit.4078.1.S1_at	133.3265	1074.185	-8.0568	AT5G50260.1	1.00E-158	cysteine proteinase, putative
Cit.21810.1.S1_x_at	123.2623	1027.805	-8.33836		NA	
Cit.29361.1.S1_s_at	125.5193	1047.204	-8.34297		NA	
Cit.9388.1.S1_x_at	104.3247	890.4498	-8.53537	AT5G61820.1	7.00E-24	FUNCTIONS IN: molecular_function unknown; INVOLVED
Cit.11079.1.S1_at	563.8634	4860.398	-8.61981	AT4G26140.1	0	BGAL12 (beta-galactosidase 12); beta-galactosidase/ cat
Cit.3002.1.S1_s_at	116.3155	1006.703	-8.65493	AT4G12320.1	1.00E-135	CYP706A6; electron carrier/ heme binding / iron ion binc
Cit.12749.1.S1_at	20.84109	181.9916	-8.73235	AT1G47480.1	2.00E-63	hydrolase
Cit.8501.1.S1_at	36.13062	334.992	-9.27169		NA	
Cit.26446.1.S1_at	35.10851	330.9354	-9.42607	AT2G36790.1	9.00E-79	UGT73C6 (UDP-glucosyl transferase 73C6); UDP-glucosyl
Cit.17178.1.S1_x_at	43.33037	422.3674	-9.74761		NA	
Cit.26455.1.S1_at	30.77388	306.0757	-9.94596	AT5G67400.1	1.00E-60	peroxidase 73 (PER73) (P73) (PRXR11)
Cit.11226.1.S1_at	85.5244	860.6757	-10.0635	AT3G26330.1	1.00E-150	CYP71B37; electron carrier/ heme binding / iron ion binc
Cit.26572.1.S1_at	32.72144	331.0298	-10.1166	AT5G42930.1	3.00E-07	triacylglycerol lipase
Cit.2906.1.S1_s_at	44.10034	460.1866	-10.435		NA	
Cit.20225.1.S1_at	51.94172	573.3059	-11.0375	AT1G07750.1	6.00E-14	cupin family protein
Cit.12743.1.S1_at	128.8136	1442.639	-11.1994	AT2G29420.1	9.00E-55	ATGSTU7 (ARABIDOPSIS THALIANA GLUTATHIONE S-TRA
Cit.11918.1.S1_at	190.6935	2225.945	-11.6729	AT4G38540.1	2.00E-78	monooxygenase, putative (MO2)

Cit.3377.1.S1_at	56.25408	663.6782	-11.7979	AT5G20230.1	4.00E-30	ATBCB (ARABIDOPSIS BLUE-COPPER-BINDING PROTEIN);
Cit.20926.1.S1_s_at	62.29515	794.8063	-12.7587	AT4G32375.1	7.00E-07	glycoside hydrolase family 28 protein / polygalacturonas
Cit.13579.1.S1_at	31.99179	421.0559	-13.1614	AT5G65660.1	3.00E-48	hydroxyproline-rich glycoprotein family protein
Cit.6827.1.S1_x_at	57.47481	805.6912	-14.0182	AT1G17010.1	7.00E-81	oxidoreductase, 2OG-Fe(II) oxygenase family protein
Cit.21717.1.S1_at	128.0997	1804.244	-14.0847	AT3G04720.1	2.00E-43	PR4 (PATHOGENESIS-RELATED 4); chitin binding
Cit.3629.1.S1_s_at	31.49201	443.6122	-14.0865	AT1G10070.2	1.00E-156	ATBCAT-2 (ARABIDOPSIS THALIANA BRANCHED-CHAIN A
Cit.1727.1.S1_s_at	116.1067	1677.362	-14.4467	AT3G54420.1	6.00E-76	ATEP3; chitinase
Cit.29688.1.S1_s_at	58.52265	852.7935	-14.572	AT3G61890.1	5.00E-40	ATHB-12 (ARABIDOPSIS THALIANA HOMEOBOX 12); tran
Cit.8515.1.S1_s_at	46.25503	674.554	-14.5834	AT5G06720.1	1.00E-116	peroxidase, putative
Cit.4474.1.S1_s_at	39.65984	580.1862	-14.6291	AT4G15610.1	2.00E-48	integral membrane family protein
Cit.6332.1.S1_at	77.71014	1137.832	-14.642	AT2G34930.1	5.00E-35	disease resistance family protein
Cit.18677.1.S1_s_at	215.8203	3448.672	-15.9794	AT3G54420.1	6.00E-76	ATEP3; chitinase
Cit.2116.1.S1_s_at	147.1974	2404.162	-16.3329	AT4G11650.1	2.00E-87	ATOSM34 (osmotin 34)
Cit.25075.1.S1_s_at	80.44727	1493.762	-18.5682	AT1G18880.1	4.00E-72	proton-dependent oligopeptide transport (POT) family p
Cit.6849.1.S1_at	33.09291	617.6151	-18.6631	AT5G41800.1	1.00E-65	amino acid transporter family protein
Cit.3171.1.S1_x_at	39.55956	789.7639	-19.9639	AT1G18880.1	4.00E-72	proton-dependent oligopeptide transport (POT) family p
Cit.11963.1.S1_at	35.09945	742.7101	-21.1602	AT4G32375.1	7.00E-07	glycoside hydrolase family 28 protein / polygalacturonas
Cit.3171.1.S1_at	29.91173	693.4229	-23.1823	AT1G18880.1	4.00E-72	proton-dependent oligopeptide transport (POT) family p
Cit.5438.1.S1_at	35.60378	840.3107	-23.6017	AT2G25625.2	8.00E-20	unknown protein
Cit.3761.1.S1_x_at	32.09652	795.9073	-24.7973		NA	
Cit.29384.1.S1_x_at	23.12693	575.722	-24.894	AT4G31500.1	1.00E-143	CYP83B1 (CYTOCHROME P450 MONOOXYGENASE 83B1)
Cit.881.1.S1_s_at	28.59974	739.3943	-25.8532	AT3G55240.1	4.00E-36	Overexpression leads to PEL (Pseudo-Etiolation in Light)
Cit.26593.1.S1_at	158.4269	4436.657	-28.0044		NA	
Cit.18008.1.S1_s_at	30.74964	904.1268	-29.4028		NA	
Cit.18037.1.S1_at	25.84605	993.1677	-38.4263	AT3G61510.1	0	ACS1 (ACC SYNTHASE 1); 1-aminocyclopropane-1-carbox
Cit.302.1.S1_s_at	60.3506	3244.373	-53.7588	AT3G12500.1	1.00E-127	ATHCHIB (ARABIDOPSIS THALIANA BASIC CHITINASE); ch
Cit.13606.1.S1_at	22.85389	1359.1	-59.4691	AT5G58390.1	5.00E-83	peroxidase, putative
Cit.1200.1.S1_s_at	41.2424	2528.732	-61.3139	AT4G11650.1	9.00E-81	ATOSM34 (osmotin 34)
Cit.20412.1.S1_s_at	69.12838	5263.507	-76.141	AT3G12500.1	1.00E-127	ATHCHIB (ARABIDOPSIS THALIANA BASIC CHITINASE); ch
Cit.25609.1.S1_s_at	58.01612	4768.814	-82.1981	AT4G35090.1	1.00E-38	CAT2 (CATALASE 2); catalase
Cit.4047.1.S1_at	23.55739	2438.128	-103.497	AT2G02990.1	7.00E-96	RNS1 (RIBONUCLEASE 1); endoribonuclease/ ribonucleas
Cit.2409.1.S1_s_at	26.47206	2746.877	-103.765	AT3G09220.1	0	LAC7 (laccase 7); laccase
Cit.17838.1.S1_at	20.77381	2628.901	-126.549	AT3G27210.1	5.00E-19	unknown protein

Supplementary Table S7. PDTA in seedless vs. seedy pineapple fruits at time point 1.

ProbeSet ID	Pineapple _Seedless	Pineapple _seedy	Ratio	AtGID	E-Score	Arabidopsis.annotation
Cit.25075.1.S1_s_at	298.9879	24.17777	12.36623	AT1G18880.1	4.00E-72	proton-dependent oligopeptide transport (POT) family protein
Cit.2409.1.S1_s_at	2715.32	234.7932	11.56473	AT3G09220.1	0	LAC7 (laccase 7); laccase
Cit.3195.1.S1_at	533.0457	47.32386	11.26378	AT1G33055.1	7.00E-13	unknown protein
Cit.35435.1.S1_x_at	1430.088	130.5198	10.95687	AT5G39110.1	6.00E-79	germin-like protein, putative
Cit.39178.1.S1_s_at	3073.83	284.3394	10.81043	AT5G06760.1	1.00E-48	late embryogenesis abundant group 1 domain-containing protein / l
Cit.10895.1.S1_at	3305.132	332.8976	9.928374	AT5G06760.1	1.00E-48	late embryogenesis abundant group 1 domain-containing protein / l
Cit.31144.1.S1_at	300.6422	30.52791	9.848109	AT1G10070.3	1.00E-119	ATBCAT-2 (ARABIDOPSIS THALIANA BRANCHED-CHAIN AMINO ACID
Cit.29688.1.S1_s_at	775.5304	79.29971	9.779738	AT3G61890.1	5.00E-40	ATHB-12 (ARABIDOPSIS THALIANA HOMEOBOX 12); transcription ac
Cit.17413.1.S1_s_at	380.0366	42.91759	8.855031	AT4G33467.2	1.00E-09	unknown protein
Cit.29507.1.S1_s_at	2374.445	268.6832	8.837341		NA	
Cit.30798.1.S1_at	1963.926	232.5522	8.445097	AT5G05340.1	1.00E-95	peroxidase, putative
Cit.10673.1.S1_at	373.8208	44.49204	8.40197	AT3G26740.1	4.00E-29	CCL (CCR-LIKE)
Cit.3665.1.S1_at	3974.427	478.5793	8.304636	AT1G75750.1	2.00E-30	GASA1 (GAST1 PROTEIN HOMOLOG 1)
Cit.13036.1.S1_at	1852.713	235.6606	7.861785	AT5G55620.1	2.00E-18	unknown protein
Cit.15458.1.S1_at	898.6732	114.6363	7.839342	AT1G18400.1	1.00E-45	BEE1 (BR Enhanced Expression 1); transcription factor
Cit.21654.1.S1_s_at	221.7178	28.88908	7.674796	AT3G54420.1	3.00E-66	ATEP3; chitinase
Cit.18045.1.S1_s_at	802.9995	105.5391	7.60855	AT1G01250.1	3.00E-26	AP2 domain-containing transcription factor, putative
Cit.2630.1.S1_at	1904.162	251.8004	7.562188		NA	
Cit.21911.1.S1_x_at	1130.08	155.0399	7.288962		NA	
Cit.2860.1.S1_at	439.2077	64.2561	6.835269		NA	
Cit.19674.1.S1_s_at	1281.658	187.5658	6.833111	AT3G45140.1	1.00E-109	LOX2 (LIPOXYGENASE 2); lipoxygenase
Cit.21182.1.S1_s_at	736.2467	108.2744	6.799822	AT1G62730.1	1.00E-48	transferase
Cit.580.1.S1_x_at	1894.757	279.0028	6.791176	AT3G04720.1	9.00E-47	PR4 (PATHOGENESIS-RELATED 4); chitin binding
Cit.19182.1.S1_at	199.1194	29.76954	6.688696		NA	
Cit.10661.1.S1_at	2218.466	339.6862	6.530928	AT1G12240.1	0	ATBETAFRUCT4; beta-fructofuranosidase/ hydrolase, hydrolyzing O-
Cit.2399.1.S1_s_at	601.4016	92.67548	6.489328	AT4G11650.1	6.00E-14	ATOSM34 (osmotin 34)
Cit.21182.1.S1_at	310.4046	47.91409	6.478357		NA	
Cit.5970.1.S1_at	1544.35	252.025	6.127765	AT5G61430.1	6.00E-92	ANAC100 (ARABIDOPSIS NAC DOMAIN CONTAINING PROTEIN 100);
Cit.10661.1.S1_s_at	2363.551	399.7015	5.91329	AT1G12240.1	0	ATBETAFRUCT4; beta-fructofuranosidase/ hydrolase, hydrolyzing O-
Cit.5819.1.S1_at	177.2247	30.04997	5.897666	AT2G28900.1	1.00E-48	ATOEP16-1 (OUTER PLASTID ENVELOPE PROTEIN 16-1); P-P-bond-hy

Cit.5438.1.S1_at	355.36	60.79565	5.845155	AT2G25625.2	8.00E-20	unknown protein
Cit.3665.1.S1_s_at	1522.691	261.5571	5.821639	AT1G75750.1	2.00E-30	GASA1 (GAST1 PROTEIN HOMOLOG 1)
Cit.10686.1.S1_at	1371.486	236.8693	5.790054	AT1G60420.1	8.00E-86	DC1 domain-containing protein
Cit.11918.1.S1_at	1644.708	286.2192	5.746323	AT4G38540.1	2.00E-78	monooxygenase, putative (MO2)
Cit.36207.1.S1_at	1193.819	207.8912	5.742518		NA	
Cit.17090.1.S1_s_at	891.8238	155.4756	5.736101	AT1G69490.1	4.00E-89	NAP (NAC-like, activated by AP3/PI); transcription factor
Cit.13606.1.S1_at	2118.059	373.9796	5.663568	AT5G58390.1	5.00E-83	peroxidase, putative
Cit.12810.1.S1_at	184.1318	32.51458	5.663053	AT1G19670.1	5.00E-65	ATCLH1 (ARABIDOPSIS THALIANA CORONATINE-INDUCED PROTEIN
Cit.34286.1.S1_at	551.6613	97.54103	5.655685		NA	
Cit.18564.1.S1_at	534.9413	94.72369	5.647387		NA	
Cit.9569.1.S1_at	528.4749	94.10336	5.615898		NA	
Cit.10672.1.S1_s_at	215.8434	38.53795	5.600801	AT5G07050.1	1.00E-155	LOCATED IN: membrane; CONTAINS InterPro DOMAIN/s: Protein of
Cit.30130.1.S1_at	115.4112	20.75545	5.560525		NA	
Cit.21810.1.S1_x_at	757.1831	136.5006	5.547105		NA	
Cit.20561.1.S1_at	1295.991	234.2579	5.532326		NA	
Cit.14926.1.S1_at	491.2705	90.79098	5.411006	AT2G04520.1	1.00E-73	eukaryotic translation initiation factor 1A, putative / eIF-1A, putativ
Cit.1496.1.S1_s_at	664.8038	123.6285	5.377432	AT3G04070.1	1.00E-98	anac047 (Arabidopsis NAC domain containing protein 47); transcrip
Cit.16807.1.S1_at	718.6112	134.7392	5.333349	AT3G63010.1	9.00E-66	GID1B (GA INSENSITIVE DWARF1B); hydrolase
Cit.38584.1.S1_at	177.6093	33.5743	5.290037		NA	
Cit.8206.1.S1_s_at	375.1398	71.21169	5.267952	AT3G45140.1	1.00E-109	LOX2 (LIPOXYGENASE 2); lipoxygenase
Cit.6827.1.S1_x_at	253.2667	48.39631	5.233182	AT1G17010.1	7.00E-81	oxidoreductase, 2OG-Fe(II) oxygenase family protein
Cit.57.1.S1_at	905.917	173.7559	5.213734	AT1G17860.1	2.00E-27	trypsin and protease inhibitor family protein / Kunitz family protein
Cit.17006.1.S1_s_at	1315.323	252.5063	5.20907	AT3G03870.2	1.00E-37	unknown protein
Cit.9570.1.S1_at	174.3084	33.58996	5.189301	AT5G64260.1	1.00E-125	EXL2 (EXORDIUM LIKE 2)
Cit.6765.1.S1_at	326.763	63.15441	5.174033	AT3G26330.1	9.00E-69	CYP71B37; electron carrier/ heme binding / iron ion binding / mono
Cit.1039.1.S1_at	639.4365	123.6858	5.169846	AT3G03870.2	1.00E-37	unknown protein
Cit.11918.1.S1_x_at	1536.781	298.5256	5.147904	AT4G38540.1	2.00E-78	monooxygenase, putative (MO2)
Cit.15887.1.S1_at	244.3268	47.62099	5.130654	AT3G59940.1	2.00E-50	kelch repeat-containing F-box family protein
Cit.20082.1.S1_x_at	814.2975	159.3367	5.110546	AT1G75750.1	7.00E-33	GASA1 (GAST1 PROTEIN HOMOLOG 1)
Cit.11985.1.S1_at	377.5025	74.15556	5.090684	AT5G52300.2	2.00E-66	LTI65 (LOW-TEMPERATURE-INDUCED 65)
Cit.266.1.S1_at	152.4581	29.98365	5.084708	AT5G47560.1	1.00E-100	TDT (TONOPLAST DICARBOXYLATE TRANSPORTER); malate transme
Cit.60.1.S1_at	165.0066	32.49066	5.078586	AT2G38540.1	1.00E-16	LP1; calmodulin binding
Cit.31342.1.S1_at	255.1447	50.33424	5.069009	AT4G29090.1	8.00E-32	reverse transcriptase, putative / RNA-dependent DNA polymerase,
Cit.26593.1.S1_at	2108.416	425.2286	4.958312		NA	
Cit.25191.1.S1_s_at	432.3693	87.54338	4.938915	AT5G10770.1	1.00E-160	chloroplast nucleoid DNA-binding protein, putative

Cit.13586.1.S1_at	774.7685	157.4641	4.920287	AT3G49940.1	2.00E-52	LBD38 (LOB DOMAIN-CONTAINING PROTEIN 38)
Cit.6376.1.S1_at	460.6694	93.89768	4.906079	AT1G68320.1	2.00E-78	MYB62 (myb domain protein 62); DNA binding / transcription factor
Cit.8697.1.S1_at	3548.229	734.7147	4.829397		NA	
Cit.881.1.S1_s_at	116.0225	24.18114	4.798057	AT3G55240.1	4.00E-36	Overexpression leads to PEL (Pseudo-Etiolation in Light) phenotype.
Cit.18395.1.S1_s_at	1928.332	402.3333	4.792872	AT4G11650.1	6.00E-14	ATOSM34 (osmotin 34)
Cit.22427.1.S1_s_at	489.1325	102.8988	4.75353	AT4G15920.1	1.00E-64	INVOLVED IN: biological_process unknown; LOCATED IN: endomem
Cit.10894.1.S1_s_at	8464.059	1780.699	4.753223	AT5G06760.1	1.00E-48	late embryogenesis abundant group 1 domain-containing protein / l
Cit.9523.1.S1_s_at	2248.442	476.7466	4.71622	AT2G47770.1	6.00E-43	benzodiazepine receptor-related
Cit.31360.1.S1_at	542.509	115.1983	4.709349		NA	
Cit.38996.1.S1_s_at	326.4605	69.7505	4.680404	AT3G03870.2	1.00E-37	unknown protein
Cit.6346.1.S1_at	126.4597	27.21135	4.647314		NA	
Cit.1497.1.S1_s_at	1119.37	241.1723	4.641371	AT3G04070.1	1.00E-98	anac047 (Arabidopsis NAC domain containing protein 47); transcript
Cit.31360.1.S1_s_at	583.4443	125.7652	4.639155		NA	
Cit.57.1.S1_x_at	972.5999	209.8912	4.633829	AT1G17860.1	2.00E-27	trypsin and protease inhibitor family protein / Kunitz family protein
Cit.10152.1.S1_s_at	784.3569	169.4547	4.628711	AT4G27410.2	1.00E-110	RD26 (RESPONSIVE TO DESICCATION 26); transcription activator/ tra
Cit.6076.1.S1_s_at	1086.103	235.5675	4.610581	AT1G22990.1	2.00E-51	heavy-metal-associated domain-containing protein / copper chaper
Cit.39779.1.S1_at	151.7638	32.9598	4.604512	AT4G28530.1	8.00E-73	anac074 (Arabidopsis NAC domain containing protein 74); transcript
Cit.2906.1.S1_s_at	466.6657	103.4069	4.512907		NA	
Cit.13693.1.S1_s_at	183.0446	40.67122	4.500593	AT4G38960.1	6.00E-28	zinc finger (B-box type) family protein
Cit.3036.1.S1_s_at	2685.7	597.5498	4.494521	AT3G04920.1	9.00E-63	40S ribosomal protein S24 (RPS24A)
Cit.3224.1.S1_s_at	926.7795	207.9718	4.456275	AT1G52140.1	2.00E-37	unknown protein
Cit.1320.1.S1_s_at	852.6748	191.4929	4.452775	AT5G65730.1	1.00E-132	xyloglucan:xyloglucosyl transferase, putative / xyloglucan endotrans
Cit.18491.1.S1_at	2423.422	545.1473	4.445444		NA	
Cit.25064.1.S1_s_at	924.2584	208.4184	4.43463	AT1G17860.1	2.00E-27	trypsin and protease inhibitor family protein / Kunitz family protein
Cit.16392.1.S1_at	362.3289	82.81542	4.375138		NA	
Cit.20225.1.S1_at	526.8265	120.4498	4.373826	AT1G07750.1	6.00E-14	cupin family protein
Cit.22463.1.S1_s_at	1186.64	271.7991	4.365872	AT4G27410.2	1.00E-110	RD26 (RESPONSIVE TO DESICCATION 26); transcription activator/ tra
Cit.3629.1.S1_s_at	257.5128	59.05473	4.360579	AT1G10070.2	1.00E-156	ATBCAT-2 (ARABIDOPSIS THALIANA BRANCHED-CHAIN AMINO ACID
Cit.19716.1.S1_x_at	3092.468	714.9509	4.325427		NA	
Cit.1201.1.S1_at	269.0224	62.24728	4.321834	AT4G11650.1	9.00E-81	ATOSM34 (osmotin 34)
Cit.26141.1.S1_s_at	4432.885	1035.696	4.280102		NA	
Cit.29940.1.S1_at	527.2173	123.6756	4.262905	AT5G57050.2	1.00E-119	ABI2 (ABA INSENSITIVE 2); protein serine/threonine phosphatase
Cit.3549.1.S1_at	1092.017	256.5429	4.256664	AT3G12360.1	9.00E-55	ITN1 (INCREASED TOLERANCE TO NACL); protein binding
Cit.10737.1.S1_s_at	4440.763	1045.926	4.245772	AT1G49320.1	3.00E-57	BURP domain-containing protein
Cit.1007.1.S1_s_at	180.1392	42.54552	4.234035	AT5G25560.1	1.00E-134	zinc finger (C3HC4-type RING finger) family protein

Cit.10594.1.S1_at	385.8105	91.31625	4.224993	AT3G54420.1	3.00E-66	ATEP3; chitinase
Cit.17018.1.S1_s_at	975.5438	231.9578	4.205695	AT1G23040.1	3.00E-31	hydroxyproline-rich glycoprotein family protein
Cit.17309.1.S1_at	372.2107	88.63428	4.199399	AT3G03341.1	3.00E-28	unknown protein
Cit.2630.1.S1_a_at	2043.858	489.6246	4.174337		NA	
Cit.31451.1.S1_s_at	548.0762	131.8695	4.156201	AT1G11530.1	5.00E-37	ATCXXS1 (C-terminal cysteine residue is changed to a serine 1); prot
Cit.603.1.S1_x_at	82.5686	20.24736	4.077993	AT1G47128.1	1.00E-128	RD21 (responsive to dehydration 21); cysteine-type endopeptidase/
Cit.8541.1.S1_at	91.62305	22.48817	4.074278	AT4G02380.1	4.00E-20	SAG21 (SENESCENCE-ASSOCIATED GENE 21)
Cit.8903.1.S1_x_at	102.3526	25.1312	4.07273		NA	
Cit.9580.1.S1_at	150.4652	37.12174	4.053291	AT3G55610.1	7.00E-16	P5CS2 (DELTA 1-PYRROLINE-5-CARBOXYLATE SYNTHASE 2); catalytic
Cit.13694.1.S1_at	365.8745	90.29633	4.051931	AT4G38960.1	6.00E-28	zinc finger (B-box type) family protein
Cit.2740.1.S1_at	349.8946	86.35655	4.051744		NA	
Cit.11890.1.S1_s_at	609.001	151.5671	4.018029	AT5G24470.1	4.00E-27	APRR5 (ARABIDOPSIS PSEUDO-RESPONSE REGULATOR 5); transcript
Cit.4721.1.S1_s_at	386.5881	96.42318	4.009286	AT2G36970.1	1.00E-107	UDP-glucoronosyl/UDP-glucosyl transferase family protein
Cit.22219.1.S1_s_at	161.5664	40.31293	4.007806	AT3G45140.1	0	LOX2 (LIPOXYGENASE 2); lipoxygenase
Cit.26455.1.S1_at	173.9261	43.81087	3.96993	AT5G67400.1	1.00E-60	peroxidase 73 (PER73) (P73) (PRXR11)
Cit.14989.1.S1_at	128.2502	32.502	3.945917	AT2G16790.1	6.00E-56	shikimate kinase family protein
Cit.3002.1.S1_s_at	506.7508	128.4316	3.945686	AT4G12320.1	1.00E-135	CYP706A6; electron carrier/ heme binding / iron ion binding / mono
Cit.6849.1.S1_at	173.0685	44.40997	3.897064	AT5G41800.1	1.00E-65	amino acid transporter family protein
Cit.10334.1.S1_at	318.5851	82.36964	3.867749	AT1G37130.1	0	NIA2 (NITRATE REDUCTASE 2); nitrate reductase (NADH)/ nitrate re
Cit.2739.1.S1_at	820.3649	213.8291	3.836545		NA	
Cit.32003.1.S1_at	176.5487	46.1976	3.821599		NA	
Cit.753.1.S1_x_at	3479.9	914.3596	3.805833	AT3G04720.1	1.00E-46	PR4 (PATHOGENESIS-RELATED 4); chitin binding
Cit.4999.1.S1_s_at	519.6502	137.4122	3.781689	AT4G32480.1	4.00E-47	unknown protein
Cit.17929.1.S1_s_at	807.199	214.1683	3.768994		NA	
Cit.30422.1.S1_x_at	333.2881	89.00075	3.744779		NA	
Cit.13750.1.S1_x_at	687.5283	183.9764	3.737046	AT4G17030.1	8.00E-88	ATEXLB1 (ARABIDOPSIS THALIANA EXPANSIN-LIKE B1)
Cit.2860.1.S1_s_at	763.0801	204.8915	3.724313	AT1G33060.2	3.00E-15	no apical meristem (NAM) family protein
Cit.10927.1.S1_s_at	1123.371	302.4687	3.714007	AT3G11660.1	2.00E-81	NHL1
Cit.26052.1.S1_s_at	79.6982	21.7711	3.660734	AT3G55646.1	3.00E-29	unknown protein
Cit.9803.1.S1_s_at	3300.191	905.7326	3.64367		NA	
Cit.21556.1.S1_x_at	2365.049	652.4581	3.624829	AT1G10310.1	6.00E-23	short-chain dehydrogenase/reductase (SDR) family protein
Cit.10033.1.S1_x_at	468.5922	129.7094	3.612631	AT1G75750.1	2.00E-36	GASA1 (GAST1 PROTEIN HOMOLOG 1)
Cit.16903.1.S1_x_at	4185.651	1160.917	3.60547		NA	
Cit.15414.1.S1_at	171.9914	48.07193	3.577793	AT5G61520.1	1.00E-174	hexose transporter, putative
Cit.20757.1.S1_at	405.4784	113.4412	3.574349		NA	

Cit.2927.1.S1_s_at	1599.897	448.0062	3.571149	AT5G23810.1	2.00E-96	AAP7; amino acid transmembrane transporter
Cit.19536.1.S1_at	191.1839	53.65848	3.562976		NA	
Cit.32755.1.S1_at	137.5333	38.62104	3.561098	ATMG00080.1	1.00E-66	encodes a mitochondrial ribosomal protein L16, which is a constitue
Cit.13424.1.S1_at	115.1341	32.65705	3.525551	AT4G19230.1	0	CYP707A1; (+)-abscisic acid 8'-hydroxylase/ oxygen binding
Cit.9388.1.S1_x_at	262.9406	74.61459	3.523984	AT5G61820.1	7.00E-24	FUNCTIONS IN: molecular_function unknown; INVOLVED IN: biologi
Cit.13963.1.S1_s_at	705.8954	200.6684	3.517721	AT1G24130.1	1.00E-135	transducin family protein / WD-40 repeat family protein
Cit.17187.1.S1_at	74.37527	21.19658	3.508834	AT1G05575.1	4.00E-17	unknown protein
Cit.18556.1.S1_at	130.7322	37.28765	3.506046		NA	
Cit.1751.1.S1_s_at	192.4738	55.17068	3.488697	AT1G67920.1	2.00E-13	unknown protein
Cit.36604.1.S1_at	123.2328	35.47857	3.473443	AT3G17520.1	2.00E-17	late embryogenesis abundant domain-containing protein / LEA dom
Cit.21295.1.S1_at	190.8201	54.988	3.470214		NA	
Cit.28253.1.S1_at	164.424	47.39031	3.46957	AT3G26220.1	9.00E-54	CYP71B3; electron carrier/ heme binding / iron ion binding / monoo
Cit.30526.1.S1_at	170.625	49.26545	3.463381	AT3G55610.1	2.00E-88	P5CS2 (DELTA 1-PYRROLINE-5-CARBOXYLATE SYNTHASE 2); catalytic
Cit.12503.1.S1_at	1109.831	320.6873	3.460789	AT3G45600.1	1.00E-133	TET3 (TETRASPANIN3)
Cit.9568.1.S1_s_at	7378.438	2142.511	3.443827	AT5G64260.1	1.00E-125	EXL2 (EXORDIUM LIKE 2)
Cit.14472.1.S1_s_at	994.8843	289.5532	3.435929		NA	
Cit.1729.1.S1_s_at	743.1808	216.5539	3.431851	AT5G20860.1	7.00E-87	pectinesterase family protein
Cit.22984.1.S1_x_at	297.7136	86.77439	3.430892	AT5G05320.1	6.00E-20	monooxygenase, putative (MO3)
Cit.15607.1.S1_at	72.35815	21.1982	3.41341	AT5G06530.1	5.00E-73	ABC transporter family protein
Cit.28162.1.S1_at	116.3212	34.09544	3.411635	AT1G17020.1	4.00E-63	SRG1 (SENESCENCE-RELATED GENE 1); oxidoreductase, acting on di
Cit.9388.1.S1_at	191.1109	56.74449	3.36792	AT5G61820.1	7.00E-24	FUNCTIONS IN: molecular_function unknown; INVOLVED IN: biologi
Cit.29637.1.S1_at	161.5085	48.18438	3.351885		NA	
Cit.18120.1.S1_at	406.0333	121.1401	3.351766	AT5G56550.1	2.00E-21	OXS3 (OXIDATIVE STRESS 3)
Cit.5514.1.S1_at	800.089	242.0776	3.305093	AT3G63060.1	4.00E-56	EDL3 (EID1-like 3)
Cit.25526.1.S1_s_at	1406.745	426.92	3.295102	AT3G14620.1	3.00E-55	CYP72A8; electron carrier/ heme binding / iron ion binding / monoo
Cit.8587.1.S1_s_at	305.491	93.28269	3.274895		NA	
Cit.17371.1.S1_at	1160.131	354.6863	3.270865	AT3G25590.1	5.00E-29	unknown protein
Cit.21486.1.S1_x_at	1276.261	393.7759	3.241085		NA	
Cit.20852.1.S1_s_at	1205.891	372.5389	3.236953	AT5G65140.1	1.00E-136	trehalose-6-phosphate phosphatase, putative
Cit.21825.1.S1_s_at	450.8277	139.4455	3.233003	AT1G64380.1	8.00E-13	AP2 domain-containing transcription factor, putative
Cit.162.1.S1_x_at	2323.026	722.494	3.215288		NA	
Cit.10460.1.S1_at	65.74502	20.69743	3.176482	AT5G01750.2	4.00E-60	unknown protein
Cit.10567.1.S1_s_at	1322.327	416.957	3.171375	AT4G16380.1	8.00E-78	metal ion binding
Cit.12743.1.S1_at	121.7694	38.45559	3.166494	AT2G29420.1	9.00E-55	ATGSTU7 (ARABIDOPSIS THALIANA GLUTATHIONE S-TRANSFERASE
Cit.6852.1.S1_at	84.58209	26.80907	3.15498	AT2G36690.1	9.00E-58	oxidoreductase, 2OG-Fe(II) oxygenase family protein

Cit.10219.1.S1_at	1289.743	409.2403	3.151554	AT1G17840.1	3.00E-79	WBC11 (WHITE-BROWN COMPLEX HOMOLOG PROTEIN 11); ATPase
Cit.17589.1.S1_x_at	1377.77	437.3328	3.150393	AT4G02380.1	8.00E-15	SAG21 (SENESCENCE-ASSOCIATED GENE 21)
Cit.2351.1.S1_at	99.60349	31.62474	3.149543	AT2G38470.1	1.00E-124	WRKY33; transcription factor
Cit.1557.1.S1_s_at	7776.49	2474.876	3.142174		NA	
Cit.2141.1.S1_s_at	276.5929	88.07915	3.140277	AT2G16060.1	2.00E-63	AHB1 (ARABIDOPSIS HEMOGLOBIN 1); oxygen binding / oxygen tran
Cit.22998.1.S1_s_at	438.6657	140.3958	3.124493	AT2G26070.1	2.00E-95	RTE1 (REVERSION-TO-ETHYLENE SENSITIVITY1)
Cit.8500.1.S1_s_at	719.1729	230.2037	3.124072	AT5G09510.1	2.00E-73	40S ribosomal protein S15 (RPS15D)
Cit.21705.1.S1_s_at	141.0229	45.22544	3.118221	AT3G25400.1	1.00E-47	FUNCTIONS IN: molecular_function unknown; INVOLVED IN: biologi
Cit.5100.1.S1_s_at	117.6375	37.79005	3.112923	AT2G36790.1	1.00E-169	UGT73C6 (UDP-glucosyl transferase 73C6); UDP-glucosyltransferase
Cit.3086.1.S1_at	568.6094	183.6334	3.096438	AT1G68320.1	2.00E-63	MYB62 (myb domain protein 62); DNA binding / transcription factor
Cit.28102.1.S1_s_at	225.882	73.58833	3.069536	AT1G08830.2	3.00E-21	CSD1 (COPPER/ZINC SUPEROXIDE DISMUTASE 1); superoxide dismu
Cit.30695.1.S1_s_at	1436.285	468.6699	3.064598	AT3G21420.1	1.00E-151	oxidoreductase, 2OG-Fe(II) oxygenase family protein
Cit.6891.1.S1_at	387.1418	126.4347	3.06199	AT1G56140.1	8.00E-65	leucine-rich repeat family protein / protein kinase family protein
Cit.38374.1.S1_at	268.8497	88.79243	3.027845	AT3G55610.1	4.00E-15	P5CS2 (DELTA 1-PYRROLINE-5-CARBOXYLATE SYNTHASE 2); catalytic
Cit.14471.1.S1_at	1245.115	411.3602	3.026824		NA	
Cit.13667.1.S1_s_at	180.331	59.7859	3.01628	AT4G05070.1	3.00E-09	unknown protein
Cit.9134.1.S1_s_at	3595.268	1195.549	3.007211	AT4G19420.1	1.00E-174	pectinacetylesterase family protein
Cit.3448.1.S1_s_at	1847.017	614.8863	3.003835	AT2G38120.1	1.00E-173	AUX1 (AUXIN RESISTANT 1); amino acid transmembrane transporter
Cit.39201.1.S1_s_at	155.6506	51.84329	3.002329	AT1G21000.2	1.00E-100	zinc-binding family protein
Cit.17868.1.S1_s_at	142	47.35847	2.998408	AT1G30220.1	0	INT2 (INOSITOL TRANSPORTER 2); carbohydrate transmembrane tra
Cit.29750.1.S1_at	155.5243	52.03632	2.988764	AT5G42680.1	8.00E-90	unknown protein
Cit.12233.1.S1_s_at	541.4124	181.3503	2.985451	AT1G49320.1	3.00E-44	BURP domain-containing protein
Cit.13724.1.S1_s_at	285.209	95.7719	2.978003	AT5G60900.1	1.00E-106	RLK1 (RECEPTOR-LIKE PROTEIN KINASE 1); ATP binding / carbohydra
Cit.5916.1.S1_at	220.7642	74.29353	2.971513		NA	
Cit.6793.1.S1_at	113.862	38.32774	2.970747	AT4G32280.1	2.00E-09	IAA29 (INDOLE-3-ACETIC ACID INDUCIBLE 29); transcription factor
Cit.5117.1.S1_at	193.5756	65.31579	2.963688	AT3G62550.1	1.00E-53	universal stress protein (USP) family protein
Cit.11086.1.S1_at	759.7762	256.4616	2.962534	AT1G02070.1	2.00E-13	unknown protein
Cit.8198.1.S1_x_at	4040.56	1367.43	2.954857		NA	
Cit.25231.1.S1_at	95.70513	32.39319	2.954483		NA	
Cit.4078.1.S1_at	119.3129	40.44424	2.950059	AT5G50260.1	1.00E-158	cysteine proteinase, putative
Cit.6860.1.S1_at	232.9323	79.60856	2.925971	AT3G21690.1	6.00E-64	MATE efflux family protein
Cit.9089.1.S1_at	4248.193	1456.84	2.916033	AT2G45550.1	1.00E-134	CYP76C4; electron carrier/ heme binding / iron ion binding / monoo
Cit.9301.1.S1_s_at	246.3755	84.76789	2.906472	AT5G06570.2	3.00E-47	hydrolase
Cit.13816.1.S1_s_at	93.20013	32.12484	2.901186	AT5G06060.1	2.00E-89	tropinone reductase, putative / tropine dehydrogenase, putative
Cit.6333.1.S1_at	2035.772	701.7134	2.901145	AT4G17030.1	3.00E-83	ATEXLB1 (ARABIDOPSIS THALIANA EXPANSIN-LIKE B1)

Cit.10194.1.S1_x_at	85.51537	29.48966	2.899843	AT1G30910.1	1.00E-123	molybdenum cofactor sulfurase family protein
Cit.12814.1.S1_s_at	1319.223	455.3976	2.89686	AT5G07330.1	2.00E-36	unknown protein
Cit.9817.1.S1_at	1465.22	506.5077	2.892789		NA	
Cit.17907.1.S1_at	130.9468	45.34678	2.887676	AT1G49320.1	3.00E-44	BURP domain-containing protein
Cit.10032.1.S1_s_at	786.8163	273.4063	2.877828	AT1G75750.1	3.00E-37	GASA1 (GAST1 PROTEIN HOMOLOG 1)
Cit.27318.1.S1_s_at	296.8849	103.3824	2.871716	AT2G42760.1	1.00E-38	unknown protein
Cit.12502.1.S1_s_at	1392.333	487.4131	2.856577	AT3G45600.1	1.00E-133	TET3 (TETRASPANIN3)
Cit.29822.1.S1_at	87.55316	30.72776	2.849318	AT1G77210.1	7.00E-75	sugar transporter, putative
Cit.5121.1.S1_at	104.6235	36.82979	2.84073	AT5G57685.1	2.00E-33	AtGDU3 (Arabidopsis thaliana GLUTAMINE DUMPER 3)
Cit.17346.1.S1_at	350.3742	124.333	2.818031	AT1G31335.1	2.00E-06	unknown protein
Cit.23748.1.S1_at	789.0158	281.5818	2.802084	AT5G67385.1	3.00E-72	protein binding / signal transducer
Cit.10032.1.S1_x_at	834.2311	299.424	2.78612	AT1G75750.1	3.00E-37	GASA1 (GAST1 PROTEIN HOMOLOG 1)
Cit.8663.1.S1_x_at	141.2675	50.74897	2.783653	AT1G68725.1	5.00E-06	AGP19 (ARABINOGALACTAN-PROTEIN 19)
Cit.2306.1.S1_at	1063.65	383.4781	2.773692		NA	
Cit.3966.1.S1_s_at	75.11527	27.10577	2.771191	AT2G37130.1	1.00E-134	peroxidase 21 (PER21) (P21) (PRXR5)
Cit.17289.1.S1_at	3275.705	1182.494	2.770166		NA	
Cit.32528.1.S1_at	405.6462	146.5015	2.768888	AT5G53280.1	2.00E-32	PDV1 (PLASTID DIVISION1)
Cit.31577.1.S1_at	67.83344	24.51626	2.766876	AT1G52080.1	9.00E-26	AR791; actin binding
Cit.712.1.S1_x_at	669.2452	242.458	2.760252		NA	
Cit.25795.1.S1_s_at	5532.491	2007.051	2.756527	AT3G57520.1	0	AtSIP2 (Arabidopsis thaliana seed imbibition 2); hydrolase, hydrolyzi
Cit.15009.1.S1_at	81.20026	29.56657	2.746354	AT4G10120.2	1.00E-160	ATSPS4F; transferase, transferring glycosyl groups
Cit.20223.1.S1_at	272.6504	99.31647	2.745269	AT5G54160.1	6.00E-64	ATOMT1 (O-METHYLTRANSFERASE 1); caffeate O-methyltransferase
Cit.10363.1.S1_s_at	1107.824	404.9355	2.735804	AT3G23730.1	1.00E-124	xyloglucan:xyloglucosyl transferase, putative / xyloglucan endotrans
Cit.1928.1.S1_s_at	2473.585	908.5209	2.722651	AT1G78860.1	1.00E-117	curculin-like (mannose-binding) lectin family protein
Cit.10753.1.S1_at	190.8266	70.10528	2.722	AT4G19040.2	1.00E-60	EDR2; lipid binding
Cit.30448.1.S1_x_at	116.7765	43.11567	2.708447	AT4G02380.1	5.00E-20	SAG21 (SENESCENCE-ASSOCIATED GENE 21)
Cit.19576.1.S1_at	397.7394	147.0713	2.704398		NA	
Cit.6015.1.S1_at	194.7485	72.04474	2.703161	AT2G39450.1	1.00E-165	MTP11; cation transmembrane transporter/ manganese ion transm
Cit.7331.1.S1_at	67.04414	24.84235	2.698784	AT2G12190.1	4.00E-54	cytochrome P450, putative
Cit.1869.1.S1_at	1986.307	739.416	2.686319		NA	
Cit.3215.1.S1_s_at	470.0292	175.721	2.674861	AT5G03230.1	8.00E-41	unknown protein
Cit.193.1.S1_s_at	246.9426	92.46617	2.670626	AT5G53370.1	0	PMEPCRF (PECTIN METHYLESTERASE PCR FRAGMENT F); pectineste
Cit.28173.1.S1_s_at	89.79433	33.73592	2.661683	AT4G21380.1	1.00E-106	ARK3 (A. THALIANA RECEPTOR KINASE 3); kinase/ transmembrane r
Cit.40221.1.S1_s_at	156.0593	58.69704	2.658725		NA	
Cit.20640.1.S1_at	105.8623	39.83305	2.65765	AT1G64380.1	2.00E-08	AP2 domain-containing transcription factor, putative

Cit.32853.1.S1_at	147.0388	55.37831	2.65517	AT3G15760.1	3.00E-22	unknown protein
Cit.5651.1.S1_s_at	605.7822	228.6123	2.649823	AT1G77210.1	2.00E-68	sugar transporter, putative
Cit.25471.1.S1_at	139.1766	52.6627	2.642793	AT1G78000.2	9.00E-96	SULTR1;2 (SULFATE TRANSPORTER 1;2); sulfate transmembrane tra
Cit.24915.1.S1_at	55.58307	21.14729	2.628378		NA	
Cit.35499.1.S1_at	109.652	41.74657	2.626611	AT4G22620.1	5.00E-44	auxin-responsive family protein
Cit.17264.1.S1_s_at	79.25228	30.1776	2.626196	AT2G05540.1	4.00E-16	glycine-rich protein
Cit.31285.1.S1_at	661.2087	251.8709	2.625189	AT2G18700.1	7.00E-51	ATTPS11; transferase, transferring glycosyl groups
Cit.10060.1.S1_s_at	1863.1	712.5918	2.61454	AT3G47340.1	0	ASN1 (GLUTAMINE-DEPENDENT ASPARAGINE SYNTHASE 1); asparag
Cit.2926.1.S1_at	65.04965	24.89308	2.613162	AT5G23810.1	6.00E-62	AAP7; amino acid transmembrane transporter
Cit.26434.1.S1_at	450.9729	172.6329	2.612323	AT1G28260.2	1.00E-14	INVOLVED IN: biological_process unknown; LOCATED IN: cellular_cc
Cit.4258.1.S1_s_at	582.2373	222.9261	2.611795	AT1G14870.1	9.00E-53	FUNCTIONS IN: molecular_function unknown; INVOLVED IN: respon
Cit.5100.1.S1_at	250.6245	95.95988	2.611763	AT2G36790.1	1.00E-169	UGT73C6 (UDP-glucosyl transferase 73C6); UDP-glucosyltransferase
Cit.10280.1.S1_x_at	6556.172	2511.464	2.610498	AT3G57520.1	0	AtSIP2 (Arabidopsis thaliana seed imbibition 2); hydrolase, hydrolyzi
Cit.21233.1.S1_at	74.58328	28.57239	2.610327	AT2G31090.1	1.00E-30	unknown protein
Cit.4934.1.S1_at	1264.961	485.205	2.607065	AT1G22360.1	1.00E-155	AtUGT85A2 (UDP-glucosyl transferase 85A2); UDP-glycosyltransfera
Cit.38054.1.S1_at	62.61427	24.05694	2.602753		NA	
Cit.14363.1.S1_at	119.1674	45.80174	2.601809	AT2G39210.1	2.00E-20	nodulin family protein
Cit.14906.1.S1_at	651.3691	250.4946	2.600332	AT5G65140.1	1.00E-136	trehalose-6-phosphate phosphatase, putative
Cit.7937.1.S1_at	278.5776	107.1453	2.599998	AT5G01900.1	4.00E-09	WRKY62; transcription factor
Cit.9025.1.S1_x_at	533.45	205.6134	2.594432	AT1G03400.1	1.00E-95	2-oxoglutarate-dependent dioxygenase, putative
Cit.11576.1.S1_at	661.0916	255.099	2.59151	AT1G30220.1	3.00E-87	INT2 (INOSITOL TRANSPORTER 2); carbohydrate transmembrane tra
Cit.3817.1.S1_x_at	831.8137	320.9852	2.591439	AT4G22920.1	1.00E-99	NYE1 (NON-YELLOWING 1)
Cit.15606.1.S1_at	172.2275	66.47957	2.590683	AT1G80130.1	1.00E-56	Tetratricopeptide repeat (TPR)-like superfamily protein; FUNCTIONS
Cit.4047.1.S1_at	97.50961	37.76426	2.582061	AT2G02990.1	7.00E-96	RNS1 (RIBONUCLEASE 1); endoribonuclease/ ribonuclease
Cit.4030.1.S1_at	355.1245	137.812	2.576876	AT1G60190.1	4.00E-82	armadillo/beta-catenin repeat family protein / U-box domain-conta
Cit.28545.1.S1_s_at	1465.412	571.3878	2.564654	AT1G28230.1	9.00E-97	PUP1 (PURINE PERMEASE 1); purine nucleoside transmembrane tra
Cit.828.1.S1_at	143.326	55.93803	2.562228	AT1G30270.1	3.00E-59	CIPK23 (CBL-INTERACTING PROTEIN KINASE 23); kinase/ protein bin
Cit.31072.1.S1_at	87.97473	34.46346	2.552696	AT3G06880.1	6.00E-07	nucleotide binding
Cit.16490.1.S1_at	69.15717	27.09579	2.552322	AT1G11190.1	1.00E-130	BFN1 (BIFUNCTIONAL NUCLEASE I); T/G mismatch-specific endonucl
Cit.3694.1.S1_s_at	499.2164	195.9477	2.547702	AT1G07430.1	1.00E-122	protein phosphatase 2C, putative / PP2C, putative
Cit.26251.1.S1_at	2171.511	852.3862	2.547567	AT5G58960.1	3.00E-50	GIL1 (GRAVITROPIC IN THE LIGHT)
Cit.17487.1.S1_at	591.6462	232.7283	2.542219	AT2G19570.1	1.00E-102	CDA1 (CYTIDINE DEAMINASE 1); cytidine deaminase
Cit.12345.1.S1_s_at	576.4248	227.2292	2.536755	AT4G12320.1	1.00E-116	CYP706A6; electron carrier/ heme binding / iron ion binding / mono
Cit.5377.1.S1_at	682.4046	269.633	2.530865	AT4G37370.1	1.00E-156	CYP81D8; electron carrier/ heme binding / iron ion binding / monoc
Cit.12906.1.S1_s_at	309.5542	122.3929	2.529184	AT3G50930.1	1.00E-98	BCS1 (CYTOCHROME BC1 SYNTHESIS); ATP binding / ATPase/ nuclec

Cit.5382.1.S1_at	283.8909	112.3361	2.527156	AT4G38060.2	7.00E-17	unknown protein
Cit.9020.1.S1_s_at	299.2076	118.5183	2.524569	AT4G30880.1	5.00E-21	protease inhibitor/seed storage/lipid transfer protein (LTP) family p
Cit.13688.1.S1_at	389.6942	154.3739	2.524353	AT2G26070.1	2.00E-95	RTE1 (REVERSION-TO-ETHYLENE SENSITIVITY1)
Cit.9089.1.S1_s_at	770.2049	305.5038	2.521098	AT2G45550.1	1.00E-134	CYP76C4; electron carrier/ heme binding / iron ion binding / monoo
Cit.26654.1.S1_x_at	203.4994	80.84155	2.517262		NA	
Cit.9091.1.S1_x_at	75.21467	29.90812	2.514858	AT2G45550.1	1.00E-128	CYP76C4; electron carrier/ heme binding / iron ion binding / monoo
Cit.2959.1.S1_at	684.4045	272.2413	2.513963	AT5G13000.2	0	ATGSL12 (glucan synthase-like 12); 1,3-beta-glucan synthase/ transf
Cit.13929.1.S1_at	99.88236	39.86393	2.505582	AT3G48660.1	2.00E-27	unknown protein
Cit.20668.1.S1_s_at	52.47596	20.97129	2.502276	AT1G27940.1	1.00E-168	PGP13 (P-GLYCOPROTEIN 13); ATPase, coupled to transmembrane r
Cit.25298.1.S1_s_at	143.6757	57.47706	2.499705	AT3G05690.1	4.00E-08	NF-YA2 (NUCLEAR FACTOR Y, SUBUNIT A2); transcription factor
Cit.24477.1.S1_s_at	1771.039	708.8872	2.498337	AT5G26230.1	7.00E-74	unknown protein
Cit.19280.1.S1_at	59.3521	23.77681	2.496218	AT4G21440.1	6.00E-16	ATMYB102 (ARABIDOPSIS MYB-LIKE 102); DNA binding / transcriptic
Cit.3195.1.S1_s_at	570.9896	228.7424	2.496212	AT1G33055.1	7.00E-13	unknown protein
Cit.28142.1.S1_s_at	226.4656	90.82072	2.493546	AT3G13730.1	8.00E-87	CYP90D1; oxidoreductase, acting on paired donors, with incorporati
Cit.8231.1.S1_s_at	2794.934	1121.366	2.492437	AT2G01890.1	1.00E-131	PAP8 (PURPLE ACID PHOSPHATASE 8); acid phosphatase/ protein se
Cit.9944.1.S1_at	331.024	133.0868	2.487279	AT4G37990.1	1.00E-156	ELI3-2 (ELICITOR-ACTIVATED GENE 3-2); aryl-alcohol dehydrogenase
Cit.21853.1.S1_at	54.68592	22.01504	2.484025	AT5G42830.1	7.00E-24	transferase family protein
Cit.21825.1.S1_at	185.5482	74.77086	2.481558	AT1G64380.1	8.00E-13	AP2 domain-containing transcription factor, putative
Cit.29379.1.S1_s_at	5387.725	2176.833	2.475029	AT2G27830.1	8.00E-49	FUNCTIONS IN: molecular_function unknown; EXPRESSED IN: 22 pla
Cit.26552.1.S1_at	60.7607	24.60271	2.469675	AT5G65380.1	6.00E-17	ripening-responsive protein, putative
Cit.16334.1.S1_at	53.64548	21.73565	2.468087	AT1G79900.1	2.00E-33	BAC2; L-ornithine transmembrane transporter/ binding / carnitine:a
Cit.31278.1.S1_at	58.92369	24.0426	2.450804	AT4G34280.1	2.00E-42	transducin family protein / WD-40 repeat family protein
Cit.3817.1.S1_at	162.9586	66.87203	2.436872	AT4G22920.1	1.00E-99	NYE1 (NON-YELLOWING 1)
Cit.22776.1.S1_s_at	403.978	166.0626	2.432685	AT4G32480.1	5.00E-71	unknown protein
Cit.35594.1.S1_at	87.33662	35.90228	2.43262	AT3G07870.1	9.00E-28	F-box family protein
Cit.29243.1.S1_at	66.41988	27.30451	2.432561	AT1G30400.2	4.00E-41	MRP1 (ARABIDOPSIS THALIANA MULTIDRUG RESISTANCE-ASSOCIAT
Cit.6364.1.S1_s_at	53.07177	21.81838	2.432434	AT2G15220.1	3.00E-83	secretory protein, putative
Cit.27139.1.S1_s_at	73.86269	30.36788	2.432264	AT5G65210.5	1.00E-144	TGA1; DNA binding / calmodulin binding / transcription factor
Cit.8454.1.S1_at	603.3276	248.0529	2.432254	AT5G04000.1	2.00E-22	unknown protein
Cit.30402.1.S1_at	606.0538	249.5115	2.428961	AT1G12780.1	1.00E-106	UGE1 (UDP-D-glucose/UDP-D-galactose 4-epimerase 1); UDP-glucos
Cit.1664.1.S1_s_at	4424.454	1822.449	2.427752	AT1G22770.1	0	GI (GIGANTEA)
Cit.11452.1.S1_at	1223.296	503.9794	2.427274	AT3G43810.1	6.00E-27	CAM7 (CALMODULIN 7); calcium ion binding
Cit.9345.1.S1_at	170.9996	70.50619	2.425313	AT5G52570.1	7.00E-98	BETA-OHASE 2 (BETA-CAROTENE HYDROXYLASE 2); carotene beta-ri
Cit.9300.1.S1_s_at	396.6886	163.6358	2.424216	AT3G28860.1	7.00E-92	ABCB19; ATPase, coupled to transmembrane movement of substan
Cit.19663.1.S1_at	108.2695	44.82685	2.415282	AT5G58570.1	4.00E-10	unknown protein

Cit.21857.1.S1_at	905.3608	374.9351	2.414713	AT2G45130.1	4.00E-29	SPX3 (SPX DOMAIN GENE 3)
Cit.4486.1.S1_at	170.621	71.08697	2.400173	AT5G01750.2	5.00E-70	unknown protein
Cit.19678.1.S1_s_at	1545.873	645.3125	2.395542	AT2G27830.1	2.00E-48	FUNCTIONS IN: molecular_function unknown; EXPRESSED IN: 22 pla
Cit.15030.1.S1_at	289.3047	120.7804	2.395295	AT5G51920.1	3.00E-18	catalytic/ pyridoxal phosphate binding
Cit.14302.1.S1_at	90.4341	37.81847	2.391268	AT4G17220.1	1.00E-84	ATMAP70-5 (microtubule-associated proteins 70-5); microtubule bi
Cit.22319.1.S1_s_at	488.3216	204.7074	2.385461	AT1G55850.1	3.00E-66	ATCSLE1; cellulose synthase/ transferase, transferring glycosyl grou
Cit.13595.1.S1_at	150.6367	63.18698	2.383983	AT4G15920.1	1.00E-64	INVOLVED IN: biological_process unknown; LOCATED IN: endomem
Cit.4810.1.S1_at	366.0489	153.6562	2.382259	AT3G23240.1	2.00E-64	ERF1 (ETHYLENE RESPONSE FACTOR 1); DNA binding / transcription
Cit.21996.1.S1_at	237.8756	99.95713	2.379776		NA	
Cit.29884.1.S1_s_at	120.9424	50.91254	2.375493	AT1G63420.1	7.00E-51	INVOLVED IN: biological_process unknown; EXPRESSED IN: 22 plant
Cit.5793.1.S1_s_at	1126.374	474.245	2.375089	AT3G03341.1	3.00E-28	unknown protein
Cit.10665.1.S1_at	598.6596	252.0868	2.374815	AT1G15100.1	2.00E-39	RHA2A; protein binding / ubiquitin-protein ligase/ zinc ion binding
Cit.10278.1.S1_x_at	3155.559	1329.986	2.372626	AT3G57520.1	5.00E-81	AtSIP2 (Arabidopsis thaliana seed imbibition 2); hydrolase, hydrolyz
Cit.21208.1.S1_at	187.2679	79.05489	2.368834	AT3G26840.1	3.00E-21	esterase/lipase/thioesterase family protein
Cit.21094.1.S1_s_at	789.7114	333.3987	2.36867	AT5G24120.1	9.00E-50	SIGE (SIGMA FACTOR E); DNA binding / DNA-directed RNA polymera
Cit.12814.1.S1_at	1065.238	450.7106	2.363463	AT5G07330.1	2.00E-36	unknown protein
Cit.10645.1.S1_at	47.79974	20.23322	2.362439	AT4G27500.1	6.00E-34	PPI1 (PROTON PUMP INTERACTOR 1); protein binding
Cit.26534.1.S1_s_at	468.9706	198.6479	2.360813	AT3G21690.1	6.00E-64	MATE efflux family protein
Cit.10319.1.S1_at	1756.979	744.7831	2.359048	AT3G51730.1	4.00E-53	saposin B domain-containing protein
Cit.19968.1.S1_at	473.5504	200.9427	2.356644	AT5G36210.1	3.00E-47	serine-type peptidase
Cit.14020.1.S1_at	967.8602	410.7784	2.356161	AT5G53970.1	1.00E-180	aminotransferase, putative
Cit.7966.1.S1_x_at	902.1342	383.7964	2.350554	AT1G17860.1	2.00E-31	trypsin and protease inhibitor family protein / Kunitz family protein
Cit.38990.1.S1_at	103.0693	43.91354	2.347096	AT1G21000.2	1.00E-100	zinc-binding family protein
Cit.21919.1.S1_s_at	1394.966	595.5466	2.342329	AT2G39130.1	1.00E-178	amino acid transporter family protein
Cit.10060.1.S1_at	563.7098	240.7768	2.341213	AT3G47340.1	0	ASN1 (GLUTAMINE-DEPENDENT ASPARAGINE SYNTHASE 1); asparag
Cit.13579.1.S1_s_at	239.3355	102.2549	2.340577	AT5G65660.1	3.00E-48	hydroxyproline-rich glycoprotein family protein
Cit.1983.1.S1_x_at	359.3327	153.5909	2.339544	AT5G56670.1	1.00E-29	40S ribosomal protein S30 (RPS30C)
Cit.4566.1.S1_at	883.7515	378.8053	2.332997	AT4G37580.1	6.00E-70	HLS1 (HOOKLESS 1); N-acetyltransferase
Cit.2350.1.S1_at	170.6837	73.21362	2.331311	AT2G38470.1	2.00E-42	WRKY33; transcription factor
Cit.13936.1.S1_s_at	116.8521	50.33518	2.32148		NA	
Cit.12839.1.S1_s_at	220.3879	94.94212	2.321287	AT3G07350.1	1.00E-78	unknown protein
Cit.14332.1.S1_at	283.9555	122.6799	2.314605	AT1G76130.1	2.00E-50	AMY2 (ALPHA-AMYLASE-LIKE 2); alpha-amylase/ calcium ion binding
Cit.7699.1.S1_at	193.6266	83.66436	2.314326	AT1G78815.1	6.00E-69	LSH7 (LIGHT SENSITIVE HYPOCOTYLS 7)
Cit.36076.1.S1_at	77.89651	33.69774	2.311624	AT2G05910.1	2.00E-58	unknown protein
Cit.29328.1.S1_at	1350.257	584.3483	2.310706	AT3G61890.1	7.00E-13	ATHB-12 (ARABIDOPSIS THALIANA HOMEOBOX 12); transcription ac

Cit.29525.1.S1_at	661.0657	286.3914	2.30826	AT1G19530.1	1.00E-16	unknown protein
Cit.4608.1.S1_at	72.68617	31.52263	2.305841	AT1G23550.1	3.00E-36	SRO2 (SIMILAR TO RCD ONE 2); NAD+ ADP-ribosyltransferase
Cit.21357.1.S1_at	58.20122	25.26306	2.303807		NA	
Cit.5916.1.S1_s_at	282.7675	122.7842	2.302963		NA	
Cit.6564.1.S1_at	58.62197	25.475	2.301157	AT1G55850.1	1.00E-102	ATCSLE1; cellulose synthase/ transferase, transferring glycosyl grou
Cit.6160.1.S1_at	69.66086	30.37363	2.293465	AT2G32510.1	2.00E-86	MAPKKK17; ATP binding / kinase/ protein kinase/ protein serine/thr
Cit.14227.1.S1_at	869.7731	380.1168	2.288173	AT4G13540.1	6.00E-30	unknown protein
Cit.11757.1.S1_at	305.7442	133.7729	2.285547	AT2G41870.1	6.00E-70	remorin family protein
Cit.1679.1.S1_at	748.1736	327.7644	2.282657	AT1G29050.1	1.00E-154	unknown protein
Cit.33180.1.S1_at	47.32221	20.76236	2.279231	AT3G06880.1	3.00E-15	nucleotide binding
Cit.7782.1.S1_at	176.2723	77.36002	2.278597	AT4G31590.1	1.00E-120	ATCSLC5 (CELLULOSE-SYNTHASE LIKE C5); cellulose synthase/ transf
Cit.28282.1.S1_at	68.83244	30.2095	2.278503		NA	
Cit.1200.1.S1_s_at	1179.935	518.1328	2.277283	AT4G11650.1	9.00E-81	ATOSM34 (osmotin 34)
Cit.20666.1.S1_at	46.67529	20.51347	2.275348	AT3G03470.1	1.00E-09	CYP89A9; electron carrier/ heme binding / iron ion binding / monoo
Cit.30527.1.S1_s_at	2226.537	981.1501	2.269313	AT2G39800.1	0	P5CS1 (DELTA1-PYRROLINE-5-CARBOXYLATE SYNTHASE 1); delta1-p
Cit.1930.1.S1_at	931.8082	410.6683	2.269004	AT1G78850.1	1.00E-97	curculin-like (mannose-binding) lectin family protein
Cit.25840.1.S1_s_at	3015.976	1331.835	2.264527	AT1G17840.1	0	WBC11 (WHITE-BROWN COMPLEX HOMOLOG PROTEIN 11); ATPase
Cit.2928.1.S1_s_at	176.2196	78.02772	2.258423	AT5G23810.1	1.00E-103	AAP7; amino acid transmembrane transporter
Cit.21090.1.S1_at	119.7373	53.04099	2.257448	AT1G12240.1	6.00E-44	ATBETAFRUCT4; beta-fructofuranosidase/ hydrolase, hydrolyzing O-
Cit.13915.1.S1_at	1037.299	460.4925	2.252586	AT3G14440.1	0	NCED3 (NINE-CIS-EPOXYCAROTENOID DIOXYGENASE 3); 9-cis-epoxy
Cit.28081.1.S1_at	325.1061	144.5484	2.249116	AT1G56130.1	2.00E-55	leucine-rich repeat family protein / protein kinase family protein
Cit.10915.1.S1_s_at	499.6905	222.364	2.247174	AT5G02020.1	7.00E-38	unknown protein
Cit.1775.1.S1_s_at	453.7674	202.4258	2.241648	AT1G56600.1	1.00E-156	AtGolS2 (Arabidopsis thaliana galactinol synthase 2); transferase, tr
Cit.11577.1.S1_s_at	1133.693	506.0197	2.240413	AT1G30220.1	0	INT2 (INOSITOL TRANSPORTER 2); carbohydrate transmembrane tra
Cit.18537.1.S1_at	108.8674	48.64061	2.2382	AT2G15890.1	5.00E-18	MEE14 (maternal effect embryo arrest 14)
Cit.4135.1.S1_at	59.48362	26.58186	2.237752	AT5G47260.1	2.00E-05	ATP binding / GTP binding / nucleoside-triphosphatase/ nucleotide
Cit.7705.1.S1_at	586.7792	262.687	2.233758	AT5G53990.1	2.00E-16	glycosyltransferase family protein
Cit.3283.1.S1_s_at	1027.556	460.33	2.232216	AT1G67750.1	0	pectate lyase family protein
Cit.5266.1.S1_s_at	845.3407	378.8429	2.231375	AT4G16380.1	4.00E-56	metal ion binding
Cit.12881.1.S1_at	179.243	80.35587	2.230615		NA	
Cit.29545.1.S1_at	85.84685	38.50851	2.229296	AT5G66730.1	1.00E-105	zinc finger (C2H2 type) family protein
Cit.34142.1.S1_at	141.2347	63.38712	2.228129	AT1G29800.2	1.00E-107	phosphoinositide binding / zinc ion binding
Cit.22861.1.S1_at	104.8459	47.0645	2.227707	AT5G04760.1	1.00E-32	myb family transcription factor
Cit.20223.1.S1_x_at	262.5823	117.8984	2.227191	AT5G54160.1	6.00E-64	ATOMT1 (O-METHYLTRANSFERASE 1); caffeate O-methyltransferase
Cit.30672.1.S1_x_at	1016.612	456.6138	2.226415		NA	

Cit.17346.1.S1_s_at	185.5734	83.3921	2.225312	AT1G31335.1	2.00E-06	unknown protein
Cit.15510.1.S1_at	242.7049	109.1346	2.223904	AT5G42146.1	9.00E-22	unknown protein
Cit.23815.1.S1_s_at	76.23911	34.31416	2.221797	AT3G05690.1	4.00E-08	NF-YA2 (NUCLEAR FACTOR Y, SUBUNIT A2); transcription factor
Cit.14168.1.S1_at	91.1539	41.04111	2.221039	AT1G22440.1	1.00E-151	alcohol dehydrogenase, putative
Cit.7775.1.S1_at	118.3214	53.32576	2.218841	AT1G17010.1	8.00E-11	oxidoreductase, 2OG-Fe(II) oxygenase family protein
Cit.23935.1.S1_at	93.95412	42.37278	2.217323	AT5G63120.2	3.00E-44	ethylene-responsive DEAD box RNA helicase, putative (RH30)
Cit.21925.1.S1_s_at	364.3968	164.8744	2.210148	AT3G07700.2	2.00E-93	ABC1 family protein
Cit.5504.1.S1_at	68.29491	30.90858	2.209578	AT1G66260.1	2.00E-16	RNA and export factor-binding protein, putative
Cit.5110.1.S1_s_at	116.3067	52.67974	2.207807	AT3G27110.2	1.00E-139	peptidase M48 family protein
Cit.35458.1.S1_at	104.2056	47.20871	2.207338		NA	
Cit.10277.1.S1_s_at	9032.77	4093.102	2.206827	AT3G57520.1	0	AtSIP2 (Arabidopsis thaliana seed imbibition 2); hydrolase, hydrolyzi
Cit.20062.1.S1_at	314.0818	142.3899	2.205787		NA	
Cit.9132.1.S1_s_at	89.15394	40.42945	2.205173	AT4G19420.1	1.00E-156	pectinacetylesterase family protein
Cit.22054.1.S1_at	230.8534	104.813	2.202526	AT3G11110.1	2.00E-35	zinc finger (C3HC4-type RING finger) family protein
Cit.39466.1.S1_at	264.6754	120.2194	2.201603		NA	
Cit.18662.1.S1_at	126.3016	57.40606	2.200144	AT1G63420.1	3.00E-20	INVOLVED IN: biological_process unknown; EXPRESSED IN: 22 plant
Cit.17316.1.S1_s_at	117.0362	53.1988	2.199978	AT5G06690.1	1.00E-55	WCRKC1 (WCRKC THIOREDOXIN 1)
Cit.1512.1.S1_s_at	213.2869	96.97033	2.199507	AT4G36860.1	1.00E-122	zinc ion binding
Cit.25344.1.S1_at	132.1125	60.0914	2.198526	AT3G42170.1	2.00E-15	DNA binding
Cit.17750.1.S1_x_at	848.6155	386.0653	2.198114		NA	
Cit.20852.1.S1_at	155.6968	70.86404	2.19712	AT4G39770.1	2.00E-20	trehalose-6-phosphate phosphatase, putative
Cit.30276.1.S1_at	73.96395	33.6646	2.197084	AT4G37300.1	7.00E-05	MEE59 (maternal effect embryo arrest 59)
Cit.18282.1.S1_at	85.83155	39.06723	2.197022		NA	
Cit.22763.1.S1_s_at	47.24914	21.51242	2.196366	AT3G23240.1	8.00E-52	ERF1 (ETHYLENE RESPONSE FACTOR 1); DNA binding / transcription
Cit.1005.1.S1_s_at	80.06181	36.47678	2.194871	AT4G31500.1	1.00E-142	CYP83B1 (CYTOCHROME P450 MONOOXYGENASE 83B1); oxidoredu
Cit.8194.1.S1_x_at	5172.105	2358.62	2.192852		NA	
Cit.13750.1.S1_at	729.7568	332.8155	2.192677	AT4G17030.1	8.00E-88	ATEXLB1 (ARABIDOPSIS THALIANA EXPANSIN-LIKE B1)
Cit.30511.1.S1_s_at	1507.566	687.6392	2.192379	AT5G61820.1	1.00E-147	FUNCTIONS IN: molecular_function unknown; INVOLVED IN: biologi
Cit.19449.1.S1_at	48.10355	21.95478	2.191029		NA	
Cit.372.1.S1_s_at	48.34868	22.13666	2.1841	AT5G26600.1	1.00E-176	catalytic/ pyridoxal phosphate binding
Cit.6350.1.S1_at	63.70574	29.18975	2.18247	AT1G59940.1	7.00E-12	ARR3 (RESPONSE REGULATOR 3); transcription regulator/ two-comp
Cit.4759.1.S1_at	380.9148	174.6088	2.181533		NA	
Cit.28589.1.S1_x_at	90.39083	41.45541	2.180435		NA	
Cit.36935.1.S1_s_at	1003.882	460.6628	2.179212	AT1G19640.1	2.00E-91	JMT (JASMONIC ACID CARBOXYL METHYLTRANSFERASE); jasmonate
Cit.30657.1.S1_s_at	4862.446	2232.661	2.17787	AT4G36740.1	1.00E-50	ATHB40 (ARABIDOPSIS THALIANA HOMEOBOX PROTEIN 40); DNA bi

Cit.18515.1.S1_at	121.2927	55.89388	2.170053		NA	
Cit.10279.1.S1_at	147.189	67.84019	2.169643	AT3G57520.1	0	AtSIP2 (Arabidopsis thaliana seed imbibition 2); hydrolase, hydrolyzi
Cit.30583.1.S1_at	54.13848	24.96386	2.168674	AT4G33865.1	7.00E-27	40S ribosomal protein S29 (RPS29C)
Cit.4776.1.S1_at	43.66365	20.16181	2.165661	AT4G24026.1	5.00E-05	unknown protein
Cit.16303.1.S1_at	189.3027	87.6249	2.160376	AT4G36810.1	1.00E-108	GGPS1 (GERANYLGERANYL PYROPHOSPHATE SYNTHASE 1); farnesyl
Cit.17803.1.S1_at	1962.005	908.1894	2.160348		NA	
Cit.28236.1.S1_at	140.3873	65.02821	2.158868	AT4G03420.1	3.00E-24	unknown protein
Cit.5235.1.S1_at	917.4626	424.9885	2.158794		NA	
Cit.10914.1.S1_s_at	1099.507	509.4054	2.158413	AT5G02020.1	7.00E-38	unknown protein
Cit.3069.1.S1_at	107.2481	49.7113	2.157419	AT5G50915.2	3.00E-44	basic helix-loop-helix (bHLH) family protein
Cit.17167.1.S1_at	107.834	49.99041	2.157094		NA	
Cit.34355.1.S1_at	44.03973	20.4265	2.15601	AT5G04000.1	7.00E-19	unknown protein
Cit.6308.1.S1_at	104.7501	48.6221	2.154372	AT2G29420.1	9.00E-60	ATGSTU7 (ARABIDOPSIS THALIANA GLUTATHIONE S-TRANSFERASE T
Cit.13189.1.S1_at	665.5337	309.1182	2.153007	AT2G26850.1	4.00E-44	F-box family protein
Cit.716.1.S1_x_at	74.99662	34.93331	2.146851	AT5G59310.1	4.00E-26	LTP4 (LIPID TRANSFER PROTEIN 4); lipid binding
Cit.10912.1.S1_at	58.23834	27.15344	2.144787	AT4G26740.1	1.00E-105	ATS1 (ARABIDOPSIS THALIANA SEED GENE 1); calcium ion binding
Cit.26004.1.S1_at	49.6837	23.17746	2.143621		NA	
Cit.4720.1.S1_at	725.0312	338.2863	2.143247	AT2G36970.1	1.00E-107	UDP-glucoronosyl/UDP-glucosyl transferase family protein
Cit.28293.1.S1_at	74.3825	34.71176	2.142862	AT1G02305.1	3.00E-20	cathepsin B-like cysteine protease, putative
Cit.4253.1.S1_x_at	182.5281	85.2008	2.142328		NA	
Cit.22916.1.S1_at	81.68926	38.13783	2.141948		NA	
Cit.5502.1.S1_at	305.2482	142.5703	2.141036	AT3G07600.1	8.00E-16	heavy-metal-associated domain-containing protein
Cit.20442.1.S1_at	143.8504	67.20541	2.140459		NA	
Cit.14453.1.S1_at	483.5656	226.0555	2.139145	AT3G25830.1	1.00E-138	ATTPS-CIN (terpene synthase-like sequence-1,8-cineole); (E)-beta-oc
Cit.4151.1.S1_s_at	87.54018	40.92938	2.13881	AT3G56710.1	5.00E-08	SIB1 (SIGMA FACTOR BINDING PROTEIN 1); binding / protein binding
Cit.40158.1.S1_s_at	1901.262	889.7998	2.13673	AT3G21670.1	0	nitrate transporter (NTP3)
Cit.27362.1.S1_s_at	272.5481	127.5946	2.136047	AT5G53050.1	1.00E-15	hydrolase, alpha/beta fold family protein
Cit.26653.1.S1_at	432.1301	202.4226	2.134792	AT4G13880.1	7.00E-15	AtRLP48 (Receptor Like Protein 48); protein binding
Cit.5793.1.S1_at	195.2115	91.53266	2.132698	AT3G03341.1	1.00E-28	unknown protein
Cit.26579.1.S1_at	166.086	77.90877	2.131801		NA	
Cit.12231.1.S1_s_at	821.3677	385.368	2.131385	AT2G23180.1	3.00E-80	CYP96A1; electron carrier/ heme binding / iron ion binding / monoo
Cit.2533.1.S1_s_at	387.685	181.9913	2.130239	AT3G62650.1	3.00E-30	unknown protein
Cit.35651.1.S1_s_at	1279.536	600.9069	2.129342	AT5G20700.1	8.00E-18	senescence-associated protein-related
Cit.21100.1.S1_at	56.9101	26.73037	2.129043		NA	
Cit.6201.1.S1_at	965.2866	453.8885	2.126704	AT3G21680.1	7.00E-12	unknown protein

Cit.7954.1.S1_at	124.0825	58.44302	2.123136	AT3G01472.1	1.00E-10	CPuORF33 (Conserved peptide upstream open reading frame 33)
Cit.3042.1.S1_s_at	922.3882	434.4465	2.123134	AT1G52565.1	1.00E-31	unknown protein
Cit.14823.1.S1_at	46.97791	22.15621	2.120304	AT3G09030.1	1.00E-167	potassium channel tetramerisation domain-containing protein
Cit.2942.1.S1_s_at	1706.202	805.3869	2.118487	AT1G19530.1	9.00E-17	unknown protein
Cit.39679.1.S1_s_at	49.25048	23.25051	2.118254	AT4G23010.2	1.00E-80	UDP-galactose transporter-related
Cit.4295.1.S1_at	43.93437	20.74379	2.117953	AT5G18640.1	1.00E-141	lipase class 3 family protein
Cit.18114.1.S1_at	174.1265	82.24693	2.117119		NA	
Cit.8156.1.S1_at	553.417	262.1389	2.111159	AT4G19170.1	4.00E-84	NCED4 (NINE-CIS-EPOXYCAROTENOID DIOXYGENASE 4)
Cit.21717.1.S1_at	250.3443	118.6018	2.110797	AT3G04720.1	2.00E-43	PR4 (PATHOGENESIS-RELATED 4); chitin binding
Cit.24586.1.S1_at	196.97	93.33244	2.110413	AT5G66150.1	4.00E-58	glycosyl hydrolase family 38 protein
Cit.20606.1.S1_at	77.65359	36.79579	2.110393	AT1G05575.1	1.00E-12	unknown protein
Cit.15956.1.S1_at	144.7746	68.6826	2.107879	AT3G54190.1	1.00E-141	FUNCTIONS IN: molecular_function unknown; INVOLVED IN: biologi
Cit.4253.1.S1_at	122.6794	58.23451	2.106644		NA	
Cit.18008.1.S1_at	80.56601	38.24607	2.106517		NA	
Cit.12539.1.S1_at	636.365	302.2397	2.105498	AT1G18100.1	1.00E-54	E12A11; phosphatidylethanolamine binding
Cit.25332.1.S1_at	117.7758	55.9391	2.105429		NA	
Cit.24426.1.S1_s_at	565.0106	268.3615	2.105409	AT1G73010.1	1.00E-110	phosphatase
Cit.19751.1.S1_s_at	2249.31	1068.897	2.104328	AT1G17840.1	0	WBC11 (WHITE-BROWN COMPLEX HOMOLOG PROTEIN 11); ATPase
Cit.361.1.S1_at	155.6634	73.9893	2.103864	AT1G15670.1	1.00E-108	kelch repeat-containing F-box family protein
Cit.22036.1.S1_at	65.84812	31.30179	2.103653	AT1G23780.1	1.00E-21	F-box family protein
Cit.35355.1.S1_s_at	386.243	183.8131	2.101281	AT1G18100.1	8.00E-55	E12A11; phosphatidylethanolamine binding
Cit.4868.1.S1_at	499.6215	238.1058	2.098317	AT3G15840.1	3.00E-99	PIFI (post-illumination chlorophyll fluorescence increase)
Cit.16070.1.S1_at	115.7373	55.17178	2.097763	AT2G38290.1	1.00E-137	ATAMT2 (AMMONIUM TRANSPORTER 2); ammonium transmembra
Cit.5127.1.S1_at	395.5148	188.595	2.097165	AT3G51000.1	1.00E-106	epoxide hydrolase, putative
Cit.31493.1.S1_at	136.4767	65.09142	2.096693		NA	
Cit.18263.1.S1_at	866.7565	413.401	2.096648		NA	
Cit.14342.1.S1_at	75.7766	36.20523	2.092974	AT3G62150.1	9.00E-65	PGP21 (P-GLYCOPROTEIN 21); ATPase, coupled to transmembrane r
Cit.13132.1.S1_s_at	334.6479	159.9671	2.09198	AT2G02800.2	1.00E-178	APK2B (PROTEIN KINASE 2B); ATP binding / kinase/ protein kinase/ p
Cit.7744.1.S1_at	117.2323	56.08578	2.090232	AT3G48690.1	2.00E-53	CXE12; carboxylesterase
Cit.5815.1.S1_at	226.8264	108.6418	2.087837	AT5G57740.1	4.00E-34	XBAT32; protein binding / zinc ion binding
Cit.24514.1.S1_at	42.56534	20.40049	2.086486	AT3G22990.1	7.00E-56	LFR (LEAF AND FLOWER RELATED); binding
Cit.13417.1.S1_s_at	291.1479	139.7237	2.08374	AT1G08650.1	4.00E-77	PPCK1 (PHOSPHOENOLPYRUVATE CARBOXYLASE KINASE); kinase/ p
Cit.30672.1.S1_at	1014.498	487.0717	2.082851		NA	
Cit.24901.1.S1_at	156.4053	75.11864	2.08211	AT2G44420.1	9.00E-19	protein N-terminal asparagine amidohydrolase family protein
Cit.33490.1.S1_at	160.8138	77.42834	2.076937	AT5G59450.1	6.00E-82	scarecrow-like transcription factor 11 (SCL11)

Cit.15185.1.S1_at	209.9146	101.0753	2.076814	AT3G25400.1	1.00E-47	FUNCTIONS IN: molecular_function unknown; INVOLVED IN: biologi
Cit.31300.1.S1_at	46.41499	22.35257	2.076495	AT3G23250.1	6.00E-66	MYB15 (MYB DOMAIN PROTEIN 15); DNA binding / transcription fac
Cit.14951.1.S1_at	1004.56	483.7961	2.076412	AT1G55910.1	1.00E-91	ZIP11 (ZINC TRANSPORTER 11 PRECURSOR); cation transmembrane
Cit.1422.1.S1_at	56.44352	27.20717	2.074583	AT5G18670.1	0	BMY3; beta-amylase/ catalytic/ cation binding
Cit.21040.1.S1_s_at	281.4758	135.9928	2.069785	AT5G11720.1	6.00E-73	alpha-glucosidase 1 (AGLU1)
Cit.18008.1.S1_s_at	71.09077	34.38474	2.067509		NA	
Cit.31324.1.S1_at	64.2963	31.11561	2.066368	AT5G65850.1	5.00E-07	F-box family protein
Cit.4230.1.S1_at	84.56381	40.99696	2.062685		NA	
Cit.5855.1.S1_at	649.3958	314.9925	2.061623	AT1G30110.1	4.00E-85	ATNUDX25 (ARABIDOPSIS THALIANA NUDIX HYDROLASE HOMOLOG
Cit.14905.1.S1_s_at	333.4874	161.8023	2.061079	AT5G65140.1	1.00E-136	trehalose-6-phosphate phosphatase, putative
Cit.1868.1.S1_s_at	2460.537	1195.468	2.058221		NA	
Cit.19984.1.S1_at	67.63444	32.98605	2.050395	AT5G53050.3	2.00E-65	hydrolase, alpha/beta fold family protein
Cit.3880.1.S1_at	252.1133	122.9997	2.049707	AT3G62730.1	5.00E-93	unknown protein
Cit.5754.1.S1_at	1888.145	922.2047	2.047425	AT3G54720.1	1.00E-138	AMP1 (ALTERED MERISTEM PROGRAM 1); carboxypeptidase/ dipep
Cit.24422.1.S1_at	53.63388	26.20512	2.046695	AT3G18760.1	1.00E-15	ribosomal protein S6 family protein
Cit.4135.1.S1_x_at	59.37753	29.01953	2.046123	AT5G47260.1	2.00E-05	ATP binding / GTP binding / nucleoside-triphosphatase/ nucleotide l
Cit.11232.1.S1_s_at	1623.092	793.4521	2.045608	AT2G43150.1	9.00E-87	proline-rich extensin-like family protein
Cit.14112.1.S1_at	178.1782	87.21804	2.042905	AT5G67390.2	2.00E-28	unknown protein
Cit.4549.1.S1_s_at	158.1236	77.4379	2.041941	AT1G05000.1	2.00E-86	tyrosine specific protein phosphatase family protein
Cit.9817.1.S1_s_at	3973.05	1945.917	2.041737	AT3G07910.1	3.00E-29	FUNCTIONS IN: molecular_function unknown; INVOLVED IN: biologi
Cit.445.1.S1_s_at	57.68005	28.27501	2.039966	AT5G01300.1	3.00E-69	phosphatidylethanolamine-binding family protein
Cit.10366.1.S1_s_at	727.8568	357.5555	2.035647	AT1G56300.1	3.00E-57	DNAJ heat shock N-terminal domain-containing protein
Cit.5575.1.S1_at	292.1655	144.0058	2.028845	AT3G50170.1	9.00E-50	unknown protein
Cit.16086.1.S1_at	106.4654	52.50225	2.027825	AT4G18530.1	2.00E-46	unknown protein
Cit.10811.1.S1_s_at	147.0362	72.51343	2.02771	AT2G43670.1	7.00E-25	glycosyl hydrolase family protein 17
Cit.8011.1.S1_at	43.14194	21.29326	2.026084	AT3G09390.1	6.00E-10	MT2A (METALLOTHIONEIN 2A); copper ion binding
Cit.17490.1.S1_at	74.23216	36.65409	2.025208		NA	
Cit.10522.1.S1_s_at	2756.888	1361.901	2.024294	AT5G67300.1	2.00E-87	MYBR1 (MYB DOMAIN PROTEIN R1); DNA binding / transcription fac
Cit.8874.1.S1_at	517.9818	256.0398	2.023052	AT4G38970.1	0	fructose-bisphosphate aldolase, putative
Cit.10453.1.S1_s_at	1972.263	974.9551	2.022927		NA	
Cit.11168.1.S1_at	211.5883	104.6053	2.02273	AT3G29090.1	1.00E-161	pectinesterase family protein
Cit.36111.1.S1_at	54.81485	27.10848	2.022055		NA	
Cit.14274.1.S1_s_at	327.0107	161.8327	2.020671	AT1G48380.1	5.00E-30	RHL1 (ROOT HAIRLESS 1); DNA binding / protein binding
Cit.37046.1.S1_s_at	1966.033	973.1323	2.020314	AT4G28240.1	7.00E-15	wound-responsive protein-related
Cit.10521.1.S1_at	253.5375	125.5083	2.020086		NA	

Cit.11855.1.S1_s_at	904.1491	447.5953	2.020015	AT5G05340.1	1.00E-103	peroxidase, putative
Cit.17268.1.S1_at	678.429	335.9167	2.019635		NA	
Cit.32867.1.S1_at	112.5812	55.77431	2.018514	AT1G07080.1	5.00E-15	gamma interferon responsive lysosomal thiol reductase family prote
Cit.14533.1.S1_at	391.1935	193.8835	2.017673	AT1G20823.1	1.00E-56	zinc finger (C3HC4-type RING finger) family protein
Cit.19339.1.S1_s_at	774.4628	383.9686	2.016995	AT5G01750.2	5.00E-70	unknown protein
Cit.12282.1.S1_at	132.3694	65.63712	2.016685	AT2G25670.2	4.00E-70	unknown protein
Cit.3693.1.S1_at	1100.257	547.6543	2.009036	AT2G29380.1	9.00E-68	protein phosphatase 2C, putative / PP2C, putative
Cit.8284.1.S1_s_at	675.5868	336.3475	2.008598	AT5G20650.1	3.00E-43	COPT5; copper ion transmembrane transporter/ high affinity coppe
Cit.21716.1.S1_at	157.7916	78.58924	2.007802		NA	
Cit.23213.1.S1_at	1562.449	779.218	2.00515	AT1G44820.1	2.00E-30	aminoacylase, putative / N-acyl-L-amino-acid amidohydrolase, puta
Cit.17840.1.S1_s_at	248.4141	124.0071	2.003225	AT1G02070.1	2.00E-13	unknown protein
Cit.9002.1.S1_at	228.3611	114.0556	2.002191	AT5G53750.1	3.00E-87	FUNCTIONS IN: molecular_function unknown; INVOLVED IN: respon
Cit.37992.1.S1_at	1040.199	519.6187	2.001851	AT3G19270.1	5.00E-28	CYP707A4; (+)-abscisic acid 8'-hydroxylase/ oxygen binding
Cit.30542.1.S1_s_at	2307.433	1152.787	2.001613	AT5G67360.1	0	ARA12; serine-type endopeptidase
Cit.22985.1.S1_at	20.93362	41.86005	-1.99966	AT3G09860.1	2.00E-33	unknown protein
Cit.5201.1.S1_at	238.1688	476.5318	-2.00082	AT2G14960.1	0	GH3.1
Cit.38103.1.S1_at	21.88422	43.78949	-2.00096	AT1G54280.1	5.00E-50	haloacid dehalogenase-like hydrolase family protein
Cit.18730.1.S1_at	22.50373	45.05103	-2.00194	AT4G13340.1	5.00E-05	leucine-rich repeat family protein / extensin family protein
Cit.22996.1.S1_s_at	37.63816	75.38343	-2.00285	AT2G28350.1	5.00E-39	ARF10 (AUXIN RESPONSE FACTOR 10); miRNA binding / transcriptio
Cit.29267.1.S1_at	62.38295	125.048	-2.00452	AT3G44735.1	4.00E-10	PSK1; growth factor
Cit.18030.1.S1_at	21.9864	44.11612	-2.00652	AT1G10490.1	3.00E-39	unknown protein
Cit.5112.1.S1_at	24.22134	48.65002	-2.00856	AT1G47128.1	1.00E-118	RD21 (responsive to dehydration 21); cysteine-type endopeptidase/
Cit.7162.1.S1_at	22.73988	45.6865	-2.00909	AT5G58820.1	1.00E-16	subtilase family protein
Cit.35979.1.S1_at	30.16337	60.6126	-2.00948		NA	
Cit.26471.1.S1_at	21.93179	44.07401	-2.00959	AT3G22910.1	1.00E-95	calcium-transporting ATPase, plasma membrane-type, putative / Ca
Cit.25548.1.S1_at	27.17571	54.72902	-2.01389	AT1G52760.1	2.00E-09	esterase/lipase/thioesterase family protein
Cit.21610.1.S1_at	66.20898	133.8276	-2.02129	AT2G29260.1	7.00E-70	tropinone reductase, putative / tropine dehydrogenase, putative
Cit.22742.1.S1_at	77.13766	156.2639	-2.02578	AT5G63660.1	6.00E-19	PDF2.5
Cit.23948.1.S1_x_at	27.829	56.38202	-2.02602		NA	
Cit.3456.1.S1_at	21.72157	44.03452	-2.02723	AT1G02850.2	1.00E-88	BGLU11 (BETA GLUCOSIDASE 11); hydrolase, hydrolyzing O-glycosyl
Cit.21883.1.S1_s_at	50.5615	102.5339	-2.0279	AT1G48970.1	8.00E-61	GTP binding / translation initiation factor
Cit.16028.1.S1_at	49.04116	99.50623	-2.02903	AT4G31080.1	2.00E-37	unknown protein
Cit.37141.1.S1_at	98.134	199.2204	-2.03009	AT2G48070.2	2.00E-32	RPH1 (RESISTANCE TO PHYTOPHTHORA 1)
Cit.37396.1.S1_at	25.16727	51.09416	-2.03018		NA	
Cit.20948.1.S1_at	26.80438	54.43387	-2.03078		NA	

Cit.22170.1.S1_x_at	41.40161	84.09808	-2.03128	AT5G37990.1	3.00E-12	S-adenosylmethionine-dependent methyltransferase/ methyltransf
Cit.30636.1.S1_at	96.98963	197.1784	-2.03298	AT5G27540.2	1.00E-163	MIRO1 (Miro-related GTP-ase 1); GTP binding
Cit.6130.1.S1_s_at	20.2731	41.22363	-2.03342	AT1G17260.1	5.00E-94	AHA10 (Autoinhibited H(+)-ATPase isoform 10); ATPase/ ATPase, co
Cit.15661.1.S1_at	391.9138	797.7257	-2.03546	AT4G24550.2	7.00E-96	clathrin adaptor complexes medium subunit family protein
Cit.38160.1.S1_at	25.9344	52.79637	-2.03577	AT5G59830.2	4.00E-16	unknown protein
Cit.38851.1.S1_s_at	39.29149	80.00111	-2.03609	AT5G65760.1	3.00E-11	serine carboxypeptidase S28 family protein
Cit.22134.1.S1_at	39.82283	81.08794	-2.03622	AT3G61110.1	3.00E-38	ARS27A (ARABIDOPSIS RIBOSOMAL PROTEIN S27); structural constit
Cit.39721.1.S1_x_at	805.9088	1643.689	-2.03955	AT3G46230.1	7.00E-69	ATHSP17.4
Cit.37424.1.S1_at	50.26038	102.5104	-2.03959		NA	
Cit.26512.1.S1_at	23.7517	48.47224	-2.04079	AT4G11740.1	2.00E-67	SAY1
Cit.25921.1.S1_at	26.29763	53.69197	-2.0417	AT5G43470.2	1.00E-11	RPP8 (RECOGNITION OF PERONOSPORA PARASITICA 8); nucleotide l
Cit.14254.1.S1_at	43.30011	88.66173	-2.04761		NA	
Cit.1887.1.S1_at	139.5172	286.4277	-2.05299	AT1G68090.1	3.00E-90	ANN5; calcium ion binding / calcium-dependent phospholipid bindir
Cit.13570.1.S1_at	31.15228	63.97183	-2.05352	AT5G05670.1	6.00E-89	signal recognition particle binding
Cit.28929.1.S1_at	28.22246	57.97375	-2.05417		NA	
Cit.25906.1.S1_at	39.94349	82.1365	-2.05632	AT1G07400.1	3.00E-12	17.8 kDa class I heat shock protein (HSP17.8-CI)
Cit.24729.1.S1_x_at	28.26157	58.18102	-2.05866	AT3G55010.2	1.00E-18	PUR5; ATP binding / phosphoribosylformylglycinamidine cyclo-ligase
Cit.9990.1.S1_x_at	788.4189	1623.632	-2.05935	AT1G33140.1	4.00E-89	PGY2 (PIGGYBACK2); structural constituent of ribosome
Cit.30979.1.S1_at	138.788	285.9839	-2.06058	AT4G18390.2	3.00E-36	TCP family transcription factor, putative
Cit.6073.1.S1_s_at	30.73825	63.42421	-2.06336	AT5G47000.1	3.00E-91	peroxidase, putative
Cit.28996.1.S1_at	26.7558	55.36155	-2.06914	AT5G12370.2	7.00E-28	SEC10 (EXOCYST COMPLEX COMPONENT SEC10)
Cit.7430.1.S1_at	22.72572	47.0309	-2.0695	AT4G16380.2	1.00E-15	LOCATED IN: cellular_component unknown; BEST Arabidopsis thalia
Cit.34886.1.S1_at	36.37075	75.31193	-2.07067		NA	
Cit.26491.1.S1_at	56.63282	117.5884	-2.07633	AT2G38820.1	1.00E-12	unknown protein
Cit.7855.1.S1_at	25.06986	52.17335	-2.08112		NA	
Cit.11962.1.S1_at	108.8912	226.7149	-2.08203	AT4G29100.1	9.00E-92	ethylene-responsive family protein
Cit.2543.1.S1_at	25.84562	53.88395	-2.08484	AT5G63160.1	7.00E-35	BT1 (BTB AND TAZ DOMAIN PROTEIN 1); protein binding / transcript
Cit.12322.1.S1_s_at	140.4674	293.3011	-2.08804	AT3G45020.1	1.00E-54	50S ribosomal protein-related
Cit.39105.1.S1_at	21.32788	44.5523	-2.08892	AT3G62680.1	6.00E-05	PRP3 (PROLINE-RICH PROTEIN 3); structural constituent of cell wall
Cit.27021.1.S1_at	31.59087	66.06311	-2.09121	AT4G26540.1	3.00E-26	kinase
Cit.34720.1.S1_at	20.45291	42.80849	-2.09303	AT5G03780.1	5.00E-22	TRFL10 (TRF-LIKE 10); DNA binding
Cit.9432.1.S1_at	33.70823	70.57021	-2.09356	AT5G20290.1	1.00E-105	40S ribosomal protein S8 (RPS8A)
Cit.23084.1.S1_at	38.67031	80.96929	-2.09384		NA	
Cit.31446.1.S1_at	54.54178	114.417	-2.09779		NA	
Cit.28860.1.S1_at	90.12408	189.0689	-2.09787	AT3G12580.1	7.00E-13	HSP70 (heat shock protein 70); ATP binding

Cit.791.1.S1_at	36.49741	76.92747	-2.10775	AT4G22670.1	3.00E-75	AtHip1 (Arabidopsis thaliana Hsp70-interacting protein 1); binding
Cit.4650.1.S1_at	227.5049	479.5261	-2.10776	AT2G33430.1	2.00E-95	DAL1 (DIFFERENTIATION AND GREENING-LIKE 1)
Cit.28986.1.S1_x_at	79.34119	167.288	-2.10846	AT1G08380.1	2.00E-42	PSAO (photosystem I subunit O)
Cit.36676.1.S1_at	30.45246	64.22674	-2.10908	AT4G32280.1	6.00E-19	IAA29 (INDOLE-3-ACETIC ACID INDUCIBLE 29); transcription factor
Cit.38806.1.S1_at	608.3167	1283.201	-2.10943	AT1G02730.1	2.00E-62	ATCSLD5; 1,4-beta-D-xylan synthase/ cellulose synthase
Cit.4416.1.S1_at	23.80457	50.23476	-2.1103	AT1G10470.1	2.00E-64	ARR4 (RESPONSE REGULATOR 4); protein binding / transcription reg
Cit.26201.1.S1_at	629.2783	1330.558	-2.11442	AT5G38710.1	4.00E-77	proline oxidase, putative / osmotic stress-responsive proline dehydr
Cit.21644.1.S1_at	20.34245	43.14766	-2.12107	AT2G17250.1	2.00E-05	EMB2762 (EMBRYO DEFECTIVE 2762)
Cit.7037.1.S1_at	28.80601	61.11368	-2.12156		NA	
Cit.11635.1.S1_at	222.5251	473.5711	-2.12817	AT5G49460.1	1.00E-170	ACLB-2 (ATP CITRATE LYASE SUBUNIT B 2); ATP citrate synthase
Cit.13768.1.S1_s_at	28.25837	60.16768	-2.1292	AT3G10720.2	0	pectinesterase, putative
Cit.21933.1.S1_at	26.81257	57.12532	-2.13054		NA	
Cit.22723.1.S1_x_at	21.78653	46.42914	-2.13109	AT3G46620.1	2.00E-05	zinc finger (C3HC4-type RING finger) family protein
Cit.18006.1.S1_at	405.7886	865.101	-2.1319	AT4G27450.1	1.00E-100	unknown protein
Cit.9964.1.S1_s_at	968.3178	2064.686	-2.13224	AT3G25810.1	1.00E-134	myrcene/ocimene synthase, putative
Cit.28381.1.S1_at	21.46954	45.8639	-2.13623	AT4G08850.1	1.00E-51	kinase
Cit.1779.1.S1_s_at	76.98117	164.4751	-2.13656	AT2G29500.1	4.00E-59	17.6 kDa class I small heat shock protein (HSP17.6B-CI)
Cit.36744.1.S1_s_at	30.11081	64.38702	-2.13834	AT3G26100.2	1.00E-130	regulator of chromosome condensation (RCC1) family protein
Cit.6773.1.S1_at	37.74214	80.71022	-2.13846	AT2G16890.2	8.00E-58	UDP-glucoronosyl/UDP-glucosyl transferase family protein
Cit.12711.1.S1_at	34.89979	74.74102	-2.14159	AT5G16150.3	9.00E-75	PGLCT (PLASTIDIC GLC TRANSLOCATOR); carbohydrate transmembra
Cit.25989.1.S1_at	122.545	262.6515	-2.14331	AT2G34190.1	2.00E-50	xanthine/uracil permease family protein
Cit.24324.1.S1_x_at	264.1488	566.4575	-2.14446	AT5G13930.1	3.00E-09	TT4 (TRANSPARENT TESTA 4); naringenin-chalcone synthase
Cit.22710.1.S1_at	843.4396	1810.292	-2.14632		NA	
Cit.33104.1.S1_at	24.44993	52.52674	-2.14834	AT5G62480.2	4.00E-12	ATGSTU9 (ARABIDOPSIS THALIANA GLUTATHIONE S-TRANSFERASE T
Cit.24753.1.S1_x_at	41.29613	88.78703	-2.15001		NA	
Cit.2831.1.S1_s_at	362.1465	779.1139	-2.15138	AT2G02100.1	3.00E-24	LCR69 (LOW-MOLECULAR-WEIGHT CYSTEINE-RICH 69); peptidase in
Cit.30930.1.S1_s_at	578.936	1248.615	-2.15674	AT5G52640.1	0	ATHSP90.1 (HEAT SHOCK PROTEIN 90.1); ATP binding / unfolded prc
Cit.22442.1.S1_at	21.80808	47.03695	-2.15686	AT1G35470.2	1.00E-51	SPla/RYanodine receptor (SPRY) domain-containing protein
Cit.25685.1.S1_at	33.15302	71.55582	-2.15835	AT5G25880.1	2.00E-32	ATNADP-ME3 (NADP-malic enzyme 3); malate dehydrogenase (oxal
Cit.9229.1.S1_x_at	224.1037	483.7724	-2.1587	AT4G34710.2	3.00E-44	ADC2 (ARGININE DECARBOXYLASE 2); arginine decarboxylase
Cit.24479.1.S1_at	41.61979	89.86327	-2.15915		NA	
Cit.38594.1.S1_at	22.79595	49.24656	-2.16032	AT3G42170.1	8.00E-05	DNA binding
Cit.29982.1.S1_at	85.61118	185.1553	-2.16275	AT3G11340.1	5.00E-74	UDP-glucoronosyl/UDP-glucosyl transferase family protein
Cit.13969.1.S1_s_at	116.1784	251.3461	-2.16345	AT5G52640.1	0	ATHSP90.1 (HEAT SHOCK PROTEIN 90.1); ATP binding / unfolded prc
Cit.17155.1.S1_s_at	120.403	260.8013	-2.16607	AT1G02850.2	1.00E-88	BGLU11 (BETA GLUCOSIDASE 11); hydrolase, hydrolyzing O-glycosyl

Cit.25696.1.S1_at	86.92068	188.298	-2.16632	AT4G13870.2	5.00E-09	WRNEXO (WERNER SYNDROME-LIKE EXONUCLEASE); 3'-5' exonucle
Cit.23371.1.S1_x_at	22.41478	48.55984	-2.16642	AT2G30860.1	9.00E-30	ATGSTF9 (GLUTATHIONE S-TRANSFERASE PHI 9); copper ion binding
Cit.22160.1.S1_x_at	134.6717	291.7957	-2.16672	AT1G27650.2	2.00E-55	ATU2AF35A; RNA binding / nucleic acid binding / nucleotide binding
Cit.29497.1.S1_at	24.33128	52.76692	-2.16869	AT2G38610.2	7.00E-67	KH domain-containing protein
Cit.37972.1.S1_at	306.1675	664.5767	-2.17063		NA	
Cit.38501.1.S1_at	34.62071	75.30125	-2.17503		NA	
Cit.37386.1.S1_at	32.19546	70.02776	-2.17508	AT3G44200.1	7.00E-22	NEK6 (NIMA (NEVER IN MITOSIS, GENE A)-RELATED 6); ATP binding ,
Cit.7434.1.S1_s_at	108.8819	237.0215	-2.17687	AT3G49080.1	1.00E-102	ribosomal protein S9 family protein
Cit.39645.1.S1_at	20.45775	44.54841	-2.17758	AT3G48750.1	9.00E-14	CDC2 (CELL DIVISION CONTROL 2); cyclin-dependent protein kinase/
Cit.6466.1.S1_at	21.64005	47.33508	-2.18738	AT2G22540.1	7.00E-76	SVP (SHORT VEGETATIVE PHASE); transcription factor/ translation re
Cit.24994.1.S1_at	326.3708	716.5255	-2.19543	AT5G10250.1	1.00E-50	DOT3 (DEFECTIVELY ORGANIZED TRIBUTARIES 3); protein binding / s
Cit.4067.1.S1_at	73.66167	161.846	-2.19715	AT3G59800.1	3.00E-72	unknown protein
Cit.29899.1.S1_at	20.62948	45.57794	-2.20936	AT3G07250.1	4.00E-33	nuclear transport factor 2 (NTF2) family protein / RNA recognition m
Cit.24560.1.S1_at	142.7725	315.6211	-2.21066		NA	
Cit.8612.1.S1_at	98.10734	217.3146	-2.21507	AT5G03370.1	2.00E-35	acylphosphatase family
Cit.9343.1.S1_x_at	28.08019	62.21158	-2.2155	AT1G11840.4	3.00E-90	ATGLX1 (GLYOXALASE I HOMOLOG); lactoylglutathione lyase/ metal
Cit.32235.1.S1_at	577.6802	1281.387	-2.21816	AT5G55520.2	1.00E-37	INVOLVED IN: biological_process unknown; EXPRESSED IN: 15 plant
Cit.34925.1.S1_at	82.15373	182.5676	-2.22227	AT5G24580.2	3.00E-20	copper-binding family protein
Cit.26693.1.S1_at	23.93289	53.20767	-2.2232	AT1G47500.1	3.00E-39	ATRBP47C' (RNA-binding protein 47C'); RNA binding
Cit.11911.1.S1_at	24.41752	54.30809	-2.22414	AT1G65450.1	1.00E-100	transferase family protein
Cit.3226.1.S1_at	31.15011	69.59489	-2.23418	AT1G65020.1	3.00E-75	FUNCTIONS IN: molecular_function unknown; INVOLVED IN: biologi
Cit.4159.1.S1_s_at	50.82398	113.577	-2.23471	AT1G74890.1	1.00E-55	ARR15 (RESPONSE REGULATOR 15); transcription regulator/ two-cor
Cit.26263.1.S1_at	57.97755	129.644	-2.23611	AT4G31940.1	2.00E-39	CYP82C4; electron carrier/ heme binding / iron ion binding / monoo
Cit.5818.1.S1_at	25.39884	56.98152	-2.24347	AT1G53708.1	9.00E-16	RTFL9 (ROTUNDIFOLIA LIKE 9)
Cit.16699.1.S1_at	646.3726	1456.828	-2.25385	AT3G51740.1	5.00E-29	IMK2 (INFLORESCENCE MERISTEM RECEPTOR-LIKE KINASE 2); ATP b
Cit.18687.1.S1_at	30.57332	69.08806	-2.25975	AT1G05680.1	3.00E-35	UDP-glucoronosyl/UDP-glucosyl transferase family protein
Cit.8866.1.S1_x_at	39.16576	88.79931	-2.26727	AT4G26850.1	0	VTC2 (vitamin c defective 2); GDP-D-glucose phosphorylase/ GDP-ga
Cit.17155.1.S1_at	235.4359	534.7839	-2.27146	AT1G02850.5	8.00E-38	BGLU11 (BETA GLUCOSIDASE 11); catalytic/ cation binding / hydrola
Cit.4377.1.S1_at	49.19728	111.7858	-2.27219	AT1G47480.1	8.00E-79	hydrolase
Cit.25928.1.S1_at	722.9603	1647.159	-2.27835		NA	
Cit.28694.1.S1_at	67.79538	154.5046	-2.27898	AT2G36830.1	1.00E-39	GAMMA-TIP (GAMMA TONOPLAST INTRINSIC PROTEIN); water char
Cit.37293.1.S1_at	21.62064	49.48685	-2.28887	AT5G35740.1	6.00E-46	glycosyl hydrolase family protein 17
Cit.29306.1.S1_at	36.02871	82.52762	-2.29061	AT5G55380.1	3.00E-33	membrane bound O-acyl transferase (MBOAT) family protein / wax
Cit.564.1.S1_at	24.67041	56.62623	-2.29531	AT3G48330.2	1.00E-35	PIMT1 (PROTEIN-L-ISOASPARTATE METHYLTRANSFERASE 1); protein
Cit.29766.1.S1_s_at	23.40748	53.80962	-2.29882	AT1G03220.1	1.00E-87	extracellular dermal glycoprotein, putative / EDGP, putative

Cit.32901.1.S1_at	20.71268	47.68092	-2.30202		NA	
Cit.35448.1.S1_at	23.28847	53.74765	-2.30791		NA	
Cit.24063.1.S1_at	27.83399	64.377	-2.31289	AT3G22440.1	3.00E-18	hydroxyproline-rich glycoprotein family protein
Cit.29074.1.S1_at	20.01985	46.36947	-2.31617		NA	
Cit.4192.1.S1_at	24.54309	56.89221	-2.31805	AT1G60200.1	1.00E-11	splicing factor PWI domain-containing protein / RNA recognition mo
Cit.15901.1.S1_at	20.45776	47.52147	-2.32291		NA	
Cit.32765.1.S1_at	74.605	173.3363	-2.32339	AT1G79790.1	1.00E-29	haloacid dehalogenase-like hydrolase family protein
Cit.13076.1.S1_at	45.86705	106.8533	-2.32963	AT1G78600.1	4.00E-83	LZF1 (LIGHT-REGULATED ZINC FINGER PROTEIN 1); transcription fact
Cit.29338.1.S1_x_at	54.64472	127.3176	-2.32992	AT3G22840.1	2.00E-14	ELIP1 (EARLY LIGHT-INDUCABLE PROTEIN); chlorophyll binding
Cit.5120.1.S1_at	52.40951	122.3248	-2.33402	AT1G74070.1	7.00E-95	peptidyl-prolyl cis-trans isomerase cyclophilin-type family protein
Cit.35664.1.S1_s_at	22.32804	52.1669	-2.33639	AT3G19440.1	1.00E-72	pseudouridine synthase family protein
Cit.31139.1.S1_at	71.24962	167.3575	-2.34889	AT5G11950.2	1.00E-100	protein homodimerization
Cit.37481.1.S1_at	74.88953	176.0519	-2.35082	AT3G15550.1	7.00E-79	unknown protein
Cit.30139.1.S1_at	191.2455	450.679	-2.35655	AT5G53870.1	2.00E-19	plastocyanin-like domain-containing protein
Cit.5139.1.S1_s_at	28.65775	67.74534	-2.36394	AT5G48560.1	4.00E-67	basic helix-loop-helix (bHLH) family protein
Cit.27820.1.S1_s_at	22.52364	53.28625	-2.36579	AT4G25140.1	5.00E-45	OLEO1 (OLEOSIN 1)
Cit.23162.1.S1_at	22.08317	52.25215	-2.36615	AT3G10150.1	2.00E-32	PAP16 (PURPLE ACID PHOSPHATASE 16); acid phosphatase/ protein
Cit.19893.1.S1_s_at	40.69079	96.50866	-2.37176	AT4G33090.1	1.00E-111	APM1 (AMINOPEPTIDASE M1); aminopeptidase
Cit.27651.1.S1_at	28.43217	67.44981	-2.37231		NA	
Cit.39387.1.S1_at	29.62634	70.33935	-2.37422	AT1G67750.1	1.00E-108	pectate lyase family protein
Cit.36656.1.S1_at	25.36083	60.21333	-2.37426	AT5G07310.1	1.00E-32	AP2 domain-containing transcription factor, putative
Cit.19813.1.S1_x_at	29.84251	70.85693	-2.37436	AT3G14470.1	9.00E-10	disease resistance protein (NBS-LRR class), putative
Cit.28604.1.S1_at	31.68892	75.33797	-2.37742		NA	
Cit.29275.1.S1_s_at	70.72484	168.7057	-2.38538	ATMG00030.1	8.00E-30	hypothetical protein
Cit.25369.1.S1_at	22.67821	54.10448	-2.38575	AT2G18360.1	9.00E-32	hydrolase, alpha/beta fold family protein
Cit.14741.1.S1_at	24.61126	58.78513	-2.38855	AT4G05320.4	7.00E-30	UBQ10 (POLYUBIQUITIN 10); protein binding
Cit.14716.1.S1_at	20.54776	49.22464	-2.39562	AT5G63220.1	1.00E-124	unknown protein
Cit.24046.1.S1_x_at	53.36663	128.2286	-2.40279	AT1G14900.1	5.00E-50	HMGA (HIGH MOBILITY GROUP A); DNA binding
Cit.10203.1.S1_x_at	35.35634	84.98469	-2.40366	AT2G42840.1	2.00E-41	PDF1 (PROTODERMAL FACTOR 1)
Cit.26152.1.S1_at	30.76341	74.16521	-2.41083		NA	
Cit.37298.1.S1_at	203.6541	491.6588	-2.41419	AT1G02730.1	2.00E-81	ATCSLD5; 1,4-beta-D-xylan synthase/ cellulose synthase
Cit.12938.1.S1_at	71.32961	172.2902	-2.41541	AT3G51670.1	0	SEC14 cytosolic factor family protein / phosphoglyceride transfer fa
Cit.40345.1.S1_at	29.00978	70.40177	-2.42683	AT2G16630.1	2.00E-53	proline-rich family protein
Cit.18734.1.S1_x_at	40.12617	97.44456	-2.42845		NA	
Cit.17682.1.S1_s_at	55.14114	134.1753	-2.43331	AT1G02840.3	1.00E-32	SR1; RNA binding / nucleic acid binding / nucleotide binding

Cit.8786.1.S1_at	49.37783	120.1707	-2.4337	AT1G05850.1	1.00E-150	POM1 (POM-POM1); chitinase
Cit.24694.1.S1_x_at	61.90144	150.6539	-2.43377	AT5G60530.1	7.00E-05	late embryogenesis abundant protein-related / LEA protein-related
Cit.16716.1.S1_at	35.4089	86.31446	-2.43765		NA	
Cit.7687.1.S1_at	37.45243	91.71809	-2.44892	AT1G05950.1	2.00E-07	unknown protein
Cit.25008.1.S1_at	22.49978	55.34796	-2.45993	AT3G14460.1	1.00E-14	disease resistance protein (NBS-LRR class), putative
Cit.24414.1.S1_at	54.2504	133.6166	-2.46296	AT5G38710.1	4.00E-36	proline oxidase, putative / osmotic stress-responsive proline dehydr
Cit.15021.1.S1_at	23.21465	57.39185	-2.47223	AT3G08040.2	6.00E-12	FRD3 (FERRIC REDUCTASE DEFECTIVE 3); antiporter/ transporter
Cit.23193.1.S1_x_at	33.56673	83.06615	-2.47466	AT3G46000.1	2.00E-26	ADF2 (ACTIN DEPOLYMERIZING FACTOR 2); actin binding
Cit.29123.1.S1_at	102.3263	253.3124	-2.47554		NA	
Cit.25441.1.S1_x_at	31.59947	78.52357	-2.48496	AT1G60690.1	1.00E-116	aldo/keto reductase family protein
Cit.30163.1.S1_at	76.70155	190.9701	-2.48978	AT1G72240.1	4.00E-12	unknown protein
Cit.27966.1.S1_s_at	29.14062	72.91695	-2.50224	AT4G36360.2	1.00E-90	BGAL3 (beta-galactosidase 3); beta-galactosidase/ catalytic/ cation b
Cit.29563.1.S1_at	24.83207	62.34853	-2.51081	AT2G44600.1	6.00E-56	unknown protein
Cit.13425.1.S1_at	64.15006	161.4252	-2.51637	AT2G39730.1	1.00E-169	RCA (RUBISCO ACTIVASE); ADP binding / ATP binding / enzyme regu
Cit.28642.1.S1_x_at	20.52863	51.83931	-2.52522		NA	
Cit.4145.1.S1_at	22.8169	57.63128	-2.52582	AT1G43800.1	1.00E-166	acyl-(acyl-carrier-protein) desaturase, putative / stearoyl-ACP desat
Cit.656.1.S1_at	31.80088	80.57368	-2.53369	AT3G08900.1	0	RGP3 (REVERSIBLY GLYCOSYLATED POLYPEPTIDE 3); transferase, tra
Cit.12593.1.S1_at	29.38227	74.51751	-2.53614	AT1G52560.1	4.00E-73	26.5 kDa class I small heat shock protein-like (HSP26.5-P)
Cit.23895.1.S1_at	23.68126	60.13485	-2.53934		NA	
Cit.28672.1.S1_at	26.72485	67.99748	-2.54435		NA	
Cit.28045.1.S1_at	60.79308	155.157	-2.55221	AT5G16620.1	2.00E-61	TIC40
Cit.6536.1.S1_at	47.5833	121.5971	-2.55546	AT4G03320.1	7.00E-68	tic20-IV (TRANSLOCON AT THE INNER ENVELOPE MEMBRANE OF CH
Cit.22077.1.S1_at	41.40157	106.0283	-2.56097		NA	
Cit.22748.1.S1_x_at	97.05338	250.2059	-2.57802	AT2G29250.1	3.00E-11	lectin protein kinase, putative
Cit.7212.1.S1_at	20.2835	52.29544	-2.57823		NA	
Cit.28447.1.S1_x_at	31.44688	81.10466	-2.5791	AT5G14200.1	3.00E-78	3-isopropylmalate dehydrogenase, chloroplast, putative
Cit.17596.1.S1_x_at	26.73581	69.05882	-2.58301	AT3G22120.1	1.00E-29	CWLP (CELL WALL-PLASMA MEMBRANE LINKER PROTEIN); lipid binc
Cit.4341.1.S1_at	20.71408	53.77438	-2.59603	AT5G58560.1	2.00E-93	phosphatidate cytidylyltransferase family protein
Cit.17489.1.S1_at	1060.389	2754.278	-2.59742	ATCG00800.1	7.00E-26	encodes a chloroplast ribosomal protein S3, a constituent of the sm
Cit.38876.1.S1_at	38.604	100.3388	-2.59918	AT3G51930.1	2.00E-24	transducin family protein / WD-40 repeat family protein
Cit.21266.1.S1_at	28.36307	73.74278	-2.59996		NA	
Cit.30834.1.S1_at	29.68092	77.61348	-2.61493	AT1G20780.1	3.00E-10	SAUL1 (SENESCENCE-ASSOCIATED E3 UBIQUITIN LIGASE 1); ubiquitir
Cit.27915.1.S1_at	21.16217	55.52611	-2.62384	AT1G17020.1	2.00E-06	SRG1 (SENESCENCE-RELATED GENE 1); oxidoreductase, acting on dip
Cit.28590.1.S1_at	48.49009	128.2944	-2.64579	AT1G76140.1	1.00E-104	serine-type endopeptidase/ serine-type peptidase
Cit.16635.1.S1_at	26.99416	71.44223	-2.64658	AT3G47180.1	5.00E-42	zinc finger (C3HC4-type RING finger) family protein

Cit.22993.1.S1_x_at	23.59061	62.51579	-2.65003		NA	
Cit.3500.1.S1_s_at	40.81806	108.6332	-2.6614	AT5G04500.1	1.00E-05	glycosyltransferase family protein 47
Cit.7302.1.S1_at	26.71582	71.548	-2.67811	AT2G01818.1	2.00E-55	zinc ion binding
Cit.22228.1.S1_at	30.85639	83.06625	-2.69203	AT1G28330.4	6.00E-21	DYL1 (DORMANCY-ASSOCIATED PROTEIN-LIKE 1)
Cit.3465.1.S1_at	25.62672	69.60511	-2.71611	AT1G16520.1	7.00E-96	unknown protein
Cit.542.1.S1_x_at	25.59425	70.08566	-2.73834	AT3G49540.1	1.00E-08	unknown protein
Cit.31350.1.S1_at	24.23538	66.62836	-2.74922	AT1G71810.1	4.00E-47	ABC1 family protein
Cit.18672.1.S1_at	27.54852	76.37849	-2.77251		NA	
Cit.36807.1.S1_s_at	28.9975	80.81519	-2.78697	AT4G21200.1	2.00E-74	GA2OX8 (GIBBERELLIN 2-OXIDASE 8); gibberellin 2-beta-dioxygenas
Cit.28237.1.S1_at	77.40388	216.0526	-2.79124		NA	
Cit.31216.1.S1_at	98.06509	274.5667	-2.79984	AT5G38020.1	5.00E-83	S-adenosyl-L-methionine:carboxyl methyltransferase family protein
Cit.18584.1.S1_s_at	24.28951	68.40508	-2.81624	AT4G24290.2	3.00E-37	FUNCTIONS IN: molecular_function unknown; INVOLVED IN: biologi
Cit.25808.1.S1_at	43.51011	124.4732	-2.86079		NA	
Cit.33092.1.S1_at	21.1606	60.79101	-2.87284	AT3G05330.1	1.00E-19	ATN (TANGLED)
Cit.25660.1.S1_x_at	27.24119	78.46193	-2.88027		NA	
Cit.10630.1.S1_at	145.3319	420.8383	-2.8957		NA	
Cit.542.1.S1_at	64.2382	186.4335	-2.90222		NA	
Cit.17523.1.S1_at	38.01758	110.5561	-2.90803	AT1G06475.1	4.00E-06	unknown protein
Cit.2694.1.S1_at	68.43862	199.104	-2.90923	AT3G25810.1	1.00E-142	myrcene/ocimene synthase, putative
Cit.7567.1.S1_at	20.37458	60.48399	-2.9686		NA	
Cit.16701.1.S1_at	47.97202	142.949	-2.97984		NA	
Cit.26708.1.S1_at	52.8608	157.6562	-2.98248	AT1G74350.1	2.00E-52	intron maturase, type II family protein
Cit.6415.1.S1_at	31.00619	92.50906	-2.98357	AT5G59730.1	1.00E-54	ATEXO70H7 (EXOCYST SUBUNIT EXO70 FAMILY PROTEIN H7); protei
Cit.38372.1.S1_at	25.74865	77.47055	-3.00872	AT2G29510.1	4.00E-15	unknown protein
Cit.26228.1.S1_at	20.09894	60.86145	-3.02809	AT1G75580.1	2.00E-37	auxin-responsive protein, putative
Cit.22380.1.S1_at	20.21744	61.34342	-3.03418	AT1G03140.1	4.00E-66	splicing factor Prp18 family protein
Cit.1802.1.S1_at	73.92919	224.6847	-3.03919	AT4G12590.1	1.00E-124	unknown protein
Cit.22726.1.S1_x_at	45.79843	144.1086	-3.14658		NA	
Cit.5017.1.S1_at	129.8159	412.7237	-3.1793	AT1G32080.1	0	membrane protein, putative
Cit.2905.1.S1_at	30.54871	97.29784	-3.18501	AT1G31330.1	5.00E-96	PSAF (photosystem I subunit F)
Cit.25802.1.S1_at	32.4248	104.4235	-3.22048		NA	
Cit.30283.1.S1_at	22.94553	74.9254	-3.26536	AT4G18390.2	2.00E-16	TCP family transcription factor, putative
Cit.30648.1.S1_s_at	405.2241	1341.322	-3.31007	AT4G27670.1	2.00E-32	HSP21 (HEAT SHOCK PROTEIN 21)
Cit.15073.1.S1_at	50.22567	166.4631	-3.3143	AT2G03200.1	6.00E-48	aspartyl protease family protein
Cit.29072.1.S1_at	182.3672	605.5111	-3.32029	AT1G56720.3	4.00E-31	protein kinase family protein

Cit.21774.1.S1_at	22.62618	76.00327	-3.35909	AT1G52560.2	7.00E-06	26.5 kDa class I small heat shock protein-like (HSP26.5-P)
Cit.38304.1.S1_at	27.6371	93.73337	-3.39158	AT1G72900.1	3.00E-25	disease resistance protein (TIR-NBS class), putative
Cit.28815.1.S1_x_at	28.23388	95.92066	-3.39736		NA	
Cit.17650.1.S1_x_at	58.43742	200.5676	-3.43218	AT3G05540.1	1.00E-67	INVOLVED IN: biological_process unknown; LOCATED IN: cytoplasm,
Cit.23637.1.S1_x_at	52.37453	181.3423	-3.46241		NA	
Cit.23697.1.S1_at	29.6914	104.8769	-3.53223	AT4G16390.1	1.00E-19	LOCATED IN: chloroplast; EXPRESSED IN: 22 plant structures; EXPRES
Cit.26433.1.S1_s_at	38.74042	137.1494	-3.54021	AT1G52560.1	4.00E-73	26.5 kDa class I small heat shock protein-like (HSP26.5-P)
Cit.10304.1.S1_x_at	228.7246	812.6461	-3.55295	AT3G58780.2	7.00E-07	SHP1 (SHATTERPROOF 1); DNA binding / protein binding / transcript
Cit.28815.1.S1_at	33.17456	119.9051	-3.61437		NA	
Cit.5512.1.S1_at	30.9966	113.1684	-3.65099	AT1G12030.1	7.00E-56	unknown protein
Cit.15971.1.S1_at	106.704	390.988	-3.66423		NA	
Cit.23133.1.S1_x_at	29.18202	108.1088	-3.70464	AT4G39630.1	3.00E-14	unknown protein
Cit.17596.1.S1_s_at	488.3468	1886.041	-3.86209		NA	
Cit.15637.1.S1_at	30.44462	119.3001	-3.91859	AT4G28780.1	9.00E-91	GDSL-motif lipase/hydrolase family protein
Cit.25271.1.S1_at	23.50817	93.89017	-3.99394		NA	
Cit.9873.1.S1_at	42.66396	174.3428	-4.08642	AT4G32330.3	4.00E-12	FUNCTIONS IN: molecular_function unknown; INVOLVED IN: biologi
Cit.25990.1.S1_at	551.7408	2301.747	-4.17179	AT1G49640.1	5.00E-06	hydrolase
Cit.25990.1.S1_x_at	901.1877	3784.191	-4.19912	AT1G49640.1	5.00E-06	hydrolase
Cit.28011.1.S1_x_at	677.771	3158.526	-4.66017	AT5G47550.1	4.00E-25	cysteine protease inhibitor, putative / cystatin, putative
Cit.30421.1.S1_x_at	31.04203	145.5358	-4.68835	AT5G47550.1	7.00E-27	cysteine protease inhibitor, putative / cystatin, putative
Cit.6467.1.S1_s_at	101.6089	495.165	-4.87324		NA	
Cit.30421.1.S1_s_at	766.525	3802.129	-4.96022	AT5G47550.1	7.00E-27	cysteine protease inhibitor, putative / cystatin, putative
Cit.20946.1.S1_at	26.02087	138.583	-5.32584	AT5G43050.1	8.00E-53	unknown protein
Cit.8163.1.S1_x_at	1345.842	7381.447	-5.48463	AT5G47550.1	2.00E-23	cysteine protease inhibitor, putative / cystatin, putative
Cit.13787.1.S1_s_at	45.81779	318.2433	-6.94585	AT2G03200.1	1.00E-50	aspartyl protease family protein
Cit.18430.1.S1_s_at	26.63369	224.3055	-8.42187		NA	
Cit.26140.1.S1_at	21.11017	357.0902	-16.9156		NA	

Supplementary Table S8. PDTA in seedless vs. seedy pineapple fruits at time point 2.

ProbeSet ID	Pineapple_ Seedless	Pineapple_ seedy	Ratio	AtGID	E-Score	Arabidopsis.annotation
Cit.11918.1.S1_at	475.1068	25.65893	18.51624	AT4G38540.1	2.00E-78	monooxygenase, putative (MO2)
Cit.25990.1.S1_at	2395.499	143.4879	16.69478	AT1G49640.1	5.00E-06	hydrolase
Cit.17596.1.S1_s_at	1638.226	100.8626	16.24216		NA	
Cit.10894.1.S1_s_at	1921.151	137.6414	13.95765	AT5G06760.1	1.00E-48	late embryogenesis abundant group 1 domain-containin
Cit.574.1.S1_at	1169.324	102.4943	11.40867		NA	
Cit.5970.1.S1_at	334.2915	29.79106	11.2212	AT5G61430.1	6.00E-92	ANAC100 (ARABIDOPSIS NAC DOMAIN CONTAINING PRO
Cit.25990.1.S1_x_at	3066.185	288.9	10.61331	AT1G49640.1	5.00E-06	hydrolase
Cit.39178.1.S1_s_at	318.0051	35.52937	8.950485	AT5G06760.1	1.00E-48	late embryogenesis abundant group 1 domain-containin
Cit.12433.1.S1_at	313.7657	40.63492	7.721578		NA	
Cit.10895.1.S1_at	322.9709	46.39428	6.961438	AT5G06760.1	1.00E-48	late embryogenesis abundant group 1 domain-containin
Cit.18556.1.S1_at	293.2265	47.2162	6.210294		NA	
Cit.4047.1.S1_at	133.0166	21.75221	6.115084	AT2G02990.1	7.00E-96	RNS1 (RIBONUCLEASE 1); endoribonuclease/ ribonucleas
Cit.29688.1.S1_s_at	127.9656	21.56618	5.933624	AT3G61890.1	5.00E-40	ATHB-12 (ARABIDOPSIS THALIANA HOMEOBOX 12); tran
Cit.8697.1.S1_at	1043.771	184.0545	5.670989		NA	
Cit.2906.1.S1_s_at	131.8376	26.43429	4.987371		NA	
Cit.25191.1.S1_s_at	128.7247	26.1733	4.918169	AT5G10770.1	1.00E-160	chloroplast nucleoid DNA-binding protein, putative
Cit.25722.1.S1_at	545.8334	114.401	4.771229	AT1G75230.2	1.00E-62	HhH-GPD base excision DNA repair family protein
Cit.18116.1.S1_at	176.8989	37.38572	4.731724	AT5G48850.1	3.00E-39	ATSDI1 (SULPHUR DEFICIENCY-INDUCED 1); binding
Cit.2121.1.S1_at	108.8804	23.88132	4.559229	AT5G40670.1	1.00E-64	PQ-loop repeat family protein / transmembrane family p
Cit.8320.1.S1_at	124.8605	28.37326	4.40064	AT1G74270.1	4.00E-50	60S ribosomal protein L35a (RPL35aC)
Cit.17413.1.S1_s_at	114.7553	27.96323	4.103793	AT4G33467.2	1.00E-09	unknown protein
Cit.29507.1.S1_s_at	285.0354	70.28761	4.055272		NA	
Cit.881.1.S1_s_at	122.6996	30.52255	4.019966	AT3G55240.1	4.00E-36	Overexpression leads to PEL (Pseudo-Etiolation in Light)
Cit.21579.1.S1_at	203.9776	52.32351	3.898393	AT2G18328.1	4.00E-09	ATRL4 (ARABIDOPSIS RAD-LIKE 4); DNA binding
Cit.2729.1.S1_at	214.1733	56.67706	3.778836	AT3G12760.1	1.00E-116	FUNCTIONS IN: molecular_function unknown; INVOLVED
Cit.28253.1.S1_at	76.22656	21.45031	3.553634	AT3G26220.1	9.00E-54	CYP71B3; electron carrier/ heme binding / iron ion bindi
Cit.9388.1.S1_at	84.73486	23.99307	3.531639	AT5G61820.1	7.00E-24	FUNCTIONS IN: molecular_function unknown; INVOLVED
Cit.1200.1.S1_s_at	321.8669	91.80767	3.505882	AT4G11650.1	9.00E-81	ATOSM34 (osmotin 34)
Cit.20412.1.S1_s_at	123.3782	36.31043	3.397872	AT3G12500.1	1.00E-127	ATHCHIB (ARABIDOPSIS THALIANA BASIC CHITINASE); ch
Cit.35483.1.S1_at	156.5875	46.14051	3.39371	AT5G27395.2	4.00E-27	P-P-bond-hydrolysis-driven protein transmembrane tran
Cit.1496.1.S1_s_at	233.7841	70.96974	3.294138	AT3G04070.1	1.00E-98	anac047 (Arabidopsis NAC domain containing protein 47

Cit.6466.1.S1_at	66.15385	20.14177	3.284411	AT2G22540.1	7.00E-76	SVP (SHORT VEGETATIVE PHASE); transcription factor/ tr
Cit.18120.1.S1_at	78.09386	24.06103	3.245657	AT5G56550.1	2.00E-21	OXS3 (OXIDATIVE STRESS 3)
Cit.34286.1.S1_at	76.70128	23.65786	3.242106		NA	
Cit.2569.1.S1_at	167.7551	51.83218	3.236505	AT1G80670.1	5.00E-48	transducin family protein / WD-40 repeat family protein
Cit.18491.1.S1_at	789.6982	250.1211	3.157263		NA	
Cit.3556.1.S1_at	91.45204	29.40551	3.110031	AT1G69740.2	7.00E-99	HEMB1; catalytic/ metal ion binding / porphobilinogen s
Cit.1497.1.S1_s_at	426.192	137.9531	3.089398	AT3G04070.1	1.00E-98	anac047 (Arabidopsis NAC domain containing protein 47
Cit.22710.1.S1_x_at	261.3826	87.14124	2.999528	AT4G11650.1	9.00E-25	ATOSM34 (osmotin 34)
Cit.25808.1.S1_at	107.7797	35.9524	2.997844		NA	
Cit.8288.1.S1_at	64.53888	21.67042	2.978202	AT5G11870.1	1.00E-66	FUNCTIONS IN: molecular_function unknown; INVOLVED
Cit.6849.1.S1_at	89.37884	30.04689	2.974645	AT5G41800.1	1.00E-65	amino acid transporter family protein
Cit.17394.1.S1_s_at	358.4572	120.9744	2.963083		NA	
Cit.9301.1.S1_s_at	151.1555	51.23121	2.950457	AT5G06570.2	3.00E-47	hydrolase
Cit.11945.1.S1_at	66.36211	22.49362	2.950264	AT1G48830.2	6.00E-87	40S ribosomal protein S7 (RPS7A)
Cit.3086.1.S1_at	158.1223	53.78384	2.939959	AT1G68320.1	2.00E-63	MYB62 (myb domain protein 62); DNA binding / transcri
Cit.4030.1.S1_at	180.8266	61.63254	2.933947	AT1G60190.1	4.00E-82	armadillo/beta-catenin repeat family protein / U-box do
Cit.24558.1.S1_at	63.63449	21.87304	2.909266	AT1G43190.2	4.00E-05	polypyrimidine tract-binding protein, putative / heteroge
Cit.18339.1.S1_at	85.23251	29.36888	2.902137		NA	
Cit.24645.1.S1_at	66.75318	23.12349	2.886813		NA	
Cit.31307.1.S1_at	82.07581	28.51284	2.878556		NA	
Cit.37918.1.S1_at	60.44016	21.24633	2.844734	AT2G38940.1	3.00E-52	ATPT2 (ARABIDOPSIS THALIANA PHOSPHATE TRANSPOR
Cit.12090.1.S1_at	57.3109	20.23727	2.831948		NA	
Cit.16028.1.S1_at	74.04913	26.20845	2.825391	AT4G31080.1	2.00E-37	unknown protein
Cit.27090.1.S1_at	86.79099	30.75241	2.82225	AT5G13490.2	7.00E-19	AAC2 (ADP/ATP carrier 2); ATP:ADP antiporter/ binding
Cit.20200.1.S1_at	106.7616	37.86829	2.819288	AT4G05320.4	2.00E-27	UBQ10 (POLYUBIQUITIN 10); protein binding
Cit.14567.1.S1_at	69.70071	24.79434	2.811154	AT1G01630.1	9.00E-93	SEC14 cytosolic factor, putative / phosphoglyceride trans
Cit.29907.1.S1_at	602.7917	215.8069	2.793199		NA	
Cit.17090.1.S1_s_at	88.69033	31.83172	2.786225	AT1G69490.1	4.00E-89	NAP (NAC-like, activated by AP3/PI); transcription factor
Cit.13579.1.S1_s_at	139.9123	50.21919	2.786033	AT5G65660.1	3.00E-48	hydroxyproline-rich glycoprotein family protein
Cit.17650.1.S1_x_at	348.4521	125.3443	2.77996	AT3G05540.1	1.00E-67	INVOLVED IN: biological_process unknown; LOCATED IN
Cit.21908.1.S1_x_at	67.0601	24.13775	2.778225		NA	
Cit.28072.1.S1_at	63.96364	23.18086	2.75933	AT5G42800.1	1.00E-05	DFR (DIHYDROFLAVONOL 4-REDUCTASE); dihydrokaemp
Cit.14989.1.S1_at	86.87534	31.6294	2.746664	AT2G16790.1	6.00E-56	shikimate kinase family protein
Cit.35536.1.S1_at	61.11246	22.27888	2.743067	AT1G62050.1	1.00E-126	FUNCTIONS IN: molecular_function unknown; INVOLVED
Cit.25381.1.S1_at	63.40878	23.16319	2.73748	AT1G29820.2	1.00E-105	unknown protein

Cit.30695.1.S1_s_at	418.109	152.7498	2.737215	AT3G21420.1	1.00E-151	oxidoreductase, 2OG-Fe(II) oxygenase family protein
Cit.580.1.S1_x_at	299.0221	109.2978	2.735847	AT3G04720.1	9.00E-47	PR4 (PATHOGENESIS-RELATED 4); chitin binding
Cit.6026.1.S1_at	105.7452	39.14219	2.701566	AT1G65420.1	5.00E-56	unknown protein
Cit.24967.1.S1_at	87.82141	32.52933	2.699761	AT3G04730.1	4.00E-17	IAA16; transcription factor
Cit.6823.1.S1_at	74.28925	27.66909	2.684918		NA	
Cit.7498.1.S1_at	65.04476	24.26862	2.6802	AT5G22120.1	3.00E-17	unknown protein
Cit.29940.1.S1_at	140.4575	52.44065	2.678409	AT5G57050.2	1.00E-119	ABI2 (ABA INSENSITIVE 2); protein serine/threonine pho
Cit.1854.1.S1_at	119.7887	44.93931	2.665566		NA	
Cit.14999.1.S1_s_at	175.4052	65.97571	2.658633	AT4G03500.1	1.00E-33	ankyrin repeat family protein
Cit.14998.1.S1_at	102.6122	38.95502	2.63412	AT4G03500.1	1.00E-33	ankyrin repeat family protein
Cit.37143.1.S1_at	63.13773	23.9721	2.633801	AT5G17800.1	4.00E-50	AtMYB56 (myb domain protein 56); DNA binding / trans
Cit.32010.1.S1_at	80.8037	30.76586	2.626408		NA	
Cit.22170.1.S1_x_at	53.52656	20.57315	2.601768	AT5G37990.1	3.00E-12	S-adenosylmethionine-dependent methyltransferase/ m
Cit.3036.1.S1_s_at	681.3484	263.2741	2.587981	AT3G04920.1	9.00E-63	40S ribosomal protein S24 (RPS24A)
Cit.5269.1.S1_at	70.2355	27.16829	2.585201		NA	
Cit.25991.1.S1_at	58.44072	23.00673	2.540158		NA	
Cit.30560.1.S1_at	93.36507	36.93051	2.528128	AT1G32928.1	7.00E-08	unknown protein
Cit.36687.1.S1_at	55.84477	22.24065	2.510932	AT5G61580.1	6.00E-24	PFK4 (PHOSPHOFRUCTOKINASE 4); 6-phosphofructokina
Cit.17018.1.S1_s_at	316.8836	127.5894	2.48362	AT1G23040.1	3.00E-31	hydroxyproline-rich glycoprotein family protein
Cit.37269.1.S1_at	123.1487	49.66241	2.479717		NA	
Cit.32792.1.S1_at	54.57	22.09604	2.469673		NA	
Cit.38075.1.S1_at	57.69498	23.50269	2.454825	AT5G62090.2	1.00E-08	FUNCTIONS IN: molecular_function unknown; INVOLVED
Cit.6661.1.S1_at	49.12143	20.01191	2.45461		NA	
Cit.2555.1.S1_at	1046.791	428.1999	2.444632	AT1G48300.1	2.00E-45	unknown protein
Cit.22978.1.S1_s_at	72.14133	29.51279	2.444409	AT1G58030.1	6.00E-24	CAT2 (CATIONIC AMINO ACID TRANSPORTER 2); amino a
Cit.29608.1.S1_at	1308.272	535.4637	2.443251		NA	
Cit.29338.1.S1_x_at	77.50691	31.78181	2.438719	AT3G22840.1	2.00E-14	ELIP1 (EARLY LIGHT-INDUCABLE PROTEIN); chlorophyll b
Cit.24055.1.S1_at	136.2595	56.00528	2.432976	AT3G14460.1	4.00E-27	disease resistance protein (NBS-LRR class), putative
Cit.5398.1.S1_at	102.9541	42.33442	2.431924	AT4G01280.1	4.00E-29	myb family transcription factor
Cit.2274.1.S1_at	66.77937	27.56469	2.422642	AT2G29980.1	1.00E-170	FAD3 (FATTY ACID DESATURASE 3); omega-3 fatty acid d
Cit.19102.1.S1_at	51.13533	21.11615	2.421622	AT2G19470.1	7.00E-10	ckl5 (Casein Kinase I-like 5); ATP binding / kinase/ protei
Cit.22883.1.S1_x_at	88.09943	36.38392	2.421384		NA	
Cit.36667.1.S1_at	63.06919	26.09948	2.416492	AT5G51290.1	2.00E-56	ceramide kinase-related
Cit.9009.1.S1_at	100.3976	41.65356	2.410301	AT2G43970.1	8.00E-80	La domain-containing protein
RandomGC24_at	118.1495	49.05272	2.408623	NA	NA	NA

Cit.24995.1.S1_at	844.9803	352.4571	2.397399	AT1G04770.1	3.00E-38	male sterility MS5 family protein
Cit.23168.1.S1_at	87.09133	36.32926	2.397278		NA	
Cit.9300.1.S1_s_at	234.9982	98.21793	2.39262	AT3G28860.1	7.00E-92	ABCB19; ATPase, coupled to transmembrane movement
Cit.16640.1.S1_at	283.6043	118.6638	2.389982	AT1G55830.1	8.00E-49	unknown protein
Cit.19942.1.S1_x_at	49.85452	20.87658	2.38806		NA	
Cit.3549.1.S1_at	560.7376	234.8317	2.387828	AT3G12360.1	9.00E-55	ITN1 (INCREASED TOLERANCE TO NACL); protein binding
Cit.12693.1.S1_at	741.1217	310.4478	2.387267	AT4G17370.1	1.00E-122	oxidoreductase family protein
Cit.29416.1.S1_at	256.6868	107.7882	2.3814	AT5G14000.1	9.00E-42	anac084 (Arabidopsis NAC domain containing protein 84
Cit.38403.1.S1_at	50.68764	21.33724	2.375548		NA	
Cit.30690.1.S1_at	48.17587	20.3242	2.37037	AT1G54260.1	7.00E-06	histone H1/H5 family protein
Cit.14472.1.S1_s_at	363.8142	153.691	2.36718		NA	
Cit.5963.1.S1_at	94.24671	39.89577	2.362323	AT5G07120.1	1.00E-108	SNX2b (SORTING NEXIN 2b); phosphoinositide binding /
Cit.7817.1.S1_at	77.15886	32.73524	2.357058	AT1G72190.1	9.00E-41	oxidoreductase family protein
Cit.35849.1.S1_at	68.11685	28.92966	2.354568	AT3G04260.1	9.00E-79	PTAC3 (PLASTID TRANSCRIPTIONALLY ACTIVE3); DNA bir
Cit.11760.1.S1_a_at	63.45186	26.96031	2.353529	AT1G52540.1	1.00E-132	protein kinase, putative
Cit.16289.1.S1_at	50.03186	21.27087	2.35213		NA	
Cit.7108.1.S1_at	81.63784	34.71804	2.351453		NA	
Cit.13914.1.S1_at	129.2922	55.11875	2.345703	AT1G06220.1	1.00E-157	MEE5 (MATERNAL EFFECT EMBRYO ARREST 5); GTP bind
Cit.32970.1.S1_at	47.60039	20.35489	2.338524	AT1G71696.2	2.00E-12	SOL1 (SUPPRESSOR OF LLP1 1); carboxypeptidase/ meta
Cit.3395.1.S1_at	266.5458	114.0081	2.337955	AT5G15270.1	3.00E-15	KH domain-containing protein
Cit.16993.1.S1_s_at	64.59454	27.71442	2.33072	AT3G55240.1	4.00E-36	Overexpression leads to PEL (Pseudo-Etiolation in Light)
Cit.38208.1.S1_at	46.34829	20.15157	2.299984		NA	
Cit.8206.1.S1_s_at	164.8341	71.87648	2.293297	AT3G45140.1	1.00E-109	LOX2 (LIPOXYGENASE 2); lipoxygenase
Cit.1877.1.S1_at	126.4111	55.13253	2.292859		NA	
Cit.22755.1.S1_at	775.6359	338.9506	2.288345	AT5G62530.1	6.00E-83	ALDH12A1; 1-pyrroline-5-carboxylate dehydrogenase/ 3
Cit.4253.1.S1_at	129.2976	56.56649	2.285763		NA	
Cit.25548.1.S1_at	46.16645	20.2106	2.284269	AT1G52760.1	2.00E-09	esterase/lipase/thioesterase family protein
Cit.24444.1.S1_x_at	55.38763	24.2821	2.281007		NA	
Cit.26643.1.S1_at	61.09171	26.78686	2.28066	AT3G14460.1	2.00E-15	disease resistance protein (NBS-LRR class), putative
Cit.31135.1.S1_at	55.2723	24.23923	2.280283	AT1G70610.1	9.00E-45	ATTAP1; ATPase, coupled to transmembrane movement
Cit.19161.1.S1_x_at	67.37305	29.54605	2.280273	AT4G19040.2	8.00E-59	EDR2; lipid binding
Cit.23152.1.S1_at	55.22165	24.32759	2.269919	AT1G19310.1	3.00E-26	zinc finger (C3HC4-type RING finger) family protein
Cit.12150.1.S1_at	103.4773	45.59881	2.269298	AT1G22170.1	6.00E-16	phosphoglycerate/bisphosphoglycerate mutase family p
Cit.39480.1.S1_at	52.27525	23.0674	2.266196	AT5G51460.1	2.00E-05	ATTPPA; trehalose-phosphatase
Cit.32260.1.S1_at	113.096	49.93909	2.264679	AT5G14360.1	2.00E-40	ubiquitin family protein

Cit.13417.1.S1_s_at	261.5317	115.7562	2.259332	AT1G08650.1	4.00E-77	PPCK1 (PHOSPHOENOLPYRUVATE CARBOXYLASE KINASE
Cit.23058.1.S1_x_at	53.98092	23.9982	2.249374	AT2G19600.1	1.00E-21	ATKEA4; potassium ion transmembrane transporter/ pot
Cit.3195.1.S1_at	63.16478	28.15878	2.243165	AT1G33055.1	7.00E-13	unknown protein
Cit.12918.1.S1_at	48.45043	21.66669	2.236171	AT4G38630.1	1.00E-149	RPN10 (REGULATORY PARTICLE NON-ATPASE 10); peptic
Cit.18861.1.S1_x_at	2082.201	935.458	2.225863		NA	
Cit.33343.1.S1_s_at	483.0837	218.0829	2.215138	AT5G13750.2	4.00E-12	ZIFL1 (ZINC INDUCED FACILITATOR-like 1); tetracycline:h
Cit.37023.1.S1_at	54.53873	24.63131	2.214203	AT1G51580.1	6.00E-12	KH domain-containing protein
Cit.12777.1.S1_at	1634.059	740.4543	2.206833	AT4G36850.1	1.00E-143	INVOLVED IN: biological_process unknown; LOCATED IN
Cit.7715.1.S1_at	74.53566	33.78731	2.206025		NA	
Cit.4997.1.S1_at	295.3919	133.954	2.205174	AT4G31890.1	7.00E-48	armadillo/beta-catenin repeat family protein
Cit.20801.1.S1_at	129.646	58.83901	2.203402	AT3G61250.1	2.00E-05	AtMYB17 (myb domain protein 17); DNA binding / trans
Cit.30294.1.S1_at	45.23684	20.58964	2.197068	AT4G25970.1	5.00E-67	PSD3 (phosphatidylserine decarboxylase 3); phosphatidy
Cit.10033.1.S1_x_at	186.599	84.97482	2.195933	AT1G75750.1	2.00E-36	GASA1 (GAST1 PROTEIN HOMOLOG 1)
Cit.25471.1.S1_at	55.21043	25.14587	2.195606	AT1G78000.2	9.00E-96	SULTR1;2 (SULFATE TRANSPORTER 1;2); sulfate transme
Cit.11107.1.S1_at	49.23838	22.46898	2.191394	AT3G25290.2	1.00E-142	auxin-responsive family protein
Cit.5201.1.S1_at	110.3577	50.45114	2.187417	AT2G14960.1	0	GH3.1
Cit.23989.1.S1_at	65.6365	30.01542	2.186759	AT2G05210.3	5.00E-16	AtPOT1a (Protection of Telomeres 1a); telomeric DNA bi
Cit.21392.1.S1_at	361.4599	165.6158	2.182521		NA	
Cit.20415.1.S1_x_at	47.6053	21.81549	2.182179		NA	
Cit.26242.1.S1_s_at	64.2927	29.52675	2.177439	AT3G17910.1	1.00E-113	SURF1 (SURFEIT 1)
Cit.21988.1.S1_s_at	405.675	186.913	2.170395	AT5G20410.1	8.00E-74	MGD2; 1,2-diacylglycerol 3-beta-galactosyltransferase/ U
Cit.24698.1.S1_x_at	145.6371	67.23471	2.1661		NA	
Cit.7311.1.S1_at	91.68704	42.35651	2.16465	AT3G16785.1	1.00E-157	PLDP1 (PHOSPHOLIPASE D P1); phospholipase D
Cit.31377.1.S1_at	129.8263	60.13199	2.159022	AT2G17040.1	2.00E-82	anac036 (Arabidopsis NAC domain containing protein 36
Cit.302.1.S1_s_at	84.78887	39.3489	2.154796	AT3G12500.1	1.00E-127	ATHCHIB (ARABIDOPSIS THALIANA BASIC CHITINASE); ch
Cit.15652.1.S1_at	77.10267	35.82879	2.151975	AT2G34450.2	3.00E-10	high mobility group (HMG1/2) family protein
Cit.17675.1.S1_at	235.0795	109.3085	2.150606	AT1G26250.1	2.00E-29	proline-rich extensin, putative
Cit.28188.1.S1_at	65.56256	30.50064	2.149547	AT3G17380.1	7.00E-08	meprin and TRAF homology domain-containing protein /
Cit.9373.1.S1_x_at	122.3901	56.96858	2.148379	AT4G39230.1	1.00E-138	isoflavone reductase, putative
Cit.16703.1.S1_at	51.21878	23.85759	2.146855		NA	
Cit.31324.1.S1_at	48.89286	22.84652	2.140057	AT5G65850.1	5.00E-07	F-box family protein
Cit.299.1.S1_at	51.35697	24.07844	2.132903		NA	
Cit.20217.1.S1_at	64.11388	30.08854	2.130841	AT3G13050.1	2.00E-24	transporter-related
Cit.22564.1.S1_x_at	52.1486	24.50987	2.127657		NA	
Cit.8093.1.S1_x_at	121.1002	57.04796	2.122779	AT5G59910.1	9.00E-69	HTB4; DNA binding

Cit.37103.1.S1_at	57.5517	27.1679	2.118371	AT4G38800.1	5.00E-05	ATMTN1; catalytic/ methylthioadenosine nucleosidase
Cit.20119.1.S1_s_at	401.0547	189.7312	2.113805		NA	
Cit.21616.1.S1_at	67.2524	31.85669	2.111092	AT2G36380.1	2.00E-11	PDR6; ATPase, coupled to transmembrane movement of
Cit.33490.1.S1_at	70.18625	33.24666	2.111077	AT5G59450.1	6.00E-82	scarecrow-like transcription factor 11 (SCL11)
Cit.6350.1.S1_at	51.01406	24.22881	2.105512	AT1G59940.1	7.00E-12	ARR3 (RESPONSE REGULATOR 3); transcription regulator
Cit.21040.1.S1_at	189.0835	89.9204	2.102788	AT5G11720.1	1.00E-05	alpha-glucosidase 1 (AGLU1)
Cit.2269.1.S1_at	58.90018	28.02137	2.101974	AT5G62190.1	5.00E-63	PRH75; ATP-dependent helicase/ DEAD/H-box RNA helic
Cit.21345.1.S1_at	115.9581	55.41735	2.092451		NA	
Cit.34604.1.S1_at	71.44328	34.14423	2.092397		NA	
Cit.8702.1.S1_s_at	10466.19	5016.016	2.086554	AT1G21310.1	9.00E-78	ATEXT3 (EXTENSIN 3); structural constituent of cell wall
Cit.18633.1.S1_at	67.6981	32.49669	2.083231	AT5G63960.1	1.00E-20	EMB2780 (EMBRYO DEFECTIVE 2780); DNA binding / DN
Cit.6061.1.S1_at	84.83689	40.73167	2.082824		NA	
Cit.21627.1.S1_at	45.83654	22.03883	2.079808	AT5G66240.2	3.00E-07	transducin family protein / WD-40 repeat family protein
Cit.14635.1.S1_at	421.1177	202.4848	2.07975	AT1G74210.1	4.00E-69	glycerophosphoryl diester phosphodiesterase family pro
Cit.22516.1.S1_at	54.84243	26.37592	2.079261		NA	
Cit.24483.1.S1_s_at	86.76575	41.76014	2.077717	AT4G37870.1	1.00E-164	PCK1 (PHOSPHOENOLPYRUVATE CARBOXYKINASE 1); AT
Cit.29751.1.S1_at	444.8984	214.2201	2.076828	AT5G20790.1	7.00E-17	unknown protein
Cit.22463.1.S1_s_at	262.085	126.2021	2.076709	AT4G27410.2	1.00E-110	RD26 (RESPONSIVE TO DESICCATION 26); transcription a
Cit.14592.1.S1_at	127.5929	61.49029	2.075009	AT3G57000.1	2.00E-68	nucleolar essential protein-related
Cit.6860.1.S1_at	97.6851	47.11602	2.073288	AT3G21690.1	6.00E-64	MATE efflux family protein
Cit.25466.1.S1_at	501.9582	243.55	2.061007	AT3G06350.1	4.00E-58	MEE32 (MATERNAL EFFECT EMBRYO ARREST 32); 3-dehy
Cit.18460.1.S1_at	73.56911	35.78464	2.055885		NA	
Cit.374.1.S1_s_at	468.8546	228.1328	2.055183	AT1G69530.2	1.00E-123	ATEXPA1 (ARABIDOPSIS THALIANA EXPANSIN A1)
Cit.6793.1.S1_at	57.80376	28.15976	2.052708	AT4G32280.1	2.00E-09	IAA29 (INDOLE-3-ACETIC ACID INDUCIBLE 29); transcript
Cit.26039.1.S1_x_at	1654.26	808.8542	2.045189	AT5G03300.1	2.00E-17	ADK2 (ADENOSINE KINASE 2); adenosine kinase/ copper
Cit.32873.1.S1_at	66.21829	32.38134	2.044952	AT1G19400.2	9.00E-23	unknown protein
Cit.30234.1.S1_at	134.8167	66.01089	2.04234	AT3G20290.2	5.00E-51	ATEHD1 (EPS15 HOMOLOGY DOMAIN 1); GTP binding / (
Cit.17832.1.S1_at	389.4252	190.7215	2.041853		NA	
Cit.11086.1.S1_at	603.7233	295.8809	2.040427	AT1G02070.1	2.00E-13	unknown protein
Cit.7094.1.S1_at	45.97265	22.54061	2.039548	AT3G51580.1	7.00E-31	unknown protein
Cit.11676.1.S1_at	89.44125	43.87187	2.038692		NA	
Cit.4608.1.S1_at	43.60609	21.39112	2.038514	AT1G23550.1	3.00E-36	SRO2 (SIMILAR TO RCD ONE 2); NAD+ ADP-ribosyltransfe
Cit.38379.1.S1_at	191.5608	93.99622	2.037963	AT3G14180.1	6.00E-24	transcription factor
Cit.7365.1.S1_at	72.63731	35.64551	2.037769	AT5G64880.1	2.00E-06	unknown protein
Cit.3069.1.S1_at	45.2747	22.22166	2.037413	AT5G50915.2	3.00E-44	basic helix-loop-helix (bHLH) family protein

Cit.24135.1.S1_at	53.27362	26.16392	2.036148	AT5G25060.1	9.00E-13	RNA recognition motif (RRM)-containing protein
Cit.6955.1.S1_at	255.8761	125.7901	2.034151	AT5G62460.1	2.00E-51	zinc finger (C3HC4-type RING finger) family protein
Cit.1419.1.S1_s_at	353.9896	174.0242	2.03414	AT5G18670.1	0	BMY3; beta-amylase/ catalytic/ cation binding
Cit.23530.1.S1_x_at	82.63921	40.68639	2.031127		NA	
Cit.17294.1.S1_at	194.132	95.77778	2.0269		NA	
Cit.7567.1.S1_at	69.27354	34.21262	2.024795		NA	
Cit.30643.1.S1_s_at	77.76395	38.41867	2.024119	AT1G04530.1	7.00E-51	binding
Cit.12261.1.S1_s_at	43.61507	21.57134	2.021899	AT1G73300.1	1.00E-113	scpl2 (serine carboxypeptidase-like 2); serine-type carbo
Cit.15649.1.S1_at	73.8981	36.57887	2.02024	AT4G25490.1	1.00E-53	CBF1 (C-REPEAT/DRE BINDING FACTOR 1); DNA binding ,
Cit.31335.1.S1_at	144.0805	71.3659	2.018898	AT2G41540.3	1.00E-09	GPDHC1; NAD or NADH binding / glycerol-3-phosphate c
Cit.5789.1.S1_at	65.81191	32.64164	2.016195	AT4G16765.1	4.00E-94	oxidoreductase, 2OG-Fe(II) oxygenase family protein
Cit.23136.1.S1_x_at	78.18677	38.78327	2.015992		NA	
Cit.5694.1.S1_at	456.544	226.5121	2.015539	AT4G27350.1	4.00E-84	unknown protein
Cit.24481.1.S1_at	138.9614	68.98461	2.014383	AT5G18570.1	2.00E-43	GTP1/OBG family protein
Cit.23003.1.S1_at	186.1622	92.45326	2.013582	AT5G66240.2	3.00E-09	transducin family protein / WD-40 repeat family protein
Cit.32303.1.S1_at	68.01521	33.84978	2.009325		NA	
Cit.32901.1.S1_at	41.14298	20.48792	2.008158		NA	
Cit.6612.1.S1_at	46.59136	23.22312	2.006249	AT1G70210.1	8.00E-98	CYCD1;1 (CYCLIN D1;1); cyclin-dependent protein kinase
Cit.7881.1.S1_at	525.97	262.6225	2.002761	AT3G13224.1	9.00E-49	RNA recognition motif (RRM)-containing protein
Cit.16931.1.S1_at	104.2222	52.05582	2.002124	AT4G02425.1	3.00E-26	unknown protein
Cit.1594.1.S1_at	397.8132	198.8362	2.000708	AT5G17165.1	4.00E-27	unknown protein
Cit.15073.1.S1_at	80.98595	153.5504	-1.89601	AT2G03200.1	6.00E-48	aspartyl protease family protein
Cit.21990.1.S1_at	60.35577	121.3569	-2.01069		NA	
Cit.31495.1.S1_at	41.60182	83.94838	-2.0179	AT2G28290.1	1.00E-07	SYD (SPLAYED); ATPase/ chromatin binding
Cit.31216.1.S1_at	88.21689	178.1596	-2.01956	AT5G38020.1	5.00E-83	S-adenosyl-L-methionine:carboxyl methyltransferase far
Cit.23061.1.S1_at	21.47288	43.62301	-2.03154		NA	
Cit.15543.1.S1_at	57.48971	116.8201	-2.03202	AT5G44390.1	5.00E-23	FAD-binding domain-containing protein
Cit.4416.1.S1_at	34.32482	69.75033	-2.03207	AT1G10470.1	2.00E-64	ARR4 (RESPONSE REGULATOR 4); protein binding / trans
Cit.5791.1.S1_at	22.01056	44.73195	-2.03229	AT5G55400.1	1.00E-109	fimbrin-like protein, putative
Cit.37665.1.S1_x_at	37.73508	76.77457	-2.03457	AT5G24960.1	5.00E-42	CYP71A14; electron carrier/ heme binding / iron ion binc
Cit.22813.1.S1_at	34.03617	69.40474	-2.03915		NA	
Cit.23903.1.S1_s_at	27.01939	55.11274	-2.03975	AT5G53050.3	2.00E-08	hydrolase, alpha/beta fold family protein
Cit.28894.1.S1_at	34.37074	70.11057	-2.03983	AT1G52510.2	3.00E-25	hydrolase, alpha/beta fold family protein
Cit.829.1.S1_at	44.65918	91.35192	-2.04554	AT3G18280.1	2.00E-25	protease inhibitor/seed storage/lipid transfer protein (L
Cit.8608.1.S1_at	471.0848	964.1801	-2.04672	AT5G06740.1	4.00E-21	lectin protein kinase family protein

Cit.7362.1.S1_at	20.61826	42.35441	-2.05422	AT4G31500.1	1.00E-112	CYP83B1 (CYTOCHROME P450 MONOOXYGENASE 83B1)
Cit.23033.1.S1_at	26.41032	54.28668	-2.05551	AT4G16720.1	5.00E-50	60S ribosomal protein L15 (RPL15A)
Cit.17595.1.S1_x_at	46.02769	94.75668	-2.05869		NA	
Cit.20597.1.S1_at	21.32218	44.00954	-2.06403		NA	
Cit.21981.1.S1_x_at	39.93776	82.64891	-2.06944	AT2G16770.1	5.00E-16	DNA binding / transcription factor
Cit.3722.1.S1_s_at	73.7567	153.0024	-2.07442	AT4G10710.1	0	SPT16 (global transcription factor C)
Cit.22641.1.S1_at	31.56446	65.64507	-2.07971	AT2G03220.1	1.00E-66	FT1 (FUCOSYLTRANSFERASE 1); fucosyltransferase/ trans
Cit.8331.1.S1_at	23.49862	48.91058	-2.08142	AT3G27925.1	3.00E-20	DEGP1 (DegP protease 1); serine-type endopeptidase/ se
Cit.17460.1.S1_at	60.19697	125.6615	-2.08751	AT5G54090.1	3.00E-23	DNA mismatch repair MutS family protein
Cit.25558.1.S1_x_at	30.02595	62.68692	-2.08776	AT2G17230.1	8.00E-59	EXL5 (EXORDIUM LIKE 5)
Cit.9706.1.S1_s_at	22.98568	48.11792	-2.09339	AT3G57270.1	1.00E-106	BG1 (BETA-1,3-GLUCANASE 1); catalytic/ cation binding ,
Cit.38620.1.S1_at	27.82704	58.58679	-2.10539		NA	
Cit.28461.1.S1_at	38.47646	81.05994	-2.10674		NA	
Cit.20679.1.S1_at	126.207	266.4032	-2.11084		NA	
Cit.29607.1.S1_at	20.60997	43.53833	-2.11249	AT3G21215.1	1.00E-157	RNA-binding protein, putative
Cit.14627.1.S1_at	44.11849	93.23077	-2.11319		NA	
Cit.15901.1.S1_at	27.55303	58.23808	-2.11367		NA	
Cit.13849.1.S1_at	22.553	47.86789	-2.12246	AT3G04910.3	1.00E-12	WNK1 (WITH NO LYSINE (K) 1); kinase/ protein kinase/ p
Cit.22526.1.S1_at	25.99832	55.23886	-2.12471	AT2G36460.2	8.00E-17	fructose-bisphosphate aldolase, putative
Cit.19983.1.S1_at	25.06415	53.26723	-2.12524		NA	
Cit.5947.1.S1_at	32.16394	68.58421	-2.13233	AT3G18660.2	2.00E-33	PGSIP1 (PLANT GLYCOGENIN-LIKE STARCH INITIATION PI
Cit.11245.1.S1_s_at	30.51474	65.58302	-2.14922	AT5G26742.2	1.00E-40	emb1138 (embryo defective 1138); ATP binding / ATP-de
Cit.12095.1.S1_at	26.21399	56.41153	-2.15196	AT5G56170.1	4.00E-54	FUNCTIONS IN: molecular_function unknown; INVOLVED
Cit.20971.1.S1_at	25.21167	54.59691	-2.16554	AT3G48670.2	1.00E-11	XH/XS domain-containing protein / XS zinc finger domain
Cit.21493.1.S1_at	252.2688	550.474	-2.18209	AT1G06490.1	2.00E-28	ATGSL07 (glucan synthase-like 7); 1,3-beta-glucan syntha
Cit.28085.1.S1_at	33.05342	72.16537	-2.1833	AT5G43600.1	5.00E-51	UAH (UREIDOGLYCOLATE AMIDOHYDROLASE); allantoate
Cit.30899.1.S1_at	23.16541	50.72565	-2.18972	AT4G33740.3	1.00E-11	unknown protein
Cit.542.1.S1_x_at	36.43145	79.84709	-2.19171	AT3G49540.1	1.00E-08	unknown protein
Cit.17732.1.S1_x_at	268.9263	589.8237	-2.19325	AT2G30860.1	1.00E-84	ATGSTF9 (GLUTATHIONE S-TRANSFERASE PHI 9); copper
Cit.40360.1.S1_at	47.40617	104.0771	-2.19543	AT5G22860.2	6.00E-16	serine carboxypeptidase S28 family protein
Cit.20948.1.S1_at	32.57476	71.67575	-2.20035		NA	
Cit.11795.1.S1_at	29.3709	64.6371	-2.20072	AT3G22750.1	4.00E-84	protein kinase, putative
Cit.24204.1.S1_x_at	21.54763	47.47224	-2.20313	AT5G60390.3	6.00E-60	elongation factor 1-alpha / EF-1-alpha
Cit.1126.1.S1_s_at	31.93209	70.37814	-2.20399		NA	
Cit.17550.1.S1_at	54.35332	119.808	-2.20424	AT2G21385.2	3.00E-32	unknown protein

Cit.10798.1.S1_at	20.38509	45.01803	-2.20838	AT1G29880.1	1.00E-129	glycyl-tRNA synthetase / glycine--tRNA ligase
Cit.23919.1.S1_at	30.76957	67.95665	-2.20857	AT1G77470.1	2.00E-05	replication factor C 36 kDA, putative
Cit.28333.1.S1_at	22.32457	49.32587	-2.20949	AT3G06240.1	2.00E-05	F-box family protein
Cit.26085.1.S1_at	43.12346	96.33015	-2.23382		NA	
Cit.29310.1.S1_at	55.40738	124.0624	-2.2391	AT2G38540.1	1.00E-05	LP1; calmodulin binding
Cit.7090.1.S1_at	27.00343	60.58872	-2.24374		NA	
Cit.14618.1.S1_at	27.15565	61.04763	-2.24806	AT5G47670.1	1.00E-55	NF-YB6 (NUCLEAR FACTOR Y, SUBUNIT B6); transcriptior
Cit.25207.1.S1_at	24.44152	55.13921	-2.25596	AT4G24770.1	7.00E-06	RBP31 (31-KDA RNA BINDING PROTEIN); RNA binding / p
Cit.15238.1.S1_at	21.66712	48.95085	-2.25922	AT2G39140.1	5.00E-52	SVR1 (SUPPRESSOR OF VARIEGATION 1); RNA binding / p
Cit.17523.1.S1_at	27.08598	61.44962	-2.26869	AT1G06475.1	4.00E-06	unknown protein
Cit.7460.1.S1_at	22.43327	51.16276	-2.28066		NA	
Cit.30651.1.S1_at	38.72441	88.44589	-2.28398	AT4G34590.1	2.00E-37	GBF6 (G-BOX BINDING FACTOR 6); DNA binding / proteir
Cit.7936.1.S1_at	20.61367	47.8465	-2.32111	AT1G11300.1	4.00E-22	ATP binding / carbohydrate binding / kinase/ protein kin
Cit.26427.1.S1_x_at	36.87792	85.80183	-2.32665		NA	
Cit.5367.1.S1_at	20.08319	47.15286	-2.34788	AT5G15500.2	1.00E-07	ankyrin repeat family protein
Cit.23856.1.S1_x_at	26.1231	61.33953	-2.3481	AT3G52760.1	7.00E-62	integral membrane Yip1 family protein
Cit.25852.1.S1_x_at	24.28991	57.10302	-2.35089		NA	
Cit.23055.1.S1_at	28.58985	67.23684	-2.35177		NA	
Cit.14150.1.S1_s_at	90.85097	213.8158	-2.35348	AT5G24860.1	2.00E-33	FPF1 (FLOWERING PROMOTING FACTOR 1)
Cit.4671.1.S1_at	648.1001	1530.827	-2.36202	AT1G12060.1	6.00E-17	ATBAG5 (ARABIDOPSIS THALIANA BCL-2-ASSOCIATED AT
Cit.39003.1.S1_s_at	102.7588	242.934	-2.36412	AT1G19150.1	1.00E-116	LHCA6; chlorophyll binding
Cit.4910.1.S1_x_at	23.95859	56.82541	-2.37182		NA	
Cit.9343.1.S1_x_at	30.2989	72.21058	-2.38327	AT1G11840.4	3.00E-90	ATGLX1 (GLYOXALASE I HOMOLOG); lactoylglutathione l
Cit.28554.1.S1_at	34.84479	83.08508	-2.38443	AT4G01080.1	8.00E-45	unknown protein
Cit.38247.1.S1_at	26.26492	62.9076	-2.39512		NA	
Cit.11673.1.S1_at	31.04817	74.5195	-2.40013	AT3G05190.1	7.00E-12	aminotransferase class IV family protein
Cit.18624.1.S1_at	20.3501	48.91444	-2.40365		NA	
Cit.13787.1.S1_s_at	76.06541	183.7327	-2.41546	AT2G03200.1	1.00E-50	aspartyl protease family protein
Cit.3922.1.S1_at	26.1338	63.21164	-2.41877	AT1G80760.1	1.00E-138	NIP6;1 (NOD26-LIKE INTRINSIC PROTEIN 6;1); boron tran
Cit.15269.1.S1_at	28.00587	67.75039	-2.41915		NA	
Cit.21150.1.S1_at	40.30083	98.33353	-2.43999		NA	
Cit.21366.1.S1_at	24.47806	59.78489	-2.44239	AT3G26932.2	1.00E-74	DRB3 (dsRNA-binding protein 3); RNA binding / double-s
Cit.13103.1.S1_at	21.12236	52.97556	-2.50803	AT1G79280.1	1.00E-10	NUA (NUCLEAR PORE ANCHOR)
Cit.14512.1.S1_at	32.02924	80.56615	-2.51539		NA	
Cit.13233.1.S1_s_at	21.5306	54.17076	-2.51599	AT5G23260.2	7.00E-50	TT16 (TRANSPARENT TESTA16); transcription factor

Cit.34208.1.S1_at	20.00511	50.69064	-2.53388		NA	
Cit.11139.1.S1_at	31.83681	80.8676	-2.54007	AT5G06700.1	2.00E-76	unknown protein
Cit.33104.1.S1_at	21.3756	54.35138	-2.54268	AT5G62480.2	4.00E-12	ATGSTU9 (ARABIDOPSIS THALIANA GLUTATHIONE S-TRA
Cit.8052.1.S1_x_at	27.26449	69.8935	-2.56354	AT5G63620.1	1.00E-177	oxidoreductase, zinc-binding dehydrogenase family prot
Cit.18274.1.S1_at	29.58945	76.02299	-2.56926	AT1G55250.1	1.00E-18	HUB2 (HISTONE MONO-UBIQUITINATION 2); protein bin
Cit.13570.1.S1_at	28.13245	72.82325	-2.58859	AT5G05670.1	6.00E-89	signal recognition particle binding
Cit.20133.1.S1_at	23.02112	59.76667	-2.59617		NA	
Cit.5435.1.S1_at	20.2299	52.74918	-2.60749	AT5G55760.1	1.00E-13	SRT1 (sirtuin 1); DNA binding / NAD or NADH binding / N
Cit.23257.1.S1_at	28.02728	73.26205	-2.61396	AT4G00740.1	5.00E-10	dehydration-responsive protein-related
Cit.20946.1.S1_at	44.57016	116.9987	-2.62505	AT5G43050.1	8.00E-53	unknown protein
Cit.18717.1.S1_at	318.0254	835.5002	-2.62715		NA	
Cit.1887.1.S1_at	152.225	406.6417	-2.67132	AT1G68090.1	3.00E-90	ANN5; calcium ion binding / calcium-dependent phosph
Cit.27254.1.S1_at	22.33031	60.43284	-2.70631	AT4G20020.2	6.00E-19	unknown protein
Cit.6543.1.S1_at	21.07479	57.72306	-2.73896	AT1G76810.1	2.00E-27	eukaryotic translation initiation factor 2 family protein /
Cit.5262.1.S1_at	21.23851	59.07846	-2.78167	AT5G05340.1	1.00E-115	peroxidase, putative
Cit.18382.1.S1_at	21.42542	60.24762	-2.81197	AT3G14470.1	5.00E-11	disease resistance protein (NBS-LRR class), putative
Cit.16701.1.S1_at	39.88643	113.565	-2.84721		NA	
Cit.12748.1.S1_s_at	111.9375	319.2775	-2.85228	AT4G26530.2	1.00E-172	fructose-bisphosphate aldolase, putative
Cit.20765.1.S1_at	32.23046	92.28675	-2.86334		NA	
Cit.5255.1.S1_at	46.81039	134.0706	-2.86412	AT1G29400.2	4.00E-75	AML5 (ARABIDOPSIS MEI2-LIKE PROTEIN 5); RNA binding
Cit.17415.1.S1_at	65.55671	190.1988	-2.90129	AT1G10070.2	1.00E-125	ATBCAT-2 (ARABIDOPSIS THALIANA BRANCHED-CHAIN A
Cit.38304.1.S1_at	64.4773	187.4568	-2.90733	AT1G72900.1	3.00E-25	disease resistance protein (TIR-NBS class), putative
Cit.25849.1.S1_at	37.06751	108.1628	-2.91799		NA	
Cit.25956.1.S1_x_at	22.42541	66.61787	-2.97064	AT1G26690.1	2.00E-08	emp24/gp25L/p24 family protein
Cit.38095.1.S1_at	26.6476	79.81985	-2.99539		NA	
Cit.28779.1.S1_at	22.80503	68.36595	-2.99785	AT1G67440.1	5.00E-78	emb1688 (embryo defective 1688); GTP binding / GTPas
Cit.5524.1.S1_at	49.71338	149.0897	-2.99899	AT4G01970.1	4.00E-29	AtSTS (Arabidopsis thaliana stachyose synthase); galacti
Cit.19813.1.S1_at	20.48631	61.45227	-2.99967	AT3G14470.1	9.00E-10	disease resistance protein (NBS-LRR class), putative
Cit.23343.1.S1_at	20.89976	63.35259	-3.03126	AT2G36270.1	3.00E-47	ABI5 (ABA INSENSITIVE 5); DNA binding / transcription a
Cit.26629.1.S1_at	21.42596	64.9798	-3.03276		NA	
Cit.13854.1.S1_at	20.27711	61.95157	-3.05525		NA	
Cit.6773.1.S1_at	20.09152	65.05351	-3.23786	AT2G16890.2	8.00E-58	UDP-glucoronosyl/UDP-glucosyl transferase family prote
Cit.30568.1.S1_x_at	259.8394	883.2369	-3.39916	AT1G63830.1	2.00E-73	proline-rich family protein
Cit.4353.1.S1_at	134.4244	464.831	-3.45794	AT5G09530.1	9.00E-74	hydroxyproline-rich glycoprotein family protein
Cit.814.1.S1_s_at	53.84759	189.5364	-3.51987	AT3G01500.3	1.00E-134	CA1 (CARBONIC ANHYDRASE 1); carbonate dehydratase/

Cit.26276.1.S1_at	183.3185	658.489	-3.59205	AT4G21200.1	2.00E-22	GA2OX8 (GIBBERELLIN 2-OXIDASE 8); gibberellin 2-beta-
Cit.27244.1.S1_at	150.0744	550.9257	-3.67102	AT5G64650.1	6.00E-54	ribosomal protein L17 family protein
Cit.6467.1.S1_s_at	185.7964	688.6945	-3.70672		NA	
Cit.4353.1.S1_s_at	137.8856	522.7448	-3.79115	AT5G09530.1	6.00E-83	hydroxyproline-rich glycoprotein family protein
Cit.36807.1.S1_s_at	64.59457	250.6793	-3.88081	AT4G21200.1	2.00E-74	GA2OX8 (GIBBERELLIN 2-OXIDASE 8); gibberellin 2-beta-
Cit.30421.1.S1_x_at	40.54687	159.3013	-3.92882	AT5G47550.1	7.00E-27	cysteine protease inhibitor, putative / cystatin, putative
Cit.22077.1.S1_at	23.15742	95.34277	-4.11716		NA	
Cit.3990.1.S1_at	29.52881	126.5329	-4.28507	AT4G27990.1	1.00E-57	YGGT family protein
Cit.2346.1.S1_at	25.0113	126.4685	-5.05645	AT2G13360.2	0	AGT (ALANINE:GLYOXYLATE AMINOTRANSFERASE); alani
Cit.8163.1.S1_x_at	1014.146	5911.082	-5.82863	AT5G47550.1	2.00E-23	cysteine protease inhibitor, putative / cystatin, putative
Cit.30421.1.S1_s_at	516.3066	3052.314	-5.91182	AT5G47550.1	7.00E-27	cysteine protease inhibitor, putative / cystatin, putative
Cit.28011.1.S1_x_at	370.6159	2268.196	-6.12007	AT5G47550.1	4.00E-25	cysteine protease inhibitor, putative / cystatin, putative
Cit.18430.1.S1_s_at	34.88031	269.8145	-7.73544		NA	

Supplementary Table S9. PDTA in seedless vs. seedy pineapple fruits at time point 3.

ProbeSet ID	Pineapple_ Seedless	Pineapple _seedy	Ratio	AtGID	E-Score	Arabidopsis.annotation
Cit.3549.1.S1_at	949.0838	183.7332	5.165554	AT3G12360.1	9.00E-55	ITN1 (INCREASED TOLERANCE TO NACL); protein binding
Cit.10894.1.S1_s_at	8464.059	1780.699	4.753223	AT5G06760.1	1.00E-48	late embryogenesis abundant group 1 domain-containing protein / l
Cit.27244.1.S1_at	628.2031	156.3747	4.017294	AT5G64650.1	6.00E-54	ribosomal protein L17 family protein
Cit.522.1.S1_s_at	589.0655	154.4964	3.812811	AT4G05320.4	0	UBQ10 (POLYUBIQUITIN 10); protein binding
Cit.22462.1.S1_at	125.4055	33.29817	3.766138	AT1G17330.1	2.00E-95	metal-dependent phosphohydrolase HD domain-containing protein
Cit.3778.1.S1_at	130.9069	40.41476	3.239086	AT1G19210.1	1.00E-45	AP2 domain-containing transcription factor, putative
Cit.34386.1.S1_at	95.50946	29.94856	3.189117		NA	
Cit.559.1.S1_at	151.7901	47.95023	3.165576		NA	
Cit.16087.1.S1_at	307.6605	97.36445	3.159885	AT3G16030.1	1.00E-80	CES101 (CALLUS EXPRESSION OF RBCS 101); ATP binding / carbohyd
Cit.7522.1.S1_at	85.36806	27.02247	3.159151	AT4G17300.1	1.00E-113	NS1; asparagine-tRNA ligase
Cit.24078.1.S1_at	71.82222	23.38269	3.071598		NA	
Cit.17325.1.S1_at	404.9929	132.9055	3.047225	AT2G18660.1	5.00E-32	EXLB3 (EXPANSIN-LIKE B3 PRECURSOR)
Cit.29915.1.S1_at	187.8589	62.99426	2.982159		NA	
Cit.38380.1.S1_at	61.98574	21.04214	2.945791		NA	
Cit.11960.1.S1_at	69.24217	23.56734	2.938056	AT1G66430.1	1.00E-159	pfkB-type carbohydrate kinase family protein
Cit.8616.1.S1_x_at	241.7861	82.86547	2.917815	AT4G09320.1	5.00E-64	NDPK1; ATP binding / nucleoside diphosphate kinase
Cit.35750.1.S1_at	75.42385	25.92346	2.909482	AT1G77360.1	5.00E-81	pentatricopeptide (PPR) repeat-containing protein
Cit.7314.1.S1_at	615.6135	211.6787	2.908245	AT5G55000.2	1.00E-126	FIP2; protein binding / voltage-gated potassium channel
Cit.22748.1.S1_x_at	250.5105	86.86642	2.883859	AT2G29250.1	3.00E-11	lectin protein kinase, putative
Cit.8320.1.S1_at	189.0493	66.48517	2.843481	AT1G74270.1	4.00E-50	60S ribosomal protein L35a (RPL35aC)
Cit.31296.1.S1_at	81.18092	28.57779	2.8407		NA	
Cit.29883.1.S1_at	79.9956	28.21828	2.834886		NA	
Cit.10062.1.S1_at	90.03383	32.01072	2.812615	AT3G47340.1	1.00E-19	ASN1 (GLUTAMINE-DEPENDENT ASPARAGINE SYNTHASE 1); asparag
Cit.30976.1.S1_at	181.6669	64.60029	2.812168	AT3G12650.1	8.00E-45	unknown protein
Cit.39268.1.S1_s_at	351.342	125.1559	2.807235	AT4G32830.1	1.00E-133	AtAUR1 (ATAURORA1); histone kinase(H3-S10 specific) / kinase/ prc
Cit.21983.1.S1_s_at	63.72789	22.72878	2.803841		NA	
Cit.26518.1.S1_at	160.8219	57.56048	2.793964		NA	
Cit.34457.1.S1_at	86.27869	31.07258	2.776683	AT1G70290.1	4.00E-60	ATTPS8; alpha,alpha-trehalose-phosphate synthase (UDP-forming)/
Cit.32435.1.S1_at	115.0755	41.91012	2.745769	AT3G20550.1	8.00E-15	DDL (DAWDLE)
Cit.24776.1.S1_at	54.41178	20.00674	2.719672	AT3G15810.1	2.00E-68	unknown protein
Cit.17675.1.S1_at	514.352	189.2051	2.718489	AT1G26250.1	2.00E-29	proline-rich extensin, putative

Cit.4482.1.S1_at	76.21876	28.08601	2.713762		NA	
Cit.7256.1.S1_at	64.82956	23.95462	2.706349	AT4G21790.1	5.00E-39	TOM1 (TOBAMOVIRUS MULTIPLICATION 1); protein binding
Cit.37306.1.S1_at	100.6084	37.61584	2.674629	AT4G22250.1	2.00E-10	zinc finger (C3HC4-type RING finger) family protein
Cit.32193.1.S1_at	220.458	83.20422	2.649601	AT1G13980.1	1.00E-16	GN (GNOM); GTP:GDP antiporter/ protein homodimerization
Cit.31057.1.S1_s_at	204.3323	77.3517	2.641601	AT5G17540.1	7.00E-34	transferase family protein
Cit.4533.1.S1_s_at	77.6532	29.64454	2.619477	AT4G16800.1	9.00E-87	enoyl-CoA hydratase, putative
Cit.7340.1.S1_at	69.28842	26.48837	2.615805	AT4G25200.1	1.00E-39	ATHSP23.6-MITO (MITOCHONDRION-LOCALIZED SMALL HEAT SHOC
Cit.4970.1.S1_s_at	102.4414	39.32437	2.605036	AT5G35732.1	2.00E-16	unknown protein
Cit.14849.1.S1_at	200.5797	77.94624	2.573308	AT5G49555.1	1.00E-67	amine oxidase-related
Cit.38340.1.S1_at	76.03668	29.74707	2.556107		NA	
Cit.37273.1.S1_at	57.39486	22.50244	2.550606		NA	
Cit.7235.1.S1_at	488.5555	191.7033	2.548498		NA	
Cit.26587.1.S1_at	111.3268	43.9904	2.530707	AT5G39050.1	3.00E-35	transferase/ transferase, transferring acyl groups other than amino-
Cit.21616.1.S1_at	180.153	71.41873	2.52249	AT2G36380.1	2.00E-11	PDR6; ATPase, coupled to transmembrane movement of substances
Cit.17425.1.S1_s_at	103.7323	41.21418	2.516908	AT1G28380.1	4.00E-86	NSL1 (necrotic spotted lesions 1)
Cit.29546.1.S1_at	55.29696	21.99016	2.514623	AT3G03900.1	1.00E-63	adenylylsulfate kinase, putative
Cit.22325.1.S1_x_at	630.6533	253.4463	2.488311	AT2G27880.1	4.00E-42	AGO5 (ARGONAUTE 5); nucleic acid binding
Cit.19161.1.S1_at	54.42554	21.90377	2.484757	AT4G19040.2	8.00E-59	EDR2; lipid binding
Cit.30057.1.S1_at	229.9833	92.94183	2.474486	AT2G21380.1	2.00E-19	kinesin motor protein-related
Cit.39416.1.S1_at	62.06665	25.09149	2.473614	AT5G50320.1	1.00E-57	ELO3 (ELONGATA 3); histone acetyltransferase
Cit.11922.1.S1_at	299.5059	121.4648	2.465784	AT5G10550.1	4.00E-15	GTE2 (Global transcription factor group E 2); DNA binding
Cit.15741.1.S1_at	57.85336	23.47815	2.464136	AT1G11330.1	3.00E-71	S-locus lectin protein kinase family protein
Cit.5608.1.S1_at	240.8334	97.74825	2.463813	AT3G52270.1	5.00E-86	ATP binding / RNA polymerase II transcription factor
Cit.4550.1.S1_at	1198.644	489.9141	2.446641	AT1G05000.1	6.00E-85	tyrosine specific protein phosphatase family protein
Cit.26938.1.S1_at	115.4805	47.25195	2.443931	AT4G38220.2	2.00E-44	aminoacylase, putative / N-acyl-L-amino-acid amidohydrolase, puta
Cit.35345.1.S1_s_at	105.1904	43.13338	2.438724	AT3G28340.1	1.00E-139	GATL10 (Galacturonosyltransferase-like 10); polygalacturonate 4-al
Cit.7103.1.S1_at	54.64086	22.45605	2.433236	AT3G01470.1	6.00E-24	ATHB-1 (ARABIDOPSIS THALIANA HOMEOBOX 1); DNA binding / pro
Cit.12441.1.S1_at	52.46063	21.58067	2.430908	AT4G25520.1	1.00E-11	SLK1 (SEUSS-LIKE 1); transcription regulator
Cit.40262.1.S1_at	598.379	246.413	2.428358	AT2G42200.1	4.00E-38	SPL9 (SQUAMOSA PROMOTER BINDING PROTEIN-LIKE 9); transcript
Cit.30068.1.S1_at	53.40549	22.04655	2.422397	AT4G31120.2	2.00E-27	SKB1 (SHK1 BINDING PROTEIN 1); protein methyltransferase
Cit.38210.1.S1_at	85.68787	35.5587	2.409758	AT2G28100.1	4.00E-52	ATFUC1 (alpha-L-fucosidase 1); alpha-L-fucosidase
Cit.10194.1.S1_x_at	73.27364	30.65924	2.389937	AT1G30910.1	1.00E-123	molybdenum cofactor sulfurase family protein
Cit.31072.1.S1_at	64.91306	27.23721	2.383249	AT3G06880.1	6.00E-07	nucleotide binding
Cit.3904.1.S1_at	1136.928	477.7713	2.379649	AT2G48020.1	0	sugar transporter, putative
Cit.2455.1.S1_s_at	361.2238	152.7679	2.364527	AT5G48335.1	1.00E-10	unknown protein

Cit.25956.1.S1_at	67.99815	28.83564	2.358129	AT1G26690.1	2.00E-08	emp24/gp25L/p24 family protein
Cit.6138.1.S1_at	684.8756	293.8551	2.330658	AT5G56120.1	9.00E-87	unknown protein
Cit.17260.1.S1_at	65.99475	28.54426	2.312015	AT3G09270.1	2.00E-49	ATGSTU8 (GLUTATHIONE S-TRANSFERASE TAU 8); glutathione trans
Cit.25956.1.S1_x_at	64.30032	27.84412	2.309296	AT1G26690.1	2.00E-08	emp24/gp25L/p24 family protein
Cit.25231.1.S1_x_at	71.29911	30.98192	2.301313		NA	
Cit.27960.1.S1_at	46.22709	20.1584	2.293192	AT2G13840.1	7.00E-86	PHP domain-containing protein
Cit.31591.1.S1_at	55.90531	24.40244	2.290972	AT2G27760.1	2.00E-52	ATIPT2 (TRNA ISOPENTENYLTRANSFERASE 2); adenylate dimethylall
Cit.11367.1.S1_x_at	930.681	409.4138	2.273204	AT1G69840.6	1.00E-126	band 7 family protein
Cit.37424.1.S1_at	108.1661	47.62231	2.271332		NA	
Cit.16794.1.S1_at	63.26413	27.85633	2.271086	AT5G10460.1	4.00E-26	haloacid dehalogenase-like hydrolase family protein
Cit.30425.1.S1_x_at	10047.55	4441.257	2.262321	AT3G12500.1	3.00E-93	ATHCHIB (ARABIDOPSIS THALIANA BASIC CHITINASE); chitinase
Cit.24266.1.S1_at	80.42986	35.66335	2.255253	AT2G33845.1	4.00E-22	DNA-binding protein-related
Cit.28117.1.S1_s_at	55.32048	24.54025	2.254275	AT5G33340.1	1.00E-34	CDR1 (CONSTITUTIVE DISEASE RESISTANCE 1); aspartic-type endope
Cit.31274.1.S1_at	494.3813	219.3218	2.254137	AT3G02410.1	4.00E-87	INVOLVED IN: biological_process unknown; LOCATED IN: cellular_cc
Cit.39183.1.S1_at	1048.448	467.5063	2.242639		NA	
Cit.31170.1.S1_at	45.81691	20.47522	2.237676	AT4G24290.2	3.00E-37	FUNCTIONS IN: molecular_function unknown; INVOLVED IN: biologi
Cit.4253.1.S1_at	116.809	52.27795	2.234384		NA	
Cit.14913.1.S1_at	57.71166	25.85333	2.232272	AT5G24090.1	2.00E-64	acidic endochitinase (CHIB1)
Cit.14331.1.S1_at	100.1685	44.90564	2.230644	AT1G76130.1	3.00E-83	AMY2 (ALPHA-AMYLASE-LIKE 2); alpha-amylase/ calcium ion binding
Cit.30296.1.S1_at	67.88457	30.53252	2.223353	AT5G59030.1	3.00E-38	COPT1 (copper transporter 1); copper ion transmembrane transport
Cit.38820.1.S1_at	99.8703	44.9371	2.222446	AT4G39970.1	1.00E-23	haloacid dehalogenase-like hydrolase family protein
Cit.2569.1.S1_at	119.355	53.74543	2.220747	AT1G80670.1	5.00E-48	transducin family protein / WD-40 repeat family protein
Cit.28087.1.S1_s_at	139.9887	63.10525	2.218337	AT1G53160.1	6.00E-26	SPL4 (SQUAMOSA PROMOTER BINDING PROTEIN-LIKE 4); DNA bindi
Cit.25332.1.S1_at	199.3954	90.42867	2.205002		NA	
Cit.17449.1.S1_at	160.8274	73.10425	2.199973	AT5G20410.1	4.00E-69	MGD2; 1,2-diacylglycerol 3-beta-galactosyltransferase/ UDP-galacto
Cit.6647.1.S1_s_at	419.0146	191.296	2.190399	AT5G41080.2	1.00E-106	glycerophosphoryl diester phosphodiesterase family protein
Cit.40018.1.S1_s_at	46.79714	21.40412	2.186361	AT1G04945.2	2.00E-58	FUNCTIONS IN: molecular_function unknown; INVOLVED IN: biologi
Cit.24513.1.S1_at	337.746	154.6045	2.184581	AT2G18350.1	1.00E-31	AtHB24 (ARABIDOPSIS THALIANA HOMEOBOX PROTEIN 24); DNA bi
Cit.6371.1.S1_at	120.7116	55.36483	2.180294	AT2G41540.3	8.00E-83	GPDHC1; NAD or NADH binding / glycerol-3-phosphate dehydrogena
Cit.48.1.S1_x_at	51.26907	23.52149	2.179669		NA	
Cit.28386.1.S1_s_at	98.72462	45.39853	2.174622	AT4G16800.1	4.00E-17	enoyl-CoA hydratase, putative
Cit.28244.1.S1_at	53.16682	24.47459	2.172327	AT1G14650.2	1.00E-56	SWAP (Suppressor-of-White-APricot)/surp domain-containing prote
Cit.21970.1.S1_at	51.70444	23.84395	2.168451		NA	
Cit.30109.1.S1_at	75.58206	34.89808	2.165794	AT3G04680.2	1.00E-77	CLPS3 (CLP-SIMILAR PROTEIN 3); binding
Cit.31122.1.S1_at	46.86653	21.66665	2.163072	AT2G26180.1	5.00E-38	IQD6 (IQ-domain 6); calmodulin binding

Cit.6136.1.S1_at	109.4116	50.64456	2.160382	AT3G51570.1	3.00E-05	disease resistance protein (TIR-NBS-LRR class), putative
Cit.14375.1.S1_x_at	444.501	205.8205	2.159654	AT4G11600.1	7.00E-07	ATGPX6 (GLUTATHIONE PEROXIDASE 6); glutathione peroxidase
Cit.28672.1.S1_at	64.79169	30.02745	2.157749		NA	
Cit.18556.1.S1_at	360.2116	166.952	2.157576		NA	
Cit.23892.1.S1_at	86.20749	39.98412	2.156043	AT1G63250.1	9.00E-45	DEAD box RNA helicase, putative
Cit.15424.1.S1_at	544.7402	252.7184	2.155523	AT1G29790.2	4.00E-78	FUNCTIONS IN: molecular_function unknown; INVOLVED IN: biologi
Cit.11806.1.S1_at	52.31479	24.29246	2.15354	AT2G31050.1	4.00E-26	plastocyanin-like domain-containing protein
Cit.11795.1.S1_at	52.52566	24.4047	2.152276	AT3G22750.1	4.00E-84	protein kinase, putative
Cit.4253.1.S1_x_at	168.581	78.33221	2.152129		NA	
Cit.11735.1.S1_at	78.86808	36.65088	2.151874	AT1G71980.1	1.00E-38	protease-associated zinc finger (C3HC4-type RING finger) family pro
Cit.29698.1.S1_at	54.59068	25.43689	2.146122	AT1G34220.2	6.00E-12	unknown protein
Cit.38147.1.S1_at	45.94385	21.41847	2.145058		NA	
Cit.31690.1.S1_at	96.26431	44.88819	2.144535	AT1G72390.1	6.00E-23	unknown protein
Cit.21254.1.S1_at	92.62286	43.20829	2.143636	AT1G77180.2	3.00E-23	chromatin protein family
Cit.22892.1.S1_at	208.0997	97.15084	2.142027		NA	
Cit.7399.1.S1_at	391.9767	183.3438	2.137933	AT1G35350.1	1.00E-157	LOCATED IN: integral to membrane; EXPRESSED IN: 18 plant structu
Cit.22228.1.S1_at	104.6876	49.04274	2.13462	AT1G28330.4	6.00E-21	DYL1 (DORMANCY-ASSOCIATED PROTEIN-LIKE 1)
Cit.37664.1.S1_at	45.86861	21.50076	2.133348	AT3G09640.2	2.00E-05	APX2 (ASCORBATE PEROXIDASE 2); L-ascorbate peroxidase
Cit.12777.1.S1_at	617.6127	290.4381	2.126487	AT4G36850.1	1.00E-143	INVOLVED IN: biological_process unknown; LOCATED IN: membrane
Cit.34703.1.S1_at	181.1626	85.36107	2.122309	AT3G54380.1	8.00E-45	SAC3/GANP family protein
Cit.7831.1.S1_at	57.09346	26.90775	2.121822	AT4G27240.1	5.00E-55	zinc finger (C2H2 type) family protein
Cit.16105.1.S1_at	63.13827	29.76419	2.121283	AT4G11130.1	2.00E-72	RDR2 (RNA-DEPENDENT RNA POLYMERASE 2); RNA-directed RNA po
Cit.38876.1.S1_at	43.08529	20.31243	2.121129	AT3G51930.1	2.00E-24	transducin family protein / WD-40 repeat family protein
Cit.12285.1.S1_x_at	60.14036	28.35516	2.120967	AT1G53310.3	1.00E-88	ATPPC1 (PHOSPHOENOLPYRUVATE CARBOXYLASE 1); catalytic/ pho
Cit.31631.1.S1_at	118.1715	55.78218	2.118445	AT5G51030.1	3.00E-20	short-chain dehydrogenase/reductase (SDR) family protein
Cit.3911.1.S1_s_at	133.3893	62.98791	2.117697	AT3G45140.1	1.00E-125	LOX2 (LIPOXYGENASE 2); lipoxygenase
Cit.31443.1.S1_at	58.61531	27.6897	2.116863	AT1G24793.2	8.00E-41	UDP-3-O-[3-hydroxymyristoyl] N-acetylglucosamine deacetylase
Cit.10669.1.S1_s_at	4216.392	1992.842	2.115768	AT2G15960.1	7.00E-09	unknown protein
Cit.16802.1.S1_at	262.3829	124.3378	2.110242	AT4G21820.1	7.00E-24	binding / calmodulin binding
Cit.37918.1.S1_at	47.62798	22.59925	2.107503	AT2G38940.1	3.00E-52	ATPT2 (ARABIDOPSIS THALIANA PHOSPHATE TRANSPORTER 2); carb
Cit.30003.1.S1_at	200.9142	95.61711	2.101237	AT1G67440.1	1.00E-165	emb1688 (embryo defective 1688); GTP binding / GTPase
Cit.2559.1.S1_s_at	474.6053	225.9008	2.100946	AT5G06860.1	1.00E-127	PGIP1 (POLYGALACTURONASE INHIBITING PROTEIN 1); protein bind
Cit.31147.1.S1_at	68.07884	32.42348	2.099677	AT3G46710.1	2.00E-31	disease resistance protein (CC-NBS-LRR class), putative
Cit.40444.1.S1_at	384.0995	182.9831	2.099098		NA	
Cit.26163.1.S1_at	94.03146	44.99188	2.089965	AT5G14760.1	2.00E-78	AO (L-ASPARTATE OXIDASE); L-aspartate oxidase/ electron carrier/ c

Cit.12618.1.S1_s_at	332.7205	159.2117	2.089799	AT3G02550.1	9.00E-83	LBD41 (LOB DOMAIN-CONTAINING PROTEIN 41)
Cit.23669.1.S1_at	228.0426	109.336	2.085705	AT2G37420.1	2.00E-33	kinesin motor protein-related
Cit.32235.1.S1_at	395.9219	190.4431	2.078951	AT5G55520.2	1.00E-37	INVOLVED IN: biological_process unknown; EXPRESSED IN: 15 plant
Cit.5127.1.S1_at	305.4329	147.0945	2.07644	AT3G51000.1	1.00E-106	epoxide hydrolase, putative
Cit.1802.1.S1_at	257.0966	123.9938	2.073463	AT4G12590.1	1.00E-124	unknown protein
Cit.16645.1.S1_at	195.2069	94.27215	2.070674	AT1G31800.1	7.00E-47	CYP97A3 (CYTOCHROME P450-TYPE MONOOXYGENASE 97A3); caro
Cit.38588.1.S1_at	154.2856	74.62517	2.067474	AT1G35580.2	1.00E-16	CINV1 (cytosolic invertase 1); beta-fructofuranosidase
Cit.28123.1.S1_at	69.41405	33.7439	2.057084	AT2G21580.2	2.00E-11	40S ribosomal protein S25 (RPS25B)
Cit.23679.1.S1_at	252.7097	122.8809	2.056542	AT3G49725.1	2.00E-14	GTP binding
Cit.27804.1.S1_at	324.6375	158.0021	2.05464	AT2G01630.1	1.00E-86	glycosyl hydrolase family 17 protein / beta-1,3-glucanase, putative
Cit.7350.1.S1_at	193.8411	94.38385	2.053753	AT1G17720.2	1.00E-133	ATB BETA; nucleotide binding / protein phosphatase type 2A regula
Cit.28645.1.S1_at	76.56793	37.29658	2.052948	AT3G03310.1	3.00E-16	lecithin:cholesterol acyltransferase family protein / LACT family prot
Cit.16581.1.S1_s_at	50.21833	24.50163	2.049591	AT1G10320.1	2.00E-87	U2 snRNP auxiliary factor-related
Cit.24992.1.S1_s_at	642.4807	313.948	2.046456	AT1G15490.1	6.00E-48	hydrolase, alpha/beta fold family protein
Cit.30018.1.S1_at	473.6965	231.5217	2.046013	AT2G39260.1	1.00E-16	RNA binding / binding / protein binding
Cit.16692.1.S1_at	402.4081	197.2764	2.039819	AT2G03430.1	3.00E-82	ankyrin repeat family protein
Cit.36490.1.S1_s_at	80.15585	39.36703	2.036116	AT5G60760.1	7.00E-93	2-phosphoglycerate kinase-related
Cit.16594.1.S1_at	70.46413	34.63573	2.034435	AT4G39640.2	1.00E-143	GGT1 (GAMMA-GLUTAMYL TRANSPEPTIDASE 1); gamma-glutamyltr
Cit.30040.1.S1_at	215.9008	106.244	2.032122	AT2G47320.1	7.00E-48	peptidyl-prolyl cis-trans isomerase cyclophilin-type family protein
Cit.29667.1.S1_at	137.7609	67.79504	2.03202	AT5G40270.1	1.00E-58	metal-dependent phosphohydrolase HD domain-containing protein
Cit.34961.1.S1_at	48.0637	23.68455	2.029327	AT4G21490.1	1.00E-47	NDB3; NADH dehydrogenase
Cit.38404.1.S1_at	112.5216	55.46248	2.028788	AT1G06660.1	4.00E-14	unknown protein
Cit.7921.1.S1_at	104.2274	51.3944	2.027991		NA	
Cit.30480.1.S1_s_at	169.0634	83.37206	2.027818	AT4G23100.1	0	GSH1 (GLUTAMATE-CYSTEINE LIGASE); glutamate-cysteine ligase
Cit.9623.1.S1_x_at	621.1498	306.5404	2.026323	AT1G61800.1	6.00E-22	GPT2; antiporter/ glucose-6-phosphate transmembrane transporter
Cit.186.1.S1_x_at	102.637	50.66813	2.025672	AT1G32410.5	1.00E-54	vacuolar protein sorting 55 family protein / VPS55 family protein
Cit.30333.1.S1_at	127.1738	62.91779	2.021269	AT1G14790.1	8.00E-72	RDR1 (RNA-DEPENDENT RNA POLYMERASE 1); RNA-directed RNA po
Cit.21988.1.S1_s_at	353.2197	174.8587	2.020029	AT5G20410.1	8.00E-74	MGD2; 1,2-diacylglycerol 3-beta-galactosyltransferase/ UDP-galacto
Cit.29830.1.S1_at	261.5027	129.5799	2.018081	AT5G65640.2	8.00E-23	bHLH093 (beta HLH protein 93); DNA binding / transcription factor
Cit.27164.1.S1_s_at	330.3629	163.8353	2.016433	AT1G44110.1	1.00E-112	CYCA1;1 (Cyclin A1;1); cyclin-dependent protein kinase regulator
Cit.25712.1.S1_at	133.3314	66.13956	2.01591		NA	
Cit.30559.1.S1_s_at	1474.115	731.8193	2.014316	AT1G33540.1	1.00E-118	scpl18 (serine carboxypeptidase-like 18); serine-type carboxypeptid
Cit.11096.1.S1_s_at	47.97766	23.82021	2.014158	AT3G46970.1	0	PHS2 (ALPHA-GLUCAN PHOSPHORYLASE 2); phosphorylase/ transfer
Cit.15253.1.S1_at	42.41894	21.09938	2.010435	AT3G54340.1	5.00E-61	AP3 (APETALA 3); DNA binding / transcription factor
Cit.20838.1.S1_x_at	376.7762	187.4429	2.010085		NA	

Cit.22771.1.S1_at	70.26238	34.96913	2.009269		NA	
Cit.9873.1.S1_at	197.1291	98.19314	2.007565	AT4G32330.3	4.00E-12	FUNCTIONS IN: molecular_function unknown; INVOLVED IN: biologi
Cit.25101.1.S1_at	401.8513	200.2992	2.006255	AT1G48050.1	2.00E-35	KU80; double-stranded DNA binding / protein binding
Cit.9020.1.S1_s_at	82.82744	41.28893	2.006045	AT4G30880.1	5.00E-21	protease inhibitor/seed storage/lipid transfer protein (LTP) family p
Cit.24953.1.S1_at	114.7358	57.23986	2.004474	AT4G33460.1	1.00E-79	ATNAP13; transporter
Cit.3465.1.S1_at	46.28325	23.10767	2.002939	AT1G16520.1	7.00E-96	unknown protein
Cit.4549.1.S1_s_at	361.8678	180.8464	2.000968	AT1G05000.1	2.00E-86	tyrosine specific protein phosphatase family protein
Cit.10123.1.S1_at	81.3316	40.6501	2.000772	AT5G02560.1	6.00E-35	HTA12; DNA binding
Cit.2519.1.S1_at	323.5208	646.9807	-1.99981	AT1G79870.1	1.00E-144	oxidoreductase family protein
Cit.13710.1.S1_at	29.92284	59.87485	-2.00097	AT5G43280.1	1.00E-109	ATDCI1 (DELTA(3,5),DELTA(2,4)-DIENOYL-COA ISOMERASE 1); delta3
Cit.24626.1.S1_x_at	62.73176	125.6228	-2.00254	AT2G16060.1	2.00E-65	AHB1 (ARABIDOPSIS HEMOGLOBIN 1); oxygen binding / oxygen tran
Cit.12019.1.S1_at	98.78291	197.9471	-2.00386	AT1G48110.2	1.00E-161	ECT7 (evolutionarily conserved C-terminal region 7)
Cit.6179.1.S1_at	32.88501	65.89805	-2.00389	AT3G49890.1	3.00E-44	unknown protein
Cit.23533.1.S1_at	22.86954	45.83035	-2.00399		NA	
Cit.7164.1.S1_at	29.9867	60.09948	-2.0042	AT4G01070.1	1.00E-64	GT72B1; UDP-glucosyltransferase/ UDP-glycosyltransferase/ transfe
Cit.161.1.S1_at	284.3079	570.3512	-2.0061	AT4G05320.4	0	UBQ10 (POLYUBIQUITIN 10); protein binding
Cit.2086.1.S1_at	579.9601	1163.461	-2.00611	AT4G01150.1	3.00E-54	unknown protein
Cit.13940.1.S1_at	215.9205	433.2629	-2.00659	AT1G06620.1	1.00E-101	2-oxoglutarate-dependent dioxygenase, putative
Cit.27802.1.S1_at	28.96963	58.14178	-2.00699	AT1G80160.1	2.00E-27	lactoylglutathione lyase family protein / glyoxalase I family protein
Cit.15643.1.S1_s_at	115.6852	232.2583	-2.00768	AT4G31940.1	8.00E-59	CYP82C4; electron carrier/ heme binding / iron ion binding / monoo
Cit.8549.1.S1_s_at	28.22201	56.67332	-2.00812	AT2G16600.1	2.00E-85	ROC3; peptidyl-prolyl cis-trans isomerase
Cit.30937.1.S1_at	41.24856	82.84904	-2.00853	AT1G11200.1	1.00E-134	unknown protein
Cit.64.1.S1_at	21.5314	43.24888	-2.00864	AT4G11600.1	2.00E-78	ATGPX6 (GLUTATHIONE PEROXIDASE 6); glutathione peroxidase
Cit.9634.1.S1_at	399.0637	801.8582	-2.00935		NA	
Cit.6170.1.S1_at	34.89146	70.13895	-2.0102		NA	
Cit.9063.1.S1_at	509.723	1027.062	-2.01494	AT5G39850.1	1.00E-101	40S ribosomal protein S9 (RPS9C)
Cit.9447.1.S1_at	183.136	369.2904	-2.01648	AT4G10300.1	1.00E-46	FUNCTIONS IN: molecular_function unknown; LOCATED IN: chloropl
Cit.21206.1.S1_x_at	451.5475	910.8134	-2.01709		NA	
Cit.20980.1.S1_at	1168.194	2356.588	-2.01729	AT1G80920.1	4.00E-48	J8; heat shock protein binding / unfolded protein binding
Cit.18008.1.S1_at	47.45446	95.81236	-2.01904		NA	
Cit.8953.1.S1_at	45.8719	92.69655	-2.02077	AT2G21580.1	1.00E-52	40S ribosomal protein S25 (RPS25B)
Cit.11548.1.S1_at	50.03883	101.1556	-2.02154	AT1G20030.2	3.00E-79	pathogenesis-related thaumatin family protein
Cit.6165.1.S1_at	38.32288	77.53077	-2.02309	AT3G23560.1	6.00E-72	ALF5 (ABERRANT LATERAL ROOT FORMATION 5); antiporter/ drug tr
Cit.16927.1.S1_x_at	49.14426	99.43219	-2.02327	AT2G38540.1	2.00E-29	LP1; calmodulin binding
Cit.25928.1.S1_at	870.9025	1762.449	-2.0237		NA	

Cit.29560.1.S1_at	113.8139	230.3313	-2.02375	AT5G12240.1	1.00E-11	unknown protein
Cit.28175.1.S1_at	37.35453	75.61635	-2.02429	AT4G31985.1	3.00E-15	60S ribosomal protein L39 (RPL39C)
Cit.17344.1.S1_x_at	22.98115	46.54255	-2.02525		NA	
Cit.10028.1.S1_x_at	208.0631	421.755	-2.02705	AT3G15260.2	1.00E-127	protein phosphatase 2C, putative / PP2C, putative
Cit.34157.1.S1_at	69.38671	140.6888	-2.0276	AT3G01370.1	1.00E-13	ATCFM2 (CRM FAMILY MEMBER 2); RNA binding
Cit.30970.1.S1_at	21.53008	43.67818	-2.0287	AT5G11330.1	6.00E-47	monooxygenase family protein
Cit.2729.1.S1_at	129.52	262.9787	-2.03041	AT3G12760.1	1.00E-116	FUNCTIONS IN: molecular_function unknown; INVOLVED IN: biologi
Cit.5121.1.S1_at	62.67048	127.2841	-2.03101	AT5G57685.1	2.00E-33	AtGDU3 (Arabidopsis thaliana GLUTAMINE DUMPER 3)
Cit.20098.1.S1_at	289.9229	588.8473	-2.03105		NA	
Cit.30105.1.S1_at	117.3072	238.5438	-2.0335		NA	
Cit.17523.1.S1_at	32.65184	66.39876	-2.03354	AT1G06475.1	4.00E-06	unknown protein
Cit.2509.1.S1_at	346.7821	705.3681	-2.03404	AT3G44850.1	1.00E-106	protein kinase-related
Cit.29877.1.S1_at	525.0151	1067.958	-2.03415		NA	
Cit.18782.1.S1_s_at	869.8151	1769.688	-2.03456	AT5G47640.1	5.00E-63	NF-YB2 (NUCLEAR FACTOR Y, SUBUNIT B2); transcription factor
Cit.1433.1.S1_at	24.80693	50.49214	-2.0354	AT4G26480.1	1.00E-82	KH domain-containing protein
Cit.15846.1.S1_at	32.22728	65.6511	-2.03713		NA	
Cit.6209.1.S1_at	104.0706	212.021	-2.03728	AT5G16070.1	6.00E-69	chaperonin, putative
Cit.32634.1.S1_at	23.53956	47.95718	-2.0373	AT2G28500.1	1.00E-05	LBD11 (LOB DOMAIN-CONTAINING PROTEIN 11)
Cit.1575.1.S1_at	1394.458	2841.325	-2.03758	AT3G05530.1	0	RPT5A (REGULATORY PARTICLE TRIPLE-A ATPASE 5A); ATPase/ calm
Cit.36526.1.S1_at	80.37086	163.8341	-2.03848	AT1G58684.1	3.00E-10	40S ribosomal protein S2, putative
Cit.146.1.S1_x_at	1430.086	2917.227	-2.0399		NA	
Cit.24025.1.S1_s_at	3730.267	7613.889	-2.04111	AT1G67340.1	4.00E-33	zinc finger (MYND type) family protein / F-box family protein
Cit.165.1.S1_s_at	1582.678	3231.526	-2.04181	AT3G22840.1	3.00E-56	ELIP1 (EARLY LIGHT-INDUCABLE PROTEIN); chlorophyll binding
Cit.29301.1.S1_s_at	138.2894	282.5872	-2.04345	AT3G13227.1	5.00E-07	serine-rich protein-related
Cit.30365.1.S1_at	38.00765	77.66919	-2.04351	AT5G49650.1	1.00E-05	xylulose kinase, putative
Cit.594.1.S1_at	27.89102	57.05357	-2.04559		NA	
Cit.24300.1.S1_at	56.88168	116.4016	-2.04638	AT5G43720.1	1.00E-22	unknown protein
Cit.21819.1.S1_at	130.8225	267.874	-2.04761	AT3G52105.1	1.00E-06	unknown protein
Cit.17528.1.S1_at	108.4209	222.2447	-2.04983		NA	
Cit.12772.1.S1_at	114.1598	234.062	-2.0503	AT2G37990.1	1.00E-128	ribosome biogenesis regulatory protein (RRS1) family protein
Cit.25150.1.S1_at	37.57385	77.07414	-2.05127	AT2G29760.1	4.00E-38	pentatricopeptide (PPR) repeat-containing protein
Cit.30507.1.S1_at	473.3311	971.0262	-2.05147	AT5G23740.1	4.00E-26	RPS11-BETA (RIBOSOMAL PROTEIN S11-BETA); structural constituer
Cit.22854.1.S1_at	72.18093	148.0796	-2.05151	AT3G24200.2	1.00E-70	FAD binding / monooxygenase/ oxidoreductase/ oxidoreductase, ac
Cit.12525.1.S1_at	116.4094	238.8654	-2.05194	AT1G14820.3	6.00E-89	SEC14 cytosolic factor family protein / phosphoglyceride transfer fa
Cit.10389.1.S1_x_at	226.8034	465.5893	-2.05283	AT4G38510.4	0	vacuolar ATP synthase subunit B, putative / V-ATPase B subunit, put

Cit.9552.1.S1_at	48.69397	99.96211	-2.05286	AT5G58710.1	1.00E-95	ROC7; peptidyl-prolyl cis-trans isomerase
Cit.21025.1.S1_s_at	279.1355	573.3104	-2.05388	AT4G14210.1	0	PDS3 (PHYTOENE DESATURASE 3); phytoene dehydrogenase
Cit.29442.1.S1_s_at	78.27652	160.7906	-2.05414	AT4G35160.1	5.00E-45	O-methyltransferase family 2 protein
Cit.31070.1.S1_at	606.3979	1247.48	-2.0572	ATCG00530.1	3.00E-21	hypothetical protein
Cit.27344.1.S1_at	20.2225	41.61198	-2.05771	AT5G42905.1	1.00E-27	nucleic acid binding / ribonuclease H
Cit.19439.1.S1_s_at	63.6013	130.9033	-2.05819		NA	
Cit.1268.1.S1_at	261.4684	538.2308	-2.05849	AT4G13500.1	2.00E-37	unknown protein
Cit.27935.1.S1_at	132.8166	273.505	-2.05927	AT2G47260.1	3.00E-48	WRKY23; transcription factor
Cit.31773.1.S1_at	28.46905	58.63759	-2.0597	AT5G17690.1	5.00E-15	TFL2 (TERMINAL FLOWER 2); DNA binding / methylated histone resi
Cit.34684.1.S1_at	28.37013	58.43666	-2.0598	AT1G16670.1	2.00E-17	protein kinase family protein
Cit.1681.1.S1_at	21.56329	44.41843	-2.05991	AT1G29050.1	1.00E-157	unknown protein
Cit.13144.1.S1_at	23.72452	48.88409	-2.06049	AT2G48010.1	1.00E-86	RKF3 (RECEPTOR-LIKE KINASE IN IN FLOWERS 3); kinase/ receptor si
Cit.28733.1.S1_at	25.44481	52.44855	-2.06127	AT2G41705.2	9.00E-57	camphor resistance CrcB family protein
Cit.21218.1.S1_at	140.9058	290.5973	-2.06235		NA	
Cit.12105.1.S1_at	25.0905	51.77144	-2.06339	AT5G16820.2	1.00E-128	HSF3 (HEAT SHOCK FACTOR 3); DNA binding / transcription factor
Cit.16919.1.S1_s_at	85.74535	176.9865	-2.06409	AT3G51630.1	1.00E-105	WNK5 (WITH NO LYSINE (K) KINASE 5); protein kinase
Cit.24983.1.S1_at	26.02471	53.74324	-2.06509		NA	
Cit.10050.1.S1_s_at	1789.059	3694.564	-2.06509	AT5G42190.1	2.00E-68	ASK2 (ARABIDOPSIS SKP1-LIKE 2); protein binding / ubiquitin-protei
Cit.23168.1.S1_at	33.43518	69.08766	-2.06632		NA	
Cit.25271.1.S1_x_at	42.47719	87.80862	-2.06719		NA	
Cit.7516.1.S1_at	54.00384	111.7006	-2.06838	AT5G55160.1	5.00E-16	SUMO2 (SMALL UBIQUITIN-LIKE MODIFIER 2); protein binding / prot
Cit.32269.1.S1_at	21.8396	45.18661	-2.06902		NA	
Cit.18739.1.S1_at	793.5597	1643.399	-2.07092	AT1G08380.1	3.00E-49	PSAO (photosystem I subunit O)
Cit.478.1.S1_at	263.0256	545.1166	-2.07248	AT2G23250.1	1.00E-109	UGT84B2 (UDP-glucosyl transferase 84B2); UDP-glycosyltransferase
Cit.28377.1.S1_at	106.3064	220.3383	-2.07267	AT2G36290.1	5.00E-55	hydrolase, alpha/beta fold family protein
Cit.17677.1.S1_at	433.8685	899.3247	-2.0728	AT2G30570.1	7.00E-32	PSBW (PHOTOSYSTEM II REACTION CENTER W)
Cit.20462.1.S1_at	624.4273	1294.63	-2.07331	AT1G06550.1	1.00E-20	enoyl-CoA hydratase/isomerase family protein
Cit.30902.1.S1_at	21.0761	43.76954	-2.07674	AT1G44191.1	7.00E-11	Encodes a ECA1 gametogenesis related family protein
Cit.24778.1.S1_at	48.93137	101.6993	-2.07841	AT2G43280.1	8.00E-41	far-red impaired responsive family protein / FAR1 family protein
Cit.20128.1.S1_at	24.62671	51.18629	-2.07849	AT3G26070.1	5.00E-25	plastid-lipid associated protein PAP / fibrillin family protein
Cit.8995.1.S1_at	47.2438	98.27557	-2.08018		NA	
Cit.6230.1.S1_at	43.2436	90.08759	-2.08326	AT5G28350.2	3.00E-80	FUNCTIONS IN: molecular_function unknown; INVOLVED IN: biologi
Cit.20021.1.S1_at	121.1183	252.6741	-2.08618		NA	
Cit.10453.1.S1_s_at	2226.984	4648.481	-2.08734		NA	
Cit.31245.1.S1_at	29.88024	62.39262	-2.08809		NA	

Cit.8420.1.S1_at	71.29572	148.8802	-2.08821	AT1G66240.1	5.00E-32	ATX1 (ARABIDOPSIS HOMOLOG OF ANTI-OXIDANT 1); metal ion bind
Cit.29626.1.S1_s_at	23.30729	48.68644	-2.08889	AT5G50260.1	1.00E-158	cysteine proteinase, putative
Cit.29123.1.S1_x_at	57.74112	120.75	-2.09123	AT1G17860.1	4.00E-09	trypsin and protease inhibitor family protein / Kunitz family protein
Cit.30593.1.S1_at	149.7811	313.269	-2.09151	AT3G12320.1	2.00E-12	unknown protein
Cit.23136.1.S1_x_at	36.21135	75.84192	-2.09442		NA	
Cit.26568.1.S1_at	22.89286	47.96407	-2.09515	AT4G22000.1	5.00E-17	unknown protein
Cit.24400.1.S1_at	39.17905	82.117	-2.09594		NA	
Cit.30285.1.S1_at	25.02871	52.46488	-2.09619		NA	
Cit.3336.1.S1_at	431.2724	904.1344	-2.09643	AT1G74410.1	4.00E-70	zinc finger (C3HC4-type RING finger) family protein
Cit.40013.1.S1_at	21.32718	44.73566	-2.09759	AT1G29890.2	2.00E-56	acetyltransferase-related
Cit.448.1.S1_at	23.36081	49.03853	-2.09918	AT1G23820.1	1.00E-147	SPDS1 (spermidine synthase 1); spermidine synthase
Cit.10662.1.S1_at	629.9579	1322.431	-2.09924	AT5G41685.1	1.00E-22	mitochondrial import receptor subunit TOM7 / translocase of outer
Cit.29310.1.S1_at	80.71988	169.4676	-2.09945	AT2G38540.1	1.00E-05	LP1; calmodulin binding
Cit.354.1.S1_at	29.42179	61.87263	-2.10295	AT3G15353.1	1.00E-17	MT3 (METALLOTHIONEIN 3); copper ion binding
Cit.9161.1.S1_s_at	682.9754	1436.977	-2.104	AT1G15270.1	2.00E-28	FUNCTIONS IN: molecular_function unknown; INVOLVED IN: biologi
Cit.1928.1.S1_s_at	651.5905	1371.96	-2.10556	AT1G78860.1	1.00E-117	curculin-like (mannose-binding) lectin family protein
Cit.7462.1.S1_at	842.6393	1775.187	-2.1067	AT4G22110.2	1.00E-51	alcohol dehydrogenase, putative
Cit.37189.1.S1_at	68.54485	144.4059	-2.10674		NA	
Cit.17489.1.S1_at	824.1931	1736.465	-2.10687	ATCG00800.1	7.00E-26	encodes a chloroplast ribosomal protein S3, a constituent of the sm
Cit.29439.1.S1_at	30.8006	64.90009	-2.1071	AT4G10270.1	2.00E-25	wound-responsive family protein
Cit.22697.1.S1_x_at	4206.364	8873.794	-2.10961		NA	
Cit.29497.1.S1_at	26.70483	56.39925	-2.11195	AT2G38610.2	7.00E-67	KH domain-containing protein
Cit.4089.1.S1_at	84.44762	178.3732	-2.11223	AT5G13530.1	1.00E-147	KEG (KEEP ON GOING); protein binding / protein kinase/ ubiquitin-p
Cit.20048.1.S1_s_at	295.2014	623.8461	-2.11329		NA	
Cit.3661.1.S1_at	420.9862	890.3043	-2.11481	AT1G60710.1	1.00E-113	ATB2; oxidoreductase
Cit.3002.1.S1_s_at	114.5361	242.2922	-2.11542	AT4G12320.1	1.00E-135	CYP706A6; electron carrier/ heme binding / iron ion binding / mono
Cit.8720.1.S1_x_at	208.5858	441.2689	-2.11553	AT5G54160.1	7.00E-35	ATOMT1 (O-METHYLTRANSFERASE 1); caffeate O-methyltransferase
Cit.19739.1.S1_at	601.7209	1273.659	-2.11669		NA	
Cit.8874.1.S1_at	675.3612	1431.321	-2.11934	AT4G38970.1	0	fructose-bisphosphate aldolase, putative
Cit.4456.1.S1_at	268.8008	569.8201	-2.11986	AT1G65890.1	3.00E-73	AAE12 (ACYL ACTIVATING ENZYME 12); catalytic
Cit.22776.1.S1_s_at	203.7317	432.2608	-2.12172	AT4G32480.1	5.00E-71	unknown protein
Cit.9345.1.S1_at	84.62929	179.5829	-2.12199	AT5G52570.1	7.00E-98	BETA-OHASE 2 (BETA-CAROTENE HYDROXYLASE 2); carotene beta-ri
Cit.18353.1.S1_x_at	5800.406	12309.04	-2.1221	AT3G15353.1	7.00E-18	MT3 (METALLOTHIONEIN 3); copper ion binding
Cit.20227.1.S1_at	35.085	74.56553	-2.12528		NA	
Cit.11471.1.S1_at	305.4336	649.2573	-2.12569	AT3G08510.2	4.00E-69	ATPLC2 (PHOSPHOLIPASE C 2); phospholipase C

Cit.11156.1.S1_s_at	23.27592	49.52778	-2.12785	AT4G22070.1	1.00E-149	WRKY31; transcription factor
Cit.4525.1.S1_at	67.19795	143.0821	-2.12926	AT4G34131.1	7.00E-30	UGT73B3 (UDP-glucosyl transferase 73B3); UDP-glycosyltransferase
Cit.15744.1.S1_at	58.4084	124.3784	-2.12946		NA	
Cit.29853.1.S1_at	62.7999	133.8154	-2.13082	AT2G38060.1	3.00E-18	PHT4;2 (PHOSPHATE TRANSPORTER 4;2); carbohydrate transmembr
Cit.26693.1.S1_at	20.05596	42.74659	-2.13137	AT1G47500.1	3.00E-39	ATRBP47C' (RNA-binding protein 47C'); RNA binding
Cit.32883.1.S1_at	38.03799	81.0949	-2.13194	AT5G01225.1	3.00E-21	unknown protein
Cit.191.1.S1_at	495.1527	1055.768	-2.13221	AT3G62290.1	1.00E-100	ATARFA1E (ADP-ribosylation factor A1E); GTP binding / phospholipa
Cit.23228.1.S1_x_at	31.32266	66.88669	-2.13541	AT2G35110.2	1.00E-07	GRL (GNARLED); transcription activator
Cit.13258.1.S1_x_at	149.5743	319.4619	-2.13581	AT4G34215.2	2.00E-86	hydrolase
Cit.2693.1.S1_at	1205.421	2577.893	-2.13858	AT5G20060.3	1.00E-114	phospholipase/carboxylesterase family protein
Cit.574.1.S1_at	601.1011	1285.636	-2.1388		NA	
Cit.16490.1.S1_at	28.84009	61.71288	-2.13983	AT1G11190.1	1.00E-130	BFN1 (BIFUNCTIONAL NUCLEASE I); T/G mismatch-specific endonucl
Cit.37138.1.S1_at	34.74173	74.35192	-2.14013	AT5G56810.1	2.00E-09	F-box family protein
Cit.4819.1.S1_at	48.14203	103.0503	-2.14055	AT3G57680.1	0	peptidase S41 family protein
Cit.18743.1.S1_at	25.90538	55.48287	-2.14175		NA	
Cit.12189.1.S1_s_at	47.73936	102.2629	-2.14211	AT2G35940.3	2.00E-10	BLH1 (BEL1-LIKE HOMEODOMAIN 1); DNA binding / protein heteroc
Cit.24791.1.S1_x_at	498.7462	1068.618	-2.14261		NA	
Cit.6849.1.S1_at	66.66148	142.8534	-2.14297	AT5G41800.1	1.00E-65	amino acid transporter family protein
Cit.2786.1.S1_at	803.377	1722.582	-2.14418		NA	
Cit.13694.1.S1_at	141.6054	303.6729	-2.1445	AT4G38960.1	6.00E-28	zinc finger (B-box type) family protein
Cit.6801.1.S1_at	47.91408	102.8579	-2.14672	AT5G35450.1	3.00E-06	disease resistance protein (CC-NBS-LRR class), putative
Cit.14831.1.S1_at	25.05607	53.78885	-2.14674	AT5G14980.1	4.00E-92	esterase/lipase/thioesterase family protein
Cit.1866.1.S1_at	106.5379	228.7647	-2.14726	AT2G22470.1	7.00E-23	AGP2 (ARABINOGALACTAN PROTEIN 2)
Cit.29366.1.S1_at	412.4072	885.8105	-2.1479	AT5G11330.1	1.00E-07	monooxygenase family protein
Cit.24609.1.S1_at	22.01384	47.31361	-2.14927		NA	
Cit.9723.1.S1_s_at	363.631	781.8625	-2.15015	AT4G20260.3	5.00E-57	DREPP plasma membrane polypeptide family protein
Cit.16876.1.S1_at	335.7355	721.8928	-2.15018	AT3G24100.1	1.00E-22	FUNCTIONS IN: molecular_function unknown; INVOLVED IN: biologi
Cit.3646.1.S1_at	20.93875	45.04047	-2.15106	AT1G60780.1	2.00E-82	HAP13 (HAPLESS 13); protein binding
Cit.9067.1.S1_at	761.3624	1637.964	-2.15136	AT2G32520.1	1.00E-117	dienelactone hydrolase family protein
Cit.9822.1.S1_s_at	527.1319	1134.846	-2.15287	AT2G47140.1	6.00E-41	short-chain dehydrogenase/reductase (SDR) family protein
Cit.20193.1.S1_at	34.14564	73.52441	-2.15326		NA	
Cit.4422.1.S1_at	335.7608	723.2775	-2.15415	AT4G01070.1	1.00E-144	GT72B1; UDP-glucosyltransferase/ UDP-glycosyltransferase/ transfe
Cit.22243.1.S1_at	100.8926	217.339	-2.15416	AT1G18800.1	2.00E-28	NRP2 (NAP1-RELATED PROTEIN 2); DNA binding / chromatin binding
Cit.14541.1.S1_at	25.48092	54.9109	-2.15498	AT5G58005.2	5.00E-39	unknown protein
Cit.8510.1.S1_x_at	48.65602	104.9403	-2.15678	AT2G38380.1	7.00E-37	peroxidase 22 (PER22) (P22) (PRXEA) / basic peroxidase E

Cit.7773.1.S1_at	41.54136	89.62639	-2.15752		NA	
Cit.30801.1.S1_at	25.69701	55.45028	-2.15785	AT3G11570.1	4.00E-33	unknown protein
Cit.3990.1.S1_at	43.48803	93.85629	-2.15821	AT4G27990.1	1.00E-57	YGGT family protein
Cit.30610.1.S1_at	127.9918	276.2826	-2.1586	AT1G15750.4	5.00E-76	TPL (TOPLESS); protein binding / protein homodimerization/ transcr
Cit.749.1.S1_x_at	82.41891	177.9093	-2.1586	AT1G73230.1	1.00E-69	nascent polypeptide-associated complex (NAC) domain-containing p
Cit.11296.1.S1_at	333.9423	721.072	-2.15927	AT5G37540.1	2.00E-87	aspartyl protease family protein
Cit.12814.1.S1_at	257.1381	555.389	-2.15989	AT5G07330.1	2.00E-36	unknown protein
Cit.20852.1.S1_s_at	149.977	324.1343	-2.16123	AT5G65140.1	1.00E-136	trehalose-6-phosphate phosphatase, putative
Cit.4205.1.S1_at	149.1158	322.335	-2.16164		NA	
Cit.1751.1.S1_s_at	41.30652	89.32964	-2.1626	AT1G67920.1	2.00E-13	unknown protein
Cit.12229.1.S1_at	336.9246	728.6575	-2.16267	AT4G04640.1	1.00E-166	ATPC1; enzyme regulator
Cit.21553.1.S1_at	52.12304	112.7251	-2.16267	AT5G17280.1	1.00E-19	unknown protein
Cit.4152.1.S1_at	89.05021	193.0361	-2.16772	AT4G37880.1	1.00E-118	protein binding / zinc ion binding
Cit.6910.1.S1_at	51.02685	110.6369	-2.16821	AT1G13030.1	6.00E-11	sphere organelles protein-related
Cit.13425.1.S1_at	113.9488	247.1662	-2.1691	AT2G39730.1	1.00E-169	RCA (RUBISCO ACTIVASE); ADP binding / ATP binding / enzyme regu
Cit.29563.1.S1_at	41.95027	91.09137	-2.17141	AT2G44600.1	6.00E-56	unknown protein
Cit.6827.1.S1_x_at	45.26925	98.36341	-2.17285	AT1G17010.1	7.00E-81	oxidoreductase, 2OG-Fe(II) oxygenase family protein
Cit.57.1.S1_x_at	407.7222	886.2563	-2.17368	AT1G17860.1	2.00E-27	trypsin and protease inhibitor family protein / Kunitz family protein
Cit.29307.1.S1_at	20.76814	45.17255	-2.17509		NA	
Cit.28996.1.S1_at	33.31951	72.50113	-2.17594	AT5G12370.2	7.00E-28	SEC10 (EXOCYST COMPLEX COMPONENT SEC10)
Cit.24987.1.S1_at	119.8394	260.8429	-2.1766		NA	
Cit.22555.1.S1_at	54.73293	119.1401	-2.17675		NA	
Cit.36157.1.S1_at	22.87709	49.84198	-2.17869	AT5G26680.2	1.00E-16	endonuclease, putative
Cit.26152.1.S1_at	33.97657	74.08546	-2.18049		NA	
Cit.36935.1.S1_s_at	23.0784	50.32663	-2.18068	AT1G19640.1	2.00E-91	JMT (JASMONIC ACID CARBOXYL METHYLTRANSFERASE); jasmonate
Cit.29293.1.S1_at	53.14542	115.9477	-2.18171	ATCG01010.1	5.00E-37	Chloroplast encoded NADH dehydrogenase unit.
Cit.6763.1.S1_at	24.70995	53.93932	-2.1829	AT4G15880.1	4.00E-18	ESD4 (EARLY IN SHORT DAYS 4); SUMO-specific protease/ cysteine-t
Cit.19421.1.S1_at	30.34334	66.32195	-2.18572		NA	
Cit.38359.1.S1_at	23.90429	52.26916	-2.1866		NA	
Cit.29486.1.S1_at	34.8084	76.13124	-2.18715	AT5G15610.2	3.00E-57	proteasome family protein
Cit.14211.1.S1_at	80.61669	176.3742	-2.18781	AT1G68185.1	7.00E-55	ubiquitin-related
Cit.27537.1.S1_at	40.14022	87.89201	-2.18962	AT5G48930.1	1.00E-25	HCT (HYDROXYCINNAMOYL-COA SHIKIMATE/QUINATE HYDROXYCIN
Cit.13978.1.S1_at	51.55802	113.0579	-2.19283	AT4G16510.1	3.00E-92	YbaK/prolyl-tRNA synthetase-related
Cit.38996.1.S1_s_at	97.76676	214.4839	-2.19383	AT3G03870.2	1.00E-37	unknown protein
Cit.18015.1.S1_s_at	2062.809	4527.03	-2.19459	AT3G29780.1	9.00E-05	RALFL27 (ralf-like 27); signal transducer

Cit.23192.1.S1_x_at	30.93183	67.92789	-2.19605		NA	
Cit.8564.1.S1_at	2138.106	4697.187	-2.19689	AT5G62350.1	9.00E-38	invertase/pectin methylesterase inhibitor family protein / DC 1.2 ho
Cit.20395.1.S1_at	22.89014	50.33424	-2.19895		NA	
Cit.20635.1.S1_at	30.16568	66.39342	-2.20096		NA	
Cit.7108.1.S1_at	27.89958	61.44781	-2.20246		NA	
Cit.23107.1.S1_x_at	21.97785	48.40616	-2.2025		NA	
Cit.5327.1.S1_at	67.19004	148.0456	-2.20339	AT5G16990.1	5.00E-06	NADP-dependent oxidoreductase, putative
Cit.38372.1.S1_at	22.99305	50.67131	-2.20377	AT2G29510.1	4.00E-15	unknown protein
Cit.17460.1.S1_at	97.0537	213.9057	-2.20399	AT5G54090.1	3.00E-23	DNA mismatch repair MutS family protein
Cit.31090.1.S1_at	72.11015	158.9583	-2.20438	AT3G61690.1	5.00E-57	unknown protein
Cit.9144.1.S1_at	392.6008	865.7409	-2.20514	AT5G50920.1	1.00E-108	CLPC1; ATP binding / ATP-dependent peptidase/ ATPase
Cit.7343.1.S1_at	86.84245	191.7632	-2.20817	AT5G01830.1	1.00E-131	armadillo/beta-catenin repeat family protein / U-box domain-conta
Cit.23109.1.S1_x_at	67.46564	149.0297	-2.20897	AT5G26570.1	4.00E-14	ATGWD3; carbohydrate kinase/ catalytic/ phosphoglucan, water dik
Cit.29255.1.S1_at	26.32359	58.15063	-2.20907		NA	
Cit.30124.1.S1_at	59.0467	130.5008	-2.21013	AT1G64230.3	6.00E-07	ubiquitin-conjugating enzyme, putative
Cit.4192.1.S1_at	29.97998	66.26572	-2.21033	AT1G60200.1	1.00E-11	splicing factor PWI domain-containing protein / RNA recognition mo
Cit.1176.1.S1_at	380.5524	841.2238	-2.21053	AT3G51800.1	1.00E-124	ATG2; aminopeptidase/ metalloexopeptidase
Cit.28253.1.S1_at	21.48695	47.50488	-2.21087	AT3G26220.1	9.00E-54	CYP71B3; electron carrier/ heme binding / iron ion binding / monoo
Cit.11802.1.S1_at	261.0914	577.4299	-2.2116	AT5G24690.1	5.00E-54	INVOLVED IN: biological_process unknown; LOCATED IN: mitochonc
Cit.7775.1.S1_at	40.67226	89.9612	-2.21186	AT1G17010.1	8.00E-11	oxidoreductase, 2OG-Fe(II) oxygenase family protein
Cit.10595.1.S1_at	23.47045	51.94179	-2.21307		NA	
Cit.10162.1.S1_at	389.4416	863.3937	-2.217	AT1G44575.1	1.00E-102	NPQ4 (NONPHOTOCHEMICAL QUENCHING); chlorophyll binding / xa
Cit.28855.1.S1_at	145.8982	323.7227	-2.21883	AT3G26040.1	7.00E-28	transferase family protein
Cit.2312.1.S1_at	26.29091	58.3732	-2.22028	AT3G25520.1	2.00E-50	ATL5 (A. THALIANA RIBOSOMAL PROTEIN L5); 5S rRNA binding / stru
Cit.31144.1.S1_at	30.68109	68.15345	-2.22135	AT1G10070.3	1.00E-119	ATBCAT-2 (ARABIDOPSIS THALIANA BRANCHED-CHAIN AMINO ACID
Cit.13644.1.S1_x_at	40.0379	88.97949	-2.22238		NA	
Cit.15471.1.S1_at	132.8216	295.2312	-2.22276	AT3G17460.1	3.00E-32	PHD finger family protein
Cit.6784.1.S1_at	38.37572	85.32519	-2.22342	AT5G46560.1	1.00E-103	unknown protein
Cit.13708.1.S1_at	44.04937	97.9918	-2.22459	AT5G63400.1	1.00E-111	ADK1 (ADENYLATE KINASE 1); ATP binding / adenylate kinase/ nucle
Cit.16732.1.S1_s_at	582.6438	1296.34	-2.22493	AT2G26890.2	9.00E-16	GRV2 (GRAVITROPISM DEFECTIVE 2); binding / heat shock protein b
Cit.14154.1.S1_at	27.6052	61.4376	-2.22558	AT1G68825.2	6.00E-13	RTFL15 (ROTUNDIFOLIA LIKE 15)
Cit.25759.1.S1_at	81.55354	181.5259	-2.22585	AT4G02380.1	8.00E-09	SAG21 (SENESCENCE-ASSOCIATED GENE 21)
Cit.34139.1.S1_at	37.05929	82.52782	-2.22691	AT3G07860.1	2.00E-47	unknown protein
Cit.11166.1.S1_at	43.40508	96.72769	-2.22849	AT4G33250.1	4.00E-96	EIF3K (EUKARYOTIC TRANSLATION INITIATION FACTOR 3K); translati
Cit.20294.1.S1_at	24.36298	54.36906	-2.23163		NA	

Cit.2486.1.S1_at	65.15678	145.4413	-2.23217	AT5G64920.1	2.00E-85	CIP8 (COP1-INTERACTING PROTEIN 8); protein binding / zinc ion bin
Cit.22519.1.S1_s_at	1163.916	2601.6	-2.23521		NA	
Cit.22642.1.S1_at	67.3353	150.5367	-2.23563	AT4G15520.1	2.00E-18	tRNA/rRNA methyltransferase (SpoU) family protein
Cit.379.1.S1_s_at	1388.87	3106.516	-2.23672	AT3G13200.1	1.00E-107	EMB2769 (EMBRYO DEFECTIVE 2769)
Cit.28752.1.S1_x_at	21.51217	48.12962	-2.23732	AT1G48030.2	9.00E-36	mtLPD1 (mitochondrial lipoamide dehydrogenase 1); ATP binding /
Cit.19182.1.S1_at	28.686	64.18421	-2.23748		NA	
Cit.12090.1.S1_at	81.51176	182.3974	-2.23768		NA	
Cit.21433.1.S1_x_at	137.7986	308.9238	-2.24185		NA	
Cit.13936.1.S1_s_at	25.04686	56.1741	-2.24276		NA	
Cit.18283.1.S1_at	94.47945	212.0818	-2.24474		NA	
Cit.17907.1.S1_at	992.0178	2227.35	-2.24527	AT1G49320.1	3.00E-44	BURP domain-containing protein
Cit.11985.1.S1_at	24.89824	55.91449	-2.24572	AT5G52300.2	2.00E-66	LTI65 (LOW-TEMPERATURE-INDUCED 65)
Cit.14513.1.S1_at	69.31467	155.6799	-2.24599	AT1G77140.1	2.00E-62	VPS45 (VACUOLAR PROTEIN SORTING 45); protein transporter
Cit.12855.1.S1_at	22.82008	51.27231	-2.24681	AT4G33625.1	9.00E-72	unknown protein
Cit.29383.1.S1_at	42.30561	95.0598	-2.24698	AT1G01470.1	1.00E-46	LEA14 (LATE EMBRYOGENESIS ABUNDANT 14)
Cit.4412.1.S1_at	36.50071	82.2104	-2.2523	AT2G10950.1	2.00E-61	BSD domain-containing protein
Cit.3526.1.S1_at	343.9345	775.1498	-2.25377	AT1G34780.2	2.00E-40	ATAPRL4 (APR-like 4)
Cit.9462.1.S1_x_at	382.5701	862.4611	-2.25439	AT1G07400.1	2.00E-62	17.8 kDa class I heat shock protein (HSP17.8-CI)
Cit.9068.1.S1_s_at	468.2367	1055.904	-2.25506	AT2G32520.1	2.00E-87	dienelactone hydrolase family protein
Cit.15732.1.S1_at	68.27157	154.1919	-2.25851		NA	
Cit.27433.1.S1_at	30.75328	69.49826	-2.25986	AT1G29040.3	7.00E-19	unknown protein
Cit.1601.1.S1_at	36.46891	82.41876	-2.25997	AT1G80510.1	0	amino acid transporter family protein
Cit.30866.1.S1_at	29.89257	67.56651	-2.26031	AT5G41980.1	1.00E-26	unknown protein
Cit.12037.1.S1_at	928.3799	2098.762	-2.26067		NA	
Cit.15944.1.S1_x_at	33.36279	75.4258	-2.26078	AT1G17860.1	5.00E-09	trypsin and protease inhibitor family protein / Kunitz family protein
Cit.1366.1.S1_x_at	91.08456	206.0136	-2.26178	AT3G16920.1	1.00E-154	chitinase
Cit.24003.1.S1_at	52.46968	118.7078	-2.26241	AT1G05310.1	1.00E-06	pectinesterase family protein
Cit.4996.1.S1_at	148.0932	335.2542	-2.26381	AT3G11450.1	2.00E-55	DNAJ heat shock N-terminal domain-containing protein / cell divisio
Cit.19780.1.S1_at	1257.015	2846.058	-2.26414		NA	
Cit.20637.1.S1_at	28.77264	65.15125	-2.26435	AT5G40800.1	6.00E-28	unknown protein
Cit.10905.1.S1_at	43.4672	98.55978	-2.26745	AT5G13810.1	1.00E-82	glutaredoxin family protein
Cit.22303.1.S1_at	287.8919	653.1721	-2.26881	AT3G46660.1	7.00E-46	UGT76E12 (UDP-GLUCOSYL TRANSFERASE 76E12); UDP-glycosyltran
Cit.18438.1.S1_s_at	252.3179	573.1808	-2.27166	AT4G13340.1	7.00E-25	leucine-rich repeat family protein / extensin family protein
Cit.22005.1.S1_x_at	79.82786	181.3817	-2.27216	AT1G17860.1	3.00E-10	trypsin and protease inhibitor family protein / Kunitz family protein
Cit.1780.1.S1_at	716.5482	1628.243	-2.27234	AT2G29500.1	2.00E-58	17.6 kDa class I small heat shock protein (HSP17.6B-CI)

Cit.11691.1.S1_at	772.5612	1756.856	-2.27407	AT3G50770.1	2.00E-49	calmodulin-related protein, putative
Cit.20628.1.S1_s_at	1413.186	3219.301	-2.27804	AT2G48150.1	1.00E-69	ATGPX4 (GLUTATHIONE PEROXIDASE 4); glutathione peroxidase
Cit.29343.1.S1_at	420.0373	958.2939	-2.28145	AT5G03240.3	5.00E-81	UBQ3 (POLYUBIQUITIN 3); protein binding
Cit.12276.1.S1_at	306.0208	698.2632	-2.28175	AT5G28020.6	1.00E-140	CYSD2 (CYSTEINE SYNTHASE D2); catalytic/ cysteine synthase/ pyrid
Cit.17589.1.S1_x_at	768.4152	1755.619	-2.28473	AT4G02380.1	8.00E-15	SAG21 (SENESCENCE-ASSOCIATED GENE 21)
Cit.4522.1.S1_at	119.5653	273.3642	-2.28632	AT5G38050.1	3.00E-71	unknown protein
Cit.4715.1.S1_at	78.80083	180.1867	-2.28661	AT4G37550.1	1.00E-110	formamidase, putative / formamide amidohydrolase, putative
Cit.326.1.S1_at	265.2585	607.2369	-2.28923	AT2G37170.1	1.00E-137	PIP2B (PLASMA MEMBRANE INTRINSIC PROTEIN 2); water channel
Cit.8302.1.S1_x_at	29.92418	68.56554	-2.29131		NA	
Cit.28642.1.S1_x_at	20.46772	46.91373	-2.29208		NA	
Cit.9864.1.S1_at	56.2736	129.1583	-2.29518	AT1G02840.3	1.00E-32	SR1; RNA binding / nucleic acid binding / nucleotide binding
Cit.1779.1.S1_s_at	170.2247	390.8302	-2.29597	AT2G29500.1	4.00E-59	17.6 kDa class I small heat shock protein (HSP17.6B-CI)
Cit.30695.1.S1_s_at	176.2418	404.7335	-2.29647	AT3G21420.1	1.00E-151	oxidoreductase, 2OG-Fe(II) oxygenase family protein
Cit.26655.1.S1_at	687.2293	1579.417	-2.29824	AT1G65660.1	3.00E-35	SMP1 (SWELLMAP 1); nucleic acid binding / single-stranded RNA bir
Cit.26039.1.S1_x_at	760.6833	1748.62	-2.29875	AT5G03300.1	2.00E-17	ADK2 (ADENOSINE KINASE 2); adenosine kinase/ copper ion binding
Cit.11658.1.S1_at	47.06799	108.2937	-2.30079	AT2G04700.1	5.00E-59	ferredoxin thioredoxin reductase catalytic beta chain family protein
Cit.20742.1.S1_s_at	229.7879	529.9112	-2.30609	AT5G64140.1	2.00E-26	RPS28 (RIBOSOMAL PROTEIN S28); structural constituent of ribosom
Cit.1779.1.S1_at	162.7323	375.4549	-2.30719	AT2G29500.1	4.00E-59	17.6 kDa class I small heat shock protein (HSP17.6B-CI)
Cit.17977.1.S1_at	82.0817	189.5407	-2.30917	AT5G23960.1	1.00E-121	TPS21 (TERPENE SYNTHASE 21); (-)-E-beta-caryophyllene synthase/
Cit.10547.1.S1_s_at	112.6622	260.316	-2.31059	AT1G44350.1	5.00E-97	ILL6; IAA-amino acid conjugate hydrolase/ metallopeptidase
Cit.19590.1.S1_x_at	559.7311	1293.799	-2.31147	AT3G23920.1	8.00E-25	BAM1 (BETA-AMYLASE 1); beta-amylase
Cit.12735.1.S1_at	49.41587	114.2723	-2.31246		NA	
Cit.3388.1.S1_at	1387.682	3211.655	-2.3144	AT1G17100.1	1.00E-35	SOUL heme-binding family protein
Cit.17150.1.S1_at	32.60947	75.5249	-2.31604	AT3G03470.1	4.00E-33	CYP89A9; electron carrier/ heme binding / iron ion binding / monoo
Cit.30539.1.S1_at	31.18334	72.25351	-2.31705	AT2G45590.1	8.00E-68	protein kinase family protein
Cit.22771.1.S1_x_at	32.02167	74.20054	-2.3172		NA	
Cit.14405.1.S1_at	615.0717	1429.53	-2.32417		NA	
Cit.21635.1.S1_at	25.95813	60.33515	-2.32433		NA	
Cit.9432.1.S1_at	40.06719	93.16985	-2.32534	AT5G20290.1	1.00E-105	40S ribosomal protein S8 (RPS8A)
Cit.29350.1.S1_at	116.6283	271.2367	-2.32565	AT2G16385.1	1.00E-13	unknown protein
Cit.17091.1.S1_at	539.9809	1256.138	-2.32626		NA	
Cit.11226.1.S1_at	93.84579	218.6853	-2.33026	AT3G26330.1	1.00E-150	CYP71B37; electron carrier/ heme binding / iron ion binding / mono
Cit.7372.1.S1_at	42.5868	99.32835	-2.33237	AT4G24060.1	5.00E-05	Dof-type zinc finger domain-containing protein
Cit.27401.1.S1_at	21.96893	51.24194	-2.33247		NA	
Cit.31167.1.S1_at	42.41011	98.95761	-2.33335	AT2G25737.1	7.00E-33	unknown protein

Cit.5818.1.S1_at	22.39396	52.38014	-2.33903	AT1G53708.1	9.00E-16	RTFL9 (ROTUNDIFOLIA LIKE 9)
Cit.850.1.S1_at	22.64233	52.99503	-2.34053		NA	
Cit.17512.1.S1_at	54.77942	128.3436	-2.34292		NA	
Cit.3817.1.S1_at	101.7891	238.5227	-2.3433	AT4G22920.1	1.00E-99	NYE1 (NON-YELLOWING 1)
Cit.6858.1.S1_at	30.67904	71.90661	-2.34384		NA	
Cit.36313.1.S1_at	21.6003	50.62917	-2.34391		NA	
Cit.8310.1.S1_x_at	478.1163	1121.662	-2.346	AT1G07070.1	8.00E-57	60S ribosomal protein L35a (RPL35aA)
Cit.21506.1.S1_s_at	1171.295	2752.981	-2.35037		NA	
Cit.4085.1.S1_at	26.6416	62.71903	-2.35418	AT4G11240.1	7.00E-83	TOPP7; protein serine/threonine phosphatase
Cit.7366.1.S1_at	20.12676	47.38209	-2.35418		NA	
Cit.14195.1.S1_at	76.32005	180.075	-2.35947	AT5G57170.1	2.00E-48	macrophage migration inhibitory factor family protein / MIF family p
Cit.599.1.S1_at	32.37317	76.48239	-2.36252	AT3G50000.1	6.00E-14	CKA2 (CASEIN KINASE II, ALPHA CHAIN 2); kinase
Cit.28011.1.S1_x_at	332.9964	786.7671	-2.36269	AT5G47550.1	4.00E-25	cysteine protease inhibitor, putative / cystatin, putative
Cit.28144.1.S1_at	150.9625	356.9392	-2.36442	AT1G17020.1	3.00E-63	SRG1 (SENESCENCE-RELATED GENE 1); oxidoreductase, acting on dip
Cit.40475.1.S1_at	118.2324	279.8875	-2.36727		NA	
Cit.26985.1.S1_at	473.4868	1121.444	-2.36848	AT5G62790.1	2.00E-14	DXR (1-DEOXY-D-XYLULOSE 5-PHOSPHATE REDUCTOISOMERASE); 1-
Cit.4776.1.S1_at	46.70251	110.6209	-2.36863	AT4G24026.1	5.00E-05	unknown protein
Cit.12872.1.S1_at	111.5551	264.6142	-2.37205	AT2G39110.1	1.00E-161	protein kinase, putative
Cit.5325.1.S1_at	248.1848	589.2433	-2.37421	AT5G60960.1	3.00E-43	pentatricopeptide (PPR) repeat-containing protein
Cit.4067.1.S1_at	57.85052	137.3521	-2.37426	AT3G59800.1	3.00E-72	unknown protein
Cit.7643.1.S1_at	28.52887	67.827	-2.37749		NA	
Cit.39356.1.S1_at	156.1228	371.238	-2.37786		NA	
Cit.11933.1.S1_at	1891.125	4497.058	-2.37798		NA	
Cit.18014.1.S1_at	901.3616	2145.789	-2.38061	AT2G31670.1	2.00E-49	FUNCTIONS IN: molecular_function unknown; INVOLVED IN: biologi
Cit.19507.1.S1_s_at	26.62174	63.39933	-2.38149		NA	
Cit.3329.1.S1_at	21.28335	50.71125	-2.38267	AT5G51120.1	5.00E-93	PABN1 (POLYADENYLATE-BINDING PROTEIN 1); RNA binding / poly(A
Cit.814.1.S1_s_at	359.3931	856.4253	-2.38298	AT3G01500.3	1.00E-134	CA1 (CARBONIC ANHYDRASE 1); carbonate dehydratase/ zinc ion bin
Cit.11675.1.S1_at	20.37763	48.59159	-2.38456	AT5G27410.1	1.00E-72	aminotransferase class IV family protein
Cit.6447.1.S1_at	37.93316	90.47745	-2.38518	AT3G48660.1	2.00E-29	unknown protein
Cit.21300.1.S1_at	29.11276	69.47181	-2.3863		NA	
Cit.36637.1.S1_s_at	20.56709	49.09688	-2.38716	AT5G07300.1	3.00E-70	BON2 (BONZAI 2); calcium-dependent phospholipid binding
Cit.16732.1.S1_at	43.291	103.5237	-2.39134	AT2G26890.2	9.00E-16	GRV2 (GRAVITROPISM DEFECTIVE 2); binding / heat shock protein b
Cit.10188.1.S1_at	27.52846	65.87496	-2.39298	AT3G17000.1	3.00E-25	UBC32 (ubiquitin-conjugating enzyme 32); ubiquitin-protein ligase
Cit.2419.1.S1_at	21.68996	51.9283	-2.39412	AT5G37510.2	1.00E-128	EMB1467 (embryo defective 1467); NADH dehydrogenase (ubiquinc
Cit.26565.1.S1_at	80.37015	192.5121	-2.39532		NA	

Cit.22363.1.S1_x_at	43.0808	103.1995	-2.39549	AT1G52340.1	5.00E-06	ABA2 (ABA DEFICIENT 2); alcohol dehydrogenase/ oxidoreductase/ x
Cit.758.1.S1_at	242.5374	581.2818	-2.39667		NA	
Cit.28805.1.S1_at	65.01902	155.8885	-2.39758		NA	
Cit.13250.1.S1_at	520.0516	1247.131	-2.39809	AT4G12290.1	0	amine oxidase/ copper ion binding / quinone binding
Cit.5925.1.S1_at	29.43121	70.60434	-2.39896	AT5G59050.1	1.00E-37	unknown protein
Cit.19309.1.S1_at	776.3213	1863.385	-2.40028		NA	
Cit.5027.1.S1_at	202.3152	485.7266	-2.40084	AT3G06440.1	1.00E-124	galactosyltransferase family protein
Cit.9511.1.S1_x_at	113.9442	273.6013	-2.40119	AT2G30860.1	4.00E-85	ATGSTF9 (GLUTATHIONE S-TRANSFERASE PHI 9); copper ion binding
Cit.21717.1.S1_at	21.79177	52.36934	-2.40317	AT3G04720.1	2.00E-43	PR4 (PATHOGENESIS-RELATED 4); chitin binding
Cit.20502.1.S1_at	46.82813	112.659	-2.4058		NA	
Cit.21646.1.S1_at	57.15662	137.5198	-2.40602	AT1G28680.1	8.00E-24	transferase family protein
Cit.32937.1.S1_at	242.1206	582.8868	-2.40742		NA	
Cit.13348.1.S1_at	71.54958	172.3175	-2.40836	AT1G66510.2	3.00E-87	AAR2 protein family
Cit.15018.1.S1_at	128.2528	308.9552	-2.40895	AT4G10270.1	2.00E-22	wound-responsive family protein
Cit.3788.1.S1_at	25.6487	61.78749	-2.40899	AT4G21450.1	1.00E-98	vesicle-associated membrane family protein / VAMP family protein
Cit.30651.1.S1_at	28.0371	67.54307	-2.40906	AT4G34590.1	2.00E-37	GBF6 (G-BOX BINDING FACTOR 6); DNA binding / protein heterodim
Cit.20929.1.S1_at	234.3044	564.6052	-2.40971	AT5G03560.1	1.00E-10	nucleobase:cation symporter
Cit.20005.1.S1_at	34.92167	84.15266	-2.40975		NA	
Cit.6162.1.S1_at	40.64473	98.00958	-2.41137		NA	
Cit.21774.1.S1_at	20.15896	48.62441	-2.41205	AT1G52560.2	7.00E-06	26.5 kDa class I small heat shock protein-like (HSP26.5-P)
Cit.26070.1.S1_at	442.19	1066.996	-2.41298		NA	
Cit.14937.1.S1_at	62.96399	151.9524	-2.41332		NA	
Cit.25649.1.S1_at	36.49699	88.10355	-2.41399	AT1G47128.1	2.00E-27	RD21 (responsive to dehydration 21); cysteine-type endopeptidase/
Cit.1148.1.S1_at	38.28441	92.48873	-2.41583	AT4G12560.2	3.00E-19	F-box family protein
Cit.11676.1.S1_at	47.30211	114.28	-2.41596		NA	
Cit.30332.1.S1_at	22.73948	54.9703	-2.41739	AT5G06750.3	1.00E-25	protein phosphatase 2C family protein / PP2C family protein
Cit.26141.1.S1_s_at	351.6162	850.1396	-2.41781		NA	
Cit.4868.1.S1_at	156.4689	379.2278	-2.42366	AT3G15840.1	3.00E-99	PIFI (post-illumination chlorophyll fluorescence increase)
Cit.9707.1.S1_at	381.2728	924.8337	-2.42565	AT4G02620.1	2.00E-52	vacuolar ATPase subunit F family protein
Cit.25156.1.S1_x_at	22.93683	55.64619	-2.42606		NA	
Cit.28659.1.S1_at	40.32103	97.92099	-2.42853	AT1G80910.1	2.00E-74	unknown protein
Cit.25099.1.S1_at	25.67952	62.37169	-2.42885	AT1G12280.1	1.00E-19	disease resistance protein (CC-NBS-LRR class), putative
Cit.15769.1.S1_at	36.51838	88.77506	-2.43097		NA	
Cit.16390.1.S1_at	26.33808	64.0752	-2.4328	AT3G56230.1	2.00E-59	speckle-type POZ protein-related
Cit.16662.1.S1_at	28.10514	68.39005	-2.43336	AT3G21430.2	1.00E-81	DNA binding

Cit.23463.1.S1_at	77.43604	188.7419	-2.43739	AT4G01070.1	6.00E-76	GT72B1; UDP-glucosyltransferase/ UDP-glycosyltransferase/ transfe
Cit.8950.1.S1_at	103.7456	252.8971	-2.43767	AT4G39780.1	2.00E-09	AP2 domain-containing transcription factor, putative
Cit.2180.1.S1_at	621.9716	1516.262	-2.43783	AT1G77350.2	5.00E-49	unknown protein
Cit.24945.1.S1_at	30.69617	74.83769	-2.43801	AT4G16490.1	1.00E-16	binding
Cit.18021.1.S1_s_at	968.5411	2361.896	-2.43861	AT2G01460.1	2.00E-74	ATP binding / kinase
Cit.30583.1.S1_at	36.50859	89.10191	-2.44057	AT4G33865.1	7.00E-27	40S ribosomal protein S29 (RPS29C)
Cit.7435.1.S1_at	21.15038	51.63437	-2.4413		NA	
Cit.28755.1.S1_at	38.27526	93.46586	-2.44194	AT3G07950.1	8.00E-18	rhomboid protein-related
Cit.25439.1.S1_at	35.90028	87.71603	-2.44332	AT3G02050.1	1.00E-16	KUP3 (K+ UPTAKE TRANSPORTER 3); potassium ion transmembrane
Cit.12237.1.S1_at	374.8986	916.0436	-2.44344	AT2G17410.1	2.00E-87	ARID/BRIGHT DNA-binding domain-containing protein
Cit.3036.1.S1_s_at	275.389	673.0965	-2.44417	AT3G04920.1	9.00E-63	40S ribosomal protein S24 (RPS24A)
Cit.37465.1.S1_at	38.67168	94.67476	-2.44817	AT1G75720.1	3.00E-19	unknown protein
Cit.22078.1.S1_at	25.85382	63.39973	-2.45224		NA	
Cit.2905.1.S1_at	60.86034	149.3579	-2.45411	AT1G31330.1	5.00E-96	PSAF (photosystem I subunit F)
Cit.20364.1.S1_at	1081.426	2659.138	-2.45892		NA	
Cit.21933.1.S1_at	34.7647	85.48631	-2.459		NA	
Cit.21495.1.S1_at	121.6967	299.2584	-2.45905		NA	
Cit.10975.1.S1_at	24.64873	60.68194	-2.46187		NA	
Cit.36252.1.S1_at	43.79829	107.8274	-2.46191		NA	
Cit.15643.1.S1_at	53.98048	133.0499	-2.46478	AT4G31940.1	8.00E-59	CYP82C4; electron carrier/ heme binding / iron ion binding / monoo
Cit.39331.1.S1_at	53.52051	132.0106	-2.46654		NA	
Cit.20650.1.S1_at	60.39902	149.3716	-2.47308		NA	
Cit.24325.1.S1_at	56.04432	138.6089	-2.4732	AT1G80260.1	6.00E-39	emb1427 (embryo defective 1427); tubulin binding
Cit.3846.1.S1_at	211.3372	522.7181	-2.47338	AT1G09920.1	2.00E-75	TRAF-type zinc finger-related
Cit.10594.1.S1_at	37.87456	93.70069	-2.47397	AT3G54420.1	3.00E-66	ATEP3; chitinase
Cit.3042.1.S1_s_at	116.0588	287.1564	-2.47423	AT1G52565.1	1.00E-31	unknown protein
Cit.20133.1.S1_at	20.72093	51.37202	-2.47923		NA	
Cit.28073.1.S1_at	598.9386	1486.571	-2.48201		NA	
Cit.30644.1.S1_at	114.9988	285.4968	-2.48261	AT5G43970.1	2.00E-23	TOM22-V (TRANSLOCASE OF OUTER MEMBRANE 22-V); P-P-bond-h
Cit.57.1.S1_at	364.716	906.7656	-2.48622	AT1G17860.1	2.00E-27	trypsin and protease inhibitor family protein / Kunitz family protein
Cit.17451.1.S1_x_at	115.85	288.5324	-2.49057		NA	
Cit.8423.1.S1_s_at	417.7329	1040.924	-2.49184	AT5G20700.1	2.00E-37	senescence-associated protein-related
Cit.5936.1.S1_at	37.17234	92.65786	-2.49266	AT4G30470.1	1.00E-123	cinnamoyl-CoA reductase-related
Cit.13124.1.S1_at	78.61594	196.1129	-2.49457	AT5G17870.1	2.00E-22	PSRP6 (PLASTID-SPECIFIC 50S RIBOSOMAL PROTEIN 6)
Cit.13378.1.S1_at	60.1079	150.0146	-2.49576		NA	

Cit.20742.1.S1_at	27.15681	67.7823	-2.49596	AT5G64140.1	1.00E-13	RPS28 (RIBOSOMAL PROTEIN S28); structural constituent of ribosom
Cit.32809.1.S1_at	486.4838	1220.036	-2.50787		NA	
Cit.34286.1.S1_at	48.49127	121.7083	-2.5099		NA	
Cit.9568.1.S1_s_at	2354.934	5913.335	-2.51104	AT5G64260.1	1.00E-125	EXL2 (EXORDIUM LIKE 2)
Cit.30439.1.S1_x_at	73.40242	185.1335	-2.52217	AT3G05890.1	5.00E-20	RCI2B (RARE-COLD-INDUCIBLE 2B)
Cit.5757.1.S1_at	62.40679	157.5458	-2.5245	AT3G53980.2	2.00E-47	protease inhibitor/seed storage/lipid transfer protein (LTP) family p
Cit.15391.1.S1_at	30.21045	76.36799	-2.52787		NA	
Cit.30190.1.S1_at	80.01517	202.4208	-2.52978	AT2G20515.1	3.00E-47	unknown protein
Cit.9985.1.S1_at	34.4197	87.07457	-2.52979	AT1G56220.3	5.00E-41	dormancy/auxin associated family protein
Cit.28249.1.S1_at	34.85795	88.47698	-2.53822	AT1G13360.1	6.00E-22	unknown protein
Cit.17832.1.S1_at	155.253	394.3719	-2.54019		NA	
Cit.13693.1.S1_s_at	67.07517	170.4664	-2.54142	AT4G38960.1	6.00E-28	zinc finger (B-box type) family protein
Cit.27861.1.S1_at	31.80997	80.8929	-2.543		NA	
Cit.18219.1.S1_at	32.51655	82.73698	-2.54446		NA	
Cit.2306.1.S1_at	266.4599	678.6611	-2.54695		NA	
Cit.7486.1.S1_at	33.92843	86.4296	-2.54741		NA	
Cit.15739.1.S1_at	149.1882	380.2626	-2.54888	AT3G06895.1	2.00E-08	unknown protein
Cit.9171.1.S1_at	1049.547	2676.879	-2.55051	AT4G37980.1	2.00E-58	ELI3-1 (ELICITOR-ACTIVATED GENE 3-1); binding / catalytic/ oxidore
Cit.20284.1.S1_at	21.06854	53.75874	-2.55161	AT1G68640.1	6.00E-70	PAN (PERIANTHIA); DNA binding / transcription factor
Cit.17116.1.S1_at	177.1179	452.1614	-2.55288		NA	
Cit.22526.1.S1_at	28.7101	73.32384	-2.55394	AT2G36460.2	8.00E-17	fructose-bisphosphate aldolase, putative
Cit.11865.1.S1_at	128.9957	329.7347	-2.55617	AT4G12780.1	4.00E-69	heat shock protein binding
Cit.29259.1.S1_at	137.9966	352.9753	-2.55786	AT5G59320.1	5.00E-07	LTP3 (LIPID TRANSFER PROTEIN 3); lipid binding
Cit.3454.1.S1_at	327.0043	837.5527	-2.56129		NA	
Cit.8163.1.S1_x_at	913.3592	2341.687	-2.56382	AT5G47550.1	2.00E-23	cysteine protease inhibitor, putative / cystatin, putative
Cit.18409.1.S1_at	38.18502	97.93789	-2.56482		NA	
Cit.4552.1.S1_at	20.98967	53.86597	-2.56631	AT2G27880.1	1.00E-119	AGO5 (ARGONAUTE 5); nucleic acid binding
Cit.21182.1.S1_at	63.87595	163.9622	-2.56688		NA	
Cit.22641.1.S1_at	31.79221	81.62131	-2.56734	AT2G03220.1	1.00E-66	FT1 (FUCOSYLTRANSFERASE 1); fucosyltransferase/ transferase, trar
Cit.12593.1.S1_at	38.18707	98.04924	-2.5676	AT1G52560.1	4.00E-73	26.5 kDa class I small heat shock protein-like (HSP26.5-P)
Cit.25075.1.S1_s_at	24.40657	62.80271	-2.57319	AT1G18880.1	4.00E-72	proton-dependent oligopeptide transport (POT) family protein
Cit.38319.1.S1_s_at	24.21473	62.38084	-2.57615	AT5G23960.1	1.00E-121	TPS21 (TERPENE SYNTHASE 21); (-)-E-beta-caryophyllene synthase/
Cit.2326.1.S1_at	35.07622	90.8484	-2.59003	AT5G55610.1	7.00E-24	unknown protein
Cit.26670.1.S1_at	110.2064	286.8668	-2.603	AT3G25830.1	1.00E-51	ATTPS-CIN (terpene synthase-like sequence-1,8-cineole); (E)-beta-o
Cit.20890.1.S1_at	21.93986	57.16631	-2.60559		NA	

Cit.175.1.S1_x_at	842.4672	2196.363	-2.60706	AT4G05050.3	1.00E-125	UBQ11 (UBIQUITIN 11); protein binding
Cit.7123.1.S1_at	30.41673	79.30102	-2.60715		NA	
Cit.573.1.S1_at	38.08311	99.32554	-2.60813	AT4G05320.6	0	UBQ10 (POLYUBIQUITIN 10); protein binding
Cit.8023.1.S1_at	63.69455	166.166	-2.60879	AT3G12580.1	0	HSP70 (heat shock protein 70); ATP binding
Cit.308.1.S1_at	111.0473	289.7317	-2.60908		NA	
Cit.17266.1.S1_at	491.0995	1282.054	-2.61058	AT5G23960.1	1.00E-41	TPS21 (TERPENE SYNTHASE 21); (-)-E-beta-caryophyllene synthase/
Cit.28519.1.S1_at	20.72063	54.13507	-2.61262		NA	
Cit.26050.1.S1_s_at	201.1212	526.2521	-2.61659		NA	
Cit.8866.1.S1_x_at	55.11712	144.3189	-2.6184	AT4G26850.1	0	VTC2 (vitamin c defective 2); GDP-D-glucose phosphorylase/ GDP-ga
Cit.30153.1.S1_at	43.78763	114.6653	-2.61867		NA	
Cit.11236.1.S1_at	33.18208	87.12151	-2.62556	AT2G30570.1	1.00E-35	PSBW (PHOTOSYSTEM II REACTION CENTER W)
Cit.234.1.S1_at	294.9279	774.9863	-2.62771	AT3G23810.1	6.00E-29	SAHH2 (S-ADENOSYL-L-HOMOCYSTEINE (SAH) HYDROLASE 2); adenc
Cit.35987.1.S1_at	22.45273	59.00051	-2.62777		NA	
Cit.23697.1.S1_at	22.73833	60.02708	-2.63991	AT4G16390.1	1.00E-19	LOCATED IN: chloroplast; EXPRESSED IN: 22 plant structures; EXPRES
Cit.28604.1.S1_at	32.85955	86.88178	-2.64403		NA	
Cit.24576.1.S1_at	27.01627	71.45895	-2.64503	AT3G55620.1	1.00E-104	emb1624 (embryo defective 1624); ribosome binding / translation i
Cit.398.1.S1_at	3144.942	8327.921	-2.64804	AT3G13920.1	0	EIF4A1 (EUKARYOTIC TRANSLATION INITIATION FACTOR 4A1); ATP-c
Cit.22666.1.S1_at	30.64801	81.22887	-2.65038	AT1G78960.1	4.00E-66	ATLUP2; beta-amyrin synthase/ lupeol synthase
Cit.28604.1.S1_x_at	26.99059	71.6003	-2.65279		NA	
Cit.23524.1.S1_at	23.06873	61.25822	-2.65547		NA	
Cit.22152.1.S1_at	21.6293	57.57245	-2.66178	AT5G03240.3	2.00E-37	UBQ3 (POLYUBIQUITIN 3); protein binding
Cit.33468.1.S1_at	163.539	435.6599	-2.66395	AT5G54760.2	9.00E-10	eukaryotic translation initiation factor SUI1, putative
Cit.20314.1.S1_at	68.11729	181.6725	-2.66705	AT2G38020.1	0	VCL1 (VACUOLELESS 1)
Cit.17117.1.S1_at	796.28	2126.611	-2.67068		NA	
Cit.21392.1.S1_at	282.3746	757.9756	-2.68429		NA	
Cit.829.1.S1_at	100.3994	269.7088	-2.68636	AT3G18280.1	2.00E-25	protease inhibitor/seed storage/lipid transfer protein (LTP) family p
Cit.12371.1.S1_at	39.80904	106.9579	-2.68677	AT4G37090.2	6.00E-14	unknown protein
Cit.19375.1.S1_s_at	51.33775	137.9982	-2.68805	AT2G01570.1	4.00E-26	RGA1 (REPRESSOR OF GA1-3 1); protein binding / transcription facto
Cit.12539.1.S1_at	183.1952	492.5514	-2.68867	AT1G18100.1	1.00E-54	E12A11; phosphatidylethanolamine binding
Cit.10295.1.S1_at	285.668	768.2814	-2.68942	AT1G32230.2	3.00E-12	RCD1 (RADICAL-INDUCED CELL DEATH1); protein binding
Cit.18219.1.S1_x_at	31.04275	83.50217	-2.68991		NA	
Cit.12814.1.S1_s_at	212.3344	572.1914	-2.69477	AT5G07330.1	2.00E-36	unknown protein
Cit.3690.1.S1_at	65.56155	176.8882	-2.69805	AT1G67785.1	7.00E-23	unknown protein
Cit.28694.1.S1_at	51.67502	139.4716	-2.69901	AT2G36830.1	1.00E-39	GAMMA-TIP (GAMMA TONOPLAST INTRINSIC PROTEIN); water char
Cit.29691.1.S1_at	20.83857	56.27568	-2.70055		NA	

Cit.20948.1.S1_at	45.21656	122.4691	-2.7085		NA	
Cit.3754.1.S1_at	64.67651	176.1006	-2.72279	AT1G53320.1	1.00E-155	AtTLP7 (TUBBY LIKE PROTEIN 7); phosphoric diester hydrolase/ tran
Cit.36128.1.S1_at	26.53485	72.25008	-2.72284		NA	
Cit.7791.1.S1_at	26.68808	72.70402	-2.72421		NA	
Cit.28860.1.S1_at	73.89974	201.3468	-2.72459	AT3G12580.1	7.00E-13	HSP70 (heat shock protein 70); ATP binding
Cit.21417.1.S1_at	45.73104	124.6054	-2.72474	AT2G31141.1	1.00E-05	unknown protein
Cit.36158.1.S1_at	20.99249	57.42181	-2.73535		NA	
Cit.1870.1.S1_s_at	85.75751	234.8004	-2.73796	AT2G17880.1	3.00E-24	DNAJ heat shock protein, putative
Cit.20276.1.S1_at	37.89417	103.8353	-2.74014		NA	
Cit.29123.1.S1_at	79.65578	218.4103	-2.74193		NA	
Cit.37064.1.S1_at	21.03176	57.69882	-2.74341		NA	
Cit.29266.1.S1_at	25.33314	69.56769	-2.74611	AT3G24160.1	4.00E-12	PMP (PUTATIVE TYPE 1 MEMBRANE PROTEIN)
Cit.18677.1.S1_s_at	53.29909	146.604	-2.75059	AT3G54420.1	6.00E-76	ATEP3; chitinase
Cit.22853.1.S1_x_at	20.34533	56.03264	-2.75408	AT2G46540.1	2.00E-19	unknown protein
Cit.16933.1.S1_x_at	1961.156	5404.292	-2.75567		NA	
Cit.36284.1.S1_at	23.19653	63.93646	-2.75629	AT1G54170.1	2.00E-14	CID3 (CTC-Interacting Domain 3)
Cit.6308.1.S1_at	23.48732	64.7401	-2.75639	AT2G29420.1	9.00E-60	ATGSTU7 (ARABIDOPSIS THALIANA GLUTATHIONE S-TRANSFERASE T
Cit.15375.1.S1_at	20.43298	56.40128	-2.76031		NA	
Cit.12261.1.S1_s_at	42.11047	116.4499	-2.76534	AT1G73300.1	1.00E-113	scpl2 (serine carboxypeptidase-like 2); serine-type carboxypeptidase
Cit.19826.1.S1_s_at	519.1091	1439.833	-2.77366	AT1G10740.2	1.00E-37	unknown protein
Cit.13644.1.S1_at	29.89674	82.9337	-2.774		NA	
Cit.8027.1.S1_s_at	27.15283	75.42318	-2.77773	AT3G12580.1	0	HSP70 (heat shock protein 70); ATP binding
Cit.19552.1.S1_at	23.92297	66.55047	-2.78186	AT1G75290.1	4.00E-35	oxidoreductase, acting on NADH or NADPH
Cit.7937.1.S1_at	49.63407	138.1583	-2.78354	AT5G01900.1	4.00E-09	WRKY62; transcription factor
Cit.3122.1.S1_at	51.92243	144.923	-2.79114	AT5G54680.1	7.00E-89	ILR3 (iaa-leucine resistant3); DNA binding / transcription factor
Cit.19942.1.S1_x_at	55.81669	155.9014	-2.7931		NA	
Cit.7100.1.S1_x_at	193.7276	543.0179	-2.803		NA	
Cit.21222.1.S1_x_at	1075.309	3015.052	-2.80389	AT3G15353.1	2.00E-17	MT3 (METALLOTHIONEIN 3); copper ion binding
Cit.31428.1.S1_at	35.24844	98.84866	-2.80434		NA	
Cit.13271.1.S1_at	180.3273	506.4665	-2.8086	AT1G59950.1	2.00E-96	aldo/keto reductase, putative
Cit.8293.1.S1_at	24.35612	68.40762	-2.80864	AT5G22875.2	3.00E-28	unknown protein
Cit.25173.1.S1_at	23.47098	65.9581	-2.8102		NA	
Cit.10776.1.S1_at	54.12499	152.2706	-2.81331	AT5G19760.1	2.00E-68	dicarboxylate/tricarboxylate carrier (DTC)
Cit.20522.1.S1_x_at	199.7757	563.5739	-2.82103		NA	
Cit.8595.1.S1_s_at	1849.974	5228.334	-2.82617	AT4G32940.1	0	GAMMA-VPE (GAMMA VACUOLAR PROCESSING ENZYME); cysteine-

Cit.15359.1.S1_at	186.9205	529.2363	-2.83134	AT4G00170.1	9.00E-51	vesicle-associated membrane family protein / VAMP family protein
Cit.6467.1.S1_s_at	535.5162	1523.034	-2.84405		NA	
Cit.24046.1.S1_x_at	29.65262	84.40247	-2.84637	AT1G14900.1	5.00E-50	HMGA (HIGH MOBILITY GROUP A); DNA binding
Cit.5112.1.S1_at	27.05195	77.08492	-2.84951	AT1G47128.1	1.00E-118	RD21 (responsive to dehydration 21); cysteine-type endopeptidase/
Cit.22614.1.S1_at	206.1772	587.8983	-2.85142	AT1G66150.1	2.00E-31	TMK1 (TRANSMEMBRANE KINASE 1); transmembrane receptor prot
Cit.20946.1.S1_at	37.34579	106.6058	-2.85456	AT5G43050.1	8.00E-53	unknown protein
Cit.18085.1.S1_s_at	370.7942	1058.481	-2.85463	AT4G30330.1	5.00E-42	small nuclear ribonucleoprotein E, putative / snRNP-E, putative / Sm
Cit.8444.1.S1_s_at	821.19	2346.283	-2.85717	AT3G46230.1	8.00E-70	ATHSP17.4
Cit.25881.1.S1_at	44.56963	127.567	-2.8622	AT5G03240.3	3.00E-20	UBQ3 (POLYUBIQUITIN 3); protein binding
Cit.30516.1.S1_at	20.98952	60.22293	-2.86919	AT2G40610.1	6.00E-48	ATEXPA8 (ARABIDOPSIS THALIANA EXPANSIN A8)
Cit.22710.1.S1_at	1045.855	3003.24	-2.87156		NA	
Cit.3370.1.S1_at	56.61296	162.8521	-2.87659	AT5G60980.1	4.00E-59	nuclear transport factor 2 (NTF2) family protein / RNA recognition m
Cit.3120.1.S1_s_at	45.78054	133.1455	-2.90834	AT1G77120.1	0	ADH1 (ALCOHOL DEHYDROGENASE 1); alcohol dehydrogenase
Cit.28289.1.S1_at	68.89423	200.7709	-2.91419	AT3G23450.1	2.00E-40	unknown protein
Cit.7793.1.S1_at	85.22746	248.609	-2.91701		NA	
Cit.9253.1.S1_at	93.1076	271.9696	-2.92102	AT2G38870.1	4.00E-18	protease inhibitor, putative
Cit.15486.1.S1_at	87.37765	255.6523	-2.92583	AT3G10740.1	4.00E-08	ASD1 (ALPHA-L-ARABINOFURANOSIDASE 1); alpha-N-arabinofurano
Cit.16894.1.S1_x_at	4363.041	12793.48	-2.93224		NA	
Cit.17057.1.S1_at	97.89565	287.8799	-2.94068	AT5G24165.1	1.00E-20	unknown protein
Cit.6583.1.S1_at	38.82476	114.1743	-2.94076	AT4G00980.1	3.00E-24	zinc knuckle (CCHC-type) family protein
Cit.12982.1.S1_x_at	597.7192	1760.588	-2.94551	AT2G17230.1	4.00E-99	EXL5 (EXORDIUM LIKE 5)
Cit.15773.1.S1_at	35.32702	104.2048	-2.94972		NA	
Cit.19893.1.S1_at	25.91463	76.4969	-2.95188		NA	
Cit.12503.1.S1_at	204.77	604.7736	-2.95343	AT3G45600.1	1.00E-133	TET3 (TETRASPANIN3)
Cit.20174.1.S1_x_at	20.23437	59.93868	-2.96222		NA	
Cit.12818.1.S1_at	39.78318	119.043	-2.99229	AT5G48930.1	1.00E-121	HCT (HYDROXYCINNAMOYL-COA SHIKIMATE/QUINATE HYDROXYCIN
Cit.26323.1.S1_at	48.58719	145.4148	-2.99286	AT5G58740.1	1.00E-05	nuclear movement family protein
Cit.29267.1.S1_x_at	71.55069	214.4741	-2.99751	AT3G44735.1	4.00E-10	PSK1; growth factor
Cit.21182.1.S1_s_at	140.201	420.6983	-3.00068	AT1G62730.1	1.00E-48	transferase
Cit.22710.1.S1_x_at	70.50534	211.783	-3.00379	AT4G11650.1	9.00E-25	ATOSM34 (osmotin 34)
Cit.1555.1.S1_at	199.4028	599.5457	-3.00671	AT1G27330.1	6.00E-24	FUNCTIONS IN: molecular_function unknown; INVOLVED IN: respon
Cit.13692.1.S1_at	122.8015	369.7198	-3.01071	AT3G26430.1	1.00E-122	GDSL-motif lipase/hydrolase family protein
Cit.24984.1.S1_at	106.3656	320.4636	-3.01285		NA	
Cit.26140.1.S1_at	44.5865	134.3842	-3.01401		NA	
Cit.12866.1.S1_x_at	1116.747	3367.492	-3.01545		NA	

Cit.23550.1.S1_x_at	81.97961	247.2425	-3.0159		NA	
Cit.1225.1.S1_at	877.1034	2645.79	-3.01651		NA	
Cit.20247.1.S1_at	20.04515	60.46815	-3.0166		NA	
Cit.8518.1.S1_at	61.12483	185.2001	-3.02987	AT3G52990.2	1.00E-126	pyruvate kinase, putative
Cit.5117.1.S1_at	42.01779	127.3988	-3.03202	AT3G62550.1	1.00E-53	universal stress protein (USP) family protein
Cit.17912.1.S1_at	30.53032	93.552	-3.06423	AT5G57800.1	3.00E-75	CER3 (ECERIFERUM 3); binding / catalytic/ iron ion binding / oxidore
Cit.8720.1.S1_at	204.0333	625.33	-3.06484	AT5G54160.1	7.00E-35	ATOMT1 (O-METHYLTRANSFERASE 1); caffeate O-methyltransferase
Cit.4770.1.S1_at	20.17232	61.90428	-3.06877	AT4G24340.1	8.00E-55	phosphorylase family protein
Cit.3114.1.S1_at	149.7693	459.9747	-3.07122	AT3G13930.1	1.00E-121	dihydrolipoamide S-acetyltransferase, putative
Cit.9609.1.S1_at	136.6505	421.4535	-3.08417	AT2G03440.1	3.00E-26	nodulin-related
Cit.25527.1.S1_at	55.05525	171.1623	-3.10892	AT3G23410.1	1.00E-46	alcohol oxidase-related
Cit.10673.1.S1_at	42.03214	130.7261	-3.11015	AT3G26740.1	4.00E-29	CCL (CCR-LIKE)
Cit.18367.1.S1_at	64.76807	201.8122	-3.11592	AT4G31300.2	2.00E-11	PBA1; endopeptidase/ peptidase/ threonine-type endopeptidase
Cit.471.1.S1_at	23.19836	72.53696	-3.12681	AT5G03240.3	1.00E-23	UBQ3 (POLYUBIQUITIN 3); protein binding
Cit.1594.1.S1_at	214.9598	676.4293	-3.14677	AT5G17165.1	4.00E-27	unknown protein
Cit.34377.1.S1_at	406.1454	1280.477	-3.15276	ATCG00670.1	3.00E-27	Encodes the only ClpP (caseinolytic protease) encoded within the pl
Cit.23393.1.S1_x_at	56.69861	179.4797	-3.1655	AT5G21160.1	2.00E-24	La domain-containing protein / proline-rich family protein
Cit.25501.1.S1_at	72.24816	229.2936	-3.17369	AT1G66240.1	5.00E-31	ATX1 (ARABIDOPSIS HOMOLOG OF ANTI-OXIDANT 1); metal ion bin
Cit.17997.1.S1_at	33.8989	107.6356	-3.17519	AT3G52090.1	1.00E-53	NRPB11; DNA binding / DNA-directed RNA polymerase
Cit.18200.1.S1_at	43.95043	139.7051	-3.1787	AT4G18060.1	2.00E-18	clathrin binding
Cit.29256.1.S1_at	148.4422	472.1941	-3.181	ATCG00670.1	4.00E-05	Encodes the only ClpP (caseinolytic protease) encoded within the pl
Cit.4932.1.S1_at	25.72871	81.91359	-3.18374	AT5G49660.1	0	leucine-rich repeat transmembrane protein kinase, putative
Cit.11790.1.S1_at	20.14178	64.21271	-3.18804	AT2G46510.1	8.00E-63	ATAIB (ABA-INDUCIBLE BHLH-TYPE TRANSCRIPTION FACTOR); DNA
Cit.28590.1.S1_at	37.90316	120.9215	-3.19027	AT1G76140.1	1.00E-104	serine-type endopeptidase/ serine-type peptidase
Cit.21765.1.S1_s_at	358.9075	1145.037	-3.19034		NA	
Cit.26582.1.S1_at	191.8931	614.0364	-3.19989		NA	
Cit.18533.1.S1_at	128.2594	411.0492	-3.20483		NA	
Cit.8210.1.S1_x_at	61.3674	197.1332	-3.21234	AT1G54410.1	1.00E-19	dehydrin family protein
Cit.36207.1.S1_at	28.61392	92.10362	-3.21884		NA	
Cit.13950.1.S1_at	22.9504	73.94553	-3.22197		NA	
Cit.23291.1.S1_at	66.1335	213.1276	-3.22269	AT5G03240.3	1.00E-74	UBQ3 (POLYUBIQUITIN 3); protein binding
CitAffx.47577.1.S1_at	3476.474	11207.64	-3.22385	NA	NA	NA
Cit.17304.1.S1_at	29.35644	95.09785	-3.23942		NA	
Cit.24893.1.S1_at	24.79037	80.32392	-3.24013	AT4G33660.1	1.00E-07	unknown protein
Cit.18564.1.S1_at	20.34662	65.94296	-3.24098		NA	

Cit.11398.1.S1_at	68.74963	222.9465	-3.24288	AT5G65110.1	0	ACX2 (ACYL-COA OXIDASE 2); acyl-CoA oxidase
Cit.15339.1.S1_at	62.8891	205.7689	-3.27193		NA	
Cit.12794.1.S1_at	34.01119	111.3121	-3.27281	AT1G33140.1	2.00E-90	PGY2 (PIGGYBACK2); structural constituent of ribosome
Cit.22617.1.S1_at	26.95257	88.24177	-3.27396		NA	
Cit.17018.1.S1_s_at	156.0117	511.2552	-3.27703	AT1G23040.1	3.00E-31	hydroxyproline-rich glycoprotein family protein
Cit.9663.1.S1_at	46.26888	151.6778	-3.27818	AT5G38480.2	8.00E-54	GRF3 (GENERAL REGULATORY FACTOR 3); ATP binding / protein bin
Cit.16405.1.S1_at	37.56624	123.4012	-3.2849	AT3G14430.1	2.00E-18	unknown protein
Cit.34907.1.S1_x_at	576.4099	1894.971	-3.28754		NA	
Cit.13667.1.S1_s_at	32.45673	106.8075	-3.29077	AT4G05070.1	3.00E-09	unknown protein
Cit.19526.1.S1_at	25.29346	83.66405	-3.30773		NA	
Cit.1991.1.S1_at	103.4353	346.4547	-3.34948	AT1G14980.1	4.00E-37	CPN10 (CHAPERONIN 10); chaperone binding
Cit.18145.1.S1_at	78.28404	263.4763	-3.36565		NA	
Cit.4990.1.S1_x_at	69.36404	235.2307	-3.39125		NA	
Cit.29302.1.S1_x_at	38.87518	131.8579	-3.39183		NA	
Cit.104.1.S1_at	157.603	534.9666	-3.39439		NA	
Cit.20191.1.S1_x_at	900.1975	3078.465	-3.41977		NA	
Cit.16304.1.S1_at	387.1992	1332.499	-3.44138	AT5G22450.1	3.00E-07	unknown protein
Cit.29715.1.S1_at	22.83575	78.84736	-3.4528		NA	
Cit.2405.1.S1_at	56.53754	195.6438	-3.46042		NA	
Cit.31342.1.S1_at	23.18287	80.48765	-3.47186	AT4G29090.1	8.00E-32	reverse transcriptase, putative / RNA-dependent DNA polymerase, [illegible]
Cit.17413.1.S1_s_at	49.9486	173.475	-3.47307	AT4G33467.2	1.00E-09	unknown protein
Cit.20196.1.S1_x_at	235.1355	817.5258	-3.47683		NA	
Cit.26063.1.S1_x_at	20.29298	70.55703	-3.47692		NA	
Cit.9025.1.S1_x_at	148.1784	515.6563	-3.47997	AT1G03400.1	1.00E-95	2-oxoglutarate-dependent dioxygenase, putative
Cit.30465.1.S1_at	70.19962	245.4432	-3.49636	AT3G10610.1	4.00E-63	40S ribosomal protein S17 (RPS17C)
Cit.30128.1.S1_at	20.83203	73.90011	-3.54743	AT4G27360.1	2.00E-37	dynein light chain, putative
Cit.34778.1.S1_at	27.08787	97.06168	-3.58322	AT5G59190.1	6.00E-32	subtilase family protein
Cit.29438.1.S1_s_at	22.98296	83.62546	-3.63859	AT1G65480.1	5.00E-76	FT (FLOWERING LOCUS T); phosphatidylethanolamine binding / prot
Cit.11423.1.S1_at	41.65829	151.5948	-3.63901	AT4G20070.1	1.00E-111	ATAAH (Arabidopsis thaliana Allantoate Amidohydrolase); allantoat
Cit.12282.1.S1_at	31.77938	116.0922	-3.65307	AT2G25670.2	4.00E-70	unknown protein
Cit.17084.1.S1_at	189.0821	691.4976	-3.65713		NA	
Cit.18491.1.S1_at	288.5714	1057.317	-3.66397		NA	
Cit.1557.1.S1_s_at	788.1061	2891.5	-3.66892		NA	
Cit.18642.1.S1_s_at	43.99136	161.6343	-3.67423	AT1G20030.2	3.00E-79	pathogenesis-related thaumatin family protein
Cit.2928.1.S1_s_at	24.45531	90.08688	-3.68373	AT5G23810.1	1.00E-103	AAP7; amino acid transmembrane transporter

Cit.20784.1.S1_at	27.22385	100.9576	-3.70842		NA	
Cit.22819.1.S1_x_at	82.28017	305.938	-3.71825		NA	
Cit.21911.1.S1_x_at	189.7403	707.6874	-3.72977		NA	
Cit.25118.1.S1_at	24.407	91.17029	-3.73542	AT5G48930.1	8.00E-33	HCT (HYDROXYCINNAMOYL-COA SHIKIMATE/QUINATE HYDROXYCIN
Cit.11745.1.S1_at	40.33918	150.912	-3.74108	AT5G18580.1	1.00E-106	FASS (FASS 1); protein phosphatase type 2B regulator
Cit.21345.1.S1_at	130.7971	490.4489	-3.74969		NA	
Cit.25808.1.S1_x_at	22.92132	86.26189	-3.76339		NA	
Cit.9464.1.S1_at	63.55746	239.5363	-3.76881	AT2G29500.1	1.00E-56	17.6 kDa class I small heat shock protein (HSP17.6B-CI)
Cit.2942.1.S1_at	31.41795	118.4468	-3.77004		NA	
Cit.1021.1.S1_at	30.22726	113.9651	-3.77028	AT5G42190.1	2.00E-70	ASK2 (ARABIDOPSIS SKP1-LIKE 2); protein binding / ubiquitin-protei
Cit.104.1.S1_x_at	183.9915	693.9125	-3.77144		NA	
Cit.14792.1.S1_at	20.17278	76.10668	-3.77274	AT5G52230.1	4.00E-33	MBD13; methyl-CpG binding
Cit.6794.1.S1_s_at	21.83582	82.43705	-3.77531	AT4G35790.1	4.00E-78	ATPLDDELTA; phospholipase D
Cit.25482.1.S1_at	25.20999	95.22921	-3.77744	AT1G18070.2	2.00E-90	EF-1-alpha-related GTP-binding protein, putative
Cit.13054.1.S1_at	150.1859	568.1935	-3.78327	AT1G60390.1	2.00E-08	BURP domain-containing protein / polygalacturonase, putative
Cit.17739.1.S1_at	36.07618	137.7336	-3.81785		NA	
Cit.8501.1.S1_at	65.82022	251.8183	-3.82585		NA	
Cit.5918.1.S1_at	134.1991	520.2982	-3.87706	AT4G04955.1	2.00E-89	ATALN (Arabidopsis allantoinase); allantoinase/ hydrolase
Cit.2304.1.S1_at	22.42456	87.63995	-3.90821	AT2G36090.1	5.00E-11	F-box family protein
Cit.11911.1.S1_at	97.7197	382.7788	-3.91711	AT1G65450.1	1.00E-100	transferase family protein
Cit.21518.1.S1_at	40.0523	158.4352	-3.95571		NA	
Cit.10437.1.S1_at	21.37954	84.65044	-3.95941	AT3G61490.2	0	glycoside hydrolase family 28 protein / polygalacturonase (pectinase
Cit.30657.1.S1_s_at	233.2729	925.6509	-3.9681	AT4G36740.1	1.00E-50	ATHB40 (ARABIDOPSIS THALIANA HOMEOBOX PROTEIN 40); DNA bi
Cit.30735.1.S1_at	51.89571	206.3055	-3.97539		NA	
Cit.18430.1.S1_at	33.7439	134.1899	-3.97672		NA	
Cit.13261.1.S1_x_at	379.0439	1543.924	-4.07321		NA	
Cit.30410.1.S1_at	102.6153	418.2812	-4.07621	AT1G28330.4	2.00E-30	DYL1 (DORMANCY-ASSOCIATED PROTEIN-LIKE 1)
Cit.177.1.S1_s_at	619.934	2560.934	-4.13098	AT3G54820.1	9.00E-51	PIP2;5 (PLASMA MEMBRANE INTRINSIC PROTEIN 2;5); water channe
Cit.36633.1.S1_s_at	361.8621	1498.443	-4.14092	AT1G74310.1	1.00E-143	ATHSP101 (ARABIDOPSIS THALIANA HEAT SHOCK PROTEIN 101); AT
Cit.15122.1.S1_at	22.5494	93.69926	-4.15529	AT4G36890.1	6.00E-07	IRX14 (irregular xylem 14); transferase, transferring glycosyl groups
Cit.48.1.S1_s_at	1073.23	4505.743	-4.1983		NA	
Cit.20082.1.S1_x_at	203.8702	866.5505	-4.2505	AT1G75750.1	7.00E-33	GASA1 (GAST1 PROTEIN HOMOLOG 1)
Cit.18430.1.S1_s_at	139.8386	594.5979	-4.25203		NA	
CitAffx.47578.1.S1_at	1387.075	5912.394	-4.26249	NA	NA	NA
Cit.4124.1.S1_at	77.76336	332.3351	-4.27367		NA	

Cit.35876.1.S1_at	33.72355	150.5853	-4.46529		NA	
Cit.10454.1.S1_at	158.3445	709.6924	-4.48195		NA	
Cit.20558.1.S1_at	87.75677	396.1083	-4.51371	AT5G10170.1	3.00E-20	MIPS3 (MYO-INOSITOL-1-PHOSTPATE SYNTHASE 3); binding / cataly
Cit.37795.1.S1_at	25.22221	114.9515	-4.55755		NA	
Cit.9474.1.S1_at	357.9766	1632.098	-4.55923		NA	
Cit.25628.1.S1_at	147.8724	678.227	-4.58657		NA	
Cit.12374.1.S1_at	712.733	3296.952	-4.62579	ATCG01100.1	4.00E-86	NADH dehydrogenase ND1
Cit.25031.1.S1_at	582.9189	2729.448	-4.68238	AT4G24690.1	6.00E-33	ubiquitin-associated (UBA)/TS-N domain-containing protein / octico
Cit.25990.1.S1_at	41.01103	192.6895	-4.69848	AT1G49640.1	5.00E-06	hydrolase
Cit.3494.1.S1_at	24.07858	115.6858	-4.80451	AT3G02700.1	7.00E-91	NC domain-containing protein
Cit.10521.1.S1_at	106.3957	518.4529	-4.87287		NA	
Cit.29299.1.S1_at	74.38498	364.5747	-4.90119	ATMG00030.1	8.00E-30	hypothetical protein
Cit.8473.1.S1_s_at	648.5146	3324.376	-5.12614	AT1G02930.1	6.00E-31	ATGSTF6 (GLUTATHIONE S-TRANSFERASE); copper ion binding / glut
Cit.1887.1.S1_at	73.53335	378.6333	-5.14914	AT1G68090.1	3.00E-90	ANN5; calcium ion binding / calcium-dependent phospholipid bindir
Cit.30448.1.S1_x_at	35.44403	182.5978	-5.15172	AT4G02380.1	5.00E-20	SAG21 (SENESCENCE-ASSOCIATED GENE 21)
Cit.29267.1.S1_s_at	93.51935	484.6416	-5.18226	AT3G44735.1	2.00E-11	PSK1; growth factor
Cit.18008.1.S1_s_at	23.32242	122.9395	-5.2713		NA	
Cit.29299.1.S1_x_at	91.27554	492.3432	-5.39403	ATMG00030.1	8.00E-30	hypothetical protein
Cit.30679.1.S1_at	23.87996	130.3574	-5.45886		NA	
Cit.1871.1.S1_at	56.26654	313.2954	-5.56806	AT4G36040.1	1.00E-24	DNAJ heat shock N-terminal domain-containing protein (J11)
Cit.6199.1.S1_at	66.27157	371.6249	-5.60761	AT4G31985.1	9.00E-25	60S ribosomal protein L39 (RPL39C)
Cit.11003.1.S1_at	46.41935	262.3503	-5.65174	AT1G68300.1	3.00E-40	universal stress protein (USP) family protein
Cit.18221.1.S1_at	24.83667	142.0759	-5.72041		NA	
Cit.31285.1.S1_at	56.14036	322.6185	-5.74664	AT2G18700.1	7.00E-51	ATTPS11; transferase, transferring glycosyl groups
Cit.20204.1.S1_x_at	320.4128	1851.707	-5.77913		NA	
Cit.14606.1.S1_at	30.57436	178.0086	-5.82215	AT1G13120.1	6.00E-28	emb1745 (embryo defective 1745)
Cit.16918.1.S1_at	358.8206	2095.751	-5.84067	AT4G23400.1	1.00E-144	PIP1;5 (PLASMA MEMBRANE INTRINSIC PROTEIN 1;5); water channe
Cit.1497.1.S1_s_at	163.7195	978.4645	-5.97647	AT3G04070.1	1.00E-98	anac047 (Arabidopsis NAC domain containing protein 47); transcript
Cit.2927.1.S1_s_at	118.2969	713.1235	-6.02825	AT5G23810.1	2.00E-96	AAP7; amino acid transmembrane transporter
Cit.26263.1.S1_at	21.42161	135.719	-6.33561	AT4G31940.1	2.00E-39	CYP82C4; electron carrier/ heme binding / iron ion binding / monoo
Cit.9388.1.S1_at	44.56059	292.9593	-6.5744	AT5G61820.1	7.00E-24	FUNCTIONS IN: molecular_function unknown; INVOLVED IN: biologi
Cit.4748.1.S1_at	31.688	208.8619	-6.5912		NA	
Cit.6332.1.S1_at	28.75158	192.6693	-6.70117	AT2G34930.1	5.00E-35	disease resistance family protein
Cit.2730.1.S1_at	21.61951	148.9216	-6.8883	AT5G58350.1	0	WNK4 (WITH NO K (=LYSINE) 4); kinase/ protein kinase
Cit.19979.1.S1_x_at	32.91951	259.6969	-7.88884	AT3G15353.1	4.00E-18	MT3 (METALLOTHIONEIN 3); copper ion binding

Cit.9569.1.S1_at	43.7102	353.4089	-8.08527		NA	
Cit.20360.1.S1_at	94.8652	788.649	-8.31336		NA	
Cit.5970.1.S1_at	43.67003	386.3721	-8.84753	AT5G61430.1	6.00E-92	ANAC100 (ARABIDOPSIS NAC DOMAIN CONTAINING PROTEIN 100);
Cit.29275.1.S1_s_at	35.78273	316.9513	-8.85766	ATMG00030.1	8.00E-30	hypothetical protein
Cit.22984.1.S1_x_at	26.26821	240.4553	-9.15385	AT5G05320.1	6.00E-20	monooxygenase, putative (MO3)
Cit.9570.1.S1_at	27.87625	256.671	-9.20752	AT5G64260.1	1.00E-125	EXL2 (EXORDIUM LIKE 2)
Cit.1496.1.S1_s_at	84.16457	863.0325	-10.2541	AT3G04070.1	1.00E-98	anac047 (Arabidopsis NAC domain containing protein 47); transcript
Cit.9388.1.S1_x_at	50.71954	545.4845	-10.7549	AT5G61820.1	7.00E-24	FUNCTIONS IN: molecular_function unknown; INVOLVED IN: biologi
Cit.13787.1.S1_s_at	15.80027	183.7327	-11.6285	AT2G03200.1	1.00E-50	aspartyl protease family protein
Cit.15073.1.S1_at	56.64073	660.0101	-11.6526	AT2G03200.1	6.00E-48	aspartyl protease family protein
Cit.14555.1.S1_at	20.33723	269.7149	-13.2621	AT2G25940.1	1.00E-146	ALPHA-VPE (alpha-vacuolar processing enzyme); cysteine-type endo
Cit.2739.1.S1_at	22.85097	305.1374	-13.3534		NA	
Cit.3665.1.S1_s_at	36.69034	615.4867	-16.7752	AT1G75750.1	2.00E-30	GASA1 (GAST1 PROTEIN HOMOLOG 1)
Cit.12433.1.S1_at	27.84544	475.8392	-17.0886		NA	
Cit.28480.1.S1_s_at	21.47077	376.158	-17.5195	AT5G49360.1	1.00E-173	BXL1 (BETA-XYLOSIDASE 1); hydrolase, hydrolyzing O-glycosyl comp
Cit.3665.1.S1_at	34.82674	2164.198	-62.1418	AT1G75750.1	2.00E-30	GASA1 (GAST1 PROTEIN HOMOLOG 1)

Supplementary Table 10. RT-qPCR validaiton of microarray data

Probe ID	AGI	E-Score	Arabidopsis annotation	Citrus genotype	Time point	Microarray	qPCR
Cit.15073.1.S1_at	AT2G03200.1	6.00E-48	aspartyl protease	Fallglo	1	-3.88	-6.30
				Fallglo	2	-3.81	-3.33
				Fallglo	3	-11.39	-10.00
				Grapefruit	1	-4.43	-5.00
				Grapefruit	2	-4.66	-5.00
				Grapefruit	3	-1.98	-2.10
				Pineapple	1	-3.31	-3.33
				Pineapple	2	-1.90	-2.00
				Pineapple	3	-11.65	-12.30
Cit.13787.1.S1_s_at	AT2G03200.1	1.00E-50	aspartyl protease	Fallglo	1	-5.00	-5.01
				Fallglo	2	-3.43	-3.33
				Fallglo	3	-10.10	-8.30
				Grapefruit	1	-3.81	-3.33
				Grapefruit	2	-4.06	-5.00
				Grapefruit	3	-4.19	-5.00
				Pineapple	1	-6.95	-6.30
				Pineapple	2	-2.42	-2.50
				Pineapple	3	-11.65	-14.00
Cit.15637.1.S1_at	AT4G28780.1	9.00E-91	GDSL-motif lipase/hydrolase family protein	Fallglo	1	-3.31	-3.60
				Fallglo	2	-2.38	-2.50
				Grapefruit	1	-3.49	-3.33
				Grapefruit	3	-3.92	-3.85
Cit.27555.1.S1_at	AT5G56970.1	1.00E-58	CKX3 (CYTOKININ OXIDASE 3); amine oxidase/ cytokinin dehydrogenase	Fallglo	1	-3.22	-3.60
				Grapefruit	1	-3.79	3.33
Cit.4078.1.S1_at	AT5G50260.1	1.00E-158	cysteine proteinase, putative	Fallglo	1	5.79	6.70
				Fallglo	3	-11.99	-10.00
				Grapefruit	3	-8.06	-9.60
				Pineapple	1	2.95	5.00
Cit.10894.1.S1_s_at	AT5G06760.1	1.00E-48	late embryogenesis abundant group 4 domain-containing protein	Fallglo	3	7.66	3.40
				Pineapple	1	4.75	5.00
				Pineapple	2	13.96	10.00

				Fallglo	1	4.18	4.44
				Fallglo	2	23.90	24.82
Cit.29507.1.S1_s_at	No hits			Pineapple	1	8.87	7.21
				Pineapple	3	4.06	4.00
				Pineapple	1	4.00	3.61
				Fallglo	2	17.00	28.00
Cit.3629.1.S1_s_at	AT1G10070.2	1.00E-156	ATBCAT-2 (ARABIDOPSIS THALIANA BRANCHED-CHAIN AMINO ACID TRANSAMINASE 2); branched-chain-amino-acid transaminase/ catalytic	Pineapple	2	5.00	13.00
				Grapefruit	2	6.00	5.00
				Fallglo	1	4.74	6.57
				Pineapple	1	6.00	5.72
Cit.12810.1.S1_at	AT1G19670.1	5.00E-65	ATCLH1 (ARABIDOPSIS THALIANA CORONATINE-INDUCED PROTEIN 1); chlorophyllase	Fallglo	2	6.00	12.00
				Pineapple	2	5.00	16.00
				Grapefruit	2	5.00	5.00
				Fallglo	3	2.70	3.70
				Fallglo	1	14.40	9.00
				Pineapple	1	9.00	11.00
Cit.17413.1.S1_s_at	AT4G33467.2	1.00E-09	unknown protein	Fallglo	2	17.00	8.00
				Pineapple	2	4.00	9.00
				Grapefruit	2	5.00	13.00
				Fallglo	3	4.20	4.00
				Fallglo	1	10.28	12.27
				Pineapple	1	6.00	6.28
Cit.3665.1.S1_s_at	AT1G75750.1	2.00E-30	GASA1 (GAST1 PROTEIN HOMOLOG 1)	Fallglo	2	9.00	13.00
				Pineapple	2	9.00	12.00
				Grapefruit	2	15.00	14.93
				Fallglo	3	4.20	5.70
				Pineapple	1	11.00	6.42
Cit.39178.1.S1_s_at	AT5G06760.1	1.00E-48	late embryogenesis abundant group 4 domain-containing protein	Pineapple	2	9.00	8.88
				Fallglo	1	14.75	15.00
				Pineapple	1	6.00	8.67
Cit.5970.1.S1_at	AT5G61430.1	6.00E-92	ANAC100 (ARABIDOPSIS NAC DOMAIN CONTAINING PROTEIN 100); transcription factor	Grapefruit	1	1.00	1.18
				Fallglo	2	18.00	18.00
				Pineapple	2	11.00	14.00
				Grapefruit	2	7.00	13.00

				Fallglo	3	12.30	5.00
				Grapefruit	3	-10.00	-5.70
				Pineapple	1	8.00	5.86
Cit.3665.1.S1_at	AT1G75750.1	2.00E-30	GASA1 (GAST1 PROTEIN HOMOLOG 1)	Grapefruit	2	6.00	14.00
				Fallglo	1	6.52	4.00
				Pineapple	1	8.00	6.00
Cit.15458.1.S1_at	AT1G18400.1	1.00E-45	BEE1 (BR Enhanced Expression 1); transcription factor	Pineapple	2		
				Grapefruit	2	5.00	4.00
				Fallglo	1	31.70	31.63
				Pineapple	1	5.00	5.34
Cit.26593.1.S1_at	No hits			Grapefruit	2	9.00	8.00
				Fallglo	1	1.97	1.14
				Pineapple	1	1.00	1.08
Cit.16321.1.S1_at	AT3G04870.2	5.00E-41	ZDS (ZETA-CAROTENE DESATURASE); carotene 7,8-desaturase	Pineapple	2	4.00	4.00
				Fallglo	1	1.08	1.80
				Pineapple	1	1.00	0.97
Cit.38568.1.S1_at	AT5G54510.1	2.00E-35	DFL1 (DWARF IN LIGHT 1); indole-3-acetic acid amido synthetase	Grapefruit	1	1.00	1.18
				Fallglo	2	3.00	1.00
				Pineapple	2	5.00	2.00

Supplementary Table 11. Primers used for RT-qPCR validation

Probe ID	Forward	Reverse
Cit.15073.1.S1_at	TAGGCCGTCAAAGAATACCG	CGACCTCGATCTGGTTCAAT
Cit.4078.1.S1_at	AAGGAAAGTTCCCCAGCAGT	TCCATGATTCAGCTCTGTGC
Cit.15637.1.S1_at	CGTGACACTCGGTGGTAATG	GTCCCCGTCACAATGACTCT
Cit.13787.1.S1_s_at	TAATCGCGAATTCAGCTGTG	TGTCATCTGAGCAGCCAAAG
Cit.27555.1.S1_at	TGGATTATTTGGAAGGCACA	CCTTGTGCAGGGTACTTTGG
Cit.10894.1.S1_s_at	ATGAAGGAATCGGCAGCTAA	CGCTTGCATTCTTCTTTCCT
Cit.29507.1.S1_s_at	CTGAGCACACCATGGAGAAG	TTCTTGGCGAAGGATCTCTG
Cit.3629.1.S1_s_at	GGACAGCTTAGTCGGTACGC	ACTGATCAACGGAGGGTGAC
Cit.12810.1.S1_at	TGGACTTGATCCCTCGATTC	GCAACAAAATGAGCACGAGA
Cit.17413.1.S1_s_at	TAGAAGCAGAGCCGAGGACT	GAAGCTCTTCTTGGCAGTCG
Cit.3665.1.S1_s_at	TCCAAGGCTTTCATTGCTTC	CTCGACGATAACTGGCACCT
Cit.39178.1.S1_s_at	CTGACAGCTACTCGCACTCG	CCCAGTATTGACTCCCATCG
Cit.5970.1.S1_at	AAAAACCAACTGGGTCATGC	CCCGAATGAGCCTAATTTCA
Cit.3665.1.S1_at	CATCGTCCAAGGCTTTCATT	CTCGACGATAACTGGCACCT
Cit.15458.1.S1_at	TGGCAGTGATGTTGGATGTT	GGCCTCCATACCCTTCTCTC
Cit.16321.1.S1_at	CGGGGTTTTGCTCATTTAGA	AGAAAACGTGCAGGCTGATT
Cit.26593.1.S1_at	ACTTTTACCCGGCCTGCTCT	GGGATGAGTATTTCGCTCGT
Cit.38568.1.S1_at	AGAAGTCCTCAGGGTTGGTG	CAGCAAGTTTTGGGTTTGGT

Supplementary Table S12. Genes with differential transcirpt abundance (GDTA) in seedless vs. seedy Fallglo fruits at all three time points.

AGI	Time 1	Time 2	Time 3	BinName[a]	Description
AT4G10960.1	3.29	2.38	2.68	cell wall.precursor synt	Symbols: UGE5 | UGE5 (UDP-D-glucose/UDP-D-galactose 4-epimerase 5);
AT4G03500.1	2.65	2.27	2.06	cell.organisation	ankyrin repeat family protein | chr4:1553092-1556571 FORWARD
AT5G06760.1	63.66	86.26	7.66	development.late emb	late embryogenesis abundant group 4 domain-containing protein / LEA gr
AT5G07050.1	2.35	-2.28	-2.22	development.unspecifi	LOCATED IN: membrane; CONTAINS InterPro DOMAIN/s: Protein of unkno
AT1G01720.1	2.92	2.15	2.44	development.unspecifi	Symbols: ATAF1, ANAC002 | ATAF1; transcription activator/ transcription
AT5G61430.1	14.75	18.11	5.05	development.unspecifi	Symbols: ANAC100, ATNAC5 | ANAC100 (ARABIDOPSIS NAC DOMAIN CON
AT4G37870.1	2.50	2.52	-2.50	gluconeogenesis / glyo	Symbols: PCK1, PEPCK | PCK1 (PHOSPHOENOLPYRUVATE CARBOXYKINASE
AT3G23240.1	3.64	2.85	2.60	hormone metabolism.e	Symbols: ERF1, ATERF1 | ERF1 (ETHYLENE RESPONSE FACTOR 1); DNA bin
AT5G44210.1	5.12	2.33	2.08	hormone metabolism.e	Symbols: ERF9, ATERF9, ATERF-9 | ERF9 (ERF DOMAIN PROTEIN 9); DNA b
AT1G75750.1	8.36	1.25	4.22	hormone metabolism.g	Symbols: GASA1 | GASA1 (GAST1 PROTEIN HOMOLOG 1) | chr1:2844152(
AT3G63010.1	4.55	2.79	2.32	hormone metabolism.g	Symbols: ATGID1B, GID1B | GID1B (GA INSENSITIVE DWARF1B); hydrolase
AT4G21200.1	-2.68	-3.35	-2.60	hormone metabolism.g	Symbols: ATGA2OX8, GA2OX8 | GA2OX8 (GIBBERELLIN 2-OXIDASE 8); gibl
AT1G30370.1	2.39	2.09	-2.16	lipid metabolism.lipid c	lipase class 3 family protein | chr1:10719169-10720758 REVERSE
AT5G01600.1	-2.39	-2.16	-2.00	metal handling.binding	Symbols: ATFER1, FER1 | ATFER1; ferric iron binding / iron ion binding | cl
AT3G57270.1	2.72	2.63	-2.26	misc.beta 1,3 glucan hy	Symbols: BG1 | BG1 (BETA-1,3-GLUCANASE 1); catalytic/ cation binding /
AT4G37370.1	4.98	4.72	2.40	misc.cytochrome P450	Symbols: CYP81D8 | CYP81D8; electron carrier/ heme binding / iron ion b
AT1G23740.1	-2.79	-2.93	-2.02	misc.oxidases - copper,	oxidoreductase, zinc-binding dehydrogenase family protein | chr1:839811
AT3G06880.1	-2.24	2.03	2.19	not assigned.no ontolo	nucleotide binding | chr3:2170589-2175686 REVERSE
AT1G14870.1	2.70	-2.05	-2.46	not assigned.no ontolo	FUNCTIONS IN: molecular_function unknown; INVOLVED IN: response to (
AT5G06570.2	6.88	3.95	4.92	not assigned.no ontolo	hydrolase | chr5:2007990-2011041 REVERSE
AT1G60420.1	4.55	2.26	2.83	not assigned.no ontolo	DC1 domain-containing protein | chr1:22261888-22264408 FORWARD
AT1G60460.3	-2.56	2.05	2.10	not assigned.unknown	unknown protein | chr1:22275484-22279007 FORWARD
AT5G05250.1	2.16	4.33	3.03	not assigned.unknown	unknown protein | chr5:1557037-1557998 REVERSE
AT4G32330.3	3.02	-2.16	2.13	not assigned.unknown	FUNCTIONS IN: molecular_function unknown; INVOLVED IN: biological_pr
AT3G55240.1	3.27	3.03	2.84	not assigned.unknown	Overexpression leads to PEL (Pseudo-Etiolation in Light) phenotype. | chr:
AT2G26070.1	3.40	2.34	2.10	not assigned.unknown	Symbols: RTE1 | RTE1 (REVERSION-TO-ETHYLENE SENSITIVITY1) | chr2:11
AT1G49640.1	3.51	-10.03	3.39	not assigned.unknown	hydrolase | chr1:18375538-18376729 REVERSE
AT1G02070.1	3.86	2.62	-2.00	not assigned.unknown	unknown protein | chr1:370257-370967 REVERSE
AT3G03341.1	4.57	3.40	2.06	not assigned.unknown	unknown protein | chr3:790130-790648 REVERSE
AT2G38680.1	2.69	-5.10	5.40	nucleotide metabolism	5'-nucleotidase/ magnesium ion binding | chr2:16174011-16176080 REVE

AT2G03200.1	-4.37	-3.61	-10.66	protein.degradation.as	aspartyl protease family protein | chr2:966451-967918 REVERSE
AT5G50260.1	5.11	2.11	-11.99	protein.degradation.cy	cysteine proteinase, putative | chr5:20455317-20457029 FORWARD
AT1G01250.1	4.06	3.82	2.28	RNA.regulation of tran	AP2 domain-containing transcription factor, putative | chr1:104491-1053
AT1G74890.1	-2.61	-2.11	-2.30	RNA.regulation of tran	Symbols: ARR15 | ARR15 (RESPONSE REGULATOR 15); transcription regul
AT3G49940.1	10.13	7.91	3.24	RNA.regulation of tran	Symbols: LBD38 | LBD38 (LOB DOMAIN-CONTAINING PROTEIN 38) | chr3:
AT4G38960.1	2.91	6.78	2.11	RNA.regulation of tran	zinc finger (B-box type) family protein | chr4:18161353-18163303 FORW
AT1G07530.1	2.17	2.28	2.36	RNA.regulation of tran	Symbols: SCL14, ATGRAS2, GRAS2 | SCL14 (SCARECROW-LIKE 14); transcri
AT2G38470.1	2.08	2.12	2.33	RNA.regulation of tran	Symbols: WRKY33, ATWRKY33 | WRKY33; transcription factor | chr2:161
AT5G54160.1	4.18	-2.03	3.17	secondary metabolism	Symbols: ATOMT1, OMT1 | ATOMT1 (O-METHYLTRANSFERASE 1); caffeat
AT5G39670.1	9.08	2.97	-2.14	signalling.calcium	calcium-binding EF hand family protein | chr5:15883179-15884067 FORW
AT1G73500.1	2.51	2.64	2.29	signalling.MAP kinases	Symbols: ATMKK9, MKK9 | MKK9 (MAP KINASE KINASE 9); MAP kinase ki
AT4G21380.1	2.42	2.12	2.24	signalling.receptor kin	Symbols: ARK3 | ARK3 (A. THALIANA RECEPTOR KINASE 3); kinase/ transm
AT3G12500.1	2.57	5.13	2.92	stress.biotic	Symbols: ATHCHIB, PR3, PR-3, CHI-B, B-CHI | ATHCHIB (ARABIDOPSIS THA
AT3G04720.1	3.28	5.05	3.10	stress.biotic	Symbols: PR4, HEL, PR-4 | PR4 (PATHOGENESIS-RELATED 4); chitin bindin
AT1G19670.1	4.74	5.76	2.25	stress.biotic	Symbols: ATCLH1, CORI1, ATHCOR1 | ATCLH1 (ARABIDOPSIS THALIANA C
AT3G54420.1	5.12	3.93	-2.08	stress.biotic	Symbols: ATEP3, ATCHITIV, CHIV | ATEP3; chitinase | chr3:20145910-201
AT5G23810.1	6.05	4.57	2.32	transport.amino acids	Symbols: AAP7 | AAP7; amino acid transmembrane transporter | chr5:80
AT1G61800.1	4.49	2.78	2.46	transport.metabolite t	Symbols: GPT2 | GPT2; antiporter/ glucose-6-phosphate transmembrane

[a] BinName used in MapMan software.

Supplementary Table S13. GDTA in seedless vs. seedy Grapefruits at all three time points.

AGI	Time 1	Time 2	Time 3	BinName[a]	Description
AT4G25810.1	-2.27382	3.994822	-2.85255	cell wall.modification	Symbols: XTR6, XTH23 | XTR6 (XYLOGLUCAN ENDOTRANSGLYCOSYLA
AT5G06760.1	-2.84189	65.10298	4.673151	development.late embryogen	late embryogenesis abundant group 4 domain-containing protein / L
AT4G27410.2	-2.25874	2.235123	-2.27837	development.unspecified	Symbols: RD26 | RD26 (RESPONSIVE TO DESICCATION 26); transcript
AT3G42170.1	2.440413	2.079316	2.917533	DNA.unspecified	DNA binding | chr3:14320952-14324069 FORWARD
AT4G37870.1	-2.18629	-2.32525	-7.04994	gluconeogenesis / glyoxylate c	Symbols: PCK1, PEPCK | PCK1 (PHOSPHOENOLPYRUVATE CARBOXYKI
AT1G53310.3	1.415169	2.011432	1.705472	glycolysis.cytosolic branch.ph	Symbols: ATPPC1 | ATPPC1 (PHOSPHOENOLPYRUVATE CARBOXYLAS
AT3G14440.1	-2.19743	2.210709	-2.43519	hormone metabolism.abscisic	Symbols: NCED3, ATNCED3, STO1, SIS7 | NCED3 (NINE-CIS-EPOXYCA
AT4G37580.1	-5.73808	8.907352	2.874905	hormone metabolism.ethylen	Symbols: HLS1, COP3, UNS2 | HLS1 (HOOKLESS 1); N-acetyltransferas
AT1G75750.1	-2.87693	2.279712	-1.08529	hormone metabolism.gibbere	Symbols: GASA1 | GASA1 (GAST1 PROTEIN HOMOLOG 1) | chr1:284
AT2G18700.1	-2.93893	3.497808	-3.15297	minor CHO metabolism.trehal	Symbols: ATTPS11, TPS11, ATTPSB | ATTPS11; transferase, transferri
AT3G26330.1	-3.11589	3.062882	-3.51011	misc.cytochrome P450	Symbols: CYP71B37 | CYP71B37; electron carrier/ heme binding / iro
AT2G45550.1	-2.55075	1.264389	-2.94003	misc.cytochrome P450	Symbols: CYP76C4 | CYP76C4; electron carrier/ heme binding / iron i
AT2G29420.1	-2.04744	2.469673	-5.98485	misc.glutathione S transferase	Symbols: ATGSTU7, GST25 | ATGSTU7 (ARABIDOPSIS THALIANA GLU
AT4G38540.1	-2.68009	2.515736	-8.81197	misc.oxidases - copper, flavon	monooxygenase, putative (MO2) | chr4:18023121-18025027 FORWA
AT1G71695.1	-3.25732	2.077575	-2.86327	misc.peroxidases	peroxidase 12 (PER12) (P12) (PRXR6) | chr1:26964249-26966688 FO
AT1G69840.6	-3.7341	2.591906	2.07725	not assigned.no ontology	band 7 family protein | chr1:26293660-26295834 REVERSE
AT2G34260.1	-2.42385	2.134572	2.409718	not assigned.no ontology	transducin family protein / WD-40 repeat family protein | chr2:1446
AT4G15520.1	-2.24998	-2.1961	2.005647	not assigned.no ontology	tRNA/rRNA methyltransferase (SpoU) family protein | chr4:8862815-
AT1G49640.1	-3.56047	2.047954	13.96099	not assigned.unknown	hydrolase | chr1:18375538-18376729 REVERSE
AT5G18420.3	-2.38935	2.068624	-2.62499	not assigned.unknown	unknown protein | chr5:6105522-6109385 REVERSE
AT1G31335.1	-2.20815	3.105811	-2.53408	not assigned.unknown	unknown protein | chr1:11216914-11217393 REVERSE
AT5G07330.1	-2.14213	2.652279	2.634844	not assigned.unknown	unknown protein | chr5:2315980-2316874 FORWARD
AT1G48300.1	2.096417	-5.69654	2.174926	not assigned.unknown	unknown protein | chr1:17847356-17848564 FORWARD
AT2G03200.1	-4.09467	-4.65776	-4.18798	protein.degradation.aspartate	aspartyl protease family protein | chr2:966451-967918 REVERSE
AT4G21860.1	-2.24579	5.09981	2.892456	protein.postranslational modi	Symbols: MSRB2 | MSRB2 (methionine sulfoxide reductase B 2); pep
AT3G13960.1	-2.02082	-2.58224	2.175109	RNA.regulation of transcriptio	Symbols: AtGRF5 | AtGRF5 (GROWTH-REGULATING FACTOR 5); trans
AT4G23810.1	-2.34187	-3.58778	-2.06047	RNA.regulation of transcriptio	Symbols: WRKY53, ATWRKY53 | WRKY53; DNA binding / protein binc
AT2G18950.1	-3.01336	2.497496	2.060069	secondary metabolism.isopre	Symbols: HPT1, TPT1, ATHPT, VTE2 | HPT1 (HOMOGENTISATE PHYTY
AT5G54160.1	-2.20439	-2.29676	-1.40689	secondary metabolism.phenyl	Symbols: ATOMT1, OMT1 | ATOMT1 (O-METHYLTRANSFERASE 1); ca
AT5G48930.1	2.382118	2.302273	-4.51134	secondary metabolism.phenyl	Symbols: HCT | HCT (HYDROXYCINNAMOYL-COA SHIKIMATE/QUINAT
AT2G45130.1	-2.55952	-3.25004	2.508622	stress.abiotic	Symbols: ATSPX3, SPX3 | SPX3 (SPX DOMAIN GENE 3) | chr2:186064

AT2G38905.1	-2.52748	2.737264	-2.85215	stress.abiotic.drought/salt	hydrophobic protein, putative / low temperature and salt responsive
AT1G54410.1	-2.23175	1.20618	2.577265	stress.abiotic.unspecified	dehydrin family protein | chr1:20309775-20310767 REVERSE
AT3G54420.1	-9.59475	2.029191	-4.78939	stress.biotic	Symbols: ATEP3, ATCHITIV, CHIV | ATEP3; chitinase | chr3:20145910
AT2G38870.1	-2.41784	2.552764	-2.22677	stress.biotic	protease inhibitor, putative | chr2:16236381-16237280 REVERSE
AT3G04720.1	-2.33034	3.087304	-8.83969	stress.biotic	Symbols: PR4, HEL, PR-4 | PR4 (PATHOGENESIS-RELATED 4); chitin bi
AT4G19040.2	2.066121	2.283174	2.791533	stress.biotic	Symbols: EDR2 | EDR2; lipid binding | chr4:10431520-10437425 REV
AT1G17860.1	-2.16701	3.427829	-1.24208	stress.biotic.PR-proteins.prote	trypsin and protease inhibitor family protein / Kunitz family protein |
AT5G23810.1	-2.17604	2.392876	-6.65791	transport.amino acids	Symbols: AAP7 | AAP7; amino acid transmembrane transporter | chr
AT5G53130.1	-3.09107	2.568003	-3.80494	transport.cyclic nucleotide or	Symbols: CNGC1, ATCNGC1 | CNGC1 (CYCLIC NUCLEOTIDE GATED CH

[a] BinName used in MapMan software.

Supplementary Table S14. GDTA in seedless vs. seedy Pineapple fruits at all three time points

AGI	Time 1	Time 2	Time 3	BinName	Description
AT1G68090.1	-2.05299	-2.67132	-5.14914	cell.organisation	Symbols: ANNAT5, ANN5 | ANN5; calcium ion binding / calcium-depe
AT3G12360.1	4.256664	2.387828	5.165554	cell.organisation	Symbols: ITN1 | ITN1 (INCREASED TOLERANCE TO NACL); protein bin
AT5G06760.1	8.497341	9.956526	4.75	development.late embryogene	late embryogenesis abundant group 4 domain-containing protein / L
AT5G61430.1	6.127765	11.2212	-8.84753	development.unspecified	Symbols: ANAC100, ATNAC5 | ANAC100 (ARABIDOPSIS NAC DOMAIN
AT1G75750.1	4.752233	2.195933	-9.64722	hormone metabolism.gibberel	Symbols: GASA1 | GASA1 (GAST1 PROTEIN HOMOLOG 1) | chr1:284
AT3G45140.1	5.369623	2.293297	2.117697	hormone metabolism.jasmona	Symbols: LOX2, ATLOX2 | LOX2 (LIPOXYGENASE 2); lipoxygenase | ch
AT2G38540.1	5.078586	-2.2391	-2.06066	lipid metabolism.lipid transfer	Symbols: LP1, LTP1, ATLTP1 | LP1; calmodulin binding | chr2:161303
AT3G26220.1	3.46957	3.553634	-2.21087	misc.cytochrome P450	Symbols: CYP71B3 | CYP71B3; electron carrier/ heme binding / iron i
AT2G30860.1	-2.16642	-2.19325	-2.40119	misc.glutathione S transferase	Symbols: ATGSTF9, GLUTTR, ATGSTF7 | ATGSTF9 (GLUTATHIONE S-T
AT4G33467.2	8.855031	4.103793	-3.47307	not assigned.no ontology	unknown protein | chr4:16101464-16102196 REVERSE
AT1G23040.1	4.205695	2.48362	-3.27703	not assigned.no ontology.hydr	hydroxyproline-rich glycoprotein family protein | chr1:8164959-816
AT5G43050.1	-5.32584	-2.62505	-2.85456	not assigned.unknown	unknown protein | chr5:17268025-17269024 FORWARD
AT1G49640.1	-4.18541	13.65404	-4.69848	not assigned.unknown	hydrolase | chr1:18375538-18376729 REVERSE
AT1G06475.1	-2.90803	-2.26869	-2.03354	not assigned.unknown	unknown protein | chr1:1973834-1974386 REVERSE
AT5G61820.1	3.028094	3.531639	-8.16041	not assigned.unknown	FUNCTIONS IN: molecular_function unknown; INVOLVED IN: biologic
AT2G03200.1	-4.48739	-2.41546	-11.6526	protein.degradation.aspartate	aspartyl protease family protein | chr2:966451-967918 REVERSE
AT5G47550.1	-4.92725	-5.27255	-2.45915	protein.degradation.cysteine p	cysteine protease inhibitor, putative / cystatin, putative | chr5:1928
AT4G05320.4	-2.38855	2.819288	2.155645	protein.degradation.ubiquitin.	Symbols: UBQ10 | UBQ10 (POLYUBIQUITIN 10); protein binding | chr
AT3G04920.1	4.494521	2.587981	-2.44417	protein.synthesis.ribosomal pr	40S ribosomal protein S24 (RPS24A) | chr3:1360882-1362295 FORW
AT3G04070.1	5.009401	3.191768	-7.55159	RNA.regulation of transcriptio	Symbols: anac047 | anac047 (Arabidopsis NAC domain containing pr
AT3G21420.1	3.064598	2.737215	-2.29647	secondary metabolism.flavonc	oxidoreductase, 2OG-Fe(II) oxygenase family protein | chr3:7541477
AT3G22840.1	-2.32992	2.438719	-2.04181	signalling.light	Symbols: ELIP1, ELIP | ELIP1 (EARLY LIGHT-INDUCABLE PROTEIN); chl
AT4G11650.1	4.470329	3.252705	-3.00379	stress.abiotic	Symbols: ATOSM34 | ATOSM34 (osmotin 34) | chr4:7024856-702614
AT4G19040.2	2.722	2.280273	2.484757	stress.biotic	Symbols: EDR2 | EDR2; lipid binding | chr4:10431520-10437425 REV
AT3G04720.1	4.235935	2.735847	-2.40317	stress.biotic	Symbols: PR4, HEL, PR-4 | PR4 (PATHOGENESIS-RELATED 4); chitin bi
AT5G41800.1	3.897064	2.974645	-2.14297	transport.amino acids	amino acid transporter family protein | chr5:16733773-16735991 FC
